# Metabolomics of Ramadan fasting: an opportunity for the controlled study of physiological responses to food intake

**DOI:** 10.1186/1479-5876-12-161

**Published:** 2014-06-06

**Authors:** Sweety Mathew, Susanne Krug, Thomas Skurk, Anna Halama, Antonia Stank, Anna Artati, Cornelia Prehn, Joel A Malek, Gabi Kastenmüller, Werner Römisch-Margl, Jerzy Adamski, Hans Hauner, Karsten Suhre

**Affiliations:** 1 Department of Physiology and Biophysics, Weill Cornell Medical College – Qatar, Doha, Qatar; 2 Else Kröner-Fresenius-Centre for Nutritional Medicine, Klinikum rechts der Isar, Technische Universität München, München, Germany; 3 ZIEL - Research Centre for Nutrition and Food Sciences, Technische Universität München, Freising-Weihenstephan, Germany; 4 Institute of Experimental Genetics, Genome Analysis Center, Helmholtz Zentrum München, German Research Center for Environmental Health, Neuherberg, Germany; 5 Institute of Bioinformatics and Systems Biology, Helmholtz Zentrum München, German Research Center for Environmental Health, Neuherberg, Germany; 6 Genomics Core, Weill Cornell Medical College – Qatar, Doha, Qatar; 7 German Center for Diabetes Research, Neuherberg, Germany; 8 Lehrstuhl für Experimentelle Genetik, Technische Universität München, Freising-Weihenstephan, Germany

**Keywords:** Metabolomics, Nutritional challenging, Ramadan fasting, Study design, Clinical research

## Abstract

High-throughput screening techniques that analyze the metabolic endpoints of biological processes can identify the contributions of genetic predisposition and environmental factors to the development of common diseases. Studies applying controlled physiological challenges can reveal dysregulation in metabolic responses that may be predictive for or associated with these diseases. However, large-scale epidemiological studies with well controlled physiological challenge conditions, such as extended fasting periods and defined food intake, pose logistic challenges. Culturally and religiously motivated behavioral patterns of life style changes provide a natural setting that can be used to enroll a large number of study volunteers. Here we report a proof of principle study conducted within a Muslim community, showing that a metabolomics study during the Holy Month of Ramadan can provide a unique opportunity to explore the pre-prandial and postprandial response of human metabolism to nutritional challenges. Up to five blood samples were obtained from eleven healthy male volunteers, taken directly before and two hours after consumption of a controlled meal in the evening on days 7 and 26 of Ramadan, and after an over-night fast several weeks after Ramadan. The observed increases in glucose, insulin and lactate levels at the postprandial time point confirm the expected physiological response to food intake. Targeted metabolomics further revealed significant and physiologically plausible responses to food intake by an increase in bile acid and amino acid levels and a decrease in long-chain acyl-carnitine and polyamine levels. A decrease in the concentrations of a number of phospholipids between samples taken on days 7 and 26 of Ramadan shows that the long-term response to extended fasting may differ from the response to short-term fasting. The present study design is scalable to larger populations and may be extended to the study of the metabolic response in defined patient groups such as individuals with type 2 diabetes.

## Introduction

The accurate diagnosis of a diseased state relies on the integration of best research evidence with clinical expertise and patient data [1]. Clinical parameters that are used to describe the health condition of a patient are often defined as concentrations of specific metabolites and (regulatory) proteins that reflect the dynamics of disease-relevant biochemical pathways in the body. Standard clinical chemistry assays such as for glucose, insulin and triglyceride levels have long been used for the diagnosis and medical management of metabolic diseases and related co-morbidities [2,3]. However, research in the past decades have shown that many common diseases are rather heterogeneous in many aspects, suggesting an urgent need for better tools facilitating disease prediction and diagnostics.

Metabolomics, a high-throughput research approach that is based on the profiling of the small molecule (metabolite) composition of an organism, offers a comprehensive view of ideally all disease-relevant biochemical pathways and may potentially meet the need for better diagnostic data [4]. The composition of the small molecules (metabolites below 1500 Da) in a biological system is determined by its genetic features, the regulation of gene expression, protein abundance and environmental influences [5]. Metabolomics has its value in the proximity to the molecular phenotype of a biological system [6] and, therefore, is frequently applied to characterize the status of an organism under specific conditions [7,8]. The recent implementation of metabolomics in human cohort studies [9-11] has already revealed novel biomarkers for numerous diseases [12-15]. The advantages of metabolomic approaches have already been reported in the identification of disease states or the monitoring of therapy efficiency [16,17].

However, the metabolome is continuously changing due to the multi-parametric response of the human body to different molecular interactions, caused by food intake, gut microbial metabolism, physical activity and other intrinsic as well as extrinsic factors [18]. The metabolic markers that associate with complex diseases may not always be manifest under “standard” conditions, such as overnight fasting. Some molecules strongly fluctuate after food intake and therefore should be examined in the fasting state. Other molecules show abnormal concentrations as a consequence of metabolic disorders only after food ingestion or other physiological challenges [19,20]. Postprandial dysmetabolism is increasingly recognized as a risk factor for disease development and it is apparent that traditional overnight-fasting blood draws may not always be appropriate and sufficient for total risk assessment [21]. New strategies capturing the metabolic plasticity are needed.

To understand the dynamic pattern of the human metabolome under challenge conditions, implementation of multiple time-point sampling is required [22]. However, the majority of metabolomics-based experiments to date were performed only after overnight fasting or under un-controlled conditions. Implementation of an experimental set-up with prolonged fasting times and controlled food-intake that enables monitoring of time-resolved changes in the postprandial metabolome is a challenging task and requires several logistic considerations [23,24]. This is especially true when a large number of study participants needs to be enrolled. Despite these challenges, this approach may promise an improvement in the identification of metabolic dysfunctions indicating early disease states that remain undetected in the fasting state and so should be developed.

Here, we propose Ramadan fasting as a natural setting to conduct physiological challenge experiments in large populations under controlled conditions. During the Holy Month of Ramadan, all around the world, Muslims are fasting for a period of 28 days between dawn (Suhoor) and sunset (Iftar), abstaining from all eating, drinking, and smoking for typically 13–18 hours daily. This makes Ramadan fasting a more intense fast than the usual overnight fast. After the Iftar prayer, the fast is often broken in the community, which may allow for the coordinated enrollment of study participants and also for the administration of a controlled meal. Moreover, since many Muslim patients with diabetes adhere to Ramadan fasting, such a study may also provide information from diseased individuals under otherwise inaccessible challenge conditions. Eventually, such a study could also help to better understand how individuals with common metabolic diseases can be best managed under Ramadan fasting conditions.

In order to study the feasibility of a metabolic challenge test during Ramadan fasting we conducted a proof-of-principle study with the support of the Islamic Centre in Freimann, Munich (Germany). The main objective of our study was to obtain a better understanding of the logistical and cultural factors that need to be addressed in a religiously sensitive setting, to explore which metabolic alterations occur under pre-prandial and postprandial conditions of a prolonged day-time fast in comparison to an overnight fast, and to investigate which effect sizes may be expected using a comprehensive targeted metabolomics analysis. These data will help inform power calculations and preparation of full-fledged Ramadan metabolomics studies.

## Materials and methods

### Study design

**Figure 1:**
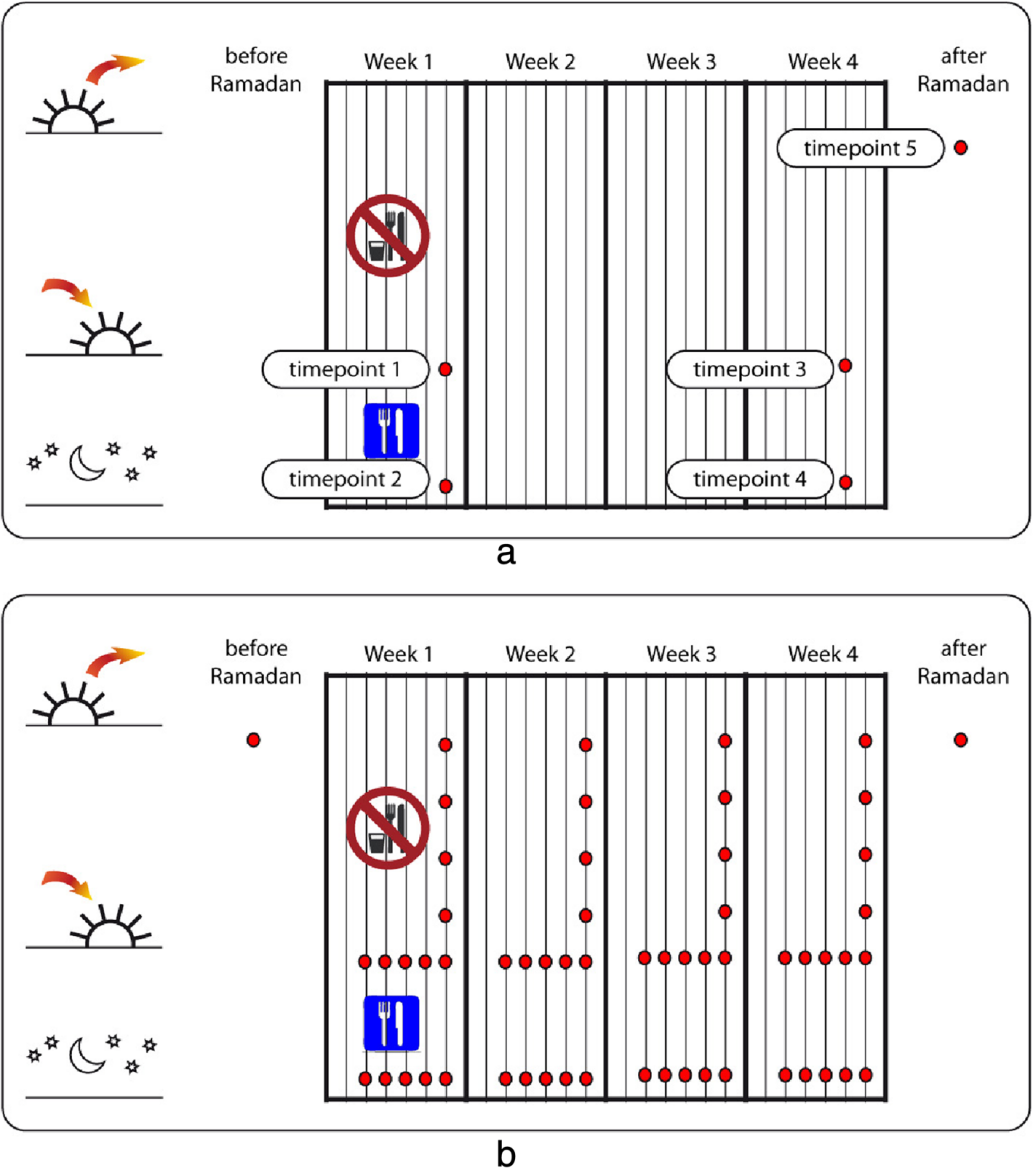
**Study design (a) of the here presented pilot study, (b) of a potential future full-fledged Ramadan study.** Red dots indicate possible time points for sample collection.

The Ramadan fasting study was conducted as a pilot study to the HuMet study [22]. The HuMet study protocol was approved by the ethical committee of the Technische Universität München (#2087/08). This study conformed with the Declaration of Helsinki. All study participants gave written informed consent. A total of eleven healthy male Muslim volunteers of varying BMI, age and nationality were enrolled at the Islamic Centre Munich in 2009. The study design is illustrated in Figure 1a. For the fast-breaking, study participants shared all the same meal in a joint cantina at the mosque. Trays with identical, pre-composed meals of substantial size were dispensed to all study participants. Some variation in food supply may have occurred, since for logistical reasons we could not verify that participants did not share some food with other members of the community who were not participating in the study. However, we estimate that this effect was minimal. Meal compositions and nutrient analysis are provided in Additional file 1: Table S1. Blood samples were collected before and after the fast-breaking at day 7 (time points 1 and 2) and day 26 (time points 3 and 4) from individuals who fasted from dawn to sunset during the month of Ramadan in 2009. A time interval of two hours between the pre- and postprandial sample collections (shortly before and after the meal) was applied. An additional blood sample was collected 4–8 weeks after Ramadan (time point 5) after an overnight fast. Venous blood samples were collected into 9 ml EDTA K_2_-Gel tubes (Sarstedt, Nümbrecht, Germany), immediately centrifuged at 2,500 g for 10 min at 20°C. The obtained plasma was then aliquoted, frozen on dry ice and stored at −80°C until further analysis. Because this study was designed to be a proof-of-principle study, and also because we were unsure about how far we would interfere with the religious proceedings by doing so, no further data was collected from the study participants. However, we found that the community was very open to this kind of study, so that collecting additional phenotype data is not expected to pose any major obstacles in future studies.

### Clinical biochemistry analysis

The clinical biochemistry analysis was performed as previously described for the HuMet study [22]. Briefly, glucose and lactate concentrations were determined by using the *Super GL easy*^+^ (Dr. Müller Geräte Bau, Freital, Germany) based on an enzymatic amperometric technique. Insulin level was measured by enzyme-linked immunosorbent assay (ELISA) (Dako #K6219, Glostrup, Denmark). The plasma concentration of triglycerides and non-esterified fatty acids (NEFAs) was measured using an enzymatic colorimetric method (Fluitest TG, Analyticon Biotechnologies AG, Lichtenfels, Germany) and (NEFA-HR, Wako Chemicals GmbH, Neuss, Germany), respectively. All of the above listed methods were used according to the manufacturer’s guidelines.

### Metabolomics analysis

The targeted metabolomics assay Absolute*IDQ*™ p180 (BIOCRATES Life Sciences AG, Innsbruck, Austria) was applied for metabolite measurements in plasma samples. Sample preparation and metabolite detection was performed according to the Absolute*IDQ*™ assay kit instructions as previously described [25,26]. Metabolite detection was performed by FIA – MS/MS (for carnitines and lipids) and LC – MS/MS (for amino acid and biogenic amines) on an API 4000 (AB Sciex) triple quadrupole mass spectrometer. Data evaluation was performed using the Met*IDQ*™ software package, which is part of the Absolute*IDQ*™ kit. The Absolute*IDQ*™ p180 assay enables the detection of 186 metabolites including: 40 acylcarnitines (free carnitine – C0 and acylcarnitines – Cx:y), 21 amino acids, 19 biogenic amines, 90 glycerophospholipids including lysophosphatidylcholines (LysoPC a Cx:y) and phosphatidylcholines with acyl (PC aa Cx:y) or ether (PC ae Cx:y) side chain, hexoses (H1) and 15 sphingolipids (SM Cx:y). As previously described [25,26], the nomenclature used for lipid metabolites refers to the Lipid Maps comprehensive classification system [27]. The nomenclature for lipids is as follows: Cx:y, where “x” denotes the number of carbons (C) and “y” represents the number of double bonds. The protocol used for the analysis of bile-acids composition in the plasma samples was implemented as follows: In-house protocols were used for the analysis of bile-acids composition in the plasma samples. Plasma samples of 100 μl in Eppendorf tube were spiked with labeled internal standards d4-deoxycholic acid, d4-glycochenodeoxycholic acid, d4-glycocholic acid and d4-lithocholic acid (all internal standards were purchased from CDN Isotopes, Dr. Ehrendorfer) to yield concentrations of 50 ng internal standards/ ml plasma. After well mixing, the samples were transferred into a 96-well plate. In addition to samples from this study, 5 pooled human reference plasma samples and a series of 6 calibration samples with a concentration range of 5 to 150 ng bile acids/ ml plasma were also pipetted into the 96-well plate. The calibration samples were prepared by spiking certain concentrations of 12 bile acids standards (cholic acid, ursodeoxycholic acid, chenodeoxycholic acid, deoxycholic acid, lithocholic acid, glycocholic acid, glycochenodeoxycholic acid, glycodeoxycholic acid, taurocholic acid, taurochenodeoxycholic acid, taurodeoxycholic acid and taurolithocholic acid; all bile acids standards were purchased from Sigma-Aldrich) and labeled internal standards (50 ng/ml plasma) into human plasma-free endogenous bile acids. Bile acid standards and labeled internal standards used to spike the plasma samples were dissolved in 50% v/v methanol containing 0.012% formic acid and 5 mM ammonium acetate. These calibration samples were applied to develop a calibration curve for analyte quantifications. Human reference plasma samples, treated like the samples of this study, served as technical replicates throughout the data set to assess process variability. Besides the above mentioned samples, 100 μL of eluent buffer and 100 μl of plasma-free endogenous bile acids were also pipetted into the 96-well plate to serve as process blanks. Proteins were precipitated and bile acids were extracted from the samples with 1 ml of acetonitrile. After centrifugation, 800 μl of supernatant was transferred into another 96-well plate for evaporation in vacuum evaporator (Uniequip). Bile acids were analyzed with 4000 Q-Trap MS (AB Sciex) equipped with LC (Shimadzu) for chromatographic separation. Prior to analysis, the dried samples were reconstituted with 150 μl of 50% v/v methanol containing 0.012% formic acid and 5 mM ammonium acetate. The MS was operated in the ion spray ionization mode. Data were acquired and processed using Analyst 1.5.1 software (AB Sciex) and IntelliQuan MQll (AB Sciex) for the quantitative processing. The measurement was performed in negative ion mode with tandem MS via collision activated dissociation (CAD) with nitrogen as collision gas. Quantitative data were acquired in the multiple reaction monitoring (MRM) modes with specific precursor to product ion parameters for each analyte. Chromatographic separation was achieved using a reverse-phase C18 column, Onyx Monolithic, 130 Å pore size, 100 × 4.6 mm internal diameter (Phenomenex). After injection of the 40 μl sample extract, the column was developed with mobile phase of 10% methanol containing 0.012% formic acid and 5 mM ammonium acetate (eluent A) and 90% methanol with 0.012% formic acid and 5 mM ammonium acetate (eluent B) in a gradient of 30% eluent A to 95% eluent B in 10 min run time, and then at constant eluent of 95% B for 2 min before it gradually eluted to 70% eluent B. The total run time was 15 min at 1 ml/min flow rate. At analysis, the column temperature was maintained constant at 30°C and the auto sample temperature was at 4°C.

### Statistical analysis

Statistical analysis was done using R version 3.0.2 (R Core Team (2013). R: A language and environment for statistical computing. R Foundation for Statistical Computing, Vienna, Austria. URL http://www.R-project.org/). Linear mixed effects models using log-scaled data were fitted to the difference (MDIFF) between data of paired samples from same individuals, before and after fasting-break, and matched time-points of week 1 and 4 of Ramadan, respectively, using the lme4 package in R (Douglas Bates, Martin Maechler, Ben Bolker and Steven Walker (2014). lme4: Linear mixed-effects models using Eigen and S4. R package version 1.1-5. http://CRAN.R-project.org/package=lme4). As fixed effect we entered the intercept and as random effect we used the participant identifier (PID) (model: MDIFF ~ 1 + (1|PID)). Significance was determined using the chi-square statistic from an ANOVA, using a null model with no intercept and the participant identifier as random effect (null model: MDIFF ~ 0 + (1|PID)). We hence test the hypothesis that the difference between the log-scaled metabolite concentrations is different from zero, which is equivalent to testing the hypothesis that the fold change of the metabolite concentrations is different from unity. Deviation from normality of the residuals was tested using the Shapiro-Wilk test. In cases where a deviation from normality was observed (p < 0.05) we conducted subsequently a paired Wilcoxon rank sum test (also known as ‘Mann-Whitney’ test). In Tables 1 and 2 we report all nominally significant associations (p < 0.05). In order to control the false discovery rate (FDR), we apply the Benjamini and Hochberg approach (as described in [28]) and focus the discussion on associations that have a FDRbelow 5%. The full association data is provided as Additional file 2: Table S2 and Additional file 3: Table S3.

**Table 1:** **Metabolites with significant differences after and before fast**-**breaking**

**Metabolite**	**N**	**Fold change**	**p-value**	**Test**	**Significance**
Insulin [U/L]	18	10.66	2.7x10^−8^	Mixed model	Bonf
Non-esterferied fatty acids [mmol/L]	18	−3.067	9.8x10^−6^	Mixed model	Bonf
Taurochenodeoxycholic acid	18	5.493	0.00020	Mixed model	Bonf
Triglycerides [mg/dl]	18	1.187	0.00026	Mixed model	FDR
Glycochhenodeoxycholic acid	18	3.823	0.00026	Mixed model	FDR
Spermidine	17	−1.181	0.00039	Mixed model	FDR
Taurocholic acid	18	4.751	0.00053	Wilcoxon	FDR
Putrescine	17	−1.473	0.00064	Mixed model	FDR
Glycodeoxycholic acid	15	5.961	0.00074	Mixed model	FDR
Glycocholic acid	18	5.199	0.00090	Mixed model	FDR
C14:2	18	−1.572	0.0016	Wilcoxon	FDR
C3	18	1.179	0.0016	Wilcoxon	FDR
Lactate [mg/dl]	18	1.560	0.0017	Mixed model	FDR
C10	18	−1.361	0.0023	Wilcoxon	FDR
C8	18	−1.238	0.0023	Wilcoxon	FDR
Glutamate	17	1.247	0.0030	Mixed model	FDR
C16:2	18	−1.420	0.0033	Mixed model	sig
H1	18	1.226	0.0035	Mixed model	sig
C10:1	18	−1.193	0.0040	Wilcoxon	sig
C14:1	18	−1.133	0.0040	Wilcoxon	sig
C14:2-OH	18	−1.189	0.0045	Wilcoxon	sig
Taurodeoxycholic acid	14	6.006	0.0056	Mixed model	sig
Arginine	17	1.202	0.0061	Mixed model	sig
Met-SO	13	2.511	0.0066	Mixed model	sig
Alanine	17	1.253	0.0067	Wilcoxon	sig
Ornithine	17	1.199	0.0074	Mixed model	sig
Proline	17	1.220	0.0084	Mixed model	sig
alpha-AAA	17	1.424	0.0094	Mixed model	sig
lysoPC a C16:0	18	1.087	0.012	Mixed model	sig
Lysine	17	1.201	0.012	Mixed model	sig
Leucine	17	1.162	0.012	Mixed model	sig
Phenylalanine	17	1.167	0.012	Mixed model	sig
Asparagine	17	1.197	0.018	Mixed model	sig
C7-DC	18	−1.190	0.021	Mixed model	sig
C0	18	1.071	0.021	Mixed model	sig
Tyrosine	17	1.139	0.022	Mixed model	sig
Isoleucine	17	1.178	0.023	Mixed model	sig
C14:1-OH	18	−1.122	0.023	Mixed model	sig
lysoPC a C17:0	18	1.103	0.026	Wilcoxon	sig
C16:1	18	−1.067	0.027	Wilcoxon	sig
PC aa C36:4	18	1.046	0.028	Mixed model	sig
C12	18	−1.244	0.034	Mixed model	sig
Tryptophan	17	1.126	0.040	Mixed model	sig
PC aa C38:5	18	1.046	0.041	Mixed model	sig
PC aa C38:4	18	1.046	0.042	Mixed model	sig
C16:1-OH	18	1.119	0.042	Mixed model	sig
Valine	17	1.137	0.043	Mixed model	sig
Citrulline	17	−1.156	0.050	Mixed model	sig

N is the number of matched pairs that entered the analysis. Fold change is the ratio between the metabolite concentrations after and before the fasting-break, based on the estimate of the mixed model. The reported (uncorrected) p-values are those of the mixed model if the Shapiro test for normality of the residual is non-significant (p > 0.05), and that of the Wilcoxon rank test otherwise. The significance level of the association is indicated: significant after Bonferroni correction (Bonf), at a false discovery rate of 5% (FDR), nominal at p < 0.05 (sig).

**Table 2:** Metabolites that display significant differences between week 4 and week 1 of Ramadan

**Metabolite**	**N**	**Fold change**	**p-value**	**Test**	**Significance**
PC aa C36:6	16	1.221	9.2x10^−5^	Wilcoxon	Bonf
Histamine	16	−1.028	0.0031	Wilcoxon	FDR
PC ae C38:0	16	1.154	0.0063	Wilcoxon	FDR
PC aa C36:0	16	1.121	0.0076	Wilcoxon	FDR
Serotonin	16	1.109	0.0083	Mixed model	FDR
SM C26:1	16	1.182	0.0086	Mixed model	sig
C3-DC (C4-OH)	16	−1.269	0.010	Mixed model	sig
PC aa C36:1	16	1.193	0.013	Mixed model	sig
PC aa C28:1	16	1.087	0.022	Mixed model	sig
C16:2-OH	16	−1.143	0.026	Mixed model	sig
PC ae C32:2	16	1.089	0.031	Mixed model	sig
Chenodeoxycholic acid	16	2.014	0.031	Mixed model	sig
PC ae C40:6	16	1.083	0.039	Wilcoxon	sig
PC ae C38:2	16	1.116	0.040	Mixed model	sig
PC ae C38:1	16	1.203	0.040	Mixed model	sig
Ursodeoxycholic acid	15	−1.905	0.043	Mixed model	sig
PC ae C36:0	16	1.094	0.044	Mixed model	sig
PC aa C38:3	16	1.107	0.046	Mixed model	sig
PC ae C34:0	16	1.108	0.046	Mixed model	sig
SM (OH) C16:1	16	1.080	0.050	Mixed model	sig

Fold change is the ratio between the metabolite concentrations observed at both time points of week 4 and week 1, based on the estimate of the mixed model (see Table 1 for full legend).

## Results and discussion

### Changes in clinical biochemistry during Ramadan

**Figure 2:**
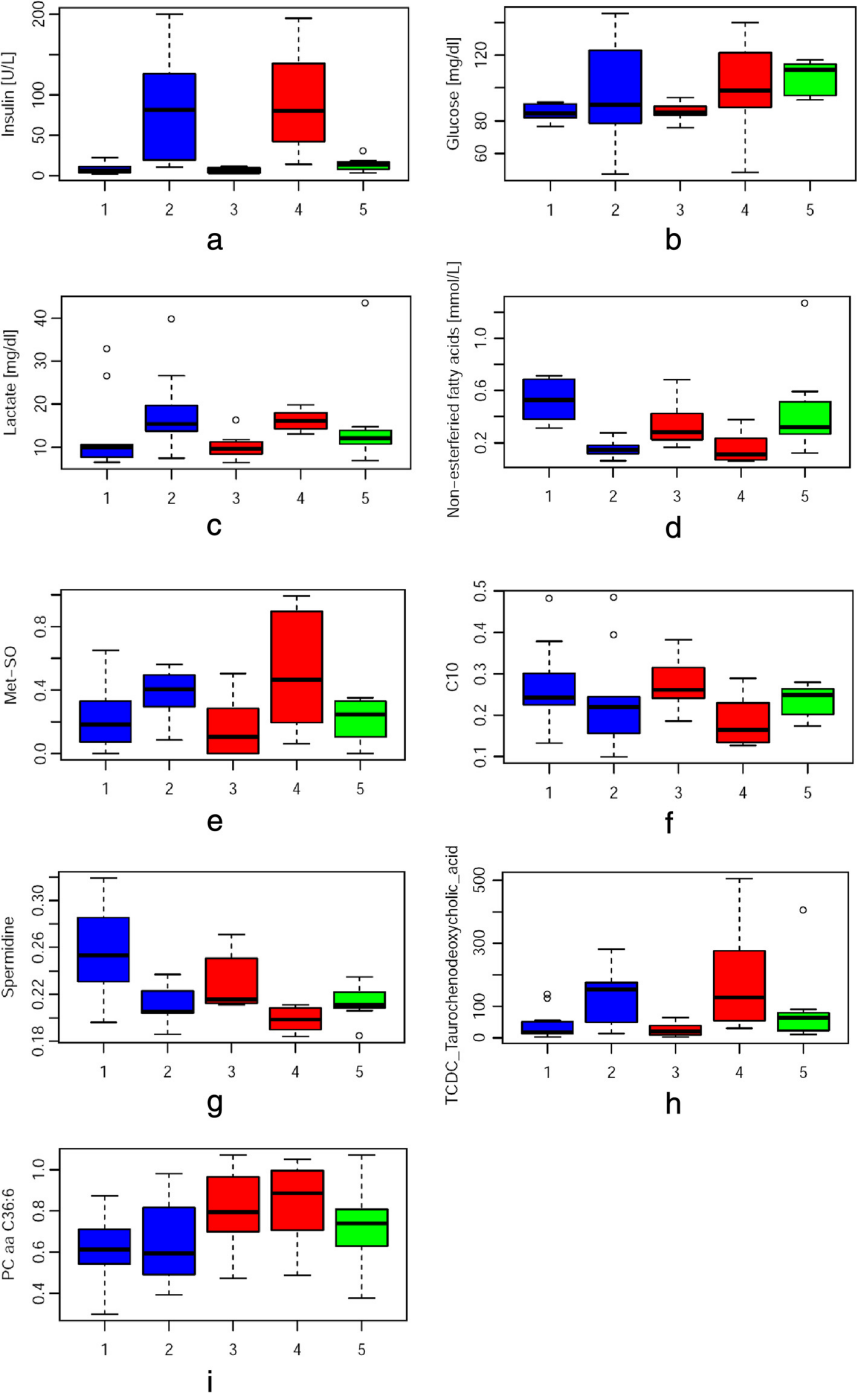
**Selected metabolites that change during fast-breaking (a**-**h) and between week one and four (i).** Bile acids are given in ng/ml all other metabolites are given in μM. Time points of sampling are presented on the x-axis and are defined as follows: 1 + 2: collected during first week of Ramadan (blue), 3 + 4 collected during last fourth of Ramadan (red), 5 collected several weeks after Ramadan (green); 1 + 3 fasting between dawn and sun set, 2 + 4 after fast-breaking with identical meal, 5 overnight fasting (for plots of all measured parameters see Additional file 4: Figure S1).

Readouts from clinical biochemistry parameters and selected metabolites are presented in Figure 2. The corresponding p-values of associations are reported in Table 1. In accordance with the fasting state, glucose, lactate and insulin levels were low before fast break (time points 1 and 3) but increased and showed much larger variability after food intake (time points 2 and 4). In agreement with the expected more intense fasting during Ramadan, insulin and glucose levels at both Ramadan fasting time points were also lower than those observed after the overnight fast (time point 5). Levels of non-esterified fatty acids (NEFAs) at both Ramadan fasting time points were significantly higher compared to time points after food ingestion. Our results are in good accordance with the previously reported NEFA fluctuation after an oral glucose tolerance test and a meal tolerance test were applied [29]. In the fasting state, NEFAs are released from triacylglycerol stored in adipocytes (lipolysis), thereby increasing in plasma [30]. Consequently, increased postprandial insulin concentrations induce a decrease in NEFA levels by rapid suppression of lipolysis [31]. In the present study, we also observed this correlation between the elevated plasma insulin levels after ingestion of food and the decrease in concentrations of plasma NEFAs in the postprandial state. Plasma triglyceride levels increased after food intake, but only slightly on day 7 of Ramadan (time points 1 and 2) and moderately on day 26 (time points 3 and 4). Previous studies report triglyceride levels that doubled and reached its peak around three hours after ingestion [32]. In our study we did not observe such a strong effect, which may in part be due to differences in sampling time in our study and that of Coppack *et al*. [32], and also in part to difference in the meal composition. Overall, we find that the observed changes in levels of insulin, glucose, lactate, NEFAs and triglycerides are coherent with the pre-prandial and postprandial conditions of the eleven subjects, especially when considering the varying BMI, age and ethnicity of the participants. These observations thus demonstrate the reliability of our experimental setup.

### Metabolomic changes associated with food intake

To monitor the global metabolic events occurring in the body during Ramadan fast-breaking we applied targeted metabolomics approaches for the detection and quantification of a total of 202 metabolites. Individual data points for all measured variables (202 metabolites and 6 parameters determined using clinical chemistry methods) are provided in Additional file 4: Figure S1. At a nominal significance level of 0.05, the levels of 48 variables differed significantly between pre- and postprandial state (Table 1). These metabolites belong to different metabolic classes: amino acids, bile acids, acylcarnitines, and polyamines. To illustrate the direction of changes at different time points, the data were visualized in the form of box-plots (Figure 2) and line plots (Additional file 4: Figure S1). The levels of many amino acids increased after food consumption, including asparagine, arginine, alanine, glutamate, proline, and phenylalanine. Methionine sulfoxide (Met-SO), which is an oxidation product of methionine with reactive oxygen species (ROS) and has been suggested as a biomarker for oxidative stress [33], also showed a strong increase. Methionine oxidation is part of a highly regulated machinery, controlling the function of signaling factors under physiological challenge conditions and conditions of oxidative stress [34]. When the amino acids levels at time points 1 and 3 were compared with the overnight fasting situation at time point 5, asparagine, arginine and phenylalanine exhibited similar levels, in contrast to proline and alanine, which after overnight fasting were comparable to the post-prandial state in Ramadan. Our observations are in good agreement with a previous study that reported a decrease in amino acids levels in both plasma and urine samples of subjects after a prolonged fasting period of 36 h [35].

Under the postprandial conditions, the glycine/taurine conjugated bile acids showed increased levels, whereas in the pre-prandial state at time points 1 and 3 their concentrations were similar to those measured after on overnight fast. Bile acids are produced from cholesterol in the liver and stored in the gall bladder. The gall bladder contracts in the postprandial state and releases bile acids into the intestine, which are reabsorbed through the enterohepatic circulation [36,37]. Thus, the postprandial plasma bile acid levels are determined by the balance between intestinal input and hepatic clearance. Bile acid levels were shown to increase rapidly within 30 minutes after meal intake in the HuMet study [22] and are in agreement with our expectations.

Not all metabolites were up-regulated after food ingestion. A significant decrease in the concentration of long-chain acyl-carnitines was visible in the postprandial stage. The decrease in long-chain acyl-carnitine levels was expected since carnitine-bound fatty acids are imported into the mitochondria for beta-oxidation and represent an energy-source under fasting conditions. Consequently, decreases in long-chain acyl-carnitine levels reflect a switch of energy metabolism from fatty acid to glucose oxidation in agreement with the altered substrate availability in the postprandial state. We also observed a decrease in the levels of the polyamines spermidine and putrescine after food ingestion. Dietary polyamines are absorbed shortly after a meal by the small intestine and degraded in the gut before reaching systemic circulation [38]. However, recently an association between fasting plasma glucose and total polyamine concentration was reported [39], which suggests a fluctuation in polyamine concentrations as a response to the nutrition stimuli. Taken together, the observed changes in the different metabolic classes reflect the expected physiological response of the human body to the break of the fasting state.

### Long-term changes in the metabolome during Ramadan fasting

We also studied the influence of long-term fasting on the human metabolic profile by comparing samples taken before fast breaking on week 1 to those on week 4, and those taken after fast breaking on week 1 to those of week 4, respectively. Overall, these changes displayed a lower level of significance than the changes observed with respect to fast-breaking. Twenty metabolites were significantly changed between week 1 and 4 of Ramadan, many of which were phosphatidylcholines (PCs) (Table 2). Many of these PCs are composed of long-chain fatty acids (C12-C18) and polyunsaturated omega-3 and omega-6 fatty acids (C18:2 and above). PC levels in the fourth week of Ramadan were higher than PC levels at the start of Ramadan, while PC levels at the start of Ramadan were very similar to those observed after an overnight fast. A recent study in a cohort of 173 families in Saudi Arabia showed that approximately two thirds of the respondents (59.5%) reported weight gain after Ramadan [40]. Hence, some of the changes observed in these metabolites may be attributed to a change in nutrition regimen during Ramadan. Our results clearly suggest that differences between the effects of short- and long- term fasting, changes in nutrition pattern, and adaptation of human metabolism to prolonged fasting conditions can be studied in a sufficiently powered metabolomics study during Ramadan.

### Potential for a full-fledged Ramadan study design

Our study was conducted in a small and heterogeneous group of eleven healthy male individuals with varying BMI, age and ethnicity and, therefore has considerable limitations. Nevertheless, the experimental design (Figure 1a) we applied in this proof-of-principle study already revealed sound metabolic signatures of the pre- and post-prandial stages as well as the effect of longer-term fasting (several weeks). Implementation of a full-fledged research study during the month of Ramadan and collection of additional phenotype information of the participants may reveal valuable information on body adaptation and the plasticity of metabolic control regarding anabolic/catabolic conditions. Countries with a large proportion of Muslims in their population provide an excellent opportunity for such studies. An example of a sample collection schedule is illustrated in Figure 1b. Time series may be collected along the month of Ramadan and also during a single day, e.g. respecting prayer times in order to reliably sample blood from study participants at different time points. Different depths of sampling density may be considered, including use of blood spots rather than venipuncture, and enrolling participants from the medical community at large clinical centers for more dense sampling protocols. These individuals may be more easily available for frequent blood sampling. Local communities often organize joint Iftar festivities in order to break the fast collectively. Such a set-up can be used for future research studies since the food consumption can then be monitored and drawing of samples from volunteers be made at dedicated mobile sampling and storage units. Furthermore, sample collection procedures can be simplified and conducted by the individual at home or work environment via blood spotting on filter paper after finger prick. Metabolic measurements in dried blood spots (DBS), covering a panel of 42 metabolites including acyl-carnitines and amino acids, were already applied in the clinic to screen infants for metabolic diseases [41]. The panel of metabolites detected from DBS can be extended to 188 small molecules as previously described [42]. Such a simplified sampling procedure could also be used in order to increase the number of samples taken during the day or to allow volunteers taking samples by themselves during the day without the requirement of venipuncture.

One aspect that may need specific consideration is circadian rhythm. During the Ramadan time the sleep patterns of some individuals may interchange day and night activities. Therefore, it is extremely important to conduct the study in a well controlled group of participants, where such changes are documented and ideally measured. Moreover, it may be important to document changes in overall activity pattern, since many Muslims use the Ramadan period to take vacation and visit family. Notwithstanding, this pilot study represents a starting basis for the design of a large-scale Ramadan metabolomics study to address the important issue of how cultural and religious fasting may affect metabolism and disease risk in a large group of individuals with variable preconditions.

### Potential for clinical studies

Recently, obesity defined by the World Health Organization (WHO) as abnormal or excessive fat accumulation that represents a risk of health [43], reached an epidemic level by affecting nearly half a billion of the world’s population [44]. Obesity is recognized as a major risk factor of chronic diseases, especially cardiovascular disease, hyperglycemia [45], and type 2 diabetes, [46]. In Arab countries, the prevalence of overweight, obesity and type 2 diabetes increased drastically over the last three decades [47]. Therefore, implementation of a metabolomics study during the month of Ramadan in countries of dominantly Muslim culture, such as the Gulf countries, offers the possibility to monitor large groups of patients with morbid obesity and diabetes under strictly controlled conditions. A large-scale population study on the metabolic regulation during the month of Ramadan opens several possibilities to explore not only the metabolic plasticity in healthy individuals, but also to monitor subjects diagnosed with complex diseases. The experimental set-up can reveal novel biomarkers for these diseases, which might only be unmasked under physiological challenge. Furthermore, this study can also be used for classification of nutrients required for energy production and for investigating the proper functioning of the human body under prolonged fasting in both healthy and diabetic individuals. The importance of dietary guidelines to control and reduce the global disease burden including diet-related complex chronic diseases has already been emphasized [48].

Last but not least, we like to draw attention to the advantages of combining metabolomics and genome-wide association studies (GWAS) in the Arab population during the month of Ramadan. The capability of GWAS in the identification of genes associated with type 2 diabetes, cardiovascular disease, as well as with clinically relevant intermediate traits, such as cholesterol and triglyceride levels has already been reported [49-51]. The first large-scale metabolomics project including genome wide association study data (mGWAS) [52] revealed relations between loci such as FADS1, ELOVL2 or SLC16A9 and changes in specific lipid species that are products and substrates of these enzymes and transporters. The advantages of applying metabolomics together with GWAS to overcome the limitations of GWAS-alone studies was recently discussed [53,54]. The physiological and metabolic consequences, with respect to genetic variation analysis, triggered by such an extreme change in dietary habits (Ramadan) have not been studied before. Subsequently, by controlling for dietary patterns during the fasting month, we shall be able to investigate the effect of genetic variations in combination with various defined nutritional intakes (gene-environment interaction) on the metabolic profile of the subjects.

## Conclusions

By conducting a proof of principle study conducted within a Muslim community we show that a metabolomics study during the Holy Month of Ramadan represents a natural setting that allows to enroll large numbers of study volunteers. Albeit based on a small number of individuals, the here observed changes in metabolite levels are in agreement with previously reported physiological response to food intake. The Ramadan study design thus provides a unique opportunity that allows to explore the pre-prandial and postprandial response of human metabolism to nutritional challenges, especially in defined patient groups that are otherwise difficult to access under challenge conditions, such as individuals with type 2 diabetes. We anticipate that based on the experience gained in this initial small-scale study, future epidemiology-scale studies on human metabolism under nutritional challenge conditions shall be conducted.

## Competing interests

The authors declare that they have no competing interest.

## Authors’ contributions

HH and KS designed the study. SK, TS, AA, CP, WRM, and JA conducted the experiments. SK, AS and KS analyzed the data. JAM and GK contributed to the concept of the Ramadan study. SM, SK, AH and KS wrote the manuscript. All authors read and approved the final manuscript.

## Supplementary Material

Additional file 1: Table S1 Meal composition. Click here for file

Additional file 2: Table S2 Association data comparing metabolites before and after fast-breaking (full association dataset, for legend see Table 1). Click here for file

Additional file 3: Table S3 Association data comparing metabolites between week 1 and week 4 of Ramadan (full association dataset, for legend see Table 2). Click here for file

Additional file 4: Figure S1 Clinical biochemistry and metabolomics data. Time points of sampling are presented on the x-axis and are defined as follows: 1 + 2: collected during the first week of Ramadan, 3 + 4: collected during the last week of Ramadan, 5: collected several weeks after Ramadan; Fasting state: 1 + 3: Ramadan fasting, 2 + 4: after fast breaking with identical meal, 5: overnight fasting. Left: data presented as boxplots; right: data presented as scatterplots, colored by participant, point before/after fast breaking are connected for each individual, red lines: increase in metabolite concentrations, blue line: decrease during fast breaking. Click here for file
