# Supplementary material for: Metabolomics of Ramadan fasting: an opportunity for the controlled study of physiological responses to food intake
Source: J Transl Med. 2014 Jun 6;12:161. doi: 10.1186/1479-5876-12-161 (PMC4063233; doi:10.1186/1479-5876-12-161)
Supplement: Additional file 1: Table S1 — Meal composition. [file 1479-5876-12-161-S1.doc]

**Additional file 1: Table S1:** Meal composition

|  |  |  |  |  |  |  |
| --- | --- | --- | --- | --- | --- | --- |
| **Week 1** | |  |  |  |  |  |
| **Amount** | | **Ingredient** |  | **Nutritient composition:** |  |  |
| 100 | g | White rice (cooked) |  | Calories | 4594 kJ | #### |
| 50 | g | Egg pasta |  | Protein | 63 g | 23.3 |
| 150 | g | Chicken meat (breast) |  | Total Fat | 45 g | 38.2 |
| 50 | g | Bell pepper (cooked) |  | Total Carbohydrate | 104 g | 38.5 |
| 50 | g | Avocado |  | Alcohol | 0 g | - |
| 20 | g | Whipping cream (30 % fat) |  |  |  |  |
| 200 | ml | Orange juice (without additives) |  |  |  |  |
| 100 | g | Italian salad |  |  |  |  |
| 100 | g | Rice pudding |  |  |  |  |
| 40 | g | Pita bread |  |  |  |  |
| 200 | ml | Vegetable soup |  |  |  |  |
|  |  |  |  |  |  |  |
| **Week 4** | |  |  |  |  |  |
| **Amount** | | **Ingredient** |  | **Nutritient composition:** |  |  |
| 120 | g | White rice (cooked) |  | Calories | 5536 kJ | #### |
| 15 | g | Raisin |  | Protein | 65 g | 19.9 |
| 15 | g | Hazelnut |  | Total Fat | 60 g | 42.3 |
| 150 | ml | Yoghurt (3,5 % fat) |  | Total Carbohydrate | 123 g | 37.8 |
| 40 | g | Pita bread |  | Alcohol | 0 g | - |
| 200 | ml | Orange juice (without additives) |  |  |  |  |
| 200 | g | Lamb muscular meat with intermuscular fat |  |  |  |  |
| 200 | ml | Lentil soup (instant) |  |  |  |  |
| 13 | g | White bread with grains |  |  |  |  |
| 100 | g | Italian salad |  |  |  |  |
