# Supplementary material for: Metabolomics of Ramadan fasting: an opportunity for the controlled study of physiological responses to food intake
Source: J Transl Med. 2014 Jun 6;12:161. doi: 10.1186/1479-5876-12-161 (PMC4063233; doi:10.1186/1479-5876-12-161)
Supplement: Additional file 2: Table S2 — Association data comparing metabolites before and after fast-breaking (full association dataset, for legend see Table 1). [file 1479-5876-12-161-S2.doc]

**Additional file 2: Table S2: Association data comparing metabolites before and after fast-breaking**

| **metabolite** | **N** | **fold change** | **p-value** | **test** | **significance** |  |  |
| --- | --- | --- | --- | --- | --- | --- | --- |
| Insulin [U/L] | 18 | 10.660 | 2.7E-08 | Mixed model | bonf |  |  |
| Non-esterferied fatty acids [mmol/L] | 18 | -3.069 | 9.8E-06 | Mixed model | bonf |  |  |
| TCDC_Taurochenodeoxycholic_acid | 18 | 5.493 | 0.00020 | Mixed model | bonf |  |  |
| Triglycerides [mg/dl] | 18 | 1.187 | 0.00026 | Mixed model | FDR |  |  |
| GCDC_Glycochhenodeoxycholic_acid | 18 | 3.823 | 0.00026 | Mixed model | FDR |  |  |
| Spermidine | 17 | -1.180 | 0.00039 | Mixed model | FDR |  |  |
| TC_Taurocholic_acid | 18 | 4.751 | 0.00053 | Wilcoxon | FDR |  |  |
| Putrescine | 17 | -1.474 | 0.00064 | Mixed model | FDR |  |  |
| GDC_Glycodeoxycholic_acid | 15 | 5.961 | 0.00074 | Mixed model | FDR |  |  |
| GC_Glycocholic_acid | 18 | 5.199 | 0.00090 | Mixed model | FDR |  |  |
| C14:2 | 18 | -1.573 | 0.0016 | Wilcoxon | FDR |  |  |
| C3 | 18 | 1.179 | 0.0016 | Wilcoxon | FDR |  |  |
| Lactate [mg/dl] | 18 | 1.560 | 0.0017 | Mixed model | FDR |  |  |
| C10 | 18 | -1.360 | 0.0023 | Wilcoxon | FDR |  |  |
| C8 | 18 | -1.238 | 0.0023 | Wilcoxon | FDR |  |  |
| Glu | 17 | 1.247 | 0.0030 | Mixed model | FDR |  |  |
| C16:2 | 18 | -1.420 | 0.0033 | Mixed model | sig |  |  |
| H1 | 18 | 1.226 | 0.0035 | Mixed model | sig |  |  |
| C10:1 | 18 | -1.194 | 0.0040 | Wilcoxon | sig |  |  |
| C14:1 | 18 | -1.132 | 0.0040 | Wilcoxon | sig |  |  |
| C14:2-OH | 18 | -1.189 | 0.0045 | Wilcoxon | sig |  |  |
| TDC_Taurodeoxycholic_acid | 14 | 6.006 | 0.0056 | Mixed model | sig |  |  |
| Arg | 17 | 1.202 | 0.0061 | Mixed model | sig |  |  |
| Met-SO | 13 | 2.511 | 0.0066 | Mixed model | sig |  |  |
| Ala | 17 | 1.253 | 0.0067 | Wilcoxon | sig |  |  |
| Orn | 17 | 1.199 | 0.0074 | Mixed model | sig |  |  |
| Pro | 17 | 1.220 | 0.0084 | Mixed model | sig |  |  |
| alpha-AAA | 17 | 1.424 | 0.0094 | Mixed model | sig |  |  |
| lysoPC a C16:0 | 18 | 1.087 | 0.012 | Mixed model | sig |  |  |
| Lys | 17 | 1.201 | 0.012 | Mixed model | sig |  |  |
| Leu | 17 | 1.162 | 0.012 | Mixed model | sig |  |  |
| Phe | 17 | 1.167 | 0.012 | Mixed model | sig |  |  |
| Asn | 17 | 1.197 | 0.018 | Mixed model | sig |  |  |
| C7-DC | 18 | -1.191 | 0.021 | Mixed model | sig |  |  |
| C0 | 18 | 1.071 | 0.021 | Mixed model | sig |  |  |
| Tyr | 17 | 1.139 | 0.022 | Mixed model | sig |  |  |
| Ile | 17 | 1.178 | 0.023 | Mixed model | sig |  |  |
| C14:1-OH | 18 | -1.123 | 0.023 | Mixed model | sig |  |  |
| lysoPC a C17:0 | 18 | 1.103 | 0.026 | Wilcoxon | sig |  |  |
| C16:1 | 18 | -1.068 | 0.027 | Wilcoxon | sig |  |  |
| PC aa C36:4 | 18 | 1.046 | 0.028 | Mixed model | sig |  |  |
| C12 | 18 | -1.244 | 0.034 | Mixed model | sig |  |  |
| Trp | 17 | 1.126 | 0.040 | Mixed model | sig |  |  |
| PC aa C38:5 | 18 | 1.046 | 0.041 | Mixed model | sig |  |  |
| PC aa C38:4 | 18 | 1.046 | 0.042 | Mixed model | sig |  |  |
| C16:1-OH | 18 | 1.119 | 0.042 | Mixed model | sig |  |  |
| Val | 17 | 1.137 | 0.043 | Mixed model | sig |  |  |
| Cit | 17 | -1.157 | 0.050 | Mixed model | sig |  |  |
| His | 17 | 1.127 | 0.051 | Wilcoxon | n.s. |  |  |
| PC aa C40:6 | 18 | 1.056 | 0.051 | Mixed model | n.s. |  |  |
| TLC_Taurolithocholic_acid | 10 | 2.954 | 0.059 | Mixed model | n.s. |  |  |
| PC ae C40:4 | 18 | 1.048 | 0.062 | Mixed model | n.s. |  |  |
| Cortisol | 18 | 1.451 | 0.067 | Mixed model | n.s. |  |  |
| PC aa C40:1 | 18 | -1.034 | 0.067 | Wilcoxon | n.s. |  |  |
| CDC_Chenodeoxycholic_acid | 18 | 2.142 | 0.067 | Wilcoxon | n.s. |  |  |
| PC aa C42:4 | 18 | 1.076 | 0.068 | Mixed model | n.s. |  |  |
| C6:1 | 18 | -1.134 | 0.069 | Mixed model | n.s. |  |  |
| C18:1 | 18 | -1.099 | 0.070 | Mixed model | n.s. |  |  |
| lysoPC a C16:1 | 18 | 1.066 | 0.075 | Mixed model | n.s. |  |  |
| Histamine | 17 | -1.015 | 0.078 | Wilcoxon | n.s. |  |  |
| PC ae C38:3 | 18 | 1.043 | 0.087 | Mixed model | n.s. |  |  |
| PC ae C36:1 | 18 | 1.032 | 0.10 | Mixed model | n.s. |  |  |
| PC aa C32:1 | 18 | 1.055 | 0.10 | Wilcoxon | n.s. |  |  |
| Progesterone | 12 | 1.361 | 0.10 | Mixed model | n.s. |  |  |
| C12:1 | 18 | -1.135 | 0.10 | Mixed model | n.s. |  |  |
| Kynurenine | 17 | 1.119 | 0.10 | Mixed model | n.s. |  |  |
| lysoPC a C18:0 | 18 | 1.059 | 0.11 | Mixed model | n.s. |  |  |
| C5 | 18 | 1.093 | 0.11 | Mixed model | n.s. |  |  |
| PC aa C34:2 | 18 | 1.034 | 0.12 | Mixed model | n.s. |  |  |
| C10:2 | 18 | -1.057 | 0.12 | Wilcoxon | n.s. |  |  |
| C2 | 18 | -1.126 | 0.13 | Mixed model | n.s. |  |  |
| C4:1 | 18 | 1.091 | 0.13 | Mixed model | n.s. |  |  |
| C18:2 | 18 | -1.126 | 0.13 | Mixed model | n.s. |  |  |
| Ser | 17 | 1.106 | 0.14 | Mixed model | n.s. |  |  |
| PC ae C44:3 | 18 | 1.067 | 0.14 | Mixed model | n.s. |  |  |
| PC ae C30:2 | 18 | 1.058 | 0.14 | Mixed model | n.s. |  |  |
| PC aa C36:2 | 18 | 1.036 | 0.15 | Mixed model | n.s. |  |  |
| C3-DC (C4-OH) | 18 | 1.057 | 0.15 | Mixed model | n.s. |  |  |
| PC ae C42:4 | 18 | 1.031 | 0.16 | Mixed model | n.s. |  |  |
| Androstenedione | 18 | 1.261 | 0.16 | Mixed model | n.s. |  |  |
| PC aa C38:6 | 18 | 1.049 | 0.17 | Wilcoxon | n.s. |  |  |
| Cortisone | 18 | 1.238 | 0.18 | Mixed model | n.s. |  |  |
| C16:2-OH | 18 | -1.068 | 0.19 | Mixed model | n.s. |  |  |
| C6 (C4:1-DC) | 18 | -1.068 | 0.19 | Mixed model | n.s. |  |  |
| Creatinine | 17 | 1.031 | 0.19 | Mixed model | n.s. |  |  |
| PC aa C36:3 | 18 | 1.035 | 0.19 | Mixed model | n.s. |  |  |
| PC aa C40:3 | 18 | 1.033 | 0.19 | Mixed model | n.s. |  |  |
| PC ae C40:3 | 18 | 1.029 | 0.20 | Mixed model | n.s. |  |  |
| PC ae C40:2 | 18 | 1.034 | 0.22 | Wilcoxon | n.s. |  |  |
| PC aa C24:0 | 18 | 1.084 | 0.22 | Mixed model | n.s. |  |  |
| C9 | 18 | -1.072 | 0.22 | Mixed model | n.s. |  |  |
| Glucose [mg/dl] | 18 | 1.102 | 0.22 | Mixed model | n.s. |  |  |
| PC ae C34:0 | 18 | 1.031 | 0.23 | Mixed model | n.s. |  |  |
| C5:1-DC | 18 | -1.097 | 0.23 | Mixed model | n.s. |  |  |
| PC ae C38:0 | 18 | 1.044 | 0.23 | Mixed model | n.s. |  |  |
| SM C26:0 | 18 | 1.083 | 0.24 | Mixed model | n.s. |  |  |
| ADMA | 17 | 1.090 | 0.24 | Mixed model | n.s. |  |  |
| Met | 17 | 1.064 | 0.24 | Wilcoxon | n.s. |  |  |
| C3-OH | 18 | 1.046 | 0.25 | Mixed model | n.s. |  |  |
| lysoPC a C26:0 | 18 | 1.091 | 0.25 | Mixed model | n.s. |  |  |
| PC ae C42:2 | 18 | 1.036 | 0.25 | Mixed model | n.s. |  |  |
| PC ae C34:1 | 18 | 1.029 | 0.25 | Mixed model | n.s. |  |  |
| PC ae C42:1 | 18 | 1.033 | 0.25 | Mixed model | n.s. |  |  |
| PC aa C32:0 | 18 | 1.027 | 0.26 | Mixed model | n.s. |  |  |
| 3-Hydroxybutyrat [µg/ml] | 18 | -1.074 | 0.26 | Mixed model | n.s. |  |  |
| C5-M-DC | 18 | -1.038 | 0.26 | Mixed model | n.s. |  |  |
| 17OH-Progesterone | 18 | 1.320 | 0.28 | Mixed model | n.s. |  |  |
| LC_Lithocholic_acid | 12 | 2.310 | 0.28 | Mixed model | n.s. |  |  |
| Taurine | 17 | 1.042 | 0.28 | Mixed model | n.s. |  |  |
| PC aa C36:5 | 18 | 1.037 | 0.28 | Wilcoxon | n.s. |  |  |
| Thr | 17 | 1.042 | 0.28 | Wilcoxon | n.s. |  |  |
| CA_Cholic_aci | 18 | 1.284 | 0.29 | Mixed model | n.s. |  |  |
| PC aa C42:0 | 18 | 1.023 | 0.30 | Wilcoxon | n.s. |  |  |
| C4 | 18 | 1.056 | 0.32 | Mixed model | n.s. |  |  |
| PC ae C44:5 | 18 | 1.029 | 0.32 | Mixed model | n.s. |  |  |
| C18:1-OH | 18 | 1.061 | 0.32 | Mixed model | n.s. |  |  |
| UDC_Ursodeoxycholic_acid | 16 | 1.207 | 0.33 | Mixed model | n.s. |  |  |
| Ac-Orn | 12 | 1.244 | 0.33 | Mixed model | n.s. |  |  |
| DC_Deoxycholic_acid | 16 | 1.216 | 0.34 | Mixed model | n.s. |  |  |
| PC aa C28:1 | 18 | 1.028 | 0.34 | Mixed model | n.s. |  |  |
| Corticosterone | 7 | 1.688 | 0.34 | Mixed model | n.s. |  |  |
| C16-OH | 18 | 1.061 | 0.34 | Mixed model | n.s. |  |  |
| PC ae C38:4 | 18 | 1.029 | 0.35 | Mixed model | n.s. |  |  |
| PC aa C34:1 | 18 | 1.043 | 0.36 | Mixed model | n.s. |  |  |
| PC ae C40:5 | 18 | 1.033 | 0.36 | Mixed model | n.s. |  |  |
| total DMA | 17 | 1.029 | 0.37 | Mixed model | n.s. |  |  |
| PC aa C40:4 | 18 | 1.034 | 0.38 | Mixed model | n.s. |  |  |
| Gln | 17 | 1.054 | 0.39 | Mixed model | n.s. |  |  |
| Serotonin | 17 | -1.036 | 0.39 | Mixed model | n.s. |  |  |
| PC ae C36:2 | 18 | 1.041 | 0.39 | Wilcoxon | n.s. |  |  |
| PC ae C40:6 | 18 | 1.030 | 0.39 | Wilcoxon | n.s. |  |  |
| PC ae C32:2 | 18 | 1.029 | 0.41 | Mixed model | n.s. |  |  |
| Gly | 17 | 1.067 | 0.41 | Mixed model | n.s. |  |  |
| PC aa C34:3 | 18 | 1.032 | 0.41 | Mixed model | n.s. |  |  |
| PC ae C42:5 | 18 | 1.027 | 0.41 | Mixed model | n.s. |  |  |
| PC ae C34:2 | 18 | 1.022 | 0.41 | Mixed model | n.s. |  |  |
| PC aa C42:2 | 18 | 1.042 | 0.41 | Mixed model | n.s. |  |  |
| lysoPC a C18:1 | 18 | 1.026 | 0.42 | Mixed model | n.s. |  |  |
| C18 | 18 | 1.019 | 0.43 | Mixed model | n.s. |  |  |
| C14 | 18 | -1.039 | 0.43 | Mixed model | n.s. |  |  |
| PC aa C38:3 | 18 | 1.034 | 0.43 | Mixed model | n.s. |  |  |
| PC ae C38:2 | 18 | 1.028 | 0.43 | Mixed model | n.s. |  |  |
| lysoPC a C24:0 | 18 | 1.051 | 0.44 | Mixed model | n.s. |  |  |
| PC ae C38:5 | 18 | 1.027 | 0.44 | Mixed model | n.s. |  |  |
| SM (OH) C22:2 | 18 | 1.021 | 0.44 | Mixed model | n.s. |  |  |
| C5-OH (C3-DC-M) | 18 | 1.040 | 0.45 | Mixed model | n.s. |  |  |
| PC aa C38:1 | 15 | 1.178 | 0.45 | Mixed model | n.s. |  |  |
| lysoPC a C18:2 | 18 | 1.033 | 0.47 | Mixed model | n.s. |  |  |
| C16 | 18 | -1.032 | 0.47 | Mixed model | n.s. |  |  |
| PC aa C40:5 | 18 | 1.028 | 0.47 | Mixed model | n.s. |  |  |
| PC ae C32:1 | 18 | 1.021 | 0.48 | Mixed model | n.s. |  |  |
| PC aa C32:3 | 18 | 1.021 | 0.49 | Mixed model | n.s. |  |  |
| SM C24:1 | 18 | 1.015 | 0.50 | Mixed model | n.s. |  |  |
| C5:1 | 18 | 1.035 | 0.50 | Mixed model | n.s. |  |  |
| PC ae C42:3 | 18 | 1.024 | 0.50 | Mixed model | n.s. |  |  |
| SM C20:2 | 18 | 1.035 | 0.50 | Mixed model | n.s. |  |  |
| SM C24:0 | 18 | 1.016 | 0.50 | Mixed model | n.s. |  |  |
| SM C26:1 | 18 | 1.036 | 0.50 | Mixed model | n.s. |  |  |
| PC aa C42:1 | 18 | 1.031 | 0.51 | Wilcoxon | n.s. |  |  |
| PC ae C36:4 | 18 | 1.016 | 0.51 | Mixed model | n.s. |  |  |
| PC aa C36:1 | 18 | 1.031 | 0.52 | Wilcoxon | n.s. |  |  |
| PC aa C36:6 | 18 | 1.032 | 0.52 | Wilcoxon | n.s. |  |  |
| PC ae C36:0 | 18 | 1.015 | 0.53 | Mixed model | n.s. |  |  |
| PC ae C34:3 | 18 | 1.016 | 0.53 | Mixed model | n.s. |  |  |
| Testosterone | 18 | 1.121 | 0.55 | Wilcoxon | n.s. |  |  |
| lysoPC a C14:0 | 18 | 1.009 | 0.56 | Mixed model | n.s. |  |  |
| Asp | 17 | 1.044 | 0.57 | Mixed model | n.s. |  |  |
| PC ae C36:5 | 18 | 1.019 | 0.58 | Mixed model | n.s. |  |  |
| SM (OH) C22:1 | 18 | 1.013 | 0.59 | Mixed model | n.s. |  |  |
| PC ae C30:1 | 11 | 1.153 | 0.59 | Mixed model | n.s. |  |  |
| PC aa C42:5 | 18 | 1.020 | 0.59 | Mixed model | n.s. |  |  |
| lysoPC a C28:1 | 18 | 1.024 | 0.59 | Mixed model | n.s. |  |  |
| PC aa C26:0 | 18 | 1.022 | 0.60 | Mixed model | n.s. |  |  |
| SM (OH) C16:1 | 18 | 1.015 | 0.60 | Mixed model | n.s. |  |  |
| SM (OH) C14:1 | 18 | 1.014 | 0.62 | Mixed model | n.s. |  |  |
| SM C18:1 | 18 | 1.020 | 0.62 | Mixed model | n.s. |  |  |
| C5-DC (C6-OH) | 18 | -1.028 | 0.63 | Mixed model | n.s. |  |  |
| PC ae C30:0 | 18 | 1.017 | 0.64 | Mixed model | n.s. |  |  |
| PC ae C36:3 | 18 | 1.013 | 0.64 | Mixed model | n.s. |  |  |
| lysoPC a C28:0 | 18 | 1.029 | 0.65 | Mixed model | n.s. |  |  |
| PC ae C38:6 | 18 | 1.022 | 0.67 | Wilcoxon | n.s. |  |  |
| PC aa C36:0 | 18 | -1.002 | 0.70 | Wilcoxon | n.s. |  |  |
| SM C18:0 | 18 | 1.015 | 0.70 | Mixed model | n.s. |  |  |
| SM C16:1 | 18 | 1.008 | 0.71 | Mixed model | n.s. |  |  |
| lysoPC a C20:4 | 18 | 1.016 | 0.72 | Mixed model | n.s. |  |  |
| PC ae C38:1 | 18 | -1.019 | 0.72 | Mixed model | n.s. |  |  |
| SM C16:0 | 18 | 1.008 | 0.73 | Mixed model | n.s. |  |  |
| C12-DC | 18 | 1.011 | 0.75 | Mixed model | n.s. |  |  |
| PC ae C44:4 | 18 | 1.006 | 0.78 | Wilcoxon | n.s. |  |  |
| PC aa C42:6 | 18 | 1.005 | 0.80 | Mixed model | n.s. |  |  |
| PC aa C32:2 | 18 | -1.008 | 0.81 | Mixed model | n.s. |  |  |
| PC aa C38:0 | 18 | 1.011 | 0.81 | Wilcoxon | n.s. |  |  |
| PC aa C30:0 | 18 | 1.012 | 0.82 | Mixed model | n.s. |  |  |
| PC ae C42:0 | 18 | 1.025 | 0.83 | Wilcoxon | n.s. |  |  |
| DOPA | 10 | -1.009 | 0.85 | Mixed model | n.s. |  |  |
| PC aa C34:4 | 18 | 1.005 | 0.89 | Mixed model | n.s. |  |  |
| PC ae C40:1 | 18 | 1.030 | 0.90 | Wilcoxon | n.s. |  |  |
| PC ae C44:6 | 18 | 1.020 | 0.90 | Wilcoxon | n.s. |  |  |
| SM (OH) C24:1 | 18 | 1.003 | 0.92 | Mixed model | n.s. |  |  |
| PC aa C40:2 | 18 | -1.004 | 0.93 | Mixed model | n.s. |  |  |
| lysoPC a C26:1 | 18 | 1.002 | 0.95 | Mixed model | n.s. |  |  |
| C3:1 | 18 | 1.002 | 0.97 | Mixed model | n.s. |  |  |
| lysoPC a C20:3 | 18 | 1.001 | 0.98 | Mixed model | n.s. |  |  |
| SDMA | 8 | 1.001 | 0.99 | Mixed model | n.s. |  |  |
