# Supplementary material for: Metabolomics of Ramadan fasting: an opportunity for the controlled study of physiological responses to food intake
Source: J Transl Med. 2014 Jun 6;12:161. doi: 10.1186/1479-5876-12-161 (PMC4063233; doi:10.1186/1479-5876-12-161)
Supplement: Additional file 3: Table S3 — Association data comparing metabolites between week 1 and week 4 of Ramadan (full association dataset, for legend see Table 2). [file 1479-5876-12-161-S3.doc]

**Additional file 3:Table S3:** Association data comparing metabolites between week 1 and week 4 of Ramadan

| **metabolite** | **N** | **fold change** | **p-value** | **test** | **significance** |  |  |
| --- | --- | --- | --- | --- | --- | --- | --- |
| PC aa C36:6 | 16 | 1.221 | 9.2E-05 | Wilcoxon | bonf |  |  |
| Histamine | 16 | -1.028 | 0.0031 | Wilcoxon | FDR |  |  |
| PC ae C38:0 | 16 | 1.154 | 0.0063 | Wilcoxon | FDR |  |  |
| PC aa C36:0 | 16 | 1.121 | 0.0076 | Wilcoxon | FDR |  |  |
| Serotonin | 16 | 1.109 | 0.0083 | Mixed model | FDR |  |  |
| SM C26:1 | 16 | 1.182 | 0.0086 | Mixed model | sig |  |  |
| C3-DC (C4-OH) | 16 | -1.269 | 0.010 | Mixed model | sig |  |  |
| PC aa C36:1 | 16 | 1.193 | 0.013 | Mixed model | sig |  |  |
| PC aa C28:1 | 16 | 1.087 | 0.022 | Mixed model | sig |  |  |
| C16:2-OH | 16 | -1.143 | 0.026 | Mixed model | sig |  |  |
| PC ae C32:2 | 16 | 1.089 | 0.031 | Mixed model | sig |  |  |
| CDC_Chenodeoxycholic_acid | 16 | 2.014 | 0.031 | Mixed model | sig |  |  |
| PC ae C40:6 | 16 | 1.083 | 0.039 | Wilcoxon | sig |  |  |
| PC ae C38:2 | 16 | 1.116 | 0.040 | Mixed model | sig |  |  |
| PC ae C38:1 | 16 | 1.203 | 0.040 | Mixed model | sig |  |  |
| UDC_Ursodeoxycholic_acid | 15 | -1.906 | 0.043 | Mixed model | sig |  |  |
| PC ae C36:0 | 16 | 1.094 | 0.044 | Mixed model | sig |  |  |
| PC aa C38:3 | 16 | 1.107 | 0.046 | Mixed model | sig |  |  |
| PC ae C34:0 | 16 | 1.108 | 0.046 | Mixed model | sig |  |  |
| SM (OH) C16:1 | 16 | 1.080 | 0.050 | Mixed model | sig |  |  |
| PC aa C36:5 | 16 | 1.247 | 0.053 | Mixed model | n.s. |  |  |
| Spermidine | 16 | -1.082 | 0.053 | Mixed model | n.s. |  |  |
| PC ae C30:0 | 16 | 1.105 | 0.054 | Mixed model | n.s. |  |  |
| Non-esterferied fatty acids [mmol/L] | 16 | -1.282 | 0.055 | Mixed model | n.s. |  |  |
| PC aa C36:3 | 16 | 1.095 | 0.057 | Mixed model | n.s. |  |  |
| PC aa C36:2 | 16 | 1.115 | 0.062 | Mixed model | n.s. |  |  |
| C9 | 16 | 1.219 | 0.063 | Mixed model | n.s. |  |  |
| PC aa C30:0 | 16 | 1.125 | 0.065 | Mixed model | n.s. |  |  |
| PC ae C40:2 | 16 | 1.083 | 0.069 | Mixed model | n.s. |  |  |
| PC aa C34:3 | 16 | 1.138 | 0.072 | Mixed model | n.s. |  |  |
| 3-Hydroxybutyrat [µg/ml] | 16 | -1.136 | 0.074 | Mixed model | n.s. |  |  |
| Pro | 16 | 1.092 | 0.076 | Mixed model | n.s. |  |  |
| PC ae C42:2 | 16 | 1.088 | 0.080 | Mixed model | n.s. |  |  |
| Glu | 16 | -1.190 | 0.082 | Mixed model | n.s. |  |  |
| PC ae C40:3 | 16 | 1.034 | 0.087 | Mixed model | n.s. |  |  |
| PC ae C32:1 | 16 | 1.052 | 0.090 | Mixed model | n.s. |  |  |
| Testosterone | 16 | 1.173 | 0.090 | Mixed model | n.s. |  |  |
| SM (OH) C14:1 | 16 | 1.064 | 0.098 | Mixed model | n.s. |  |  |
| PC aa C38:0 | 16 | 1.069 | 0.10 | Wilcoxon | n.s. |  |  |
| PC ae C40:1 | 16 | 1.082 | 0.10 | Wilcoxon | n.s. |  |  |
| PC ae C42:5 | 16 | 1.060 | 0.11 | Mixed model | n.s. |  |  |
| PC aa C32:1 | 16 | 1.156 | 0.12 | Wilcoxon | n.s. |  |  |
| PC ae C36:1 | 16 | 1.086 | 0.12 | Mixed model | n.s. |  |  |
| Lactate [mg/dl] | 16 | -1.207 | 0.13 | Wilcoxon | n.s. |  |  |
| PC aa C40:3 | 16 | 1.093 | 0.14 | Mixed model | n.s. |  |  |
| C16:2 | 16 | -1.311 | 0.14 | Mixed model | n.s. |  |  |
| PC aa C42:5 | 16 | 1.129 | 0.14 | Mixed model | n.s. |  |  |
| PC ae C42:3 | 16 | 1.082 | 0.14 | Mixed model | n.s. |  |  |
| PC aa C40:6 | 16 | 1.113 | 0.15 | Mixed model | n.s. |  |  |
| PC aa C40:4 | 16 | 1.104 | 0.15 | Mixed model | n.s. |  |  |
| alpha-AAA | 16 | -1.178 | 0.15 | Mixed model | n.s. |  |  |
| Ala | 16 | 1.086 | 0.15 | Mixed model | n.s. |  |  |
| PC aa C32:3 | 16 | 1.092 | 0.16 | Mixed model | n.s. |  |  |
| SM C20:2 | 16 | -1.108 | 0.16 | Mixed model | n.s. |  |  |
| PC ae C36:4 | 16 | -1.099 | 0.17 | Mixed model | n.s. |  |  |
| PC aa C38:1 | 12 | 1.233 | 0.17 | Mixed model | n.s. |  |  |
| C5:1 | 16 | -1.073 | 0.17 | Mixed model | n.s. |  |  |
| PC ae C30:2 | 16 | 1.059 | 0.17 | Mixed model | n.s. |  |  |
| C5 | 16 | -1.062 | 0.17 | Mixed model | n.s. |  |  |
| PC aa C38:6 | 16 | 1.058 | 0.18 | Wilcoxon | n.s. |  |  |
| PC ae C38:3 | 16 | 1.083 | 0.18 | Mixed model | n.s. |  |  |
| PC aa C40:2 | 16 | 1.118 | 0.18 | Mixed model | n.s. |  |  |
| PC aa C34:1 | 16 | 1.067 | 0.19 | Mixed model | n.s. |  |  |
| PC aa C32:2 | 16 | 1.136 | 0.19 | Mixed model | n.s. |  |  |
| PC ae C44:5 | 16 | 1.075 | 0.19 | Wilcoxon | n.s. |  |  |
| C18:2 | 16 | -1.145 | 0.20 | Mixed model | n.s. |  |  |
| PC ae C34:1 | 16 | 1.050 | 0.20 | Mixed model | n.s. |  |  |
| C14:2 | 16 | -1.219 | 0.20 | Mixed model | n.s. |  |  |
| SM C18:1 | 16 | 1.067 | 0.20 | Mixed model | n.s. |  |  |
| C18:1-OH | 16 | 1.059 | 0.20 | Mixed model | n.s. |  |  |
| Arg | 16 | 1.066 | 0.22 | Mixed model | n.s. |  |  |
| C3 | 16 | 1.065 | 0.23 | Mixed model | n.s. |  |  |
| PC aa C42:1 | 16 | 1.088 | 0.23 | Mixed model | n.s. |  |  |
| PC aa C42:6 | 16 | 1.094 | 0.23 | Wilcoxon | n.s. |  |  |
| lysoPC a C28:0 | 16 | 1.075 | 0.23 | Mixed model | n.s. |  |  |
| PC aa C38:5 | 16 | 1.082 | 0.24 | Mixed model | n.s. |  |  |
| PC aa C40:5 | 16 | 1.090 | 0.24 | Mixed model | n.s. |  |  |
| C2 | 16 | -1.122 | 0.24 | Mixed model | n.s. |  |  |
| DC_Deoxycholic_acid | 14 | 1.285 | 0.25 | Mixed model | n.s. |  |  |
| SM (OH) C22:2 | 16 | 1.040 | 0.25 | Mixed model | n.s. |  |  |
| C14 | 16 | 1.077 | 0.25 | Mixed model | n.s. |  |  |
| PC aa C34:4 | 16 | 1.095 | 0.26 | Mixed model | n.s. |  |  |
| lysoPC a C14:0 | 16 | 1.038 | 0.26 | Mixed model | n.s. |  |  |
| C14:1 | 16 | -1.067 | 0.26 | Mixed model | n.s. |  |  |
| Orn | 16 | -1.060 | 0.27 | Mixed model | n.s. |  |  |
| lysoPC a C18:2 | 16 | 1.072 | 0.27 | Mixed model | n.s. |  |  |
| lysoPC a C20:3 | 16 | 1.070 | 0.28 | Mixed model | n.s. |  |  |
| Thr | 16 | -1.060 | 0.29 | Mixed model | n.s. |  |  |
| lysoPC a C20:4 | 16 | -1.058 | 0.29 | Mixed model | n.s. |  |  |
| C5-DC (C6-OH) | 16 | -1.059 | 0.29 | Wilcoxon | n.s. |  |  |
| DOPA | 9 | 1.048 | 0.29 | Mixed model | n.s. |  |  |
| lysoPC a C18:1 | 16 | 1.069 | 0.30 | Mixed model | n.s. |  |  |
| Val | 16 | 1.045 | 0.30 | Wilcoxon | n.s. |  |  |
| PC aa C34:2 | 16 | 1.038 | 0.30 | Wilcoxon | n.s. |  |  |
| Met | 16 | -1.073 | 0.31 | Mixed model | n.s. |  |  |
| Androstenedione | 16 | 1.108 | 0.32 | Mixed model | n.s. |  |  |
| Ser | 16 | -1.020 | 0.32 | Wilcoxon | n.s. |  |  |
| C6 (C4:1-DC) | 16 | -1.057 | 0.32 | Wilcoxon | n.s. |  |  |
| PC aa C24:0 | 16 | 1.079 | 0.33 | Mixed model | n.s. |  |  |
| Creatinine | 16 | -1.024 | 0.33 | Mixed model | n.s. |  |  |
| GCDC_Glycochhenodeoxycholic_acid | 16 | 1.265 | 0.33 | Mixed model | n.s. |  |  |
| PC ae C42:4 | 16 | -1.026 | 0.33 | Mixed model | n.s. |  |  |
| Ile | 16 | 1.044 | 0.33 | Mixed model | n.s. |  |  |
| SM C16:0 | 16 | 1.026 | 0.34 | Mixed model | n.s. |  |  |
| PC aa C42:2 | 16 | 1.091 | 0.37 | Mixed model | n.s. |  |  |
| C7-DC | 16 | -1.072 | 0.37 | Mixed model | n.s. |  |  |
| SM C18:0 | 16 | 1.052 | 0.37 | Mixed model | n.s. |  |  |
| PC ae C38:5 | 16 | -1.047 | 0.38 | Mixed model | n.s. |  |  |
| lysoPC a C16:1 | 16 | 1.041 | 0.38 | Mixed model | n.s. |  |  |
| PC aa C36:4 | 16 | -1.031 | 0.38 | Mixed model | n.s. |  |  |
| C16 | 16 | -1.053 | 0.39 | Mixed model | n.s. |  |  |
| TC_Taurocholic_acid | 16 | -1.175 | 0.40 | Wilcoxon | n.s. |  |  |
| PC ae C44:6 | 16 | -1.052 | 0.42 | Mixed model | n.s. |  |  |
| SM C16:1 | 16 | 1.025 | 0.42 | Mixed model | n.s. |  |  |
| C16:1 | 16 | -1.043 | 0.42 | Mixed model | n.s. |  |  |
| Cit | 16 | 1.036 | 0.42 | Mixed model | n.s. |  |  |
| PC aa C42:0 | 16 | 1.066 | 0.42 | Mixed model | n.s. |  |  |
| CA_Cholic_aci | 16 | 1.317 | 0.44 | Mixed model | n.s. |  |  |
| PC aa C42:4 | 16 | 1.047 | 0.45 | Mixed model | n.s. |  |  |
| PC aa C26:0 | 16 | 1.055 | 0.46 | Mixed model | n.s. |  |  |
| C3:1 | 16 | -1.047 | 0.46 | Mixed model | n.s. |  |  |
| GDC_Glycodeoxycholic_acid | 13 | 1.330 | 0.47 | Mixed model | n.s. |  |  |
| C12-DC | 16 | 1.019 | 0.48 | Mixed model | n.s. |  |  |
| C3-OH | 16 | -1.026 | 0.48 | Mixed model | n.s. |  |  |
| LC_Lithocholic_acid | 11 | -1.692 | 0.49 | Mixed model | n.s. |  |  |
| PC ae C38:4 | 16 | -1.037 | 0.49 | Mixed model | n.s. |  |  |
| PC ae C30:1 | 11 | 1.224 | 0.49 | Mixed model | n.s. |  |  |
| SM C24:1 | 16 | 1.027 | 0.49 | Mixed model | n.s. |  |  |
| C16:1-OH | 16 | -1.052 | 0.50 | Mixed model | n.s. |  |  |
| PC ae C36:2 | 16 | 1.046 | 0.50 | Mixed model | n.s. |  |  |
| Lys | 16 | -1.034 | 0.51 | Mixed model | n.s. |  |  |
| C6:1 | 16 | -1.067 | 0.51 | Mixed model | n.s. |  |  |
| Cortisol | 16 | 1.111 | 0.51 | Mixed model | n.s. |  |  |
| SDMA | 7 | 1.057 | 0.51 | Mixed model | n.s. |  |  |
| ADMA | 16 | 1.035 | 0.52 | Mixed model | n.s. |  |  |
| Kynurenine | 16 | 1.030 | 0.52 | Mixed model | n.s. |  |  |
| Taurine | 16 | 1.027 | 0.52 | Mixed model | n.s. |  |  |
| PC ae C40:5 | 16 | 1.028 | 0.54 | Mixed model | n.s. |  |  |
| SM C26:0 | 16 | 1.033 | 0.54 | Mixed model | n.s. |  |  |
| SM (OH) C24:1 | 16 | 1.019 | 0.55 | Mixed model | n.s. |  |  |
| C18:1 | 16 | -1.116 | 0.56 | Wilcoxon | n.s. |  |  |
| PC ae C42:1 | 16 | -1.039 | 0.57 | Mixed model | n.s. |  |  |
| SM C24:0 | 16 | -1.014 | 0.58 | Mixed model | n.s. |  |  |
| C10:2 | 16 | 1.035 | 0.58 | Mixed model | n.s. |  |  |
| PC ae C36:5 | 16 | -1.035 | 0.60 | Mixed model | n.s. |  |  |
| PC ae C36:3 | 16 | -1.039 | 0.60 | Mixed model | n.s. |  |  |
| PC aa C32:0 | 16 | 1.019 | 0.61 | Mixed model | n.s. |  |  |
| Gly | 16 | 1.048 | 0.63 | Wilcoxon | n.s. |  |  |
| PC ae C34:2 | 16 | -1.036 | 0.64 | Mixed model | n.s. |  |  |
| Ac-Orn | 11 | -1.114 | 0.65 | Mixed model | n.s. |  |  |
| lysoPC a C16:0 | 16 | -1.026 | 0.65 | Mixed model | n.s. |  |  |
| Met-SO | 13 | -1.166 | 0.66 | Mixed model | n.s. |  |  |
| C5-OH (C3-DC-M) | 16 | 1.026 | 0.66 | Mixed model | n.s. |  |  |
| PC aa C40:1 | 16 | 1.024 | 0.67 | Mixed model | n.s. |  |  |
| Trp | 16 | 1.010 | 0.67 | Wilcoxon | n.s. |  |  |
| His | 16 | -1.018 | 0.67 | Mixed model | n.s. |  |  |
| Corticosterone | 8 | 1.234 | 0.67 | Mixed model | n.s. |  |  |
| PC ae C44:4 | 16 | -1.016 | 0.68 | Mixed model | n.s. |  |  |
| Triglycerides [mg/dl] | 16 | 1.032 | 0.68 | Mixed model | n.s. |  |  |
| C4 | 16 | 1.023 | 0.68 | Mixed model | n.s. |  |  |
| PC ae C40:4 | 16 | -1.011 | 0.69 | Mixed model | n.s. |  |  |
| Putrescine | 16 | -1.036 | 0.69 | Mixed model | n.s. |  |  |
| TDC_Taurodeoxycholic_acid | 11 | -1.235 | 0.70 | Mixed model | n.s. |  |  |
| Phe | 16 | 1.014 | 0.70 | Mixed model | n.s. |  |  |
| 17OH-Progesterone | 16 | -1.083 | 0.71 | Mixed model | n.s. |  |  |
| C14:2-OH | 16 | 1.005 | 0.71 | Wilcoxon | n.s. |  |  |
| Progesterone | 10 | 1.072 | 0.71 | Mixed model | n.s. |  |  |
| C0 | 16 | 1.009 | 0.73 | Mixed model | n.s. |  |  |
| C5:1-DC | 16 | 1.030 | 0.73 | Mixed model | n.s. |  |  |
| PC ae C44:3 | 16 | -1.029 | 0.74 | Mixed model | n.s. |  |  |
| Asp | 16 | 1.020 | 0.74 | Wilcoxon | n.s. |  |  |
| lysoPC a C24:0 | 16 | -1.018 | 0.75 | Mixed model | n.s. |  |  |
| C4:1 | 16 | -1.017 | 0.76 | Mixed model | n.s. |  |  |
| C18 | 16 | -1.015 | 0.76 | Mixed model | n.s. |  |  |
| PC ae C34:3 | 16 | 1.027 | 0.77 | Mixed model | n.s. |  |  |
| Asn | 16 | -1.012 | 0.77 | Mixed model | n.s. |  |  |
| Gln | 16 | 1.013 | 0.77 | Mixed model | n.s. |  |  |
| TLC_Taurolithocholic_acid | 9 | 1.124 | 0.79 | Mixed model | n.s. |  |  |
| C5-M-DC | 16 | 1.007 | 0.80 | Mixed model | n.s. |  |  |
| Glucose [mg/dl] | 16 | 1.017 | 0.80 | Mixed model | n.s. |  |  |
| lysoPC a C18:0 | 16 | 1.020 | 0.82 | Mixed model | n.s. |  |  |
| Tyr | 16 | 1.033 | 0.82 | Wilcoxon | n.s. |  |  |
| lysoPC a C17:0 | 16 | 1.010 | 0.82 | Wilcoxon | n.s. |  |  |
| lysoPC a C28:1 | 16 | 1.047 | 0.82 | Wilcoxon | n.s. |  |  |
| C8 | 16 | -1.013 | 0.83 | Mixed model | n.s. |  |  |
| lysoPC a C26:1 | 16 | -1.005 | 0.84 | Mixed model | n.s. |  |  |
| Cortisone | 16 | 1.019 | 0.84 | Mixed model | n.s. |  |  |
| C14:1-OH | 16 | -1.017 | 0.85 | Mixed model | n.s. |  |  |
| SM (OH) C22:1 | 16 | 1.005 | 0.85 | Mixed model | n.s. |  |  |
| GC_Glycocholic_acid | 16 | 1.153 | 0.86 | Wilcoxon | n.s. |  |  |
| TCDC_Taurochenodeoxycholic_acid | 16 | -1.070 | 0.87 | Mixed model | n.s. |  |  |
| PC ae C38:6 | 16 | 1.011 | 0.88 | Mixed model | n.s. |  |  |
| lysoPC a C26:0 | 16 | -1.015 | 0.88 | Mixed model | n.s. |  |  |
| Insulin [U/L] | 16 | 1.023 | 0.89 | Mixed model | n.s. |  |  |
| PC aa C38:4 | 16 | -1.006 | 0.90 | Mixed model | n.s. |  |  |
| Leu | 16 | 1.018 | 0.90 | Wilcoxon | n.s. |  |  |
| C10:1 | 16 | -1.011 | 0.90 | Mixed model | n.s. |  |  |
| C16-OH | 16 | 1.008 | 0.92 | Mixed model | n.s. |  |  |
| C12 | 16 | -1.010 | 0.93 | Mixed model | n.s. |  |  |
| C12:1 | 16 | 1.008 | 0.95 | Mixed model | n.s. |  |  |
| PC ae C42:0 | 16 | 1.002 | 0.96 | Mixed model | n.s. |  |  |
| C10 | 16 | 1.004 | 0.97 | Mixed model | n.s. |  |  |
| H1 | 16 | -1.002 | 0.97 | Mixed model | n.s. |  |  |
| total DMA | 16 | 1.001 | 0.97 | Mixed model | n.s. |  |  |
