# Supplementary material for: Metabolomics of Ramadan fasting: an opportunity for the controlled study of physiological responses to food intake
Source: J Transl Med. 2014 Jun 6;12:161. doi: 10.1186/1479-5876-12-161 (PMC4063233; doi:10.1186/1479-5876-12-161)
Supplement: Additional file 4: Figure S1 — Clinical biochemistry and metabolomics data. Time points of sampling are presented on the x-axis and are defined as follows: 1 + 2: collected during the first week of Ramadan, 3 + 4: collected during the last week of Ramadan, 5: collected several weeks after Ramadan; Fasting state: 1 + 3: Ramadan fasting, 2 + 4: after fast breaking with identical meal, 5: overnight fasting. Left: data presented as boxplots; right: data presented as scatterplots, colored by participant, point before/after fast breaking are connected for each individual, red lines: increase in metabolite concentrations, blue line: decrease during fast breaking. [file 1479-5876-12-161-S4.pdf]

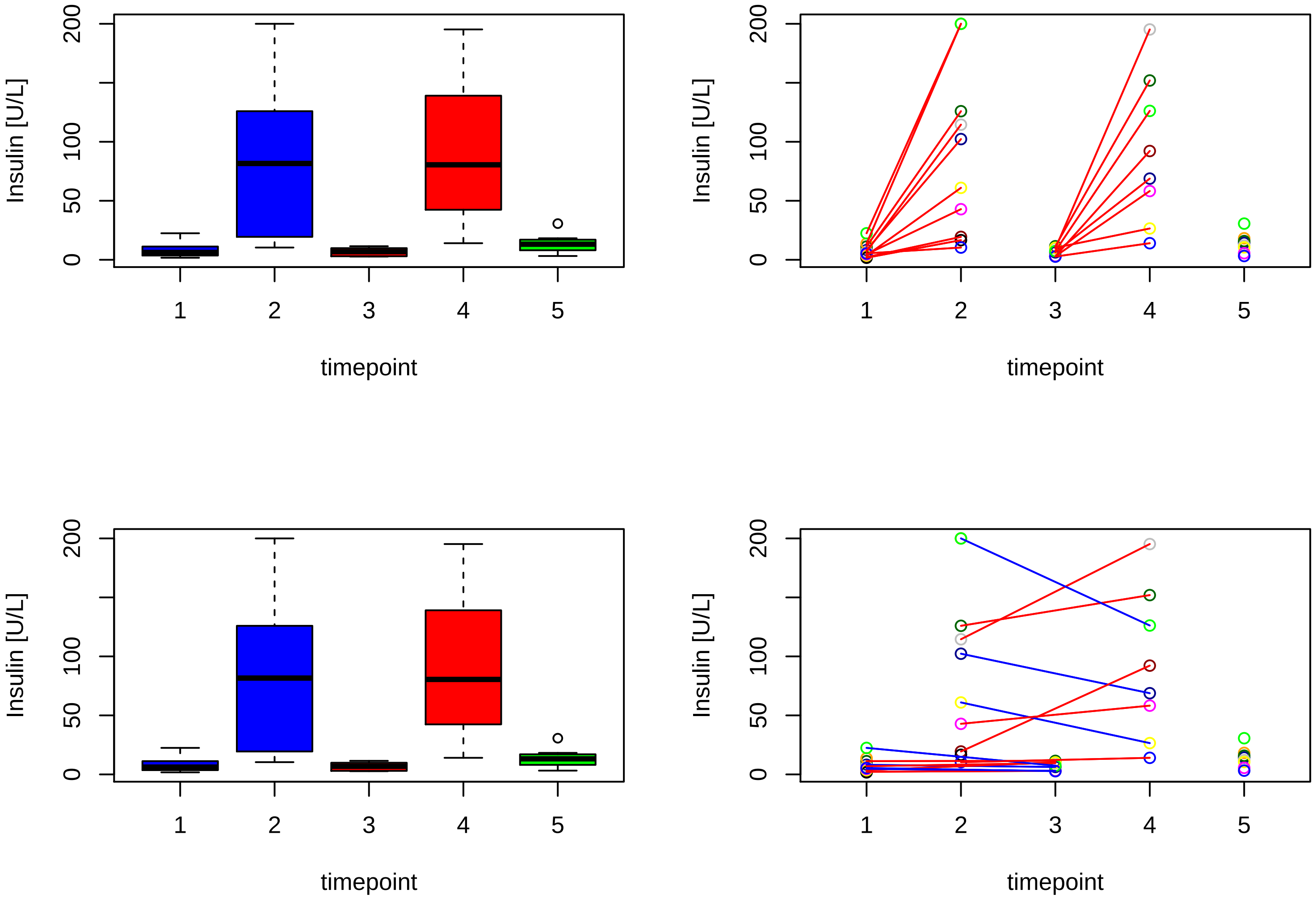
Insulin [U/L]
0
50
100
200
1
2
3
4
5
timepoint
Insulin [U/L]
0
50
100
200
1
2
3
4
5
timepoint
Insulin [U/L]
0
50
100
200
1
2
3
4
5
timepoint
Insulin [U/L]
0
50
100
200
1
2
3
4
5
timepoint

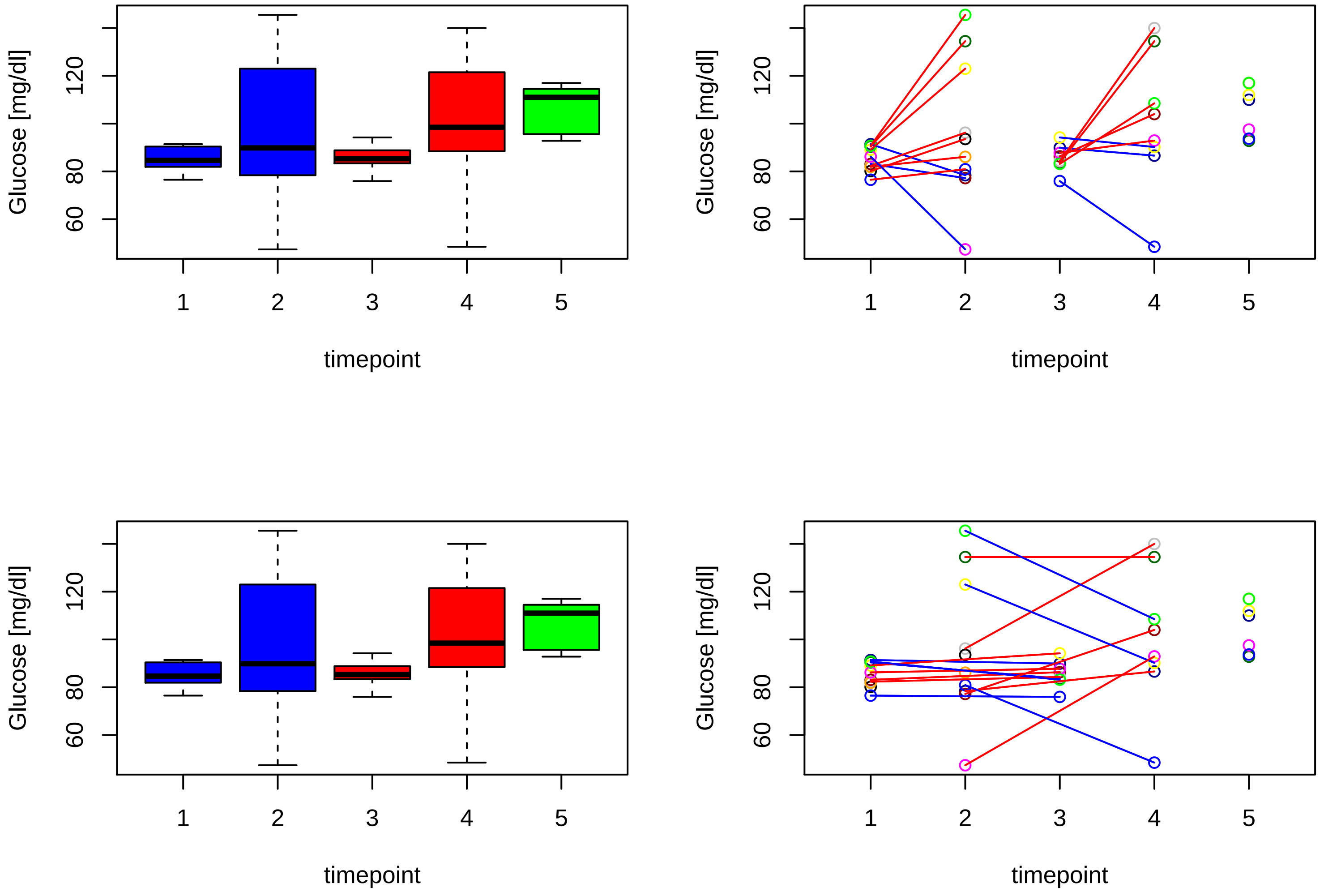

Glucose [mg/dl]
60
80
120
1
2
3
4
5
timepoint

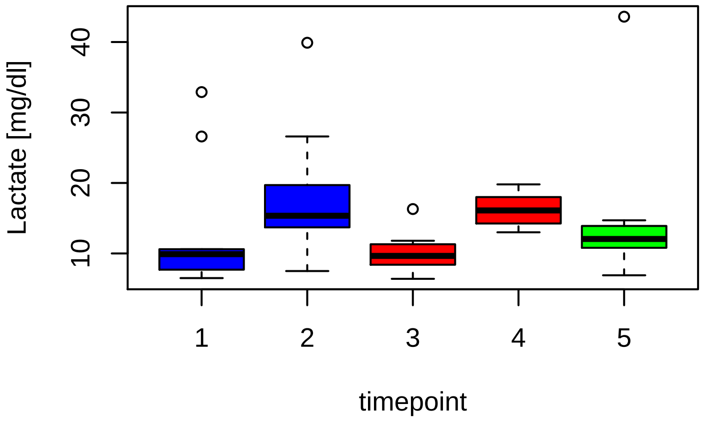
Lactate [mg/dl]
10
20
30
40
1
2
3
4
5
timepoint

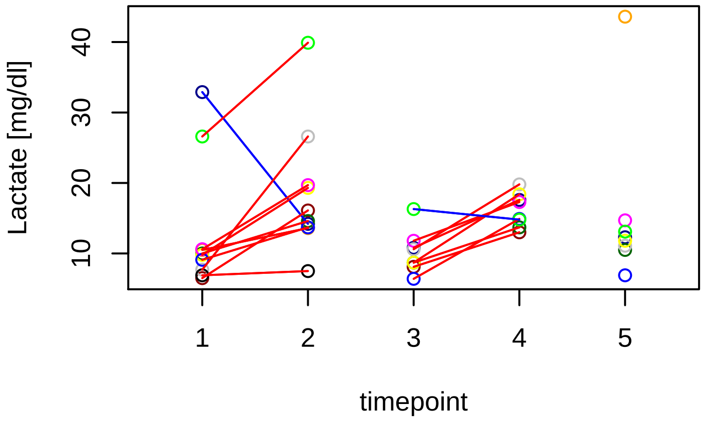
Lactate [mg/dl]
10
20
30
40
1
2
3
4
5
timepoint

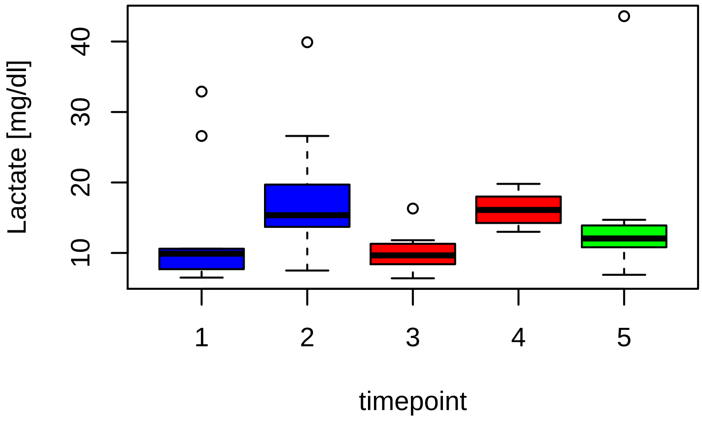
Lactate [mg/dl]
10
20
30
40
1
2
3
4
5
timepoint

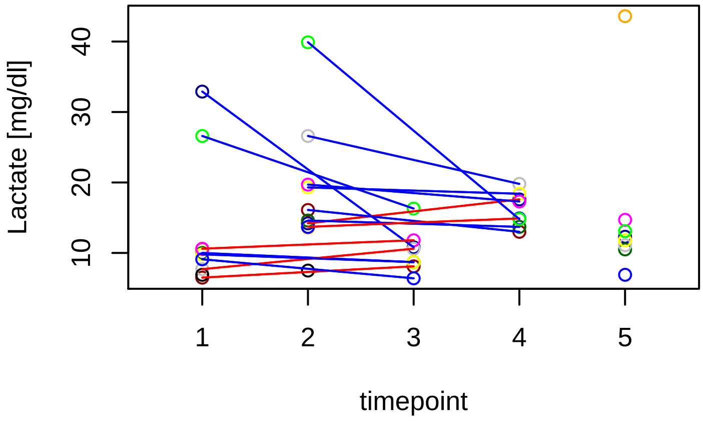
Lactate [mg/dl]
10
20
30
40
1
2
3
4
5
timepoint

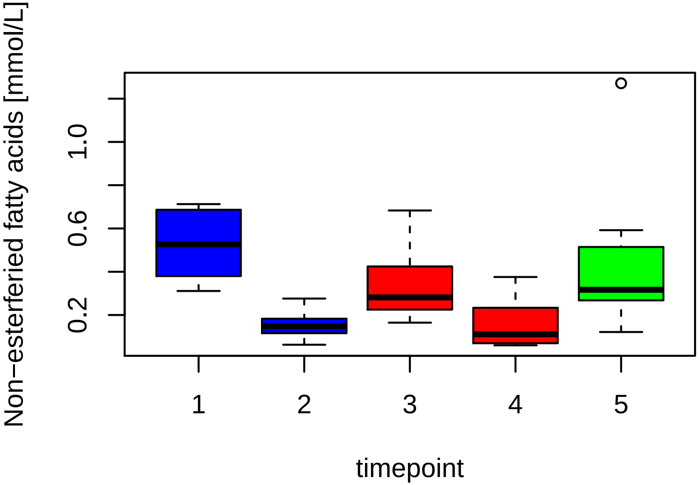
Non−esterfied fatty acids [mmol/L]
0.2
0.6
1.0
1
2
3
4
5
timepoint

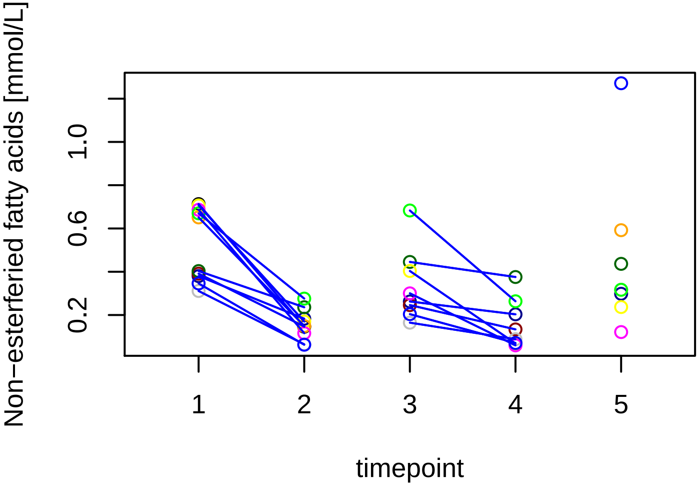
Non−esterfied fatty acids [mmol/L]
0.2
0.6
1.0
1
2
3
4
5
timepoint

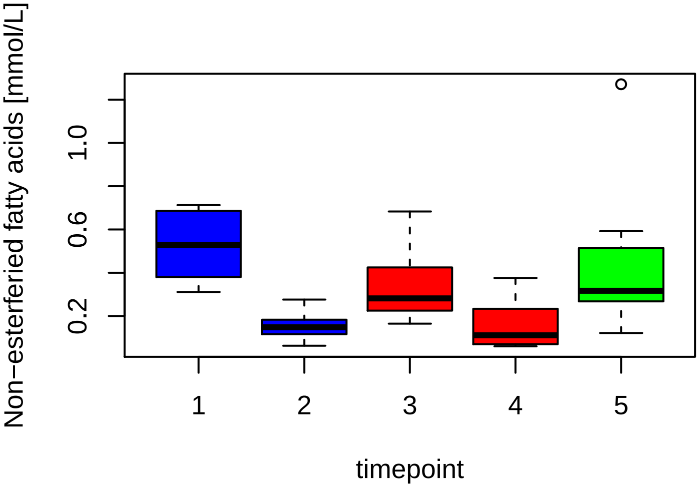
Non−esterfied fatty acids [mmol/L]
0.2
0.6
1.0
1
2
3
4
5
timepoint

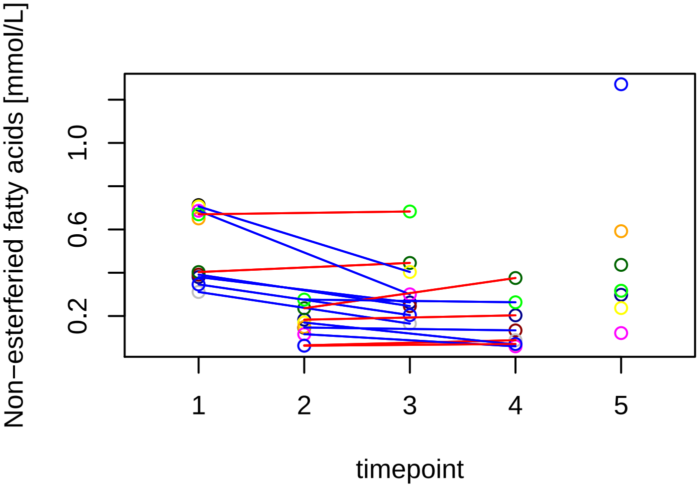
Non−esterfied fatty acids [mmol/L]
0.2
0.6
1.0
1
2
3
4
5
timepoint

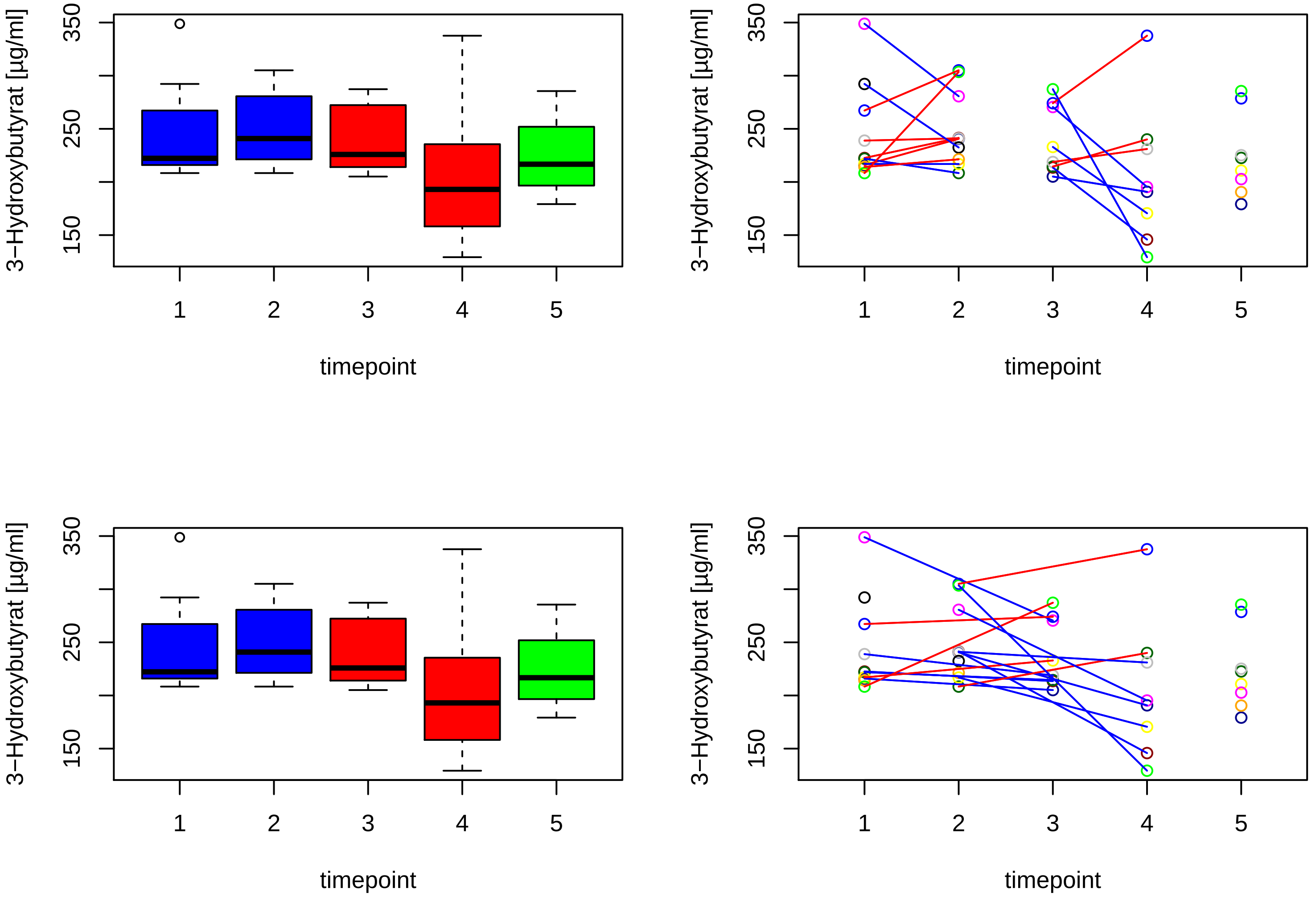
3−Hydroxybutyrat [µg/ml]
150
250
350
1
2
3
4
5
timepoint
3−Hydroxybutyrat [µg/ml]
150
250
350
1
2
3
4
5
timepoint
3−Hydroxybutyrat [µg/ml]
150
250
350
1
2
3
4
5
timepoint
3−Hydroxybutyrat [µg/ml]
150
250
350
1
2
3
4
5
timepoint

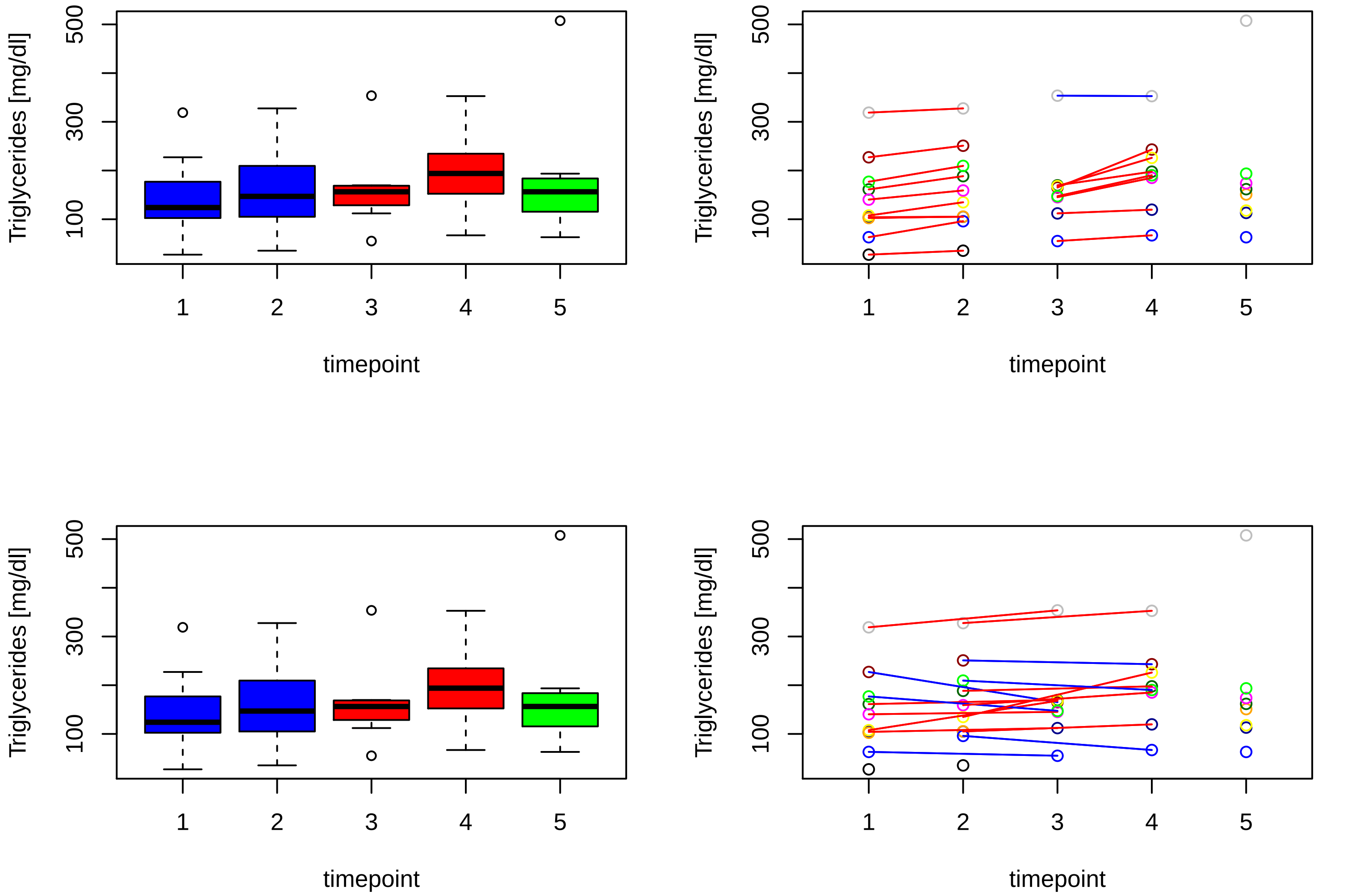
Triglycerides [mg/dl]
100
300
500
1
2
3
4
5
timepoint

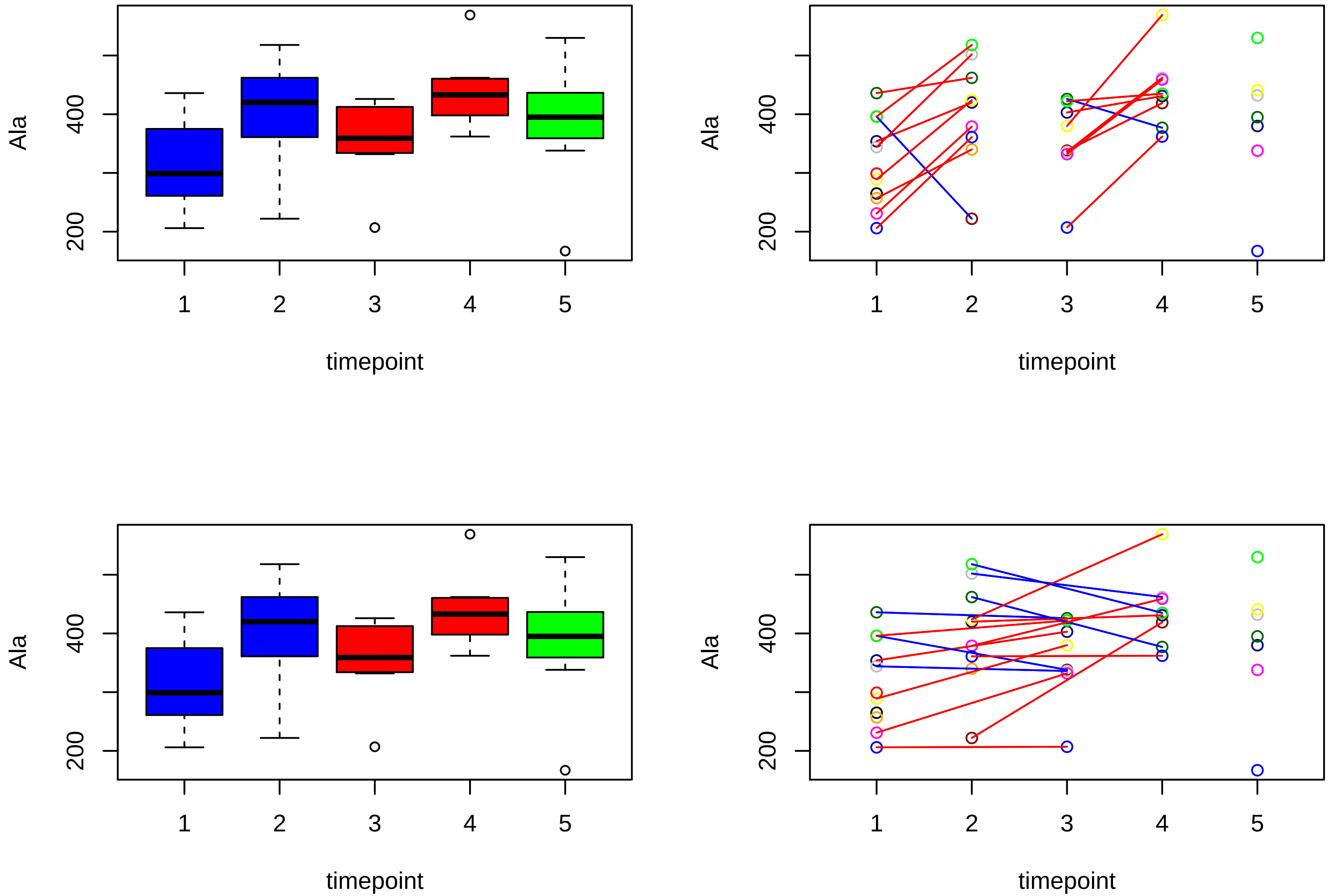
Ala
200
400
1
2
3
4
5
timepoint

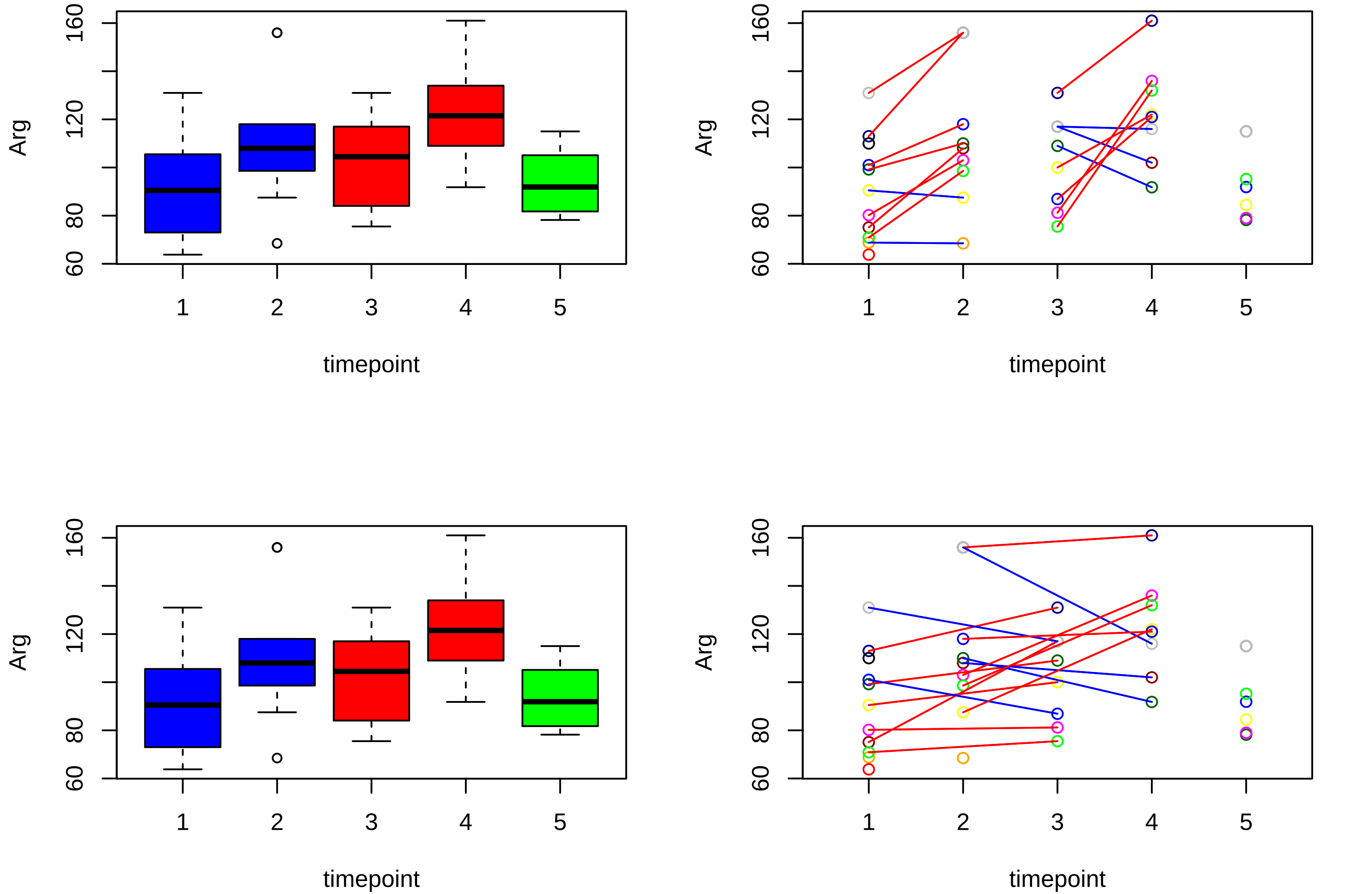

Arg
160
120
80
60
1
2
3
4
5
timepoint

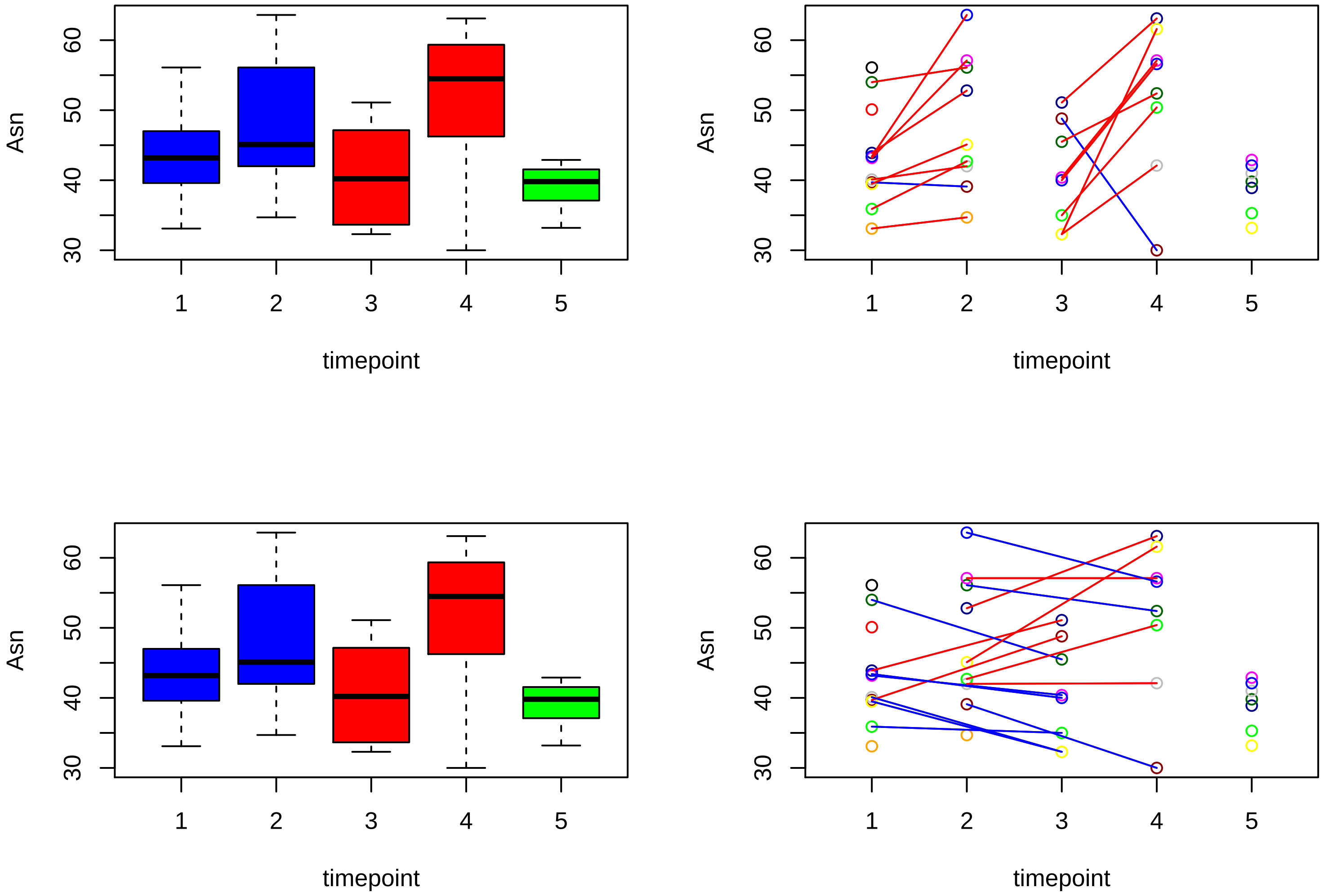
Asn
30
40
50
60
1
2
3
4
5
timepoint
Asn
30
40
50
60
1
2
3
4
5
timepoint
Asn
30
40
50
60
1
2
3
4
5
timepoint
Asn
30
40
50
60
1
2
3
4
5
timepoint

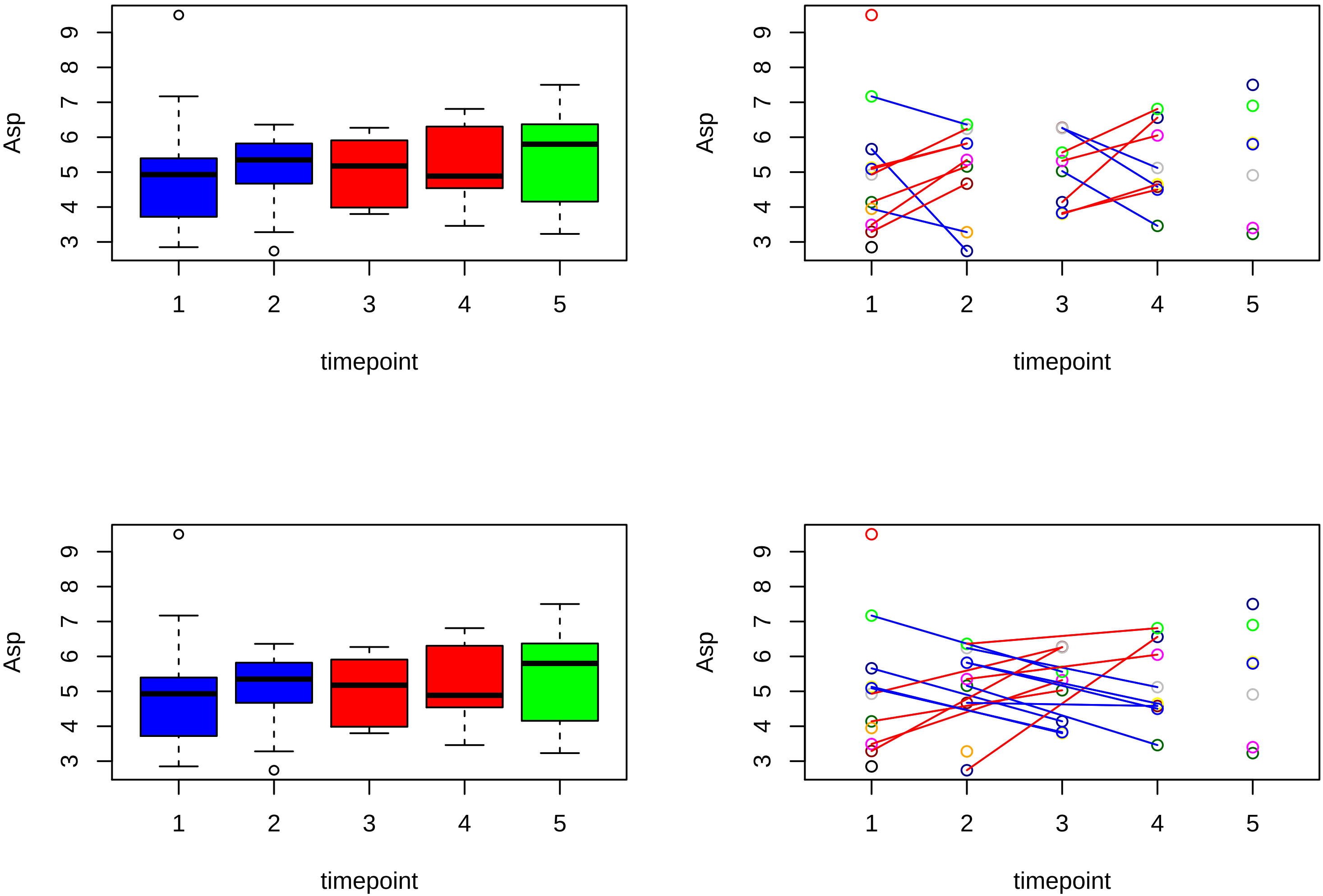
Asp
timepoint
Asp
timepoint
Asp
timepoint
Asp
timepoint

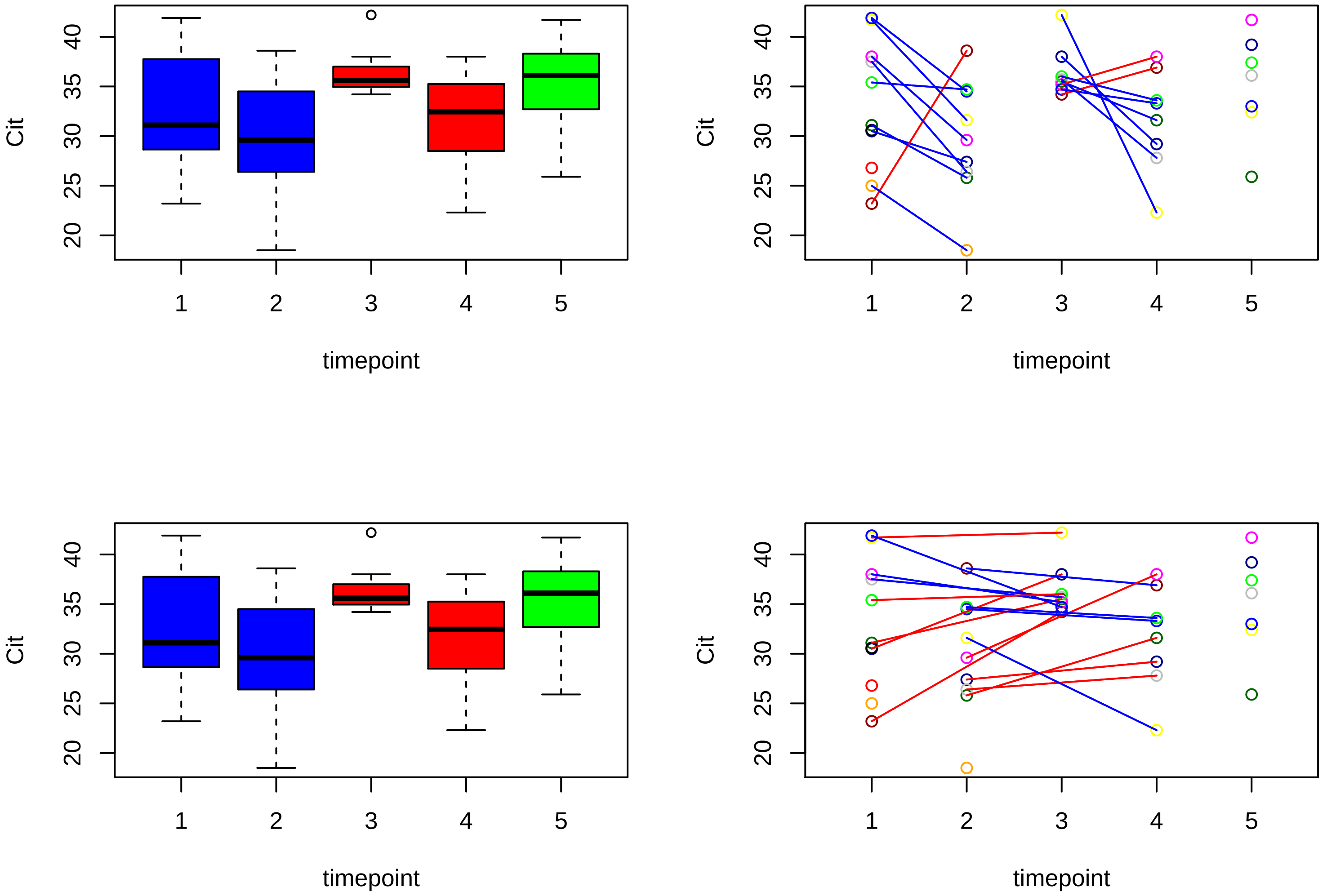

Cit
20
25
30
35
40
1
2
3
4
5
timepoint
Cit
20
25
30
35
40
1
2
3
4
5
timepoint
Cit
20
25
30
35
40
1
2
3
4
5
timepoint
Cit
20
25
30
35
40
1
2
3
4
5
timepoint

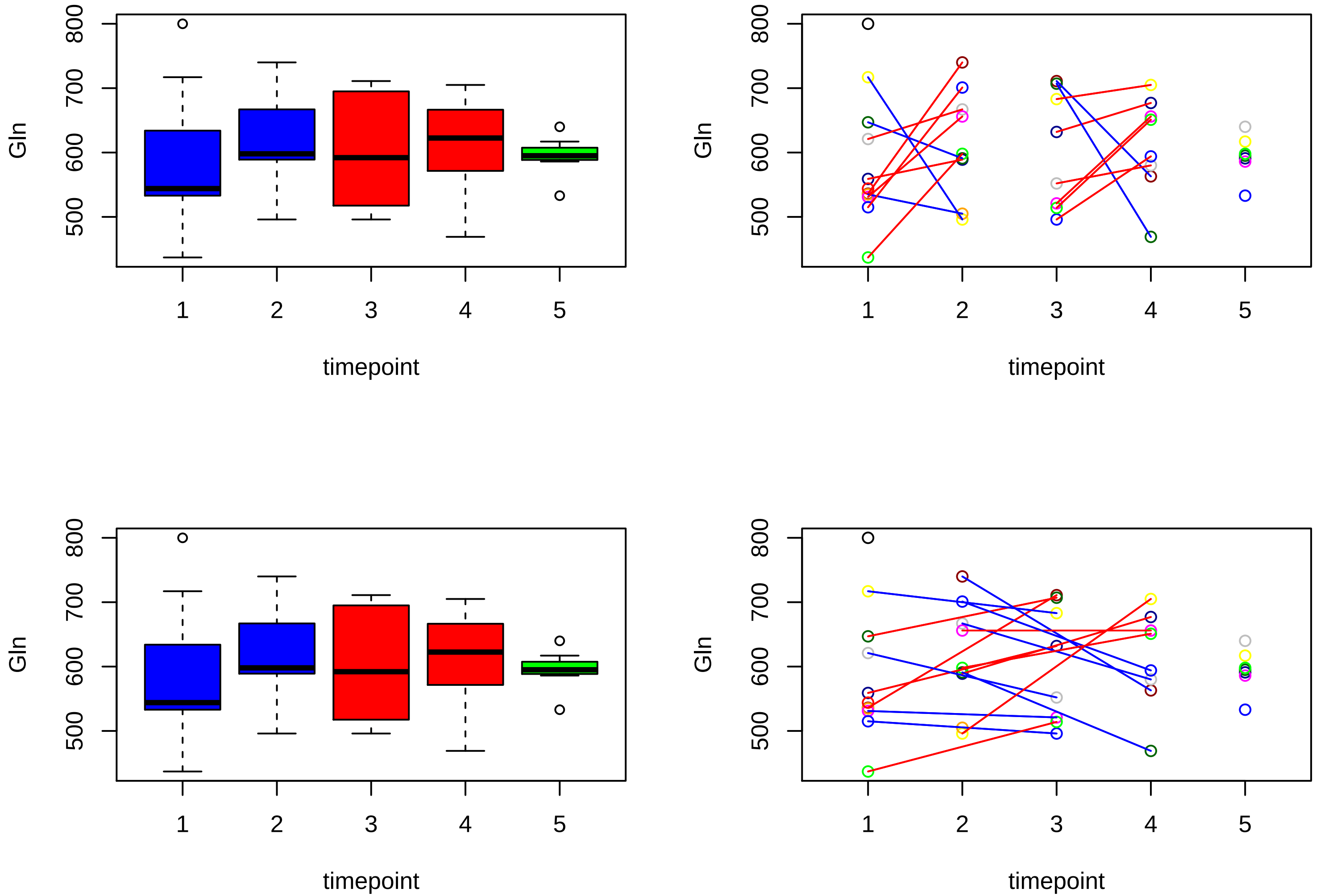
Gln
500
600
700
800
1
2
3
4
5
timepoint
Gln
500
600
700
800
1
2
3
4
5
timepoint
Gln
500
600
700
800
1
2
3
4
5
timepoint
Gln
500
600
700
800
1
2
3
4
5
timepoint

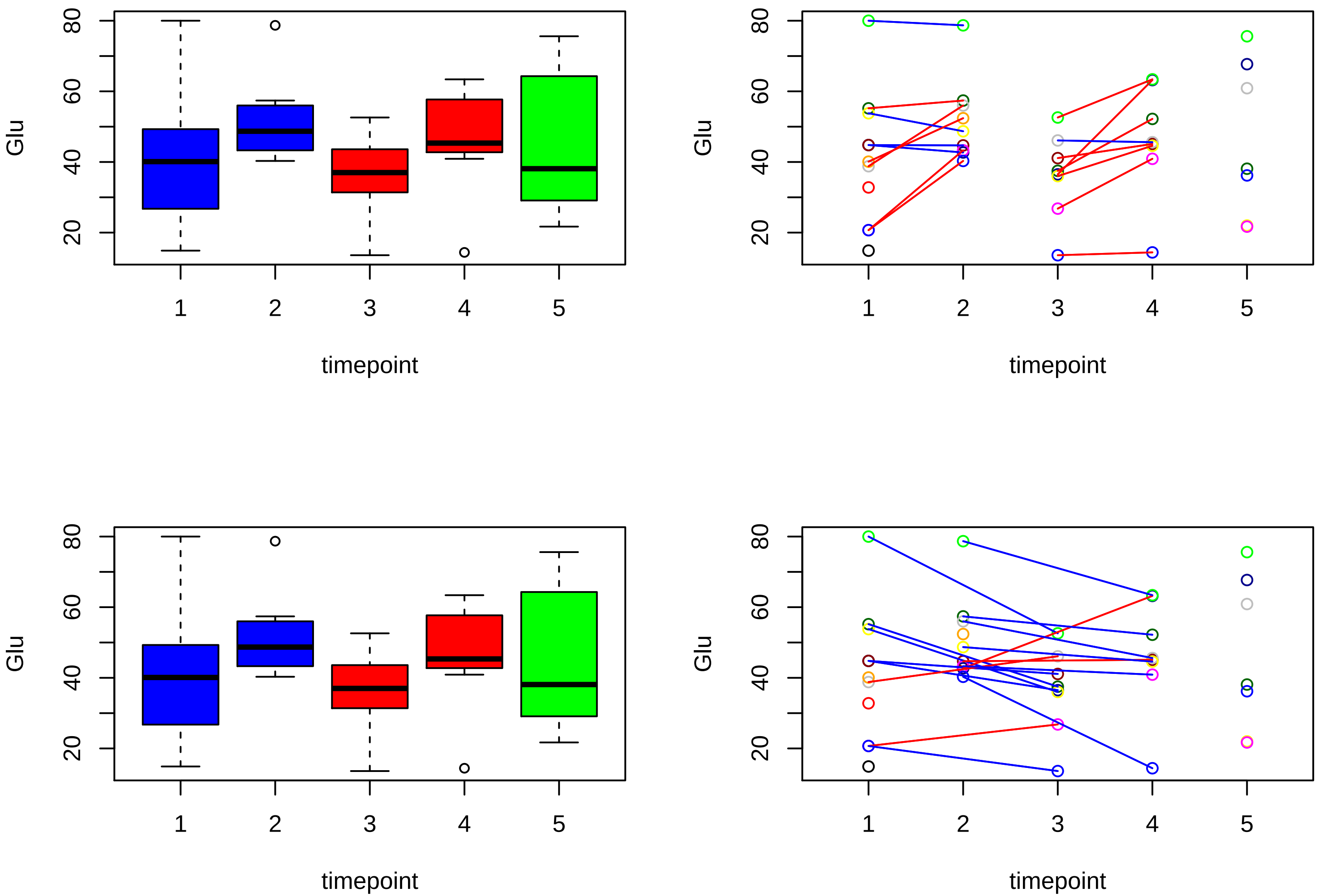
Glu
timepoint
20
40
60
80
1
2
3
4
5

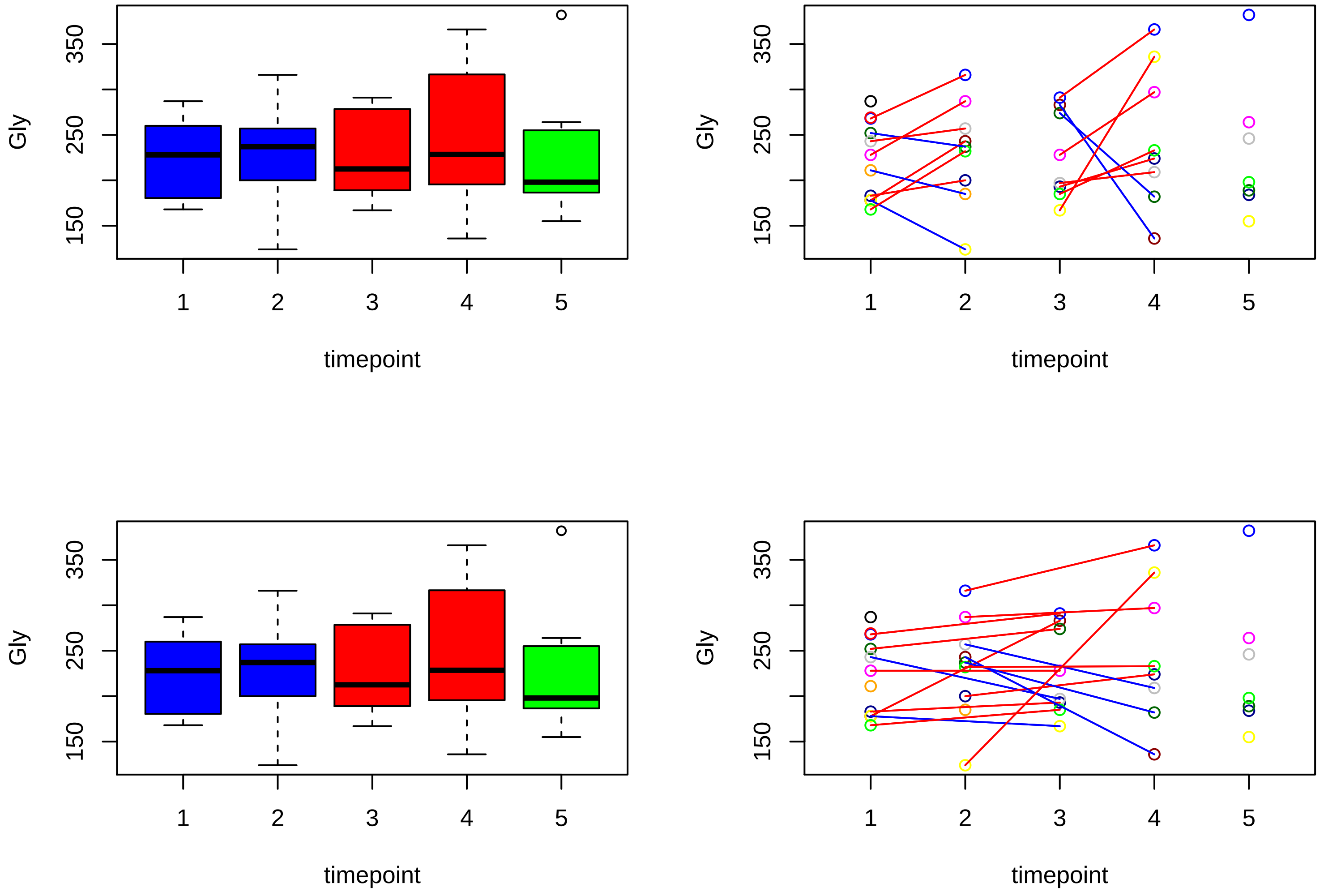
Gly
150
250
350
1
2
3
4
5
timepoint

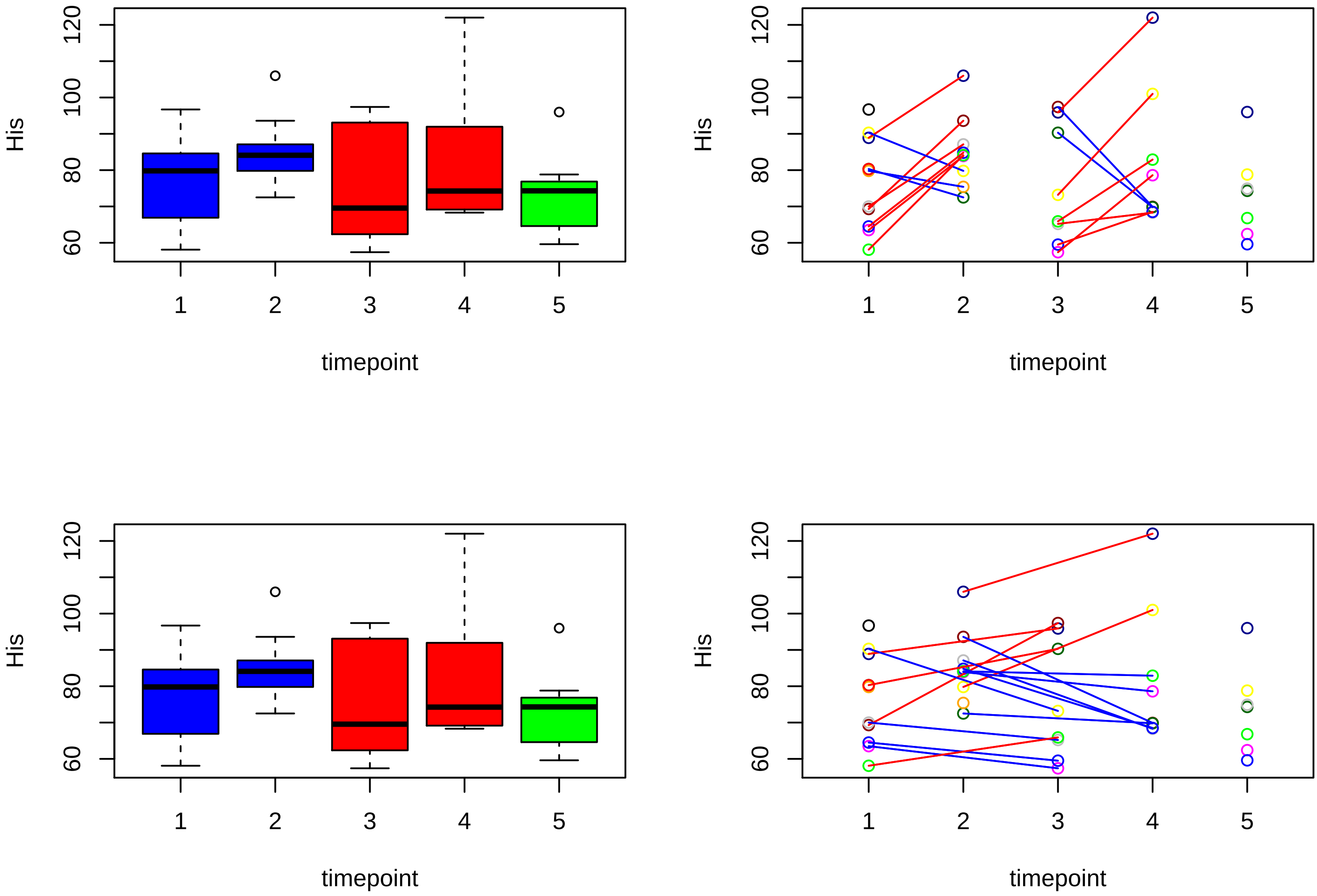
His
timepoint
60
80
100
120
1
2
3
4
5

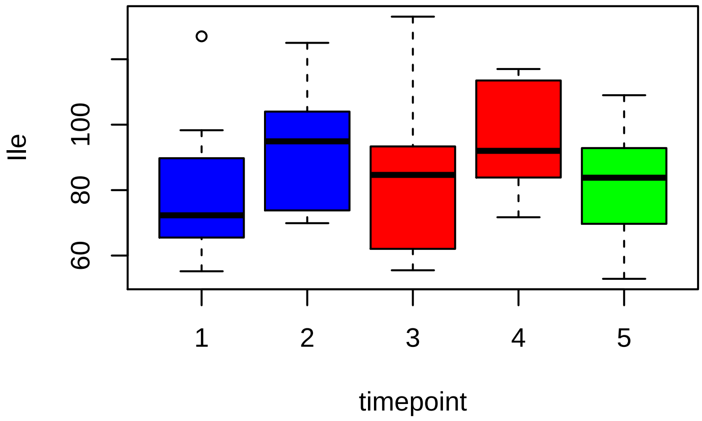

lle
60
80
100
1
2
3
4
5
timepoint

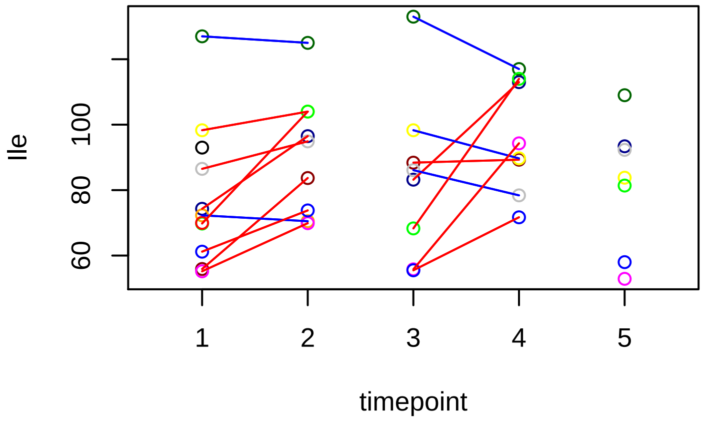

lle
60
80
100
1
2
3
4
5
timepoint

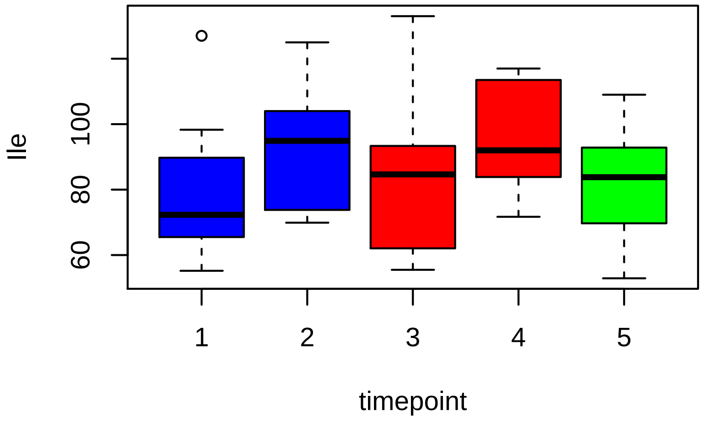

lle
60
80
100
1
2
3
4
5
timepoint

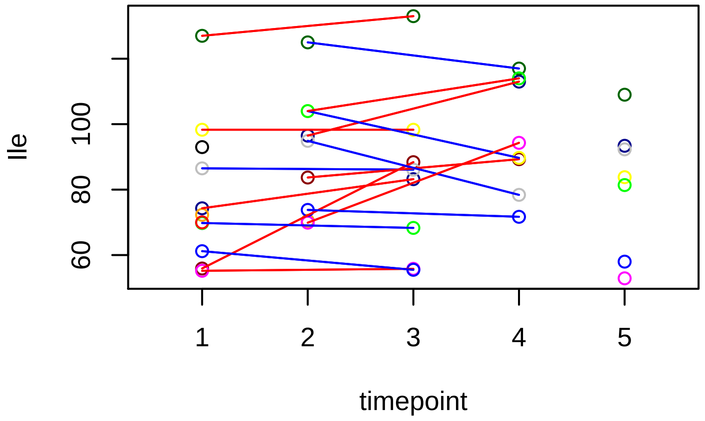

lle
60
80
100
1
2
3
4
5
timepoint

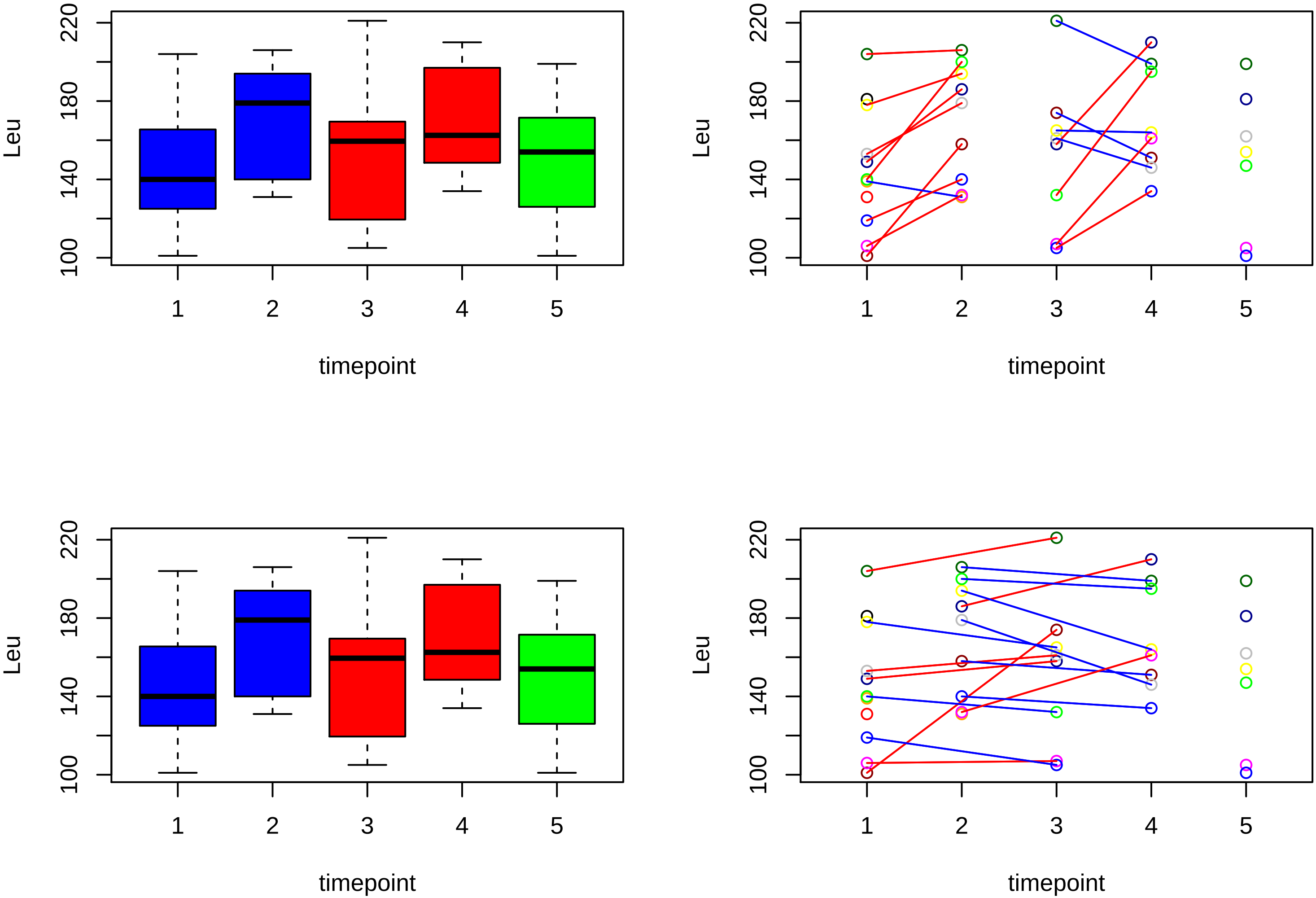

Leu
220
180
140
100
1
2
3
4
5
timepoint

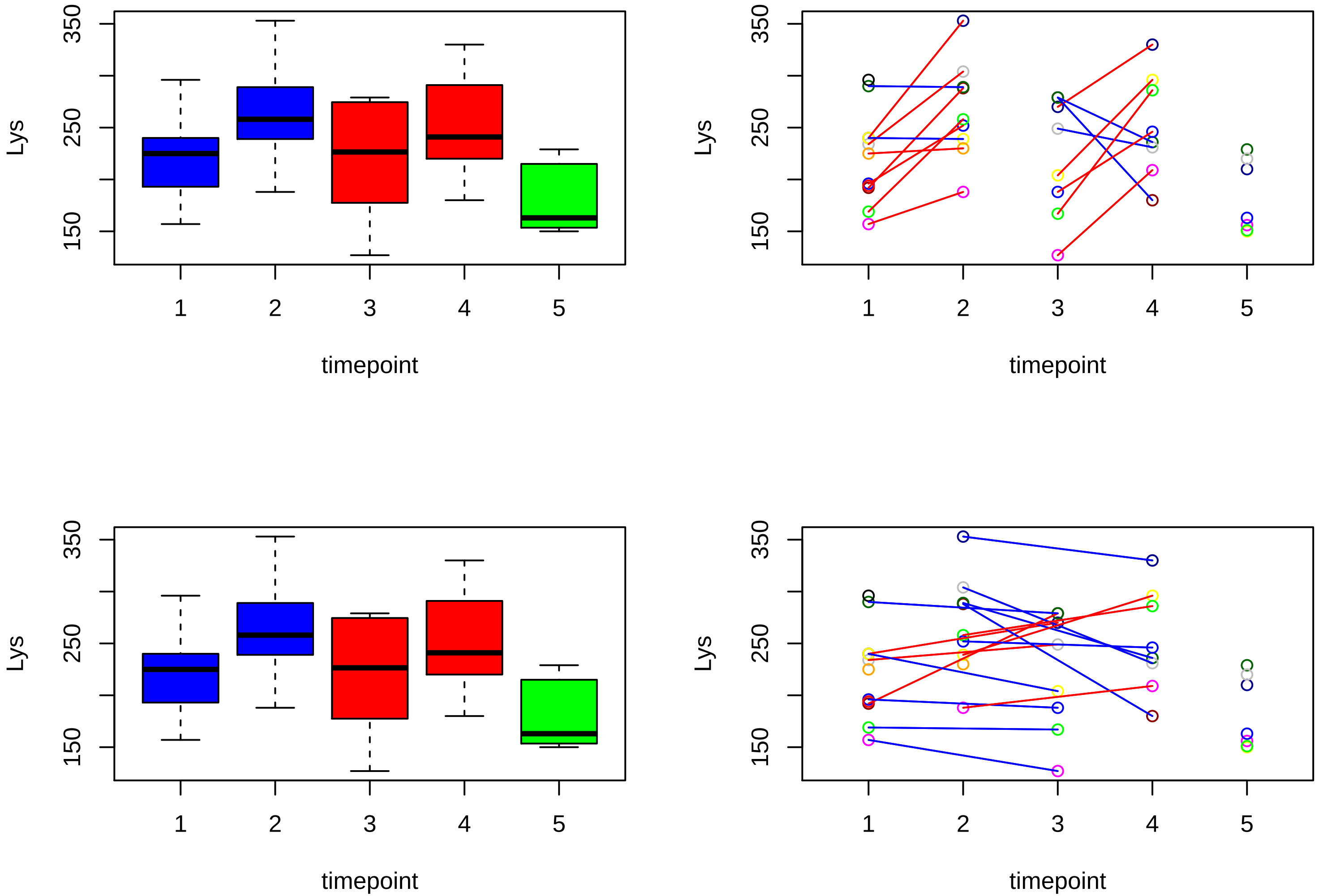

Lys
350
250
150
1
2
3
4
5
timepoint
Lys
350
250
150
1
2
3
4
5
timepoint
Lys
350
250
150
1
2
3
4
5
timepoint
Lys
350
250
150
1
2
3
4
5
timepoint

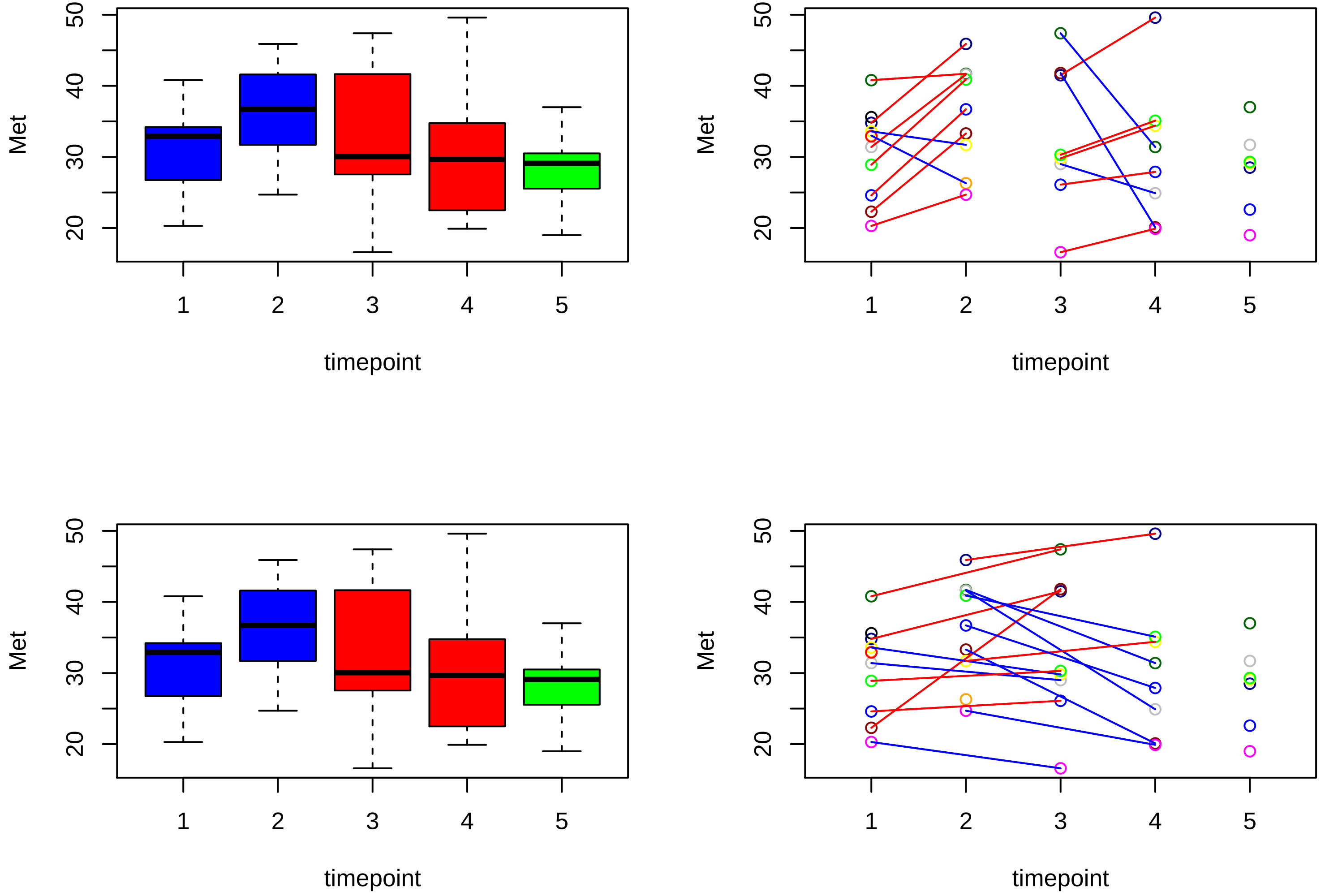
Met
50
40
30
20
1
2
3
4
5
timepoint
Met
50
40
30
20
1
2
3
4
5
timepoint
Met
50
40
30
20
1
2
3
4
5
timepoint
Met
50
40
30
20
1
2
3
4
5
timepoint

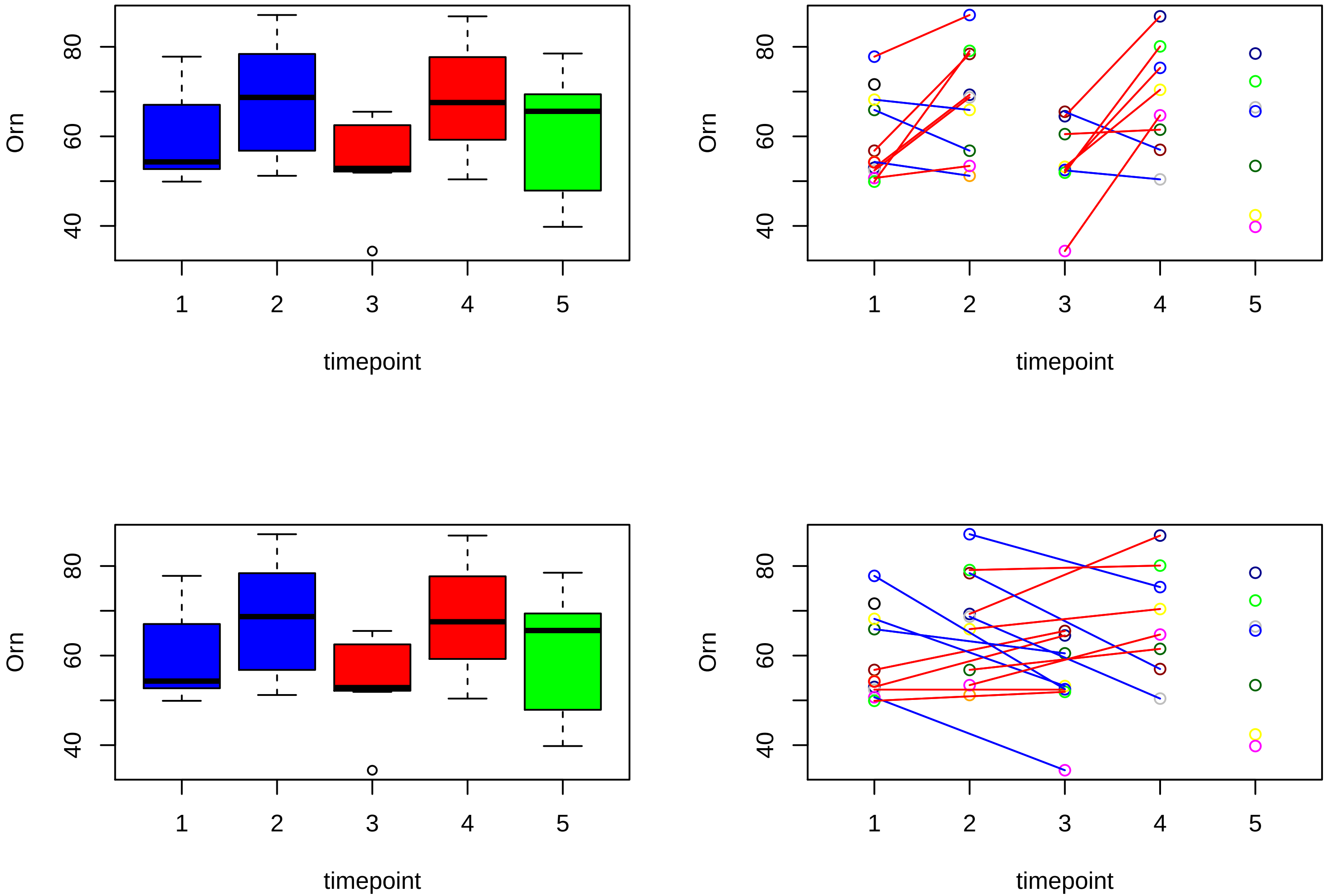

Orn
80
60
40
1
2
3
4
5
timepoint
Orn
80
60
40
1
2
3
4
5
timepoint
Orn
80
60
40
1
2
3
4
5
timepoint
Orn
80
60
40
1
2
3
4
5
timepoint

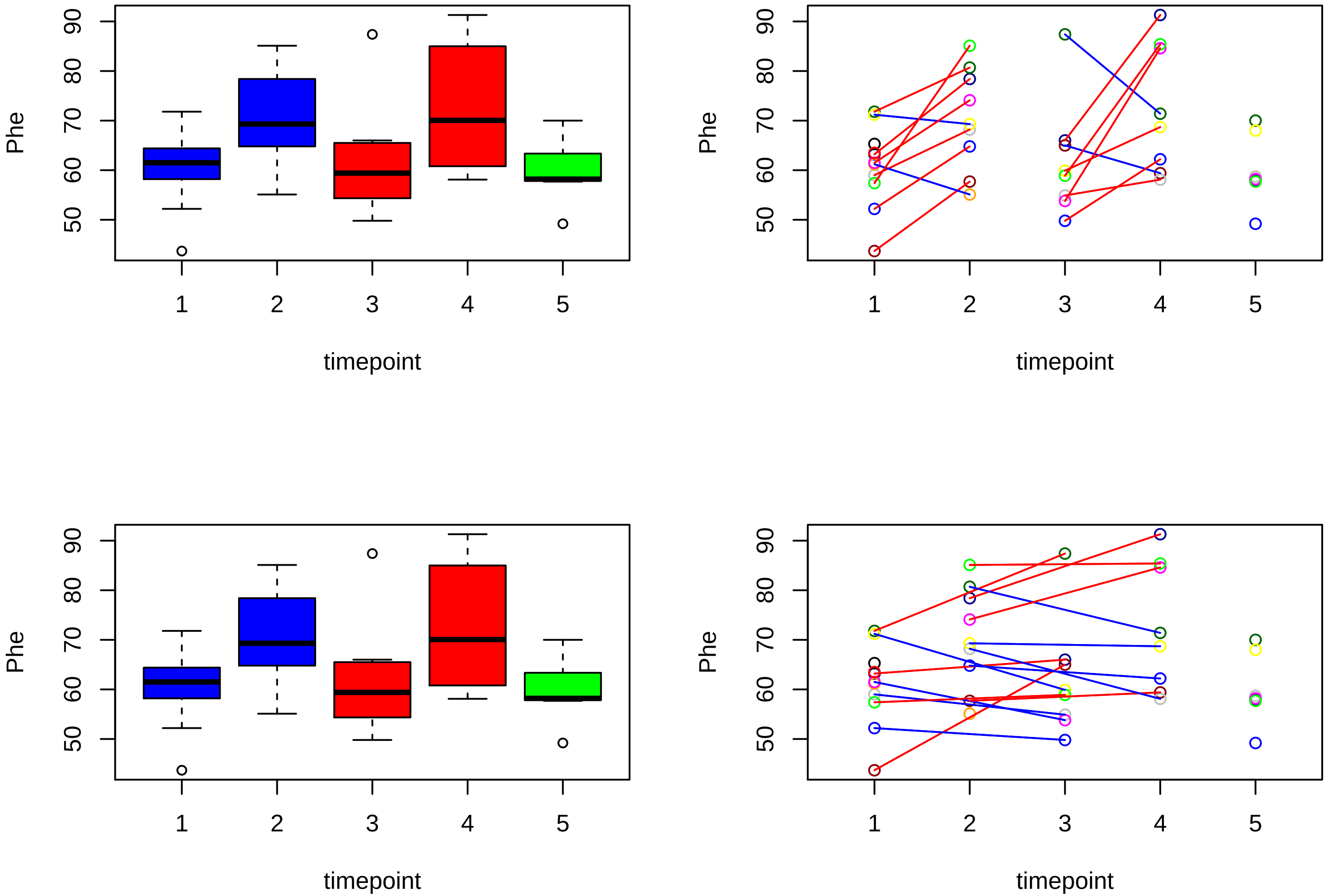
Phe
timepoint
50
60
70
80
90
1
2
3
4
5

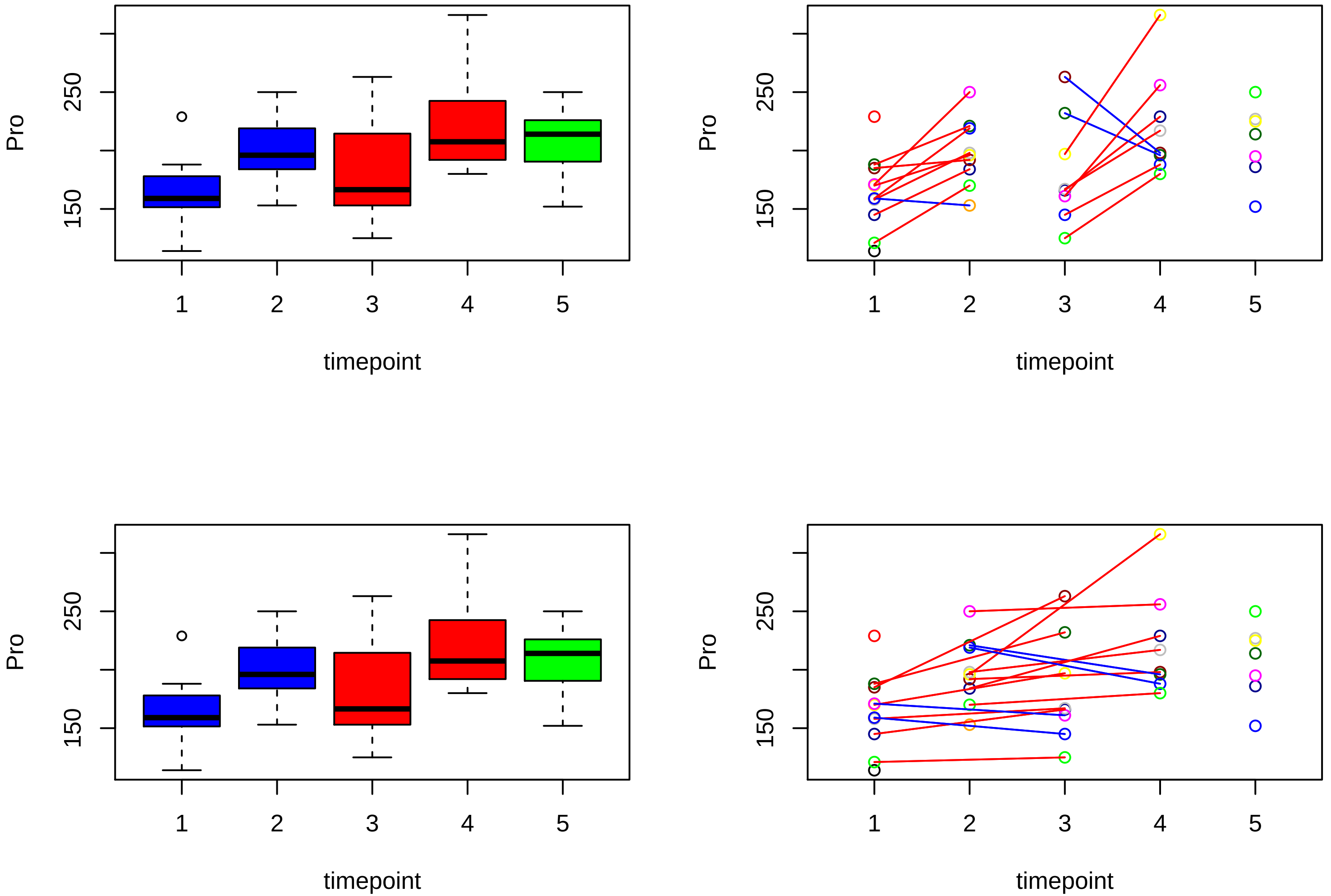
Pro
150
250
1
2
3
4
5
timepoint
Pro
150
250
1
2
3
4
5
timepoint
Pro
150
250
1
2
3
4
5
timepoint
Pro
150
250
1
2
3
4
5
timepoint

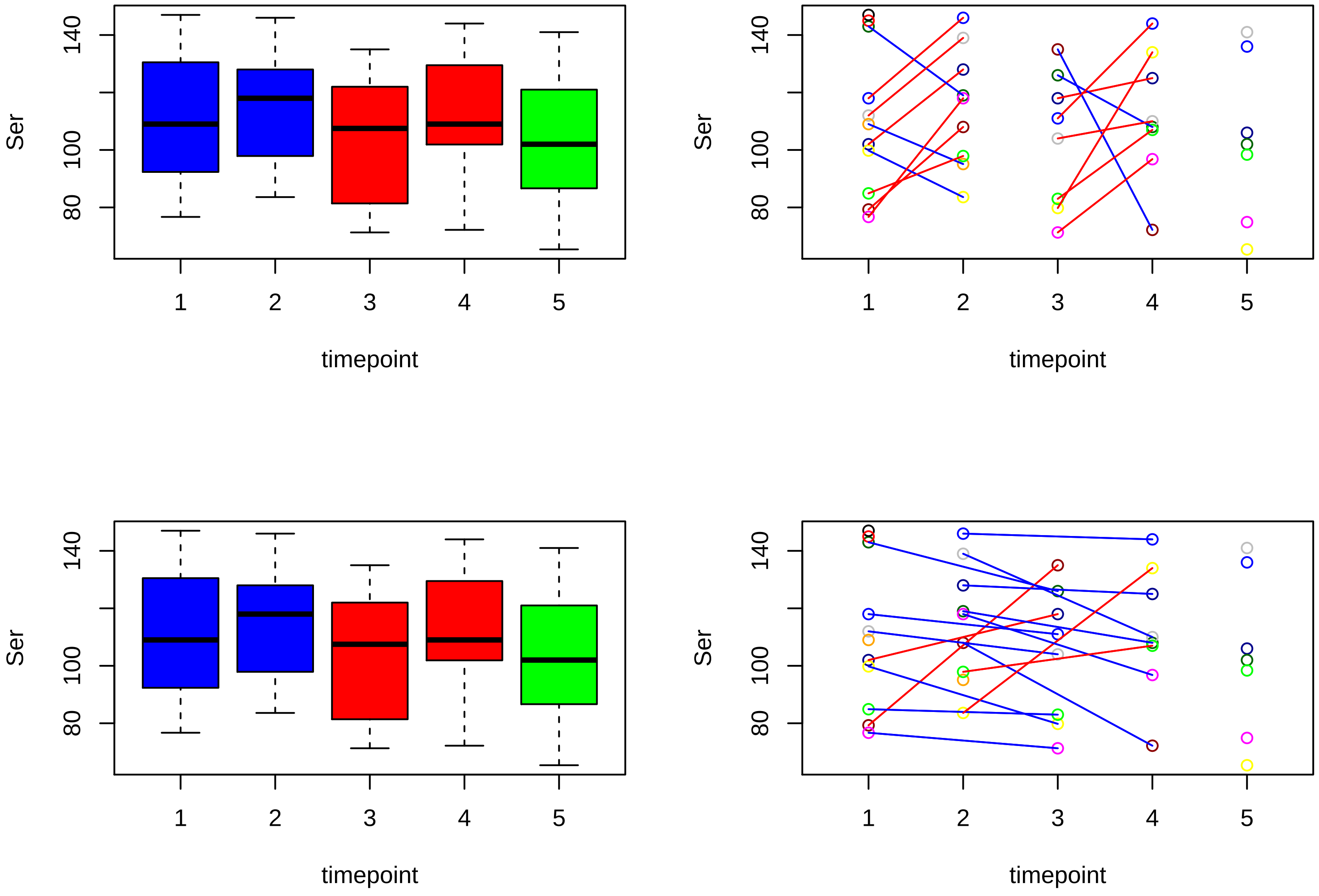
Ser
140
100
80
1
2
3
4
5
timepoint

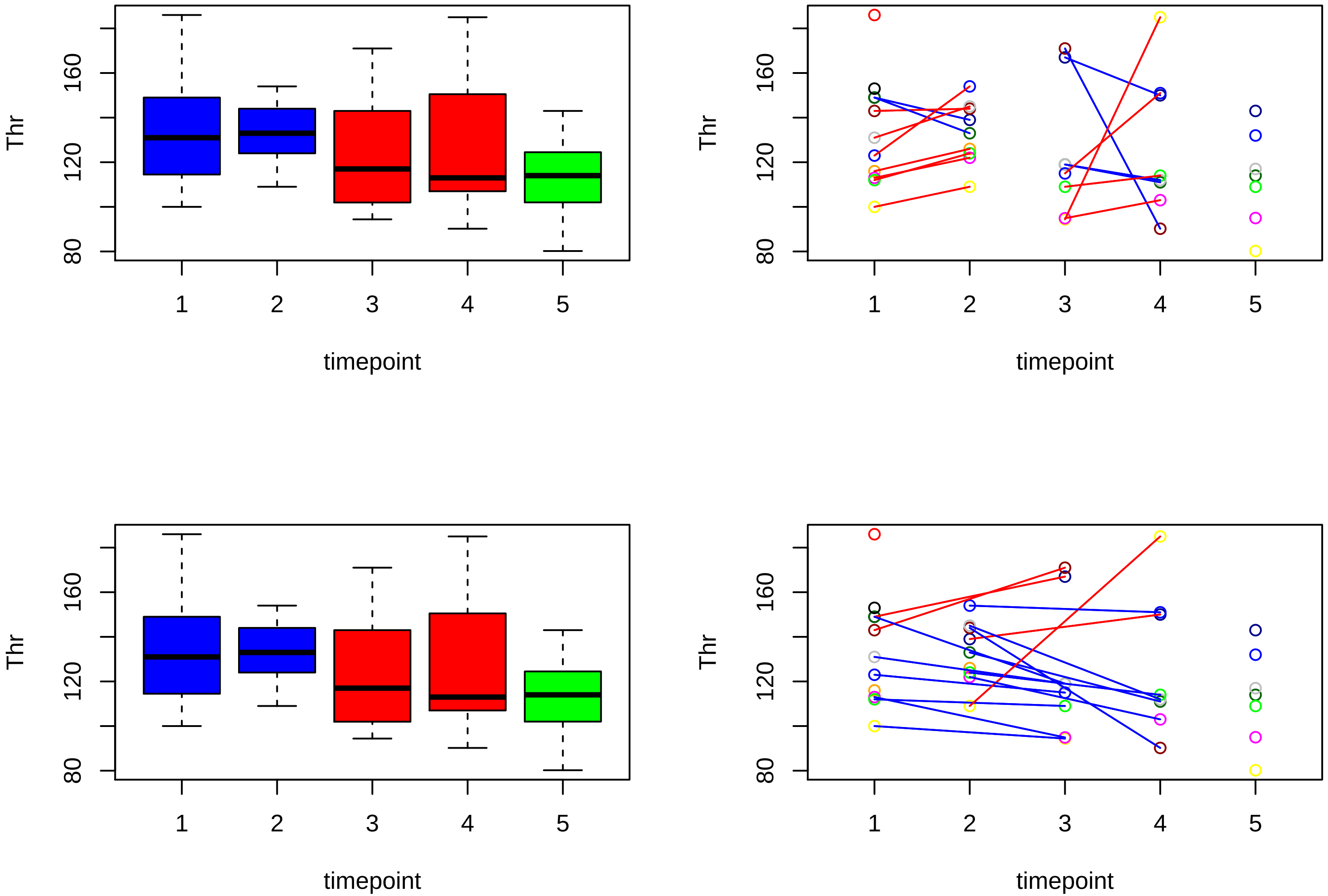
Thr
80
120
160
1
2
3
4
5
timepoint
Thr
80
120
160
1
2
3
4
5
timepoint
Thr
80
120
160
1
2
3
4
5
timepoint
Thr
80
120
160
1
2
3
4
5
timepoint

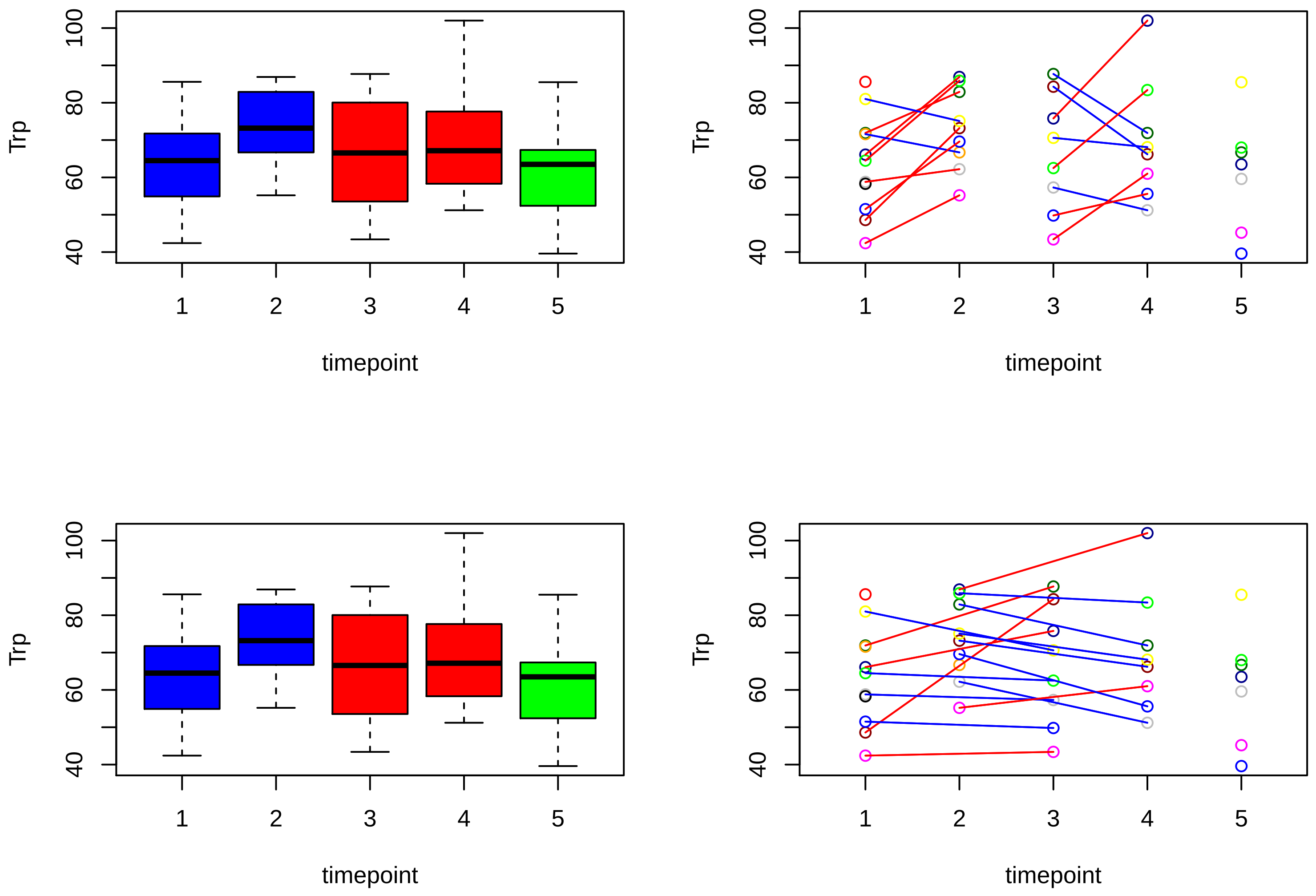

Trp
timepoint
1
2
3
4
5
40
60
80
100

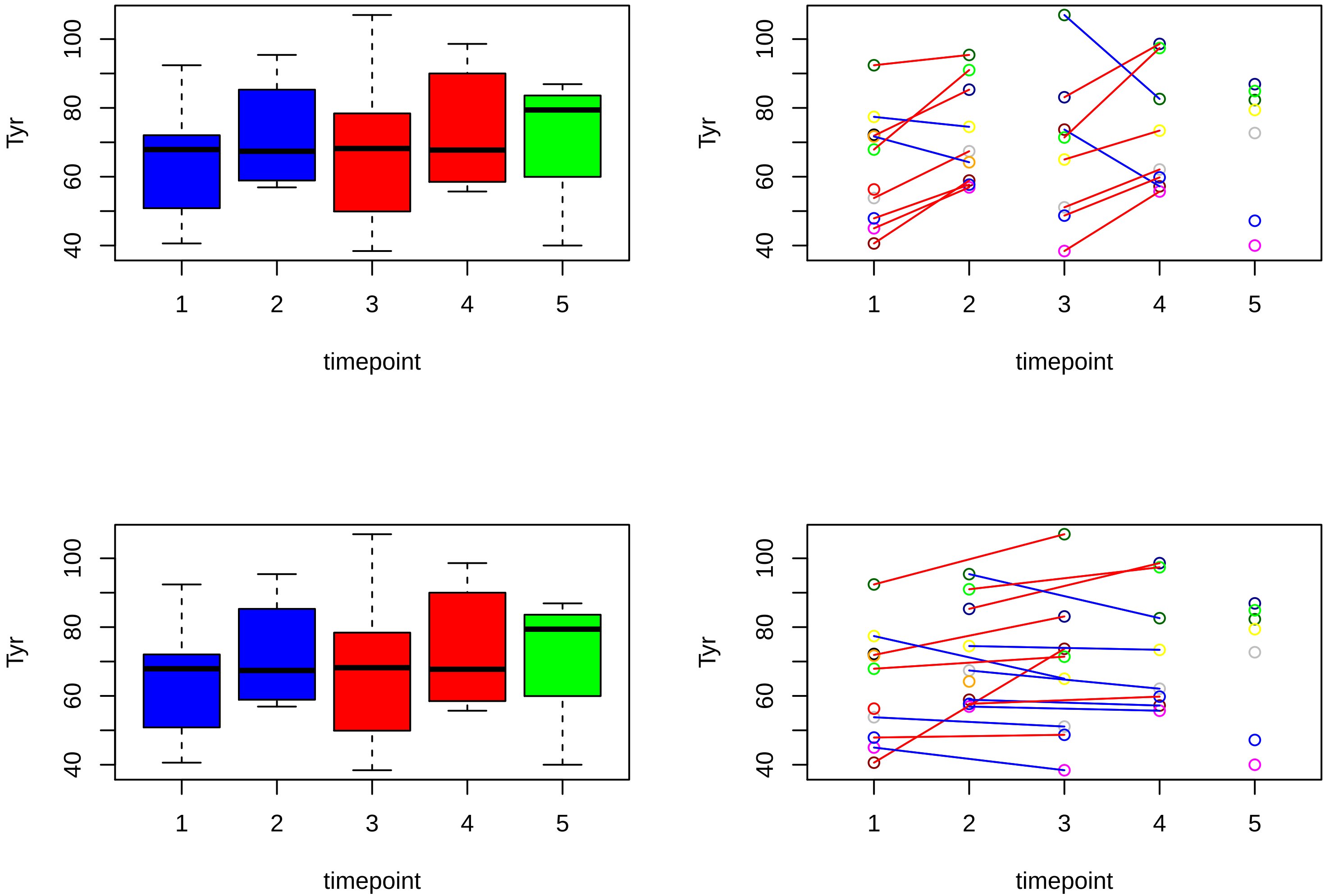
Tyr
100
80
60
40
1
2
3
4
5
timepoint
Tyr
100
80
60
40
1
2
3
4
5
timepoint
Tyr
100
80
60
40
1
2
3
4
5
timepoint
Tyr
100
80
60
40
1
2
3
4
5
timepoint

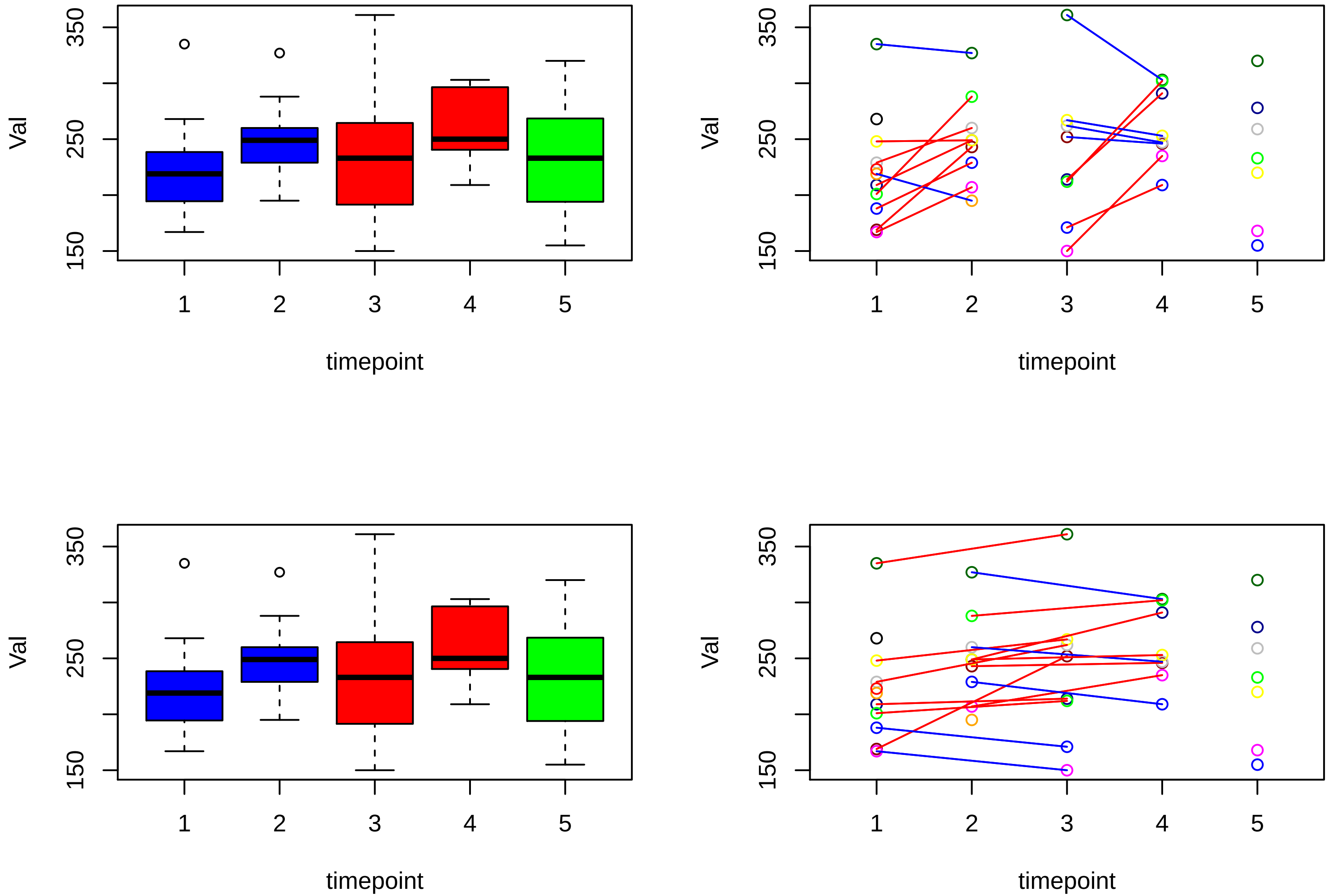

Val
150
250
350
1
2
3
4
5
timepoint

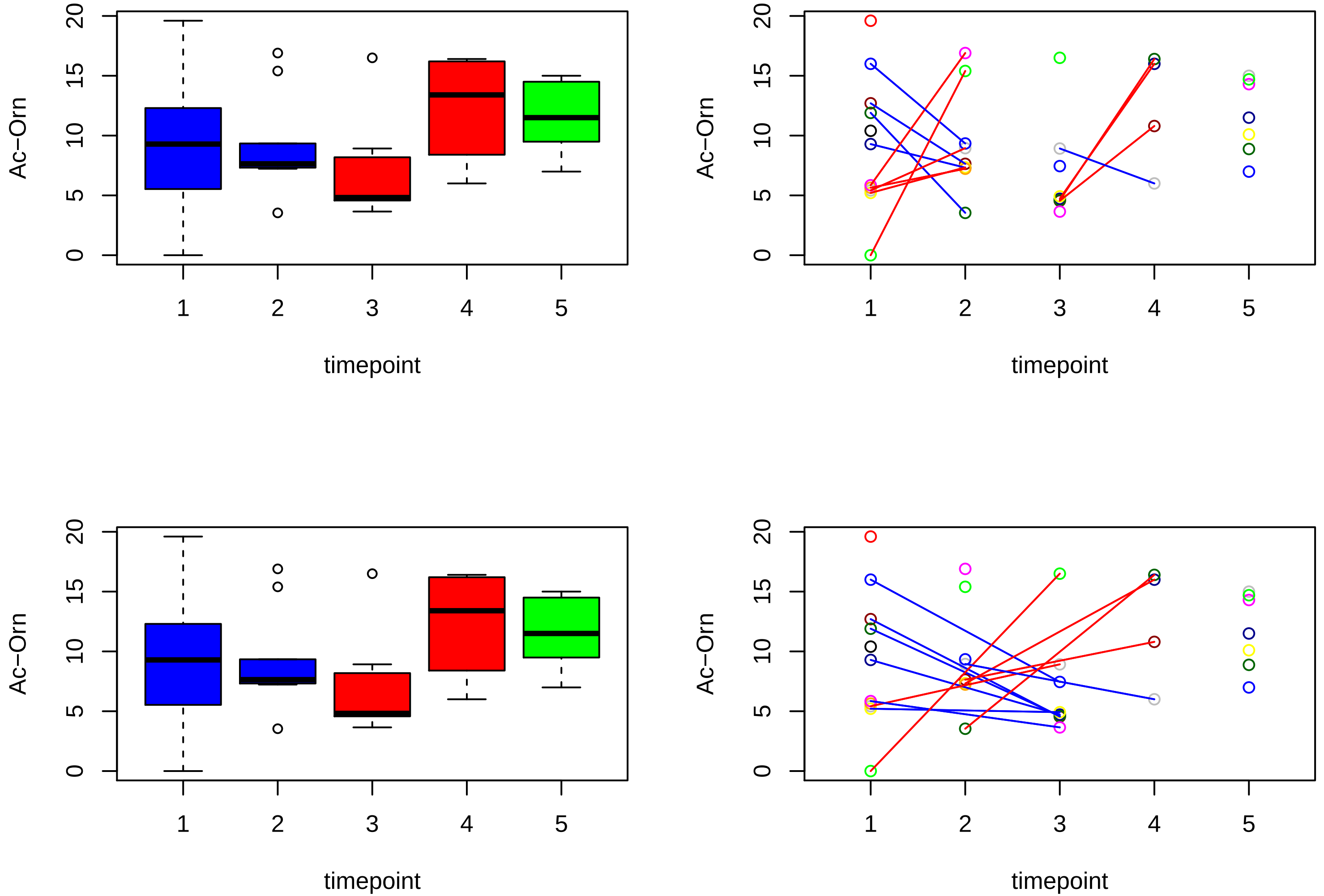
Ac−Orn
timepoint
0
5
10
15
20
1
2
3
4
5

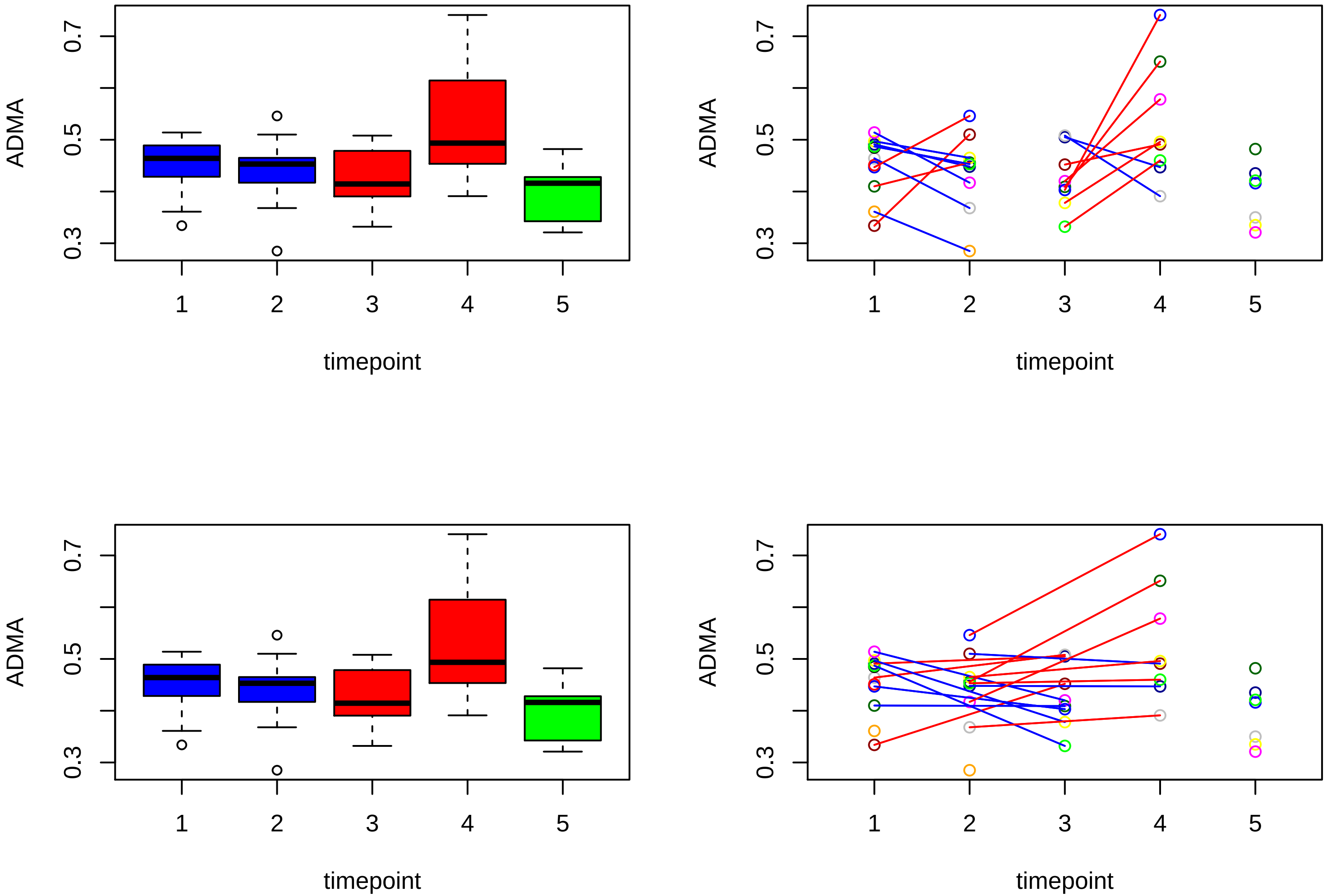

ADMA
0.3
0.5
0.7
1
2
3
4
5
timepoint

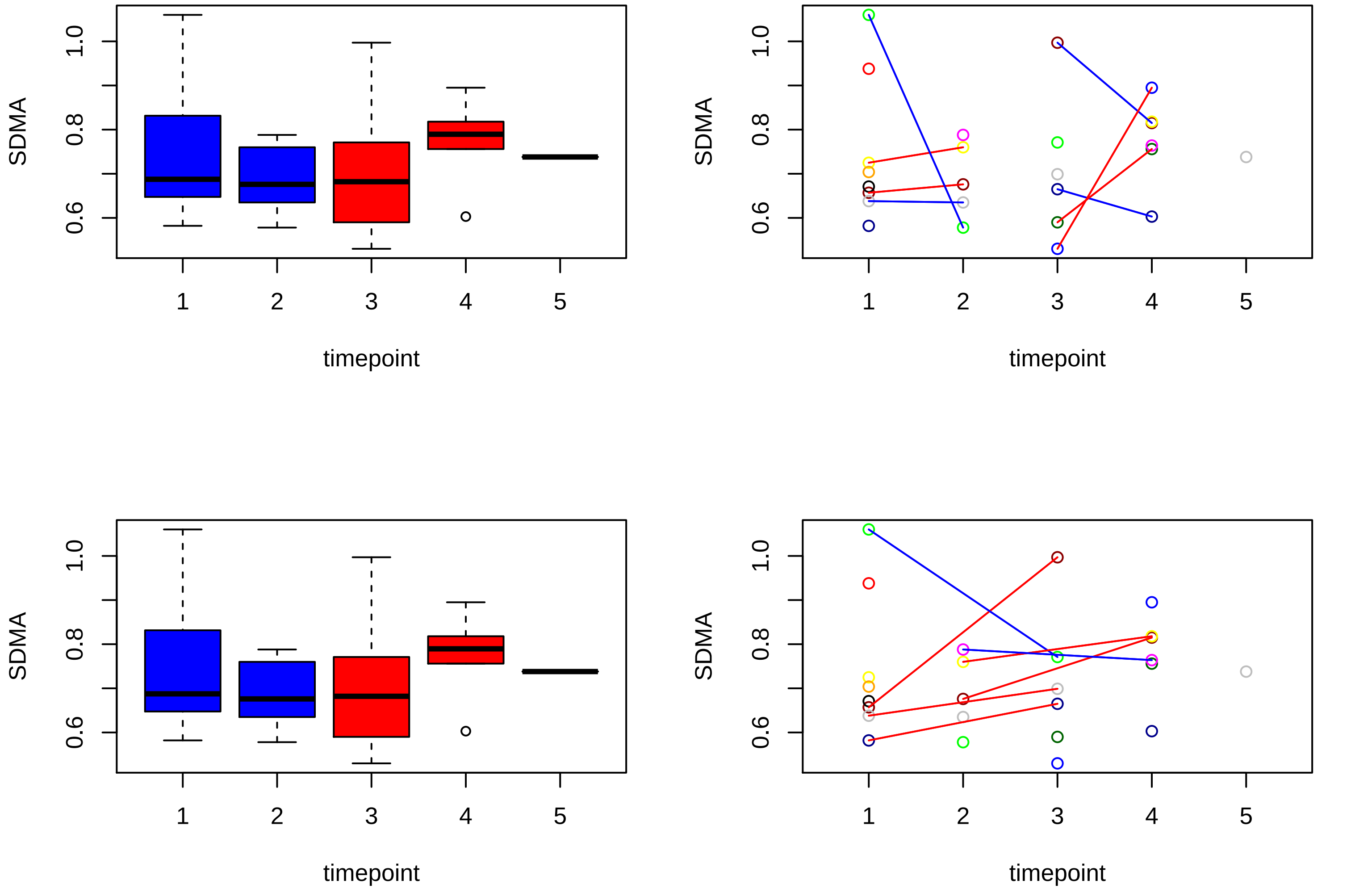
SDMA
1.0
0.8
0.6
1
2
3
4
5
timepoint

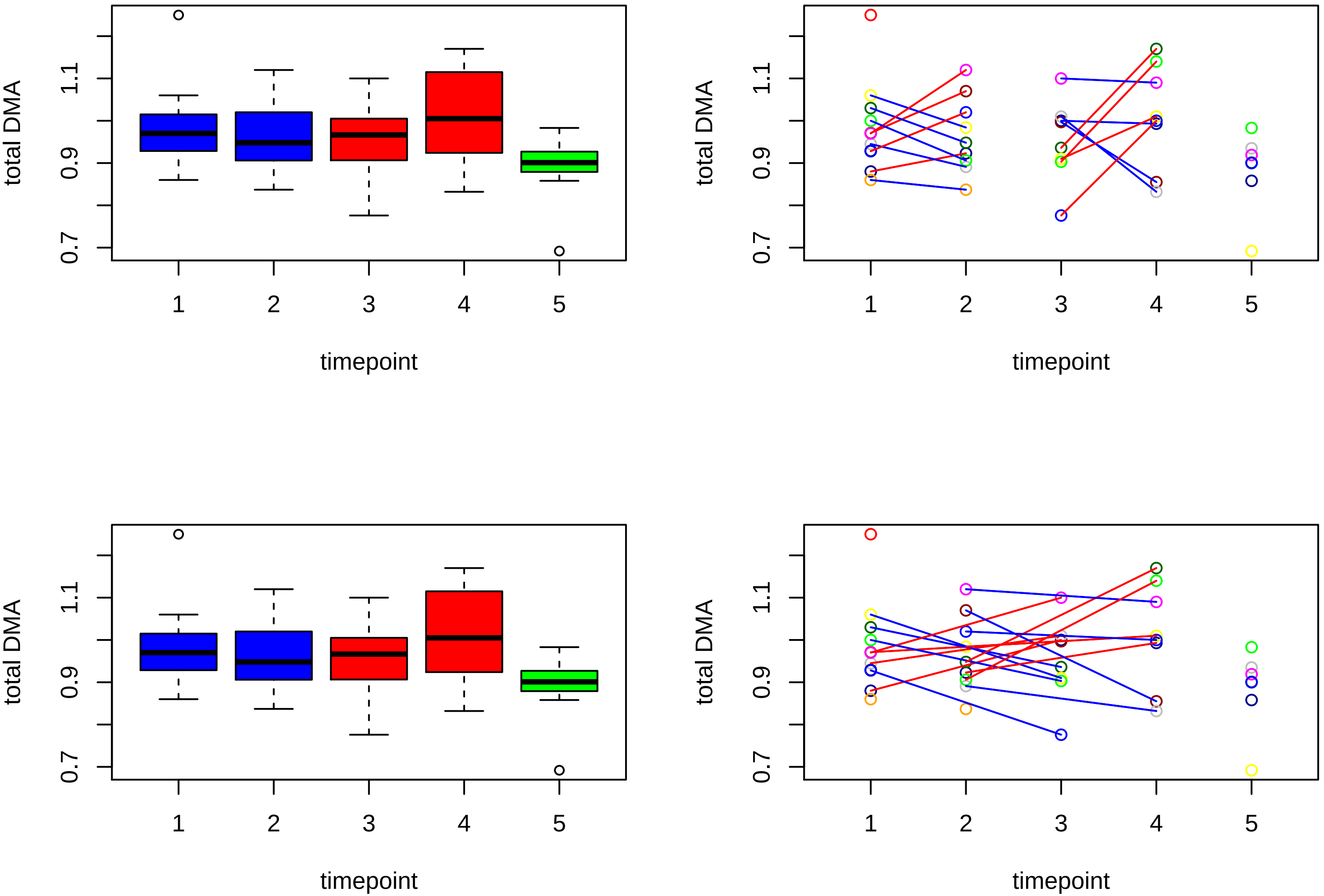
total DMA
0.7
0.9
1.1
1
2
3
4
5
timepoint
total DMA
0.7
0.9
1.1
1
2
3
4
5
timepoint
total DMA
0.7
0.9
1.1
1
2
3
4
5
timepoint
total DMA
0.7
0.9
1.1
1
2
3
4
5
timepoint

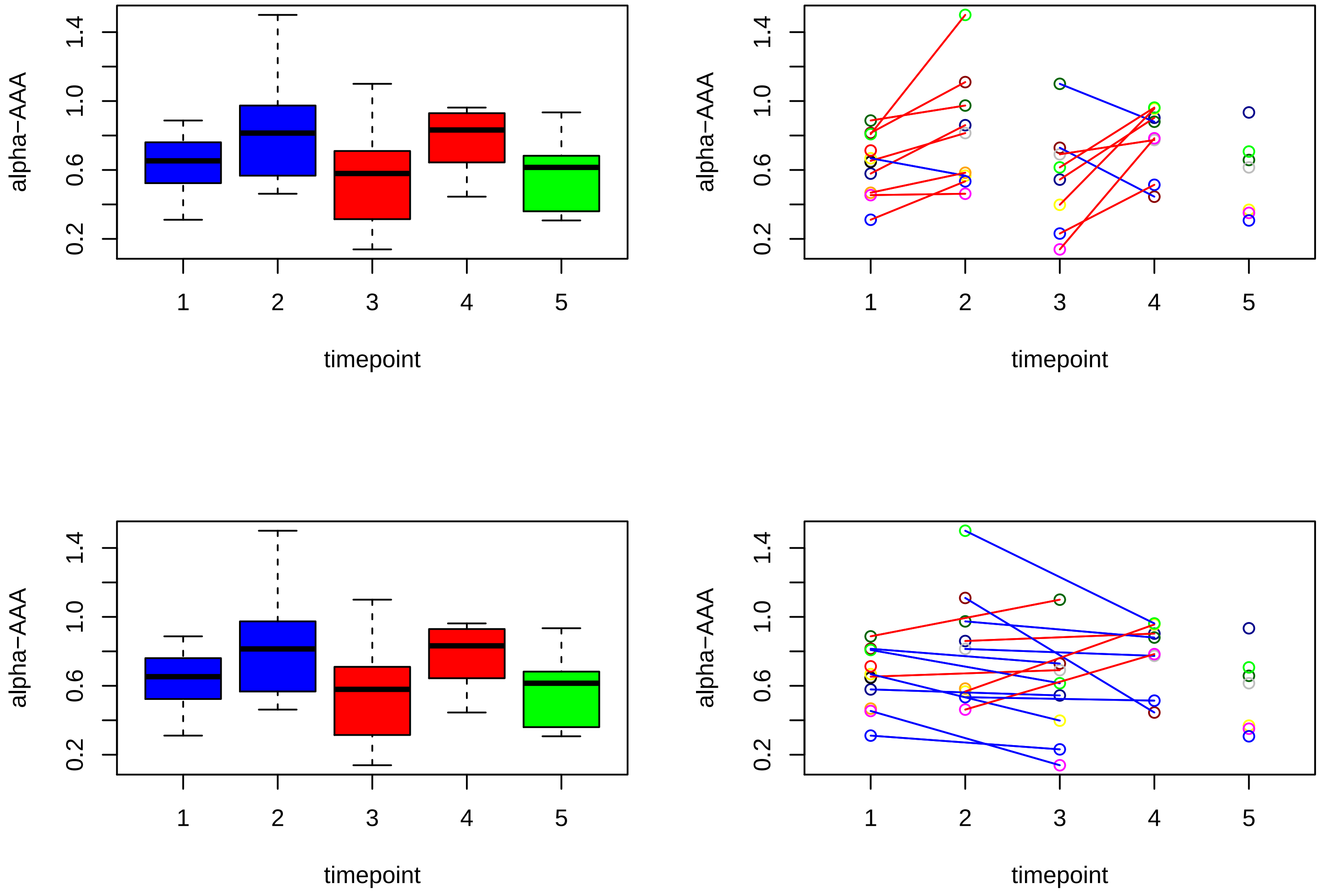

alpha−AAA
timepoint

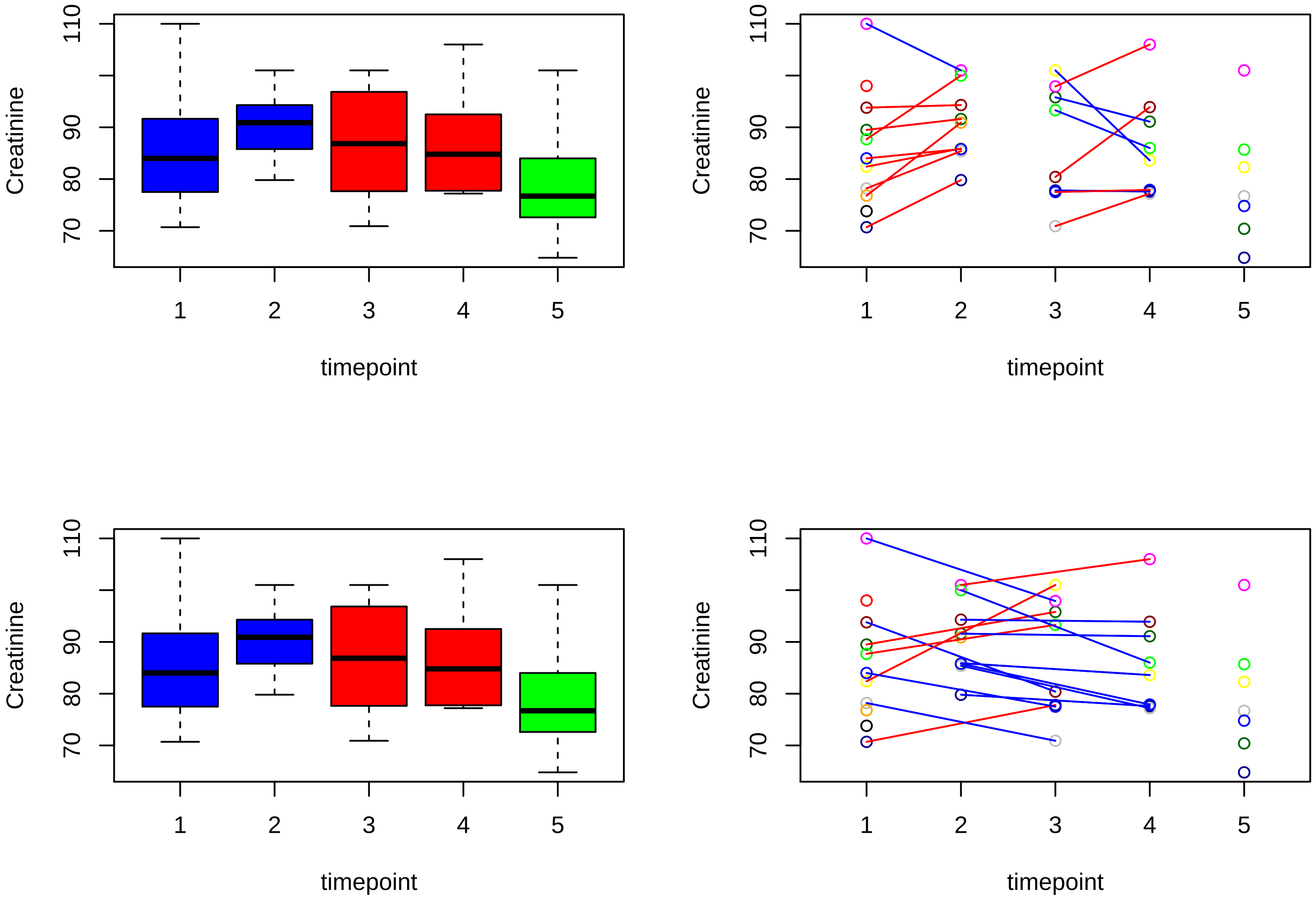

Creatinine
110
90
80
70
1
2
3
4
5
timepoint
Creatinine
110
90
80
70
1
2
3
4
5
timepoint
Creatinine
110
90
80
70
1
2
3
4
5
timepoint
Creatinine
110
90
80
70
1
2
3
4
5
timepoint

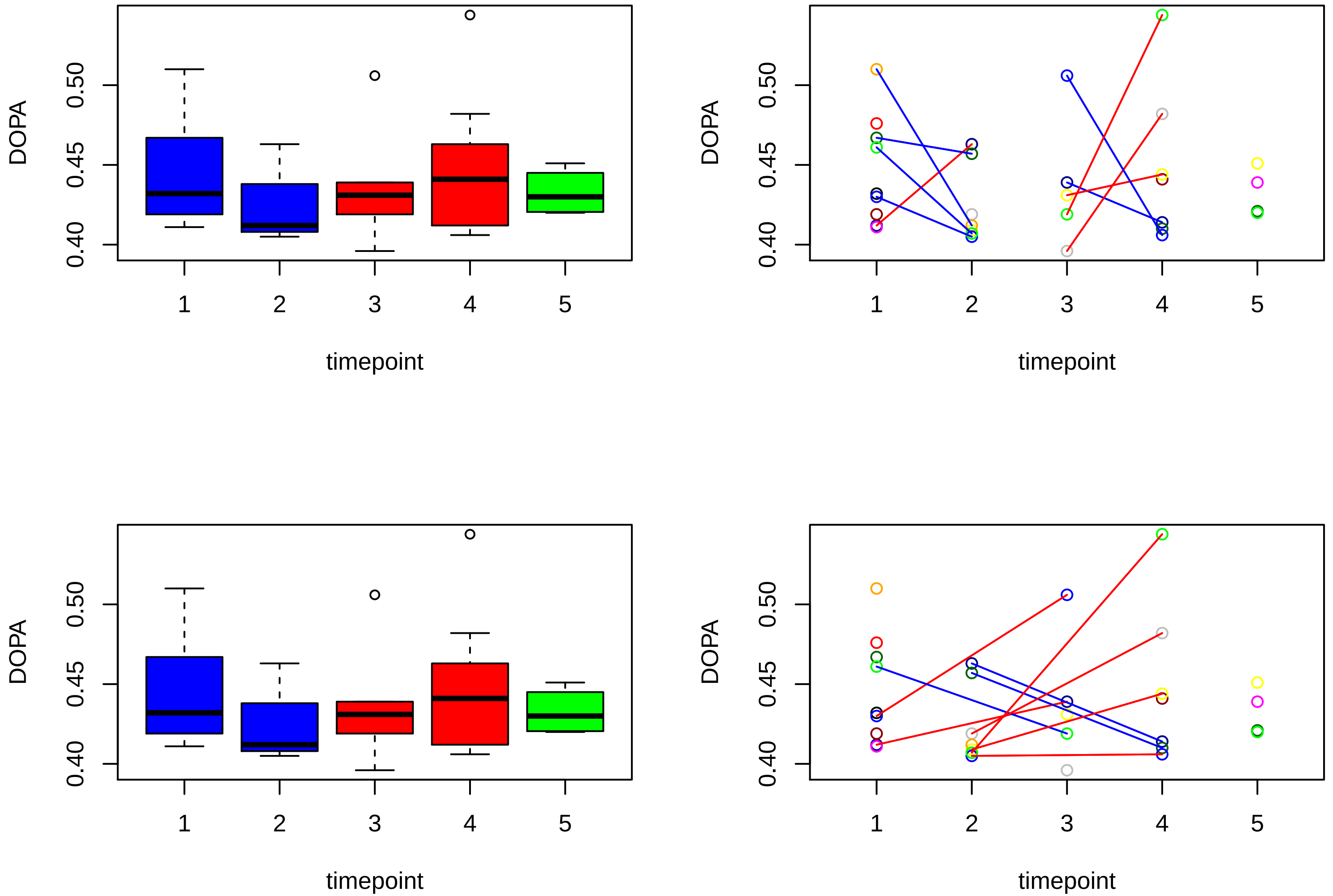

DOPA
0.40
0.45
0.50
1
2
3
4
5
timepoint

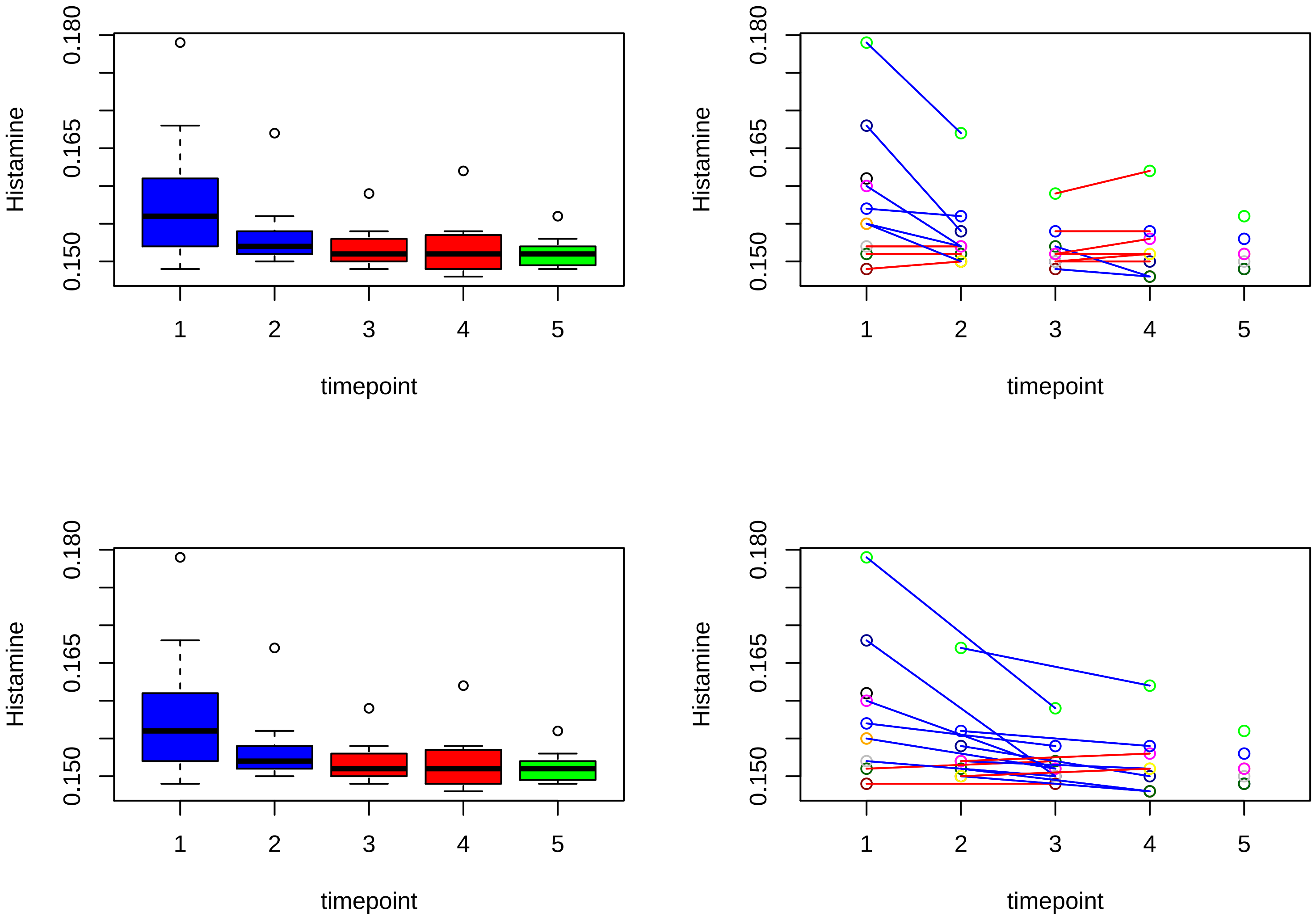
Histamine
0.150
0.165
0.180
1
2
3
4
5
timepoint
Histamine
0.150
0.165
0.180
1
2
3
4
5
timepoint
Histamine
0.150
0.165
0.180
1
2
3
4
5
timepoint
Histamine
0.150
0.165
0.180
1
2
3
4
5
timepoint

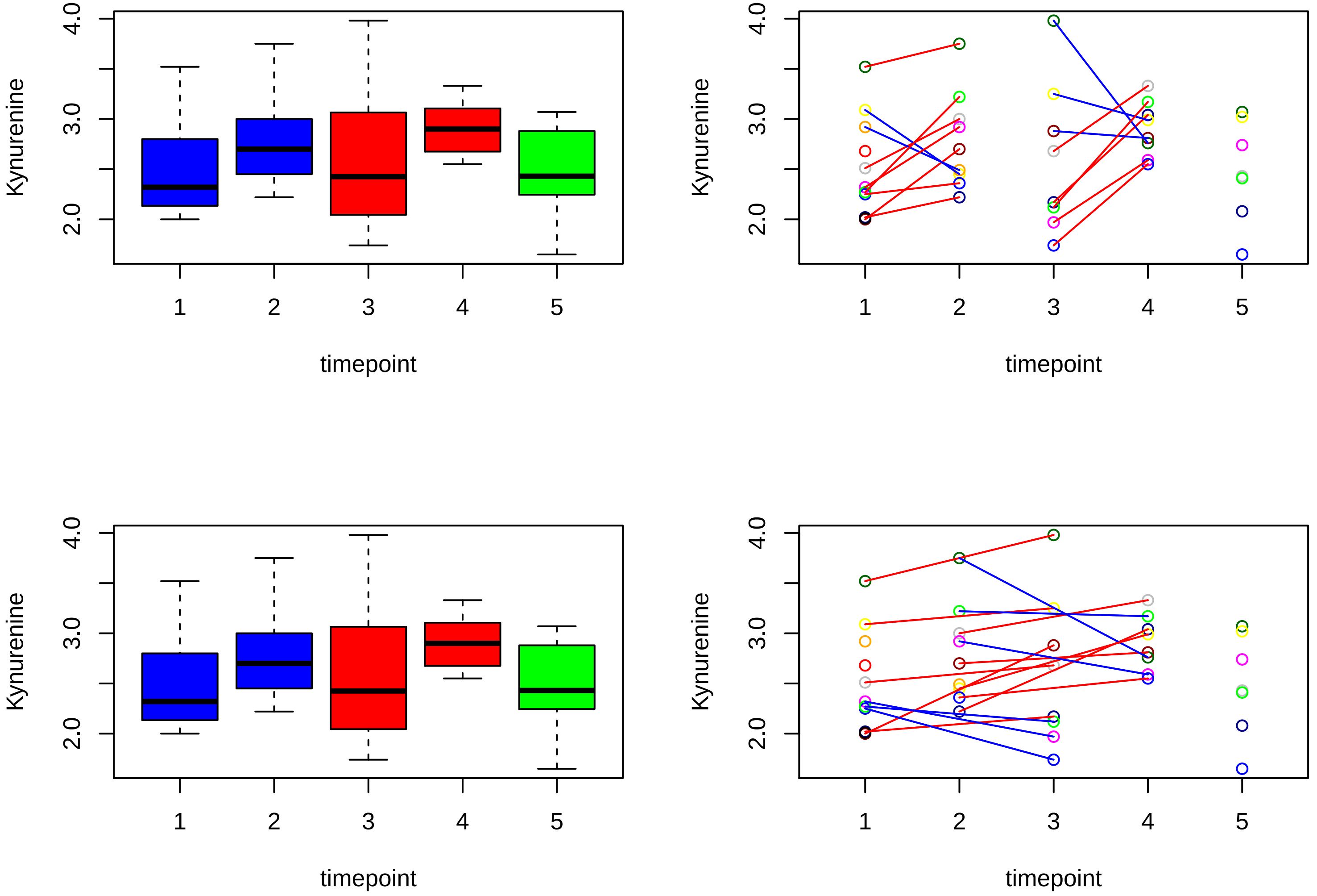

Kynurenine
timepoint
4.0
3.0
2.0
1
2
3
4
5

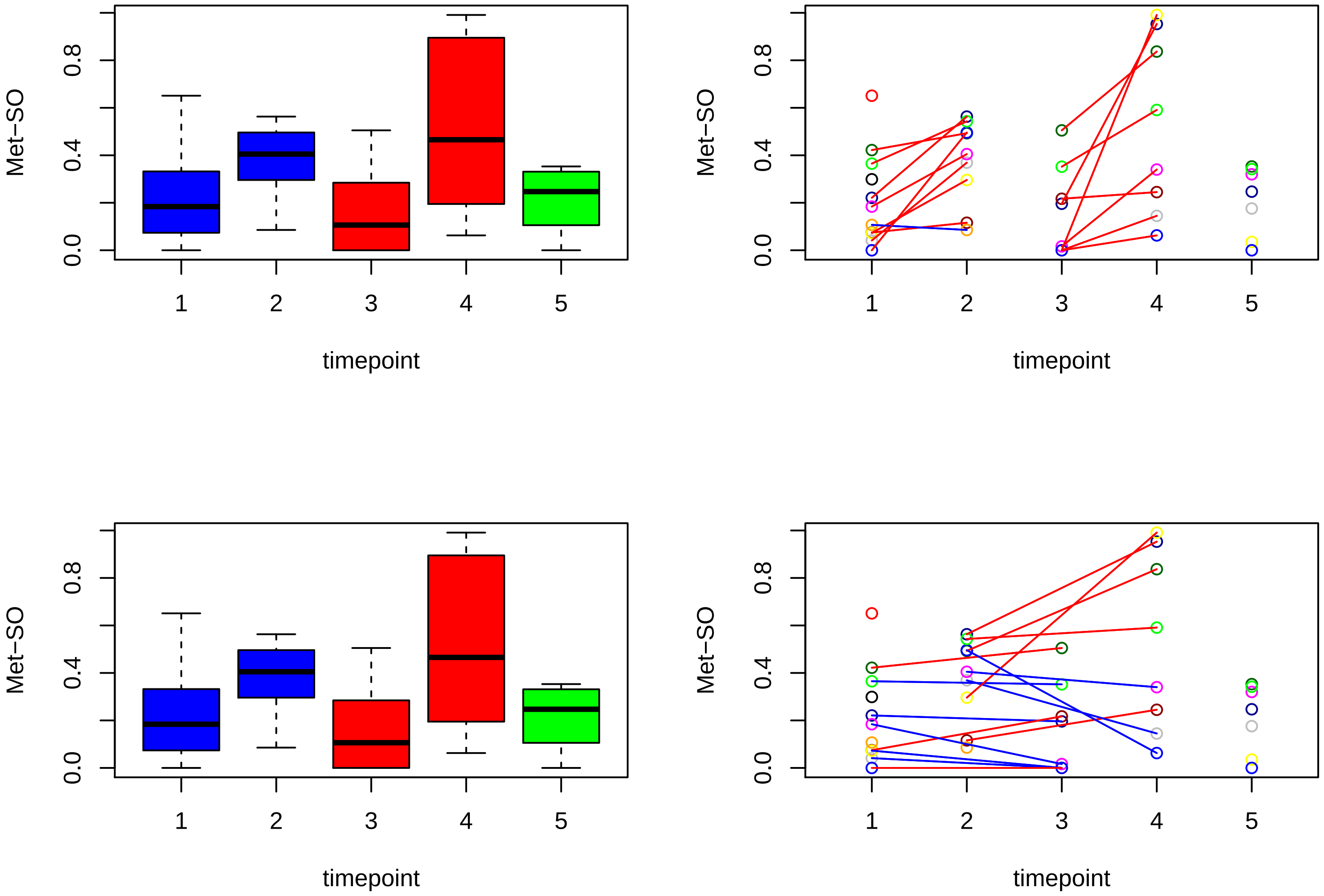

Met−SO
0.0
0.4
0.8
1
2
3
4
5
timepoint

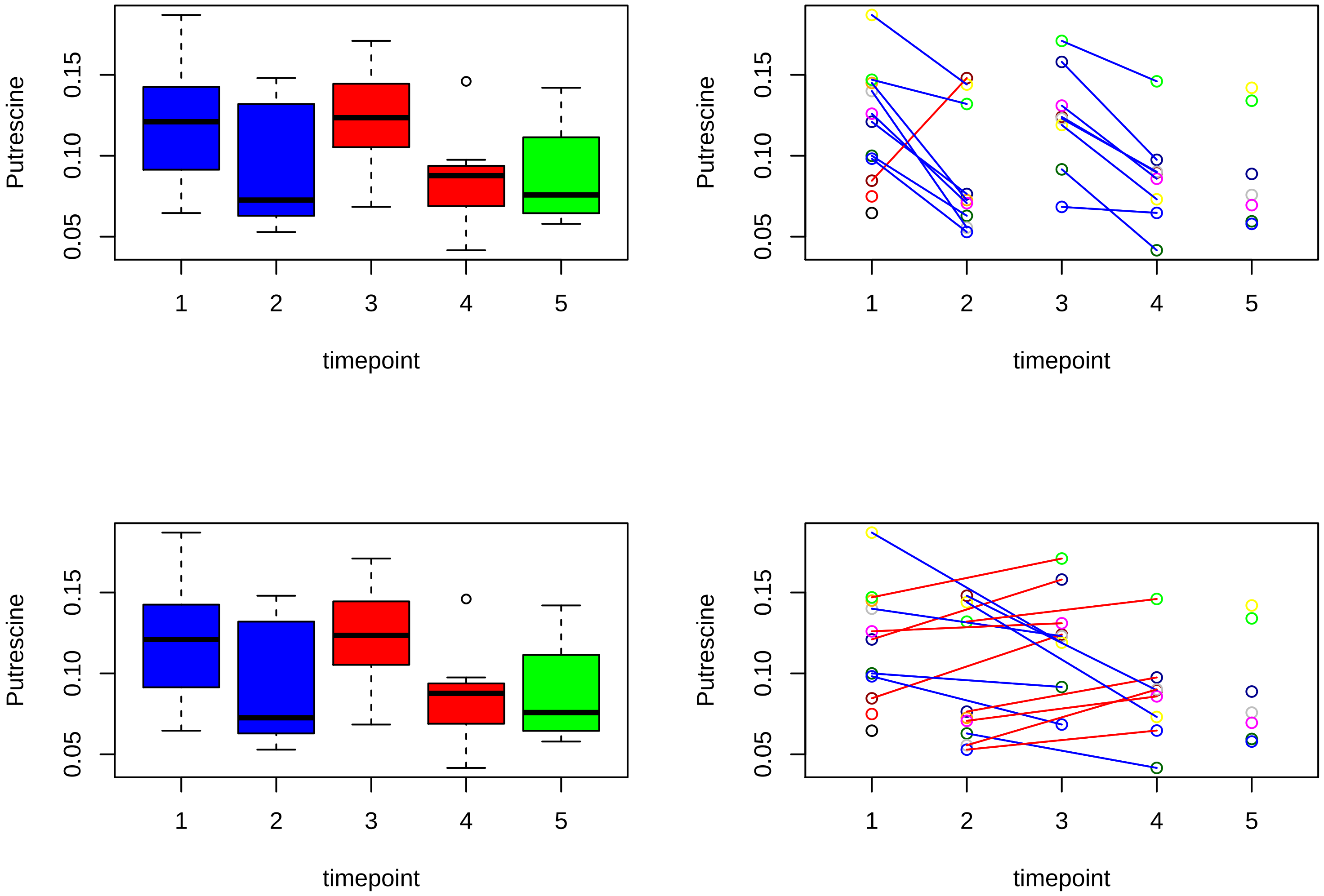

Putrescine
0.05
0.10
0.15
1
2
3
4
5
timepoint

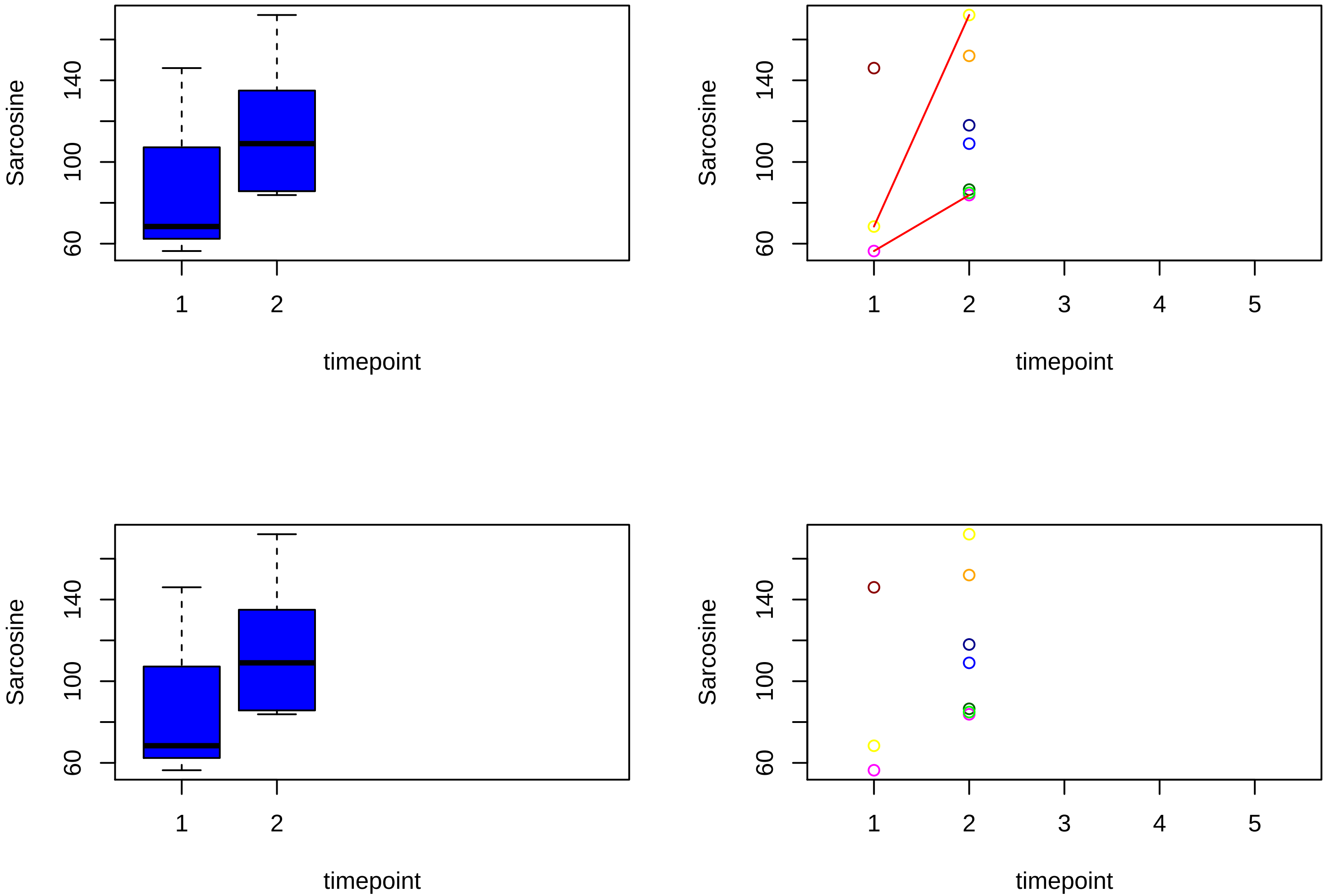
Sarcosine
60
100
140
1
2
timepoint
Sarcosine
60
100
140
1
2
3
4
5
timepoint
Sarcosine
60
100
140
1
2
timepoint
Sarcosine
60
100
140
1
2
3
4
5
timepoint

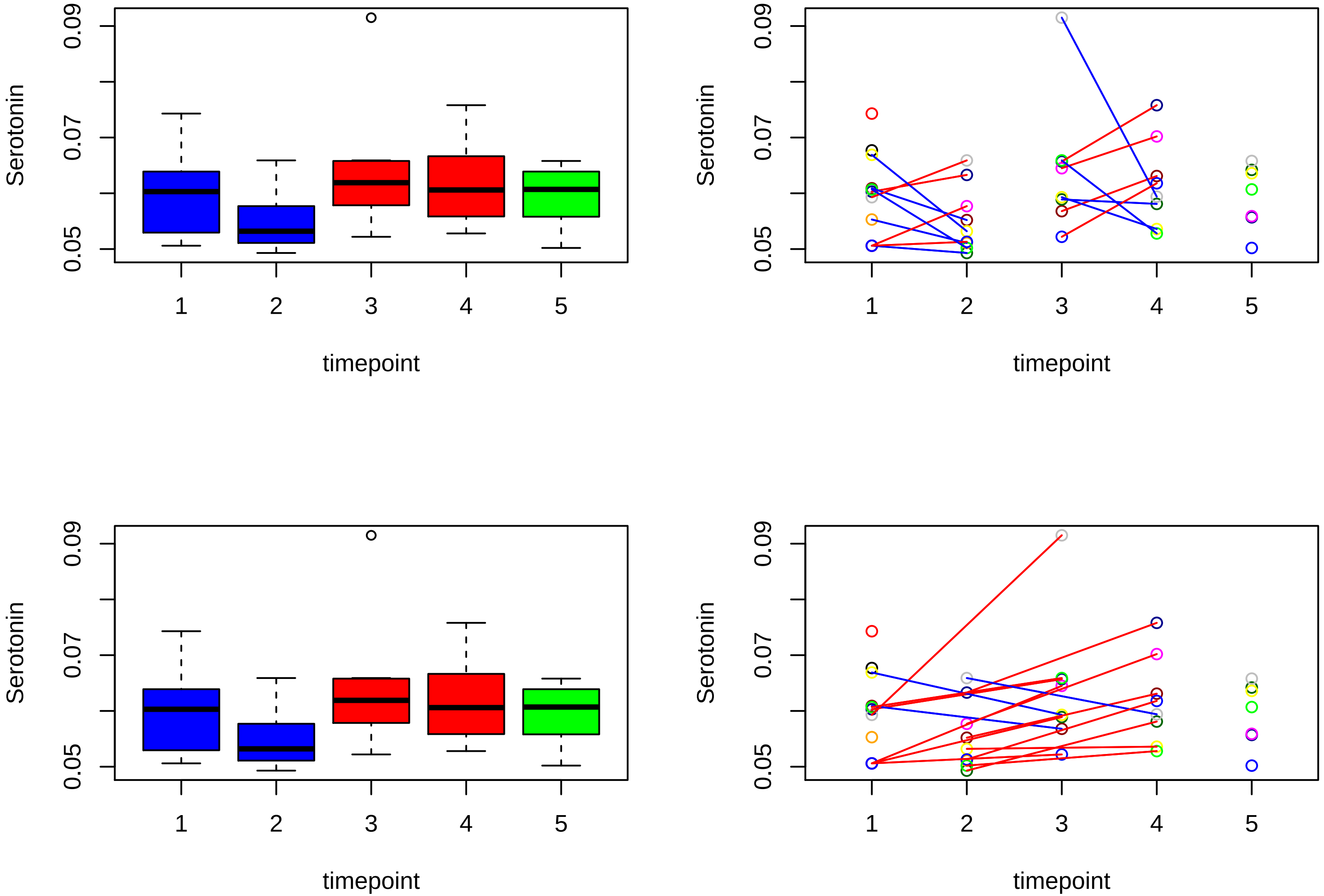
Serotonin
0.05
0.07
0.09
1
2
3
4
5
timepoint
Serotonin
0.05
0.07
0.09
1
2
3
4
5
timepoint
Serotonin
0.05
0.07
0.09
1
2
3
4
5
timepoint
Serotonin
0.05
0.07
0.09
1
2
3
4
5
timepoint

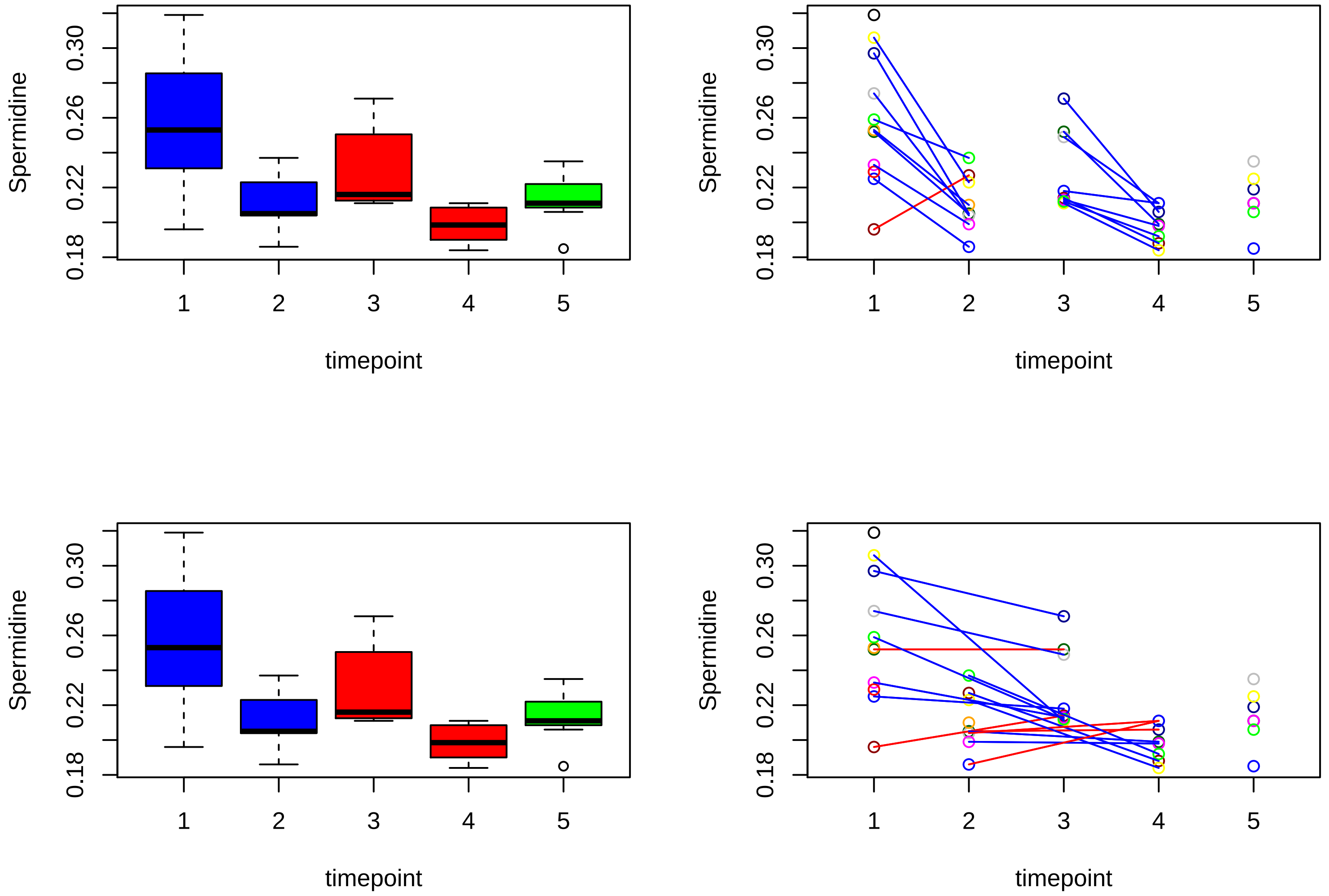
Spermidine
0.18
0.22
0.26
0.30
1
2
3
4
5
timepoint
Spermidine
0.18
0.22
0.26
0.30
1
2
3
4
5
timepoint
Spermidine
0.18
0.22
0.26
0.30
1
2
3
4
5
timepoint
Spermidine
0.18
0.22
0.26
0.30
1
2
3
4
5
timepoint

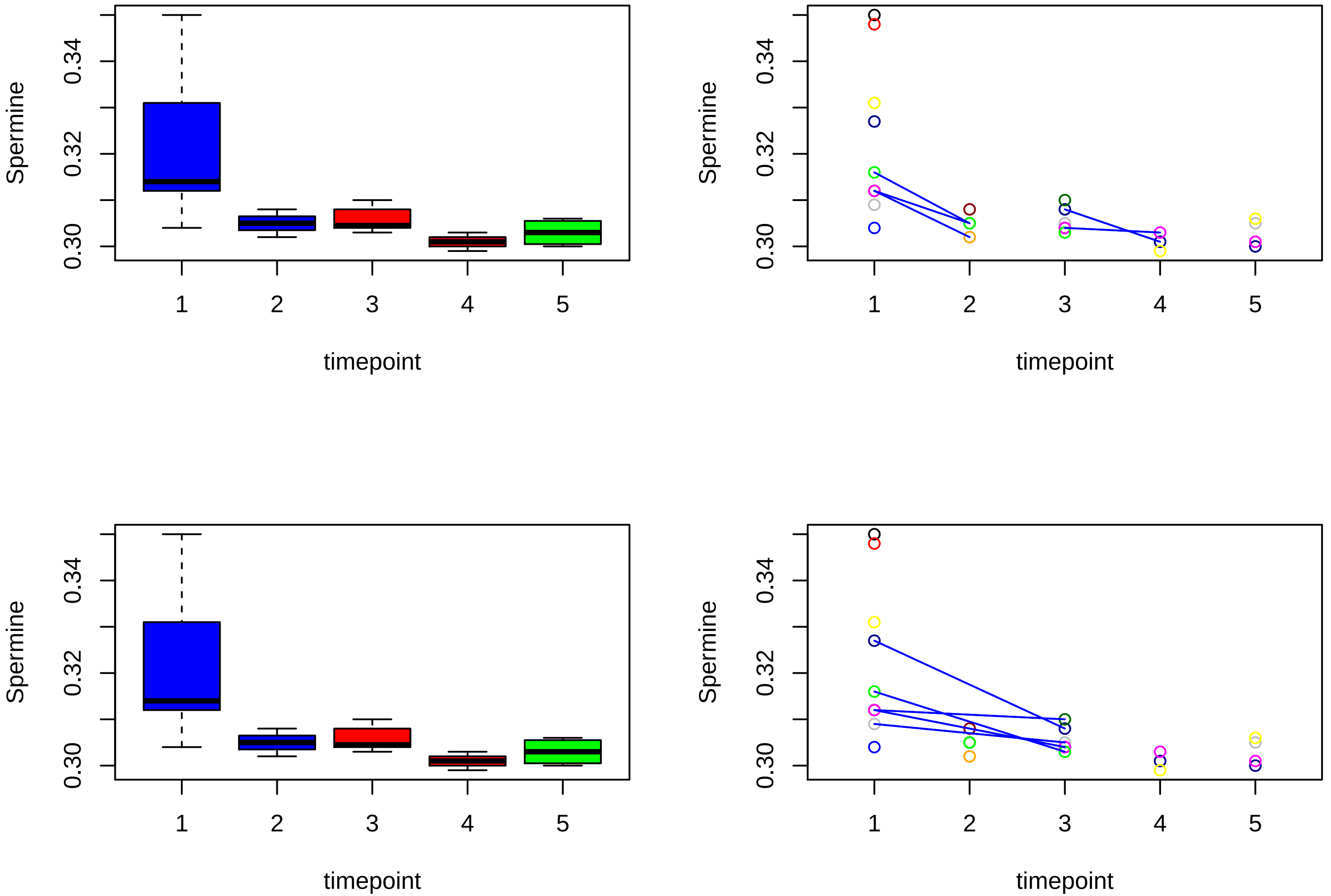

Spermine
0.30
0.32
0.34
1
2
3
4
5
timepoint

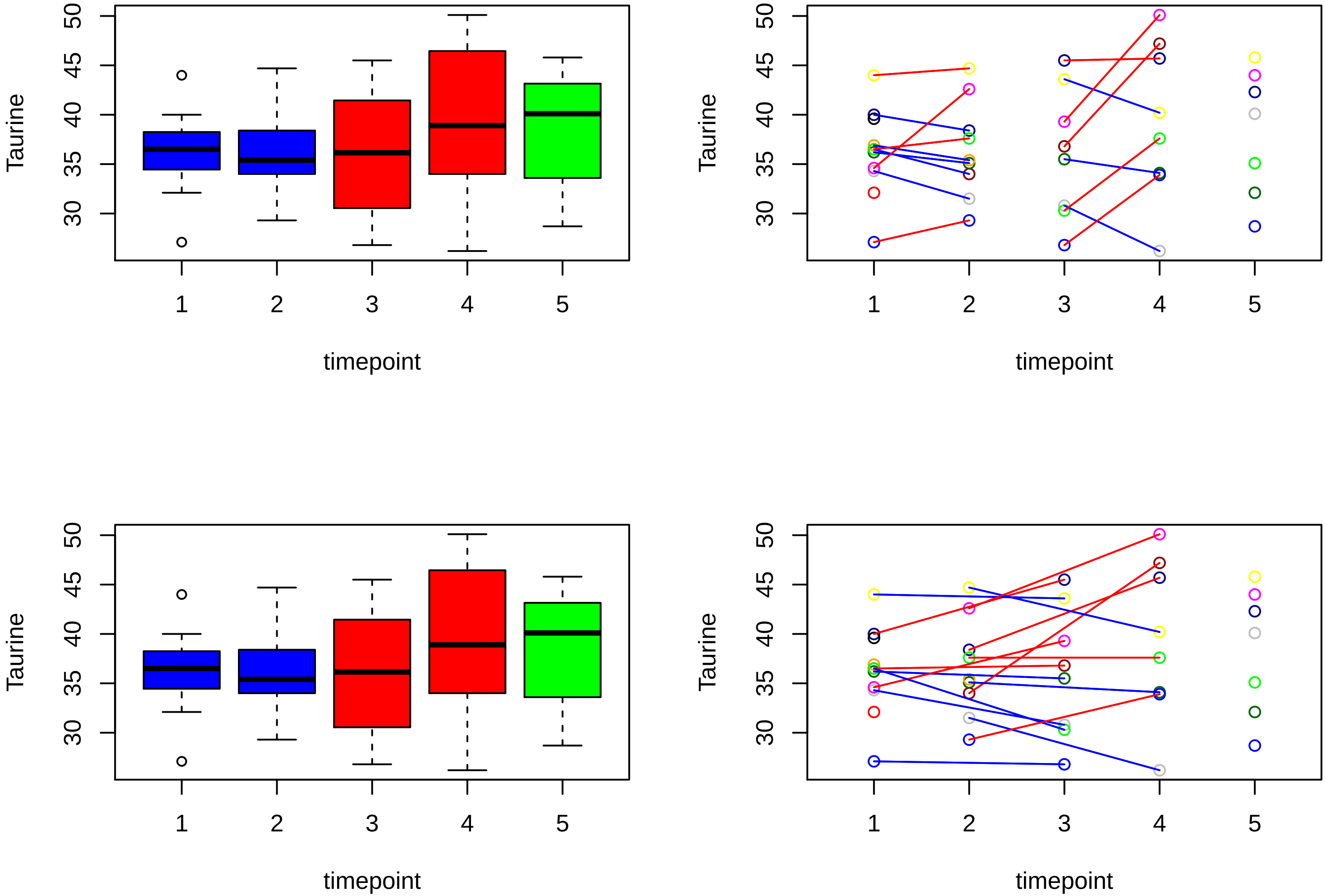

Taurine
50
45
40
35
30
1
2
3
4
5
timepoint

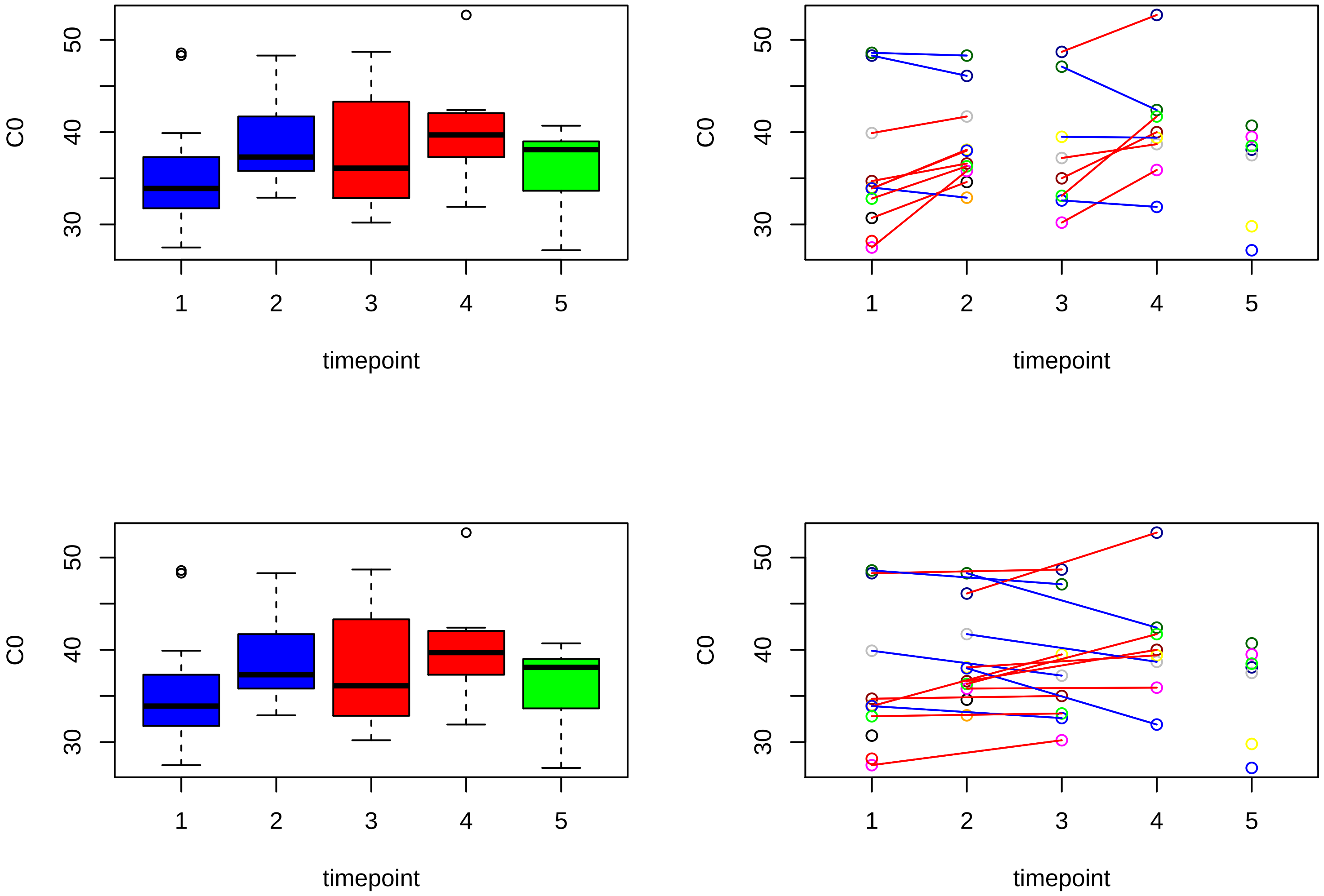

C0
50
40
30
1
2
3
4
5
timepoint

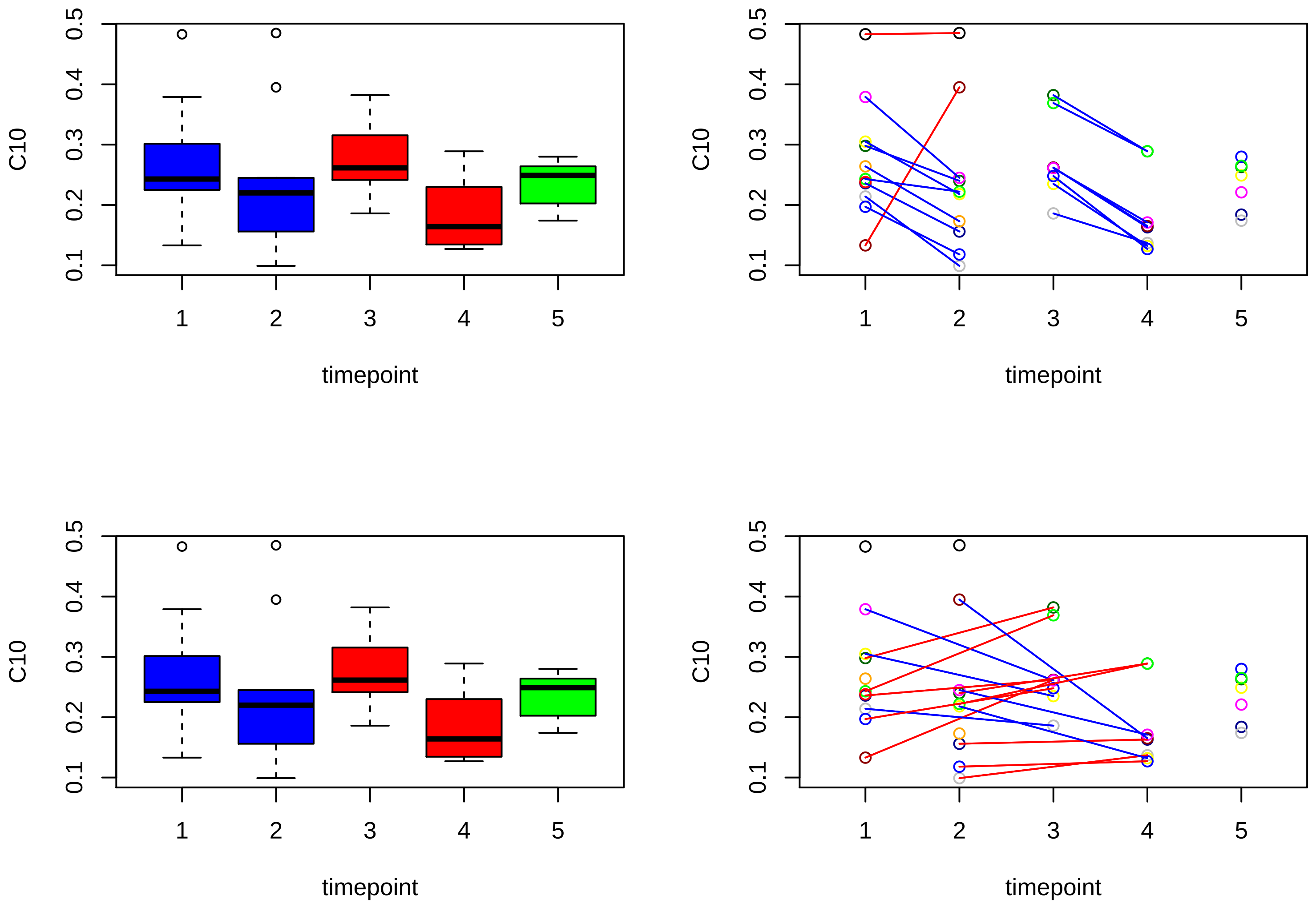
C10
0.1
0.2
0.3
0.4
0.5
1
2
3
4
5
timepoint

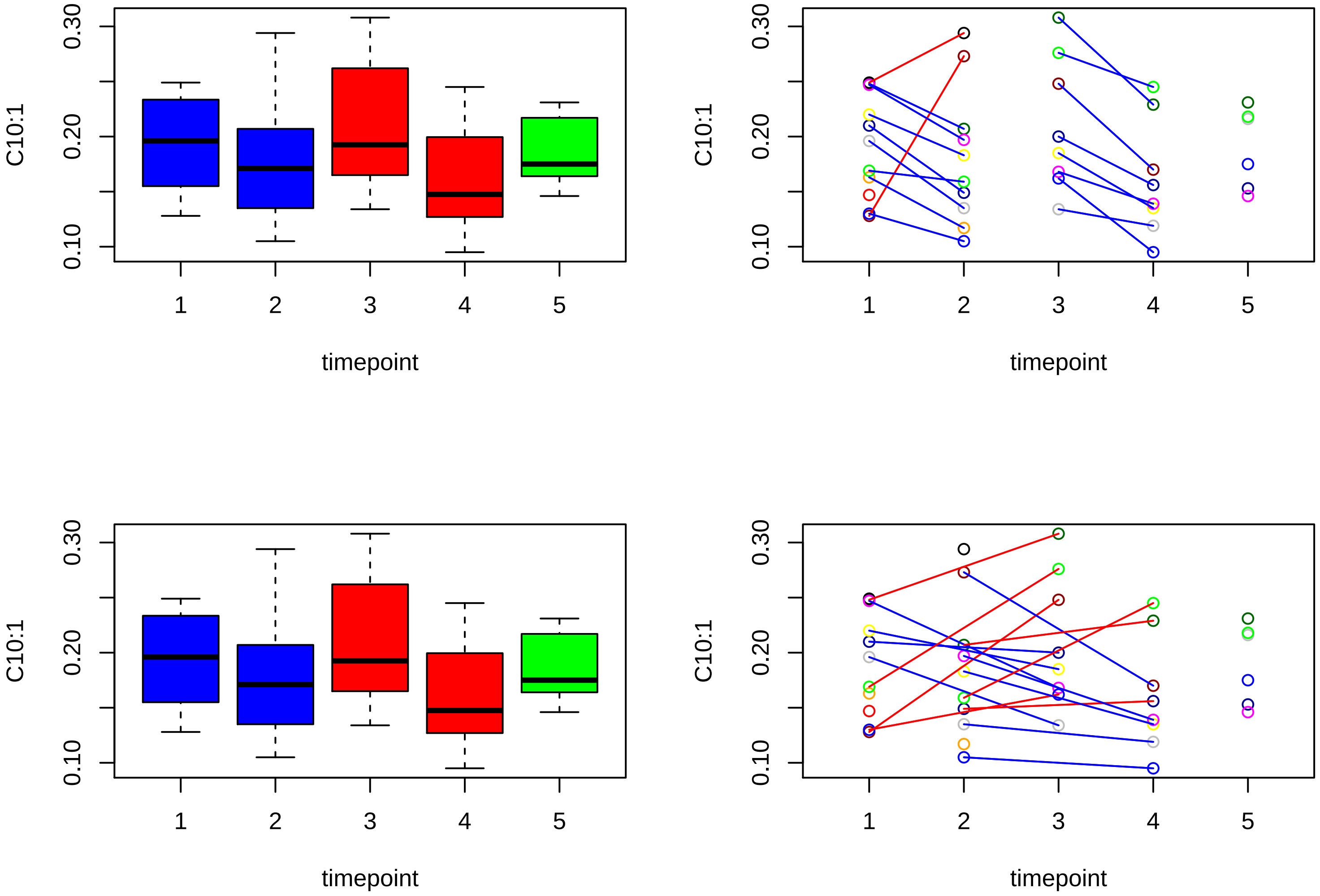

C10:1
0.10
0.20
0.30
1
2
3
4
5
timepoint

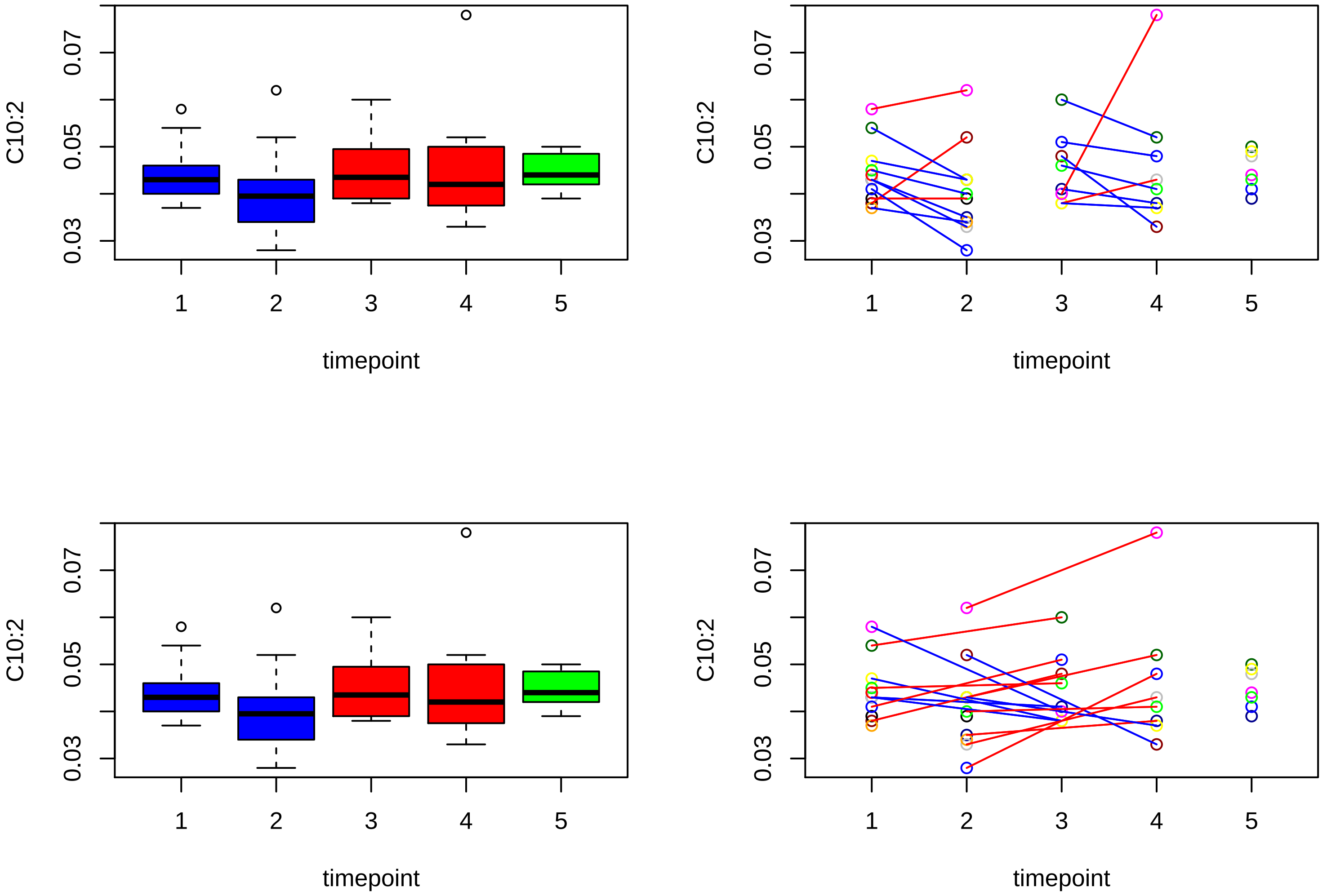
C10:2
0.03
0.05
0.07
1
2
3
4
5
timepoint
C10:2
0.03
0.05
0.07
1
2
3
4
5
timepoint
C10:2
0.03
0.05
0.07
1
2
3
4
5
timepoint
C10:2
0.03
0.05
0.07
1
2
3
4
5
timepoint

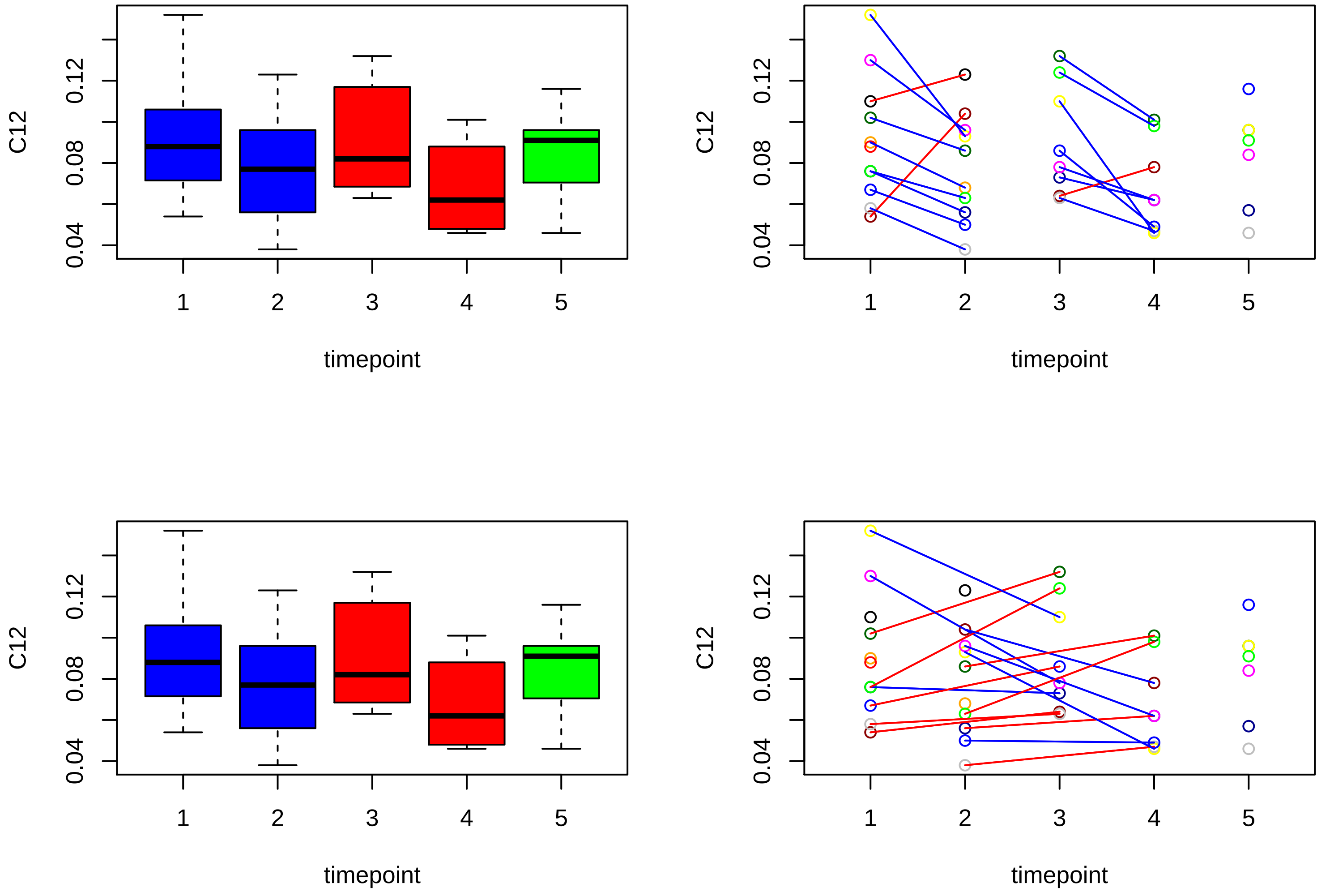

C12
0.04
0.08
0.12
1
2
3
4
5
timepoint

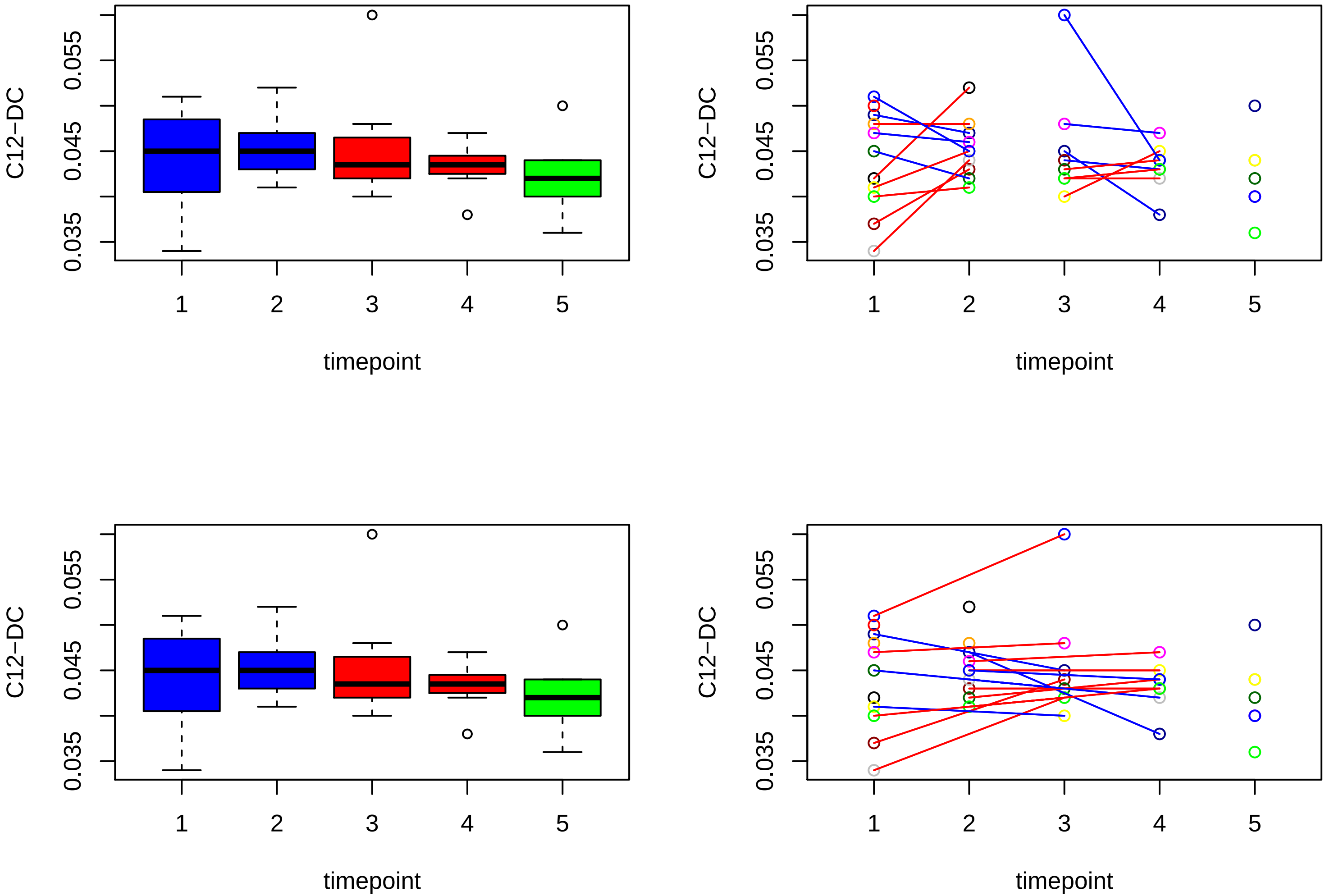
C12−DC
0.035
0.045
0.055
1
2
3
4
5
timepoint

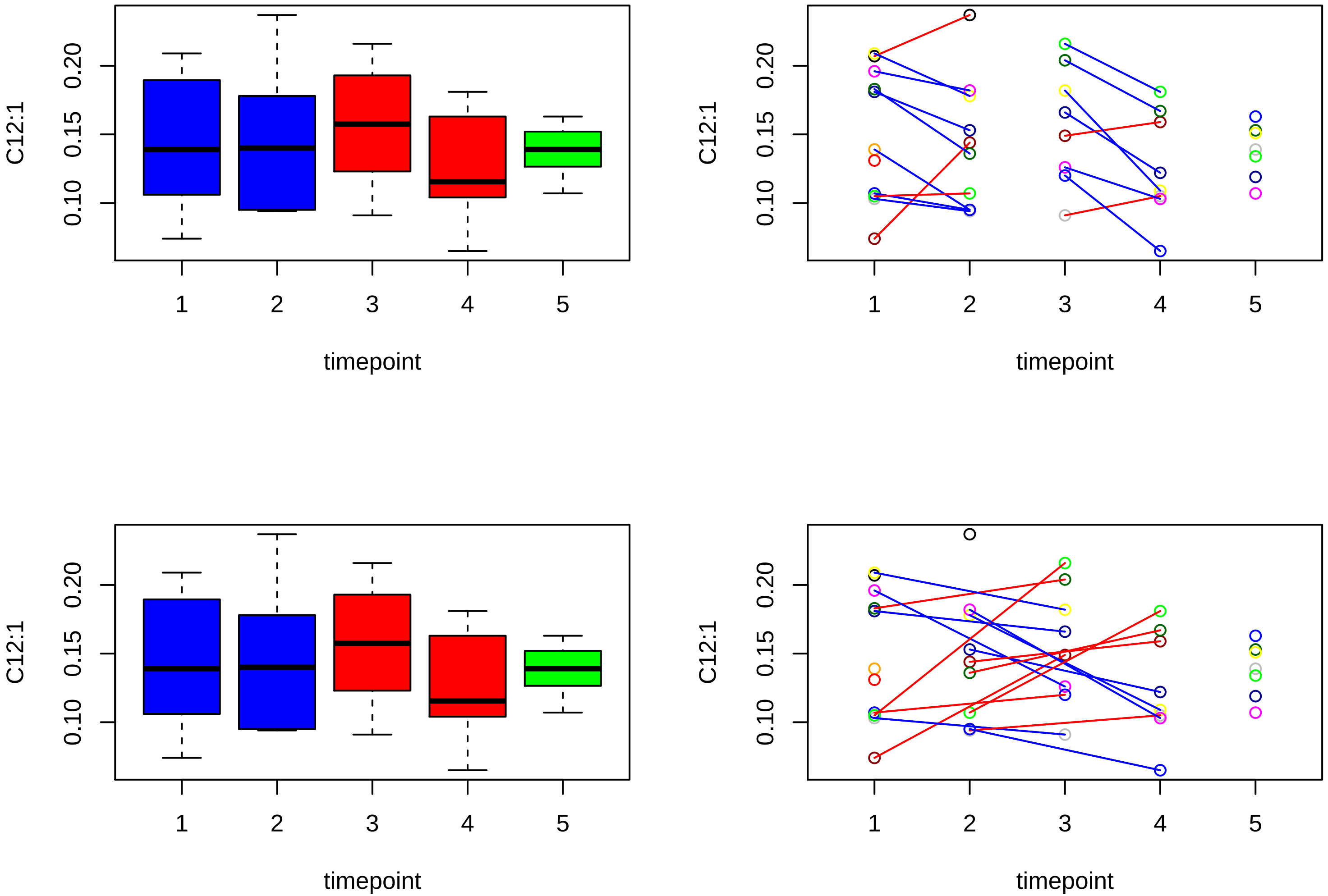
C12:1
0.10
0.15
0.20
1
2
3
4
5
timepoint

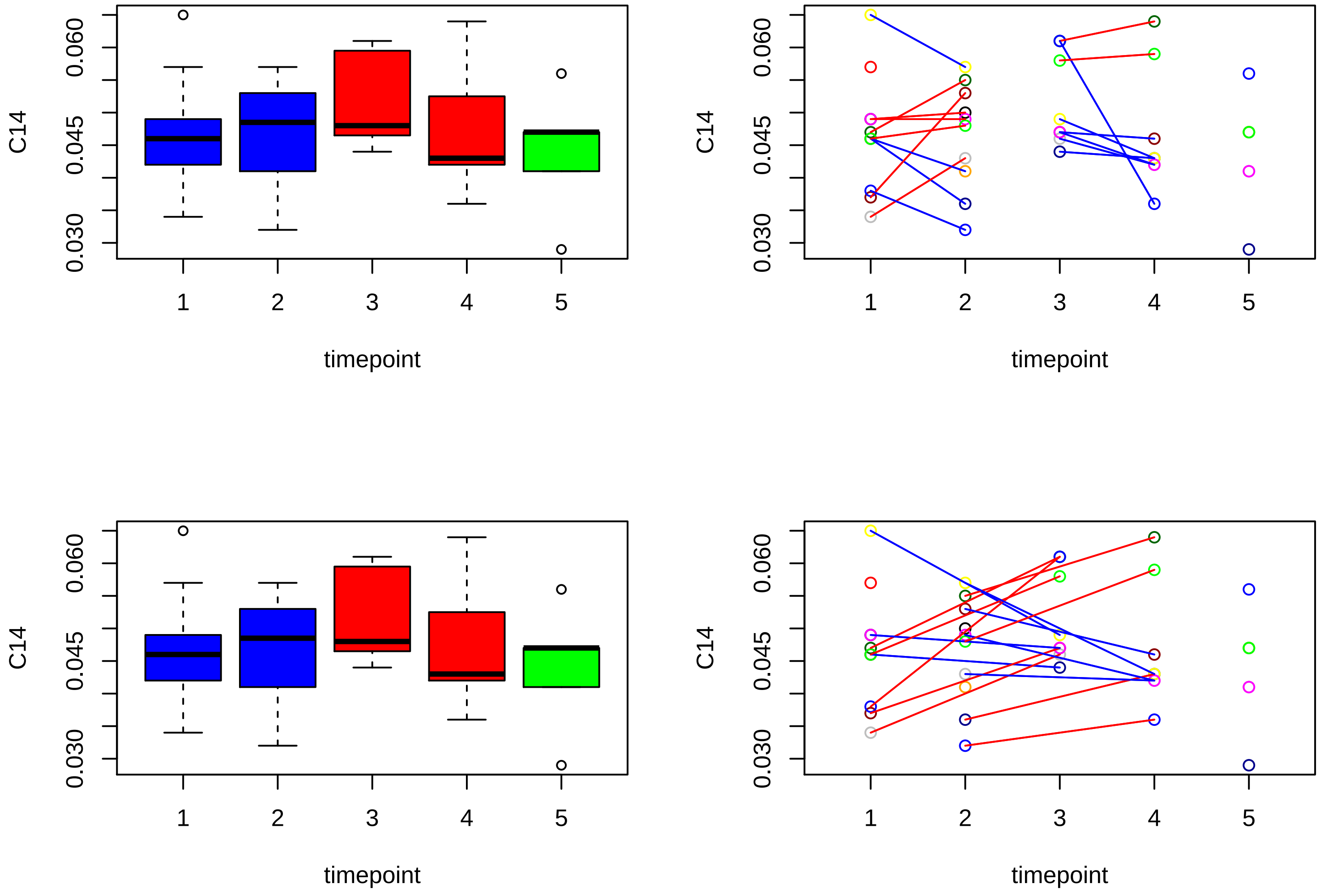

C14
0.030
0.045
0.060
1
2
3
4
5
timepoint

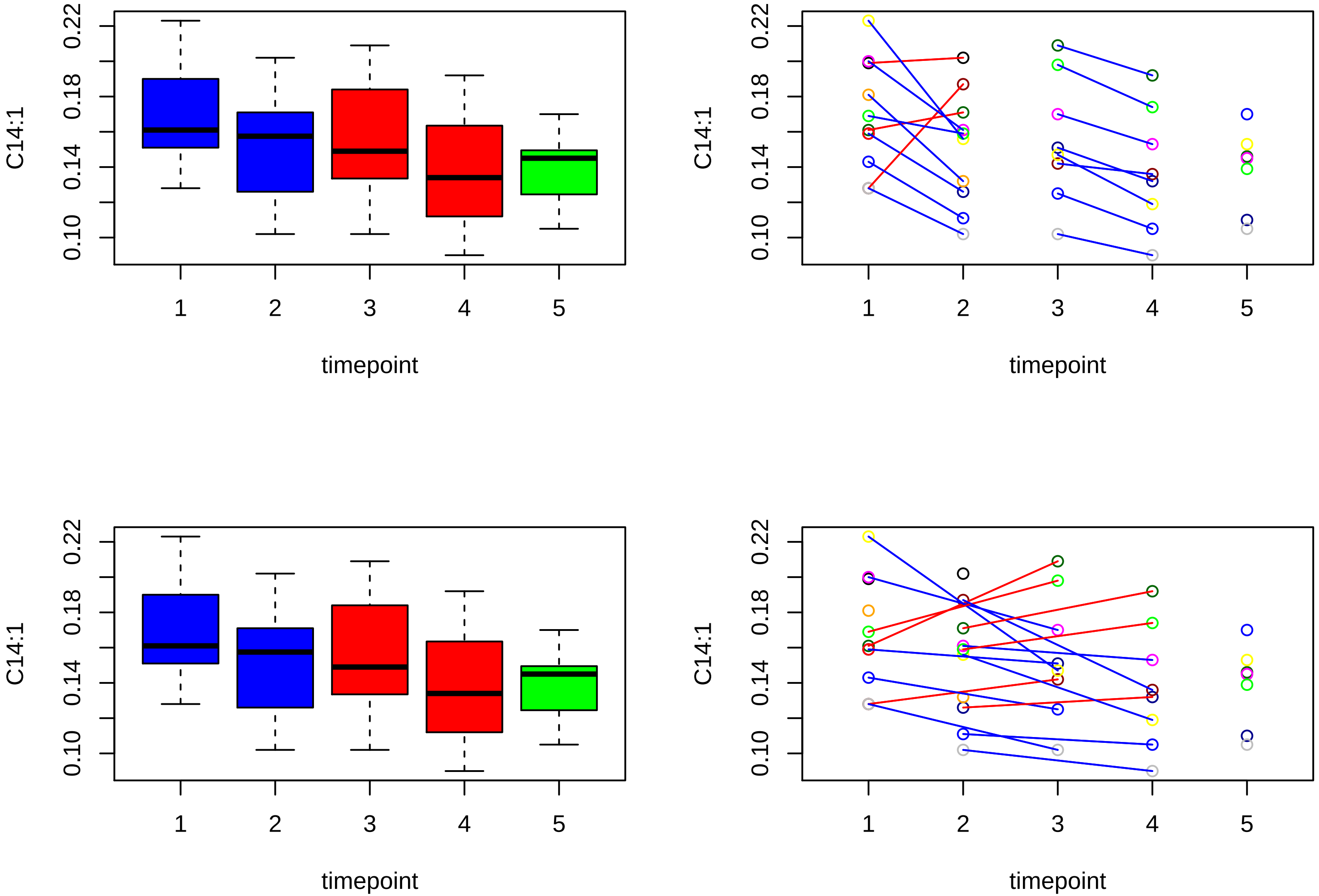
C14:1
0.10
0.14
0.18
0.22
1
2
3
4
5
timepoint
C14:1
0.10
0.14
0.18
0.22
1
2
3
4
5
timepoint
C14:1
0.10
0.14
0.18
0.22
1
2
3
4
5
timepoint
C14:1
0.10
0.14
0.18
0.22
1
2
3
4
5
timepoint

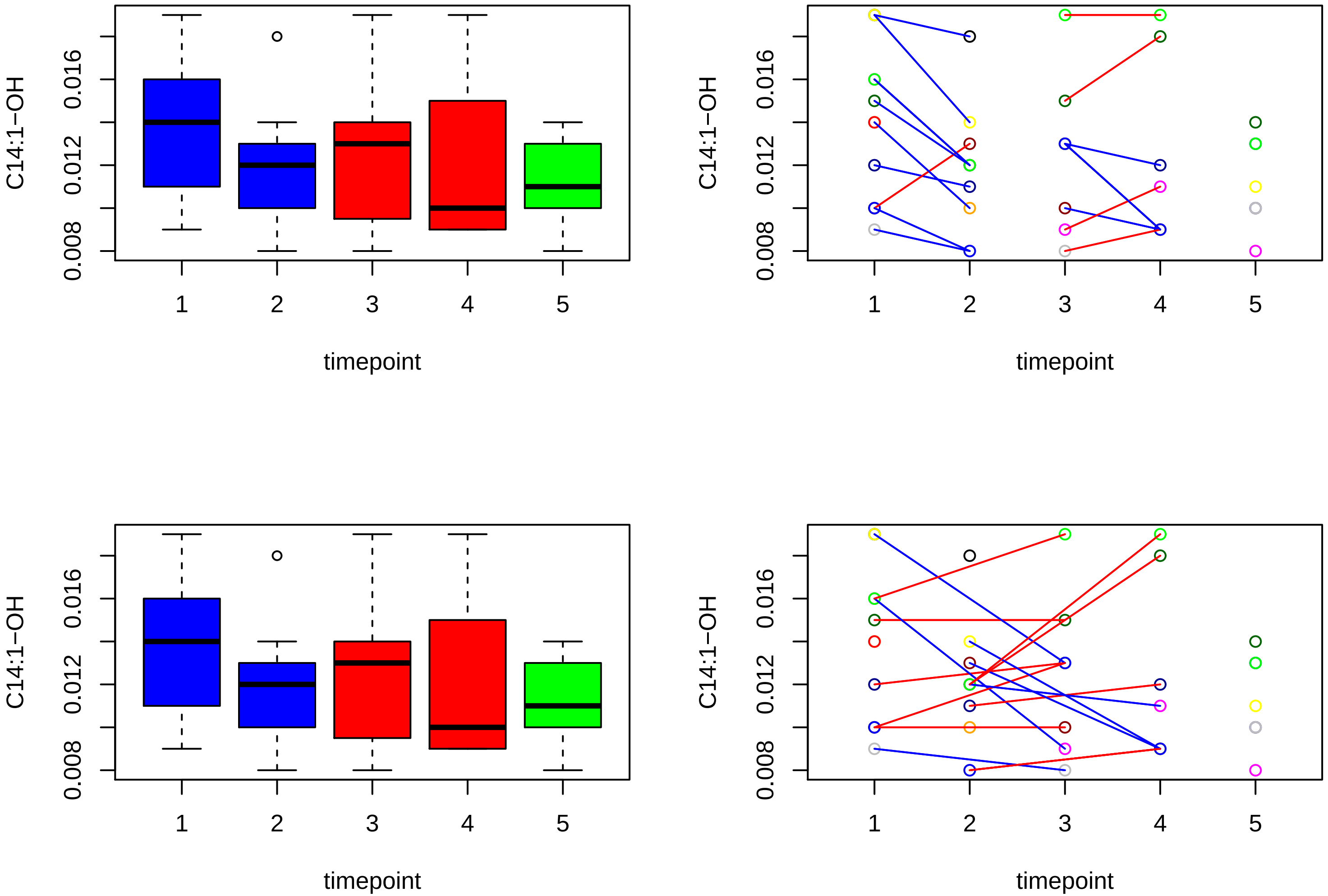
C14:1−OH
0.008
0.012
0.016
1
2
3
4
5
timepoint

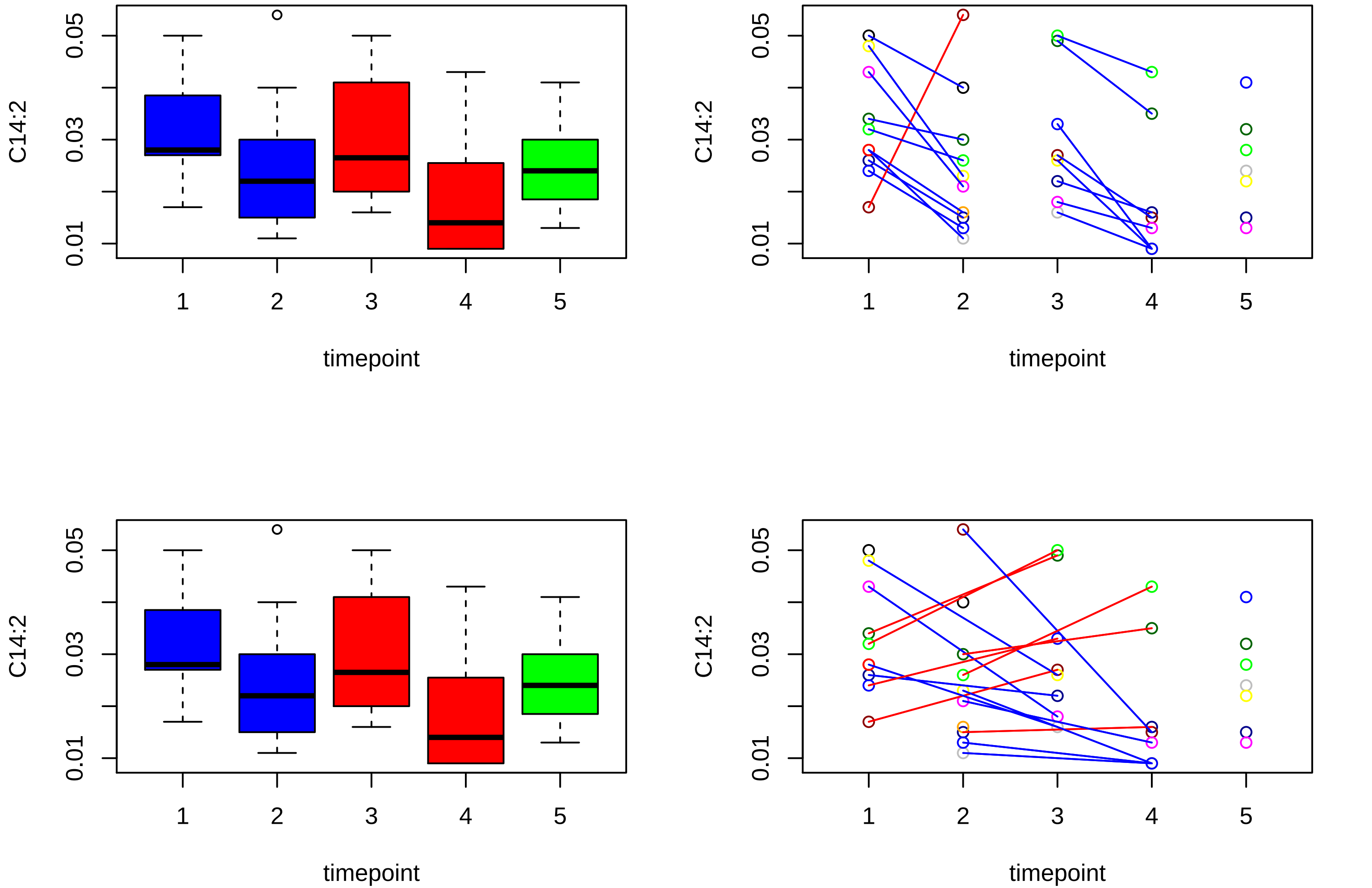

C14:2
0.01
0.03
0.05
1
2
3
4
5
timepoint

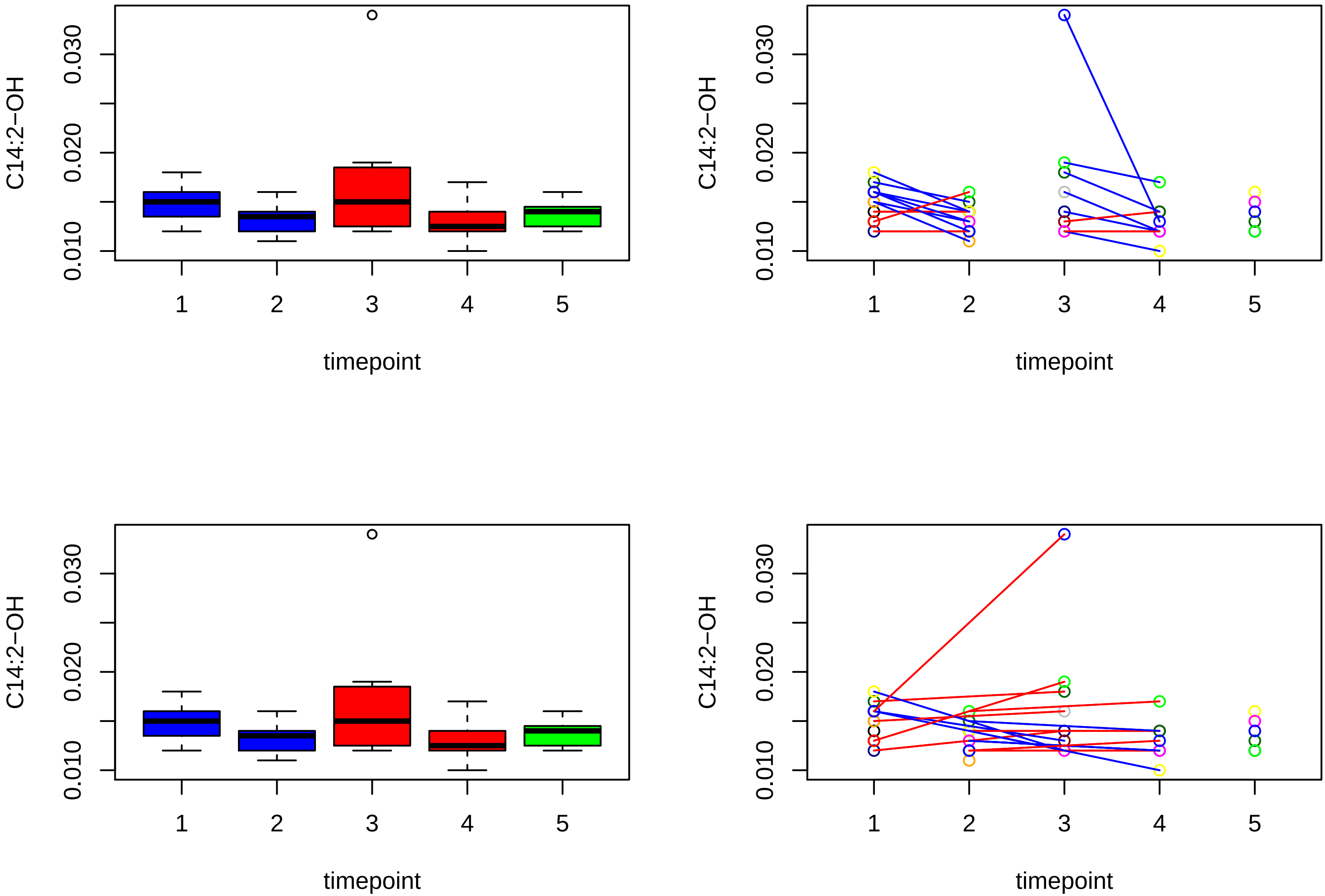

C14:2−OH
0.010
0.020
0.030
1
2
3
4
5
timepoint

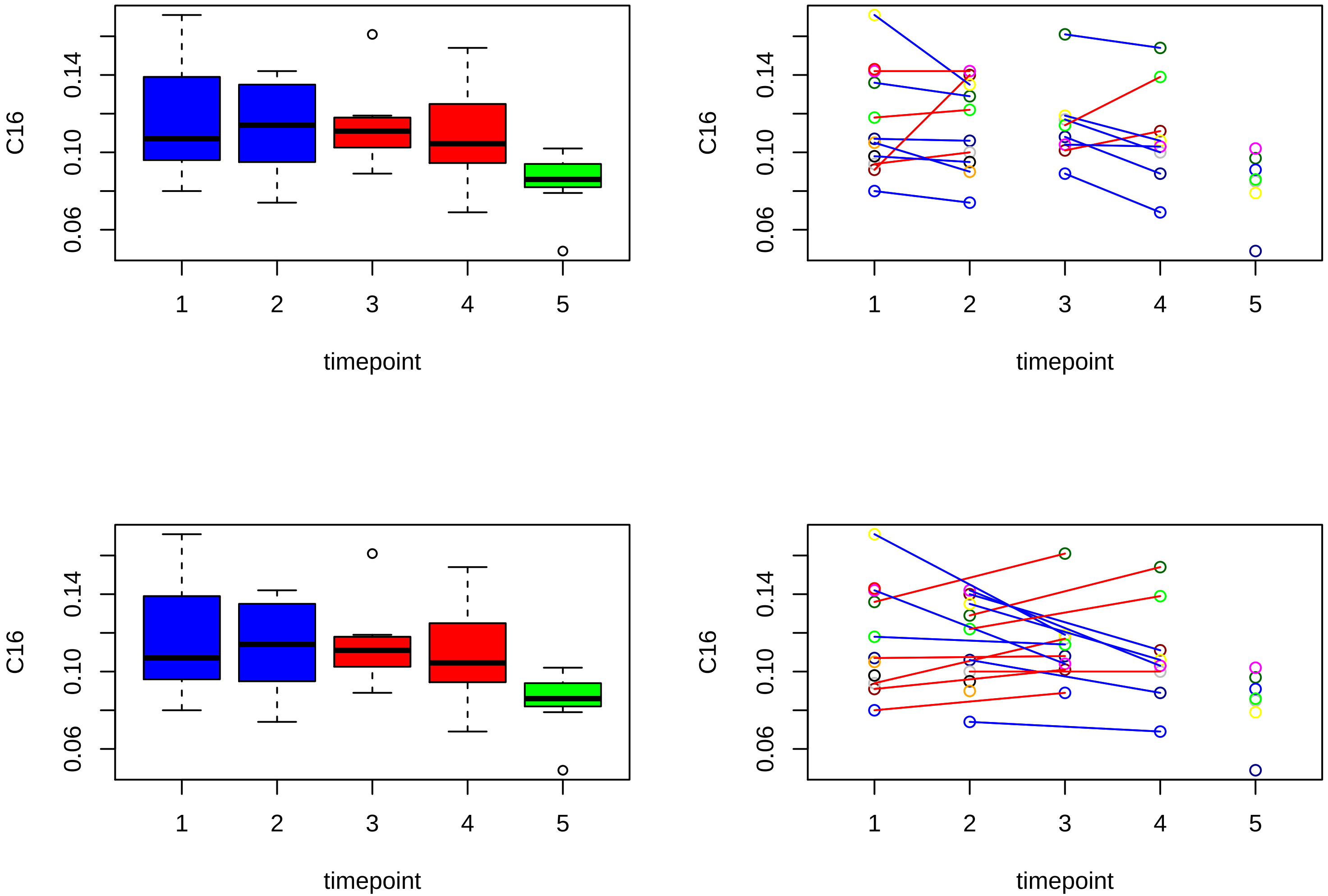
C16
0.06
0.10
0.14
1
2
3
4
5
timepoint
C16
0.06
0.10
0.14
1
2
3
4
5
timepoint
C16
0.06
0.10
0.14
1
2
3
4
5
timepoint
C16
0.06
0.10
0.14
1
2
3
4
5
timepoint

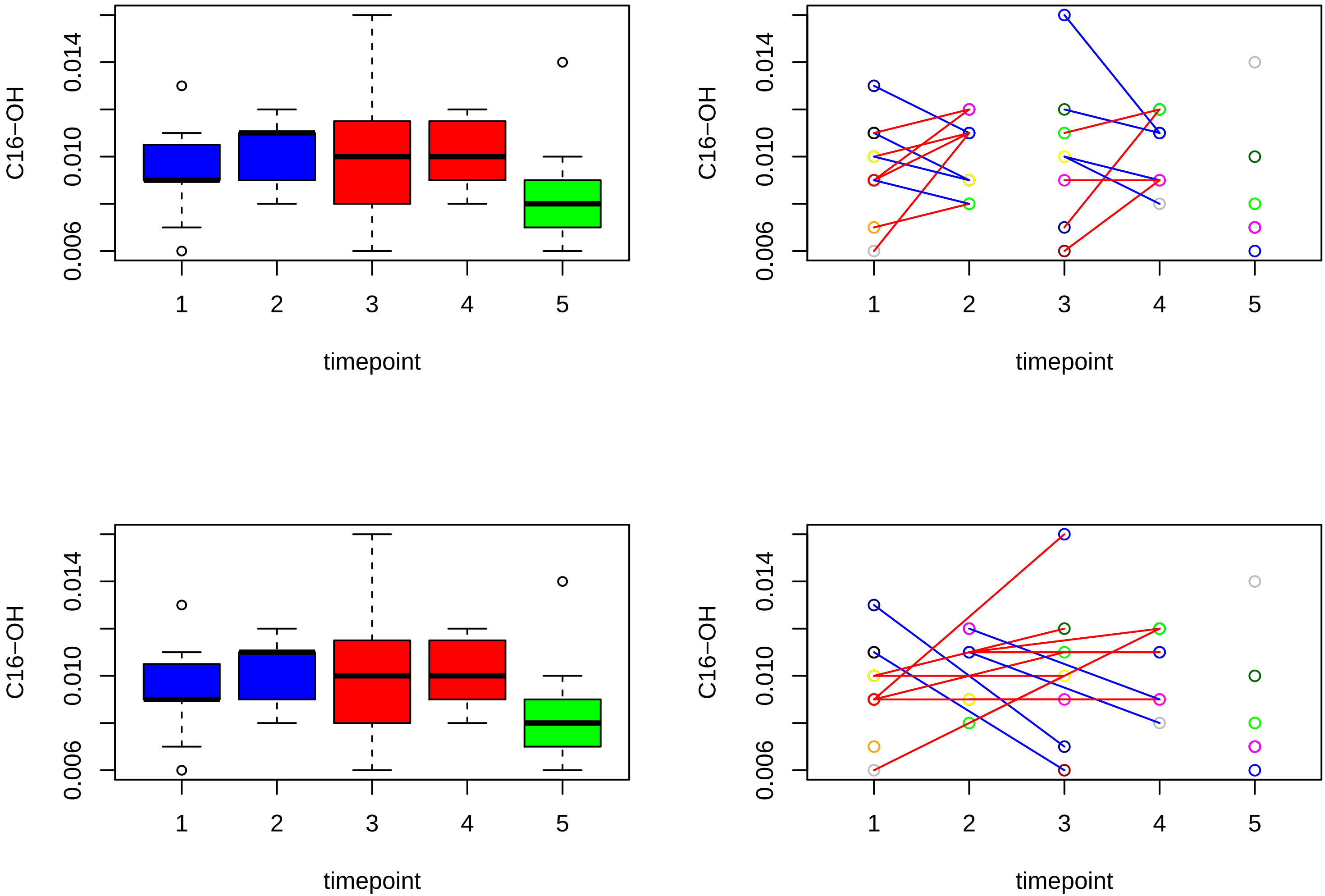

C16−OH
0.006
0.010
0.014
1
2
3
4
5
timepoint

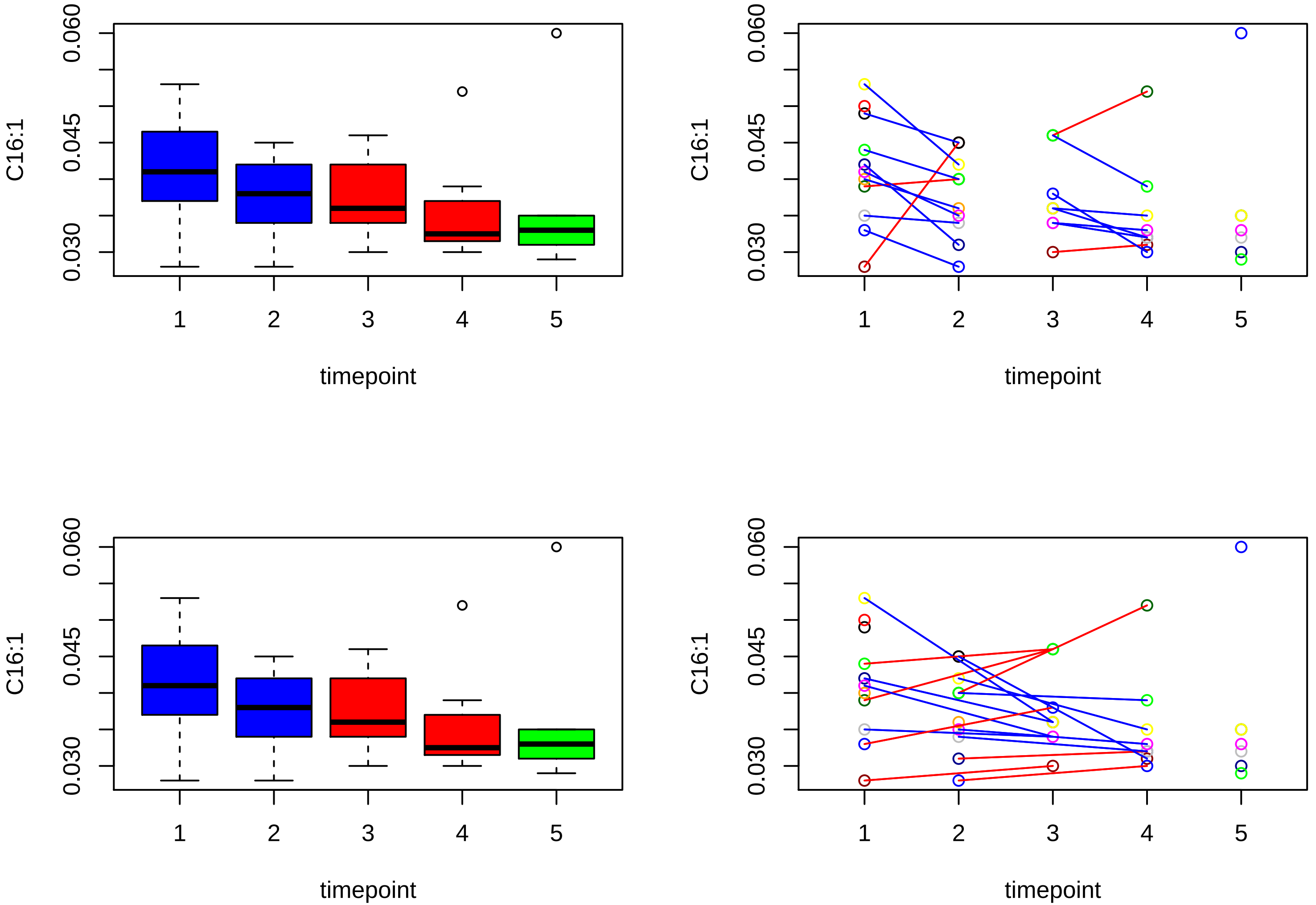
C16:1
0.030
0.045
0.060
1
2
3
4
5
timepoint

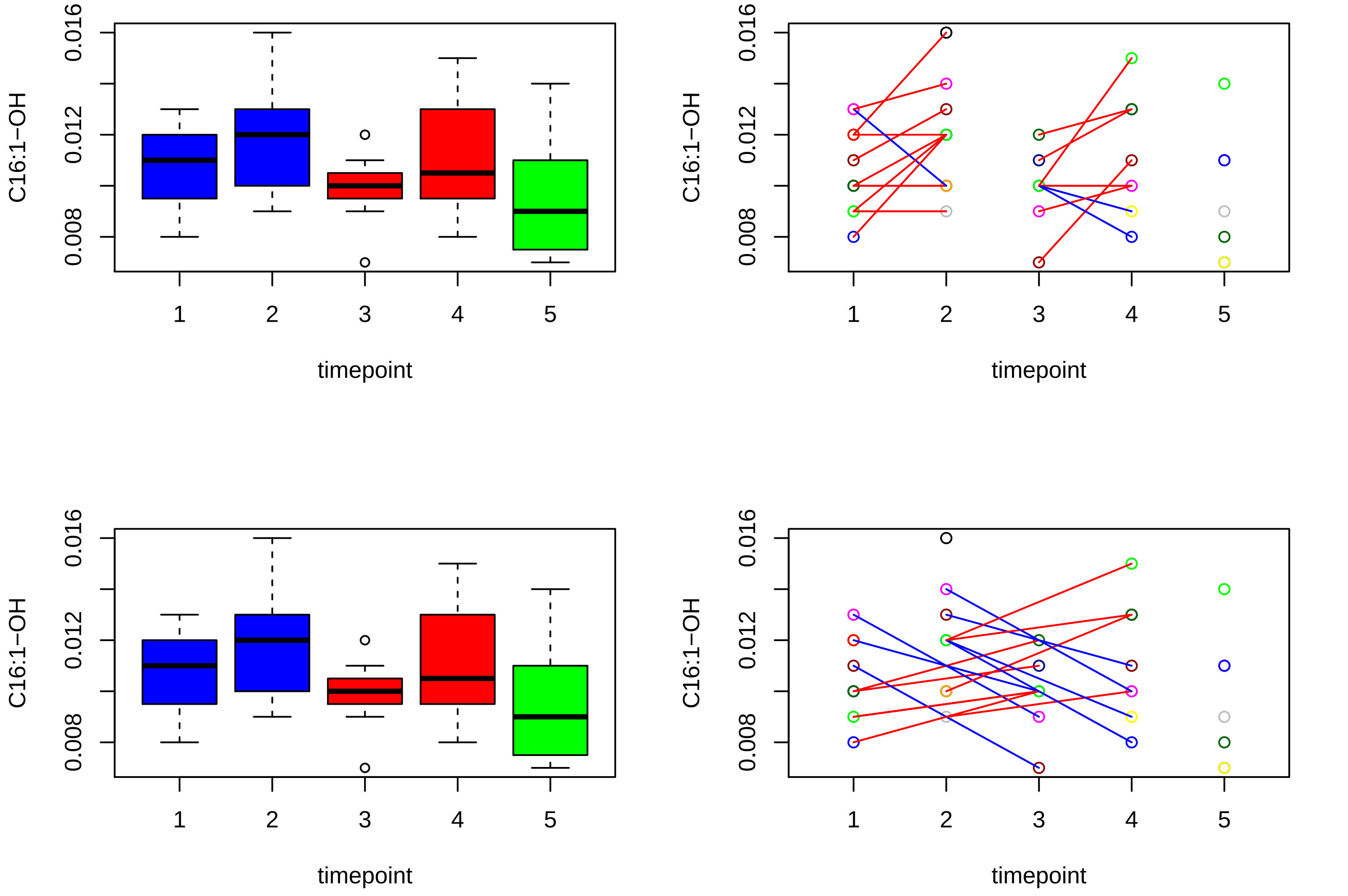
C16:1−OH
0.008
0.012
0.016
1
2
3
4
5
timepoint

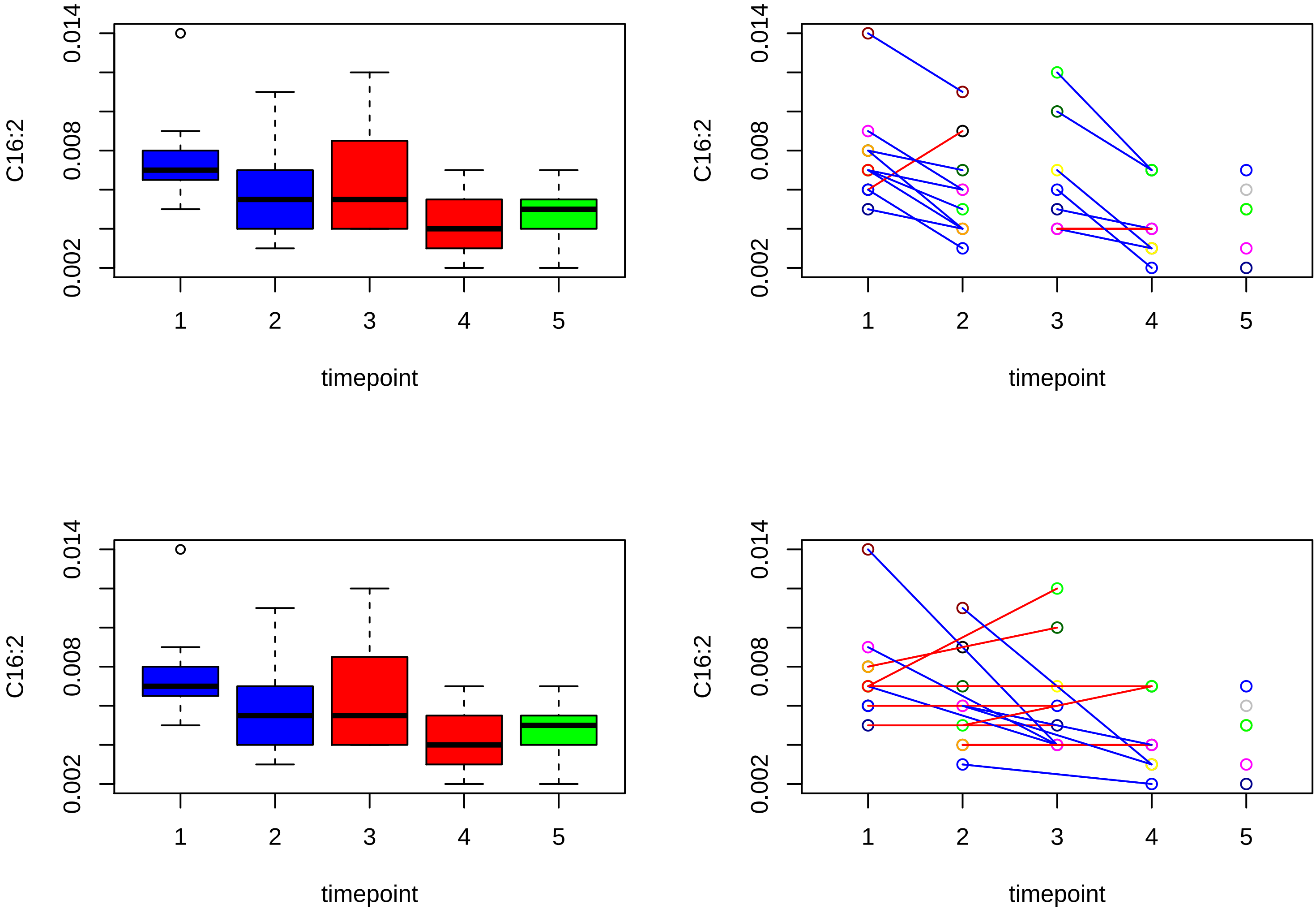
C16:2
0.002
0.008
0.014
1
2
3
4
5
timepoint
C16:2
0.002
0.008
0.014
1
2
3
4
5
timepoint
C16:2
0.002
0.008
0.014
1
2
3
4
5
timepoint
C16:2
0.002
0.008
0.014
1
2
3
4
5
timepoint

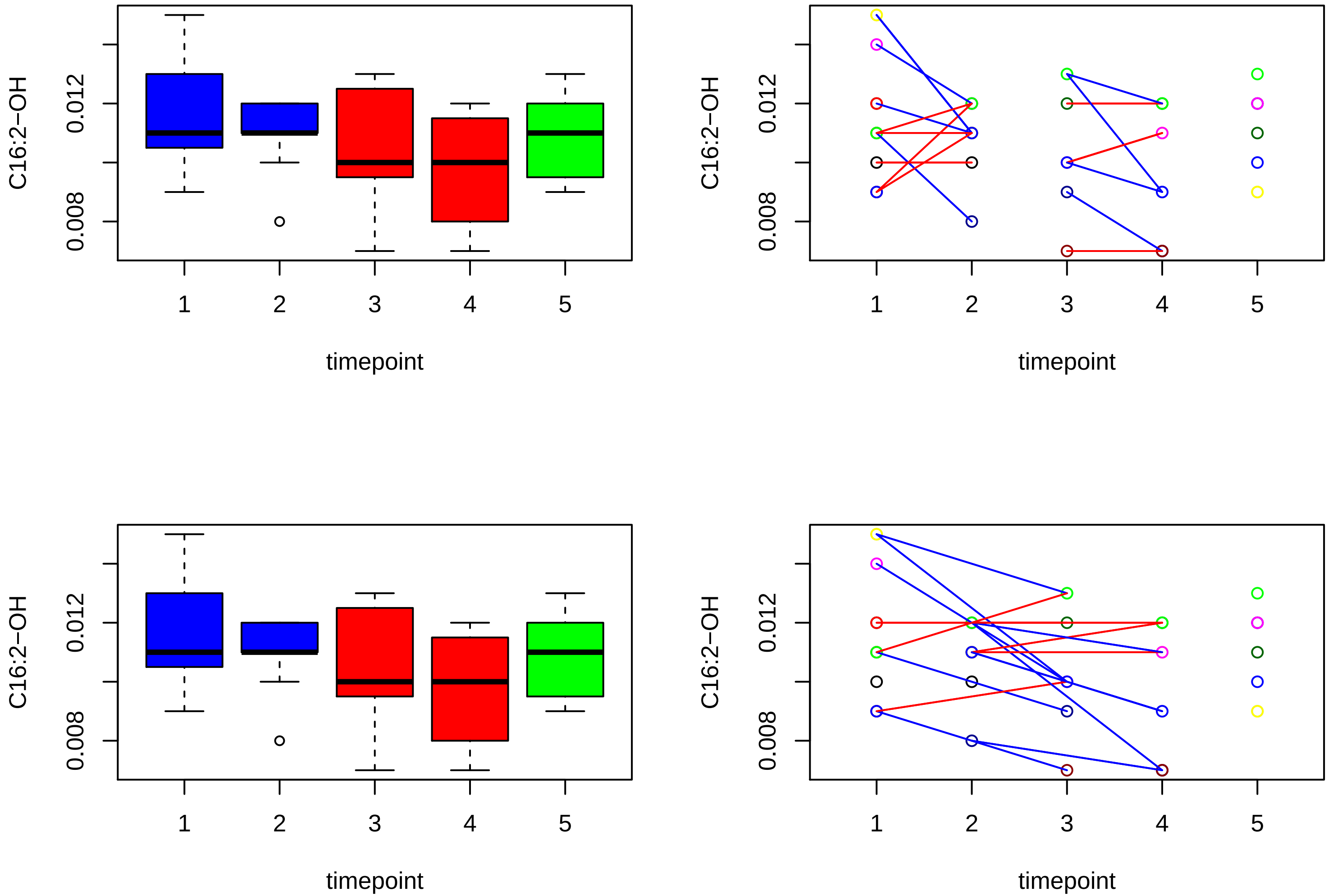

C16:2−OH
0.008
0.012
1
2
3
4
5
timepoint

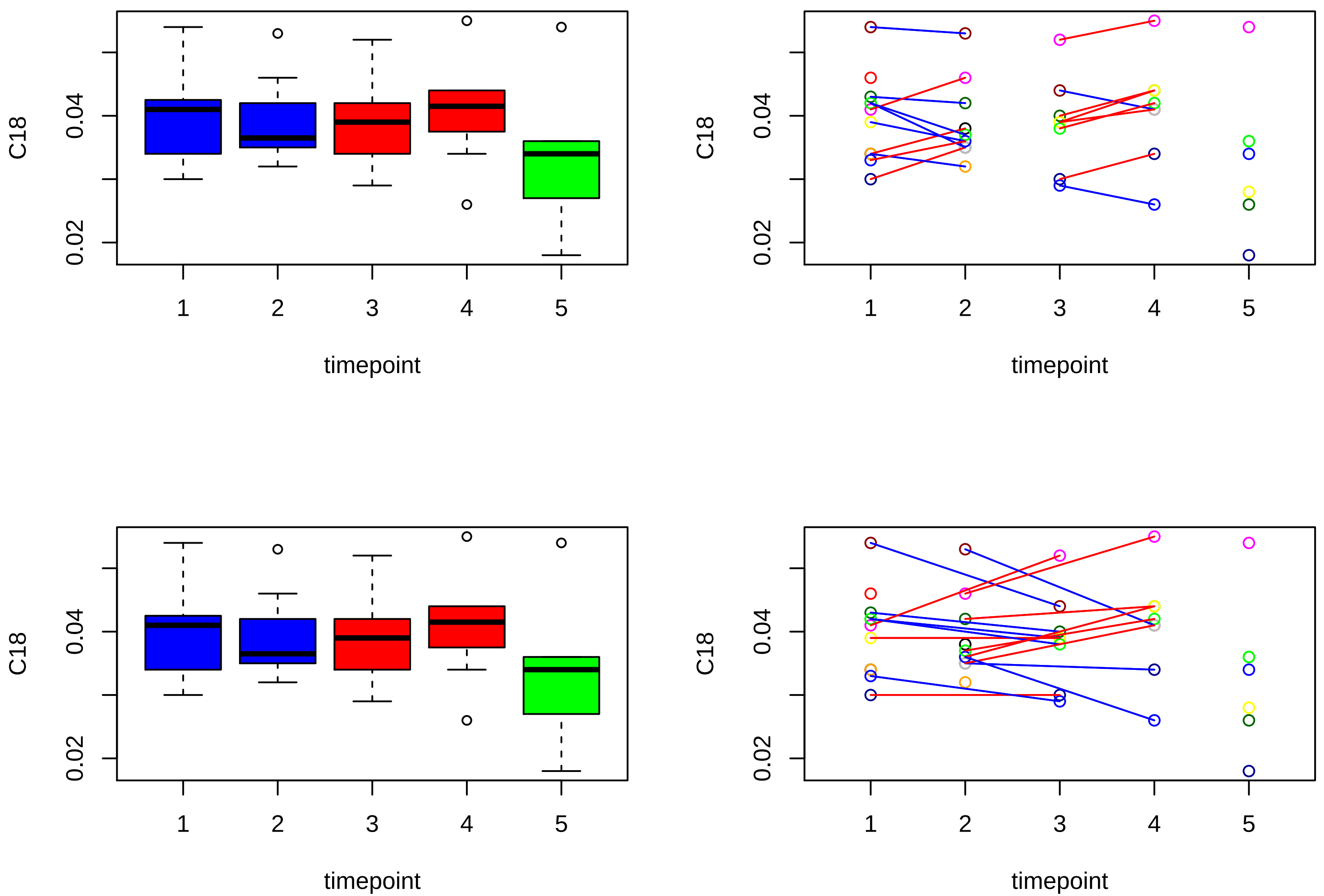
C18
0.02
0.04
1
2
3
4
5
timepoint
C18
0.02
0.04
1
2
3
4
5
timepoint
C18
0.02
0.04
1
2
3
4
5
timepoint
C18
0.02
0.04
1
2
3
4
5
timepoint

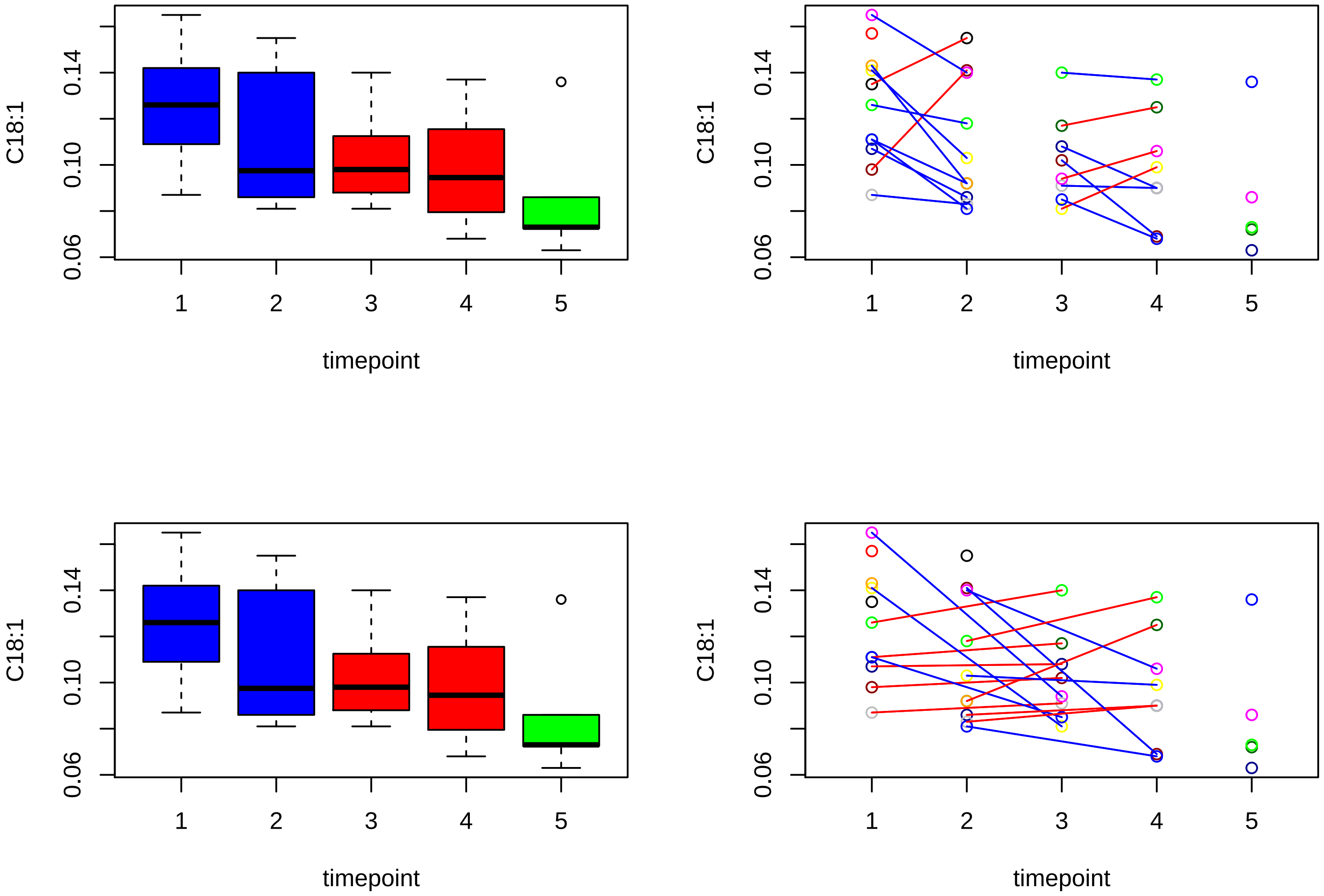
C18:1
0.06
0.10
0.14
1
2
3
4
5
timepoint

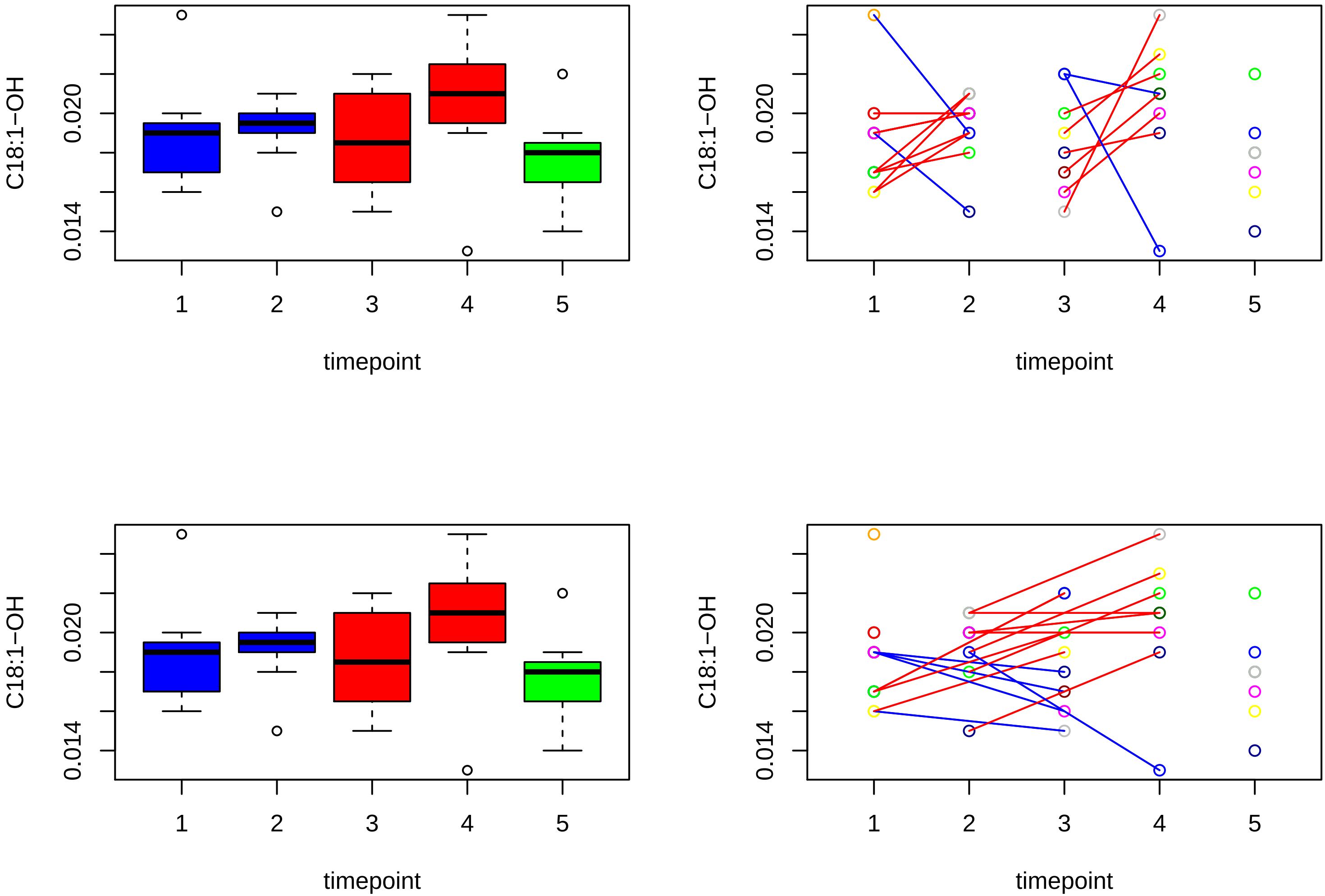
C18:1−OH
0.014
0.020
1
2
3
4
5
timepoint
C18:1−OH
0.014
0.020
1
2
3
4
5
timepoint
C18:1−OH
0.014
0.020
1
2
3
4
5
timepoint
C18:1−OH
0.014
0.020
1
2
3
4
5
timepoint

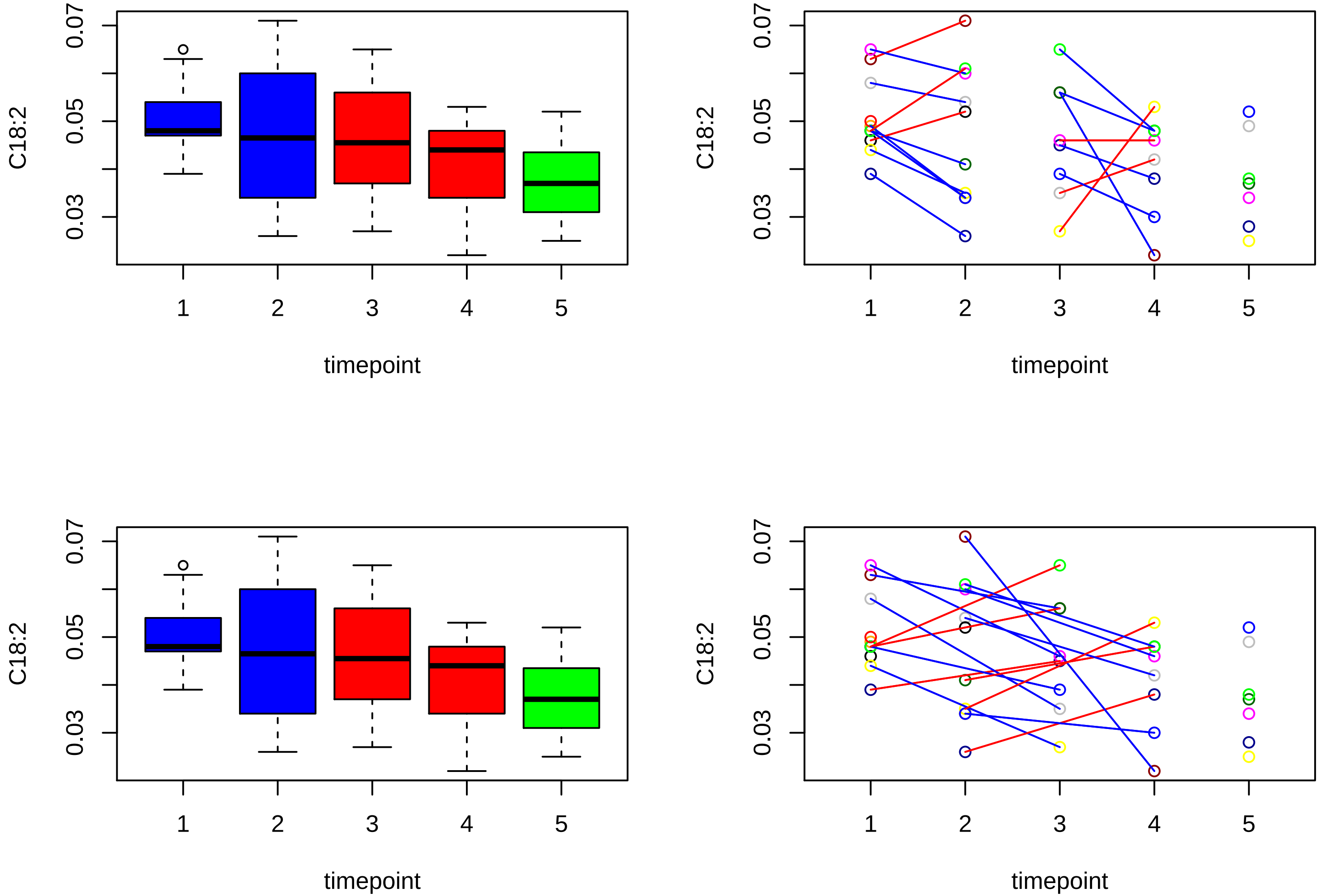

C18:2
0.03
0.05
0.07
1
2
3
4
5
timepoint

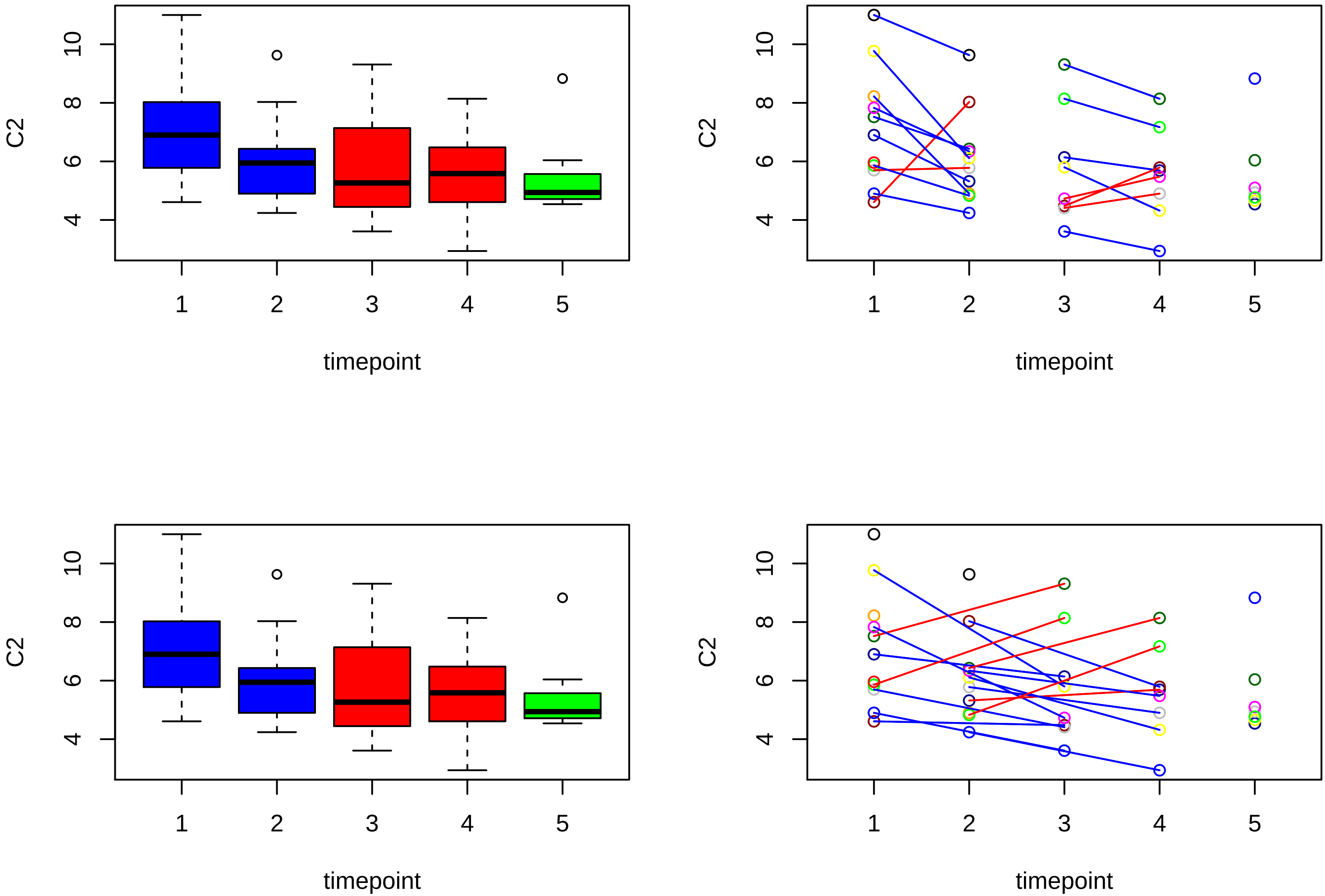
C2
10
8
6
4
1
2
3
4
5
timepoint

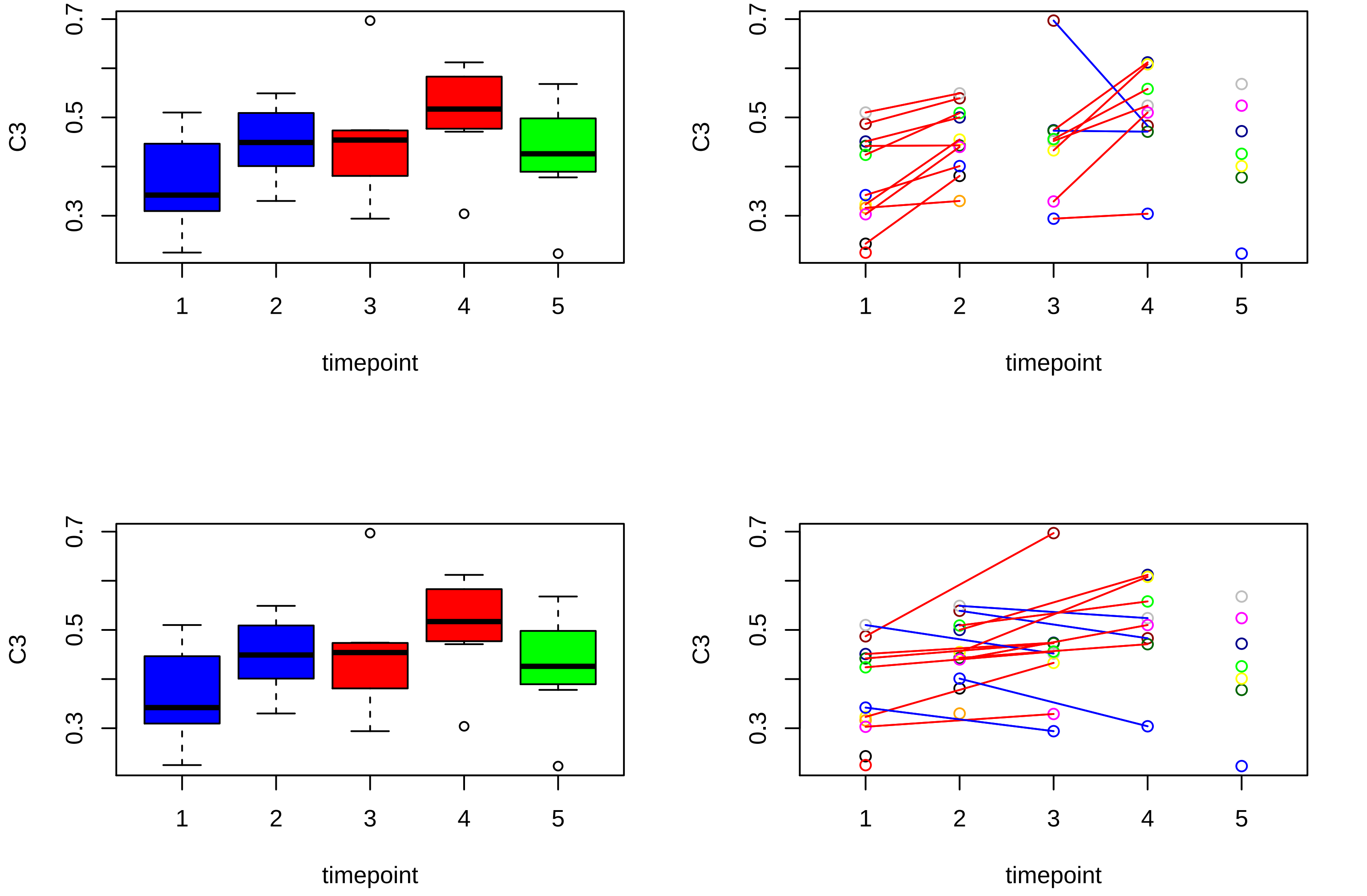
C3
0.3
0.5
0.7
1
2
3
4
5
timepoint
C3
0.3
0.5
0.7
1
2
3
4
5
timepoint
C3
0.3
0.5
0.7
1
2
3
4
5
timepoint
C3
0.3
0.5
0.7
1
2
3
4
5
timepoint

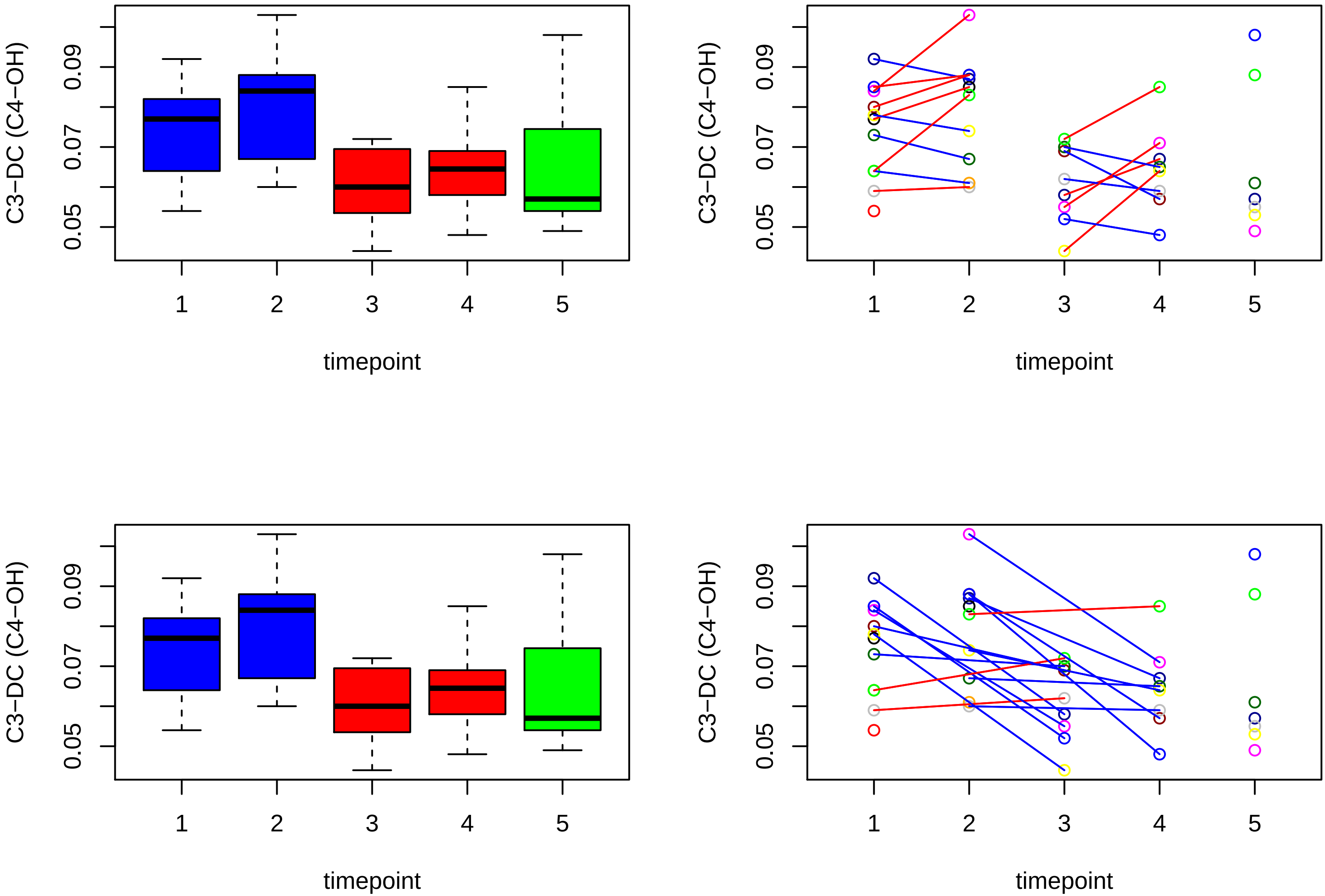
C3−DC (C4−OH)
0.05
0.07
0.09
1
2
3
4
5
timepoint

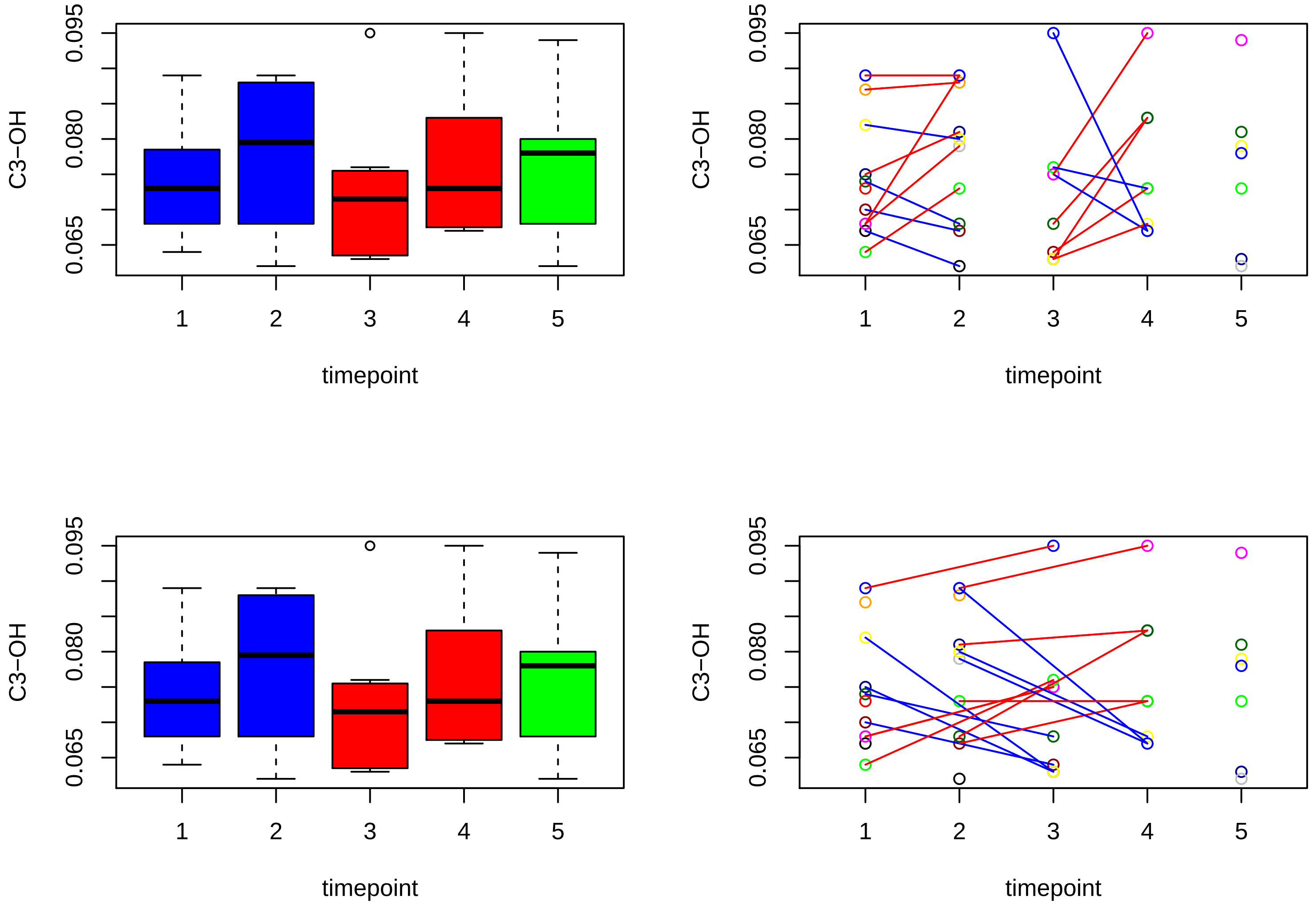
C3−OH
0.065
0.080
0.095
1
2
3
4
5
timepoint

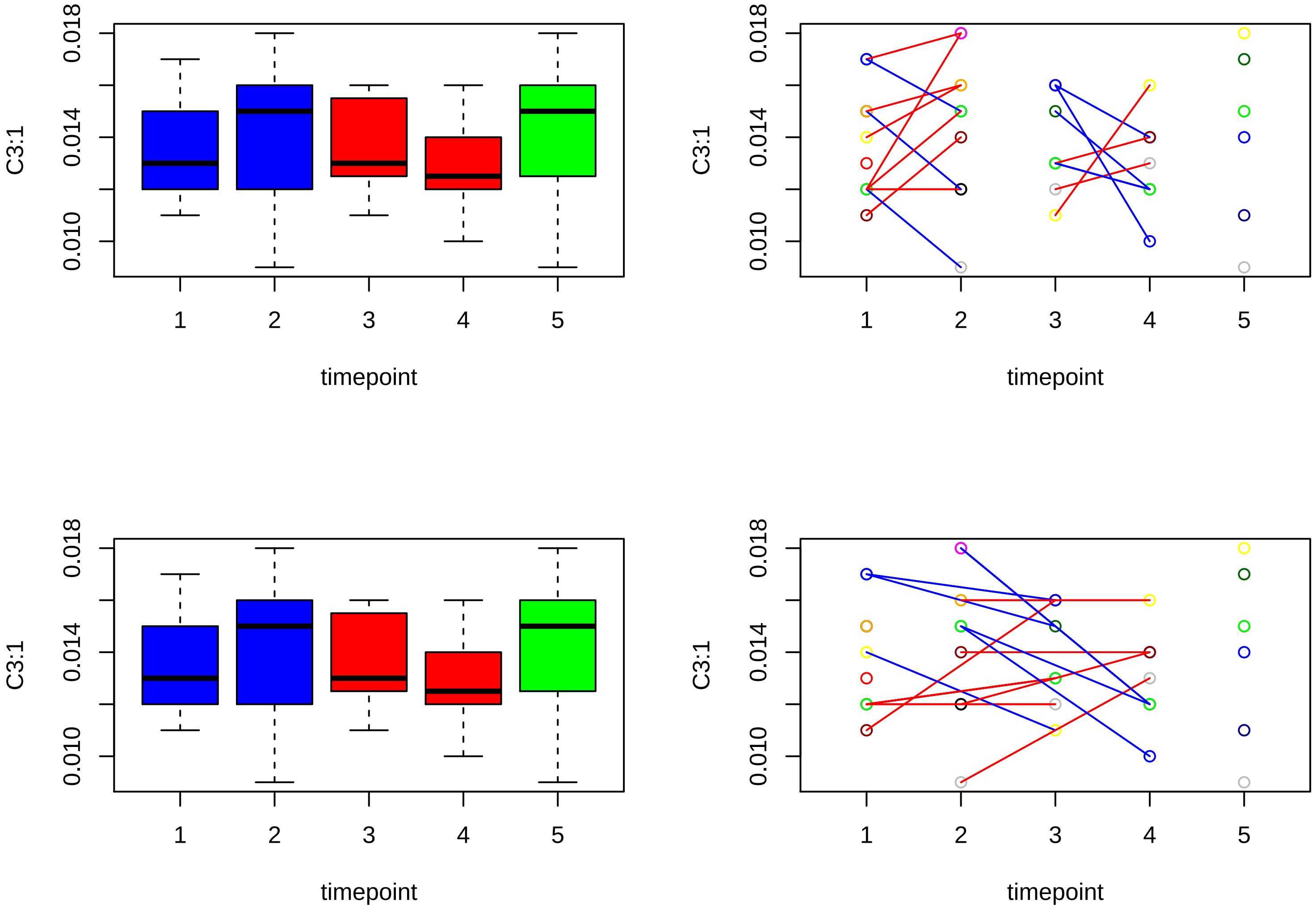
C3:1
0.010
0.014
0.018
1
2
3
4
5
timepoint

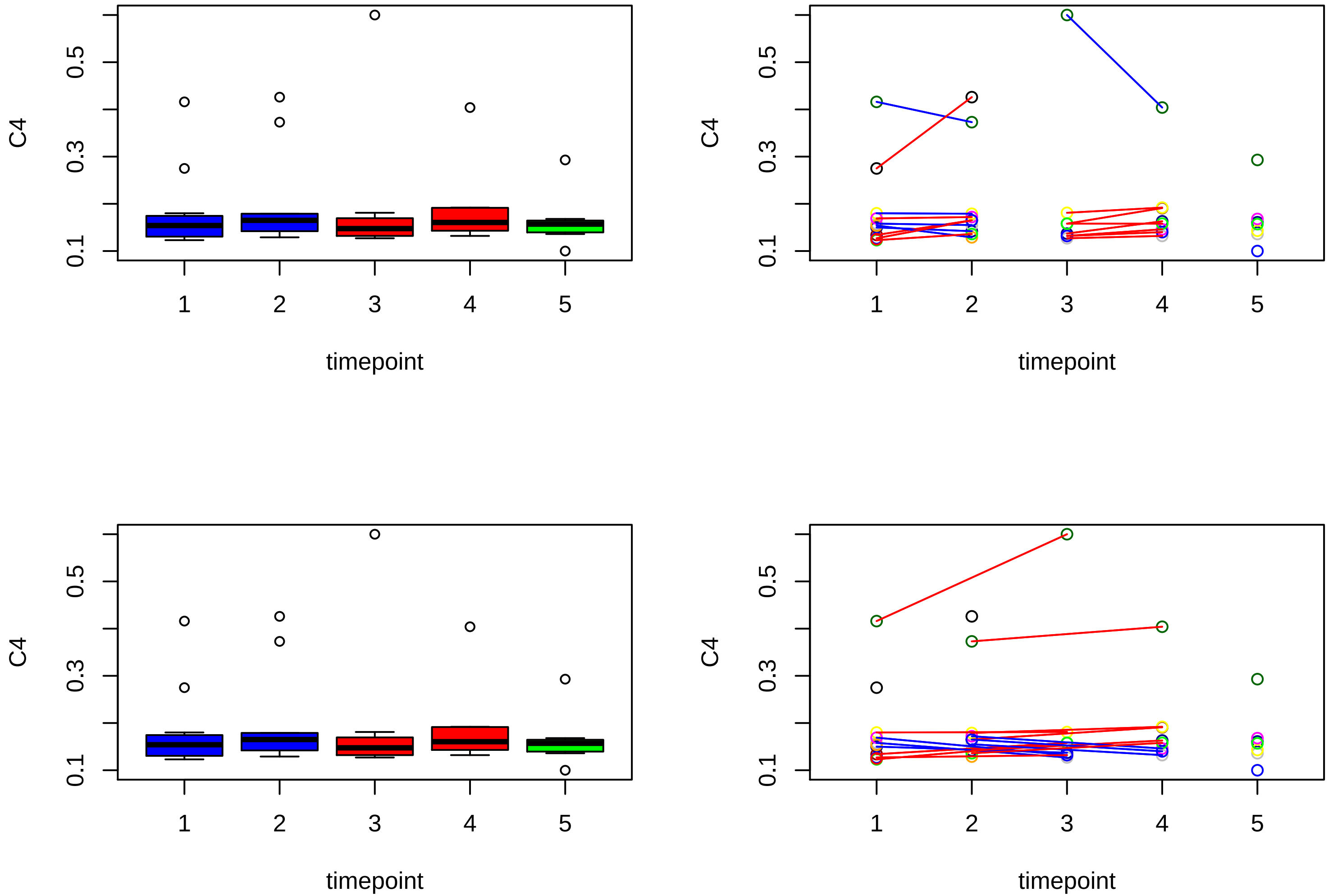
C4
0.1
0.3
0.5
1
2
3
4
5
timepoint

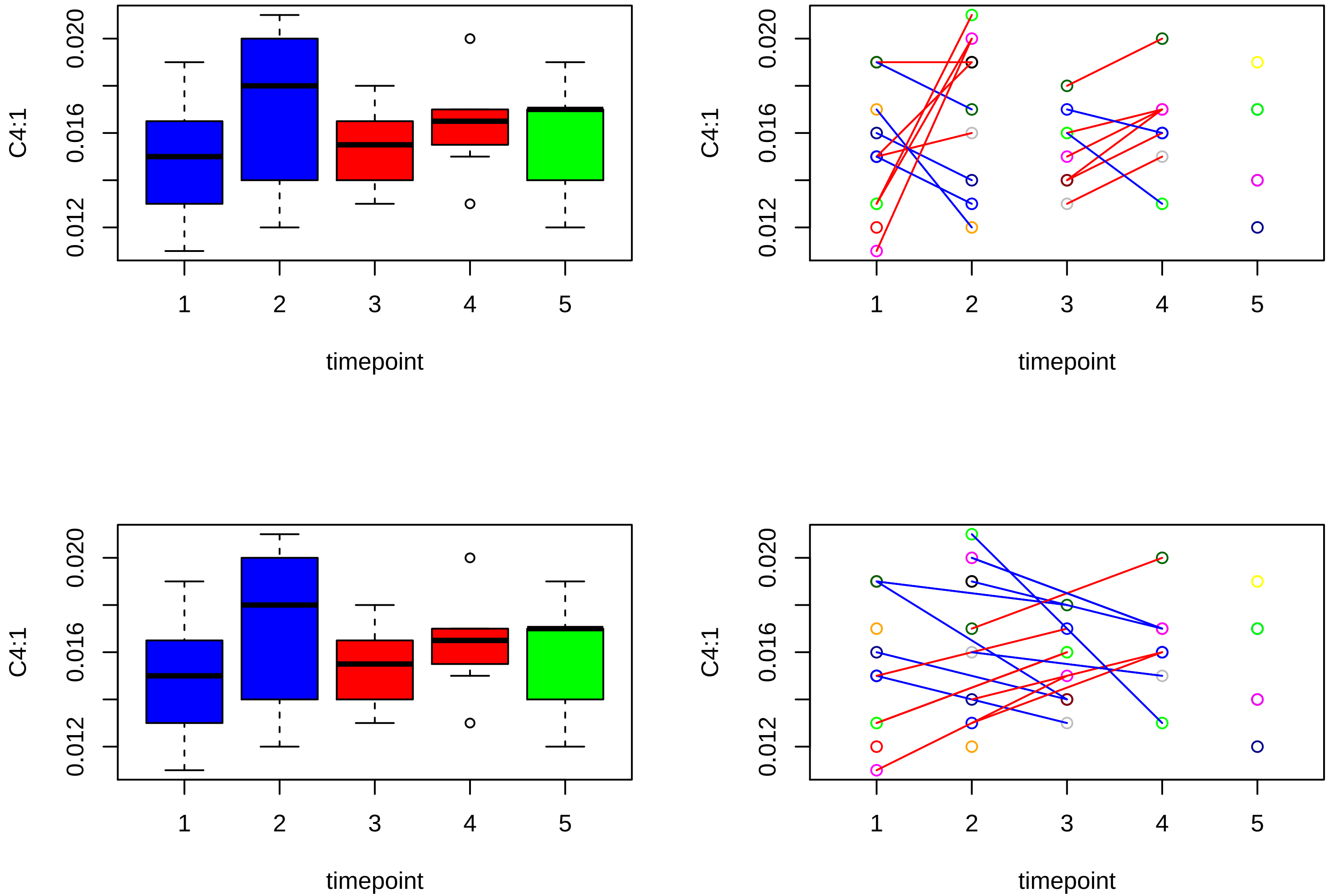
C4:1
0.012
0.016
0.020
1
2
3
4
5
timepoint
C4:1
0.012
0.016
0.020
1
2
3
4
5
timepoint
C4:1
0.012
0.016
0.020
1
2
3
4
5
timepoint
C4:1
0.012
0.016
0.020
1
2
3
4
5
timepoint

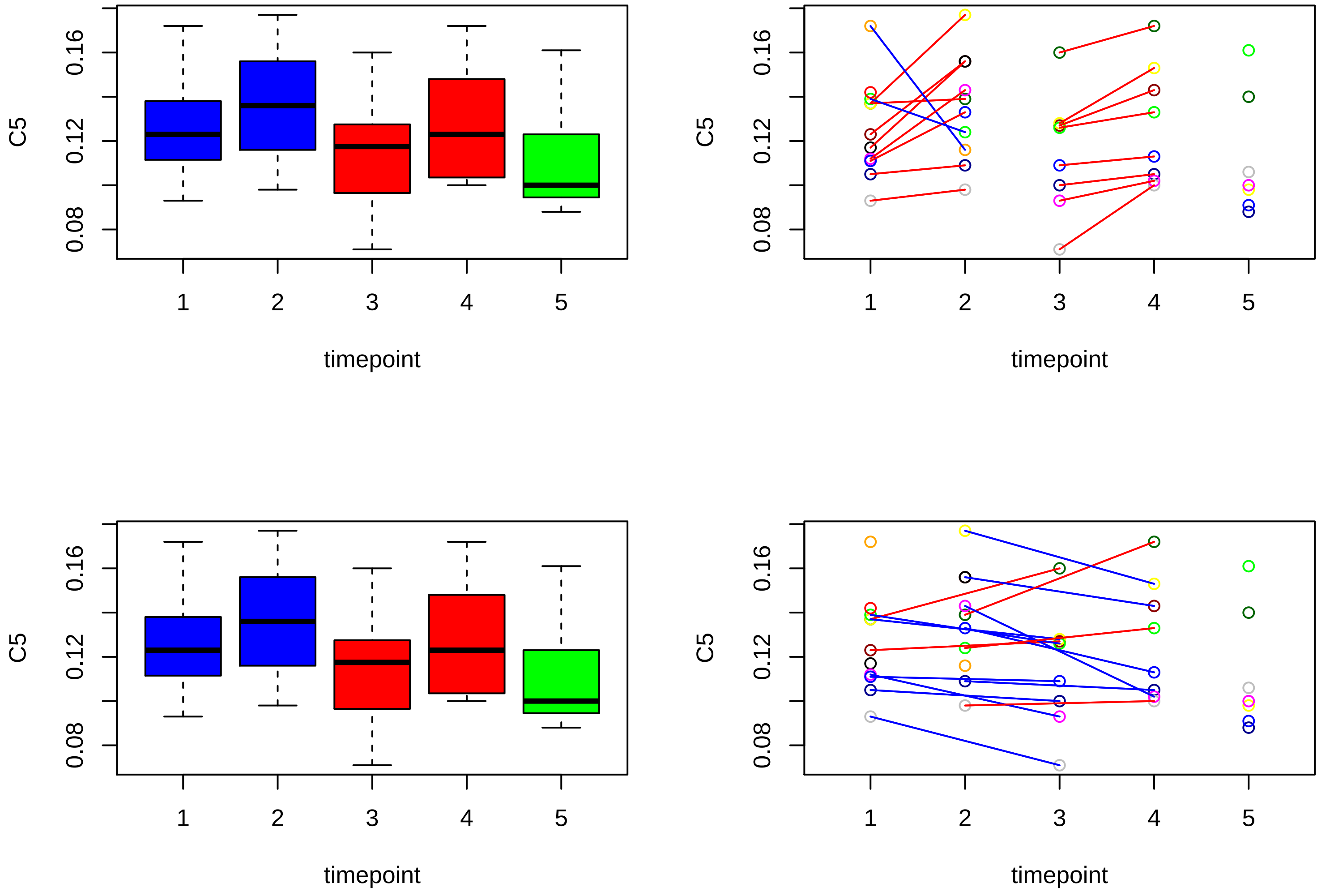

C5
0.08
0.12
0.16
1
2
3
4
5
timepoint

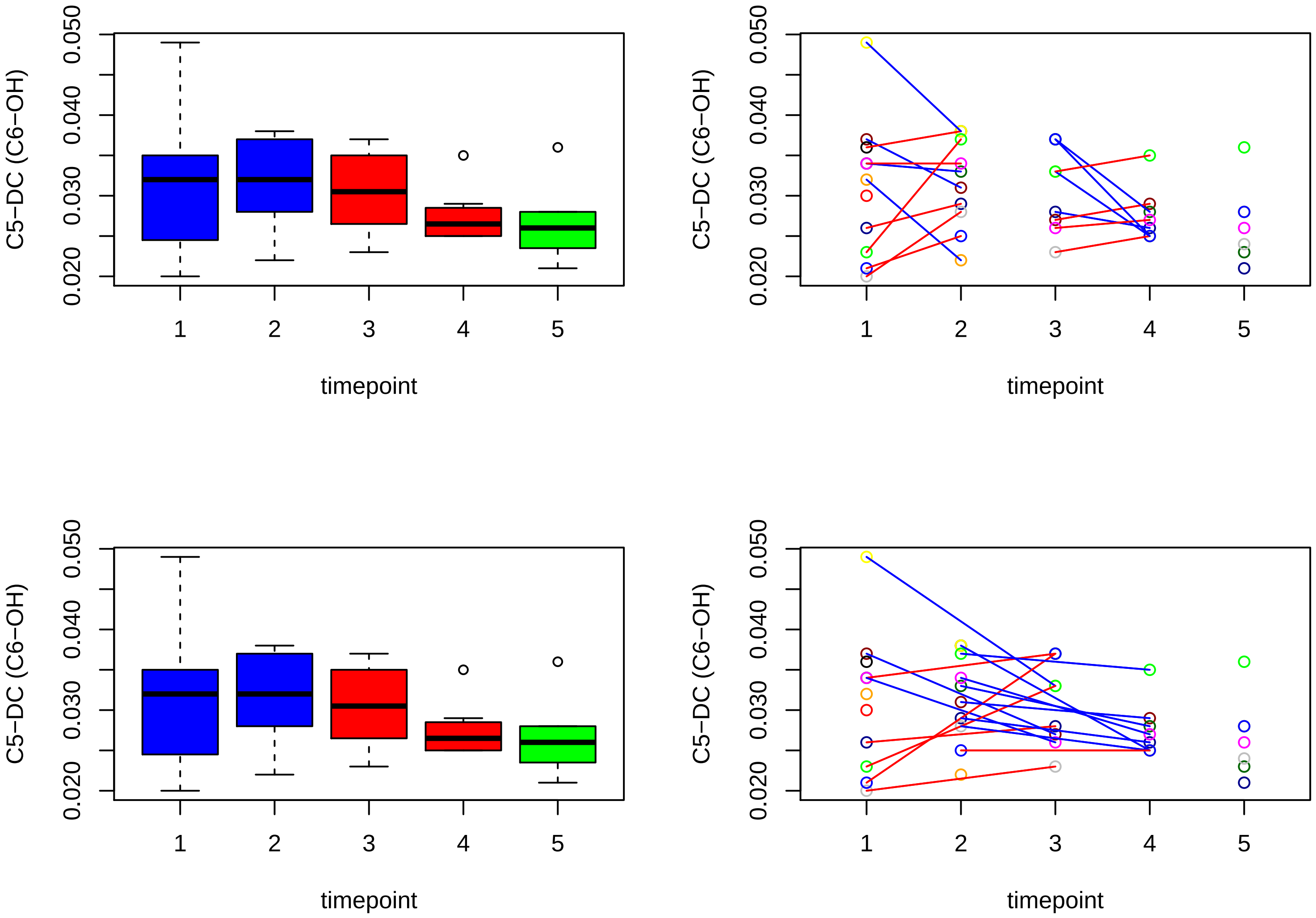
C5−DC (C6−OH)
0.020
0.030
0.040
0.050
1
2
3
4
5
timepoint

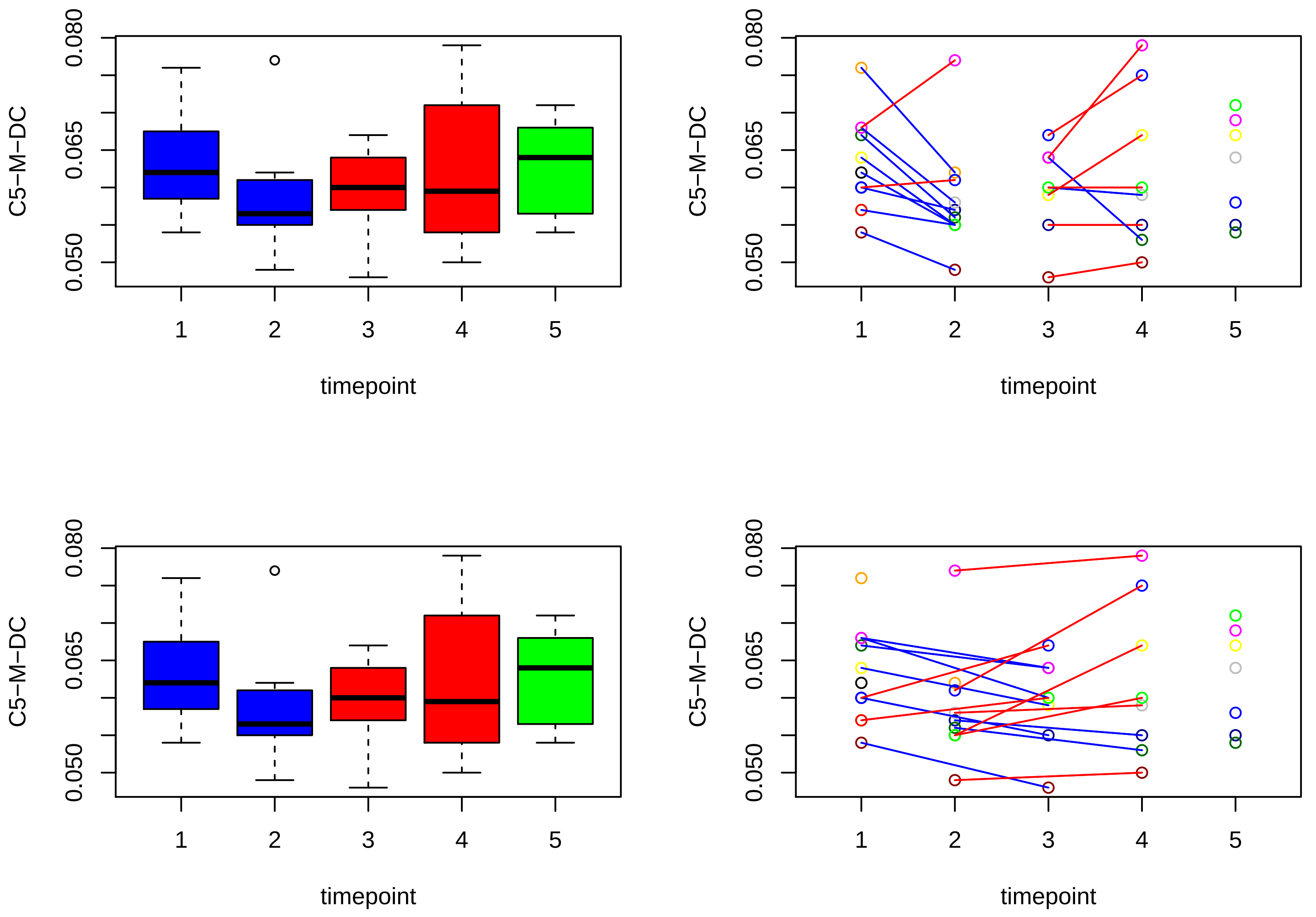

C5−M−DC
0.050
0.065
0.080
1
2
3
4
5
timepoint

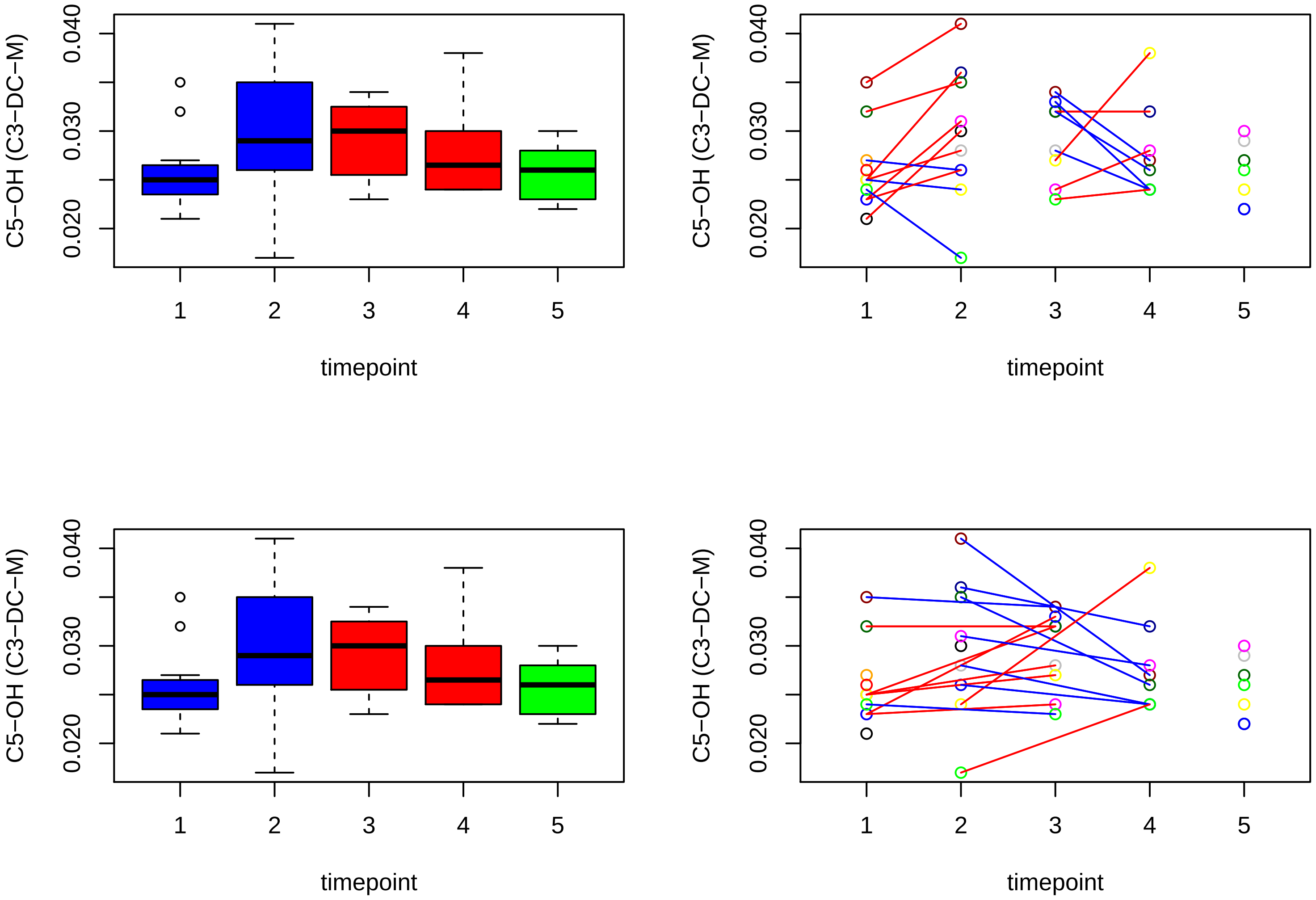
C5−OH (C3−DC−M)
0.020
0.030
0.040
1
2
3
4
5
timepoint

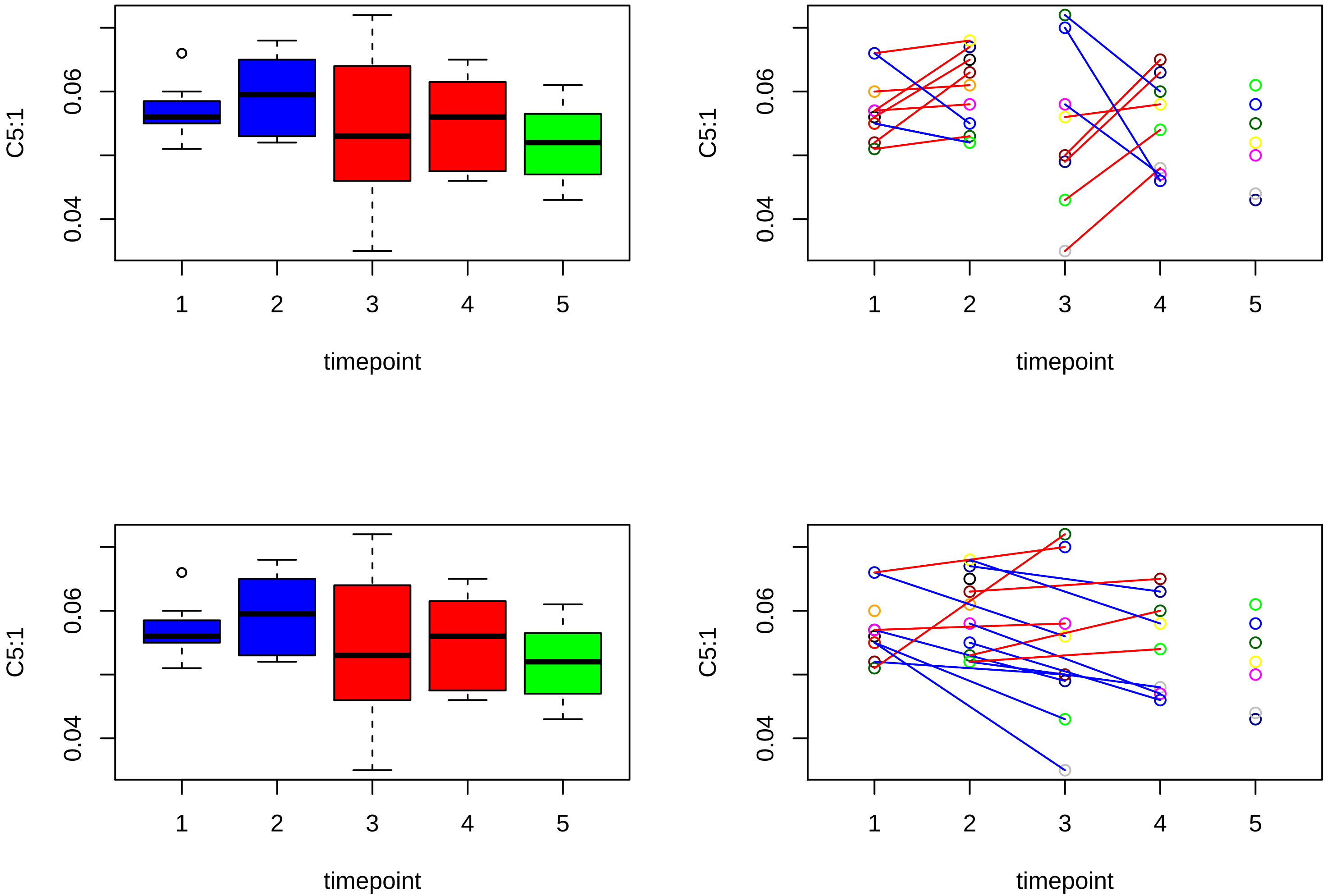
C5:1
0.04
0.06
1
2
3
4
5
timepoint

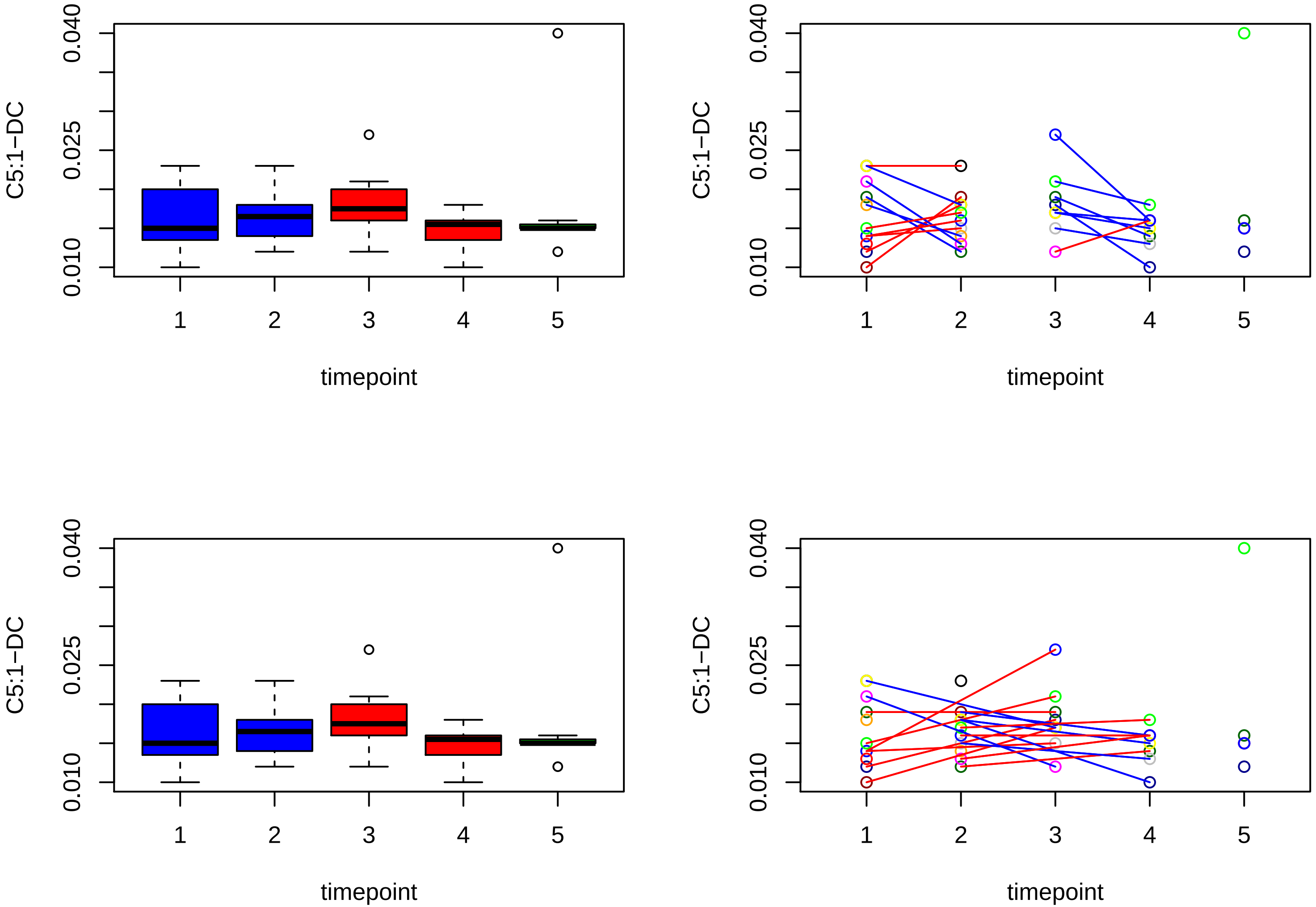

C5:1−DC
0.010
0.025
0.040
1
2
3
4
5
timepoint

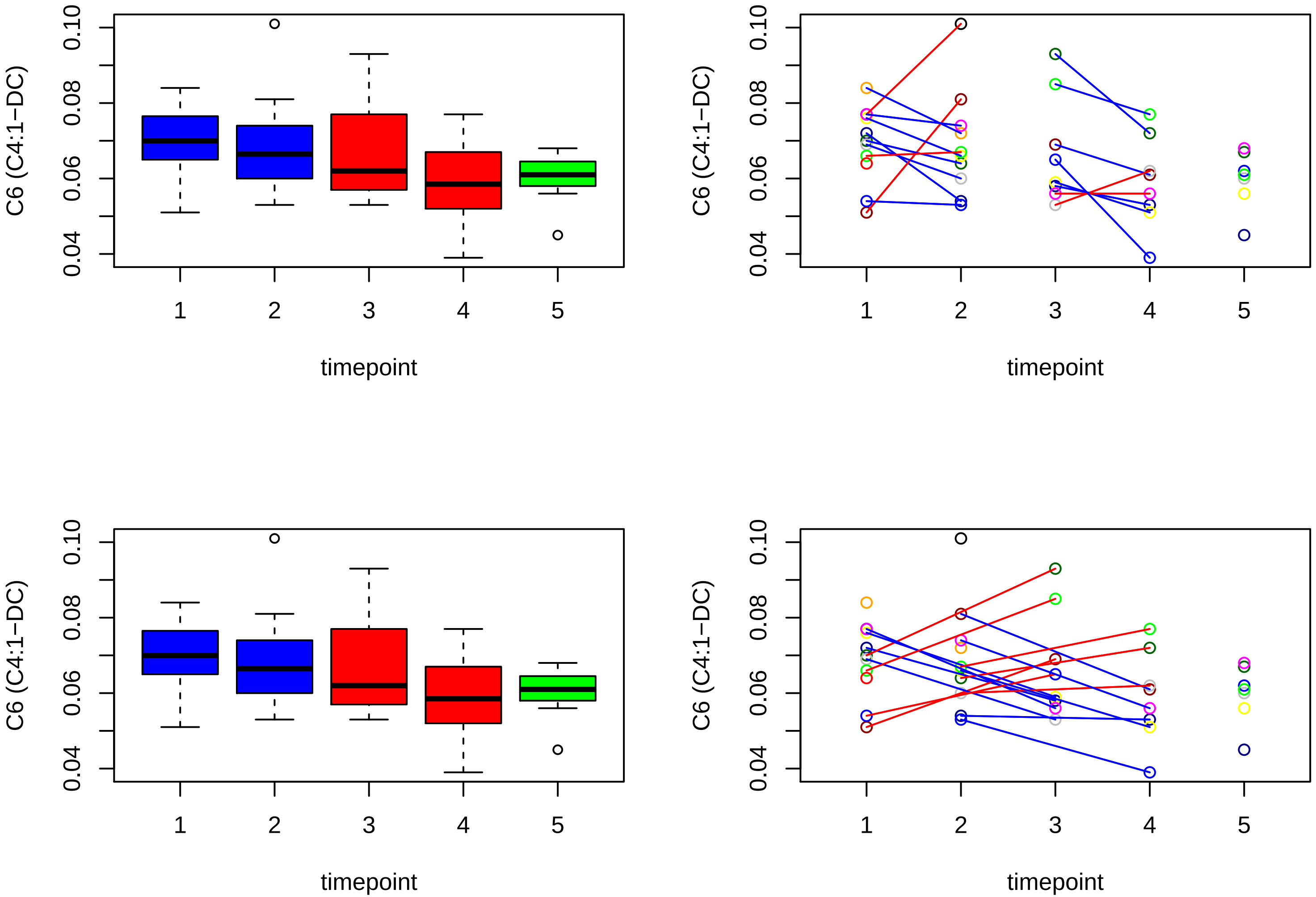
C6 (C4:1−DC)
0.04
0.06
0.08
0.10
1
2
3
4
5
timepoint

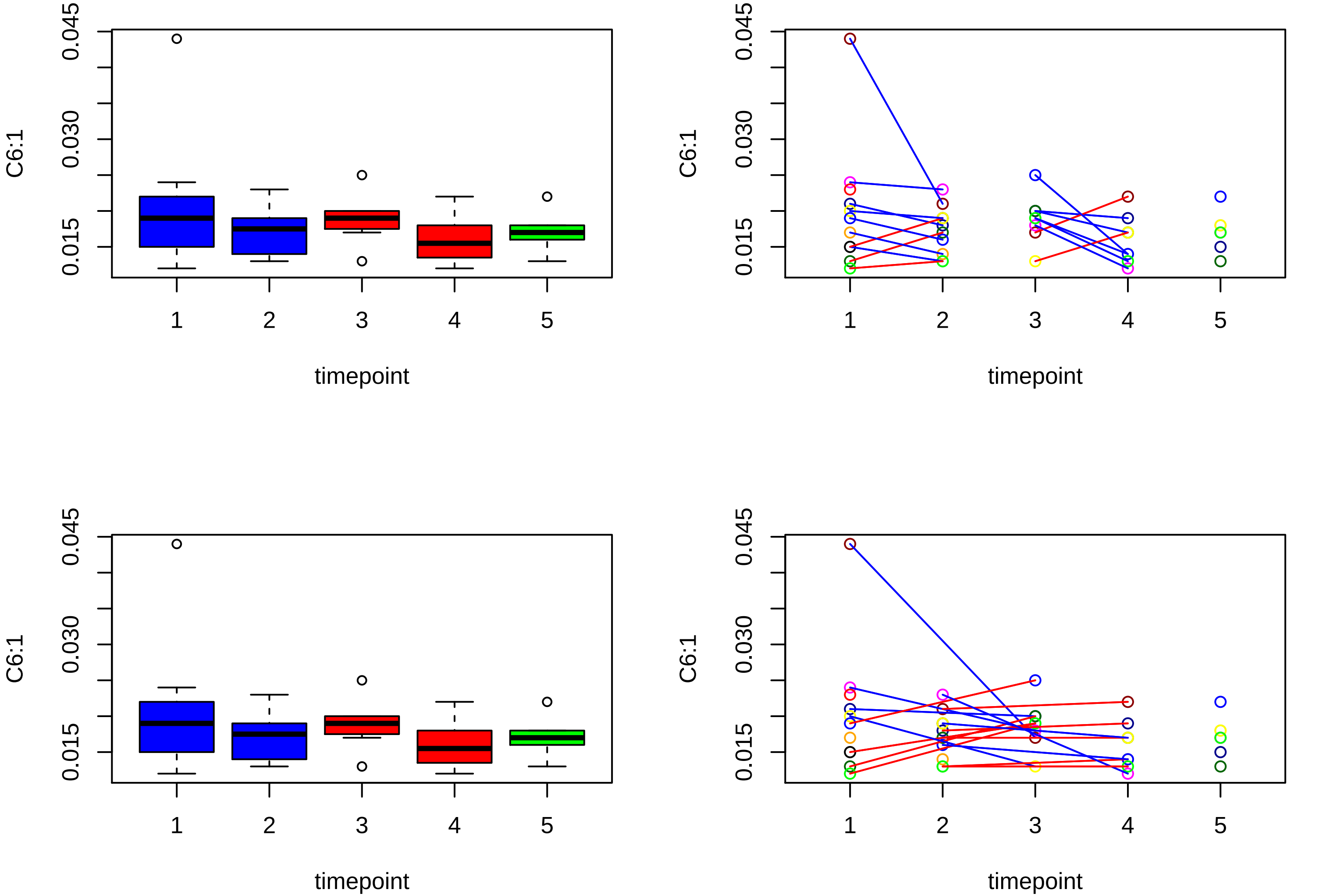
C6:1
0.015
0.030
0.045
1
2
3
4
5
timepoint
C6:1
0.015
0.030
0.045
1
2
3
4
5
timepoint
C6:1
0.015
0.030
0.045
1
2
3
4
5
timepoint
C6:1
0.015
0.030
0.045
1
2
3
4
5
timepoint

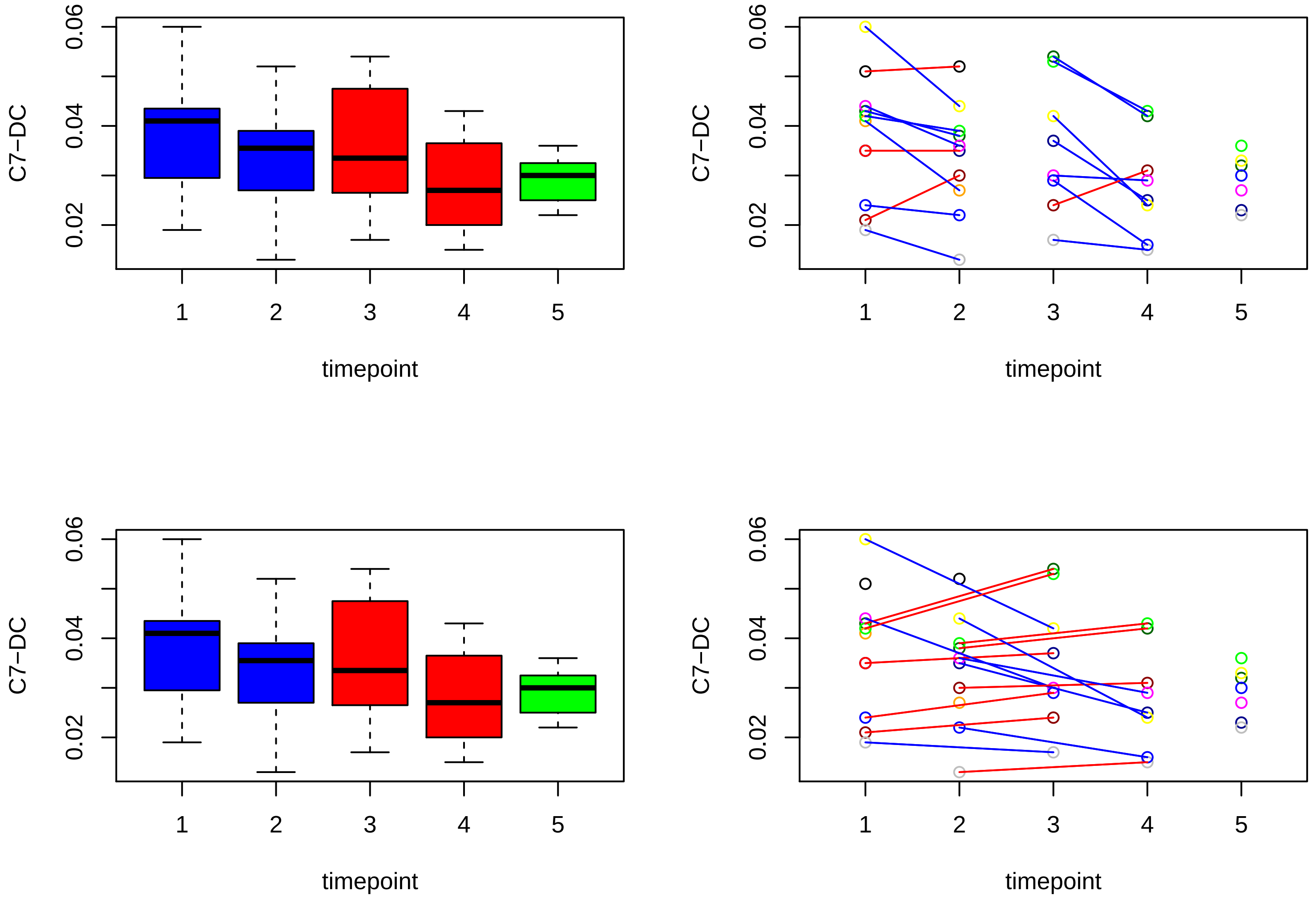

C7−DC
0.02
0.04
0.06
1
2
3
4
5
timepoint

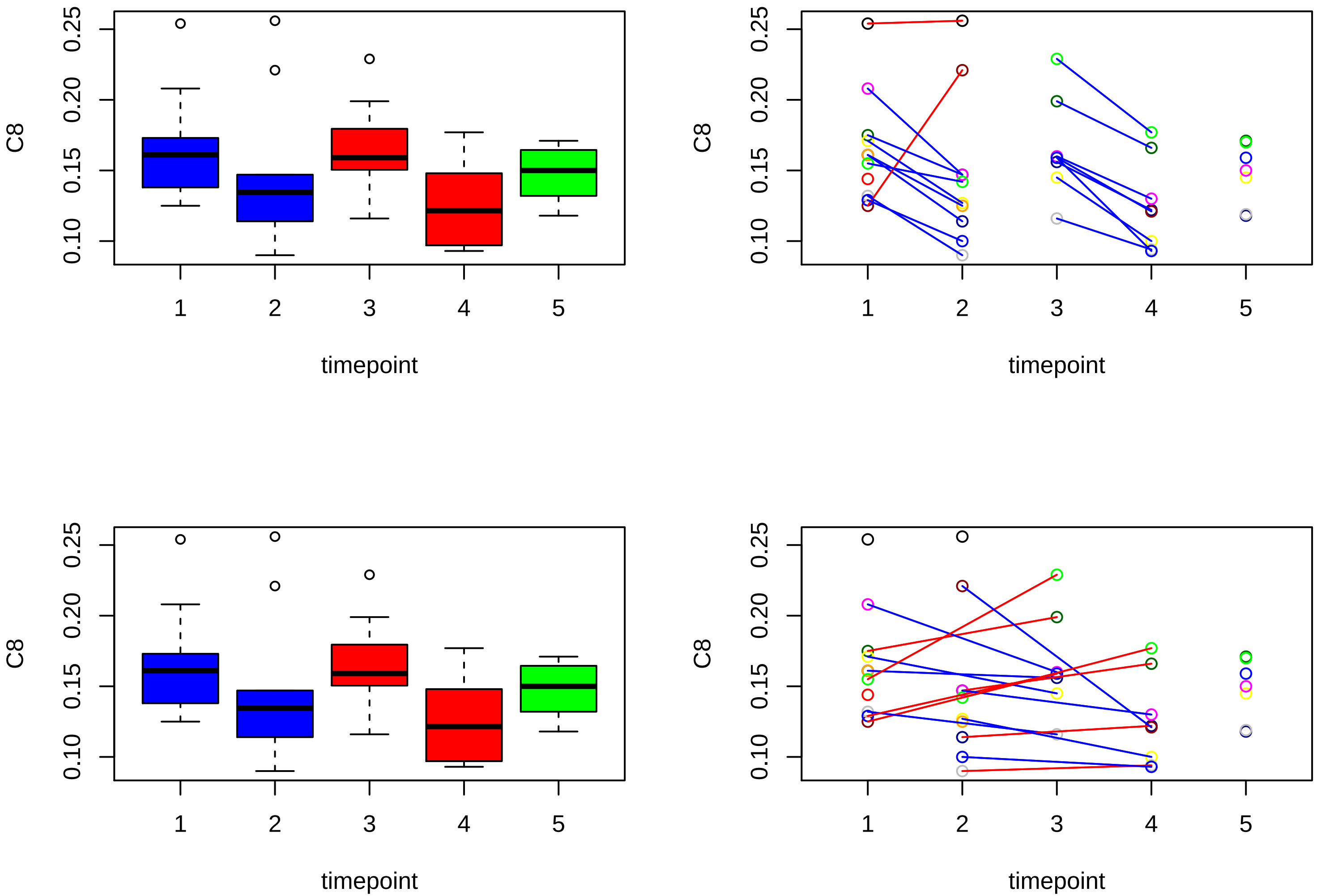
C8
0.10
0.15
0.20
0.25
1
2
3
4
5
timepoint

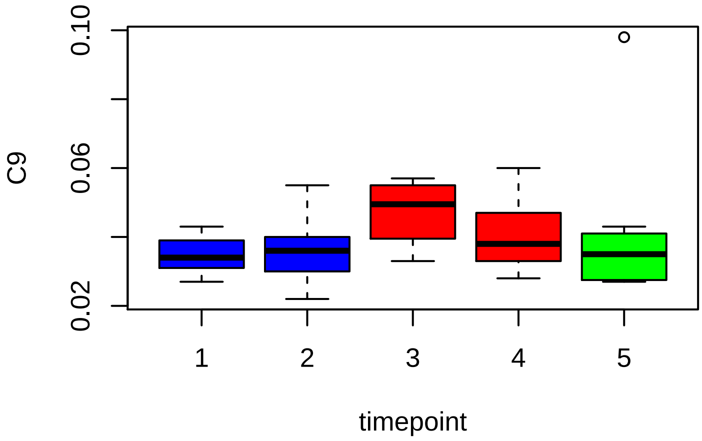

C9
0.02
0.06
0.10
1
2
3
4
5
timepoint

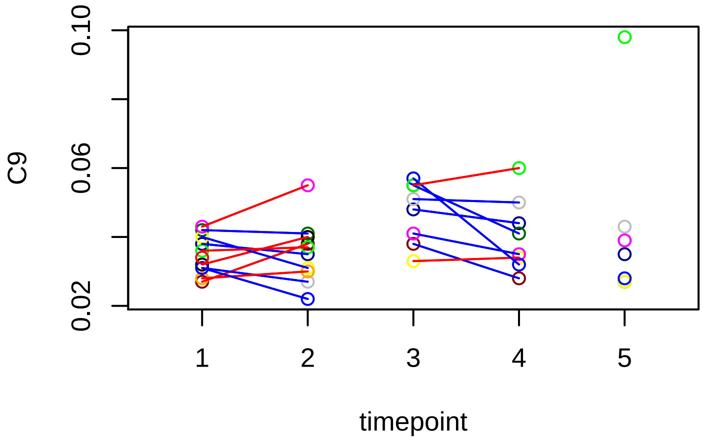

C9
0.02
0.06
0.10
1
2
3
4
5
timepoint

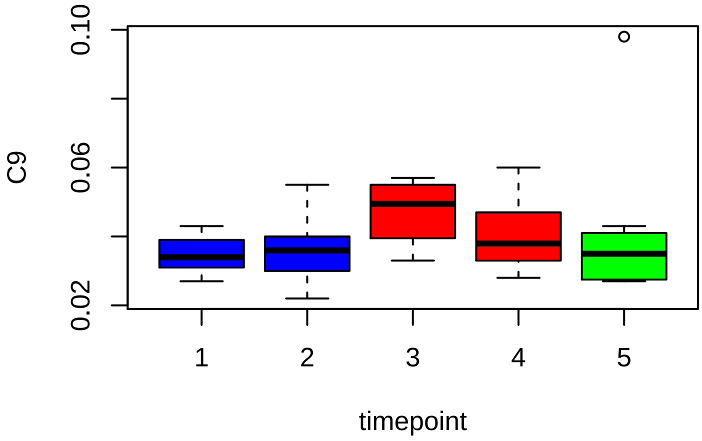

C9
0.02
0.06
0.10
1
2
3
4
5
timepoint

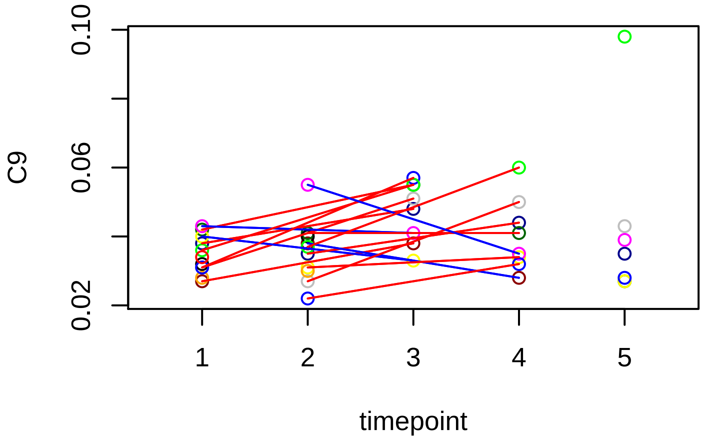

C9
0.02
0.06
0.10
1
2
3
4
5
timepoint

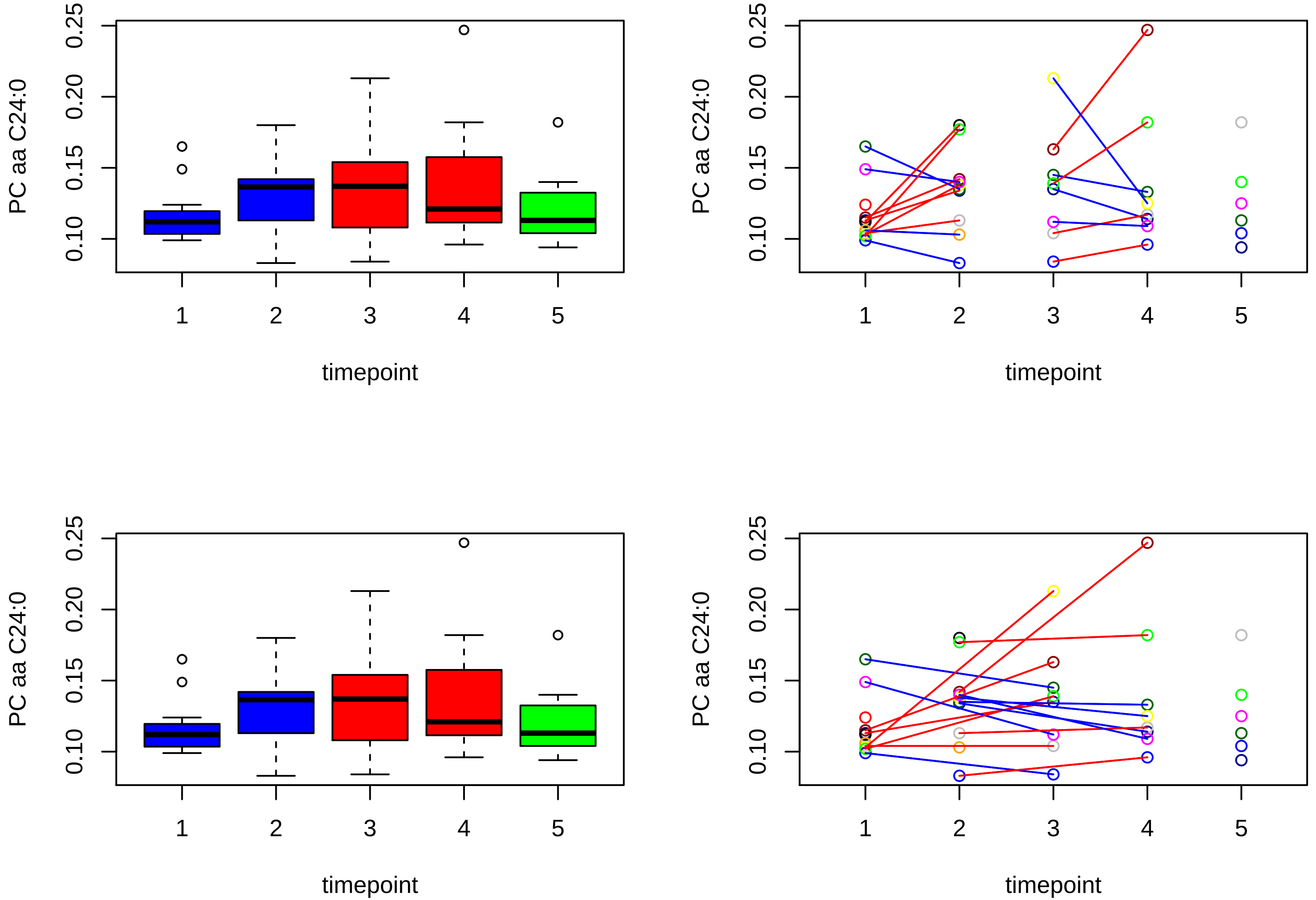
PC aa C24:0
0.10
0.15
0.20
0.25
1
2
3
4
5
timepoint

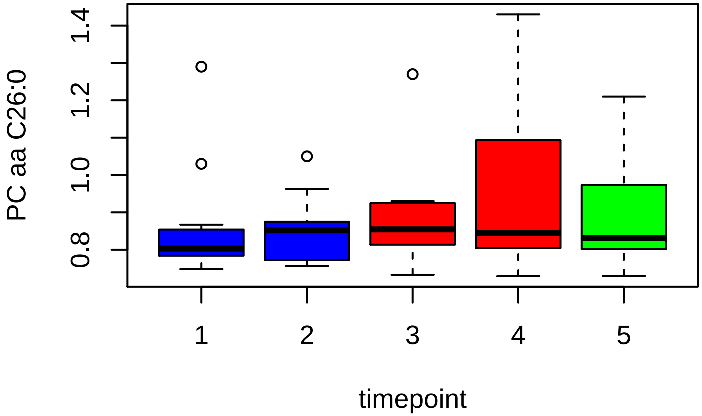
PC aa C26:0
0.8
1.0
1.2
1.4
1
2
3
4
5
timepoint

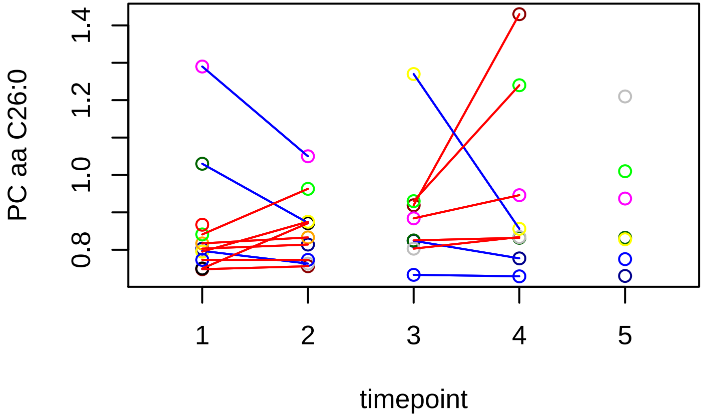
PC aa C26:0
0.8
1.0
1.2
1.4
1
2
3
4
5
timepoint

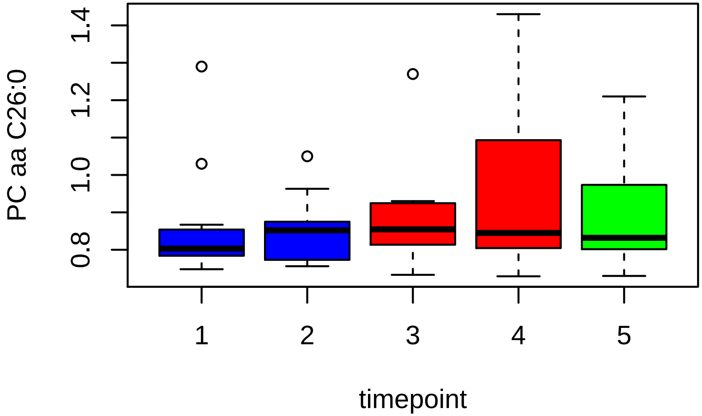
PC aa C26:0
0.8
1.0
1.2
1.4
1
2
3
4
5
timepoint

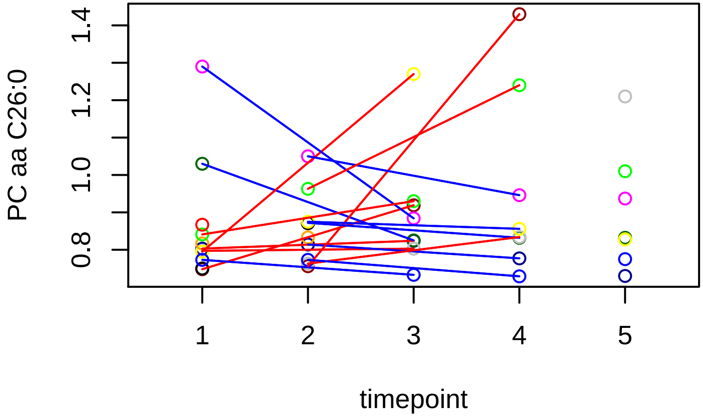
PC aa C26:0
0.8
1.0
1.2
1.4
1
2
3
4
5
timepoint

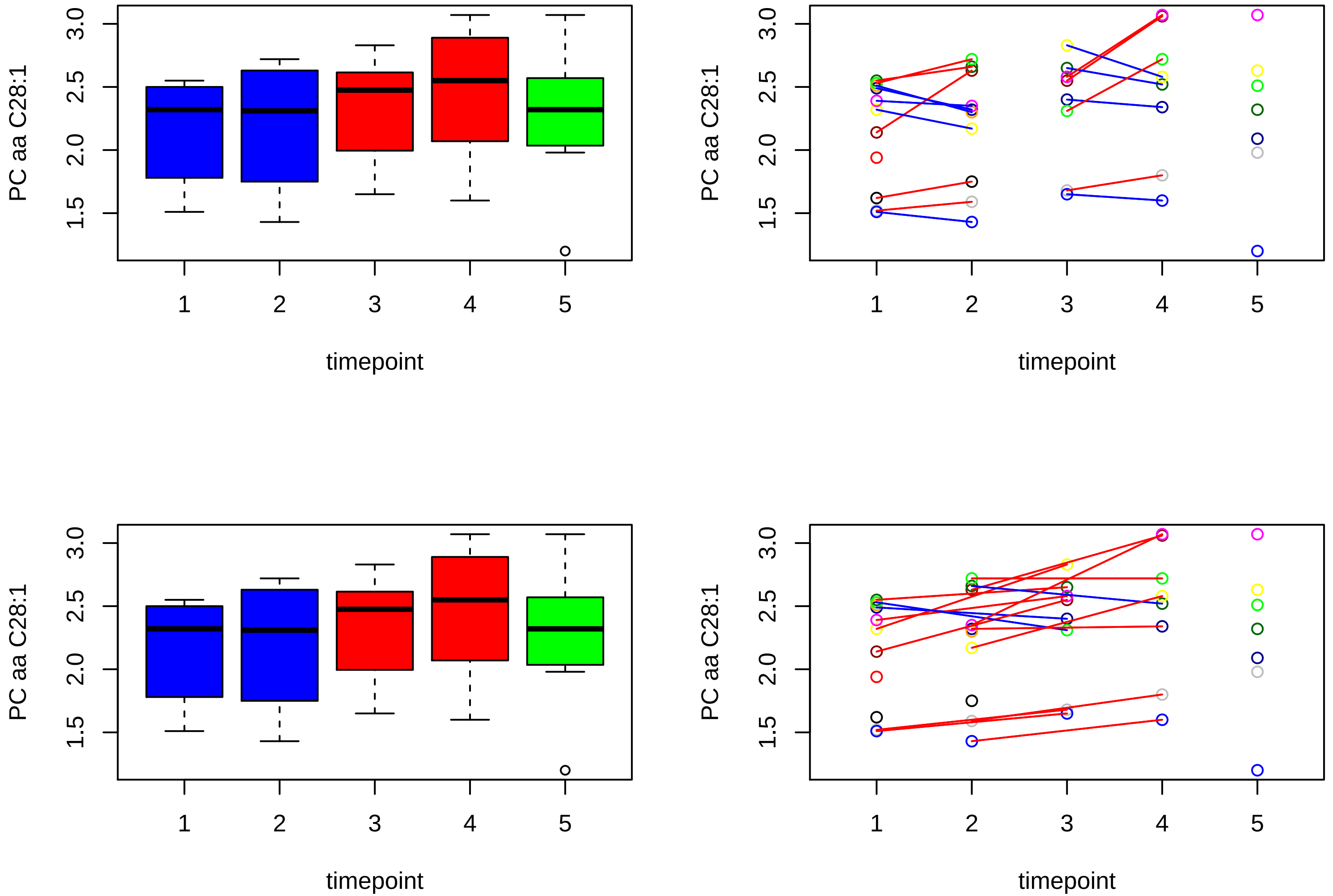
PC aa C28:1
timepoint
1.5
2.0
2.5
3.0
1
2
3
4
5

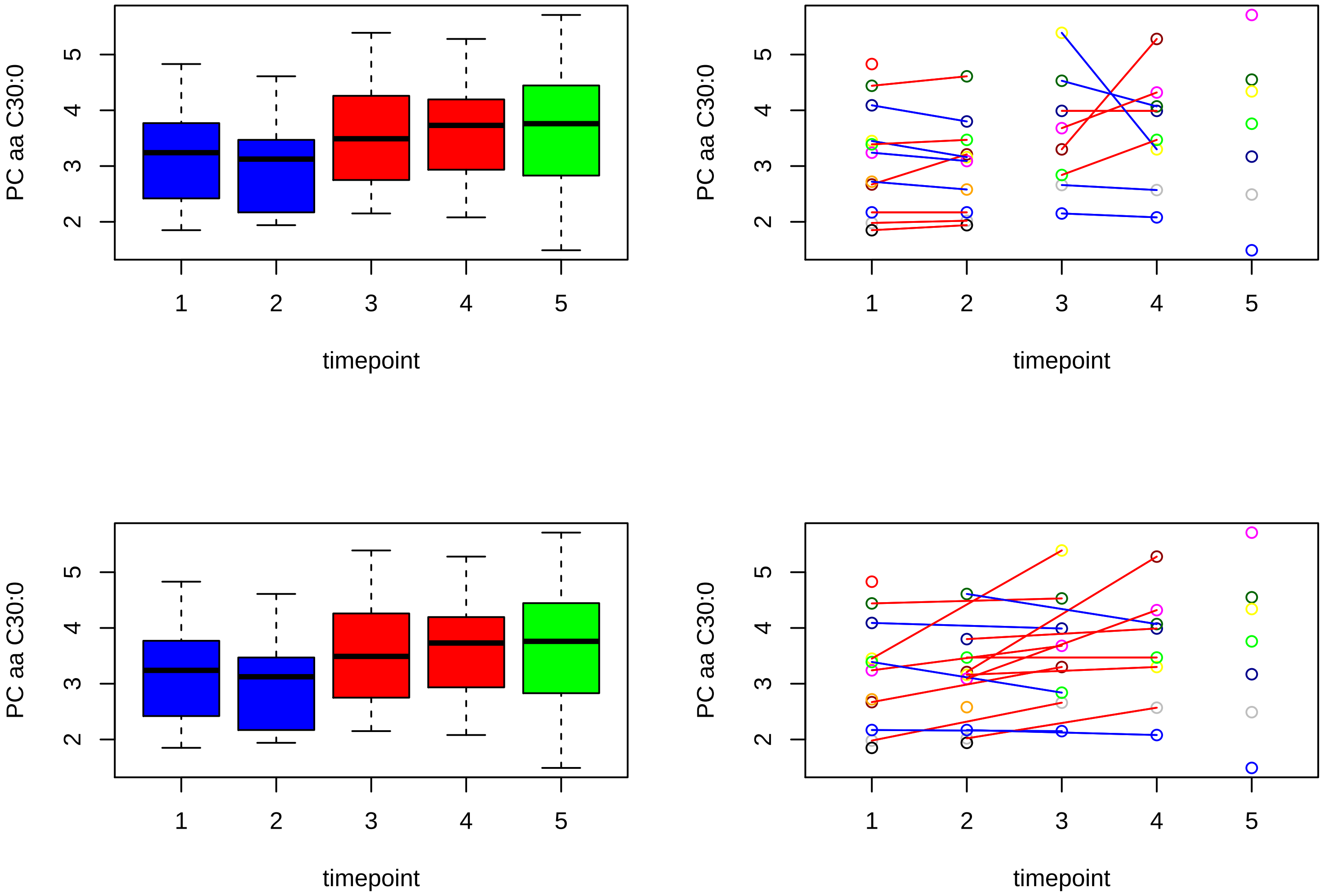
PC aa C30:0
timepoint
1
2
3
4
5

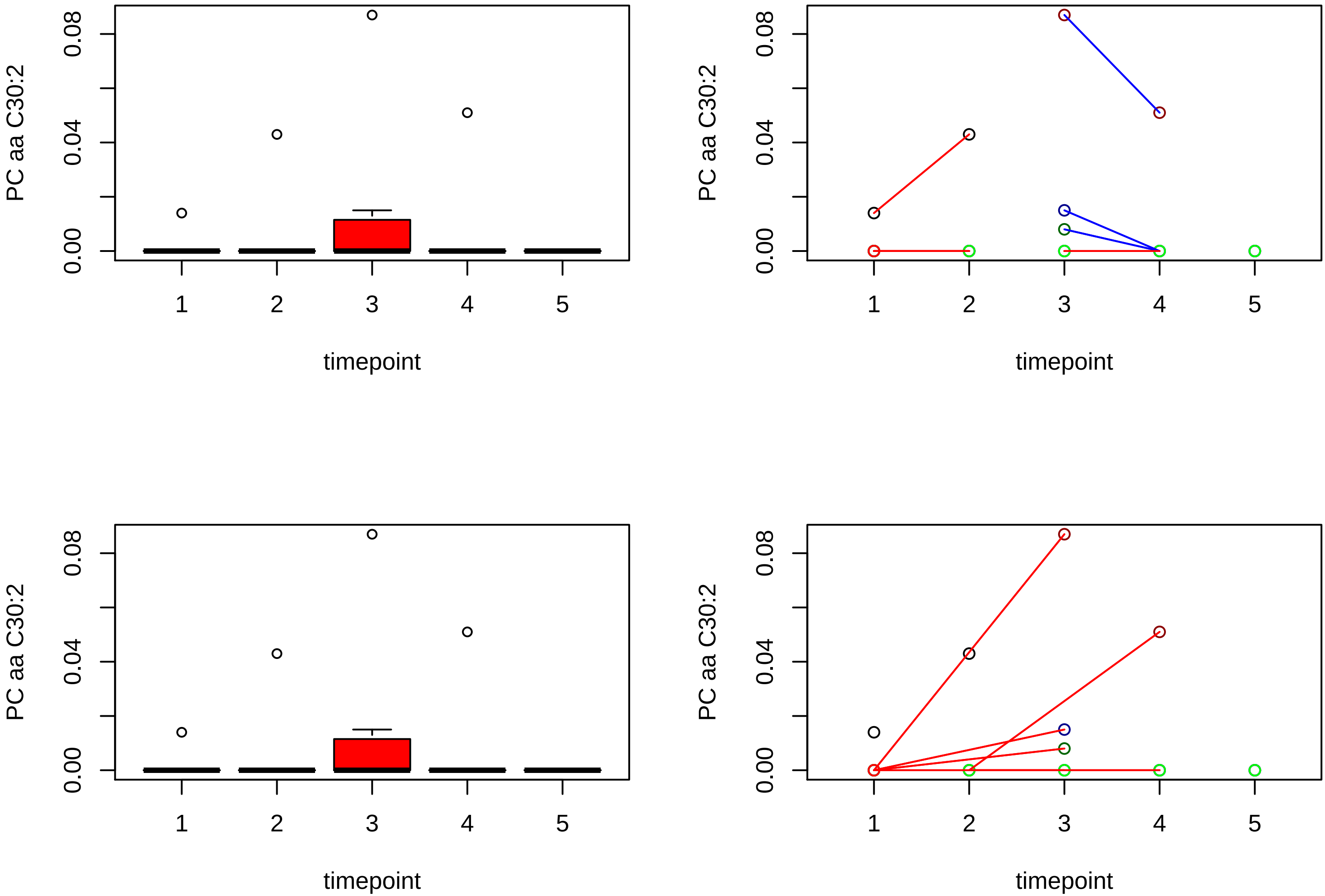

PC aa C30:2
0.00
0.04
0.08
1
2
3
4
5
timepoint

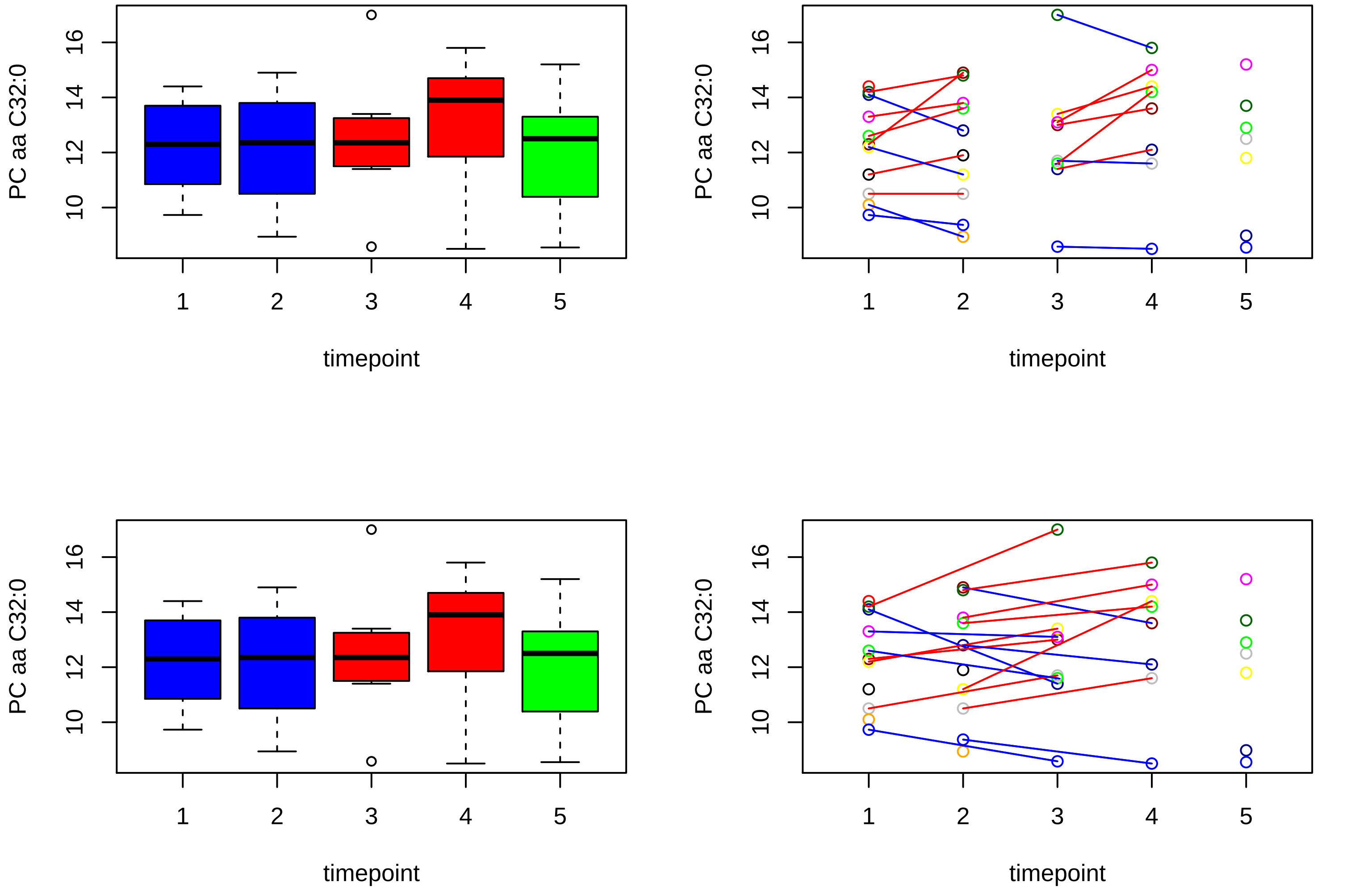

PC aa C32:0
timepoint
10
12
14
16
1
2
3
4
5

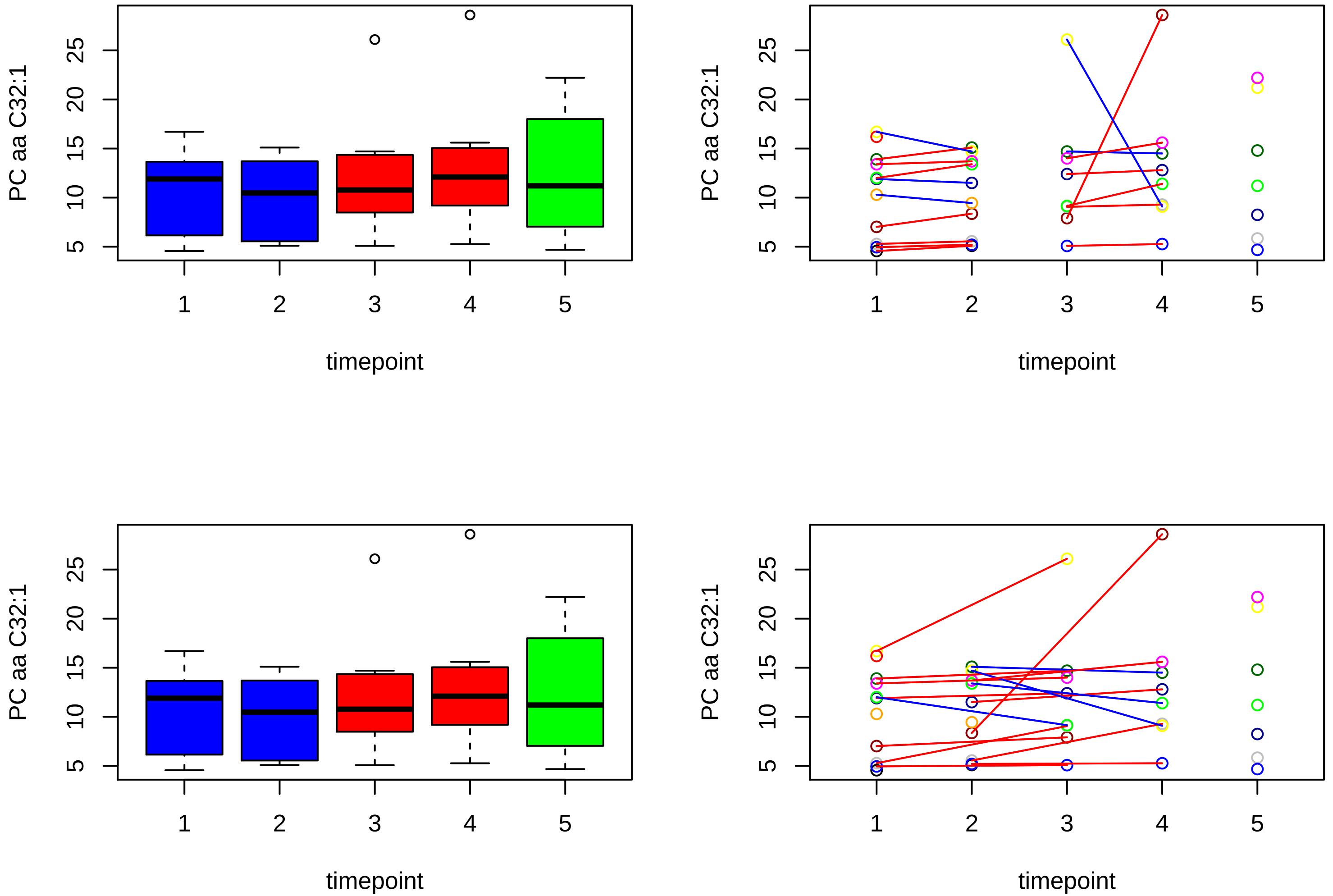
PC aa C32:1
timepoint
5
10
15
20
25
1
2
3
4
5

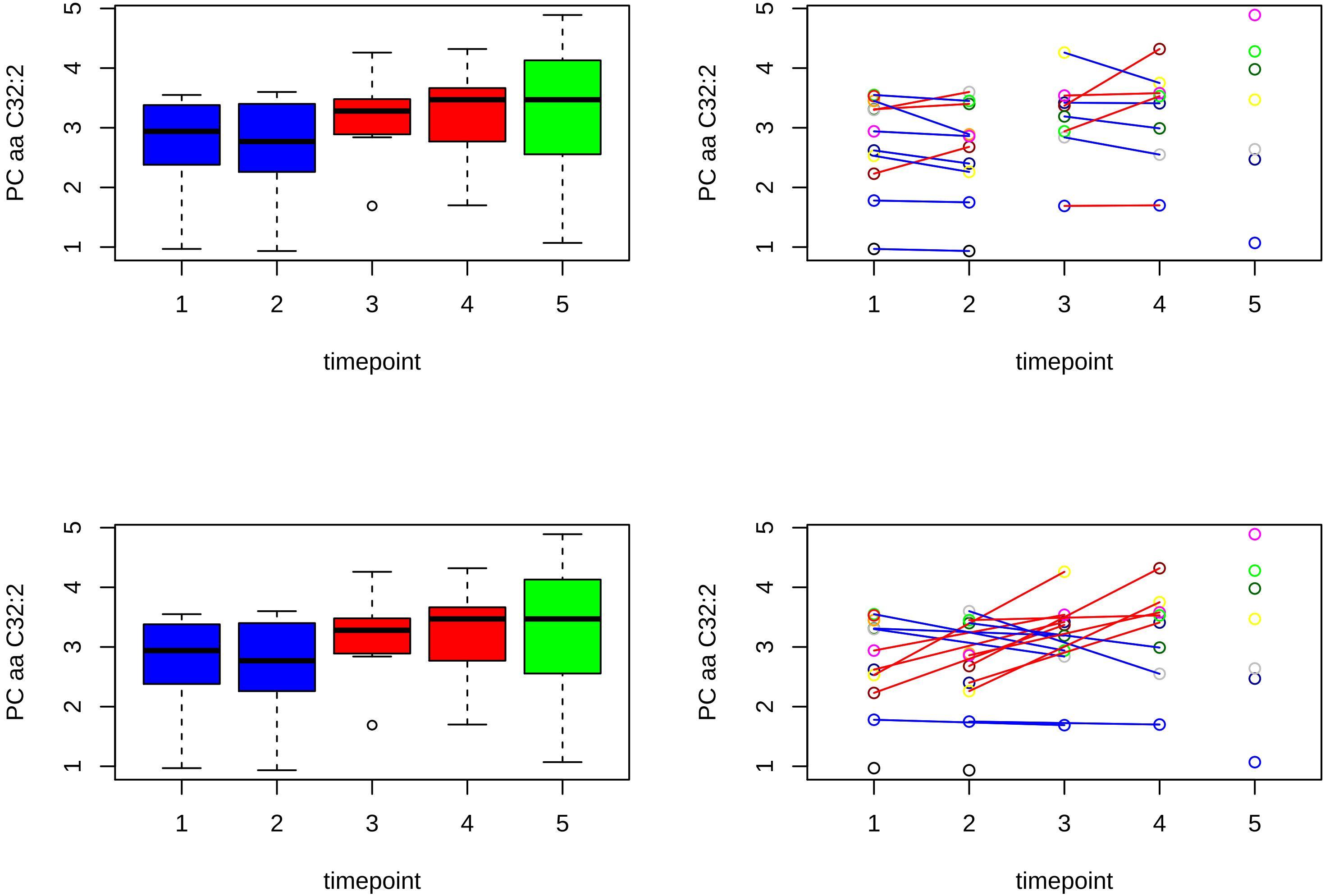

PC aa C32:2
timepoint
1
2
3
4
5

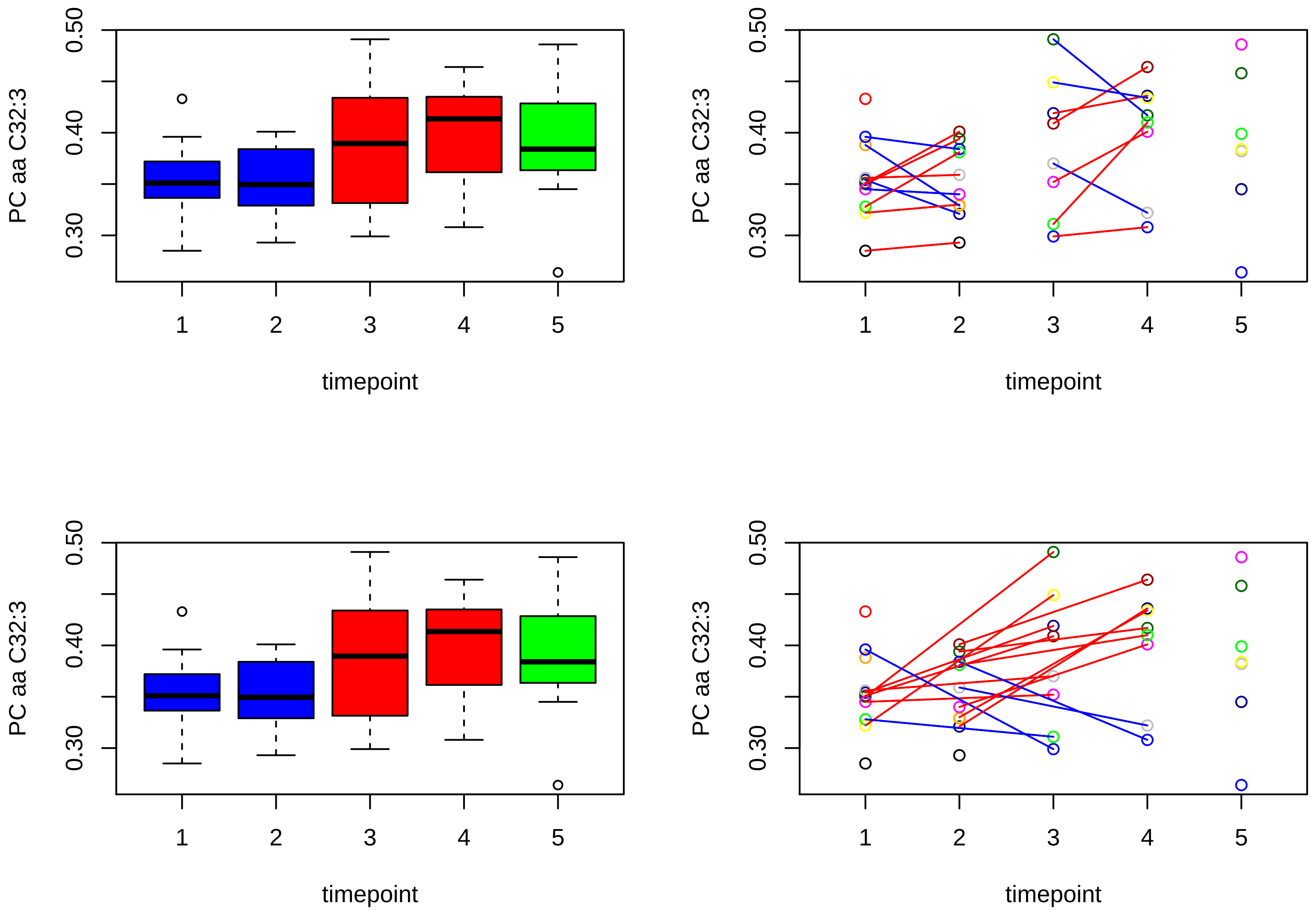

PC aa C32:3
0.30
0.40
0.50
1
2
3
4
5
timepoint

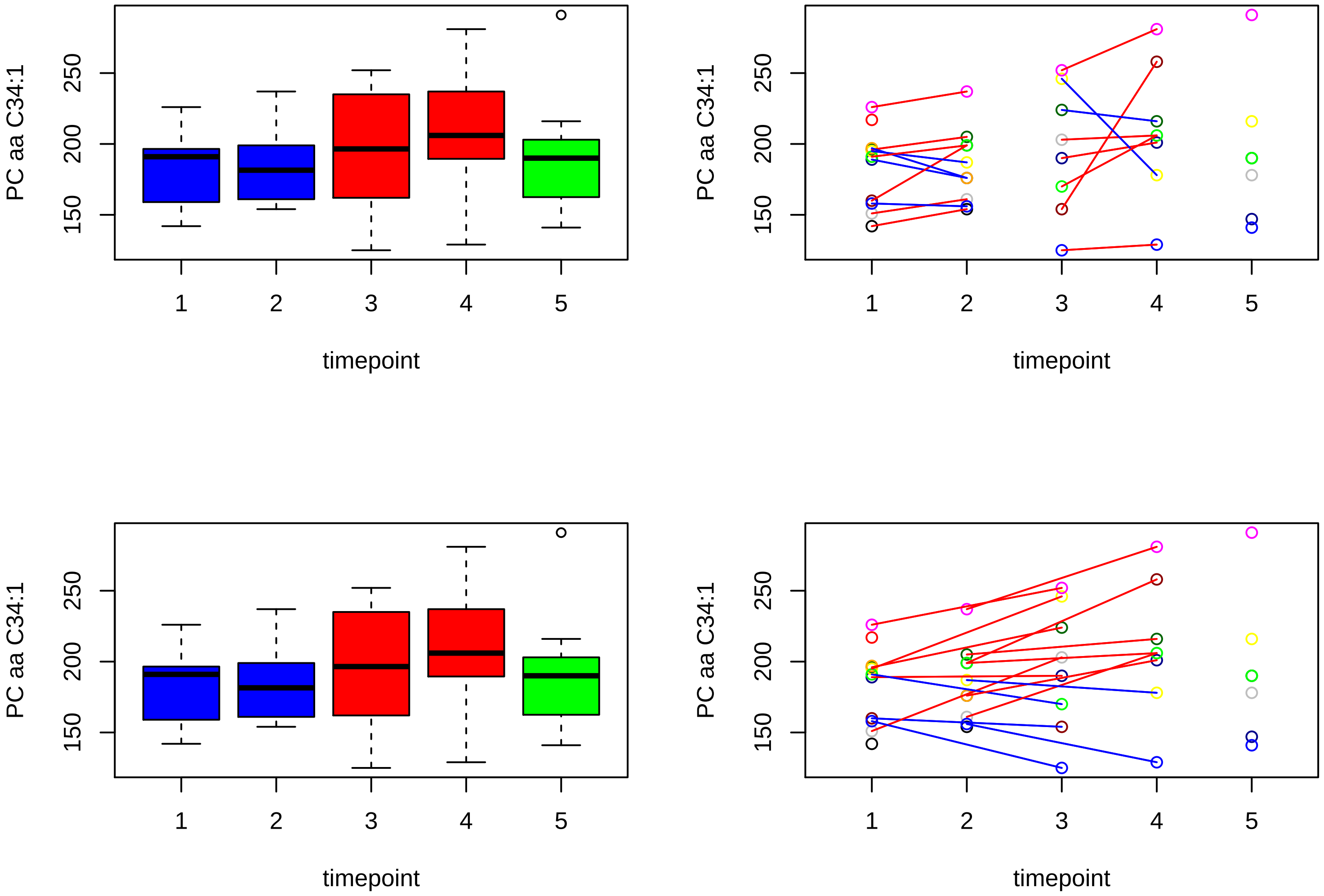
PC aa C34:1
150
200
250
1
2
3
4
5
timepoint
PC aa C34:1
150
200
250
1
2
3
4
5
timepoint
PC aa C34:1
150
200
250
1
2
3
4
5
timepoint
PC aa C34:1
150
200
250
1
2
3
4
5
timepoint

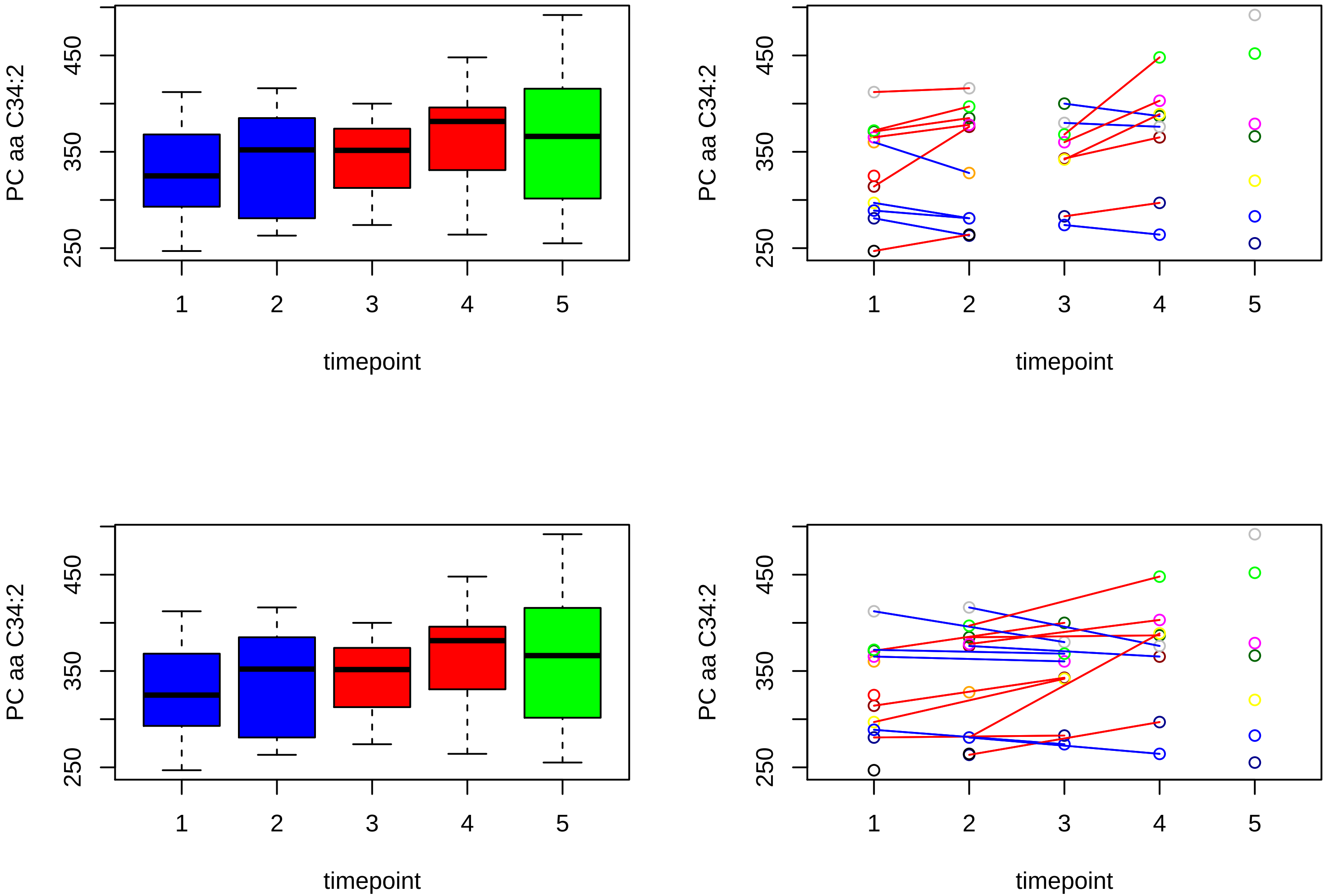

PC aa C34:2
250
350
450
1
2
3
4
5
timepoint

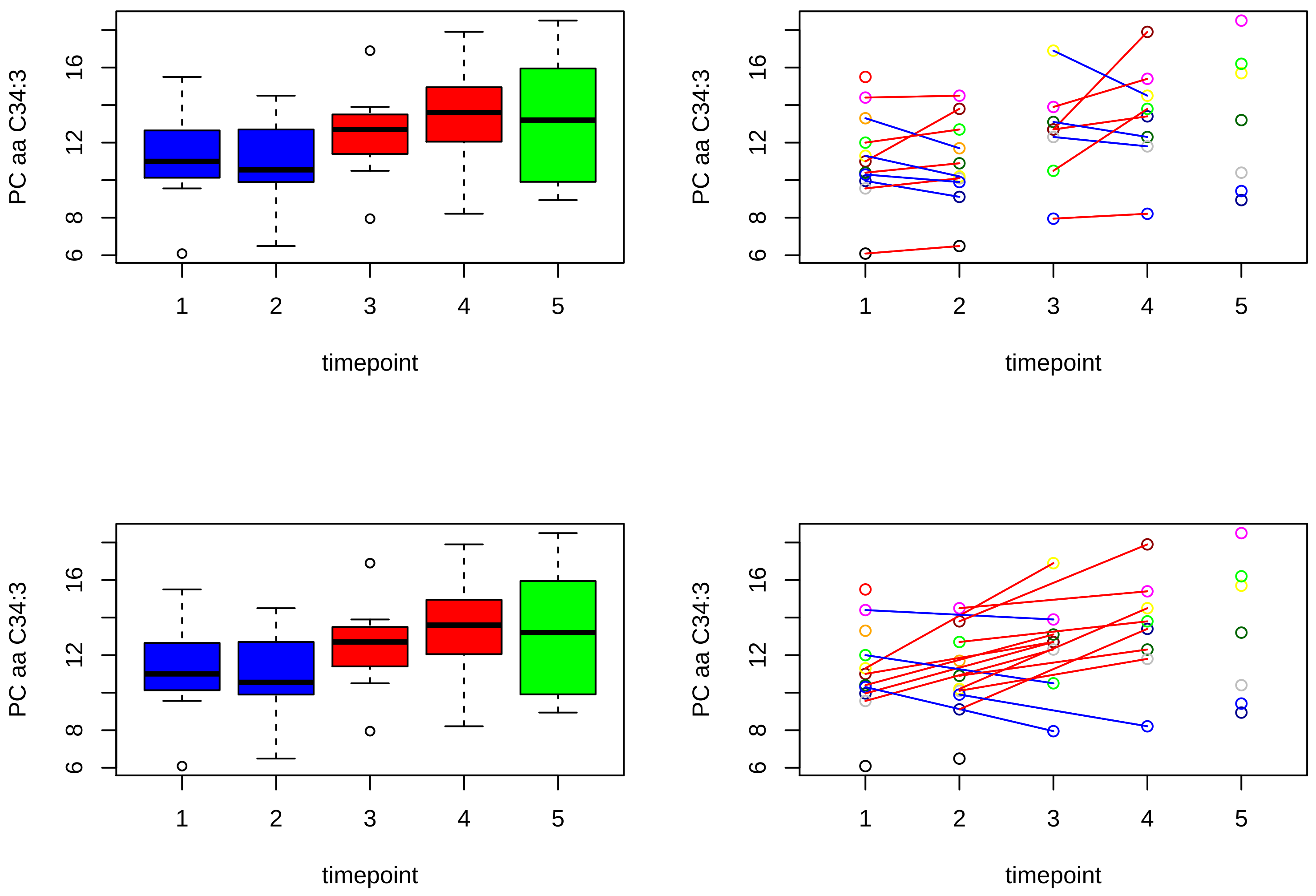
PC aa C34:3
6
8
12
16
1
2
3
4
5
timepoint
PC aa C34:3
6
8
12
16
1
2
3
4
5
timepoint
PC aa C34:3
6
8
12
16
1
2
3
4
5
timepoint
PC aa C34:3
6
8
12
16
1
2
3
4
5
timepoint

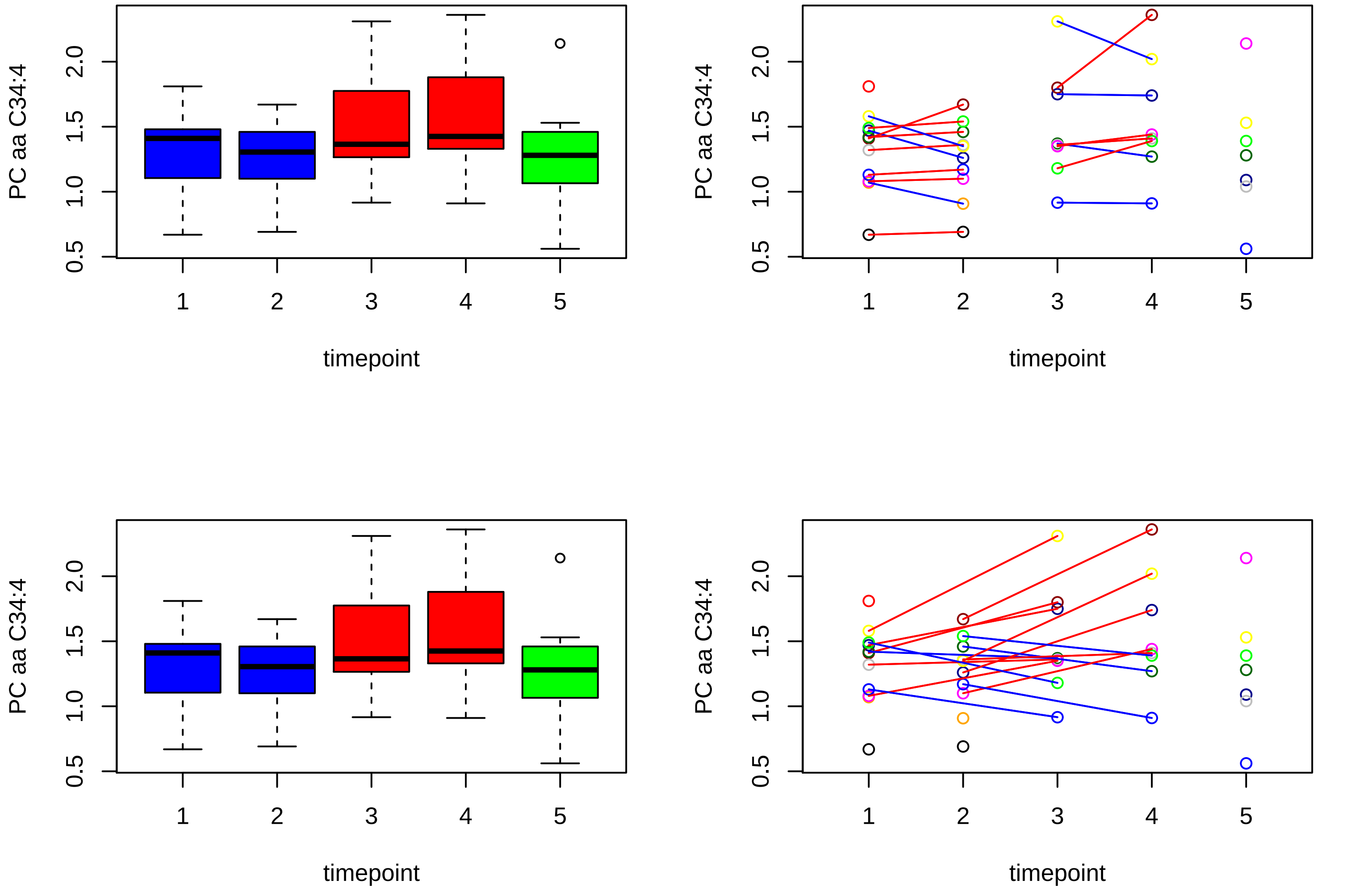
PC aa C34:4
timepoint
0.5
1.0
1.5
2.0
1
2
3
4
5

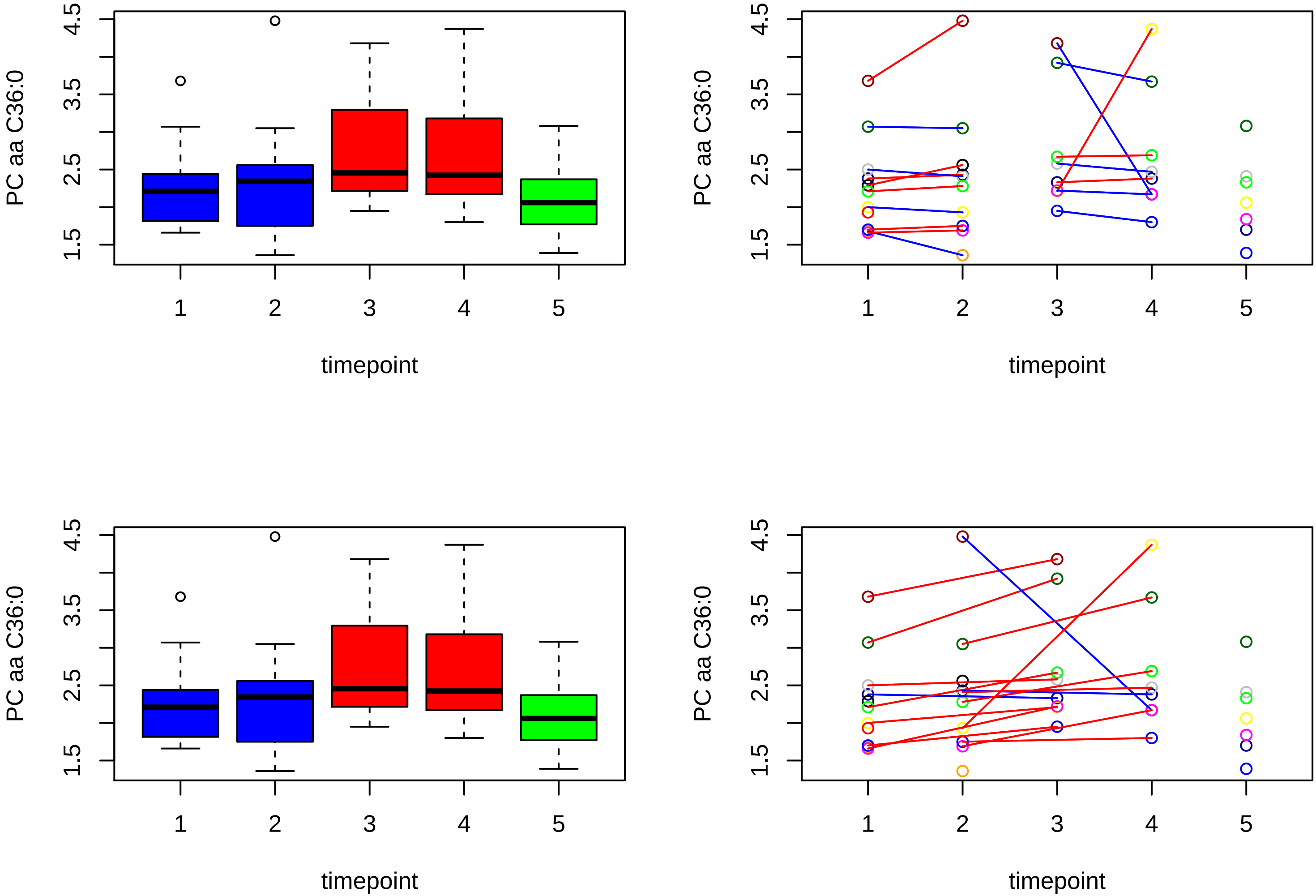

PC aa C36:0
timepoint
1.5
2.5
3.5
4.5
1
2
3
4
5

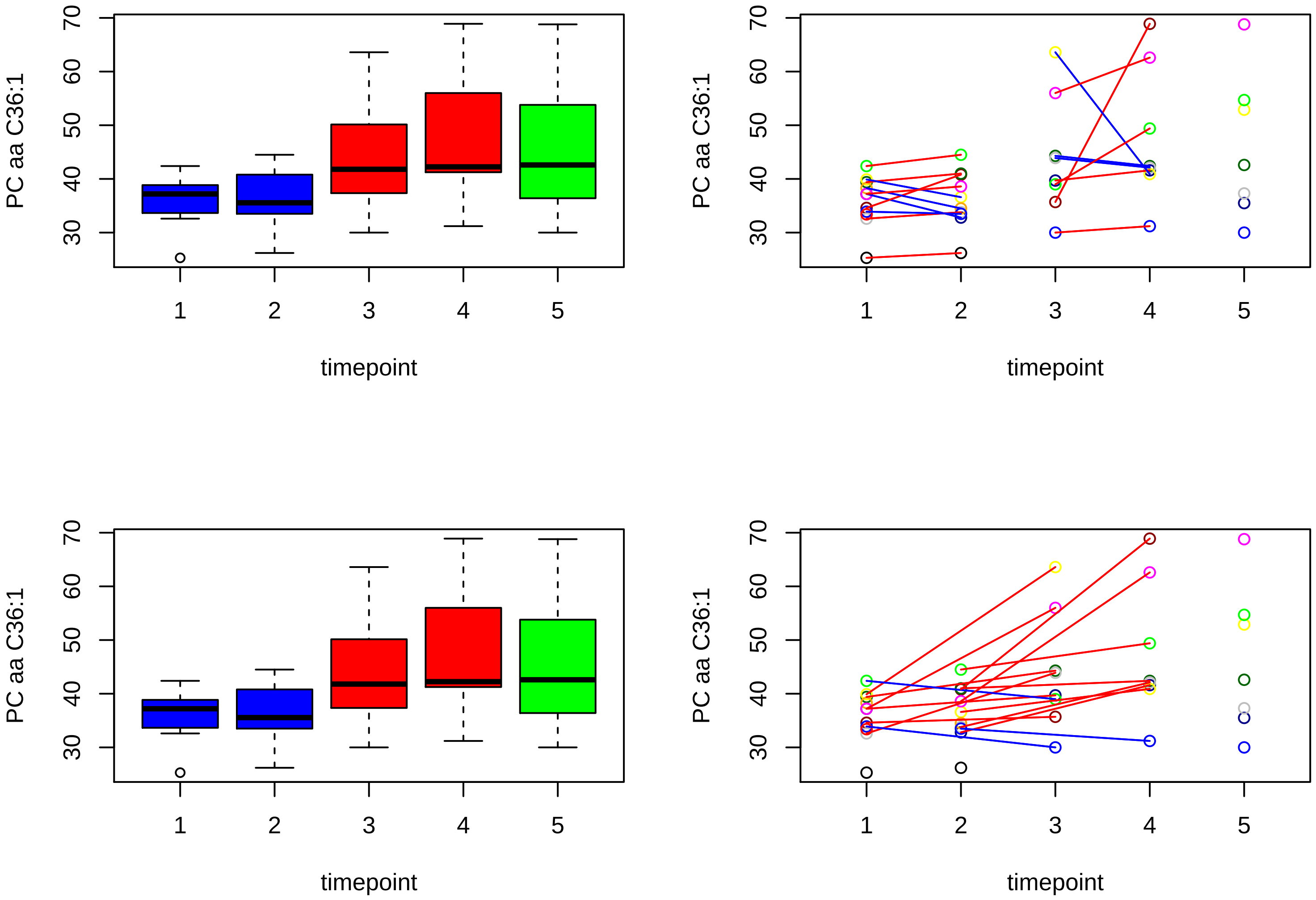
PC aa C36:1
70
60
50
40
30
1
2
3
4
5
timepoint
PC aa C36:1
70
60
50
40
30
1
2
3
4
5
timepoint
PC aa C36:1
70
60
50
40
30
1
2
3
4
5
timepoint
PC aa C36:1
70
60
50
40
30
1
2
3
4
5
timepoint

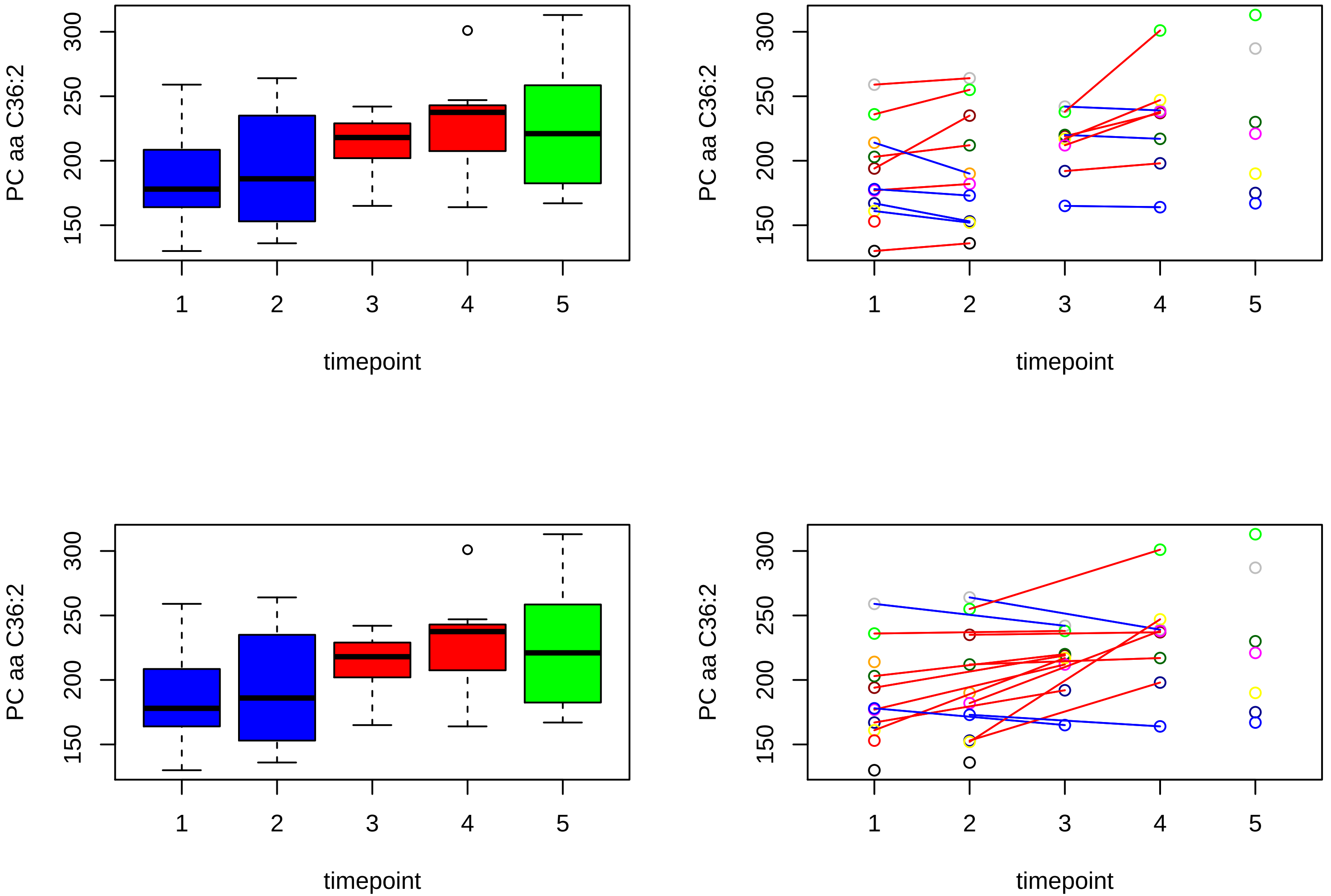
PC aa C36:2
timepoint
150
200
250
300
1
2
3
4
5

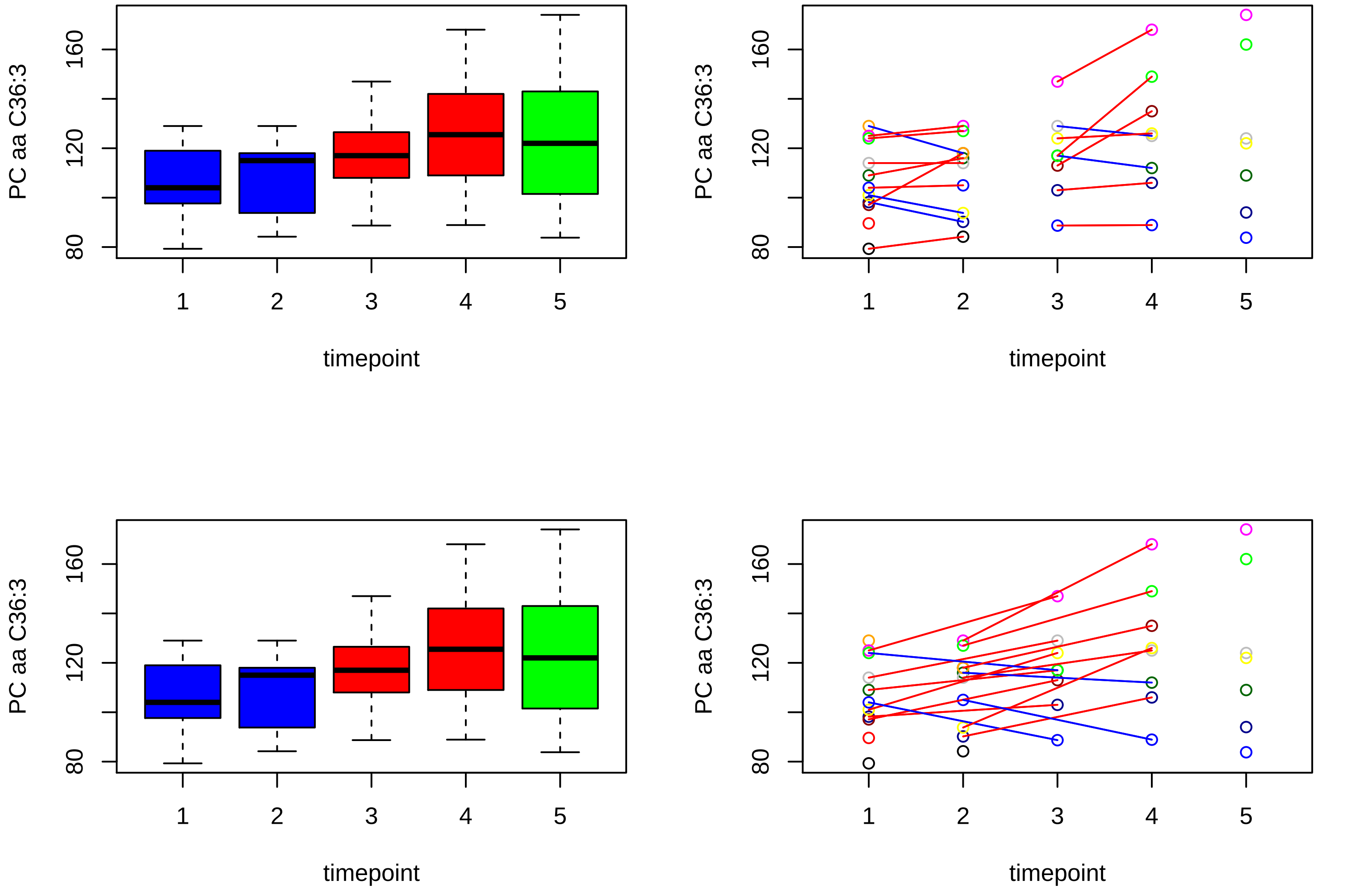

PC aa C36:3
80
120
160
1
2
3
4
5
timepoint

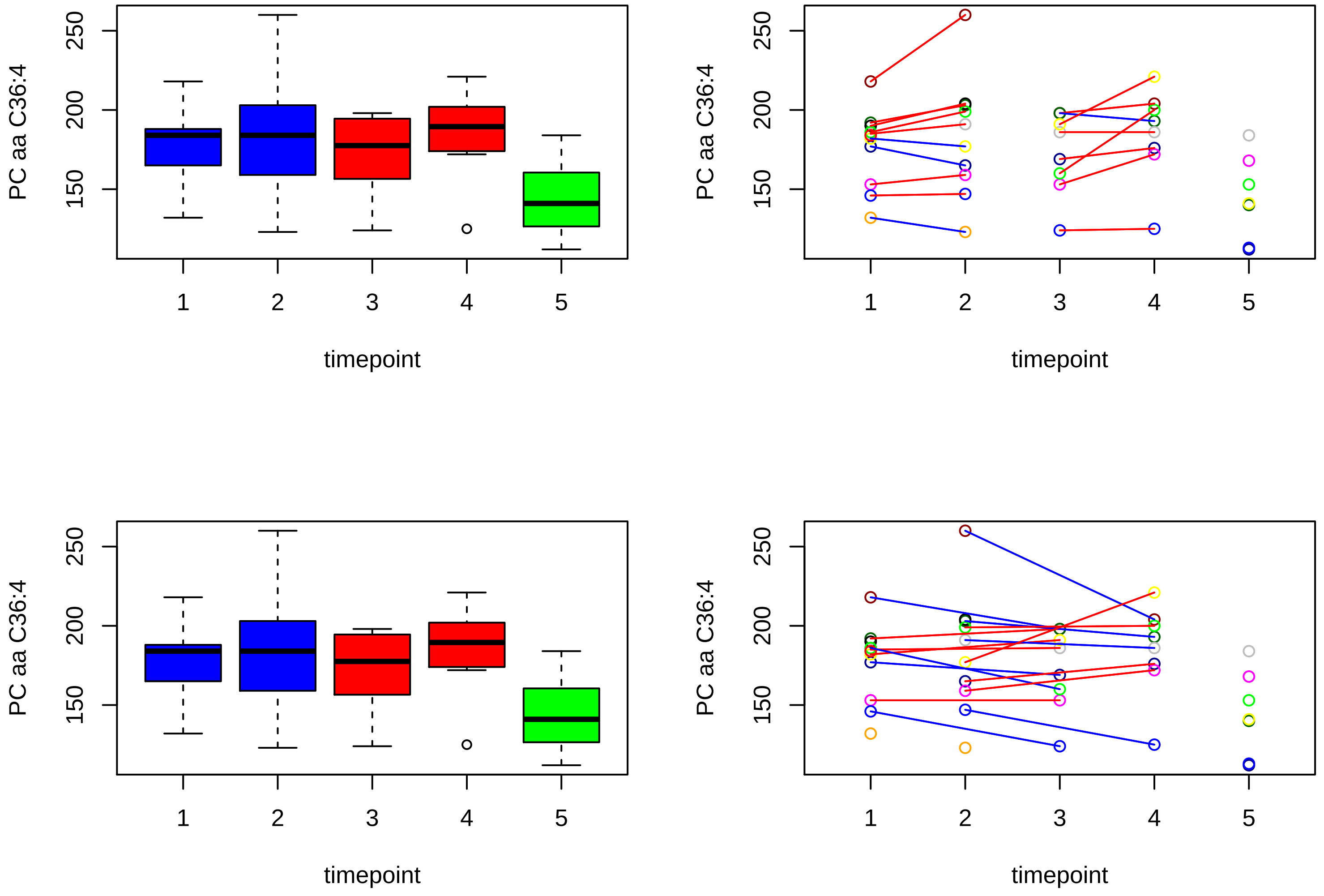
PC aa C36:4
timepoint
250
200
150
1
2
3
4
5

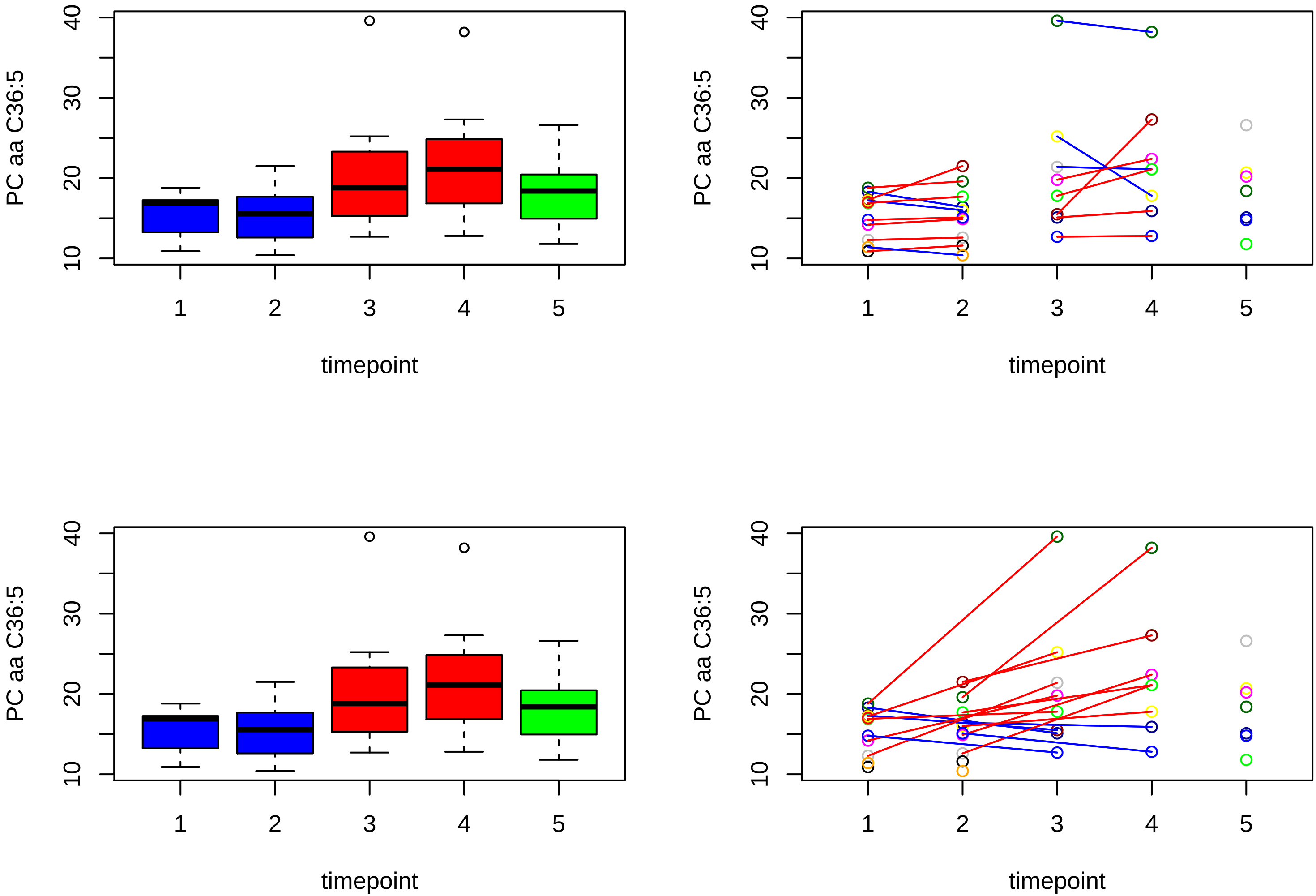

PC aa C36:5
timepoint
PC aa C36:5
timepoint
PC aa C36:5
timepoint
PC aa C36:5
timepoint

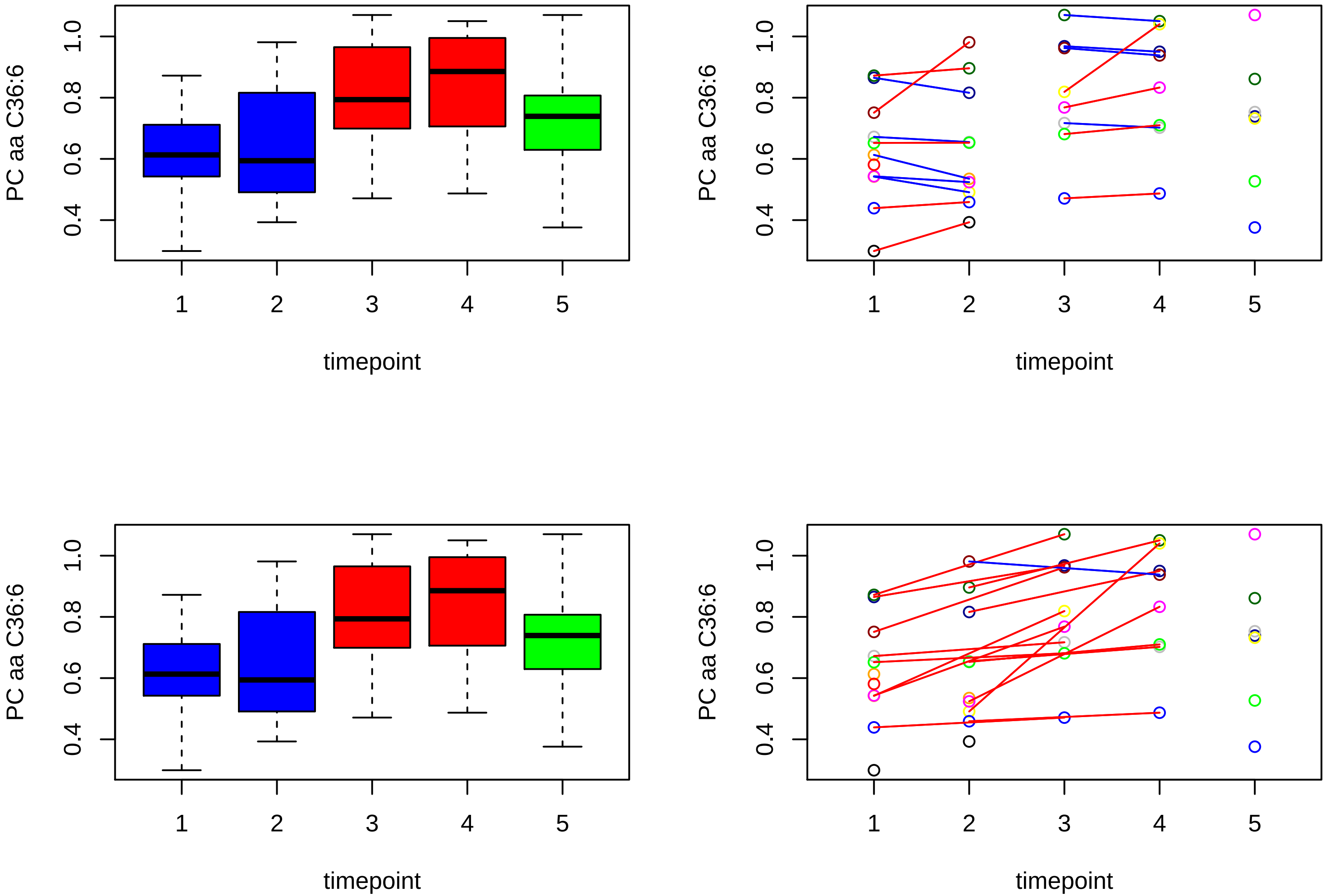

PC aa C36:6
timepoint
PC aa C36:6
timepoint
PC aa C36:6
timepoint
PC aa C36:6
timepoint

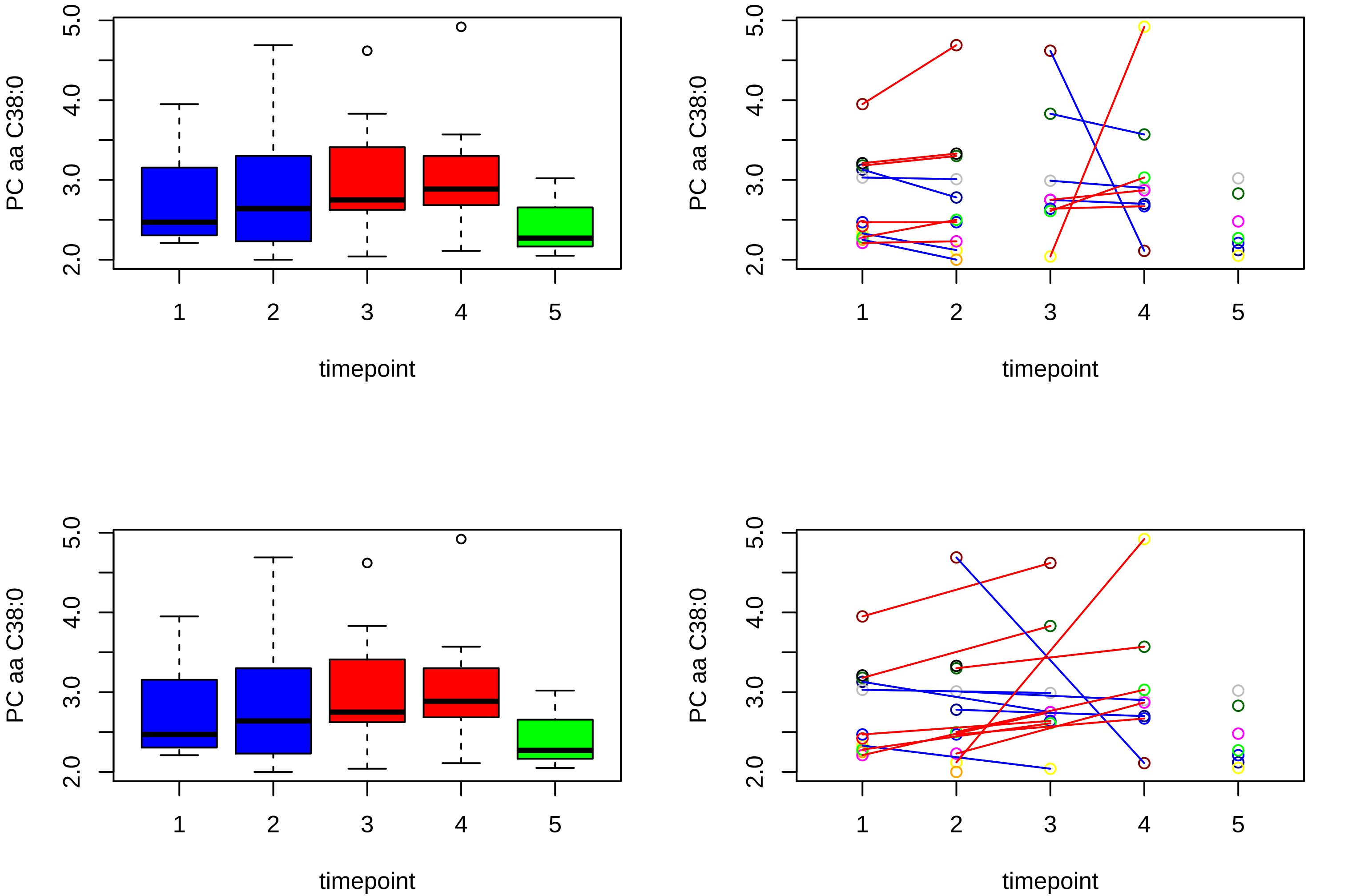
PC aa C38:0
5.0
4.0
3.0
2.0
1
2
3
4
5
timepoint

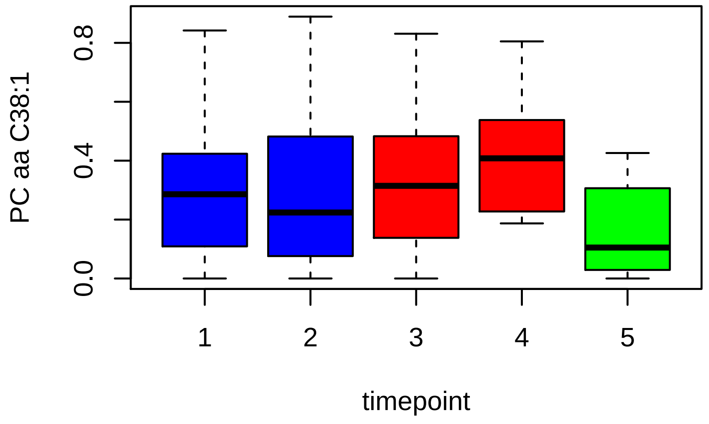
PC aa C38:1
0.0
0.4
0.8
1
2
3
4
5
timepoint

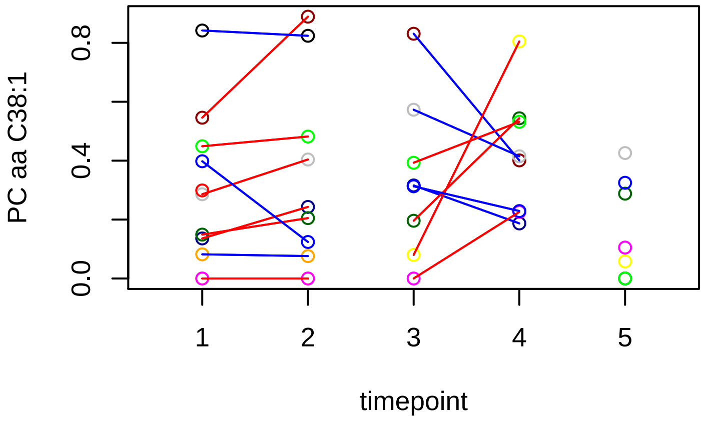
PC aa C38:1
0.0
0.4
0.8
1
2
3
4
5
timepoint

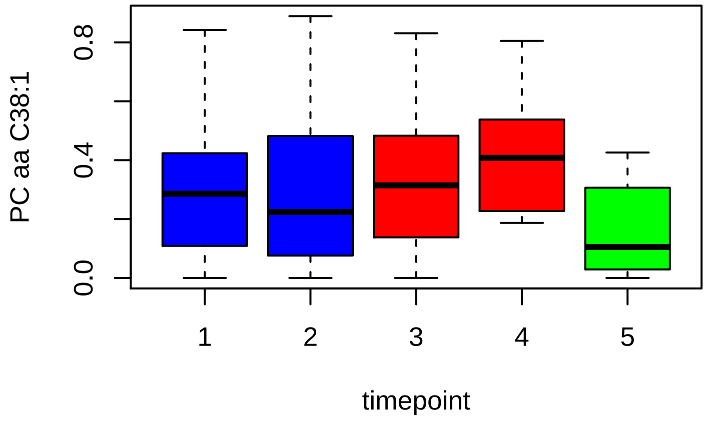
PC aa C38:1
0.0
0.4
0.8
1
2
3
4
5
timepoint

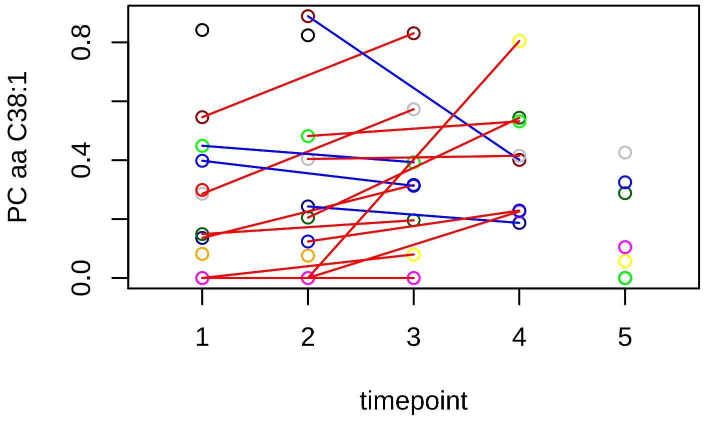
PC aa C38:1
0.0
0.4
0.8
1
2
3
4
5
timepoint

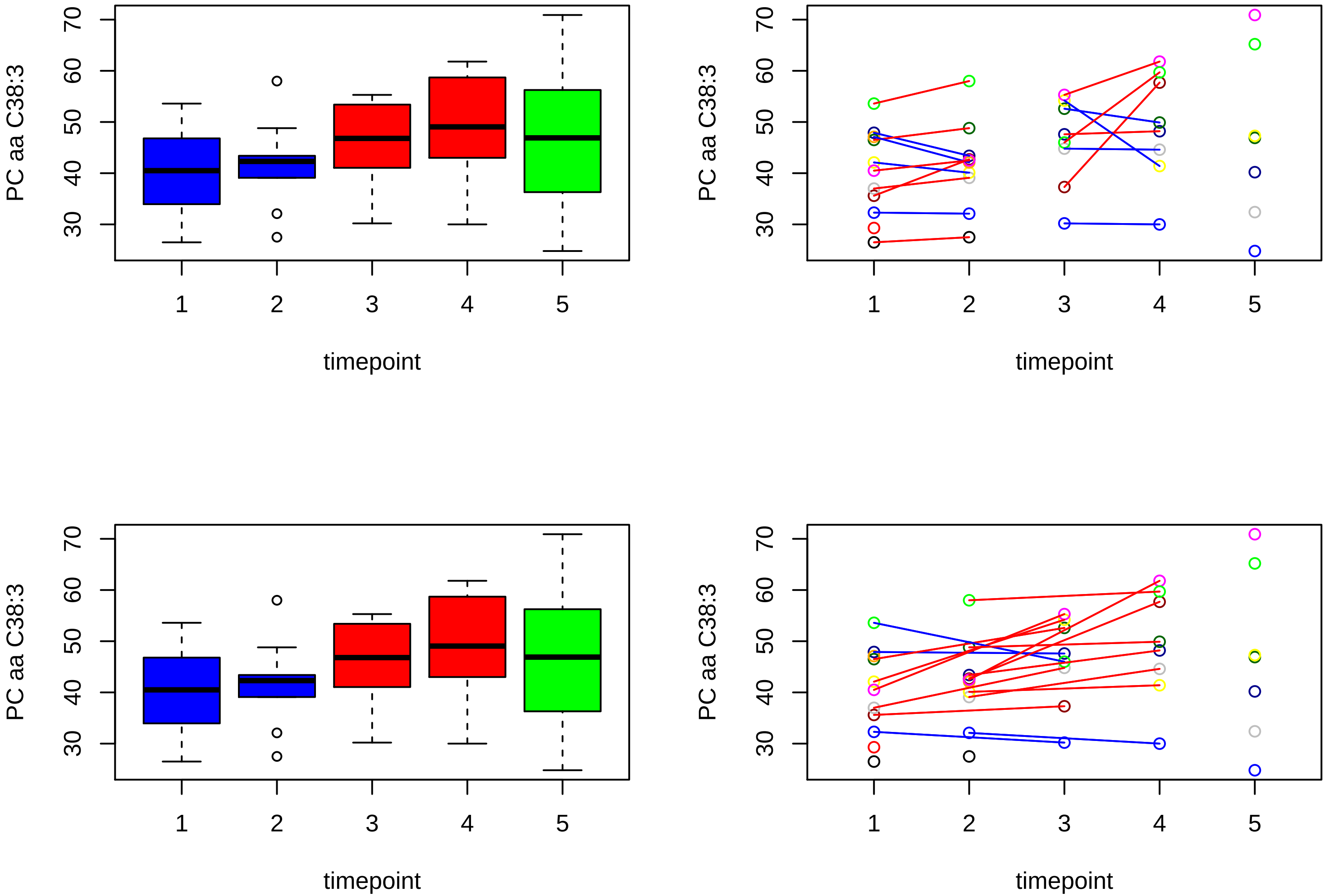

PC aa C38:3
timepoint
PC aa C38:3
timepoint
PC aa C38:3
timepoint
PC aa C38:3
timepoint

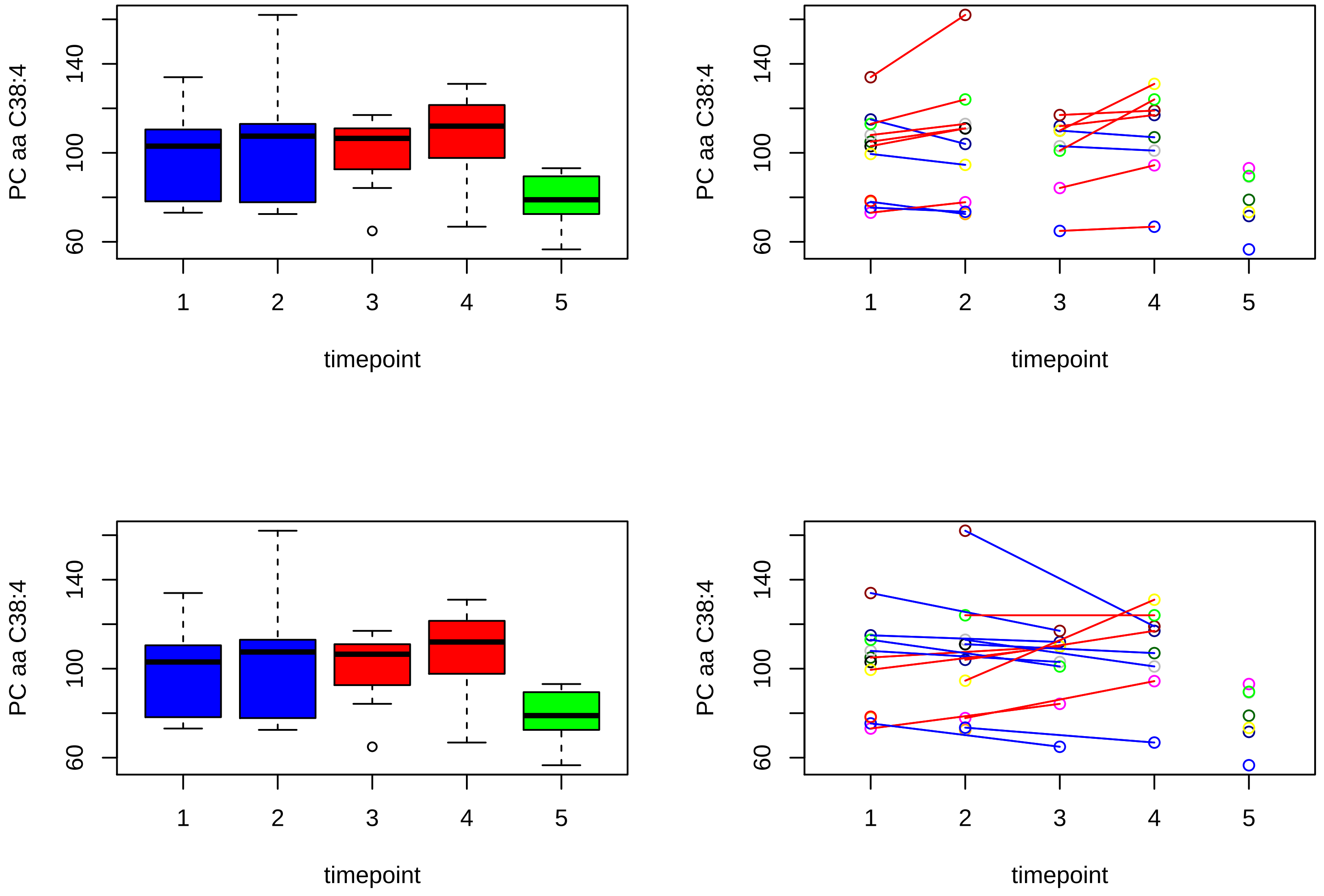
PC aa C38:4
timepoint
60
100
140
1
2
3
4
5

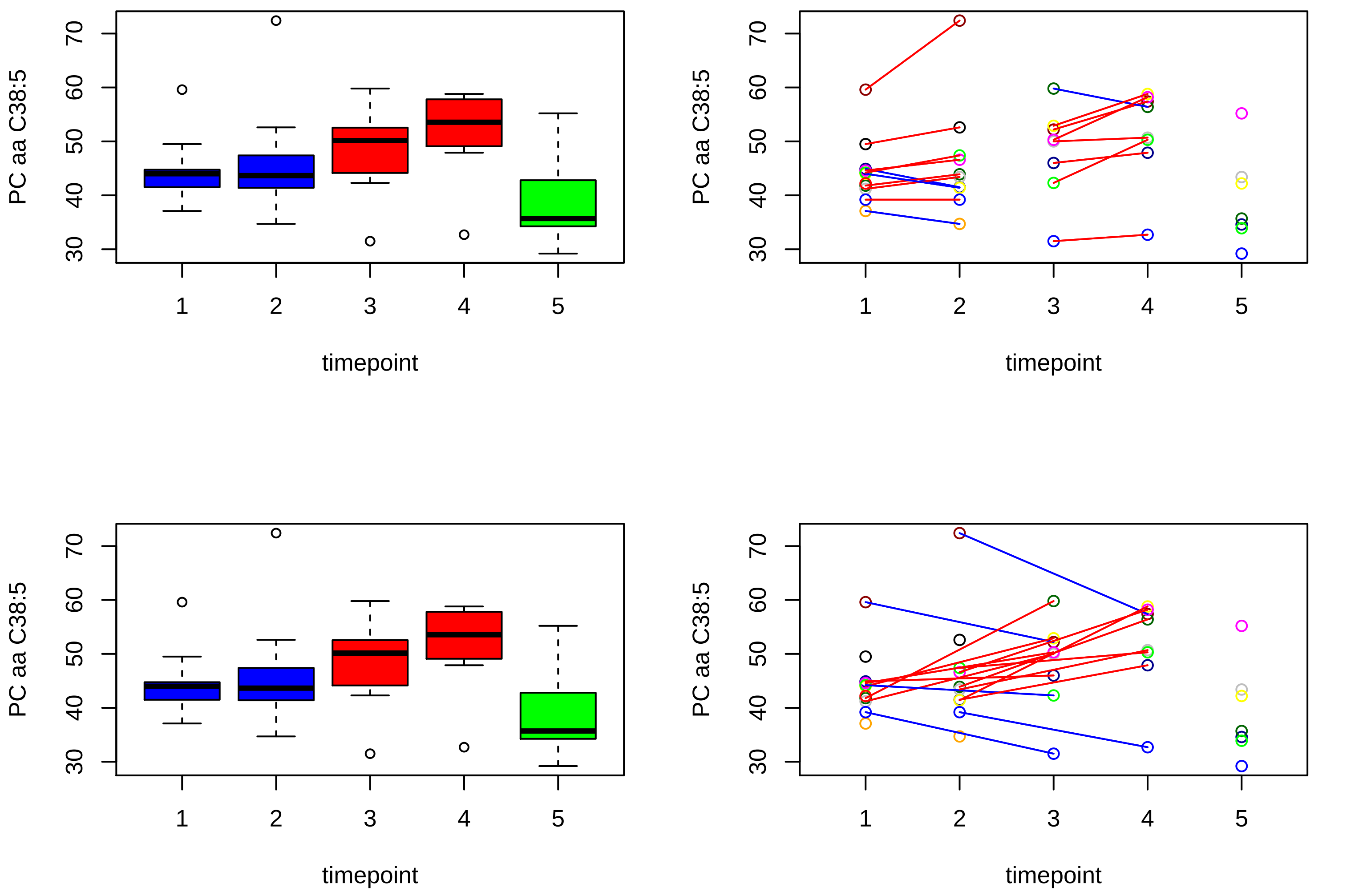

PC aa C38:5
timepoint
1
2
3
4
5
30
40
50
60
70

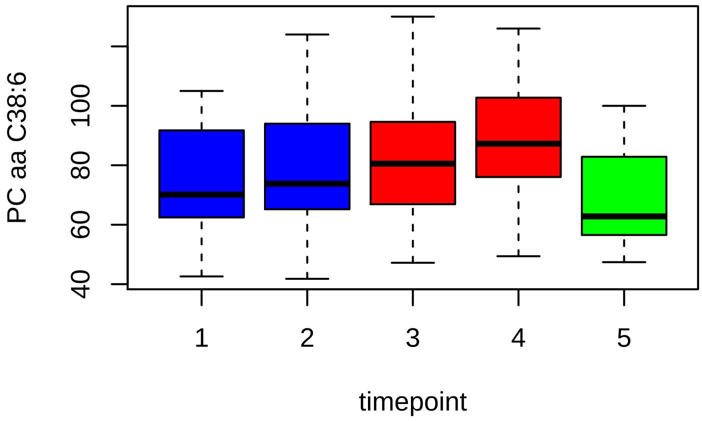
PC aa C38:6
40
60
80
100
1
2
3
4
5
timepoint

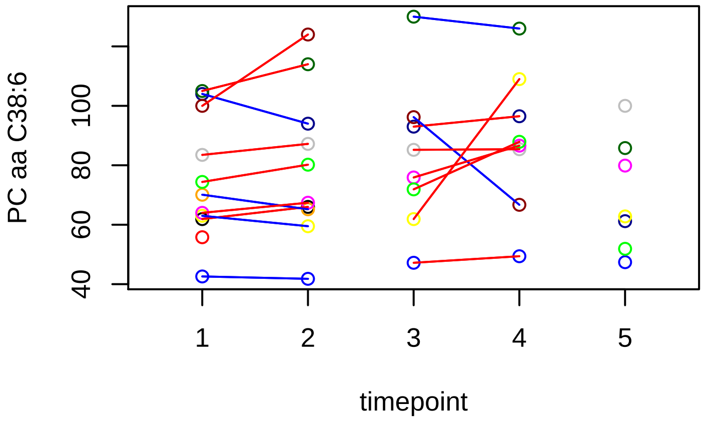
PC aa C38:6
40
60
80
100
1
2
3
4
5
timepoint

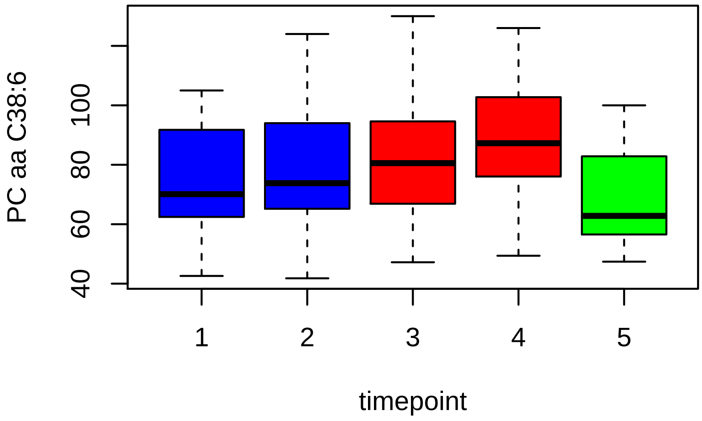
PC aa C38:6
40
60
80
100
1
2
3
4
5
timepoint

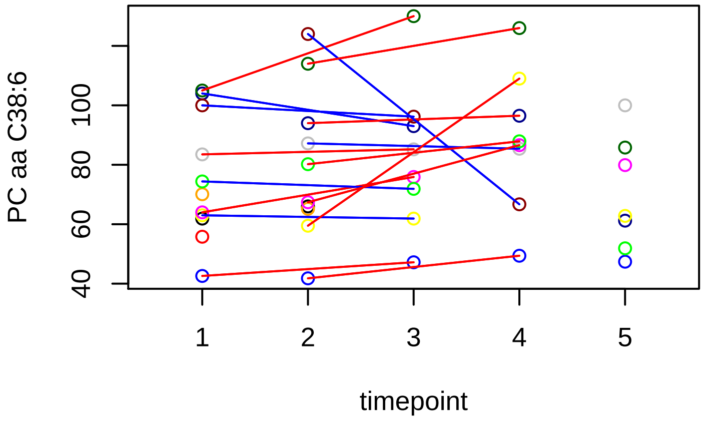
PC aa C38:6
40
60
80
100
1
2
3
4
5
timepoint

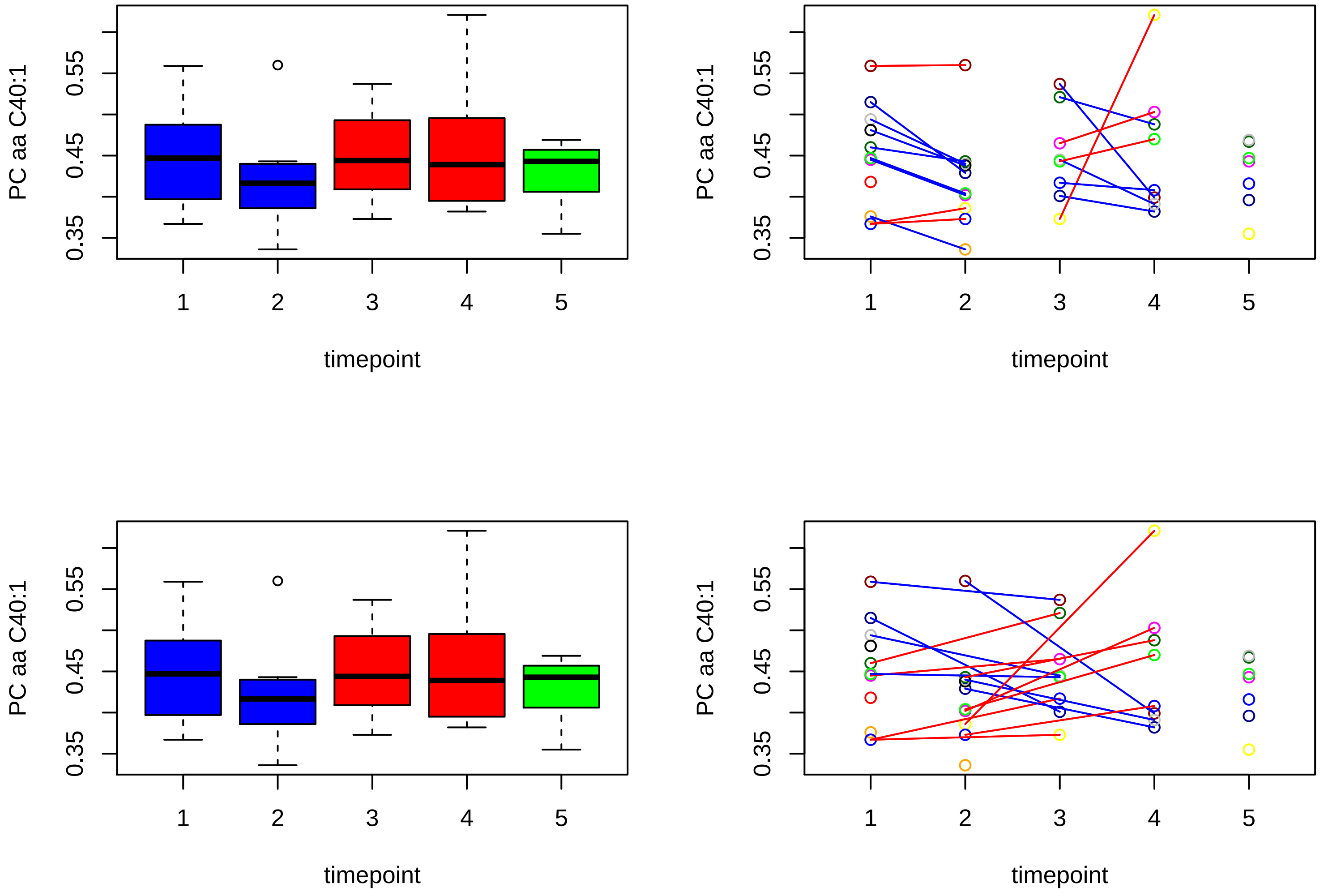

PC aa C40:1
0.35
0.45
0.55
1
2
3
4
5
timepoint

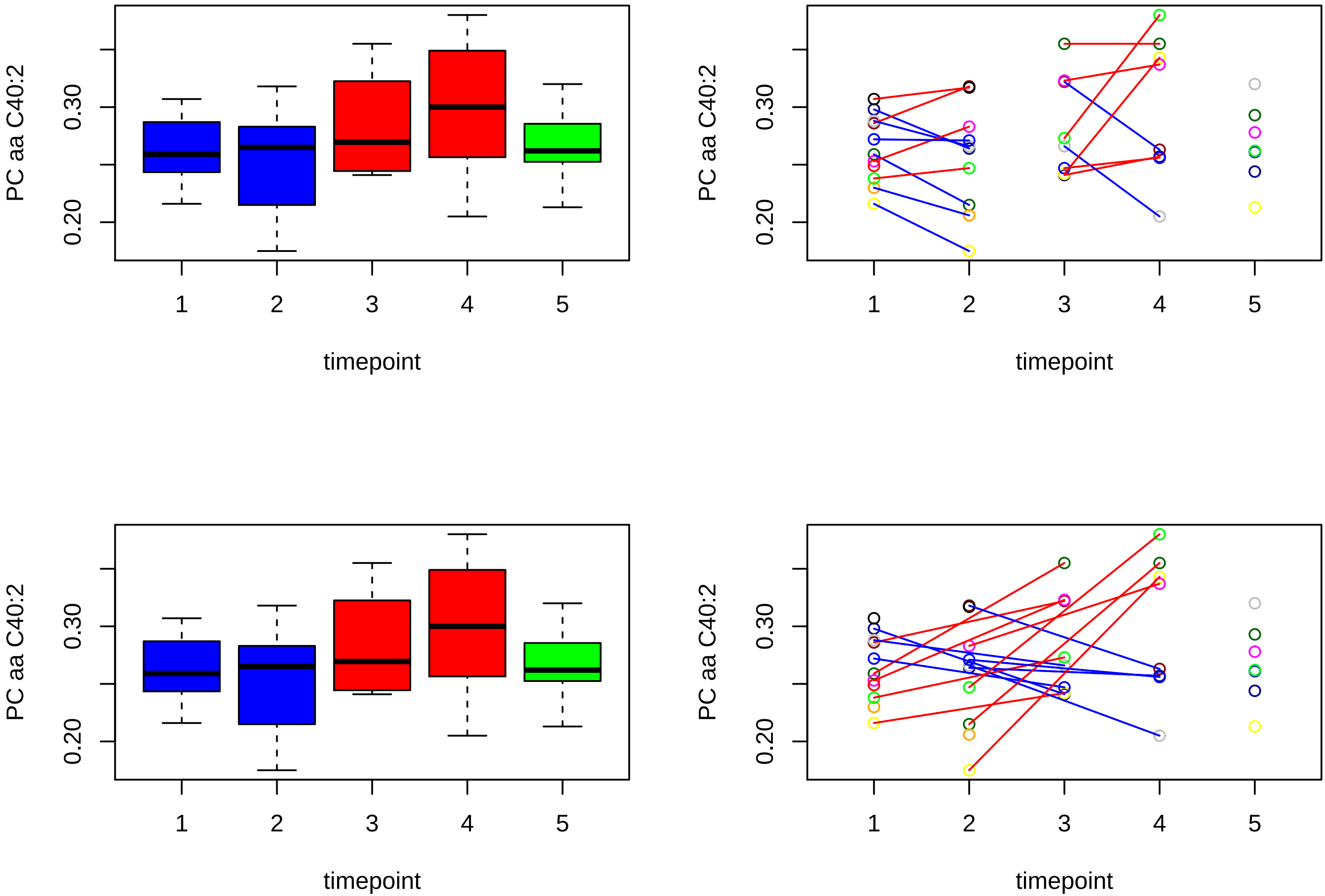
PC aa C40:2
0.20
0.30
1
2
3
4
5
timepoint

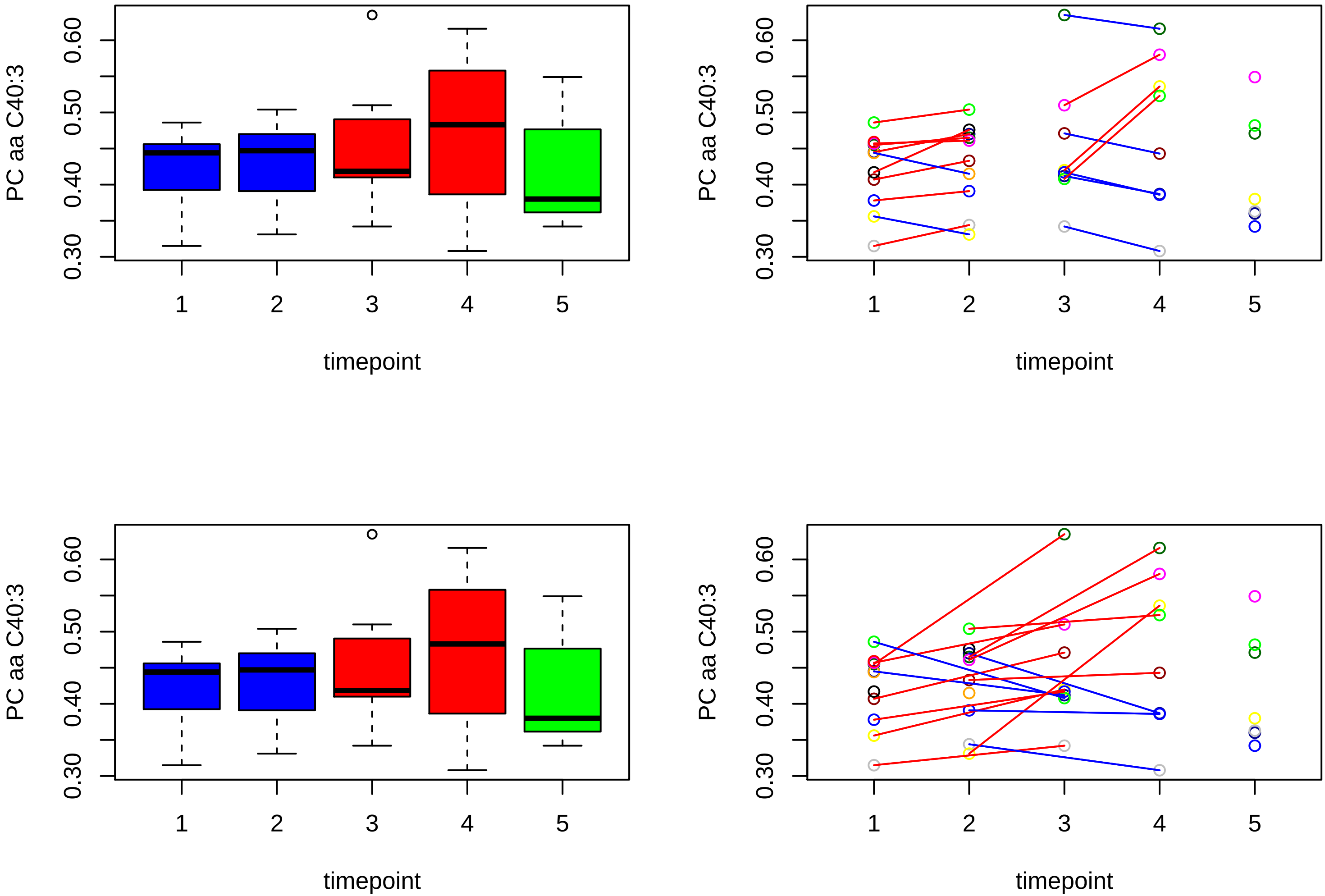
PC aa C40:3
0.30
0.40
0.50
0.60
1
2
3
4
5
timepoint

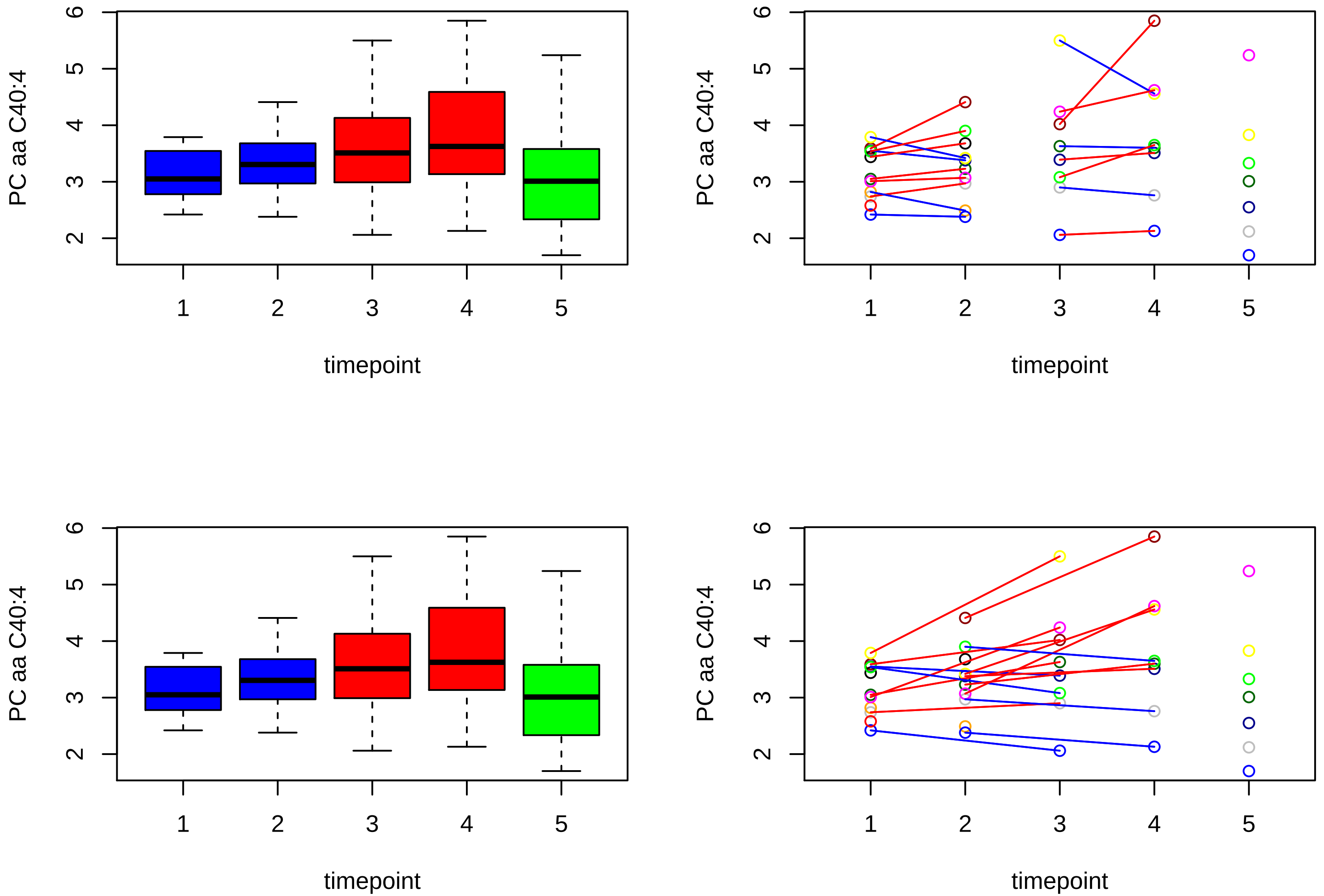
PC aa C40:4
timepoint
PC aa C40:4
timepoint
PC aa C40:4
timepoint
PC aa C40:4
timepoint

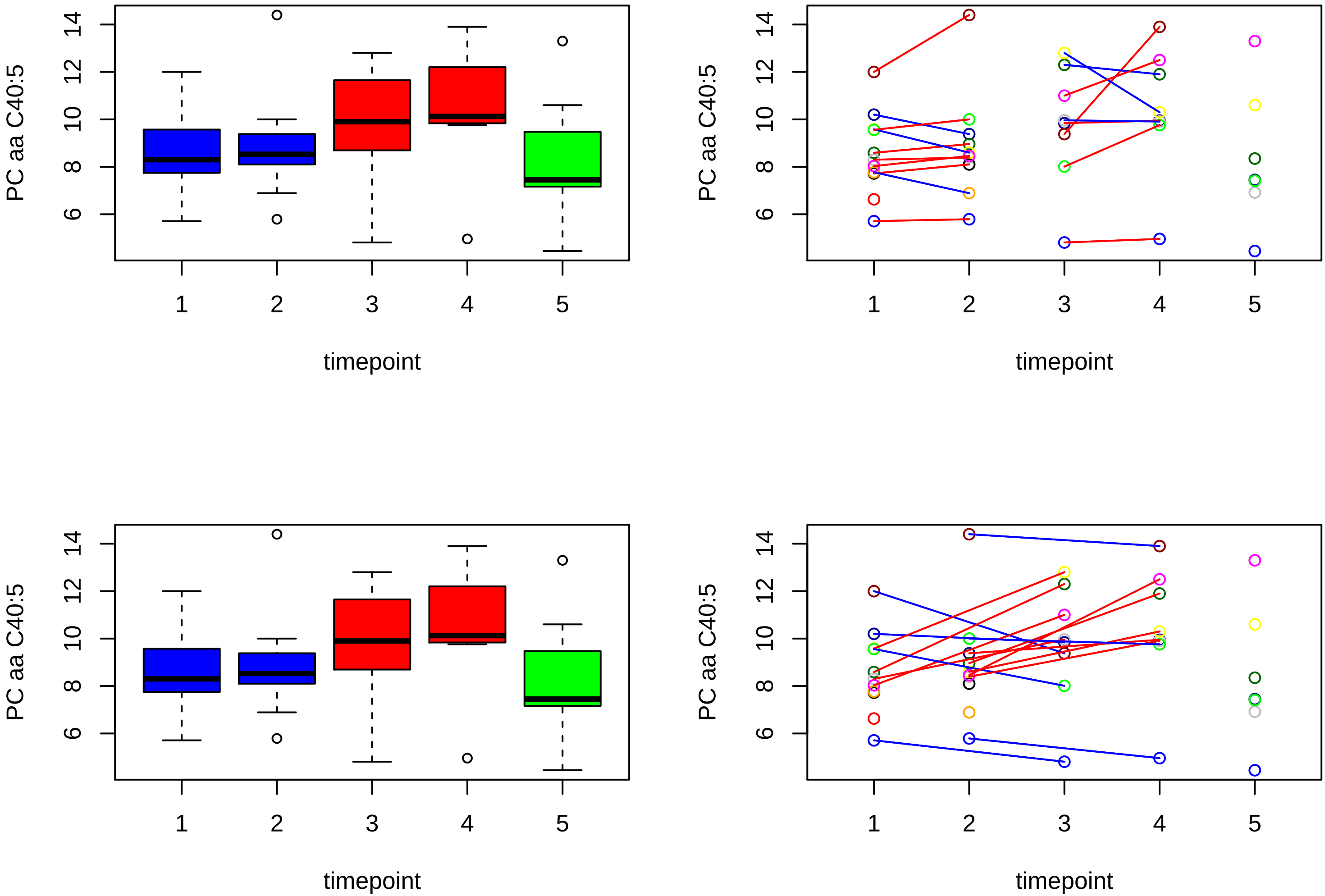

PC aa C40:5
timepoint
1
2
3
4
5
6
8
10
12
14

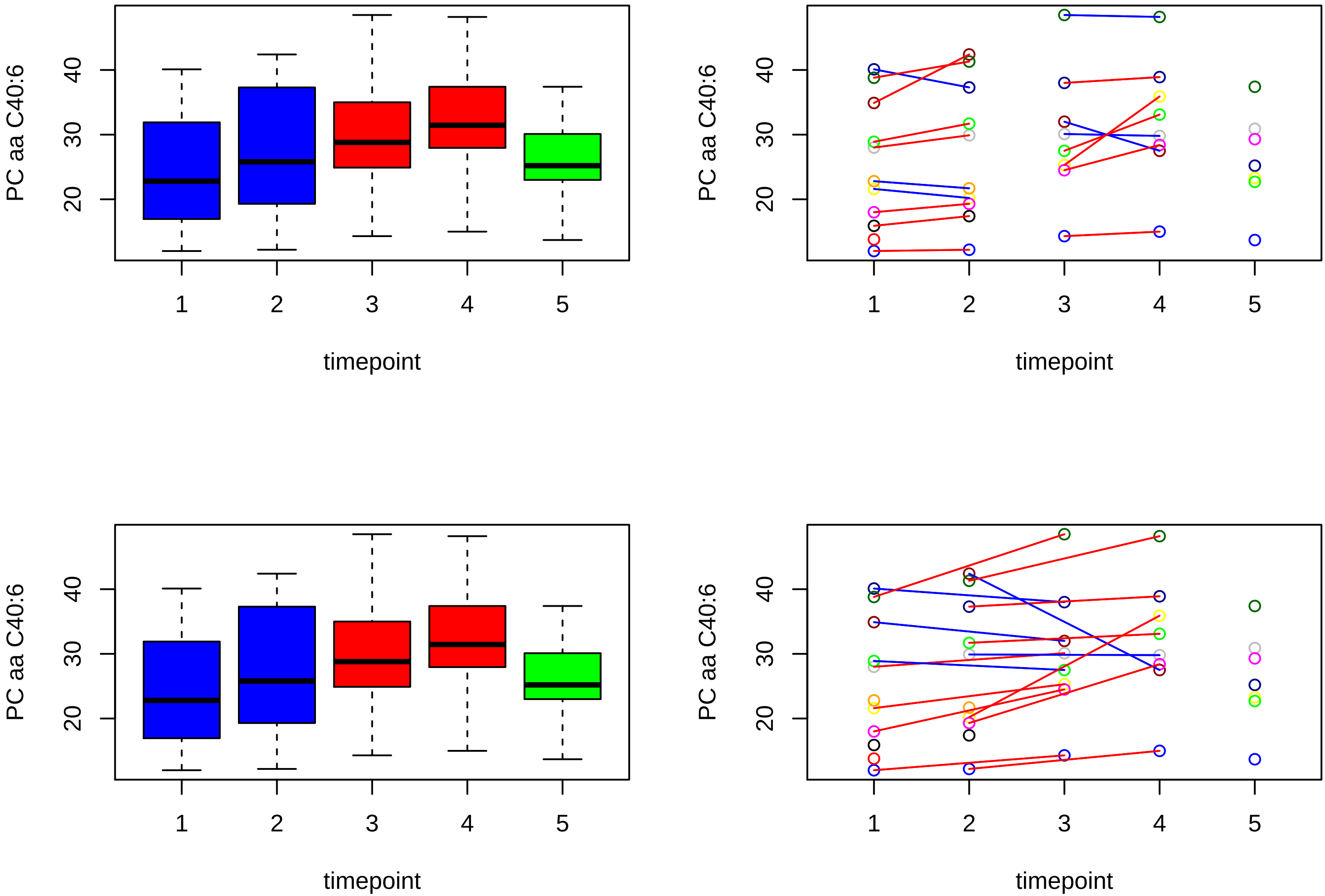

PC aa C40:6
20
30
40
1
2
3
4
5
timepoint

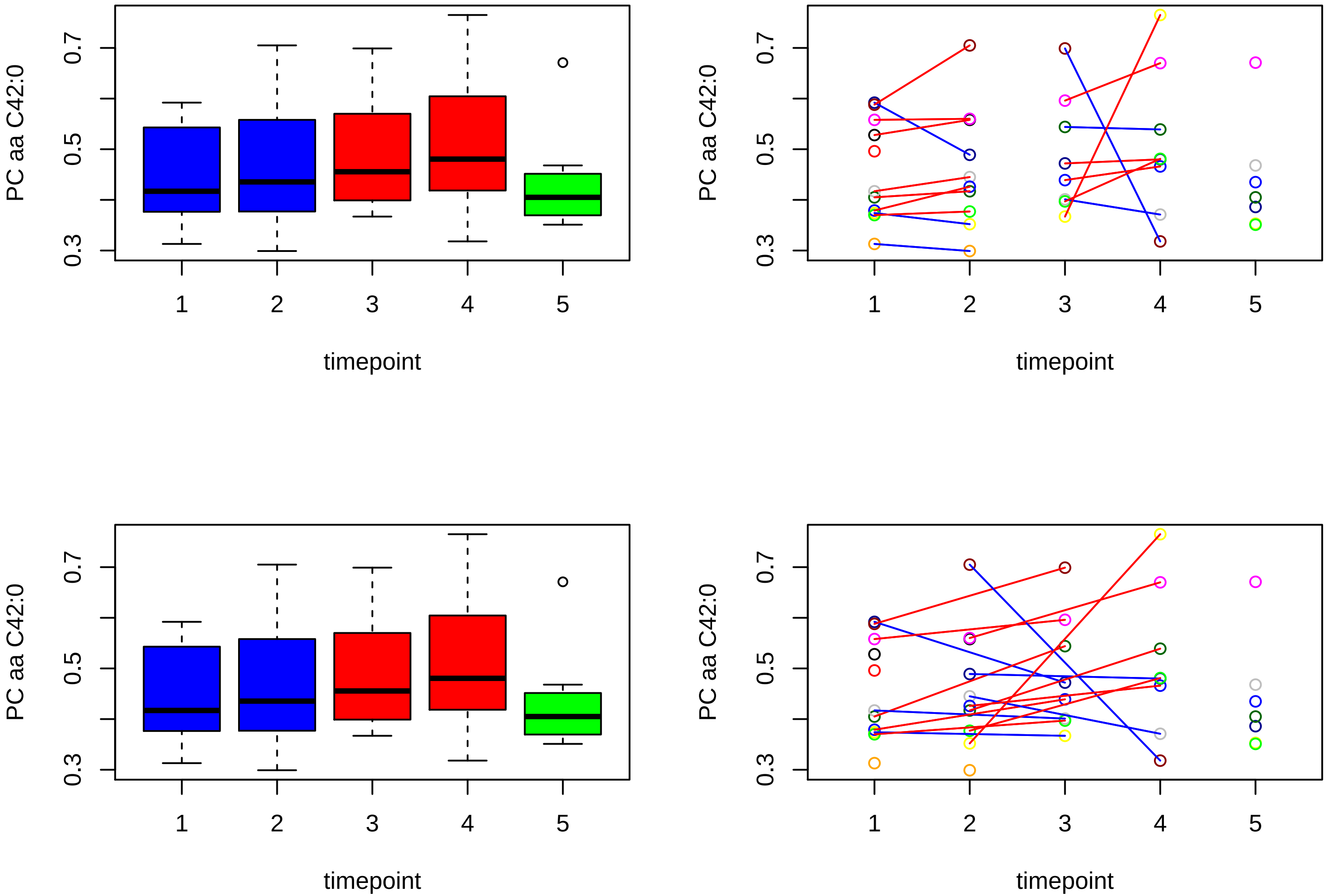
PC aa C42:0
0.3
0.5
0.7
1
2
3
4
5
timepoint

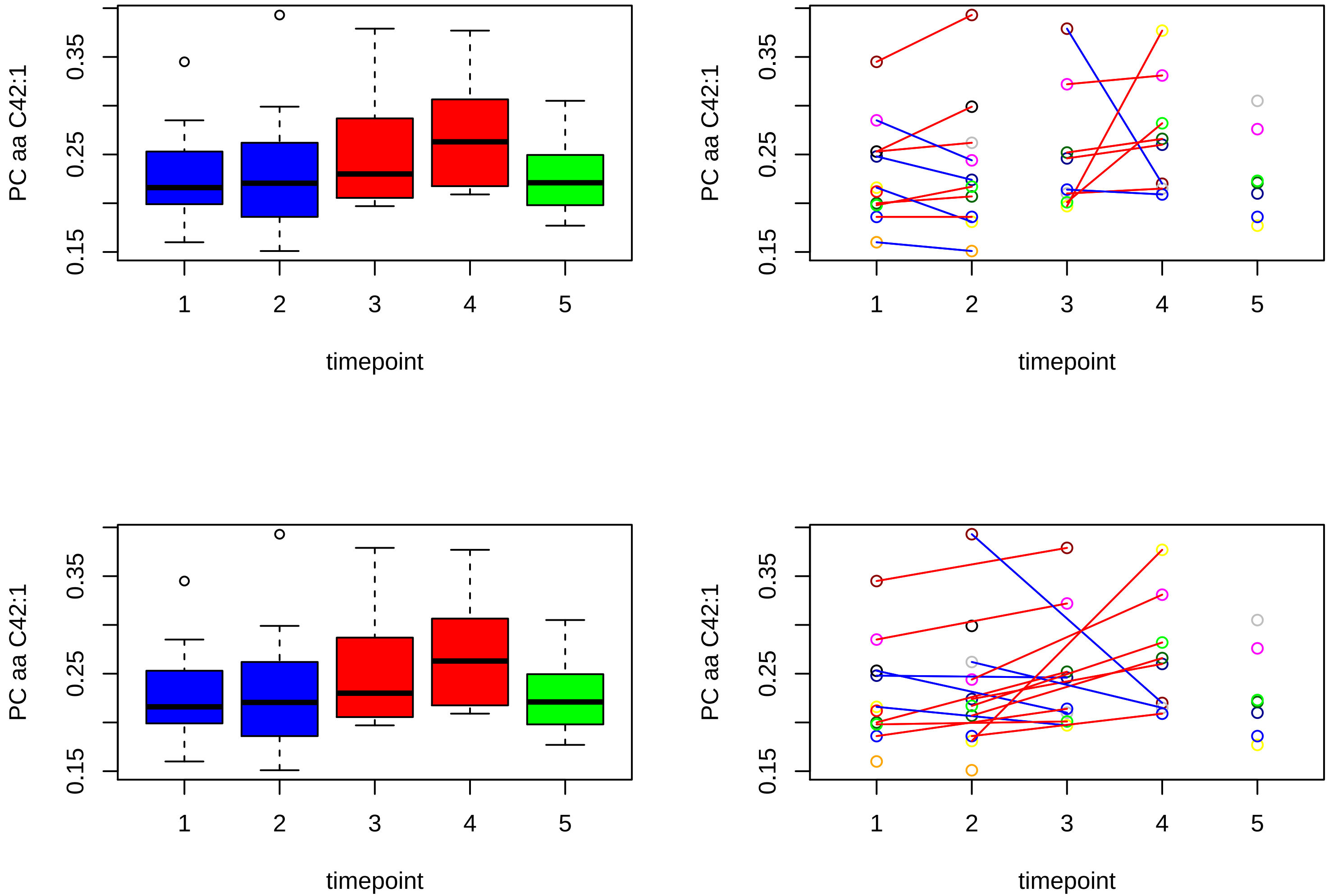
PC aa C42:1
0.15
0.25
0.35
1
2
3
4
5
timepoint

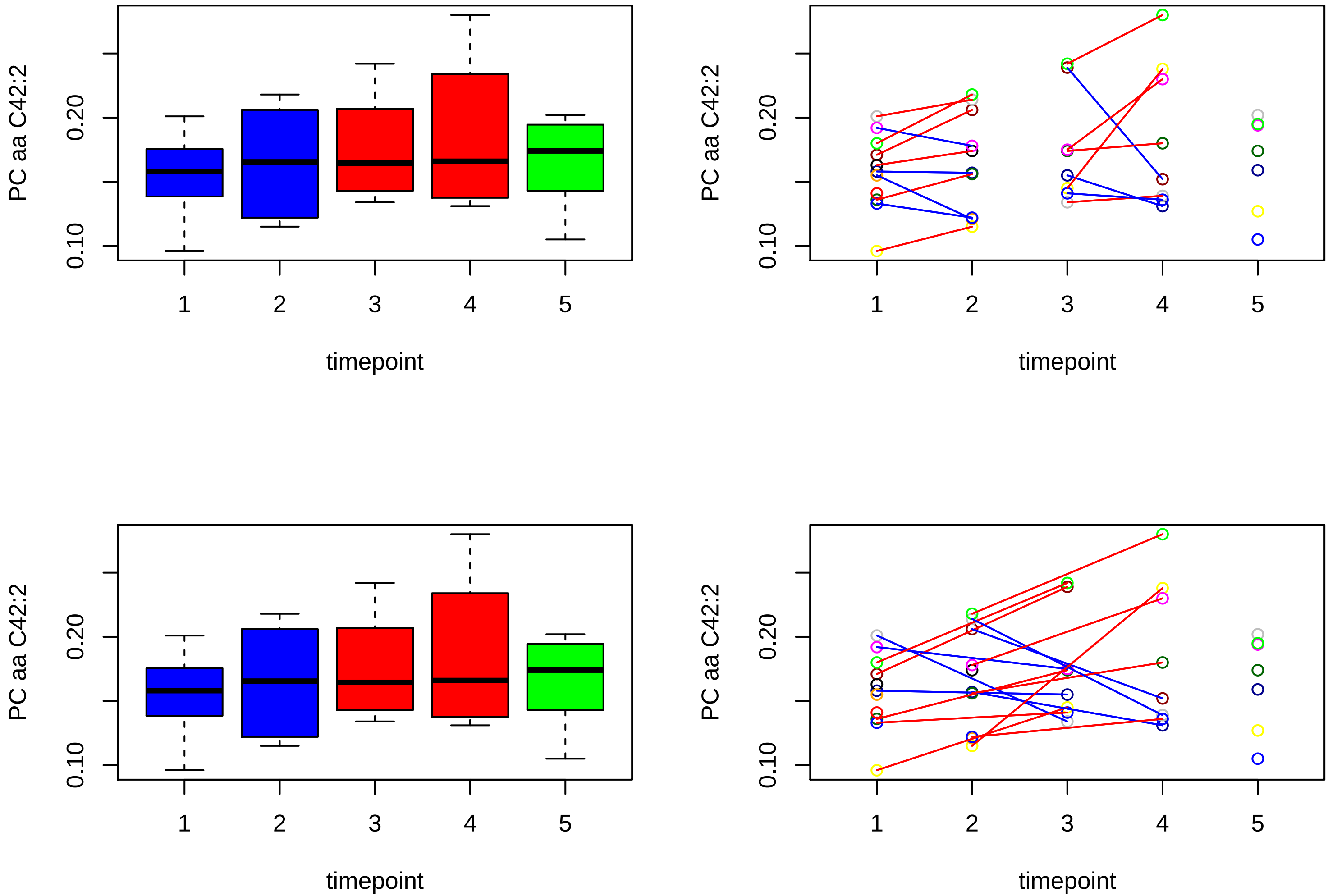
PC aa C42:2
0.10
0.20
1
2
3
4
5
timepoint

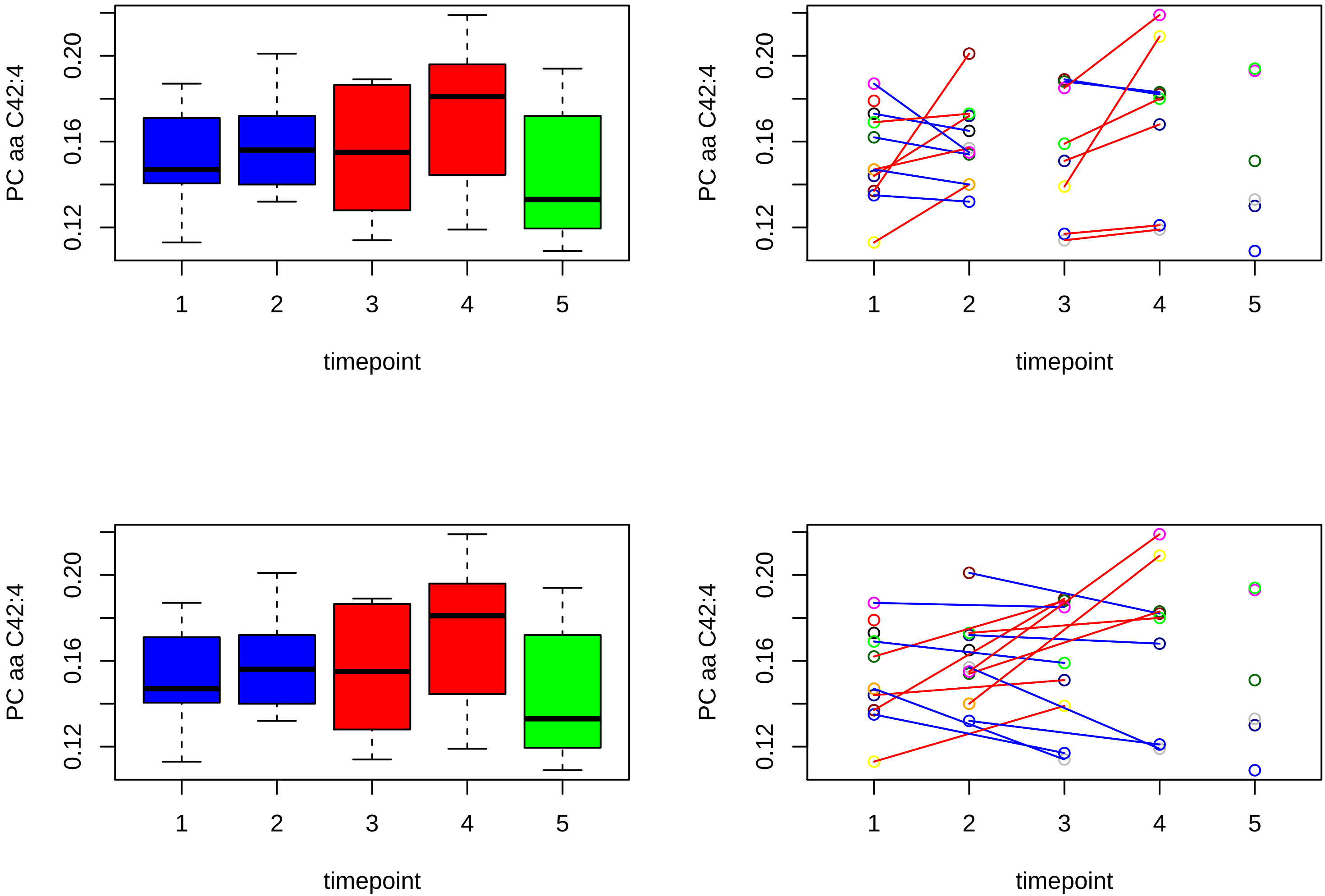

PC aa C42:4
0.12
0.16
0.20
1
2
3
4
5
timepoint

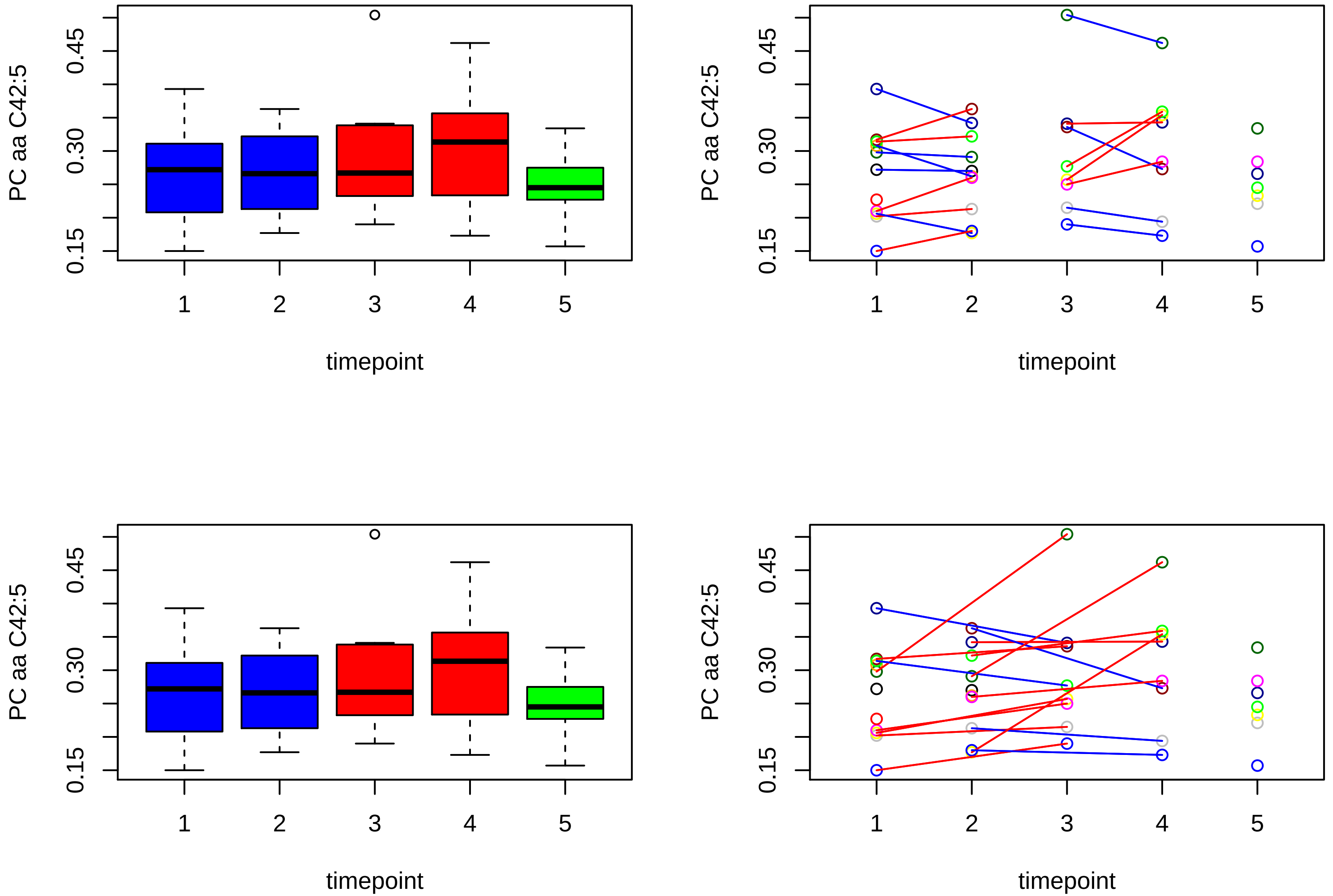
PC aa C42:5
0.15
0.30
0.45
1
2
3
4
5
timepoint

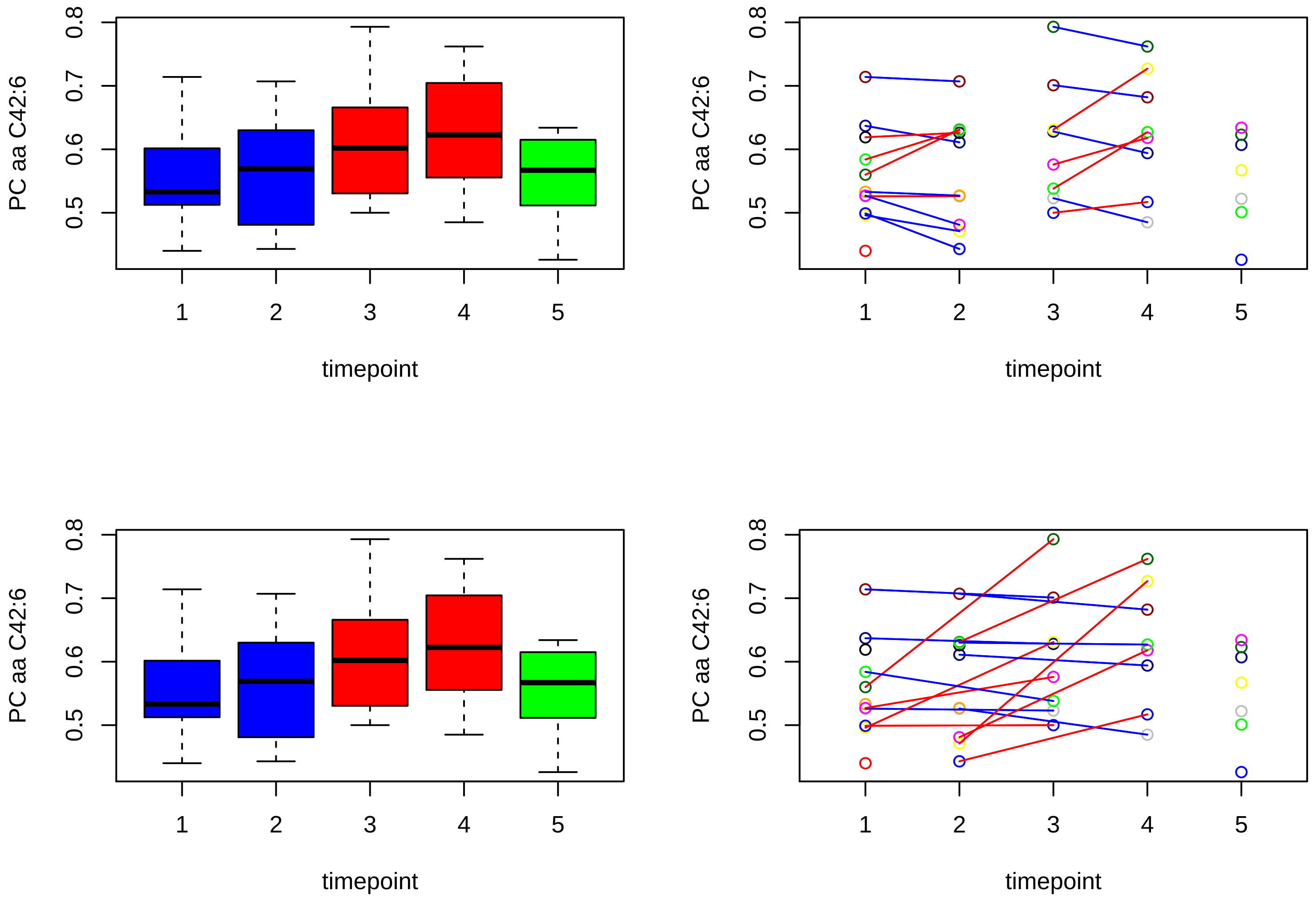

PC aa C42:6
0.5
0.6
0.7
0.8
1
2
3
4
5
timepoint

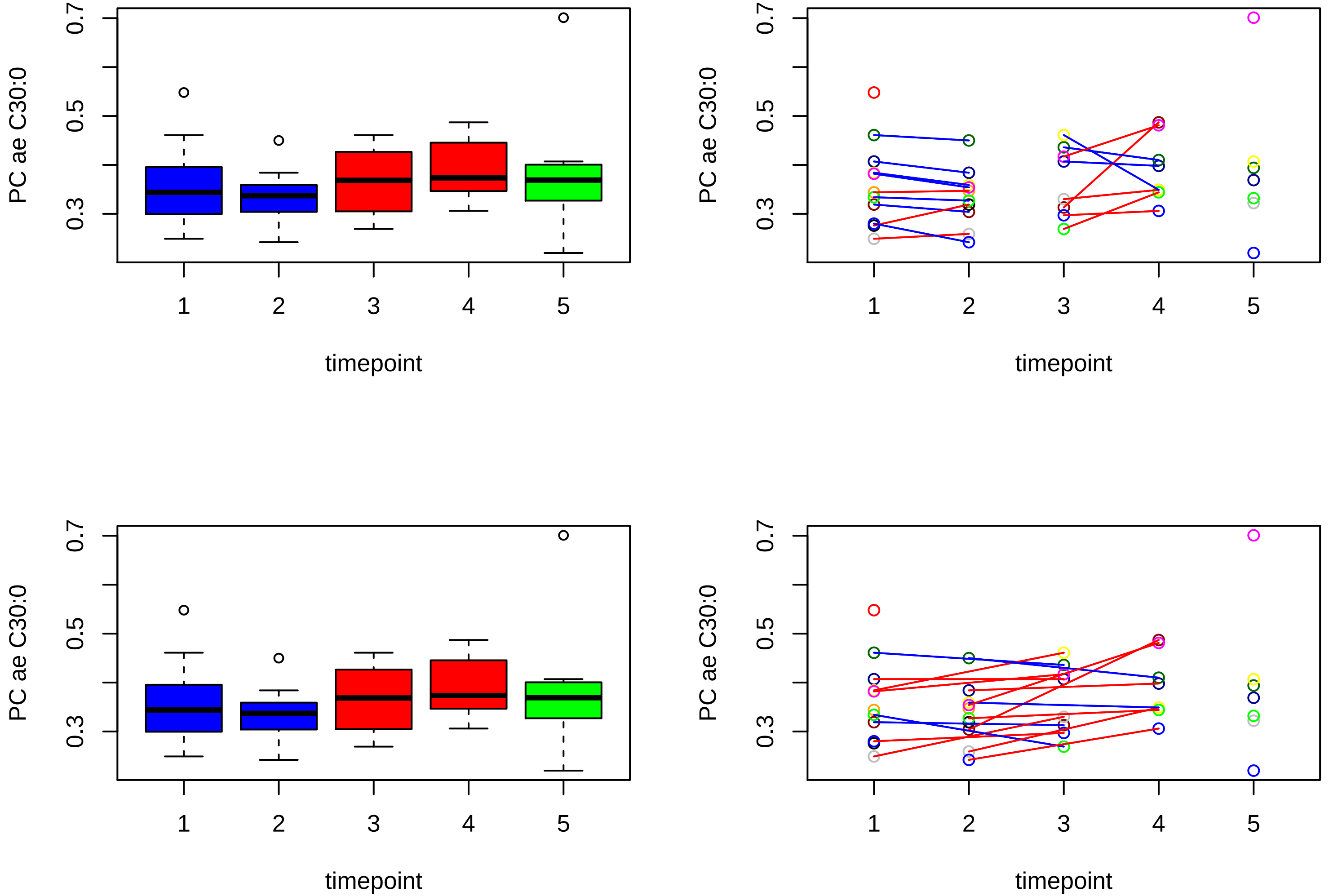
PC ae C30:0
0.3
0.5
0.7
1
2
3
4
5
timepoint
PC ae C30:0
0.3
0.5
0.7
1
2
3
4
5
timepoint
PC ae C30:0
0.3
0.5
0.7
1
2
3
4
5
timepoint
PC ae C30:0
0.3
0.5
0.7
1
2
3
4
5
timepoint

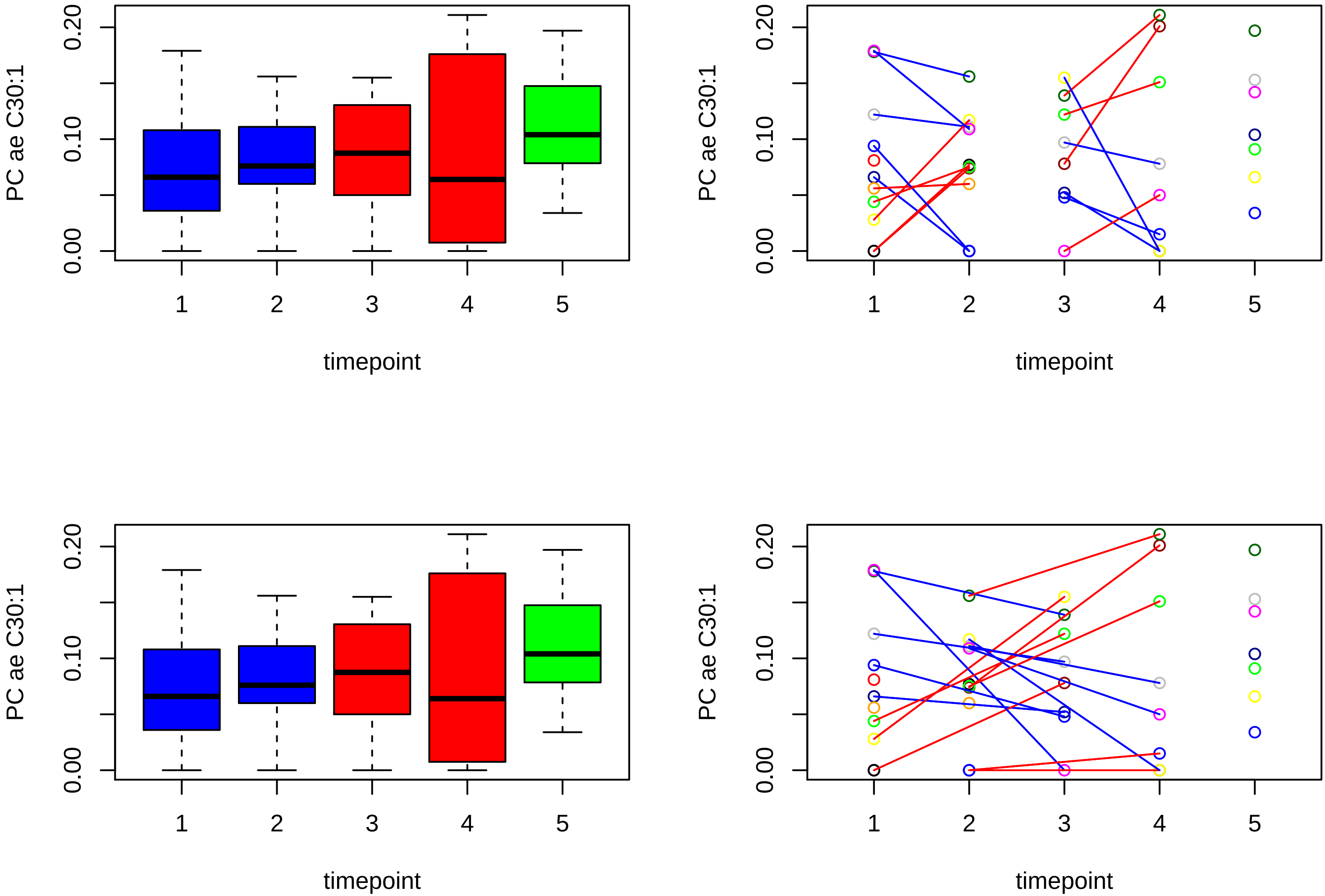
PC ae C30:1
0.00
0.10
0.20
1
2
3
4
5
timepoint
PC ae C30:1
0.00
0.10
0.20
1
2
3
4
5
timepoint
PC ae C30:1
0.00
0.10
0.20
1
2
3
4
5
timepoint
PC ae C30:1
0.00
0.10
0.20
1
2
3
4
5
timepoint

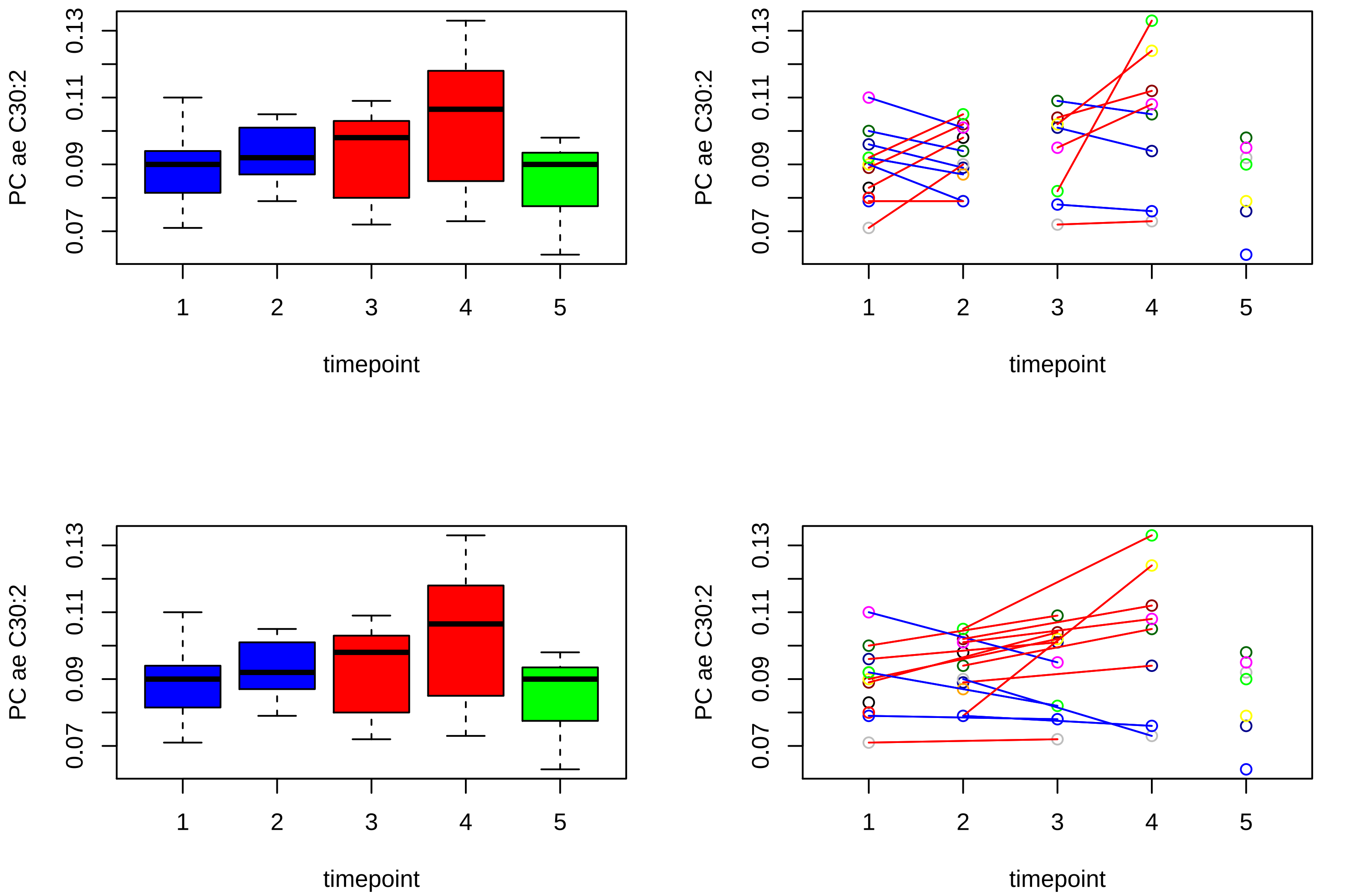

PC ae C30:2
0.07
0.09
0.11
0.13
1
2
3
4
5
timepoint

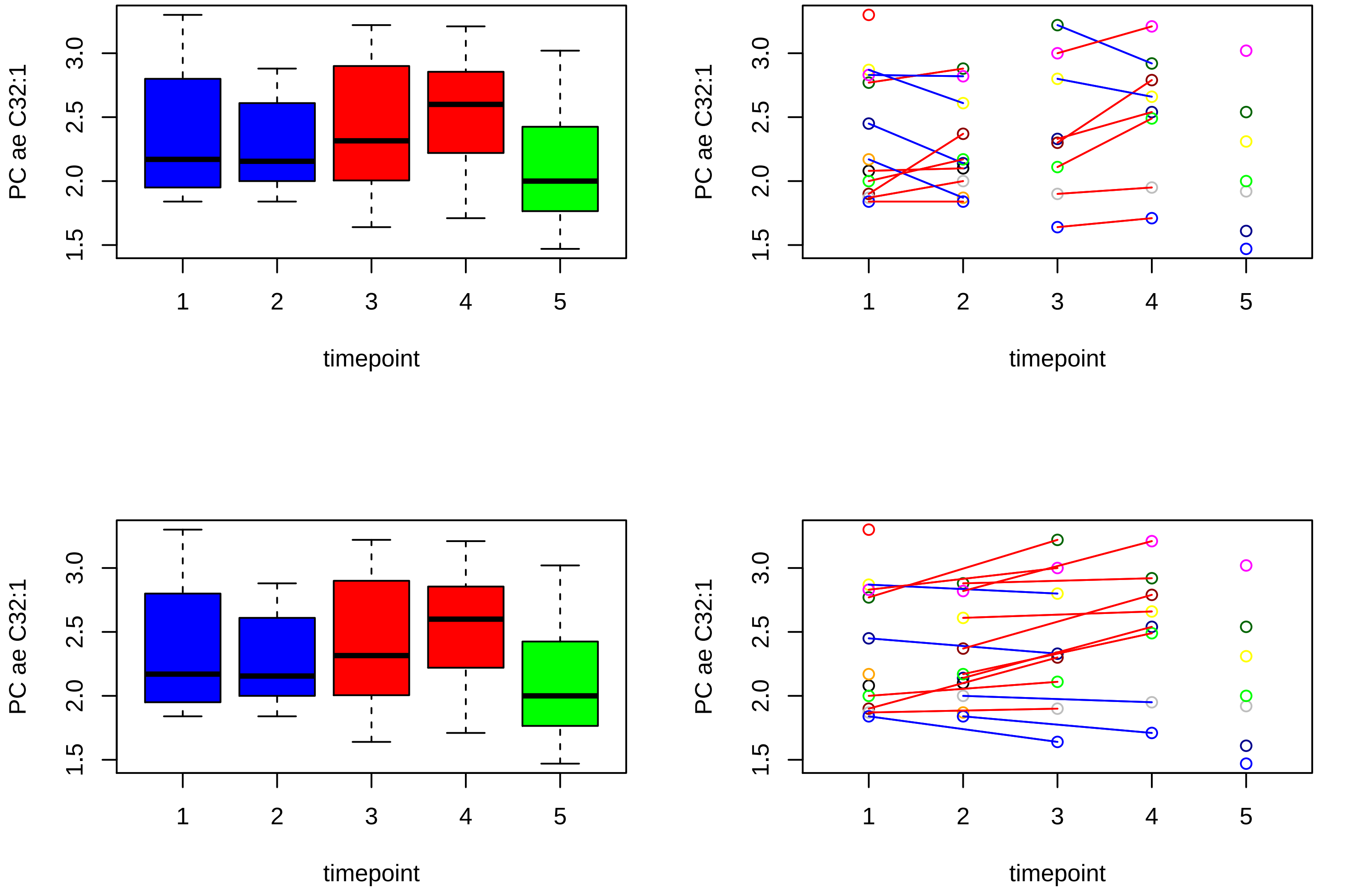
PC ae C32:1
timepoint
1.5
2.0
2.5
3.0
1
2
3
4
5

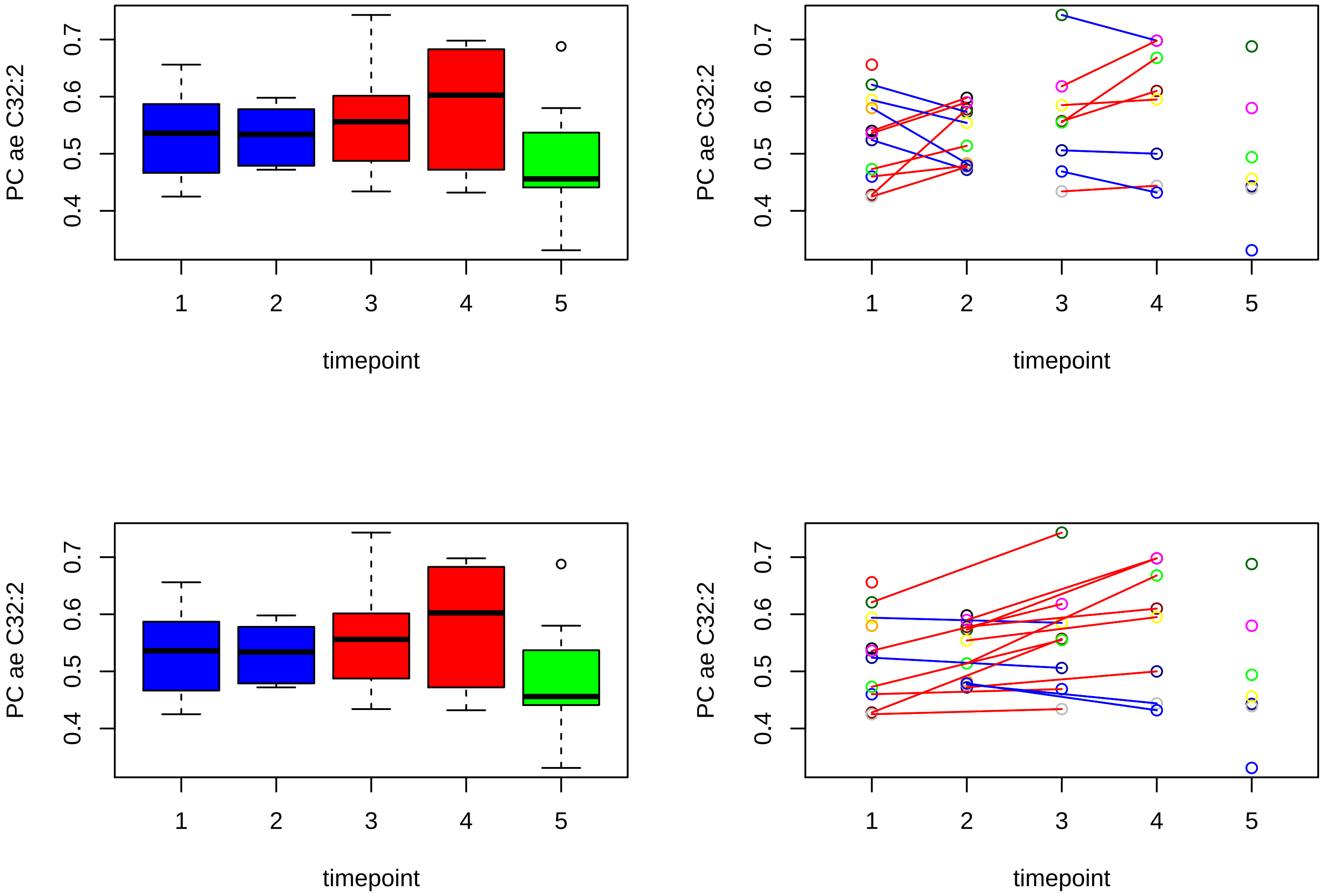
PC ae C32:2
0.4
0.5
0.6
0.7
1
2
3
4
5
timepoint
PC ae C32:2
0.4
0.5
0.6
0.7
1
2
3
4
5
timepoint
PC ae C32:2
0.4
0.5
0.6
0.7
1
2
3
4
5
timepoint
PC ae C32:2
0.4
0.5
0.6
0.7
1
2
3
4
5
timepoint

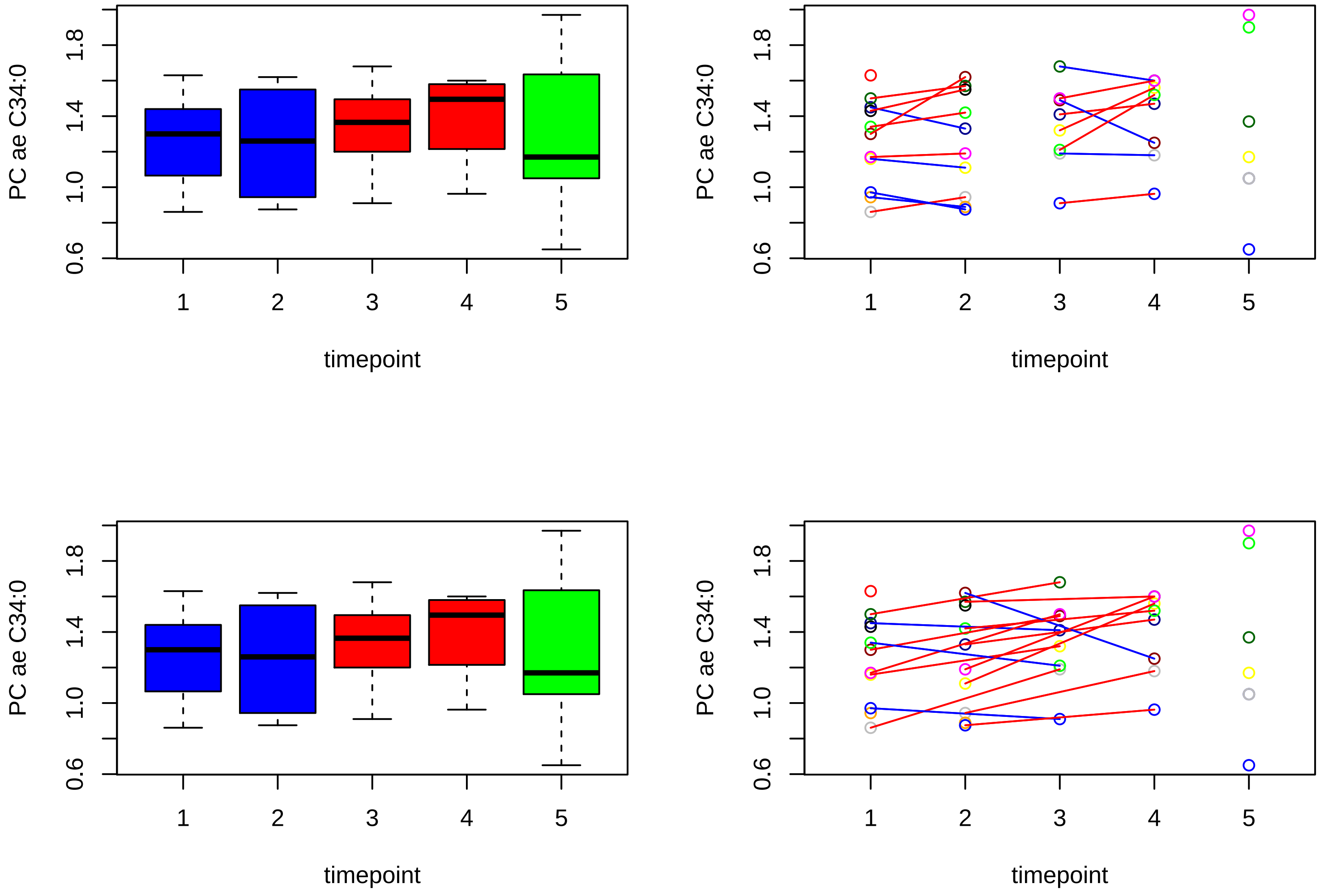
PC ae C34:0
0.6
1.0
1.4
1.8
1
2
3
4
5
timepoint

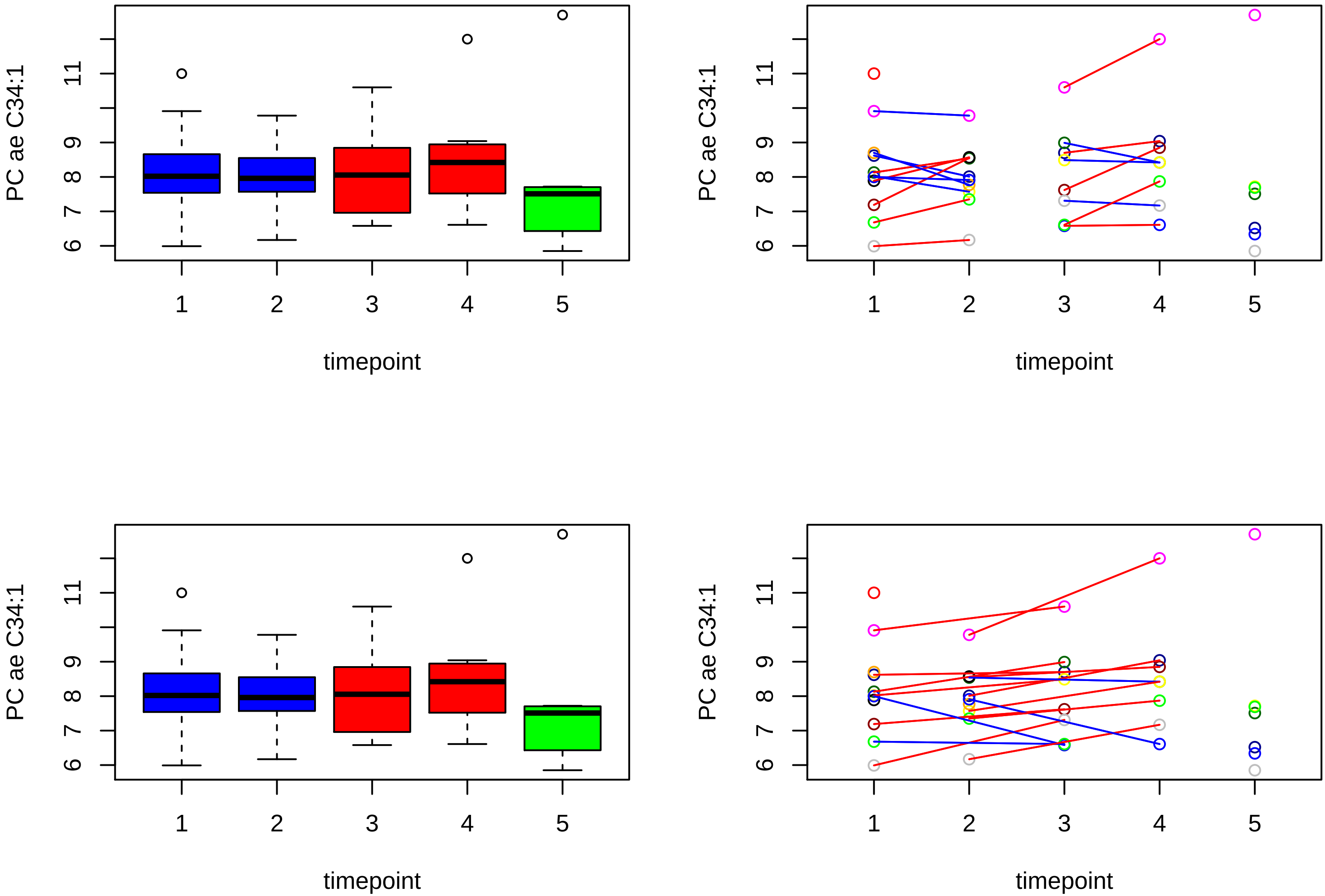
PC ae C34:1
timepoint
PC ae C34:1
timepoint
PC ae C34:1
timepoint
PC ae C34:1
timepoint

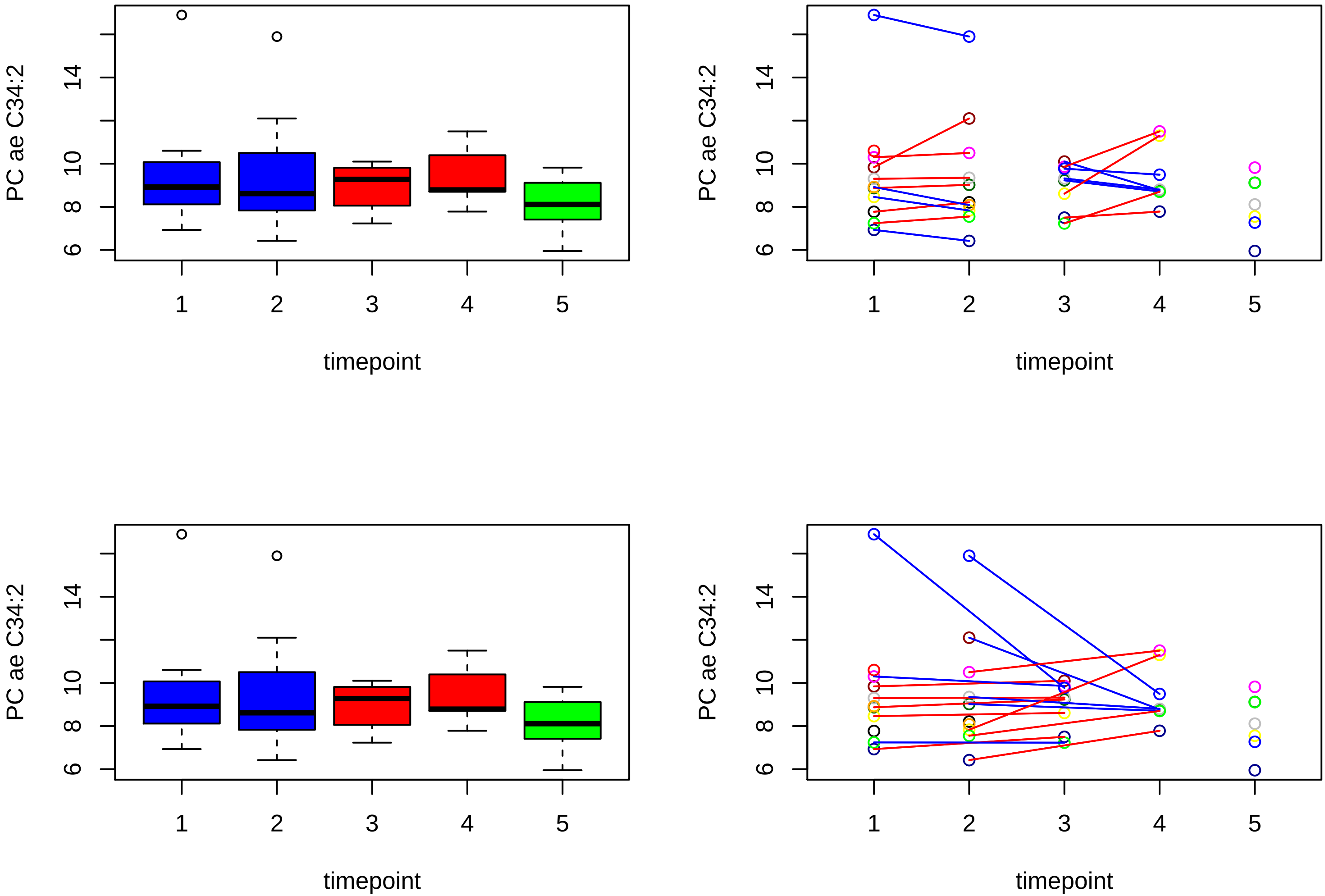
PC ae C34:2
6
8
10
14
1
2
3
4
5
timepoint

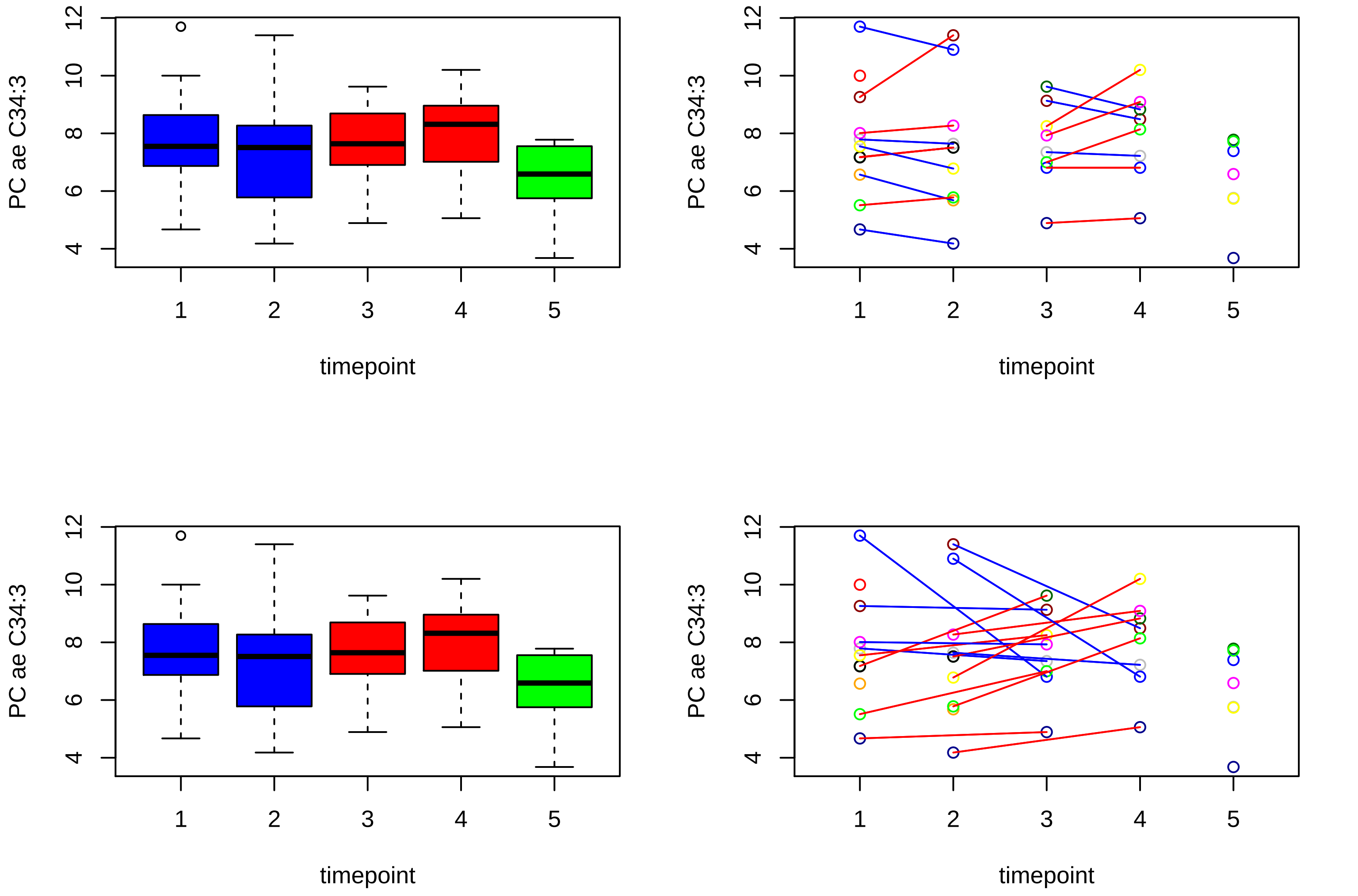
PC ae C34:3
timepoint
PC ae C34:3
timepoint
PC ae C34:3
timepoint
PC ae C34:3
timepoint

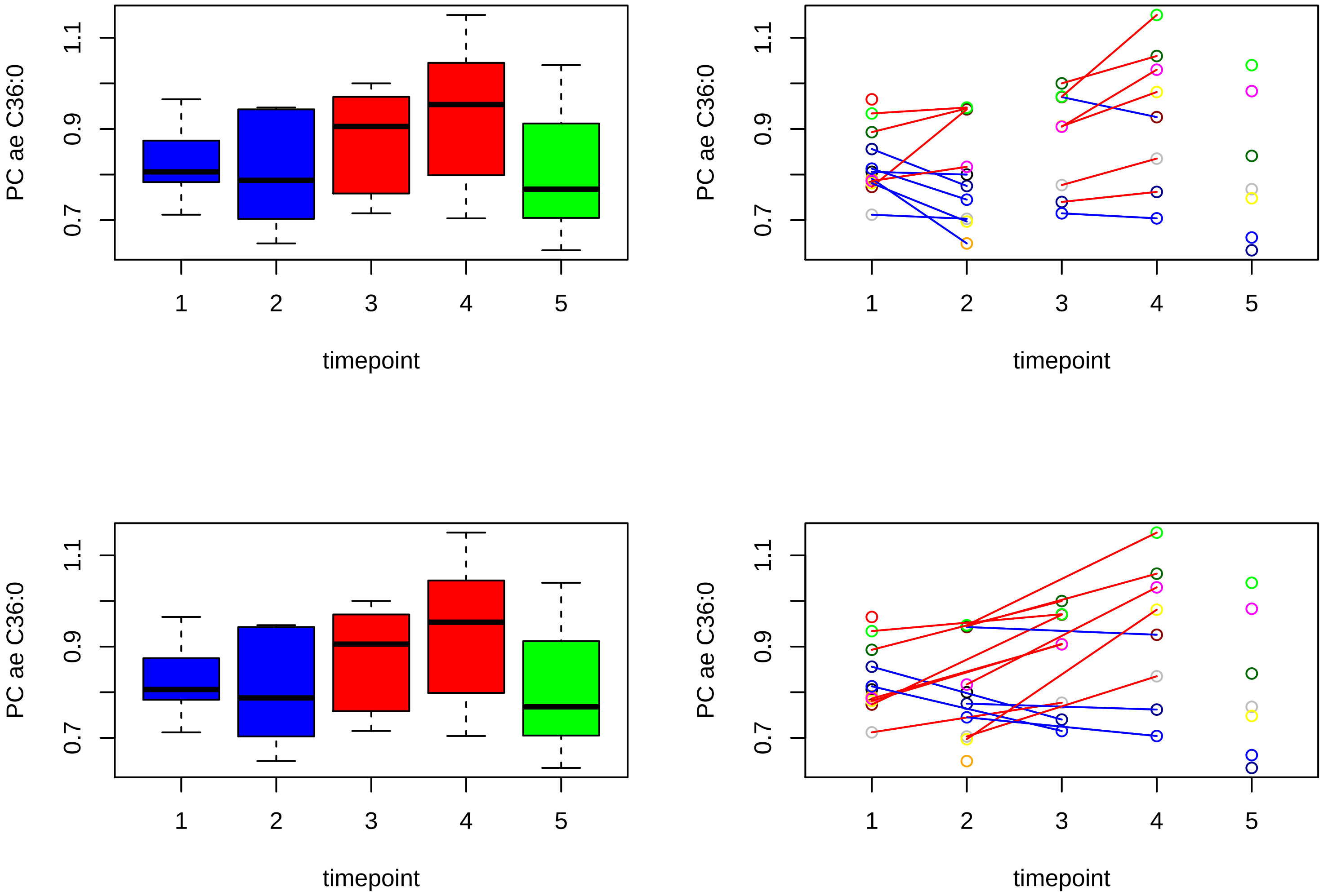
PC ae C36:0
0.7
0.9
1.1
1
2
3
4
5
timepoint

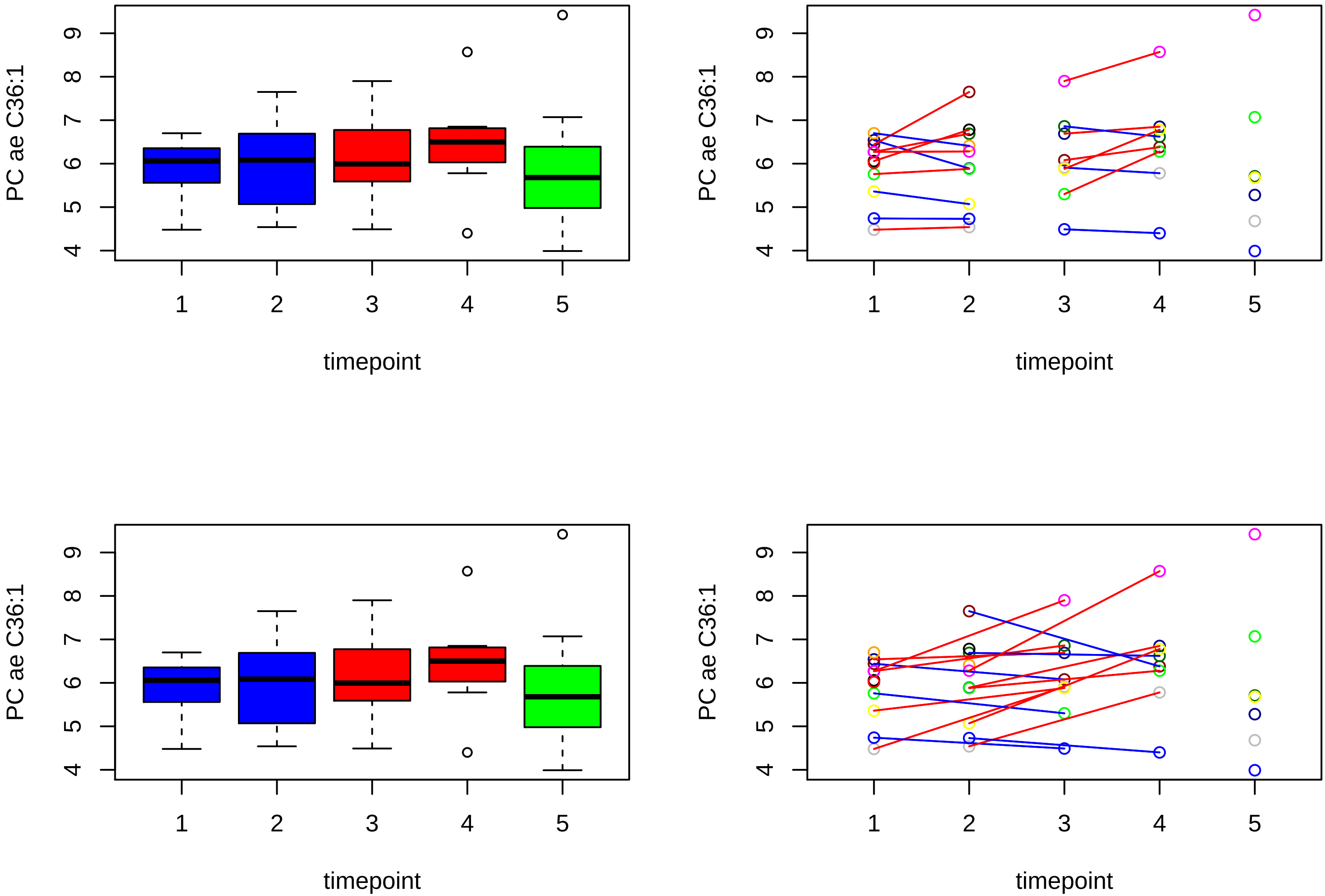
PC ae C36:1
timepoint
PC ae C36:1
timepoint
PC ae C36:1
timepoint
PC ae C36:1
timepoint

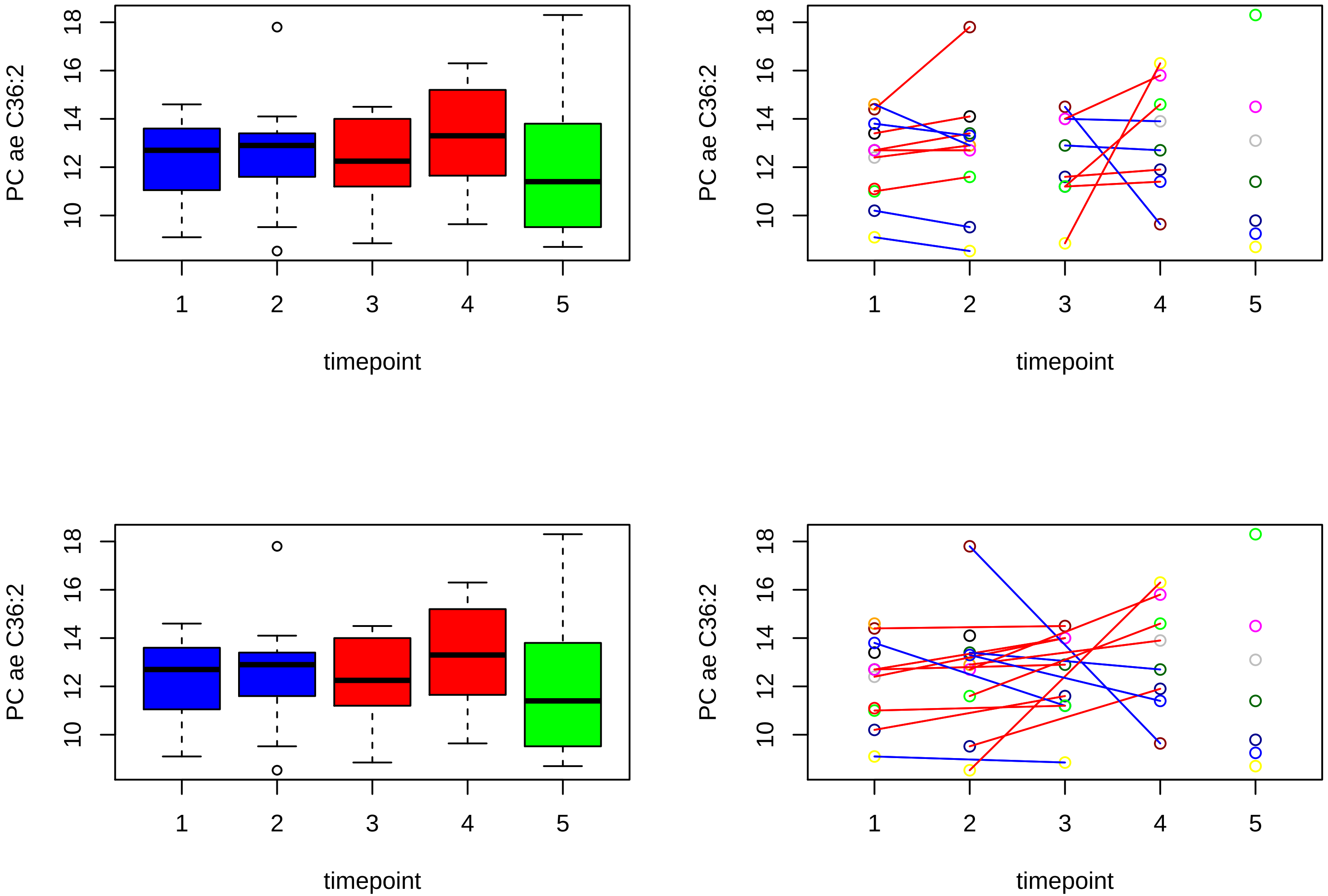

PC ae C36:2
10
12
14
16
18
1
2
3
4
5
timepoint
PC ae C36:2
10
12
14
16
18
1
2
3
4
5
timepoint
PC ae C36:2
10
12
14
16
18
1
2
3
4
5
timepoint
PC ae C36:2
10
12
14
16
18
1
2
3
4
5
timepoint

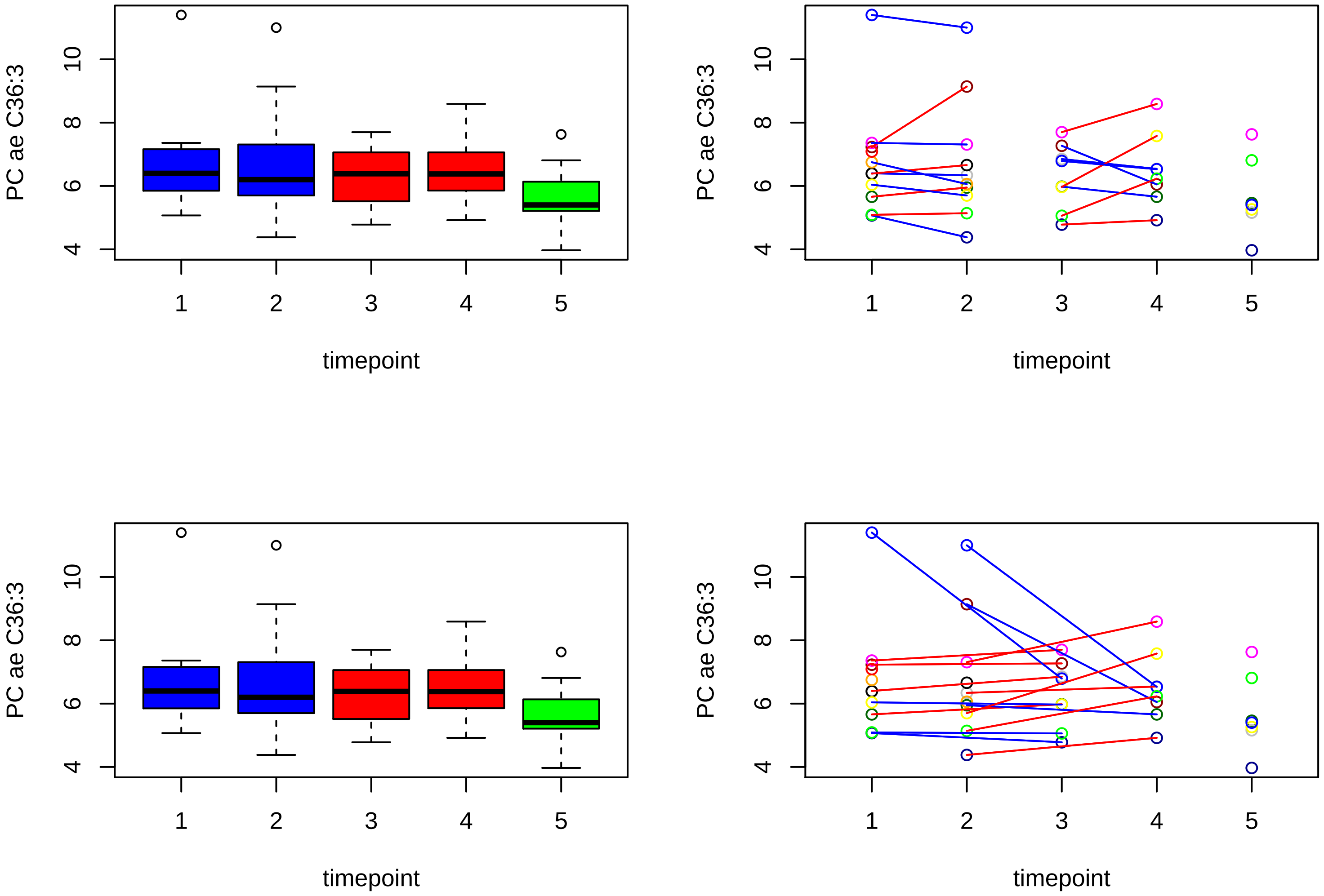
PC ae C36:3
4
6
8
10
1
2
3
4
5
timepoint

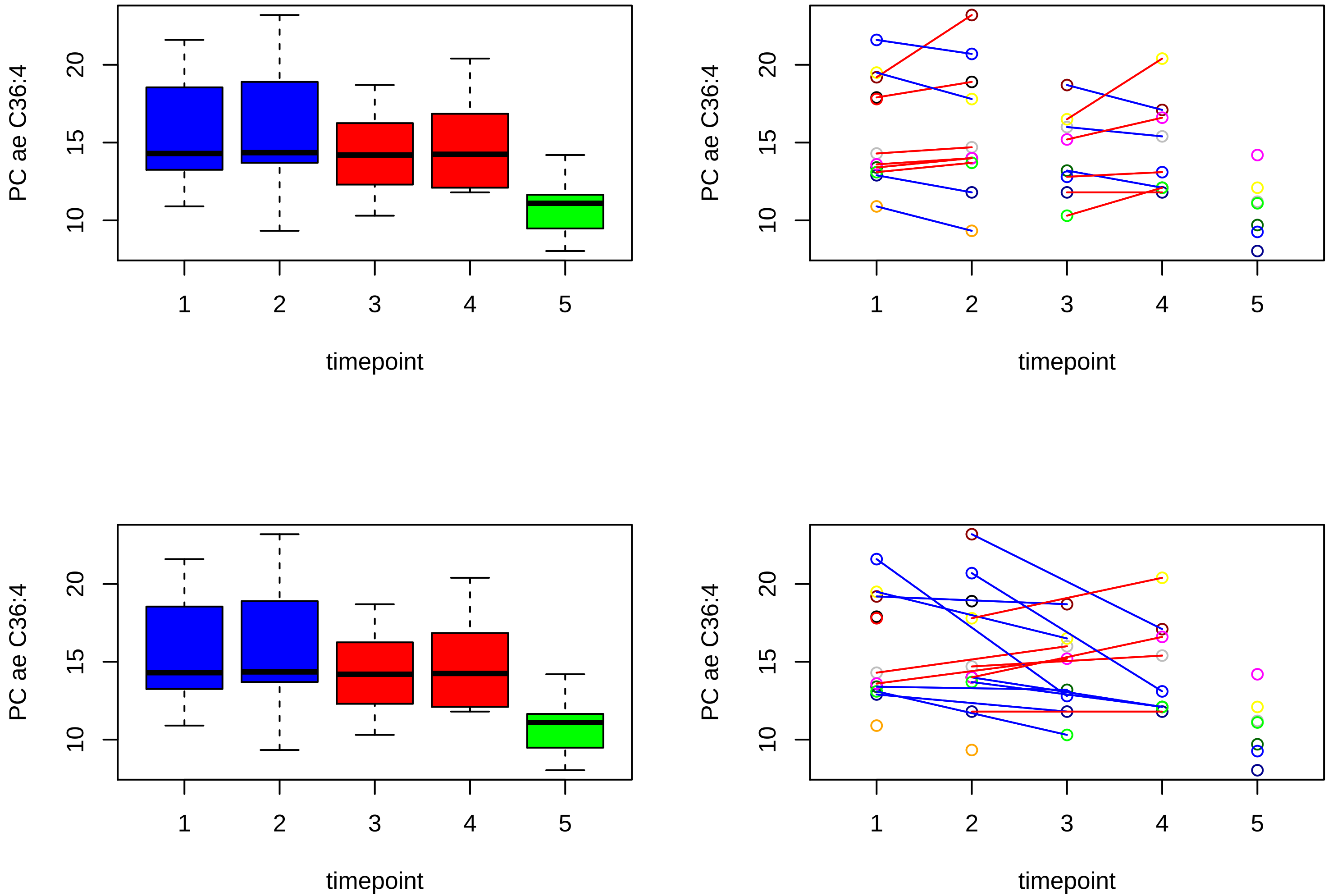
PC ae C36:4
10
15
20
1
2
3
4
5
timepoint
PC ae C36:4
10
15
20
1
2
3
4
5
timepoint
PC ae C36:4
10
15
20
1
2
3
4
5
timepoint
PC ae C36:4
10
15
20
1
2
3
4
5
timepoint

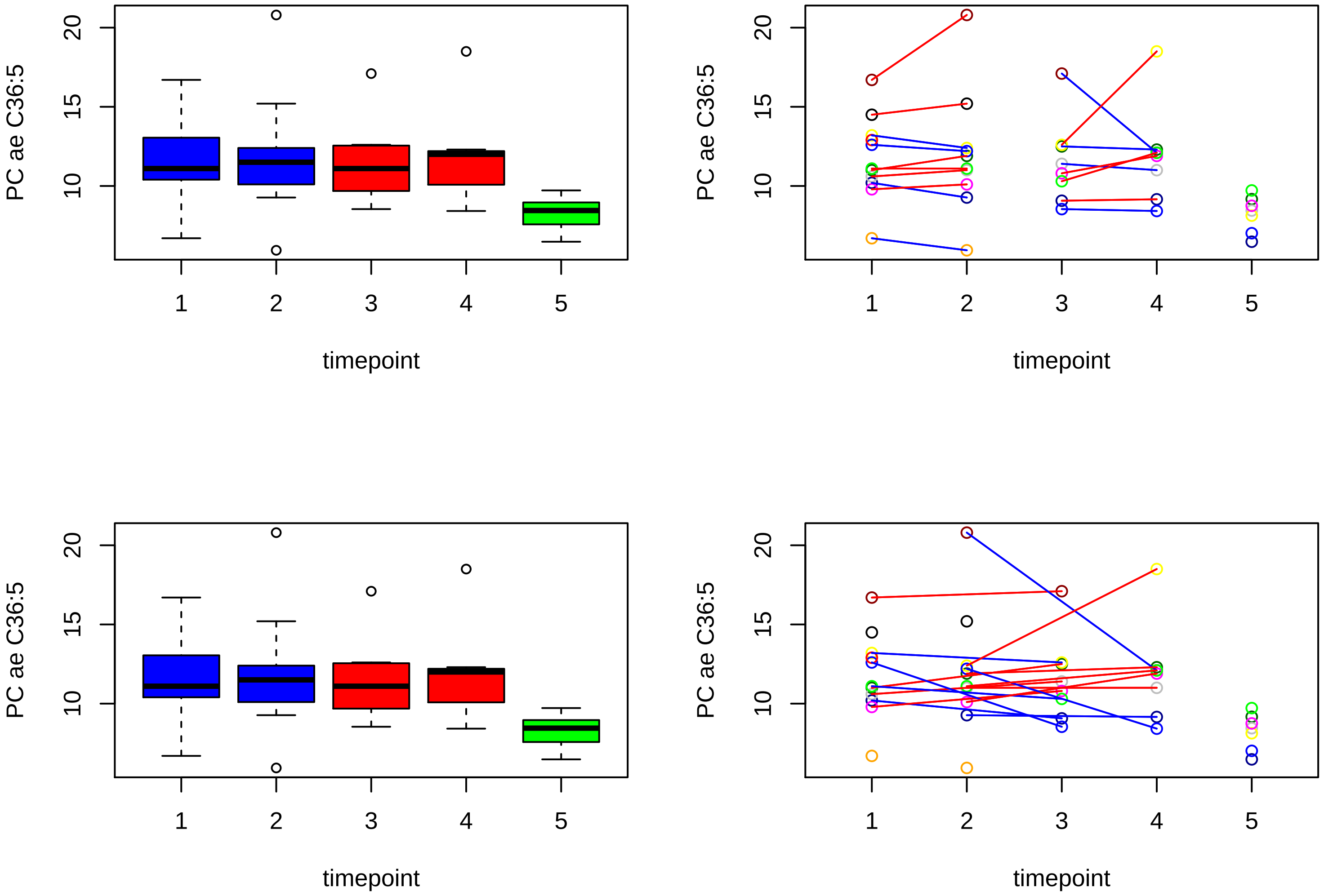
PC ae C36:5
timepoint
PC ae C36:5
timepoint
PC ae C36:5
timepoint
PC ae C36:5
timepoint

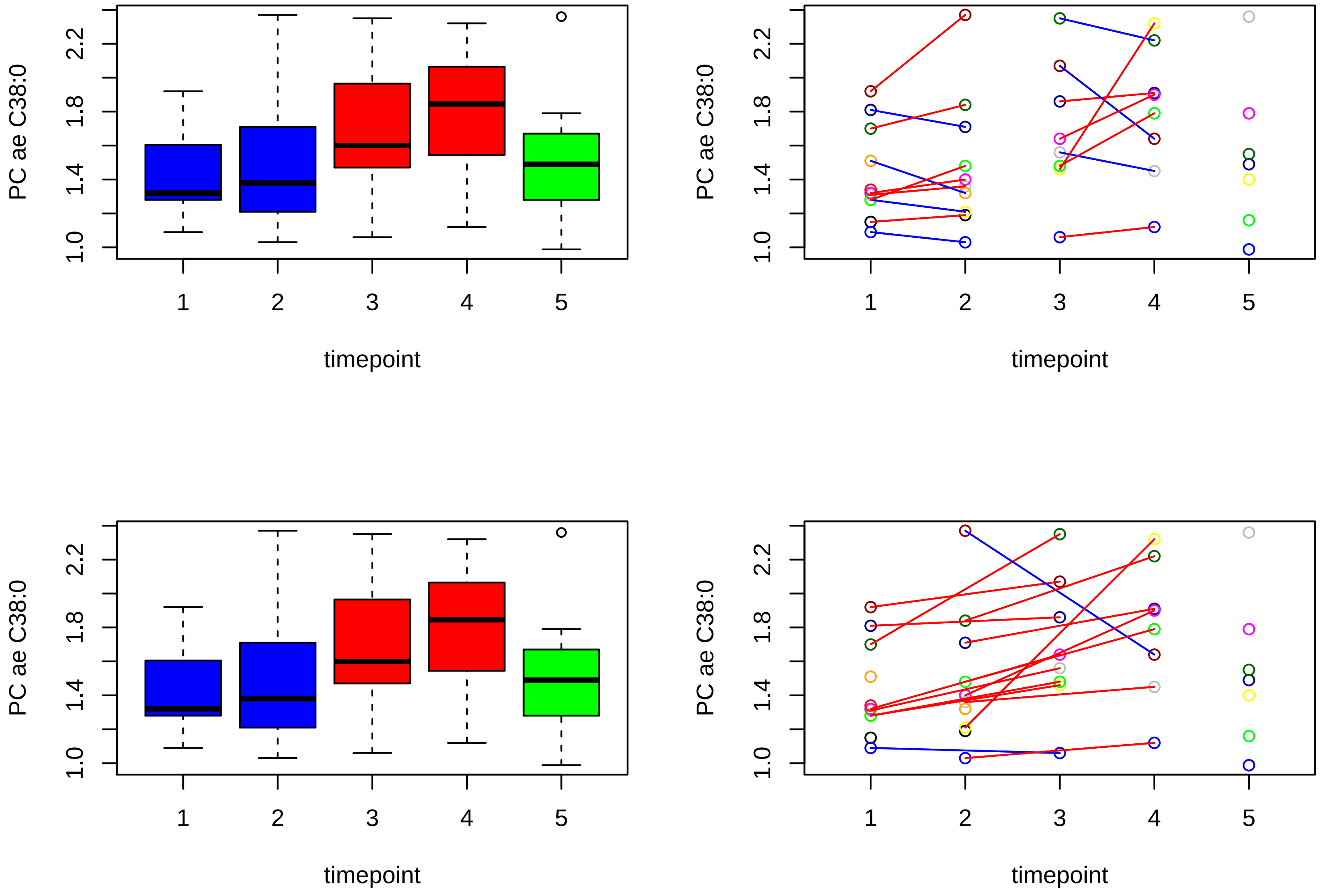

PC ae C38:0
1.0
1.4
1.8
2.2
1
2
3
4
5
timepoint

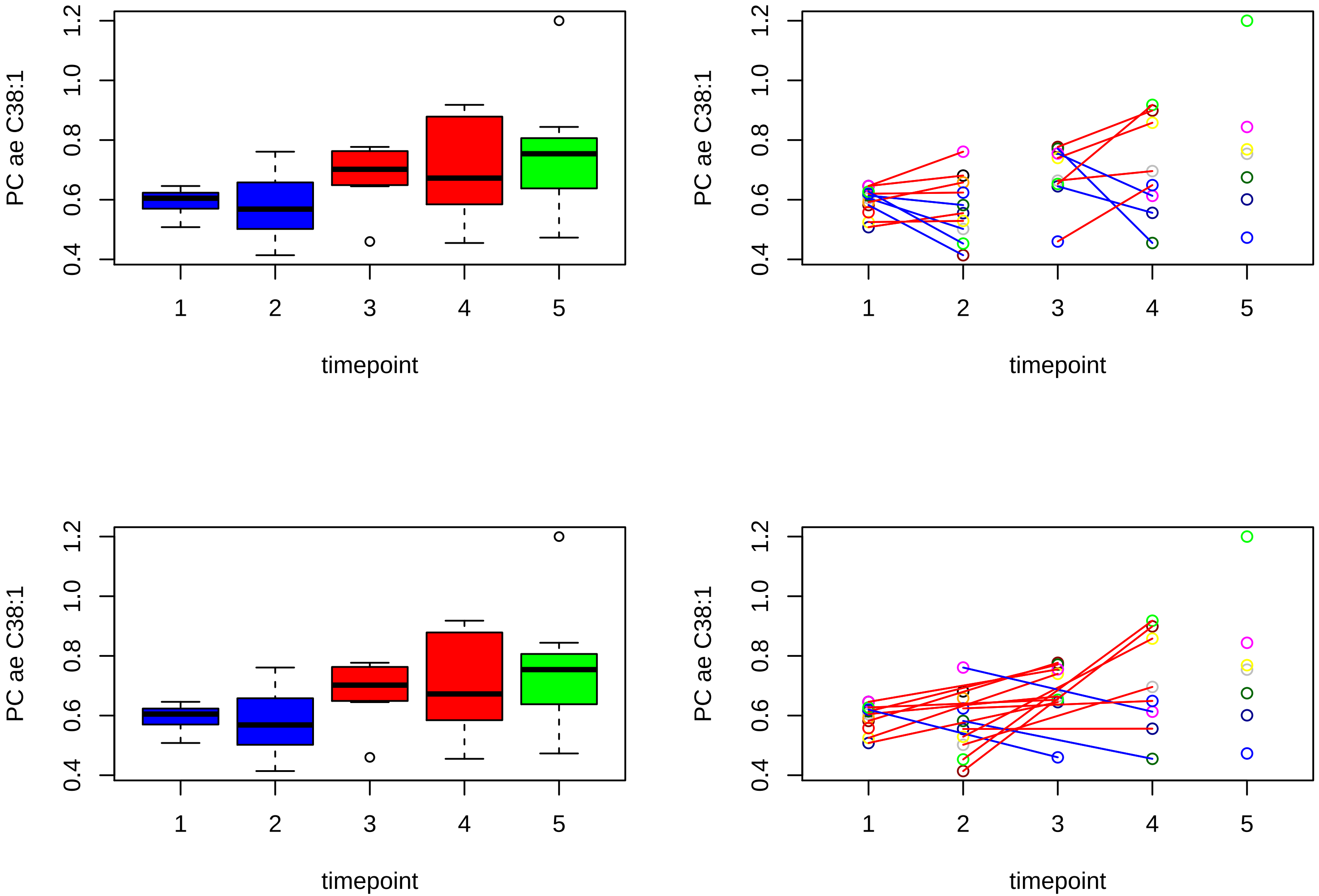
PC ae C38:1
timepoint
1
2
3
4
5
0.4
0.6
0.8
1.0
1.2

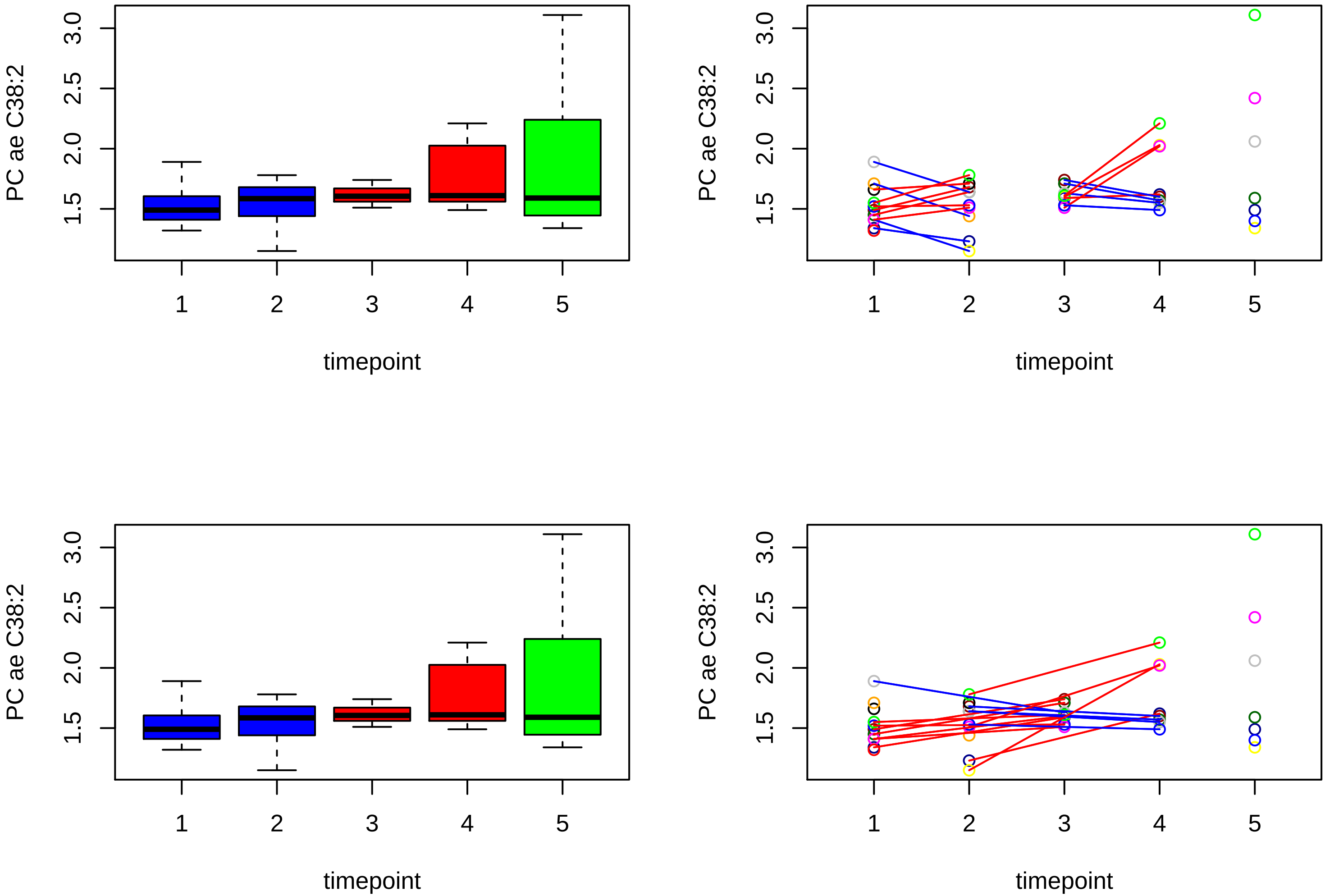

PC ae C38:2
1.5
2.0
2.5
3.0
1
2
3
4
5
timepoint

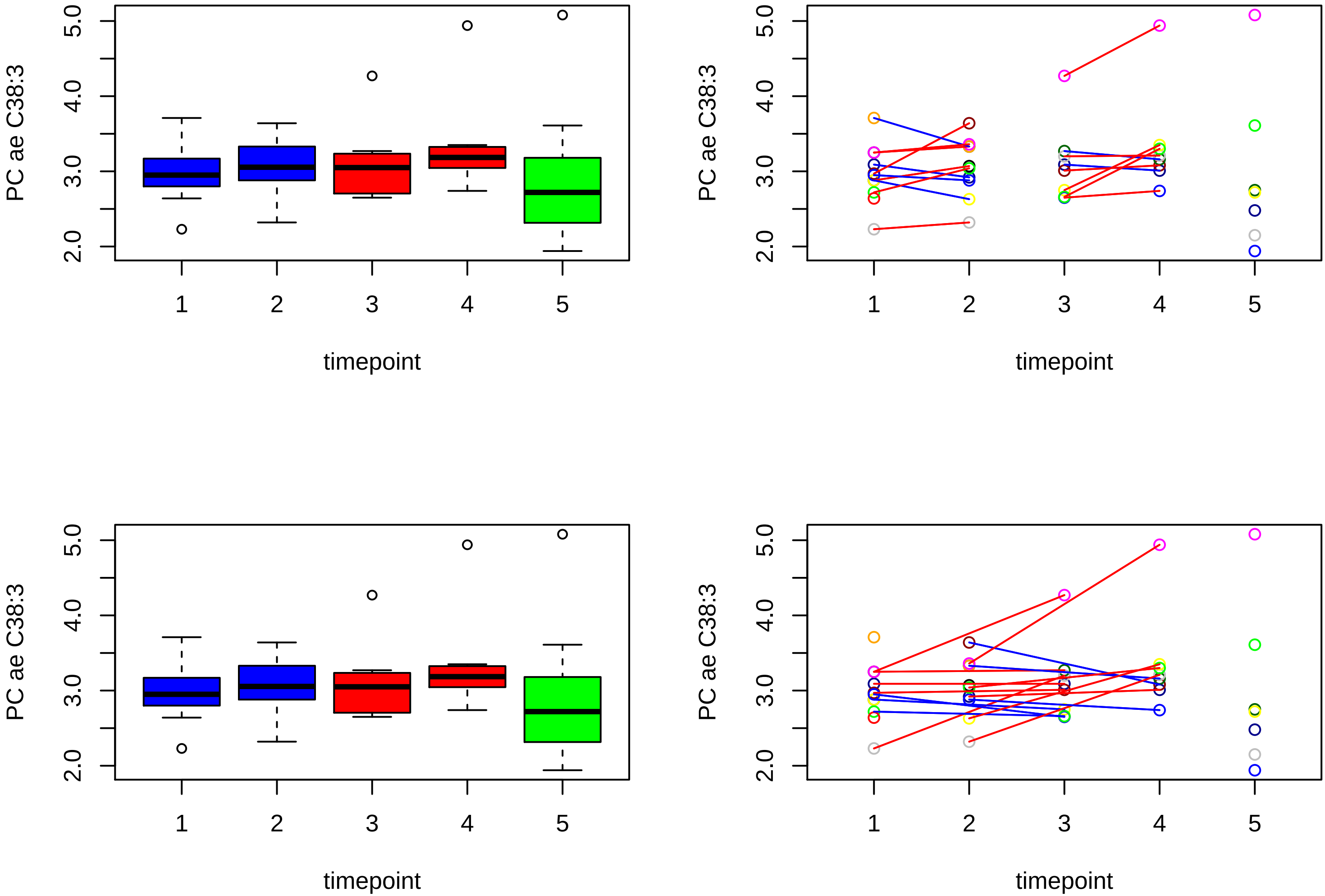
PC ae C38:3
timepoint
1
2
3
4
5
2.0
3.0
4.0
5.0

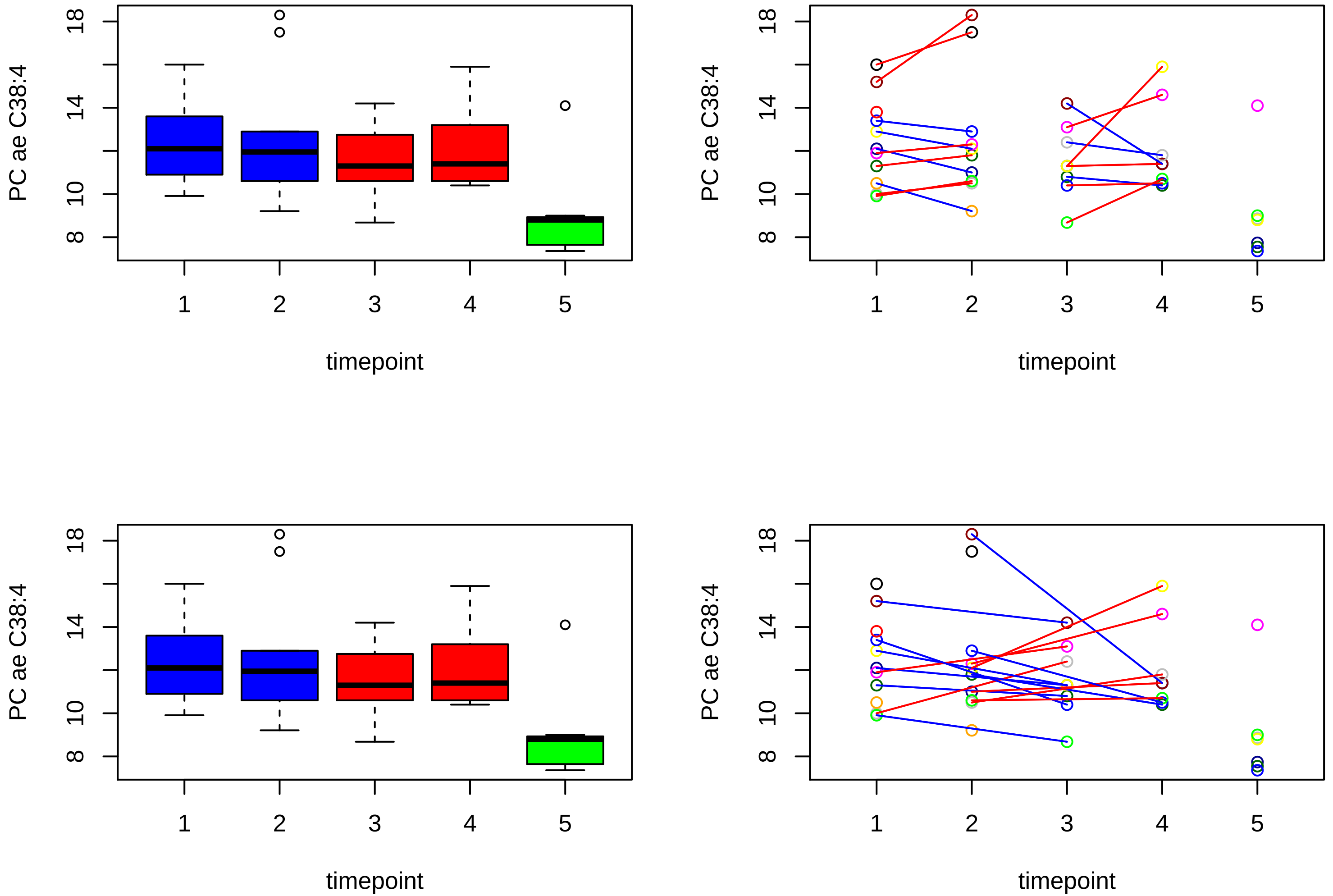

PC ae C38:4
timepoint
8
10
14
18
1
2
3
4
5

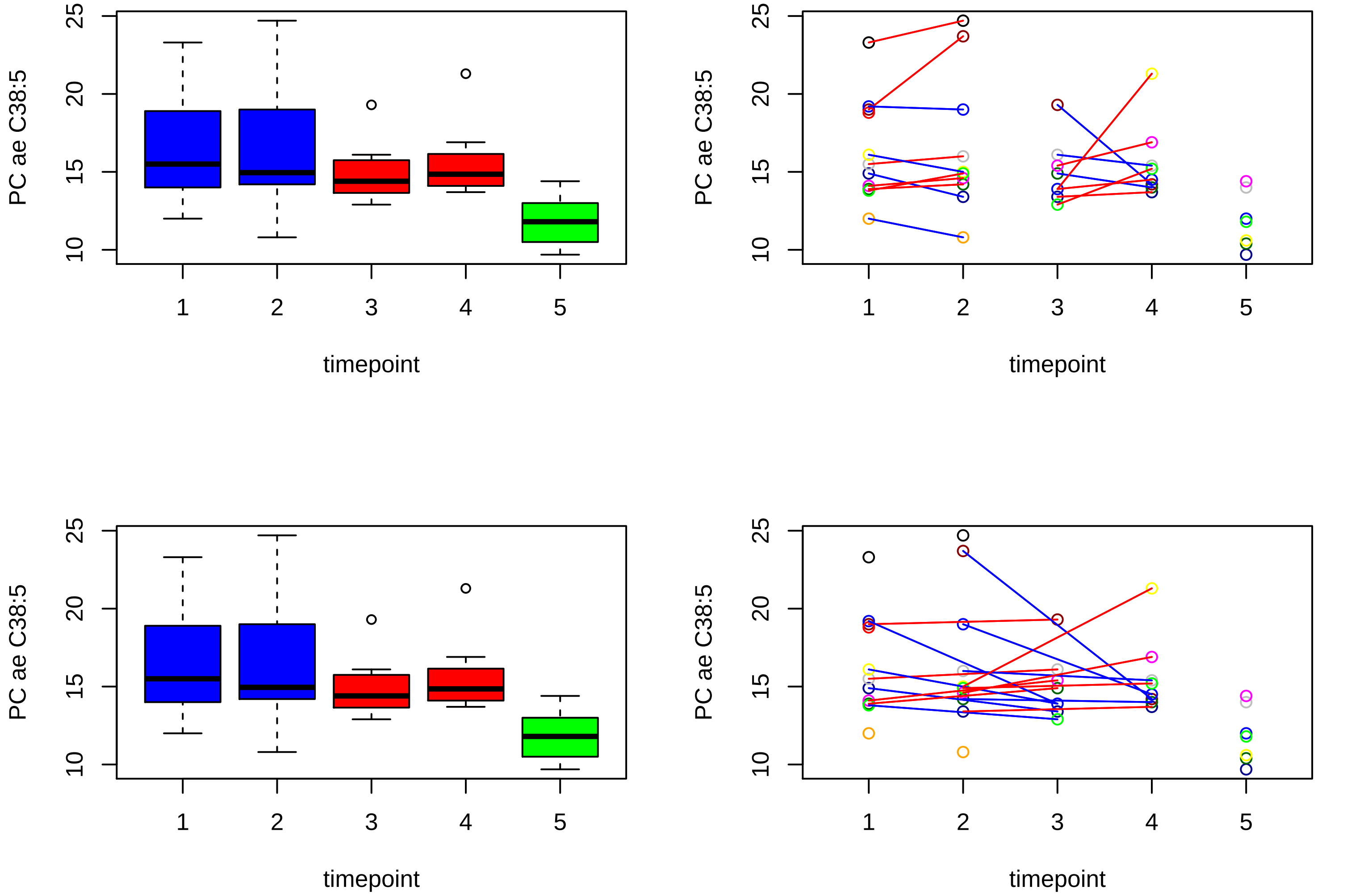
PC ae C38:5
timepoint
PC ae C38:5
timepoint
PC ae C38:5
timepoint
PC ae C38:5
timepoint

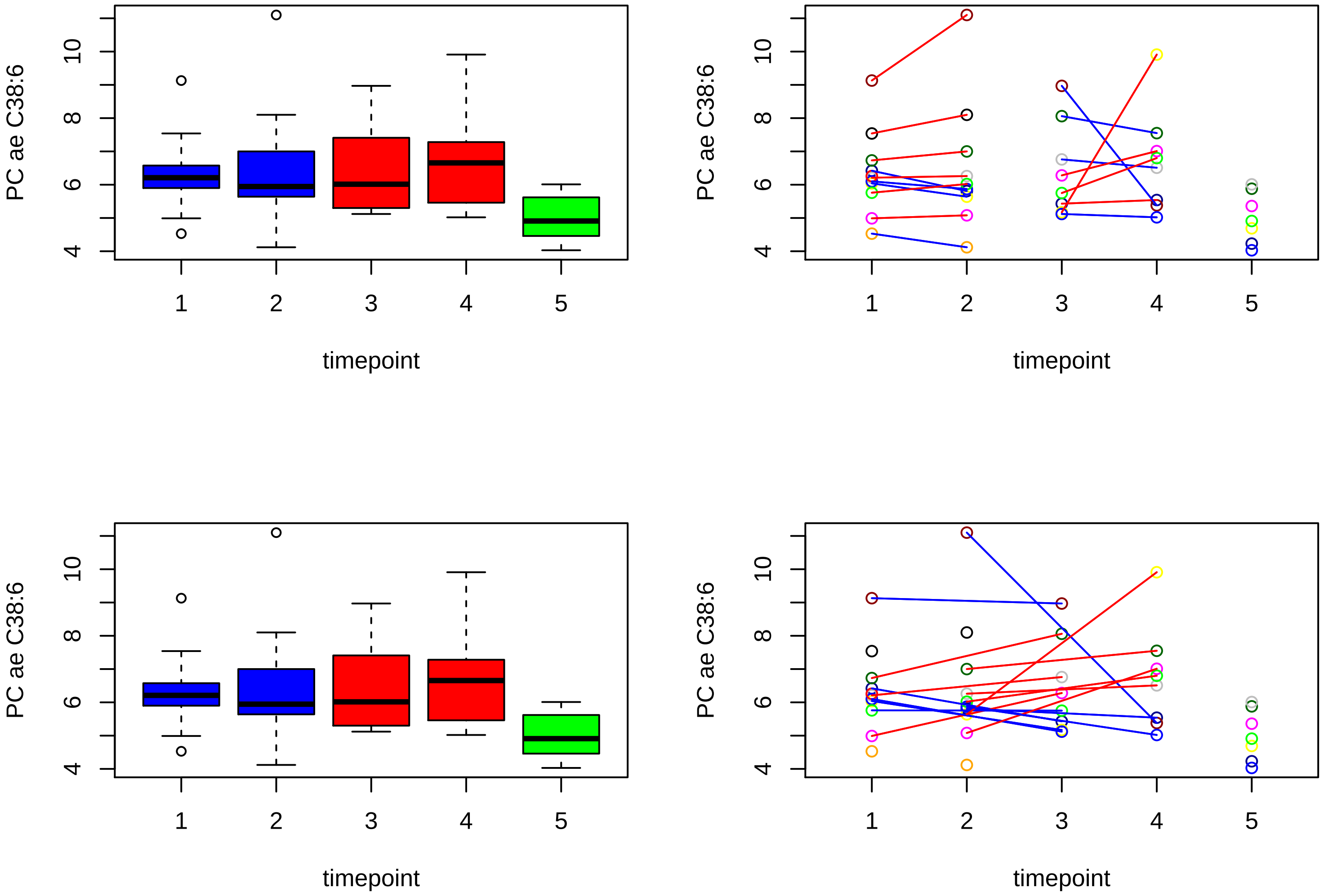
PC ae C38:6
timepoint
4
6
8
10
1
2
3
4
5

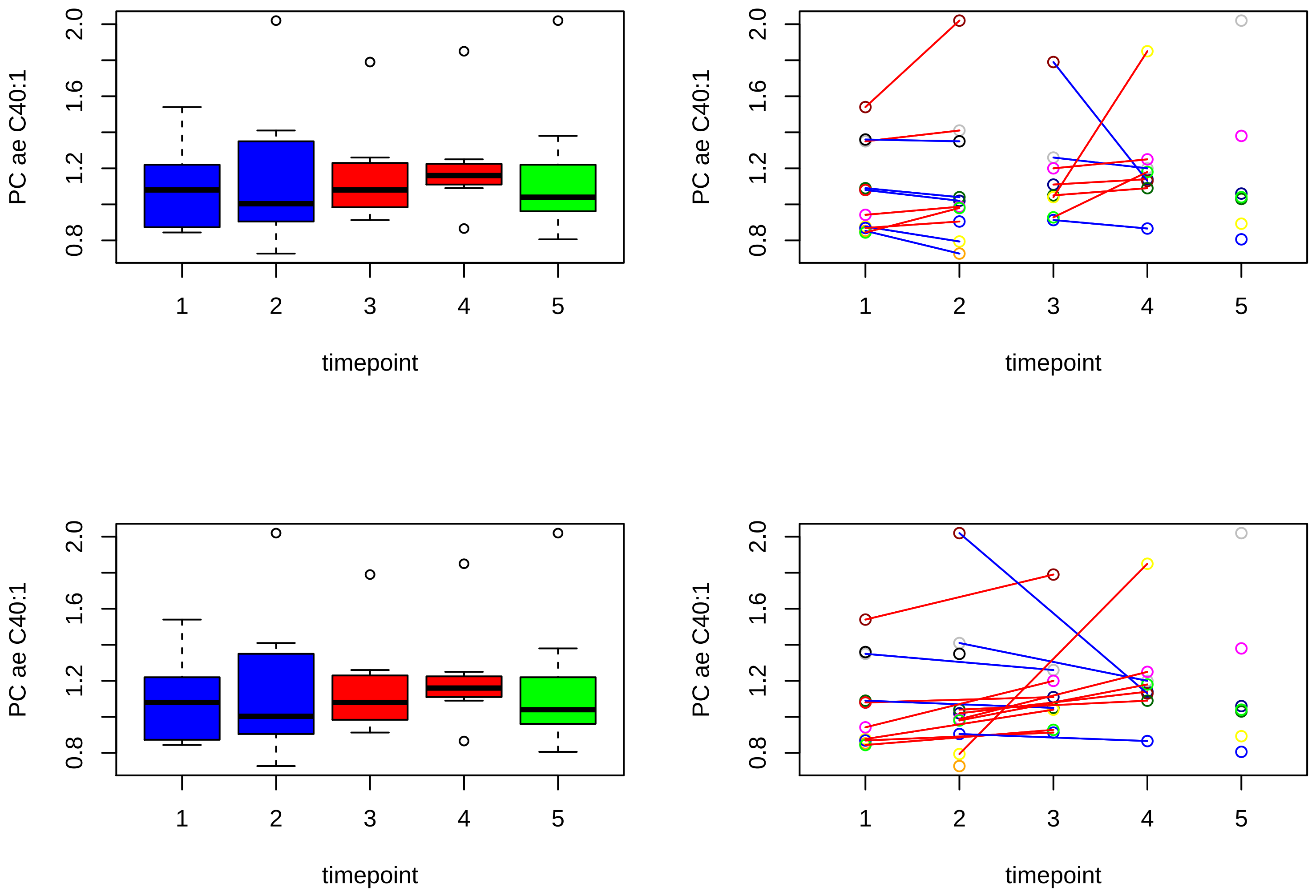

PC ae C40:1
0.8
1.2
1.6
2.0
1
2
3
4
5
timepoint

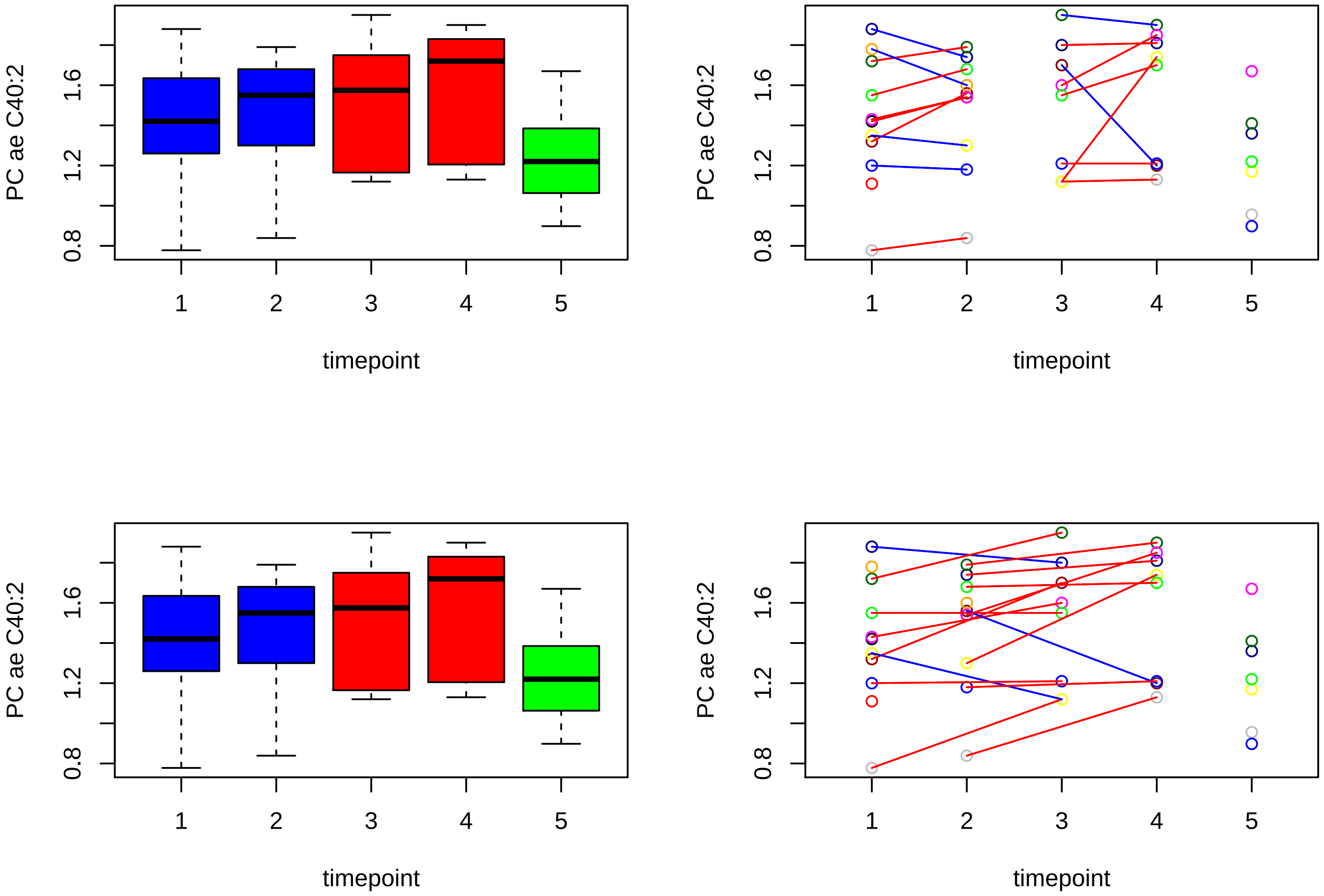
PC ae C40:2
0.8
1.2
1.6
1
2
3
4
5
timepoint
PC ae C40:2
0.8
1.2
1.6
1
2
3
4
5
timepoint
PC ae C40:2
0.8
1.2
1.6
1
2
3
4
5
timepoint
PC ae C40:2
0.8
1.2
1.6
1
2
3
4
5
timepoint

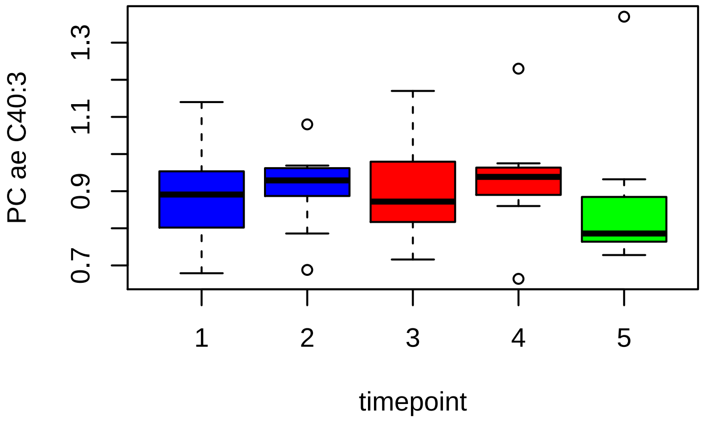

PC ae C40:3
0.7
0.9
1.1
1.3
1
2
3
4
5
timepoint

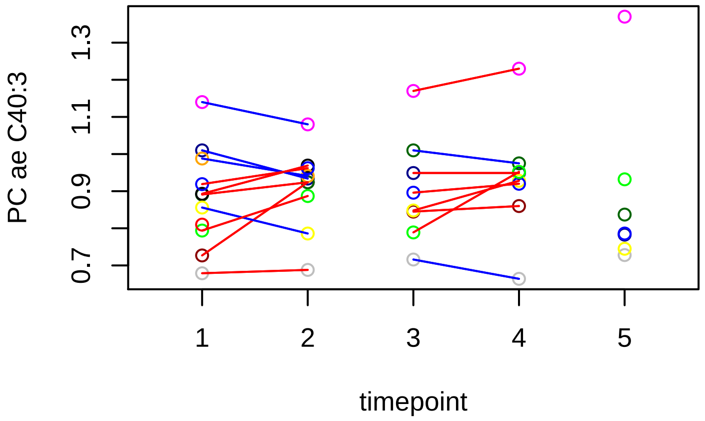

PC ae C40:3
0.7
0.9
1.1
1.3
1
2
3
4
5
timepoint

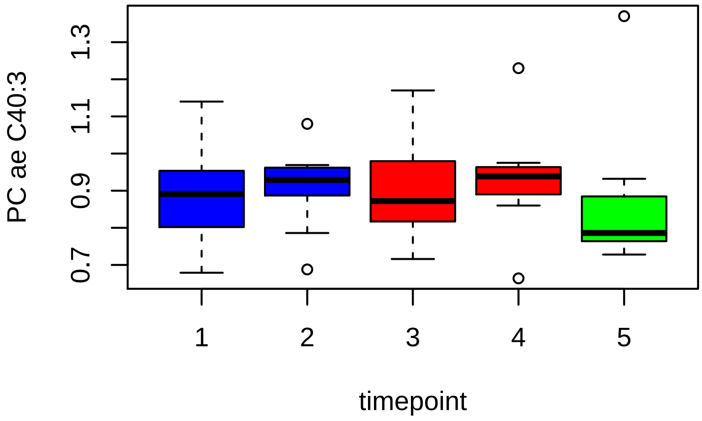

PC ae C40:3
0.7
0.9
1.1
1.3
1
2
3
4
5
timepoint

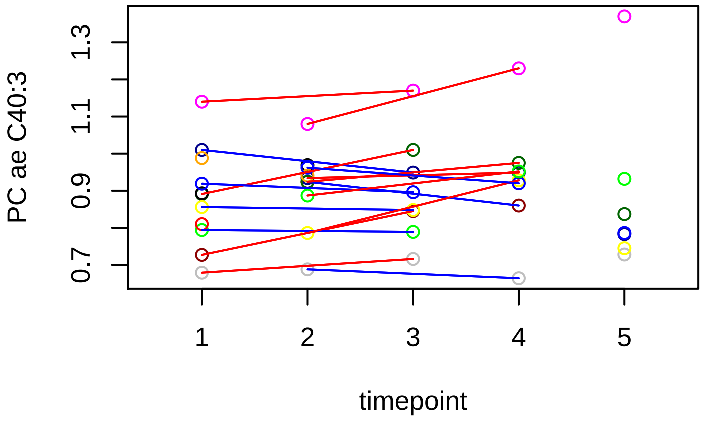

PC ae C40:3
0.7
0.9
1.1
1.3
1
2
3
4
5
timepoint

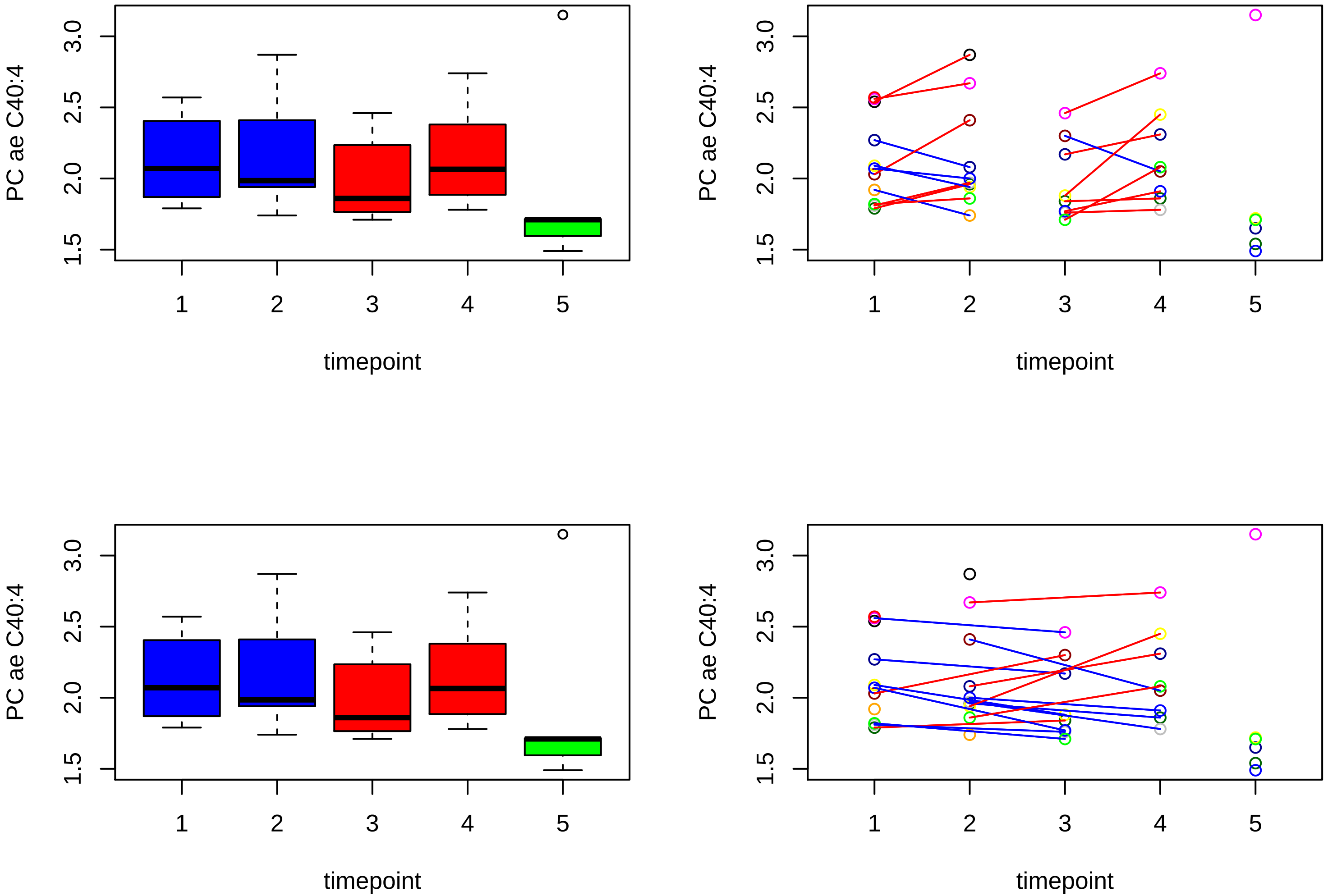
PC ae C40:4
timepoint
1.5
2.0
2.5
3.0
1
2
3
4
5

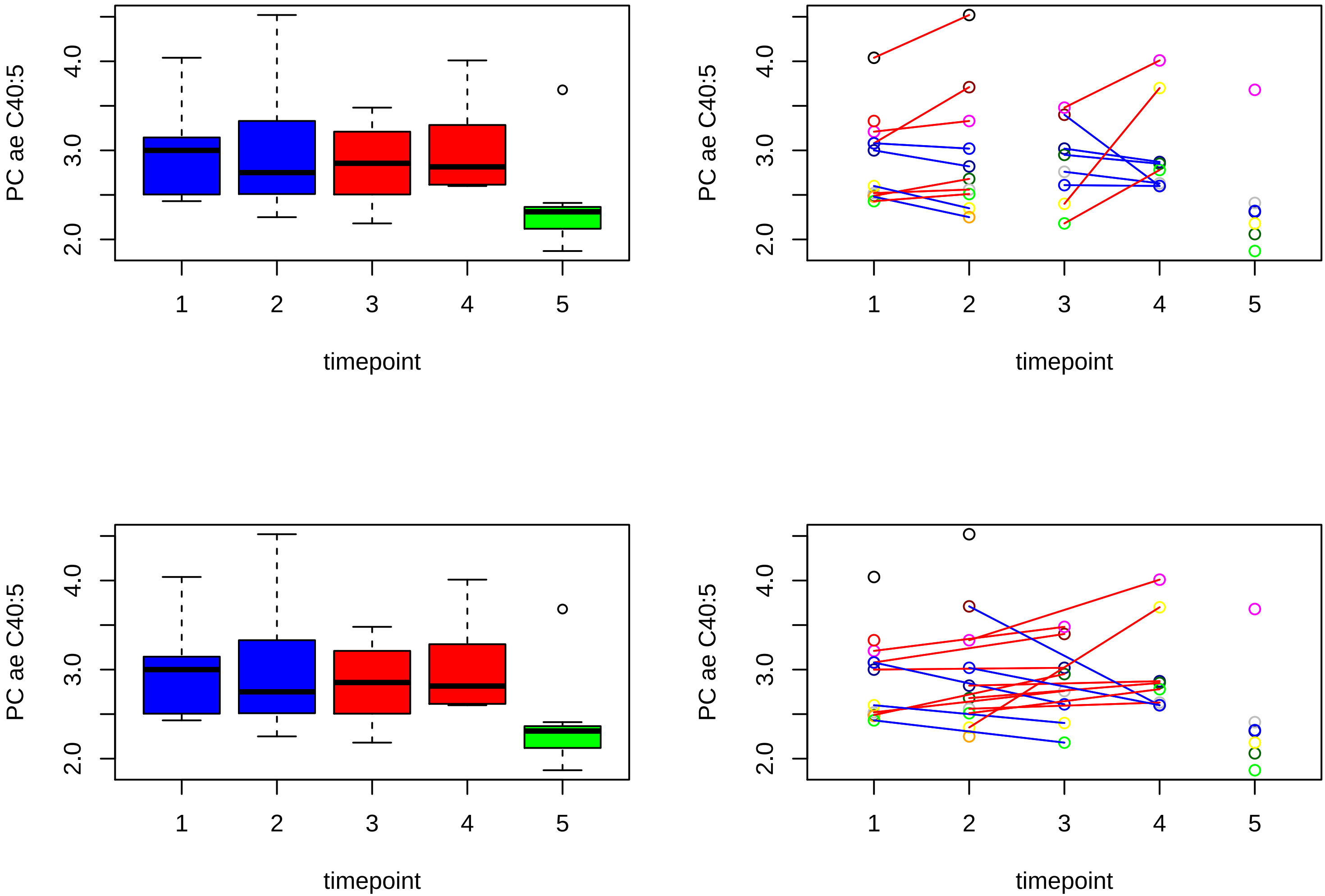

PC ae C40:5
2.0
3.0
4.0
1
2
3
4
5
timepoint
PC ae C40:5
2.0
3.0
4.0
1
2
3
4
5
timepoint
PC ae C40:5
2.0
3.0
4.0
1
2
3
4
5
timepoint
PC ae C40:5
2.0
3.0
4.0
1
2
3
4
5
timepoint

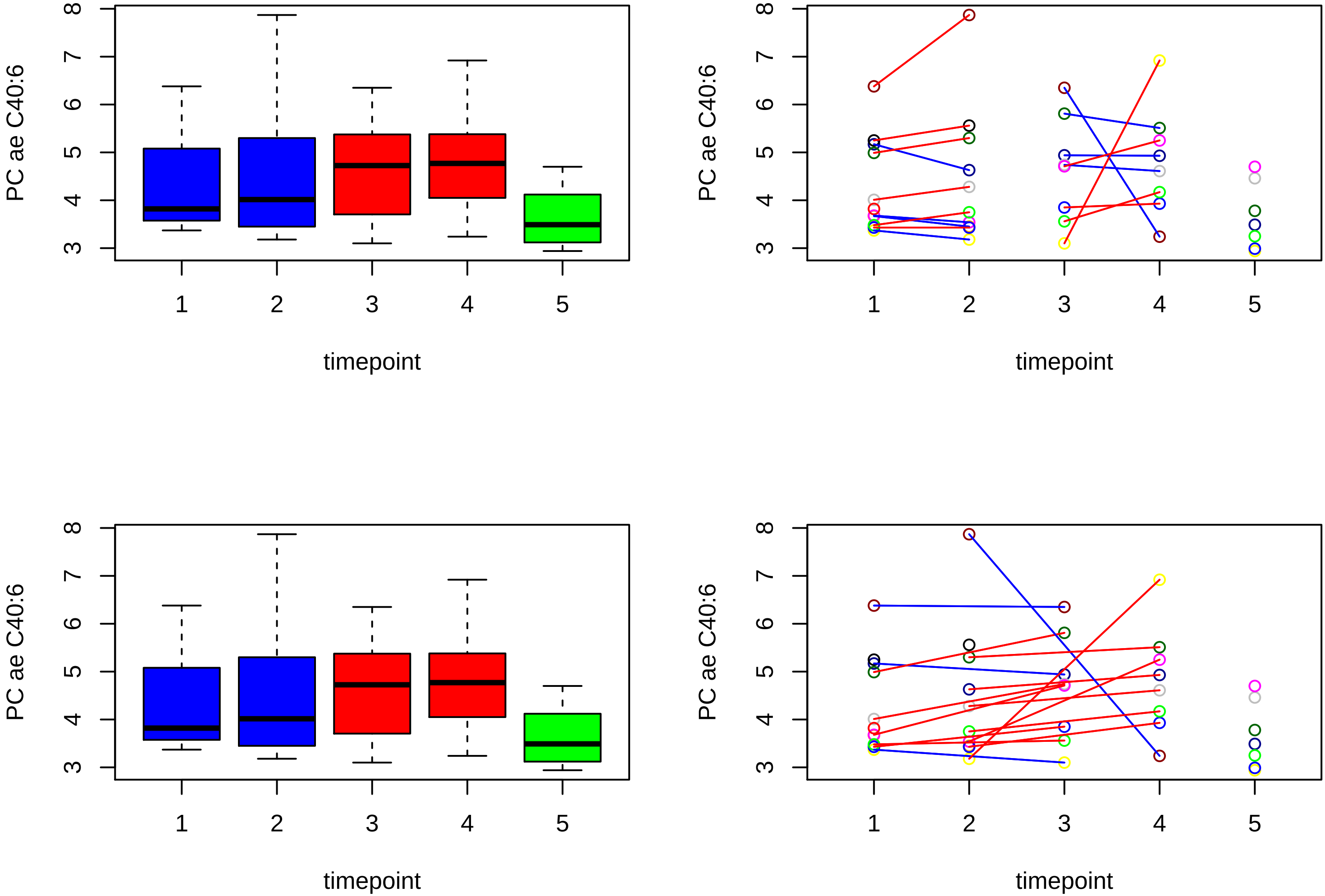
PC ae C40:6
timepoint
1
2
3
4
5
3
4
5
6
7
8

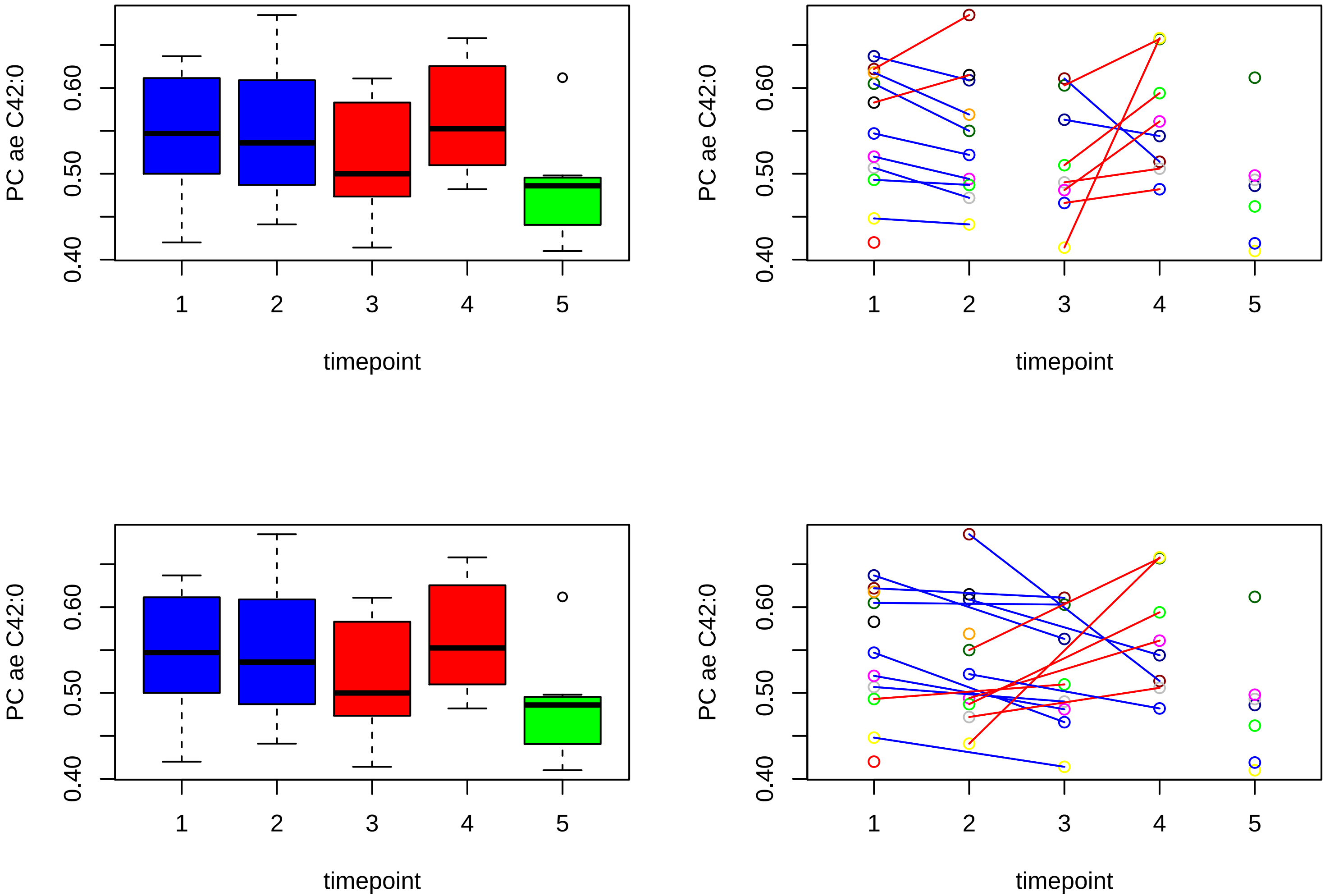
PC ae C42:0
0.40
0.50
0.60
1
2
3
4
5
timepoint

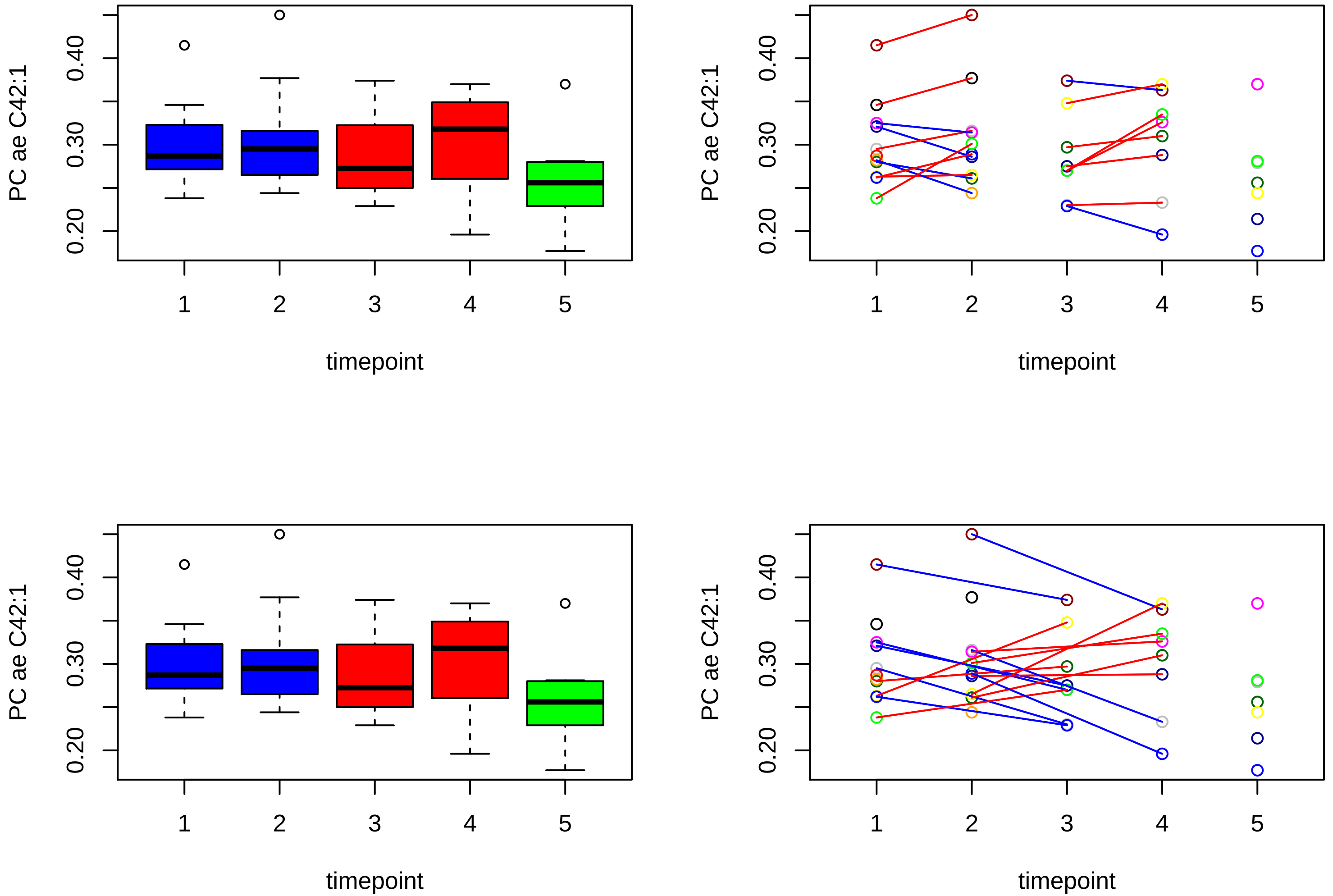
PC ae C42:1
0.20
0.30
0.40
1
2
3
4
5
timepoint
PC ae C42:1
0.20
0.30
0.40
1
2
3
4
5
timepoint
PC ae C42:1
0.20
0.30
0.40
1
2
3
4
5
timepoint
PC ae C42:1
0.20
0.30
0.40
1
2
3
4
5
timepoint

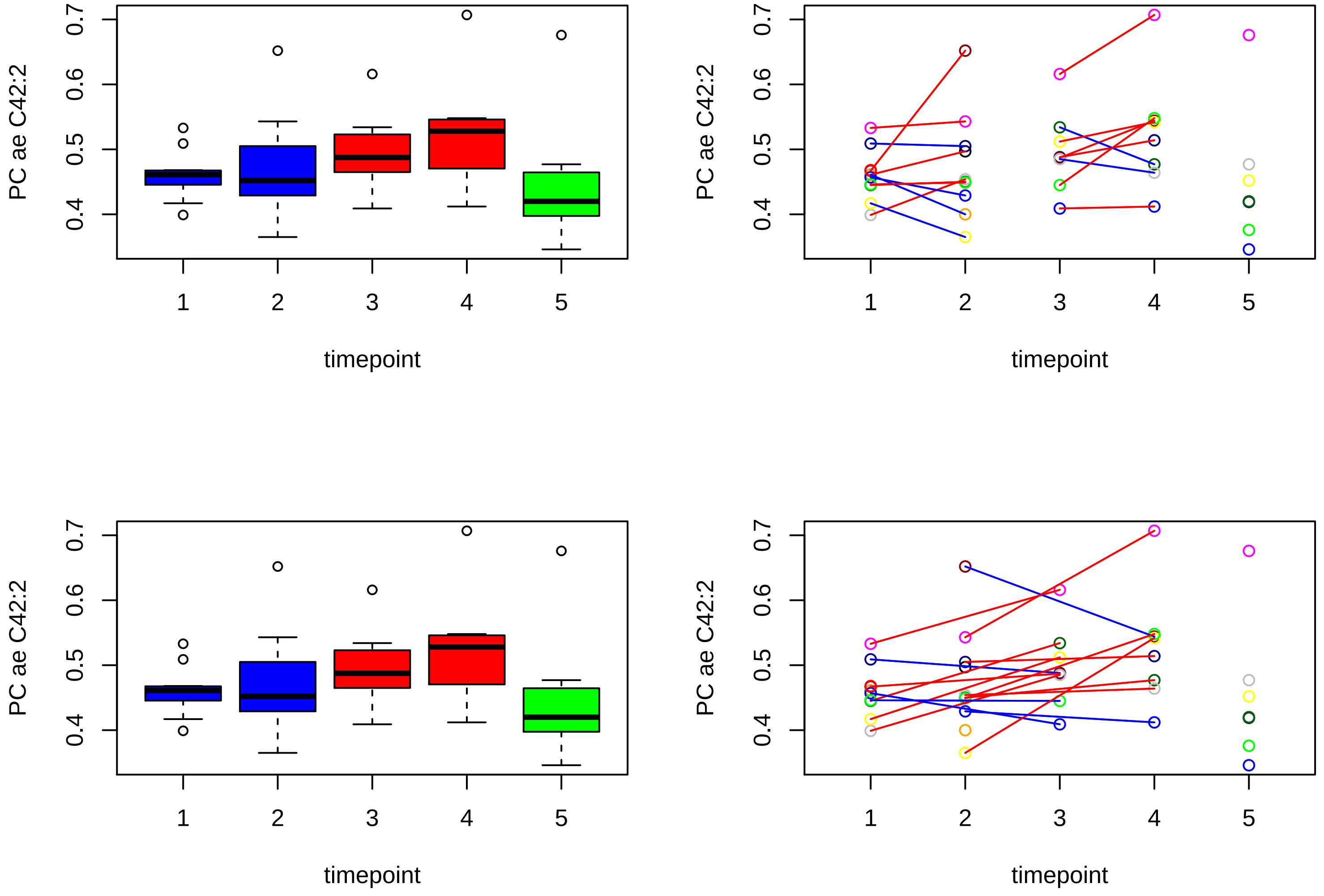
PC ae C42:2
0.4
0.5
0.6
0.7
1
2
3
4
5
timepoint

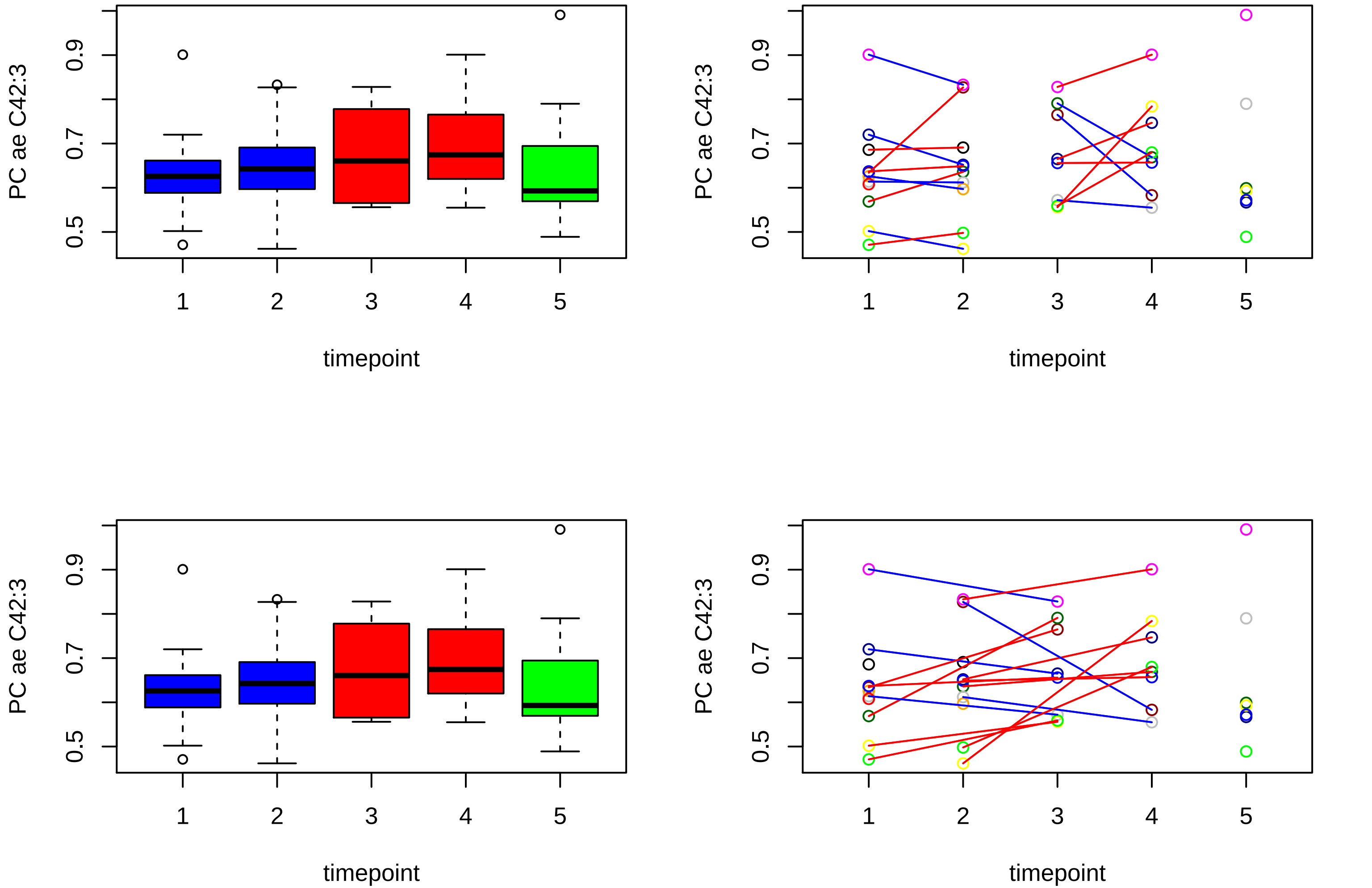
PC ae C42:3
0.5
0.7
0.9
1
2
3
4
5
timepoint
PC ae C42:3
0.5
0.7
0.9
1
2
3
4
5
timepoint
PC ae C42:3
0.5
0.7
0.9
1
2
3
4
5
timepoint
PC ae C42:3
0.5
0.7
0.9
1
2
3
4
5
timepoint

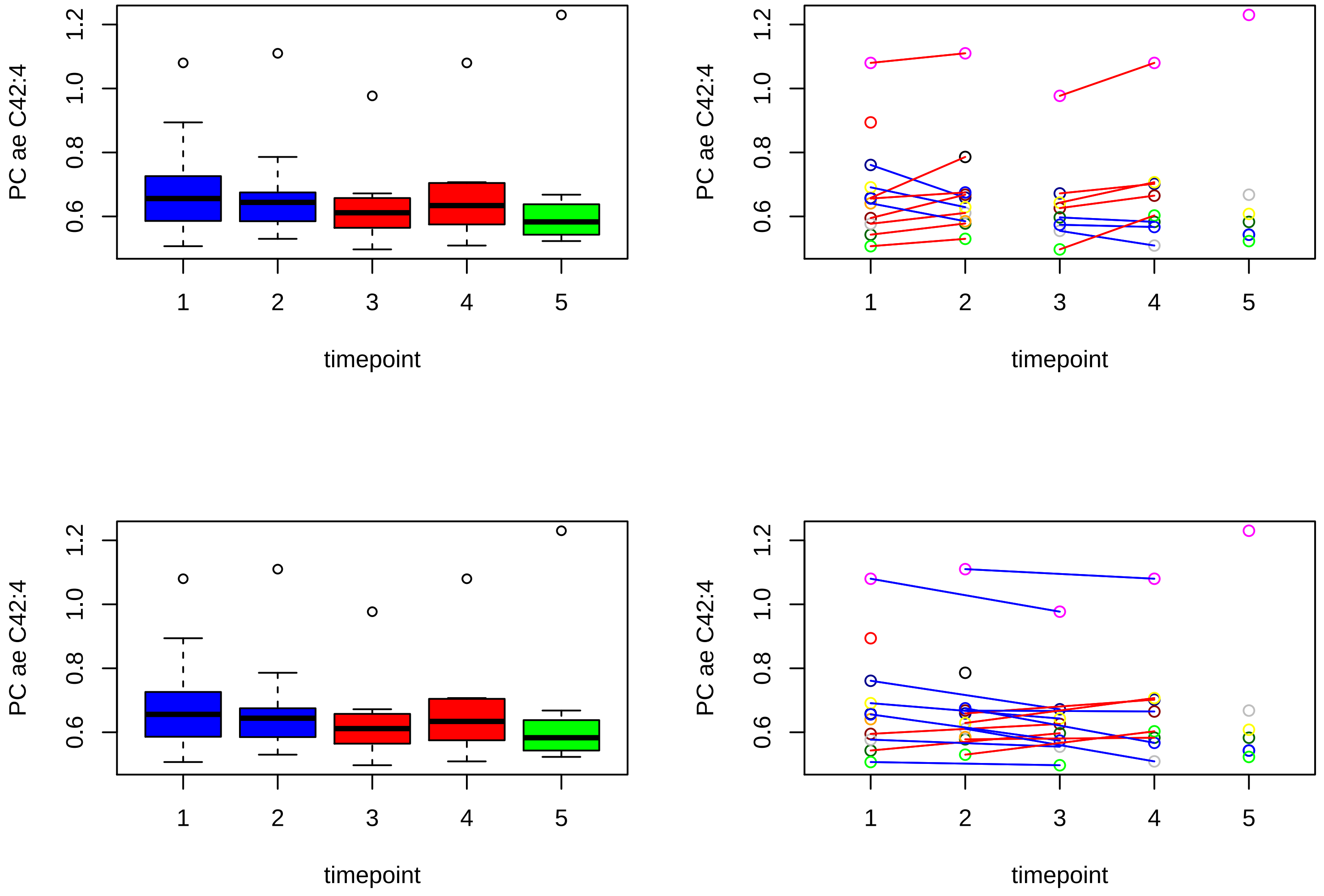

PC ae C42:4
timepoint
0.6
0.8
1.0
1.2
1
2
3
4
5

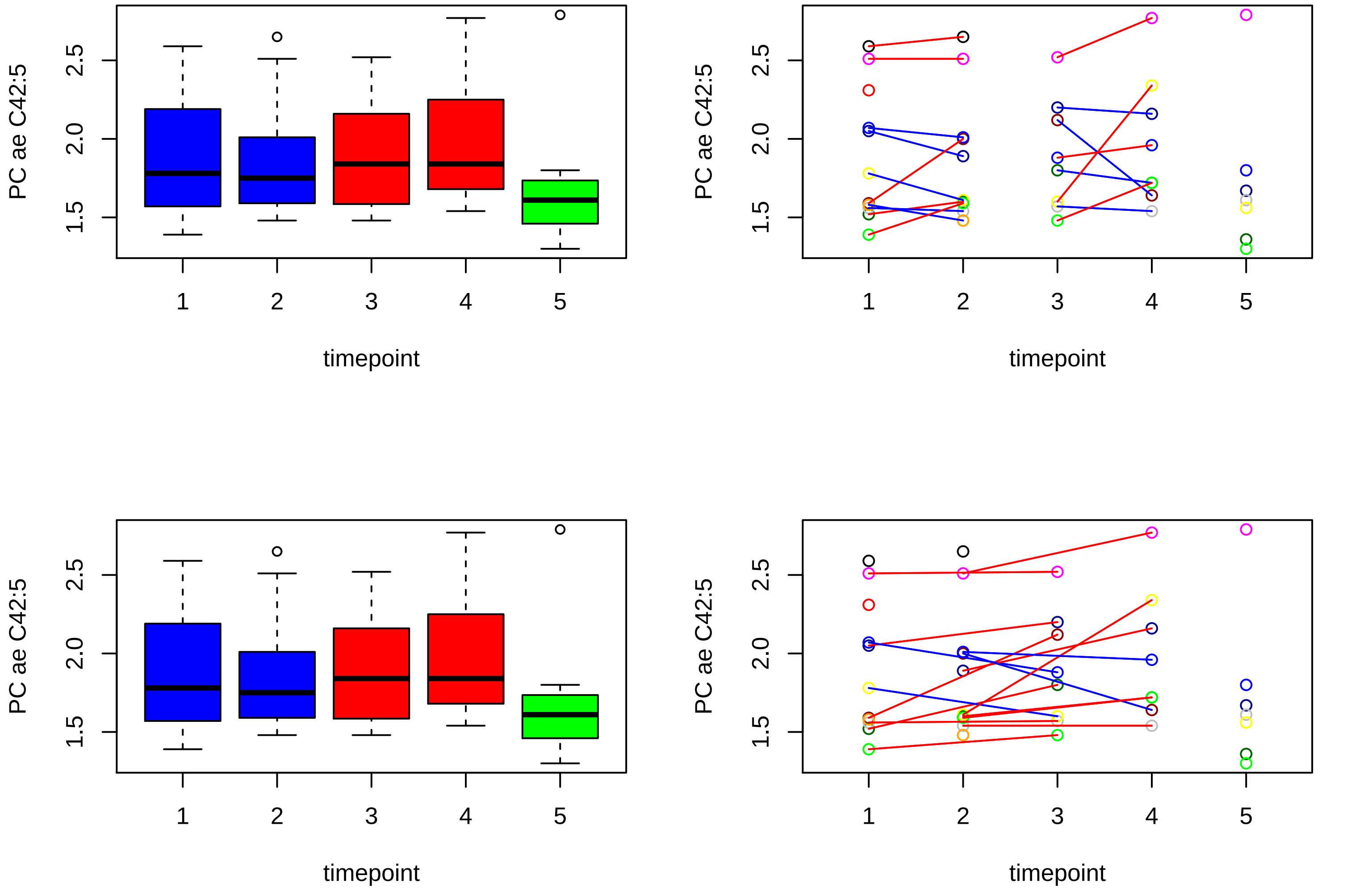

PC ae C42:5
1.5
2.0
2.5
1
2
3
4
5
timepoint

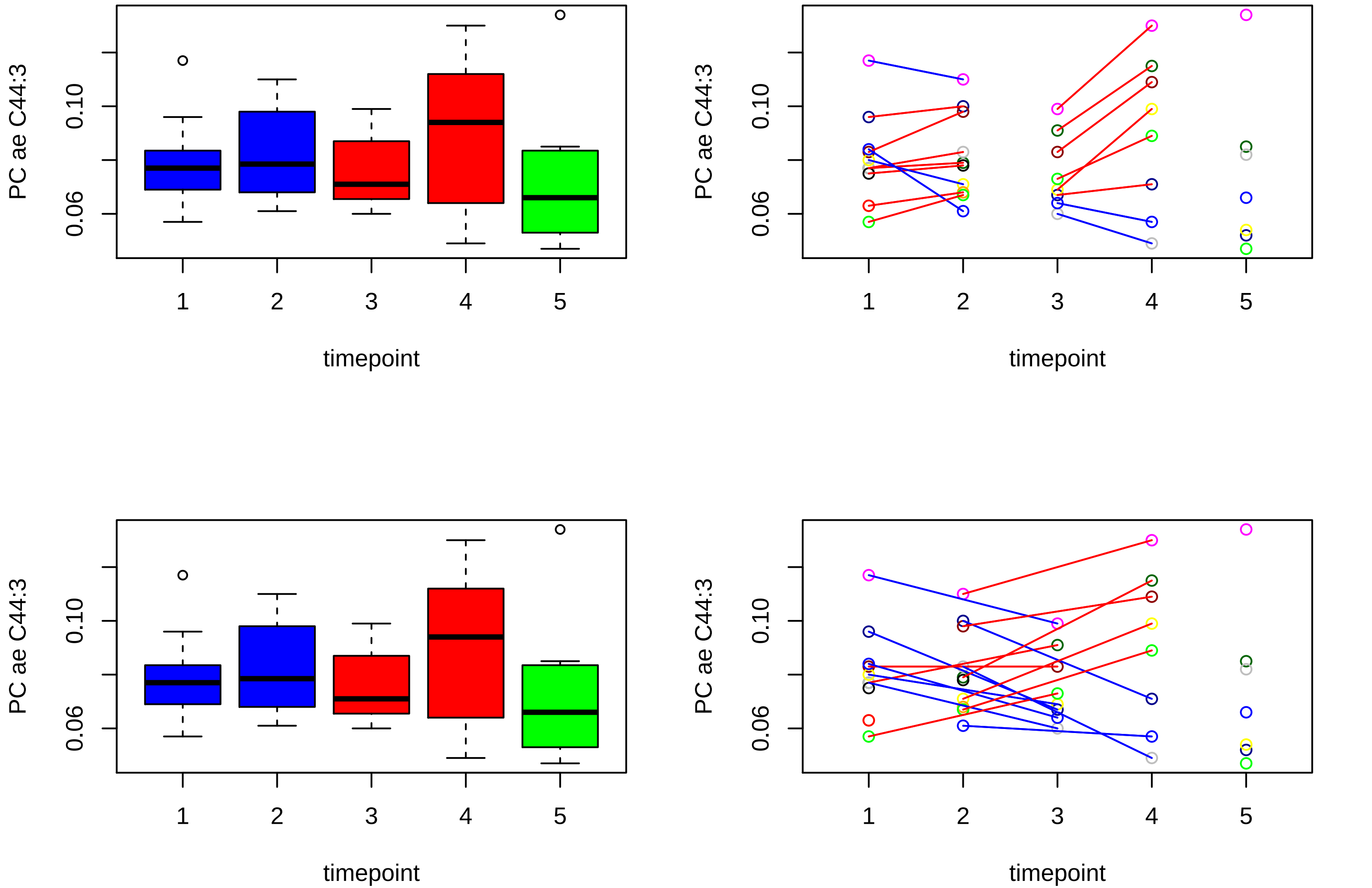
PC ae C44:3
0.06
0.10
1
2
3
4
5
timepoint
PC ae C44:3
0.06
0.10
1
2
3
4
5
timepoint
PC ae C44:3
0.06
0.10
1
2
3
4
5
timepoint
PC ae C44:3
0.06
0.10
1
2
3
4
5
timepoint

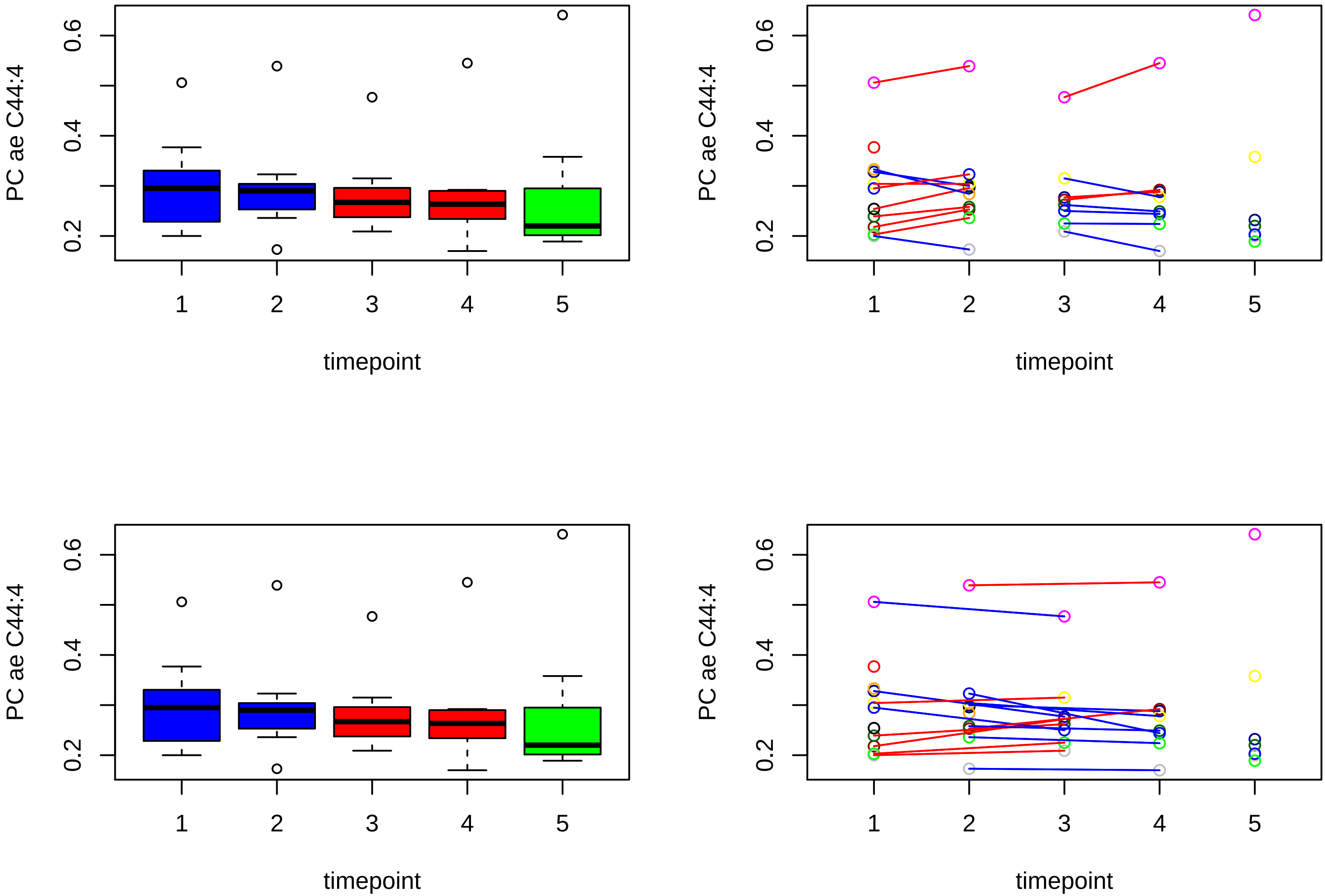
PC ae C44:4
0.2
0.4
0.6
1
2
3
4
5
timepoint
PC ae C44:4
0.2
0.4
0.6
1
2
3
4
5
timepoint
PC ae C44:4
0.2
0.4
0.6
1
2
3
4
5
timepoint
PC ae C44:4
0.2
0.4
0.6
1
2
3
4
5
timepoint

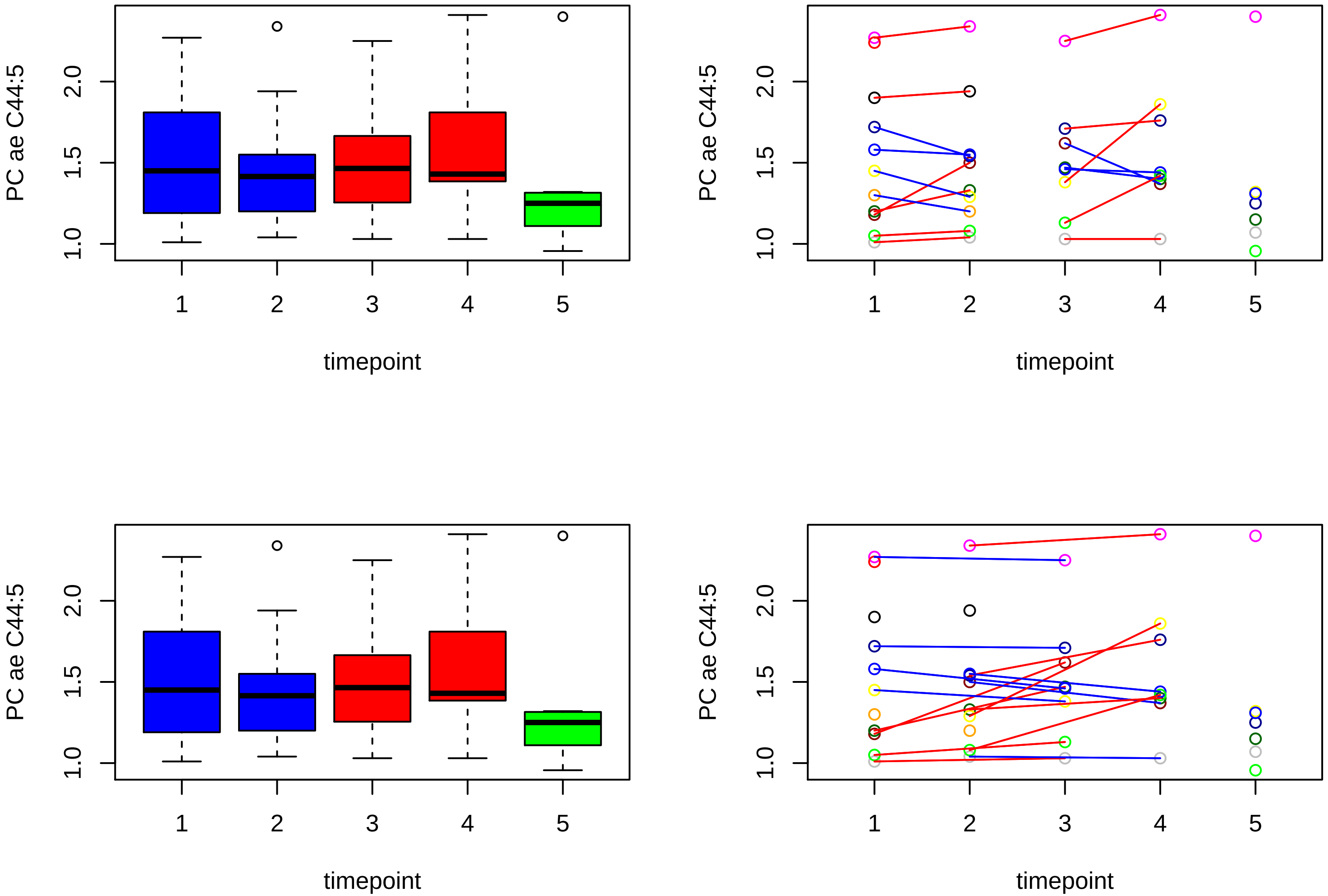
PC ae C44:5
1.0
1.5
2.0
1
2
3
4
5
timepoint

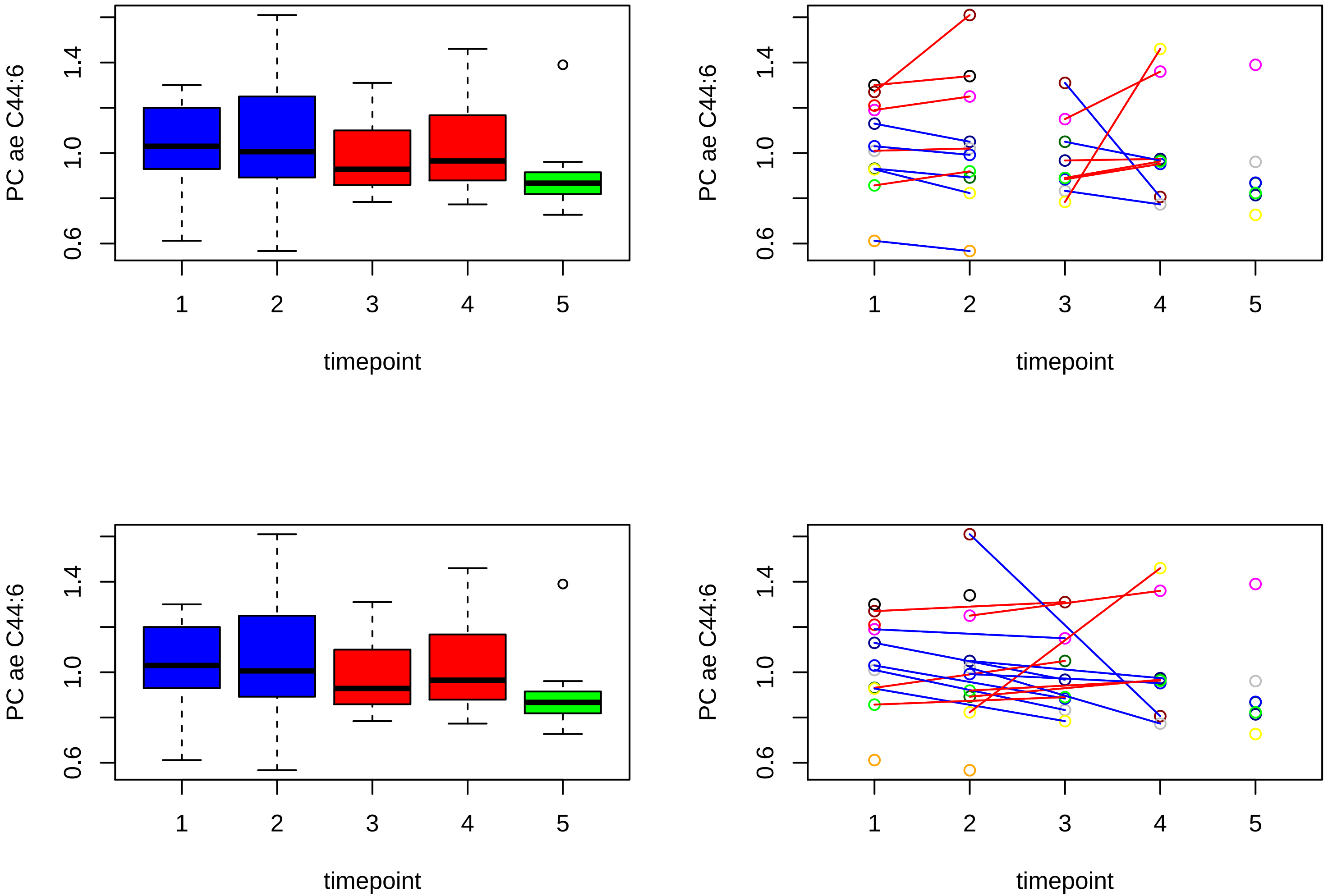
PC ae C44:6
0.6
1.0
1.4
1
2
3
4
5
timepoint
PC ae C44:6
0.6
1.0
1.4
1
2
3
4
5
timepoint
PC ae C44:6
0.6
1.0
1.4
1
2
3
4
5
timepoint
PC ae C44:6
0.6
1.0
1.4
1
2
3
4
5
timepoint

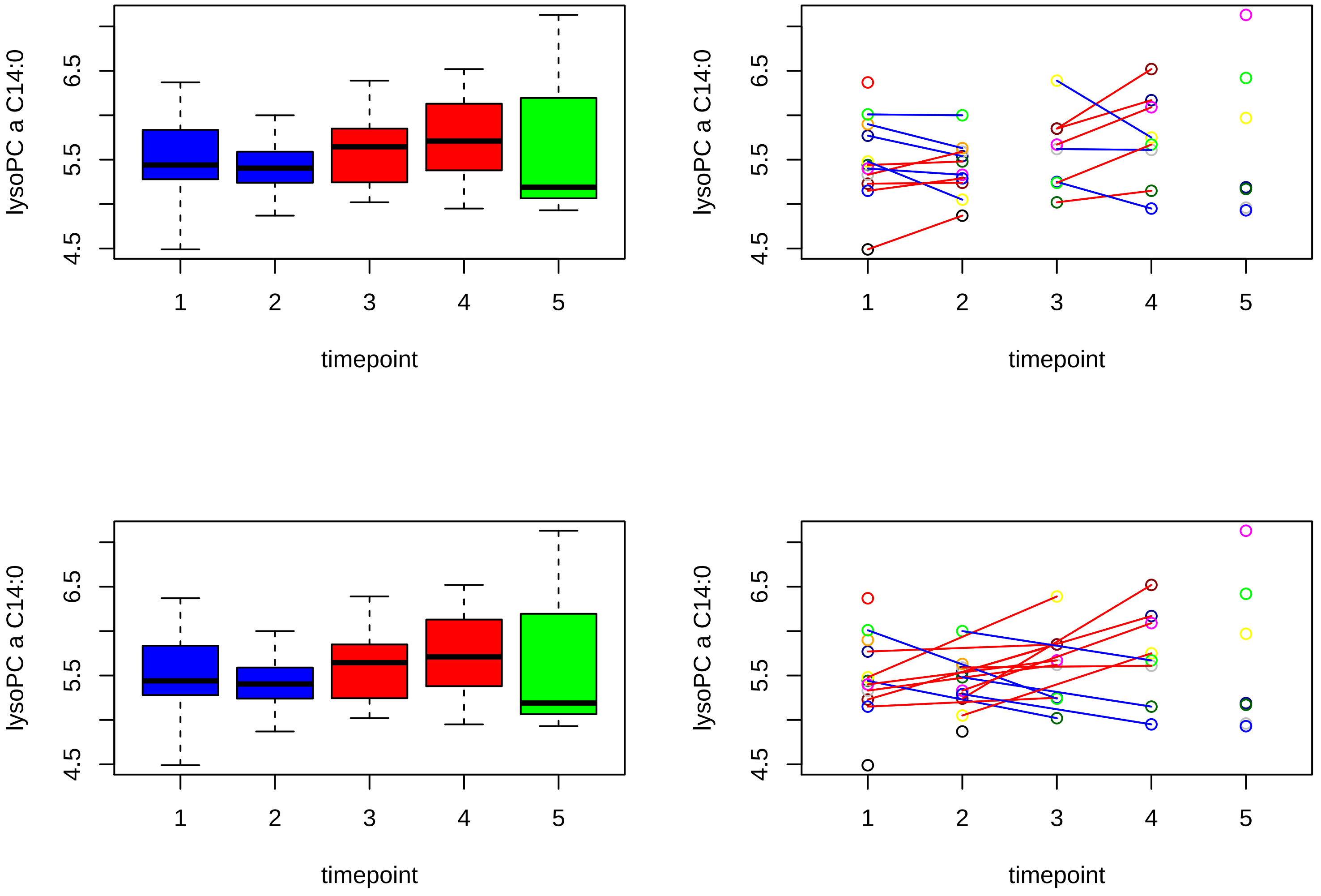
lysoPC a C14:0
4.5
5.5
6.5
1
2
3
4
5
timepoint
lysoPC a C14:0
4.5
5.5
6.5
1
2
3
4
5
timepoint
lysoPC a C14:0
4.5
5.5
6.5
1
2
3
4
5
timepoint
lysoPC a C14:0
4.5
5.5
6.5
1
2
3
4
5
timepoint

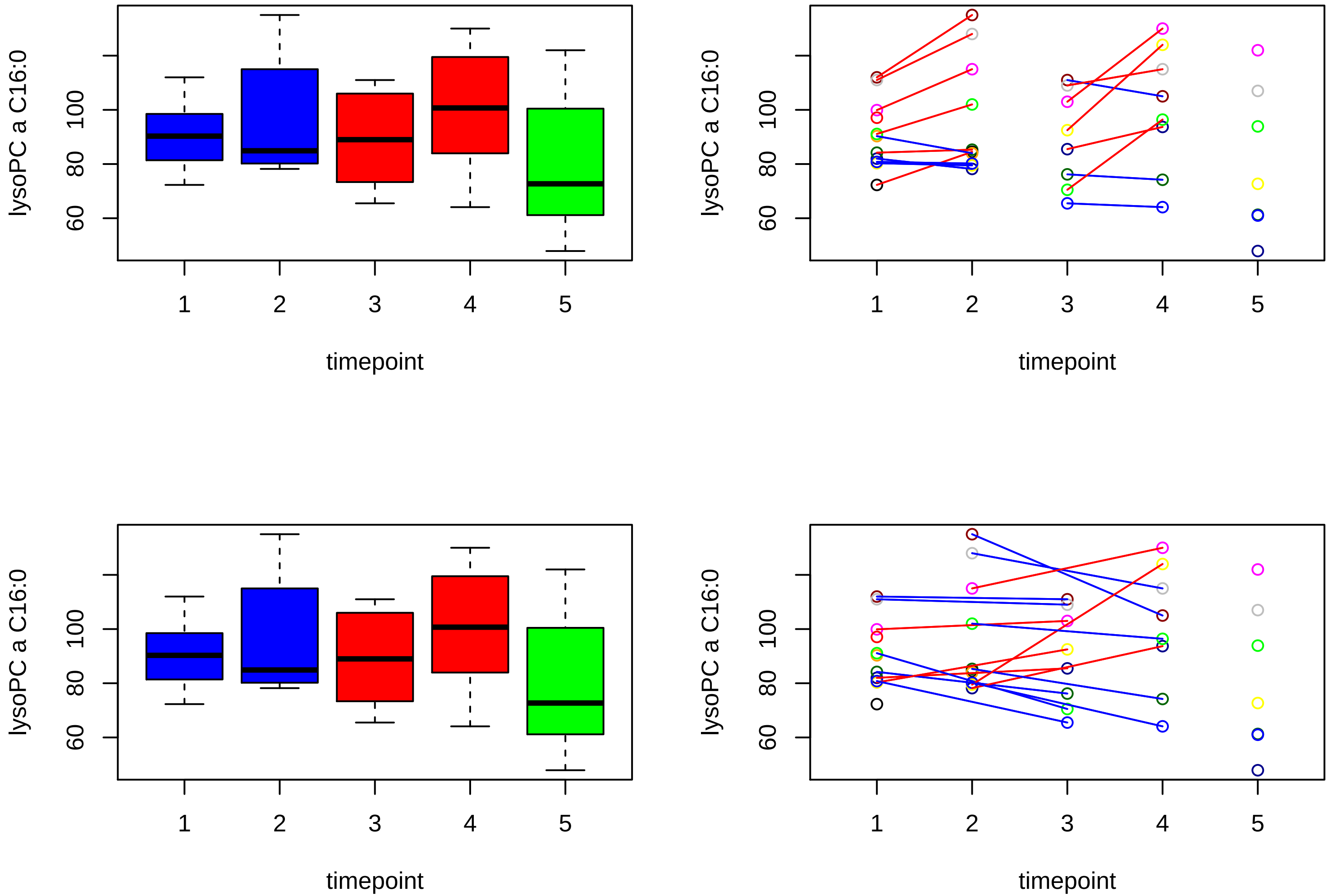
lysoPC a C16:0
60
80
100
1
2
3
4
5
timepoint

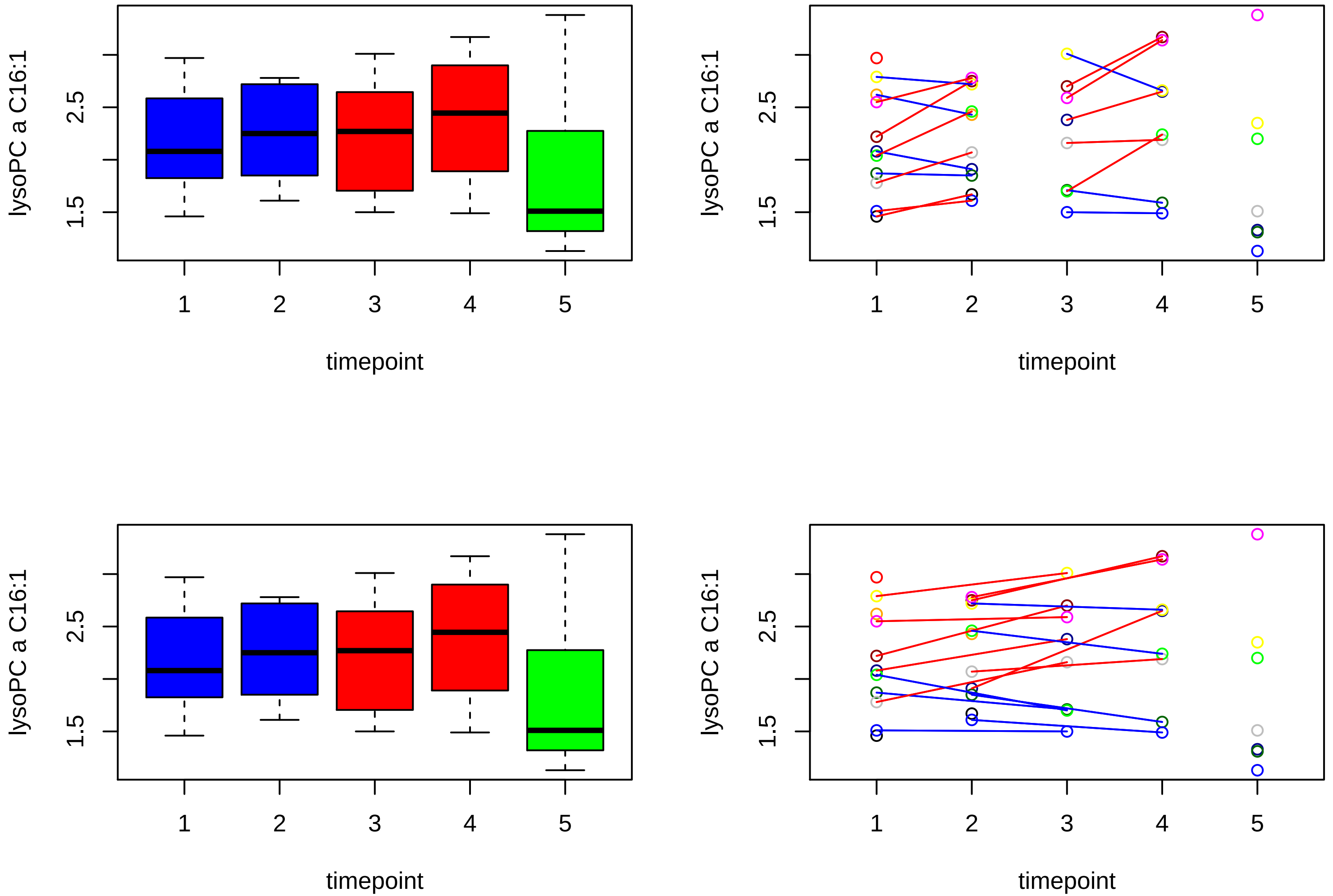
lysoPC a C16:1
1.5
2.5
1
2
3
4
5
timepoint

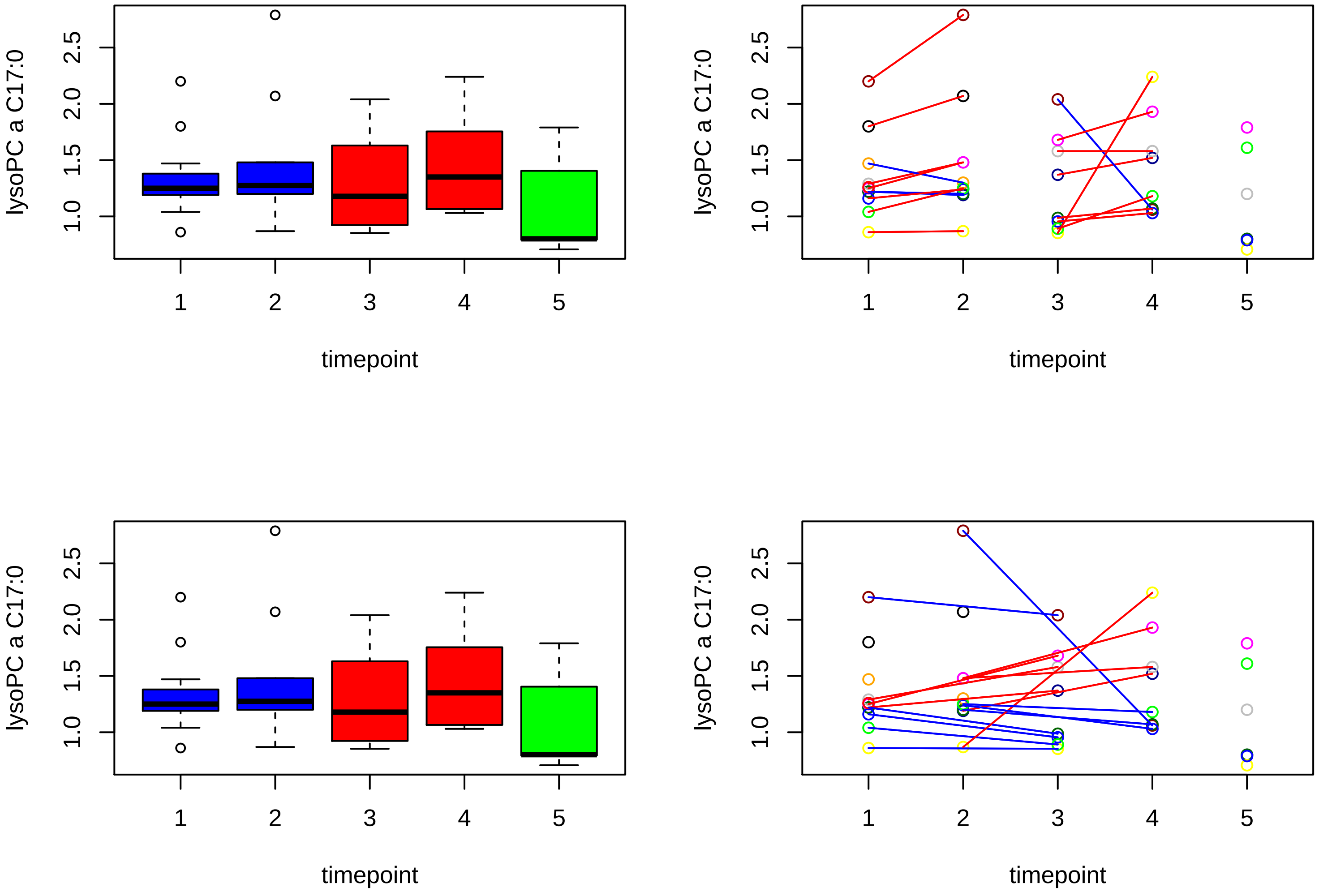

lysoPC a C17:0
1.0
1.5
2.0
2.5
1
2
3
4
5
timepoint

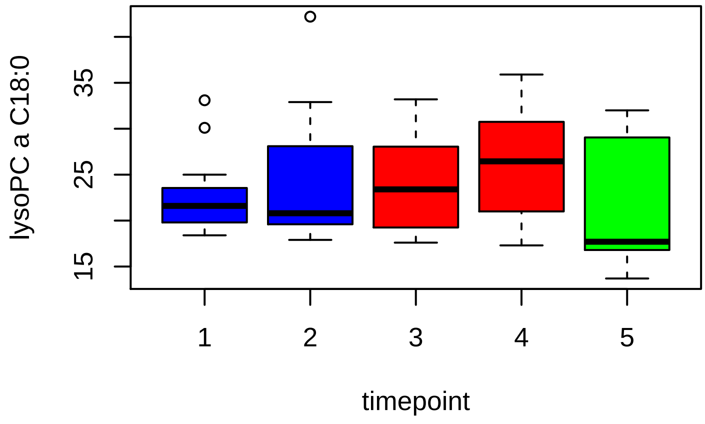

lysoPC a C18:0
15
25
35
1
2
3
4
5
timepoint

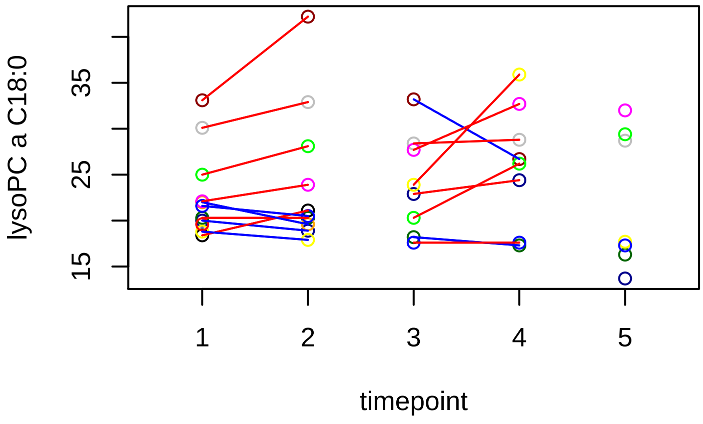

lysoPC a C18:0
15
25
35
1
2
3
4
5
timepoint

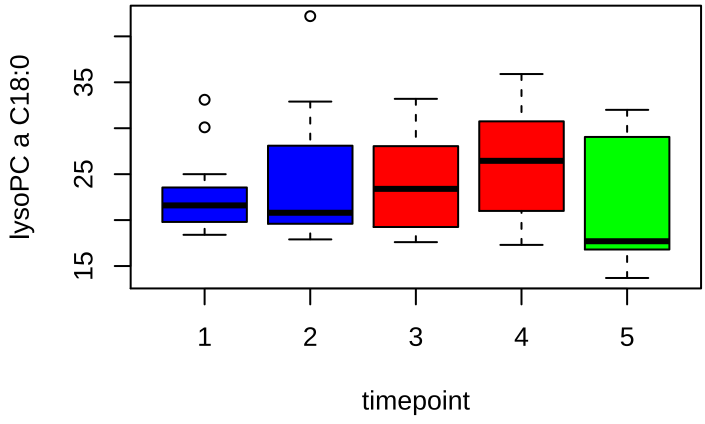

lysoPC a C18:0
15
25
35
1
2
3
4
5
timepoint

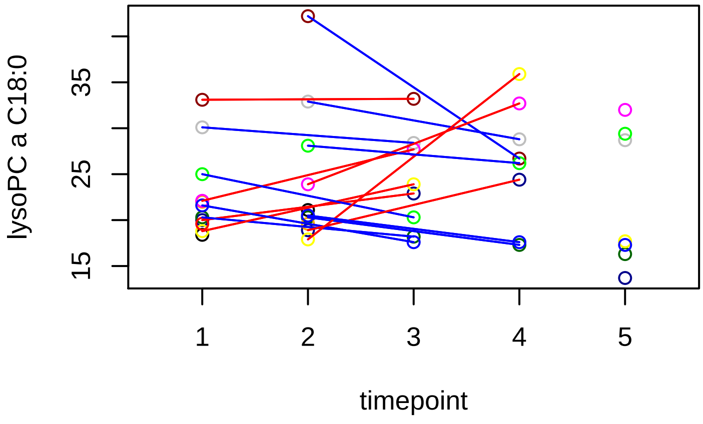

lysoPC a C18:0
15
25
35
1
2
3
4
5
timepoint

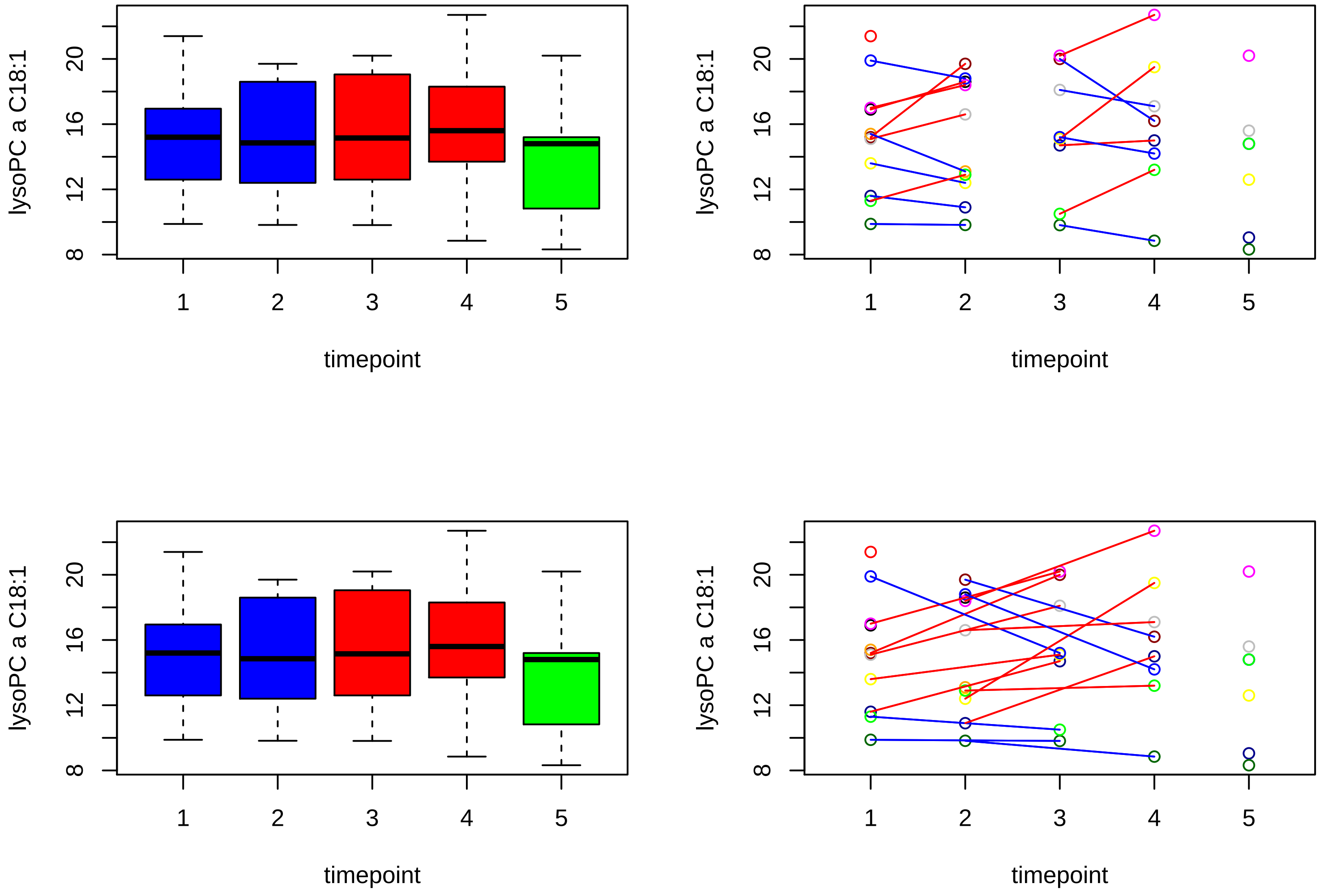
lysoPC a C18:1
timepoint
lysoPC a C18:1
timepoint
lysoPC a C18:1
timepoint
lysoPC a C18:1
timepoint

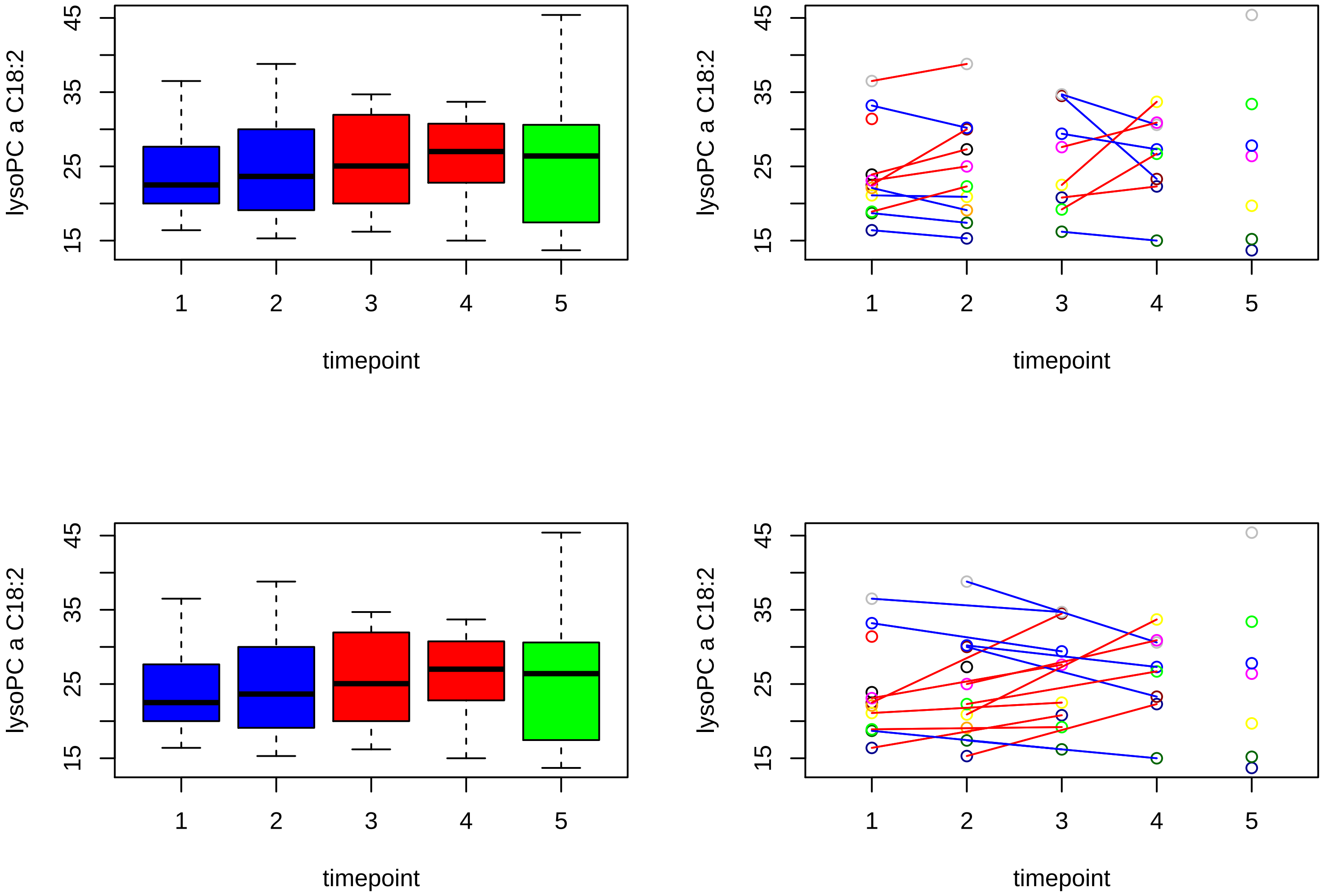
lysoPC a C18:2
timepoint
15
25
35
45
1
2
3
4
5

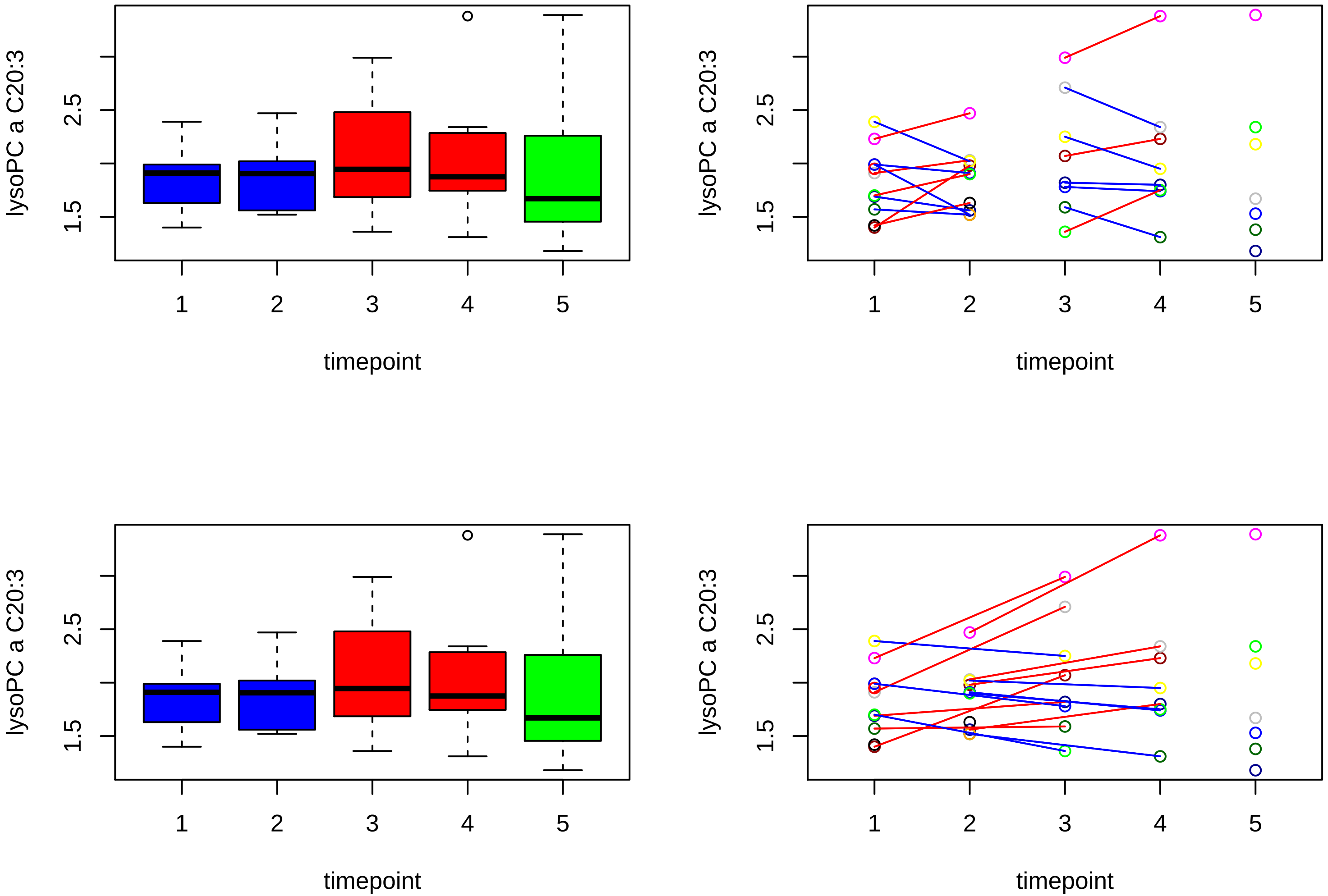
lysoPC a C20:3
1.5
2.5
1
2
3
4
5
timepoint

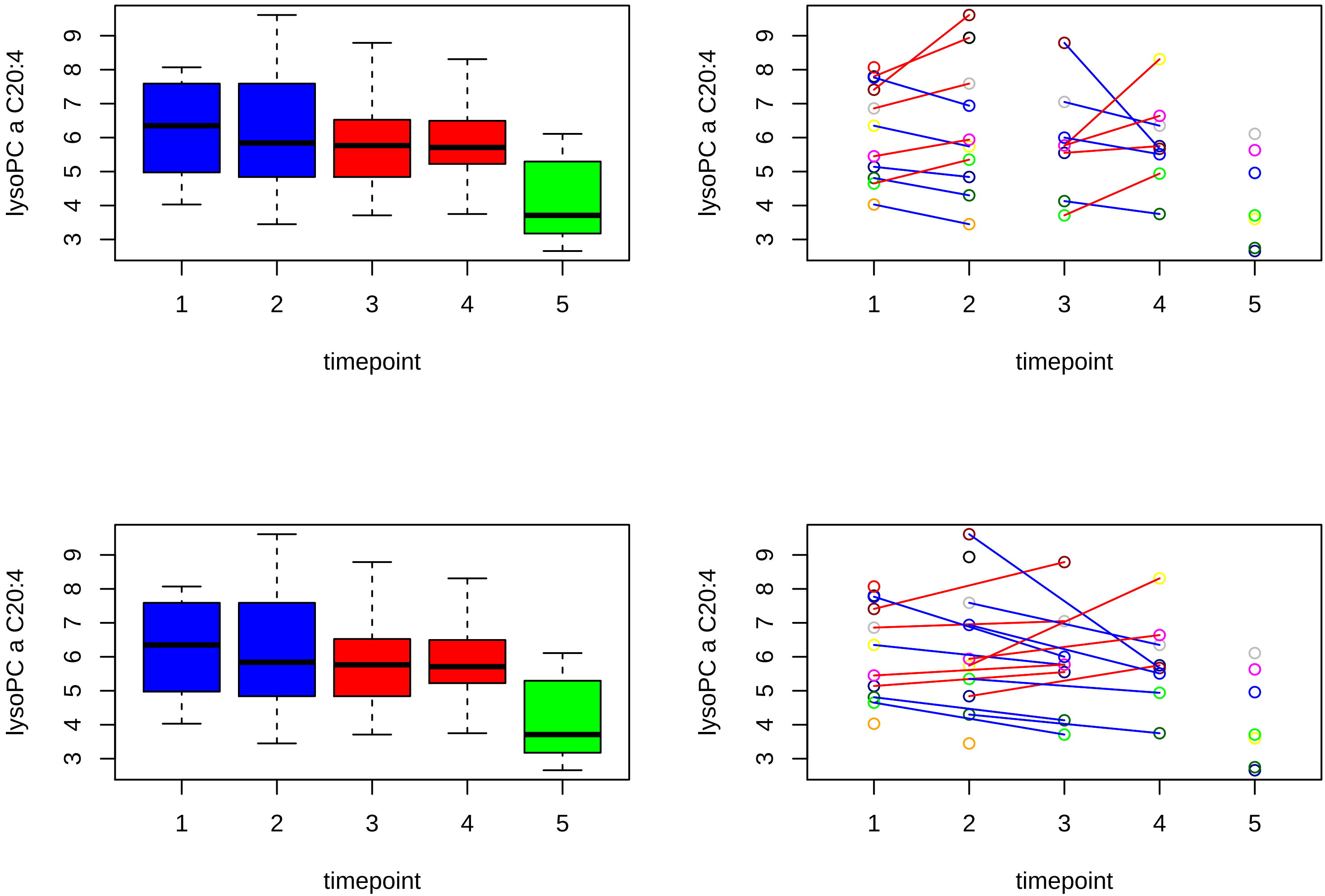
lysoPC a C20:4
timepoint
lysoPC a C20:4
timepoint
lysoPC a C20:4
timepoint
lysoPC a C20:4
timepoint

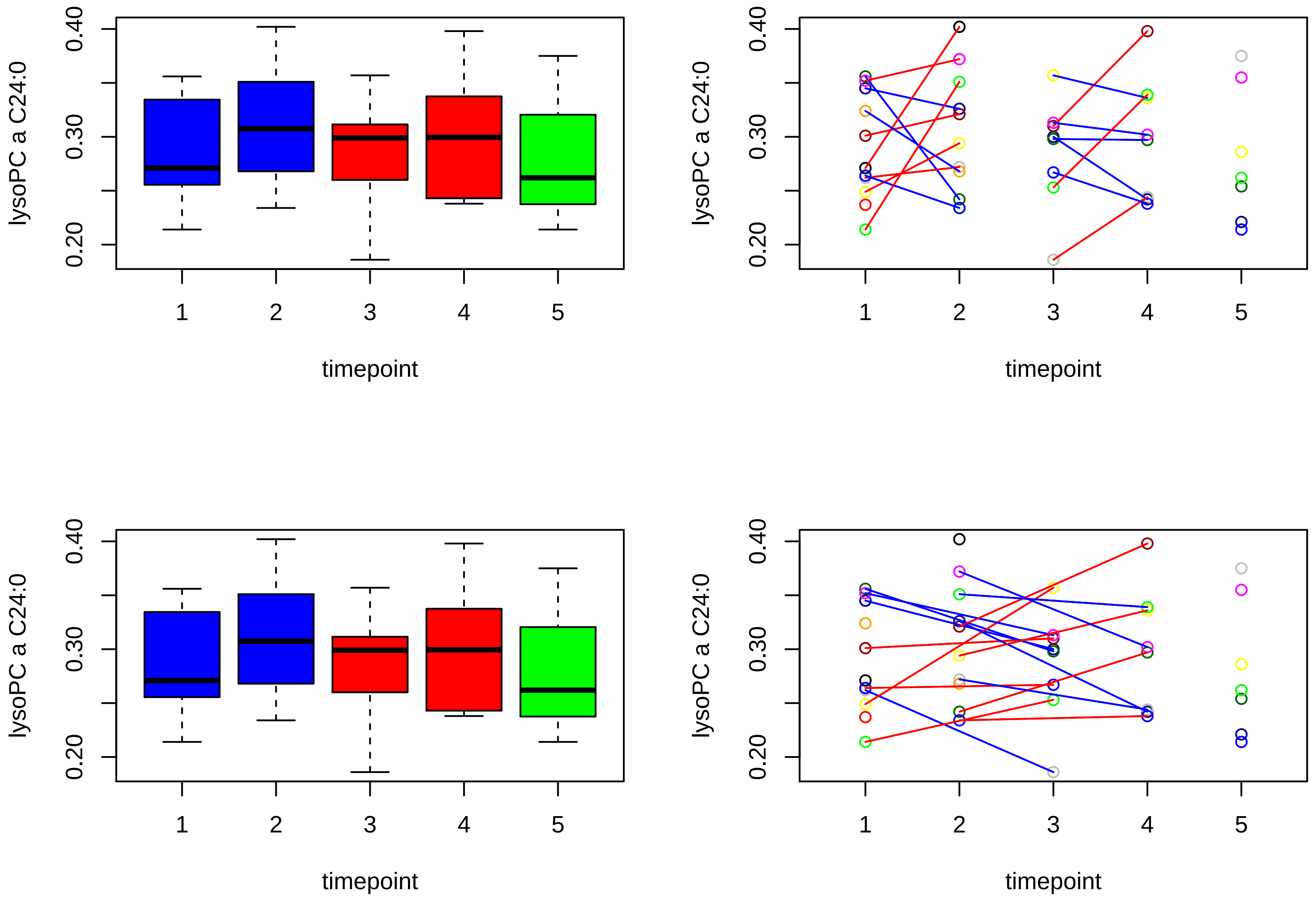

lysoPC a C24:0
0.20
0.30
0.40
1
2
3
4
5
timepoint

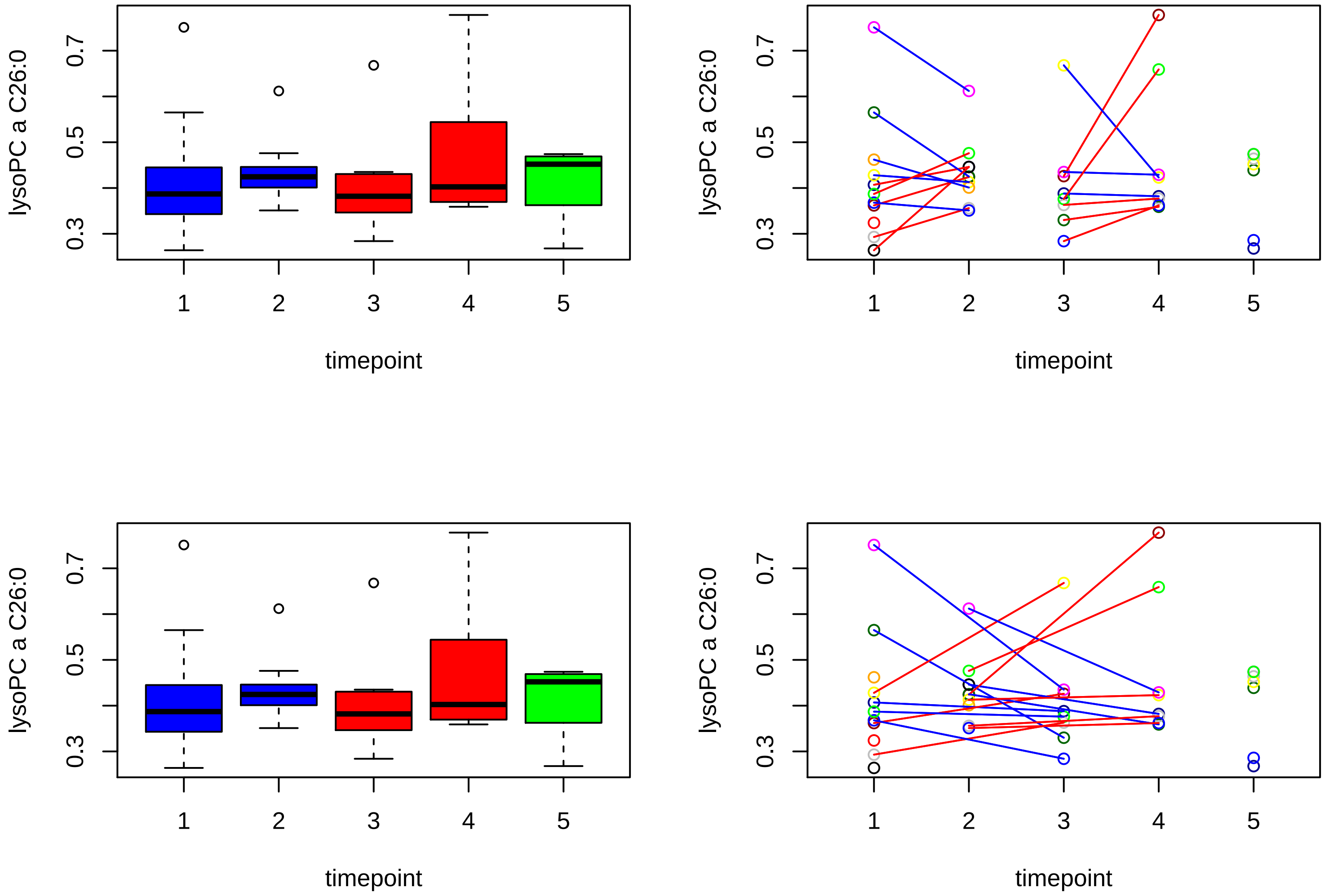
lysoPC a C26:0
0.3
0.5
0.7
1
2
3
4
5
timepoint
lysoPC a C26:0
0.3
0.5
0.7
1
2
3
4
5
timepoint
lysoPC a C26:0
0.3
0.5
0.7
1
2
3
4
5
timepoint
lysoPC a C26:0
0.3
0.5
0.7
1
2
3
4
5
timepoint

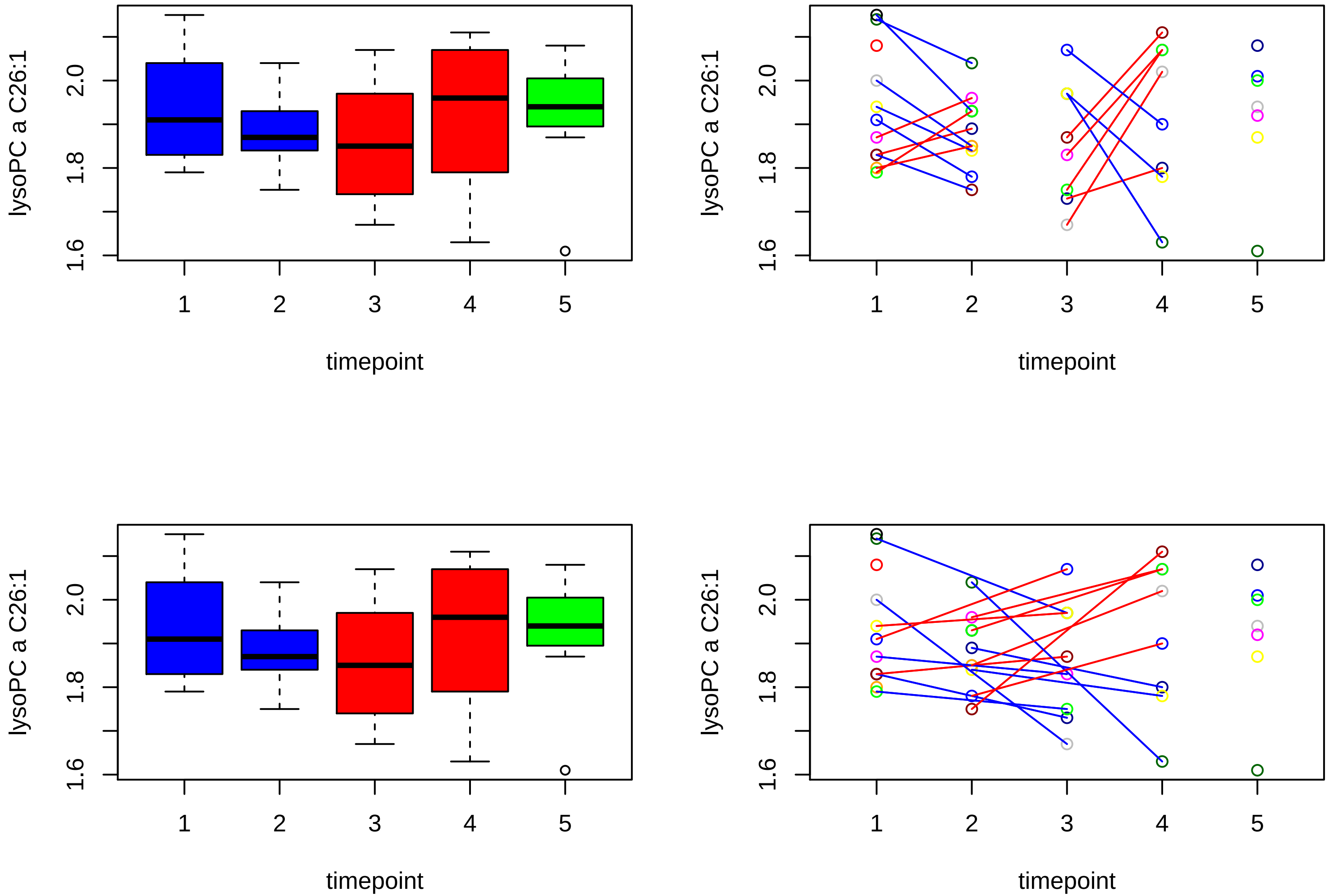

lysoPC a C26:1
1.6
1.8
2.0
1
2
3
4
5
timepoint
lysoPC a C26:1
1.6
1.8
2.0
1
2
3
4
5
timepoint
lysoPC a C26:1
1.6
1.8
2.0
1
2
3
4
5
timepoint
lysoPC a C26:1
1.6
1.8
2.0
1
2
3
4
5
timepoint

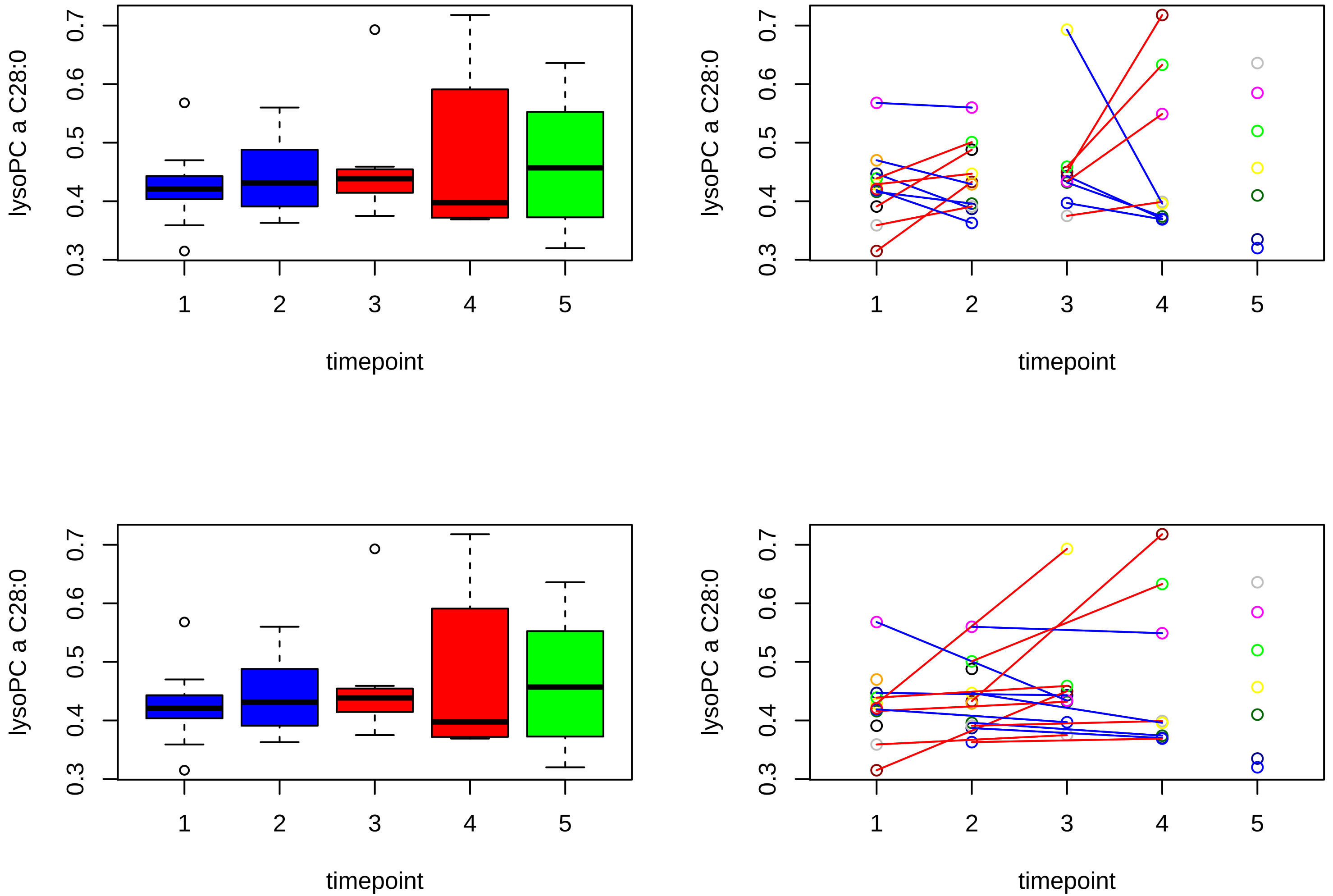
lysoPC a C28:0
timepoint
0.3
0.4
0.5
0.6
0.7
1
2
3
4
5

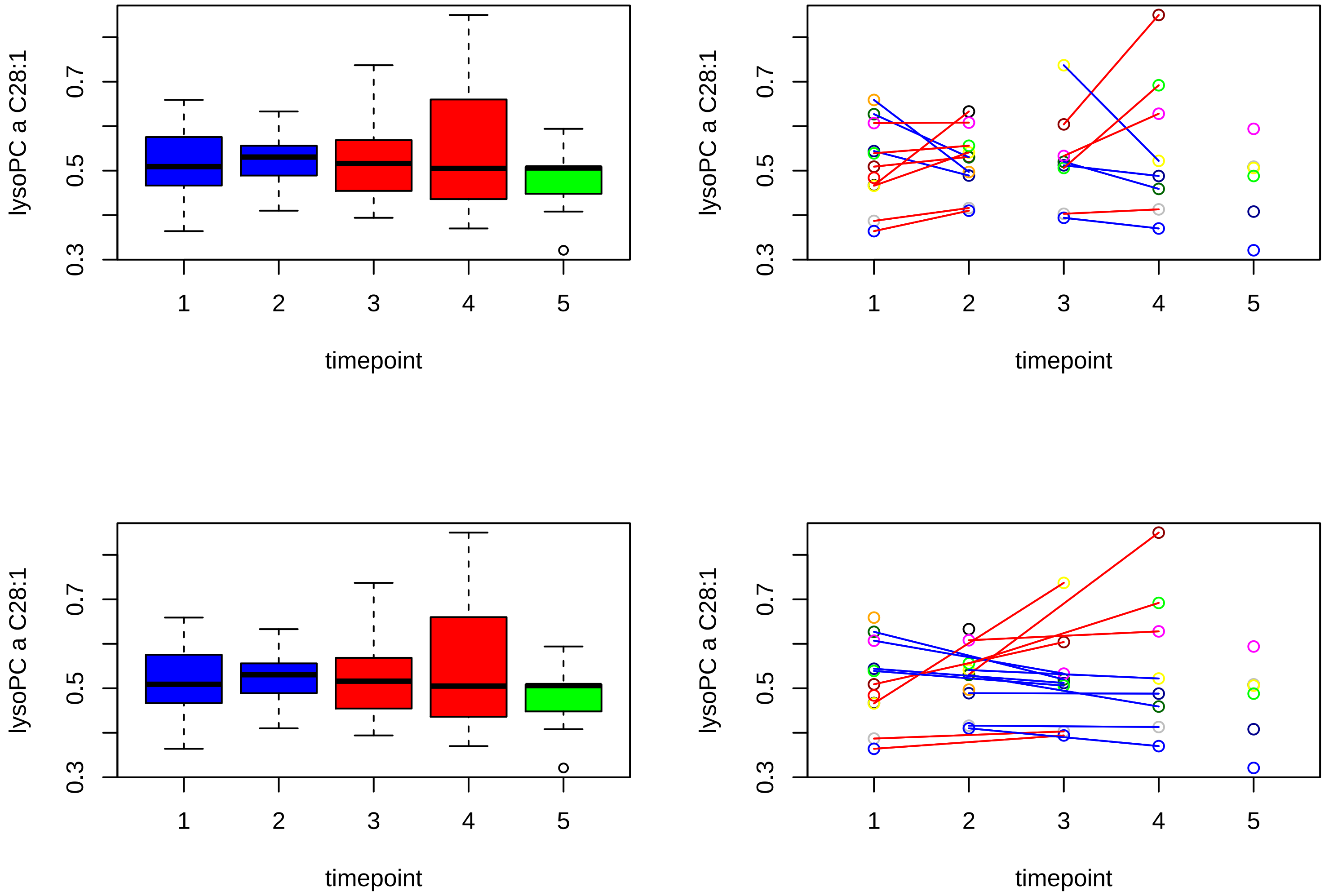

lysoPC a C28:1
0.3
0.5
0.7
1
2
3
4
5
timepoint
lysoPC a C28:1
0.3
0.5
0.7
1
2
3
4
5
timepoint
lysoPC a C28:1
0.3
0.5
0.7
1
2
3
4
5
timepoint
lysoPC a C28:1
0.3
0.5
0.7
1
2
3
4
5
timepoint

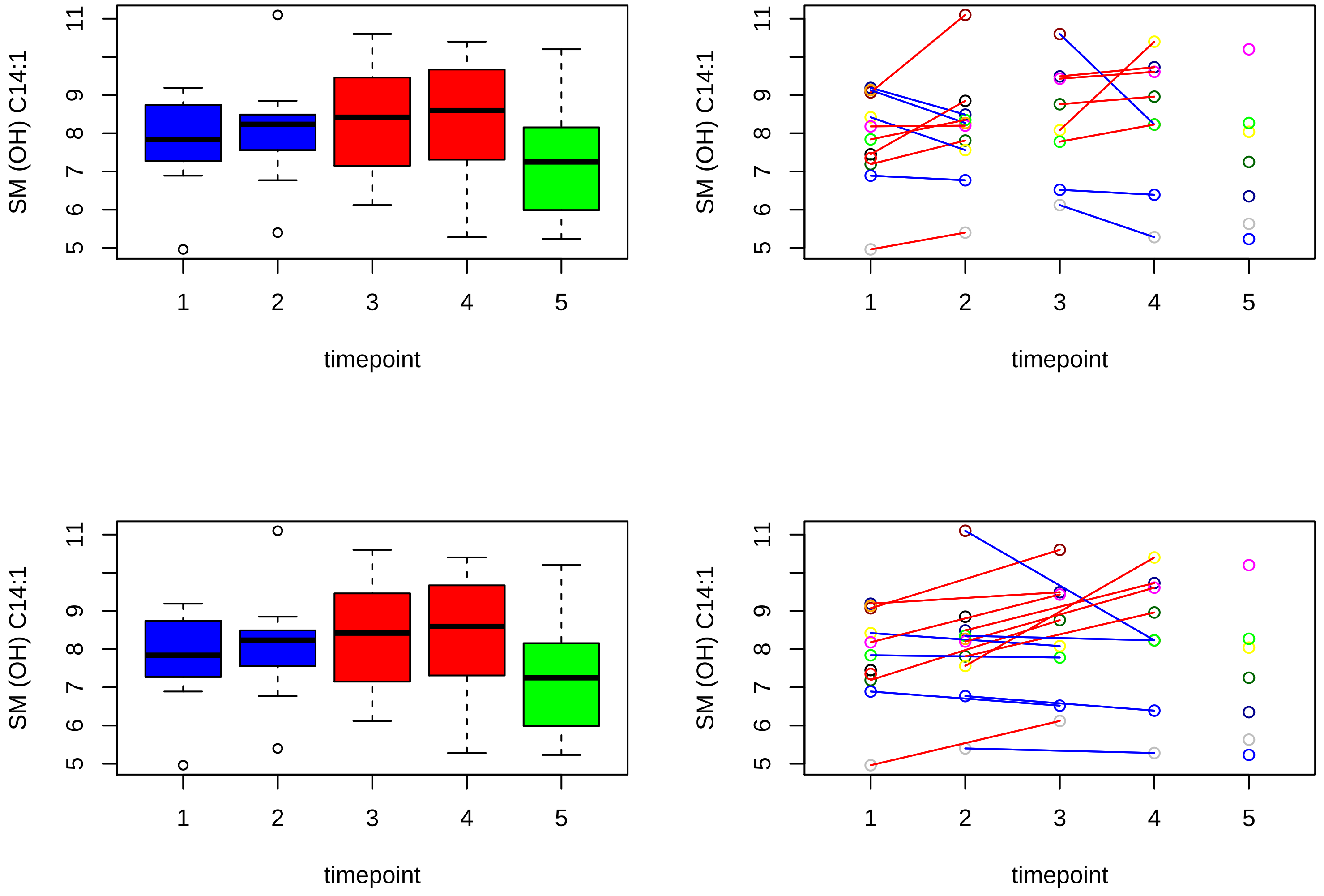
SM (OH) C14:1
timepoint
5 6 7 8 9 11
1 2 3 4 5

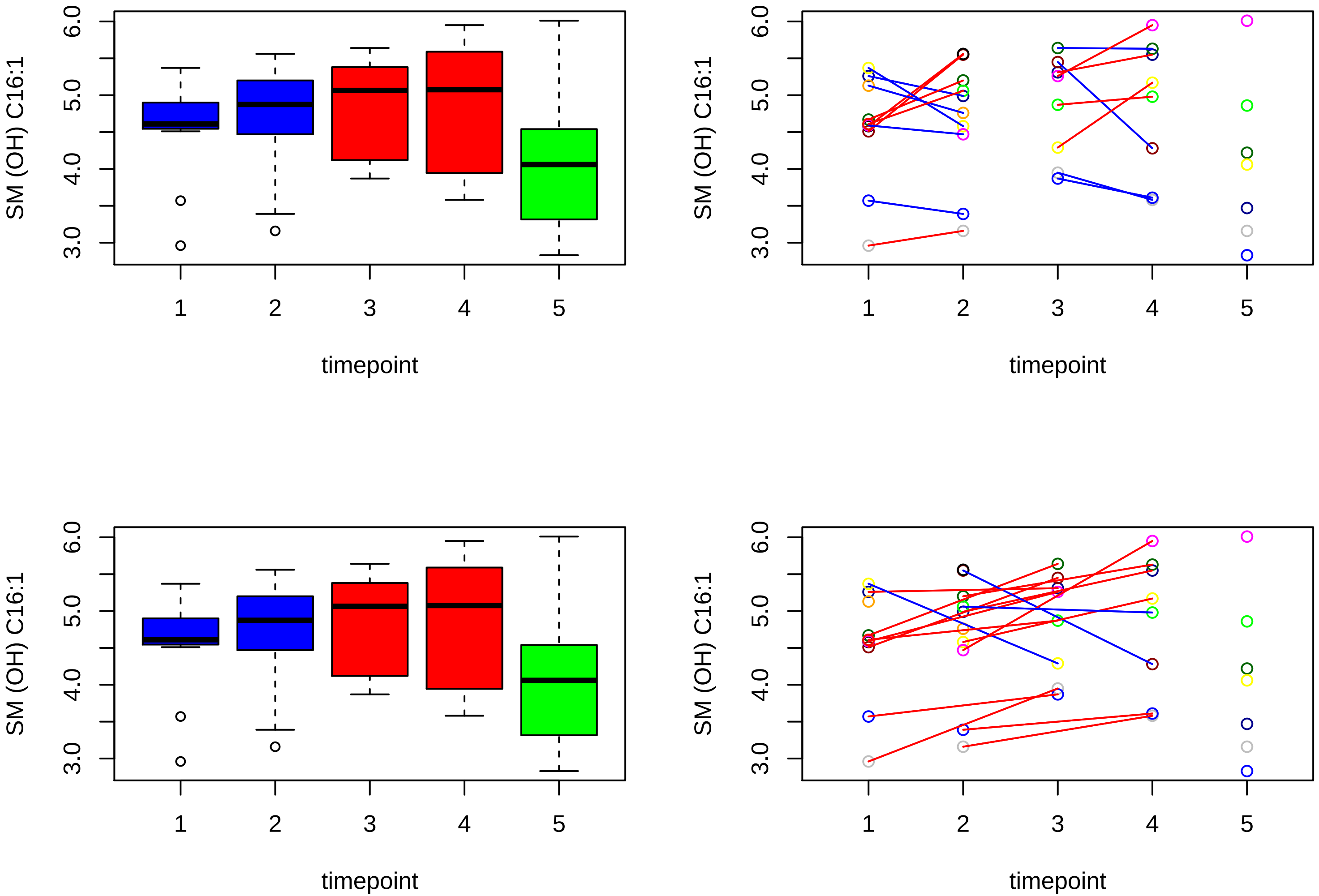

SM (OH) C16:1
timepoint
3.0
4.0
5.0
6.0
1
2
3
4
5

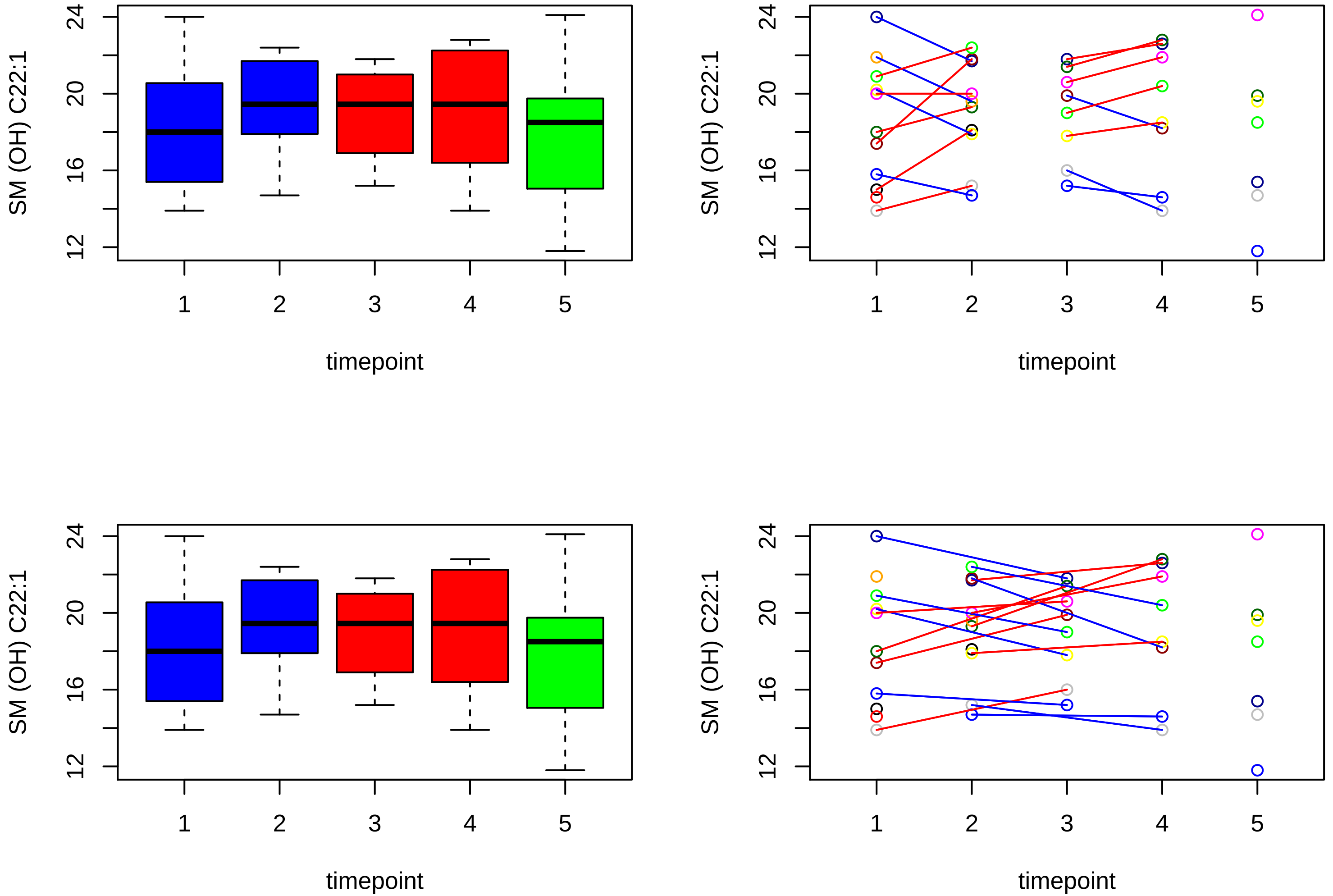
SM (OH) C22:1
12
16
20
24
1
2
3
4
5
timepoint
SM (OH) C22:1
12
16
20
24
1
2
3
4
5
timepoint
SM (OH) C22:1
12
16
20
24
1
2
3
4
5
timepoint
SM (OH) C22:1
12
16
20
24
1
2
3
4
5
timepoint

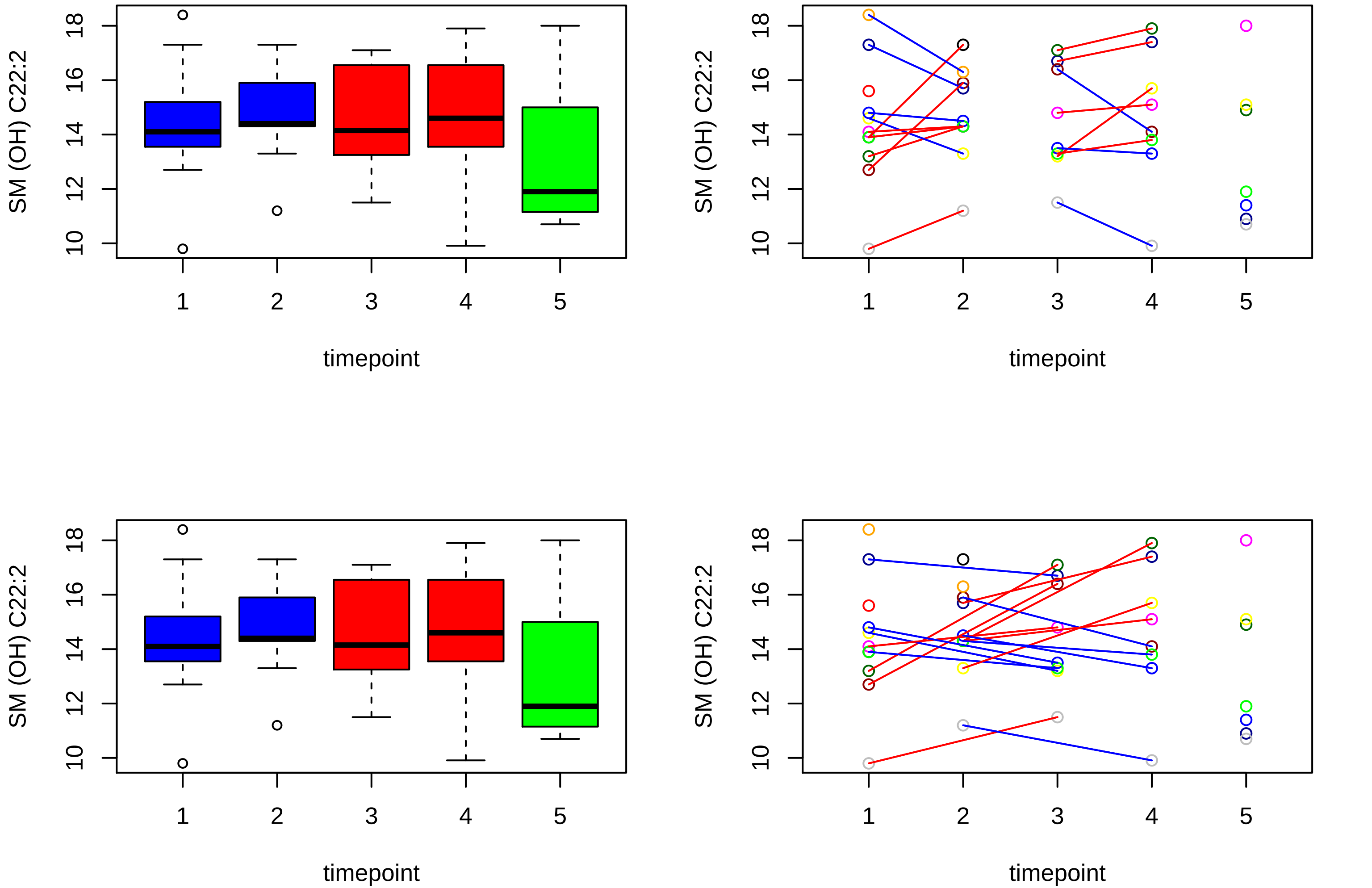

SM (OH) C22:2
10
12
14
16
18
1
2
3
4
5
timepoint

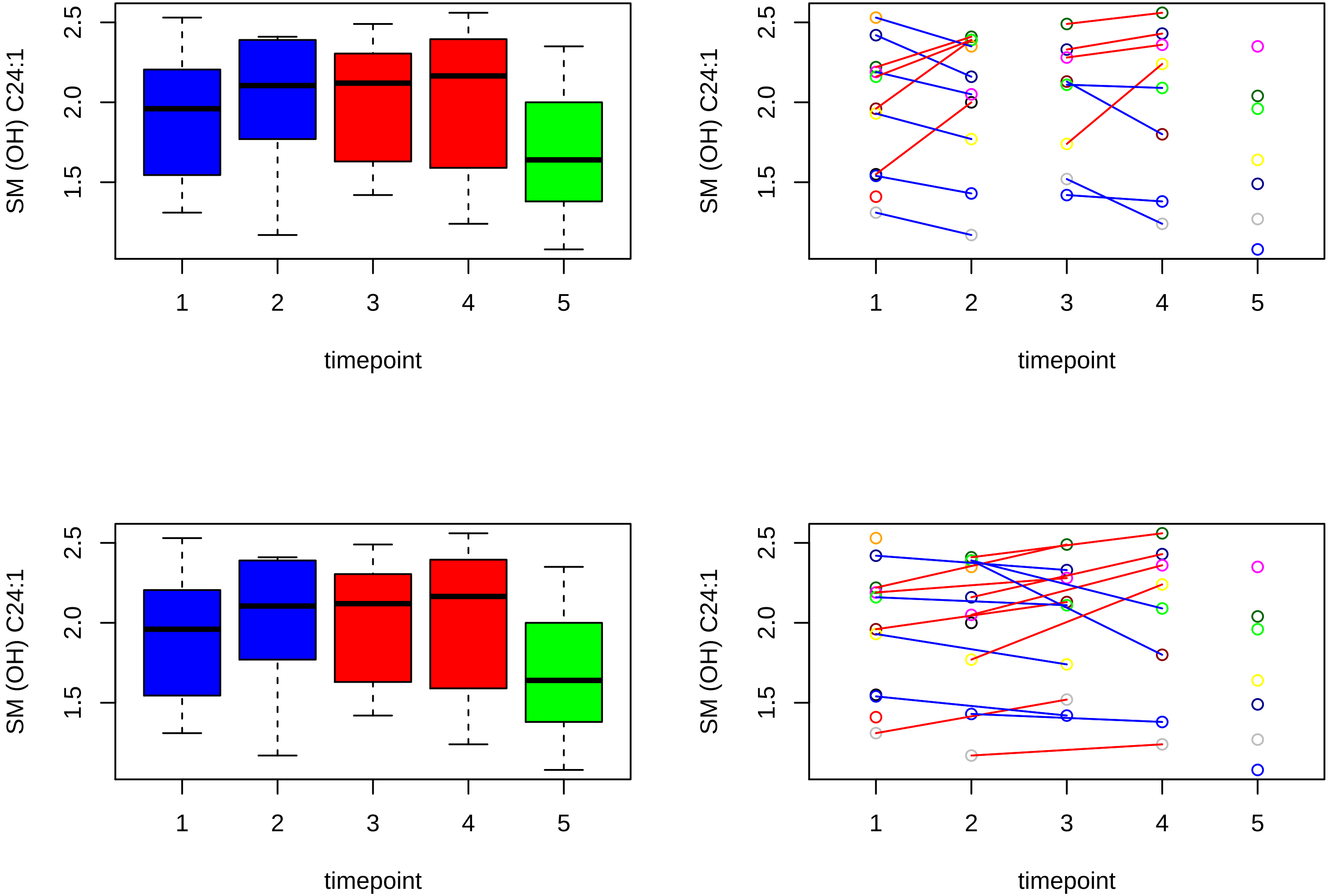
SM (OH) C24:1
timepoint
1.5
2.0
2.5
1
2
3
4
5

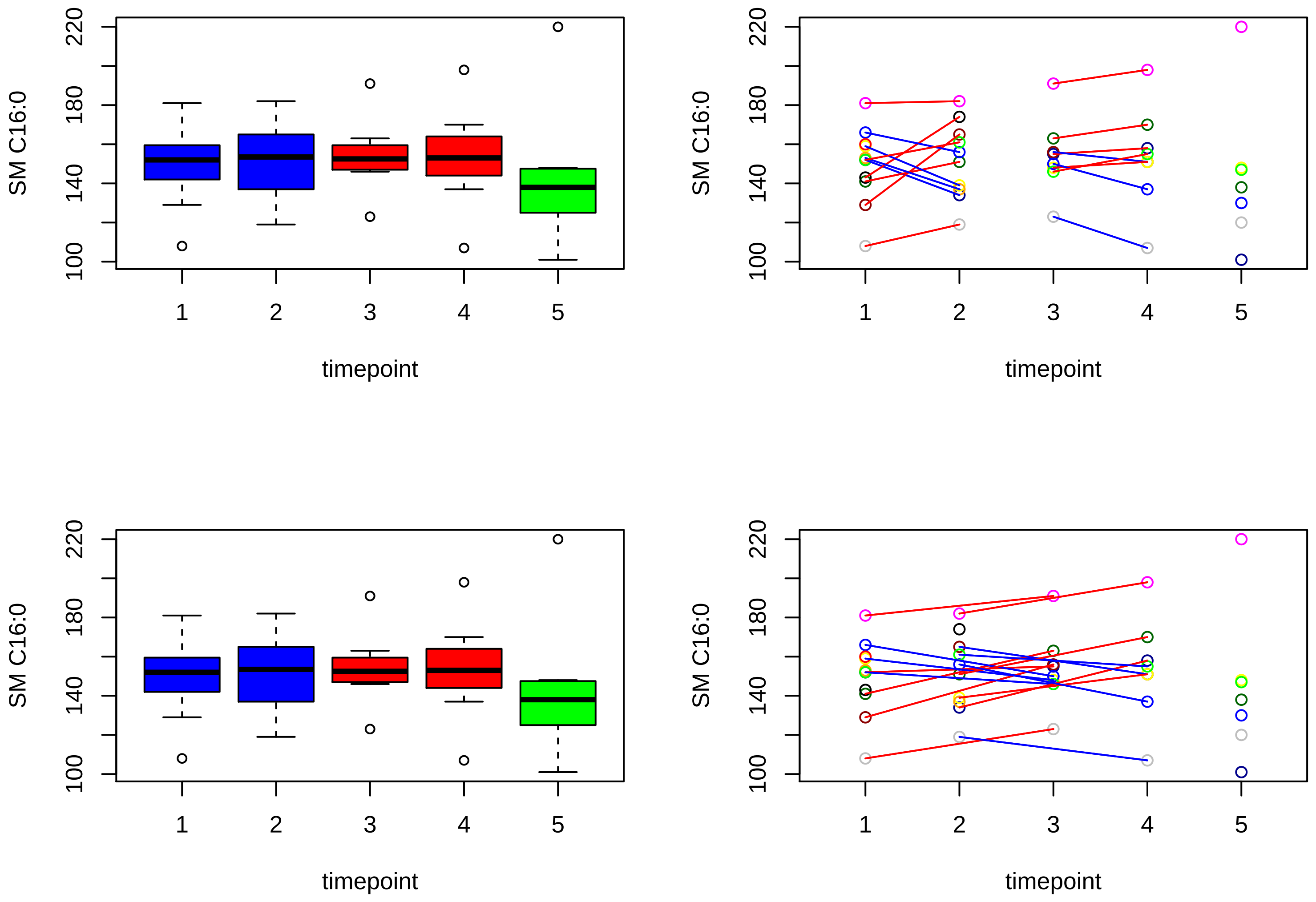

SM C16:0
100
140
180
220
1
2
3
4
5
timepoint

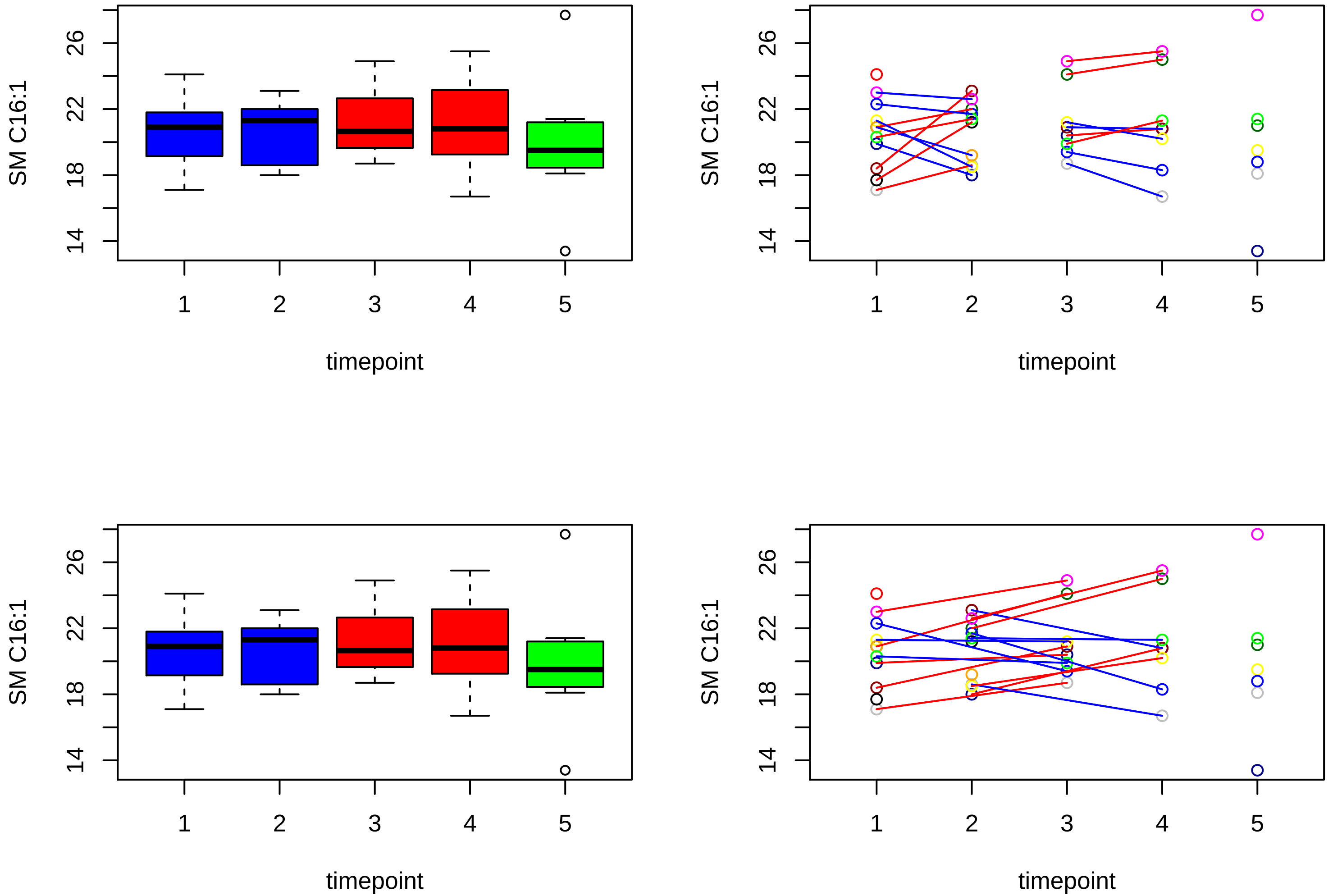

SM C16:1
14
18
22
26
1
2
3
4
5
timepoint
SM C16:1
14
18
22
26
1
2
3
4
5
timepoint
SM C16:1
14
18
22
26
1
2
3
4
5
timepoint
SM C16:1
14
18
22
26
1
2
3
4
5
timepoint

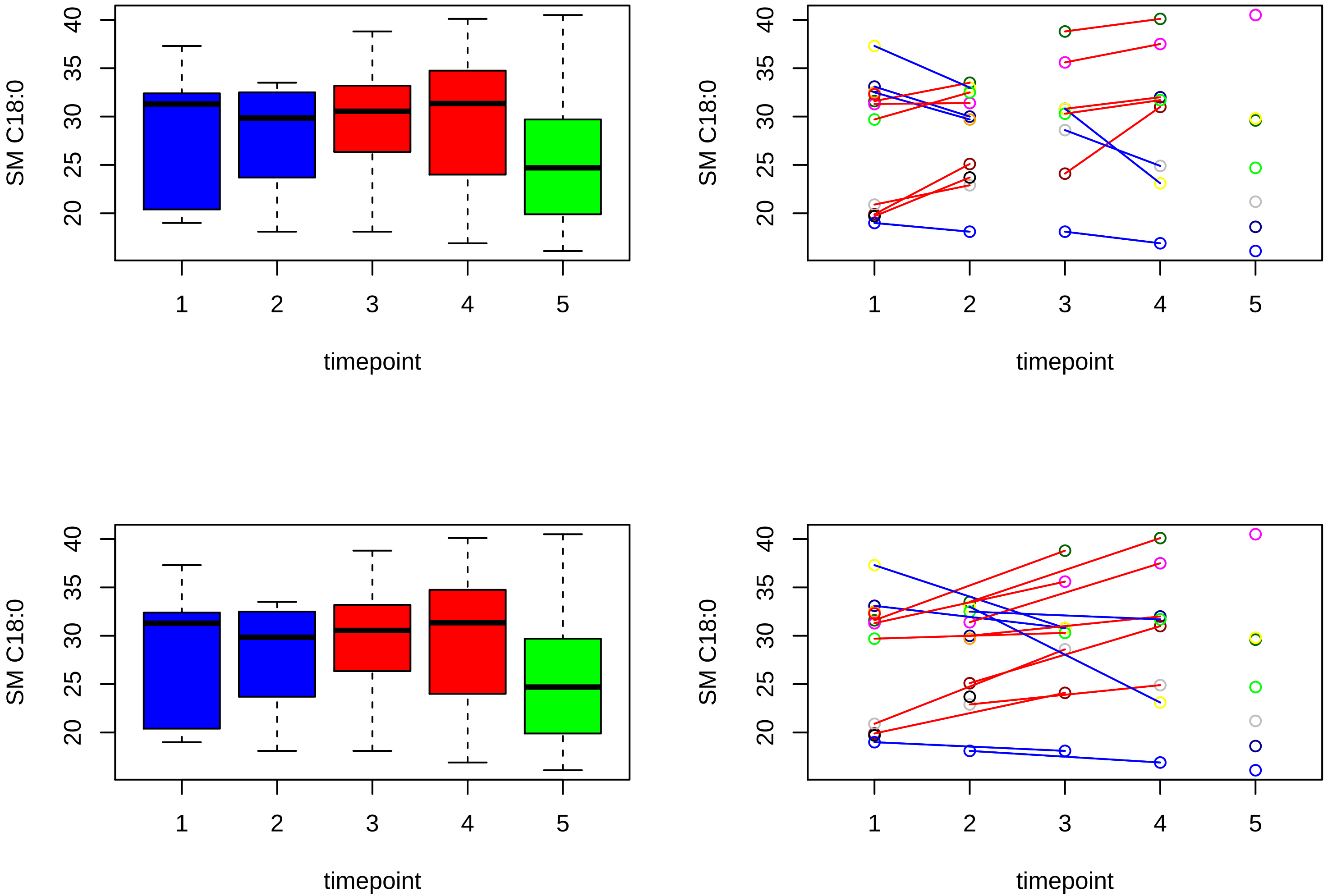
SM C18:0
20
25
30
35
40
1
2
3
4
5
timepoint

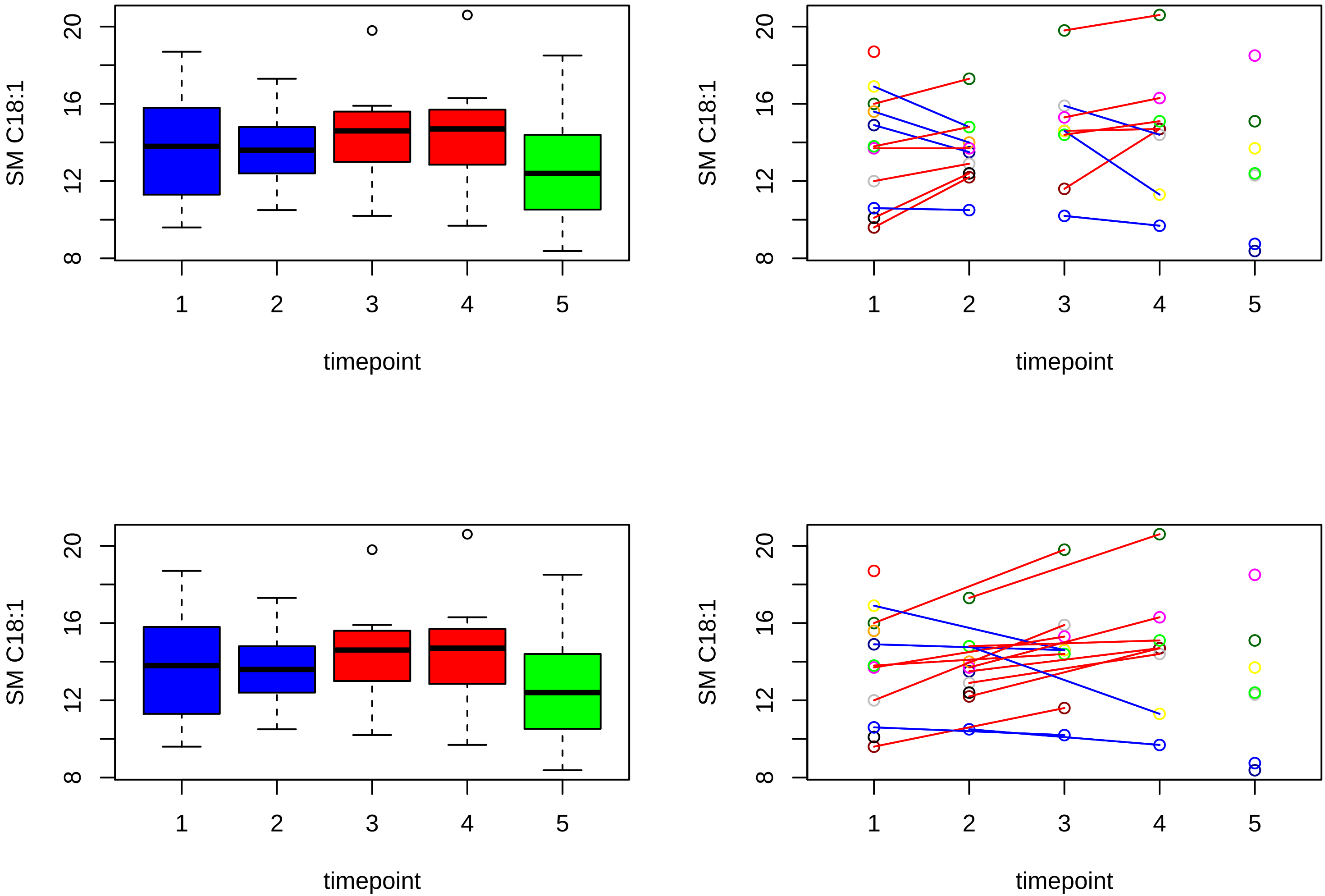

SM C18:1
timepoint
SM C18:1
timepoint
SM C18:1
timepoint
SM C18:1
timepoint

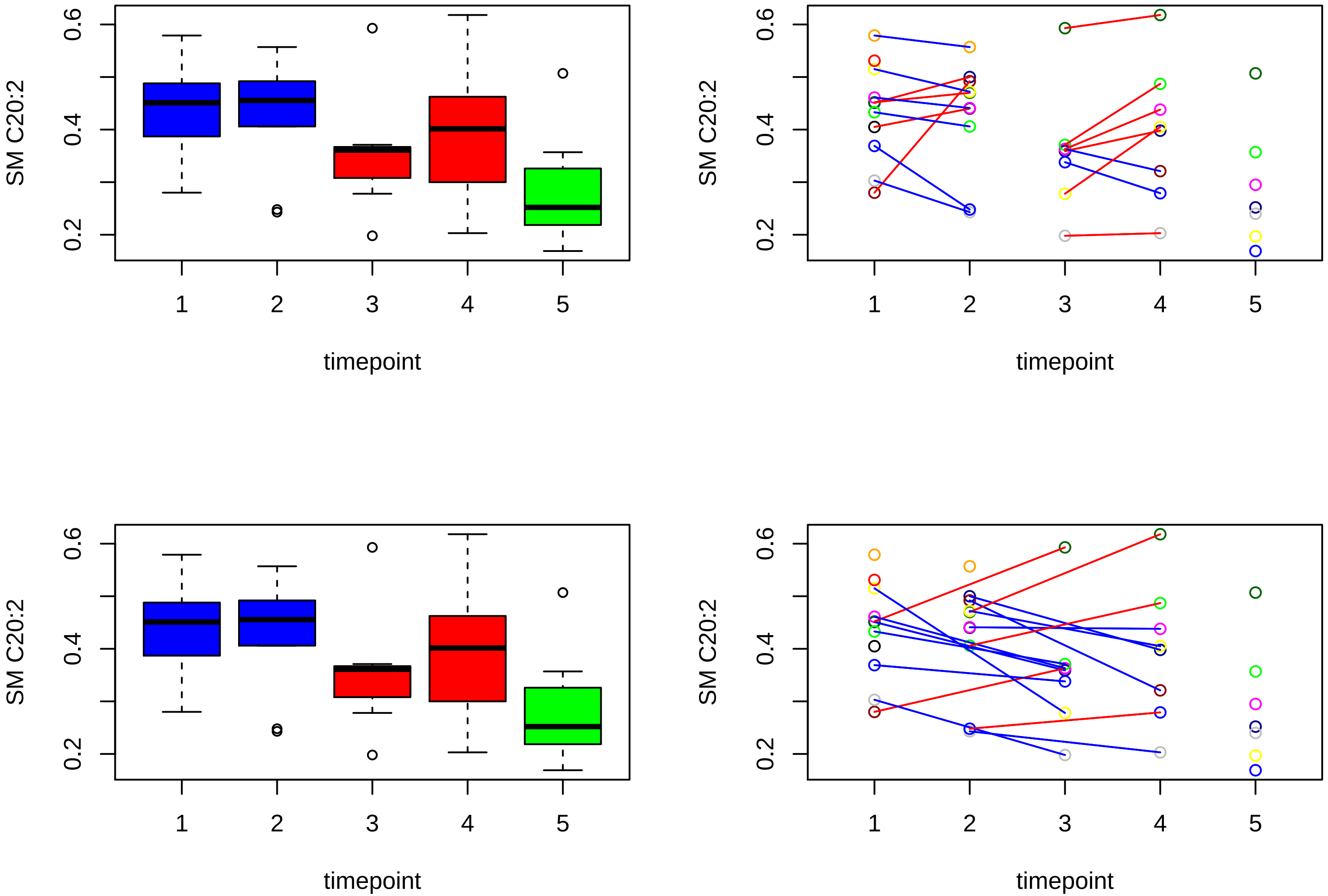

SM C20:2
0.2
0.4
0.6
1
2
3
4
5
timepoint

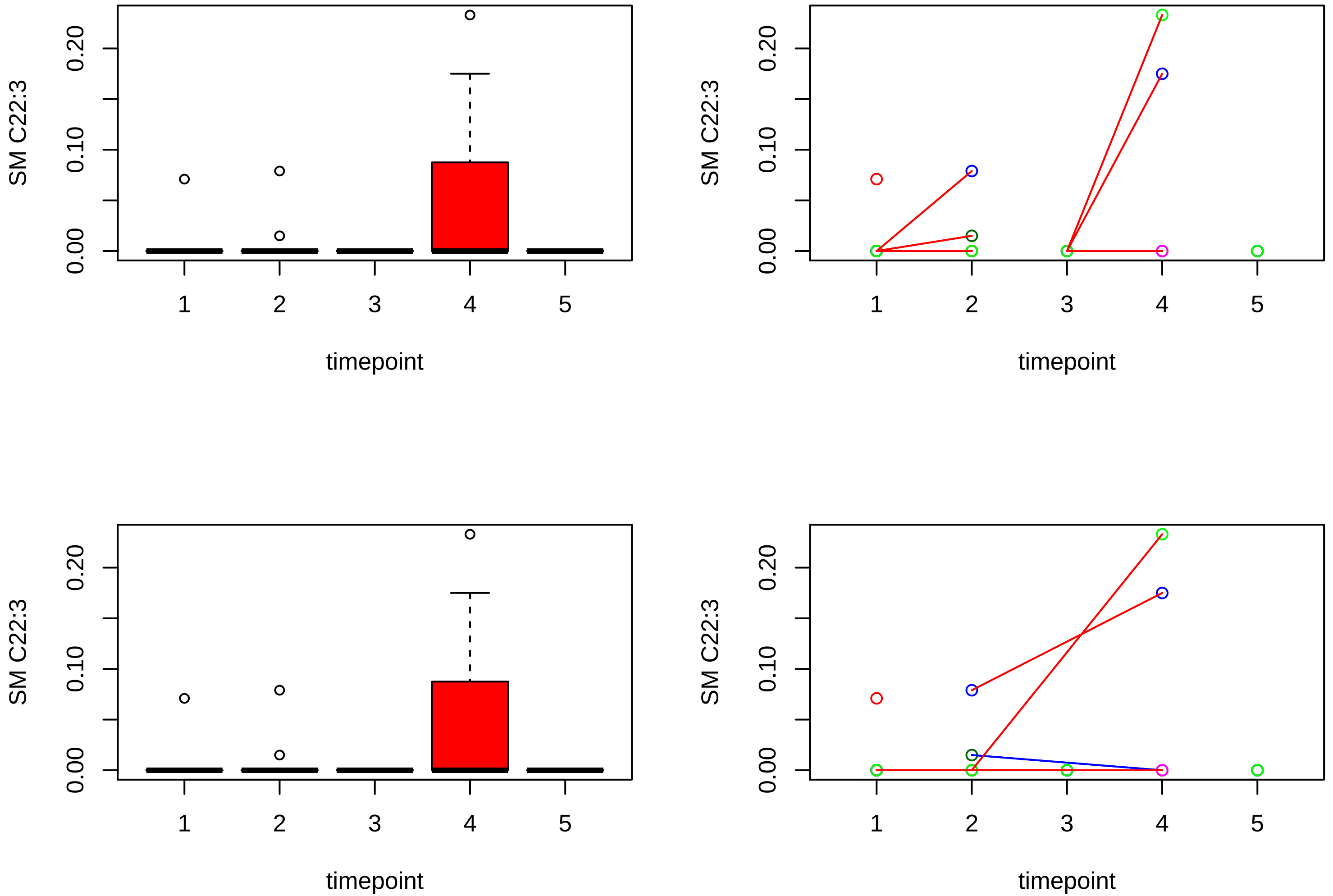

SM C22:3
0.00
0.10
0.20
1
2
3
4
5
timepoint

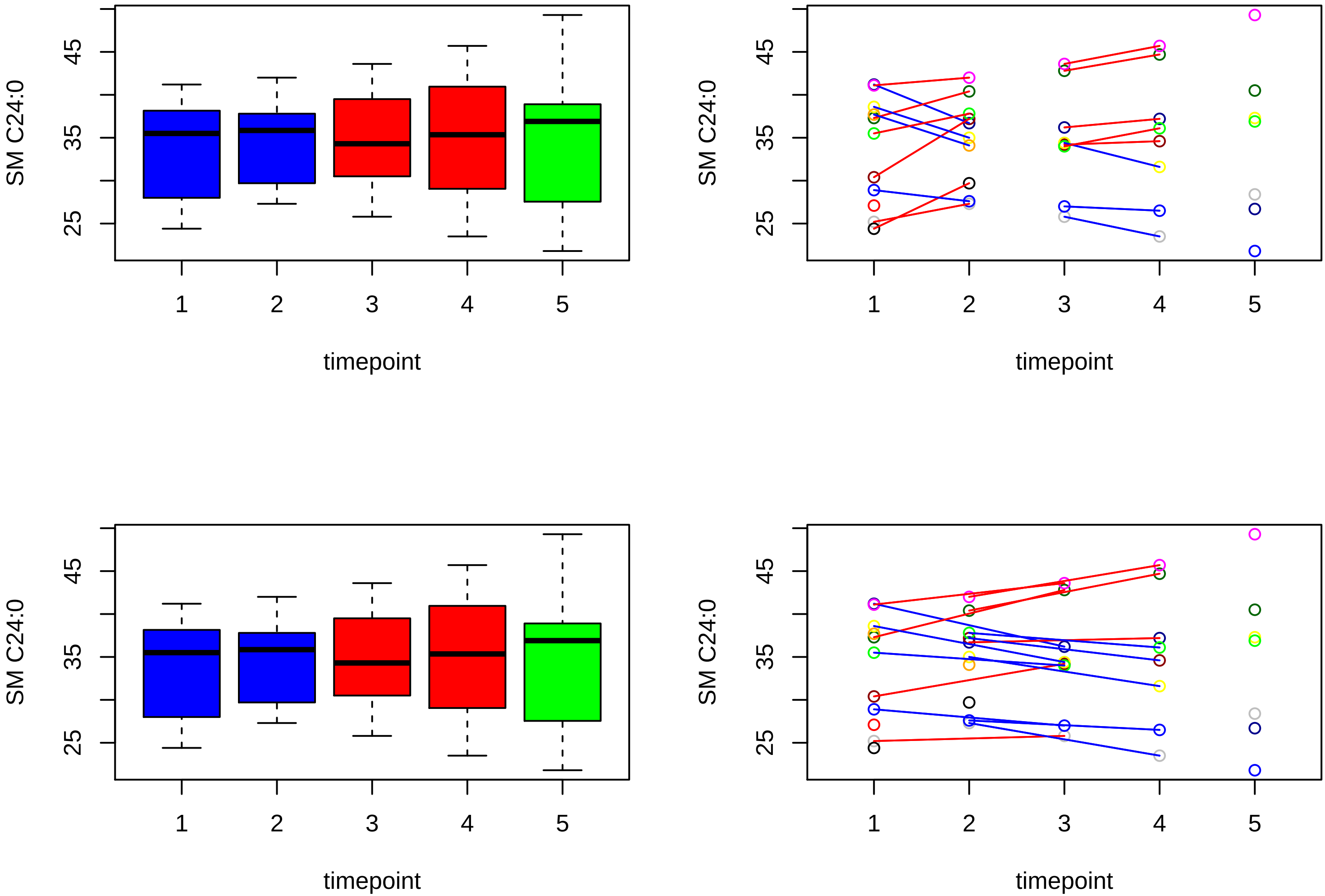

SM C24:0
timepoint

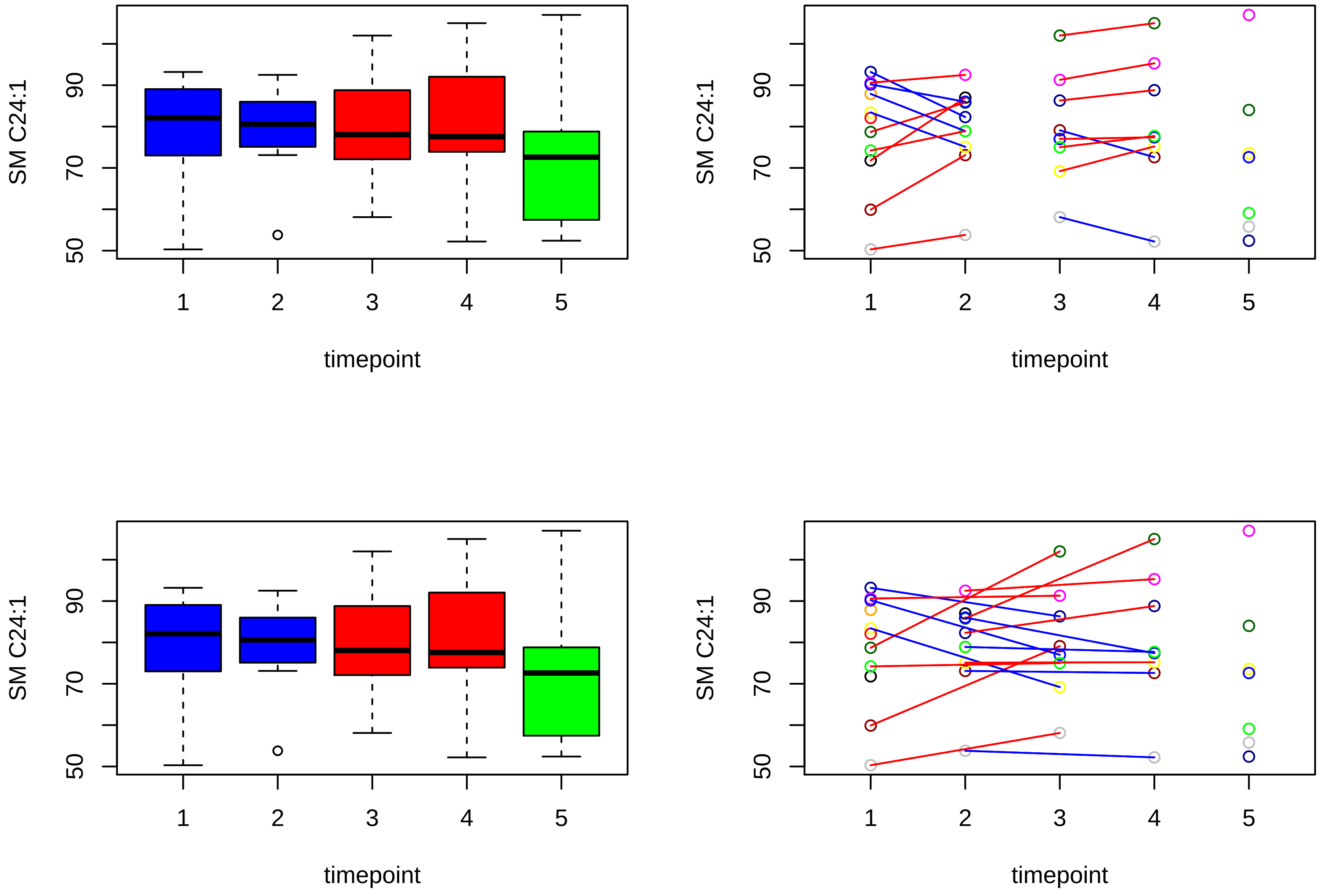

SM C24:1
50
70
90
1
2
3
4
5
timepoint

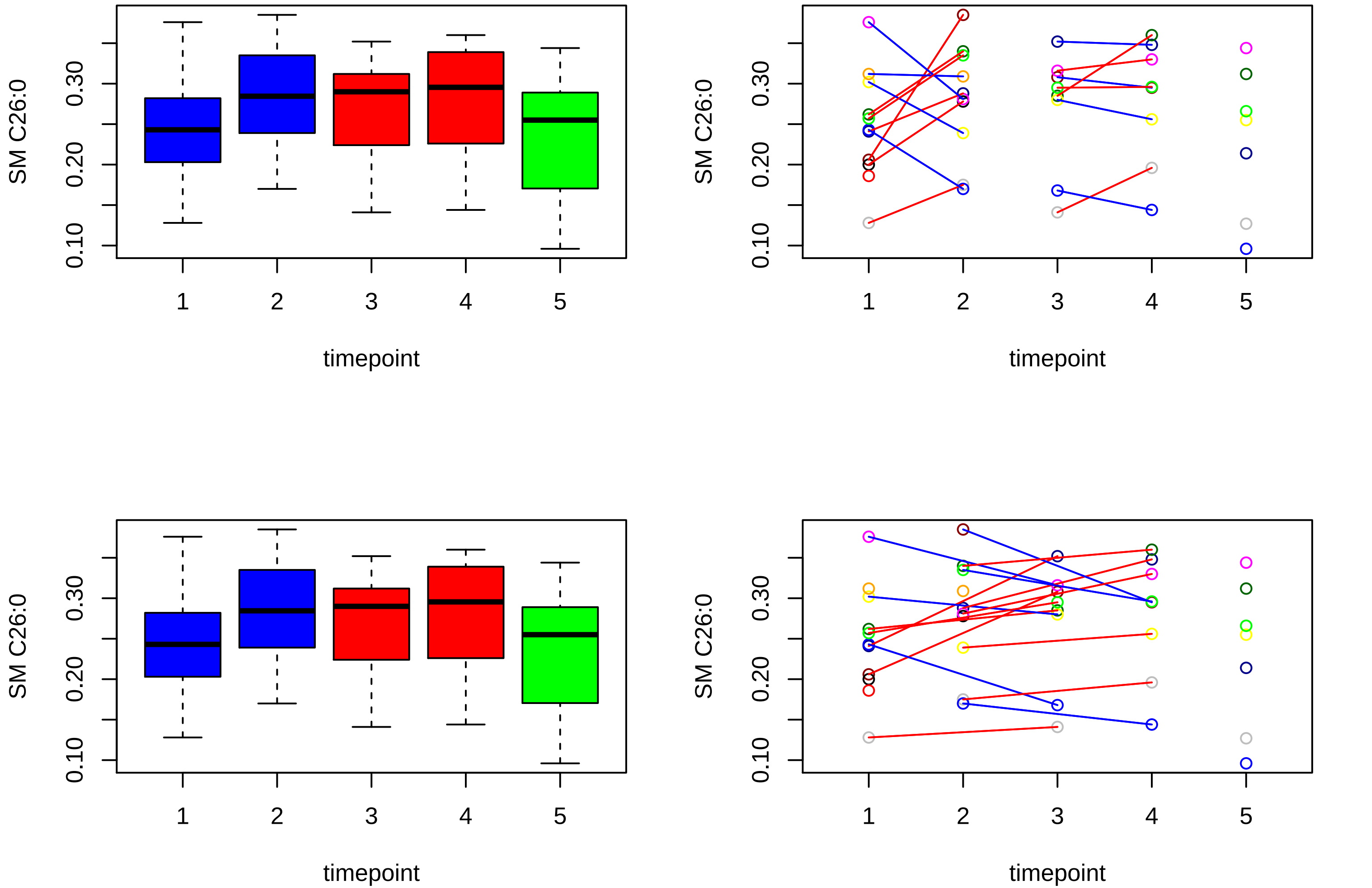

SM C26:0
0.10
0.20
0.30
1
2
3
4
5
timepoint
SM C26:0
0.10
0.20
0.30
1
2
3
4
5
timepoint
SM C26:0
0.10
0.20
0.30
1
2
3
4
5
timepoint
SM C26:0
0.10
0.20
0.30
1
2
3
4
5
timepoint

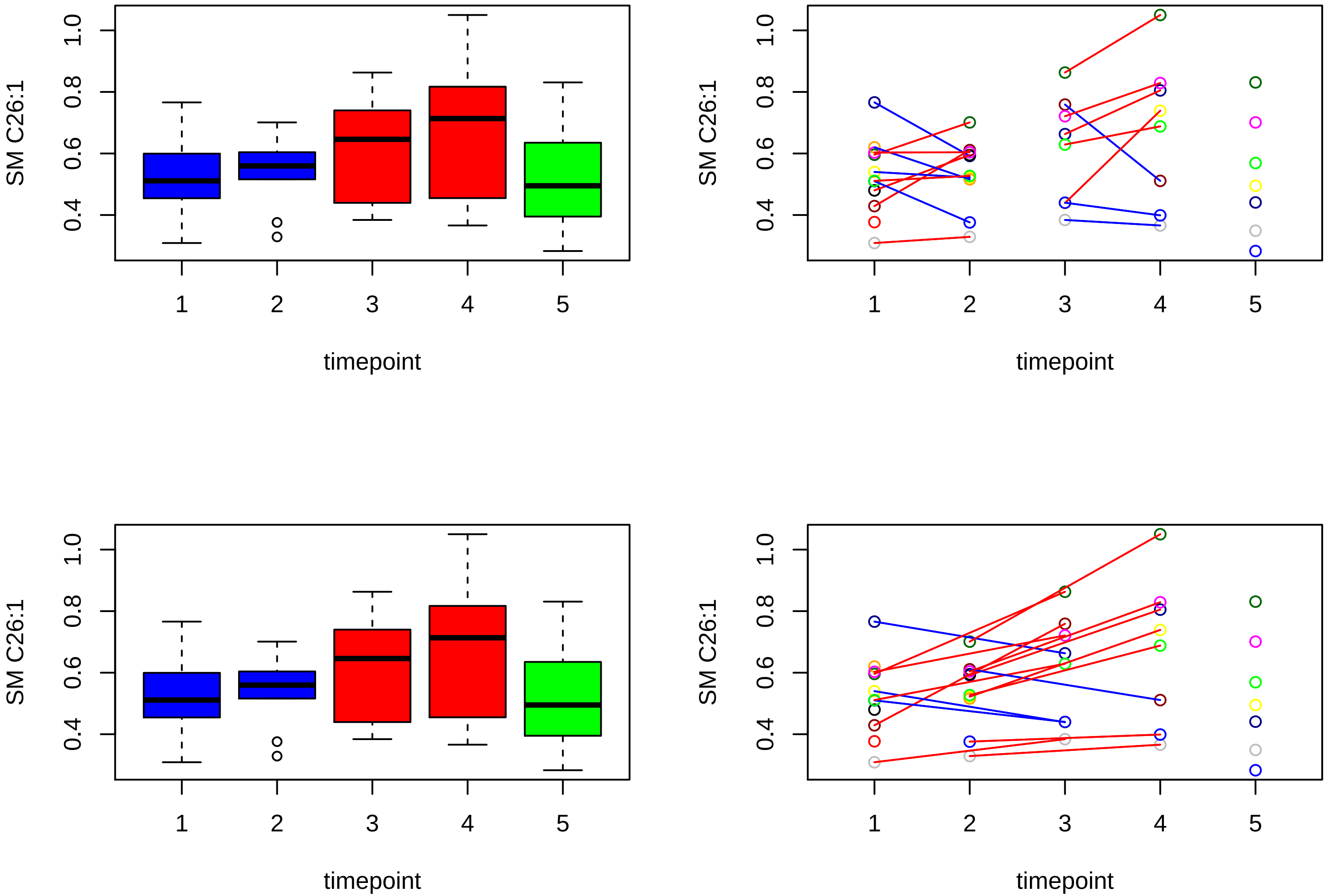

SM C26:1
0.4
0.6
0.8
1.0
1
2
3
4
5
timepoint

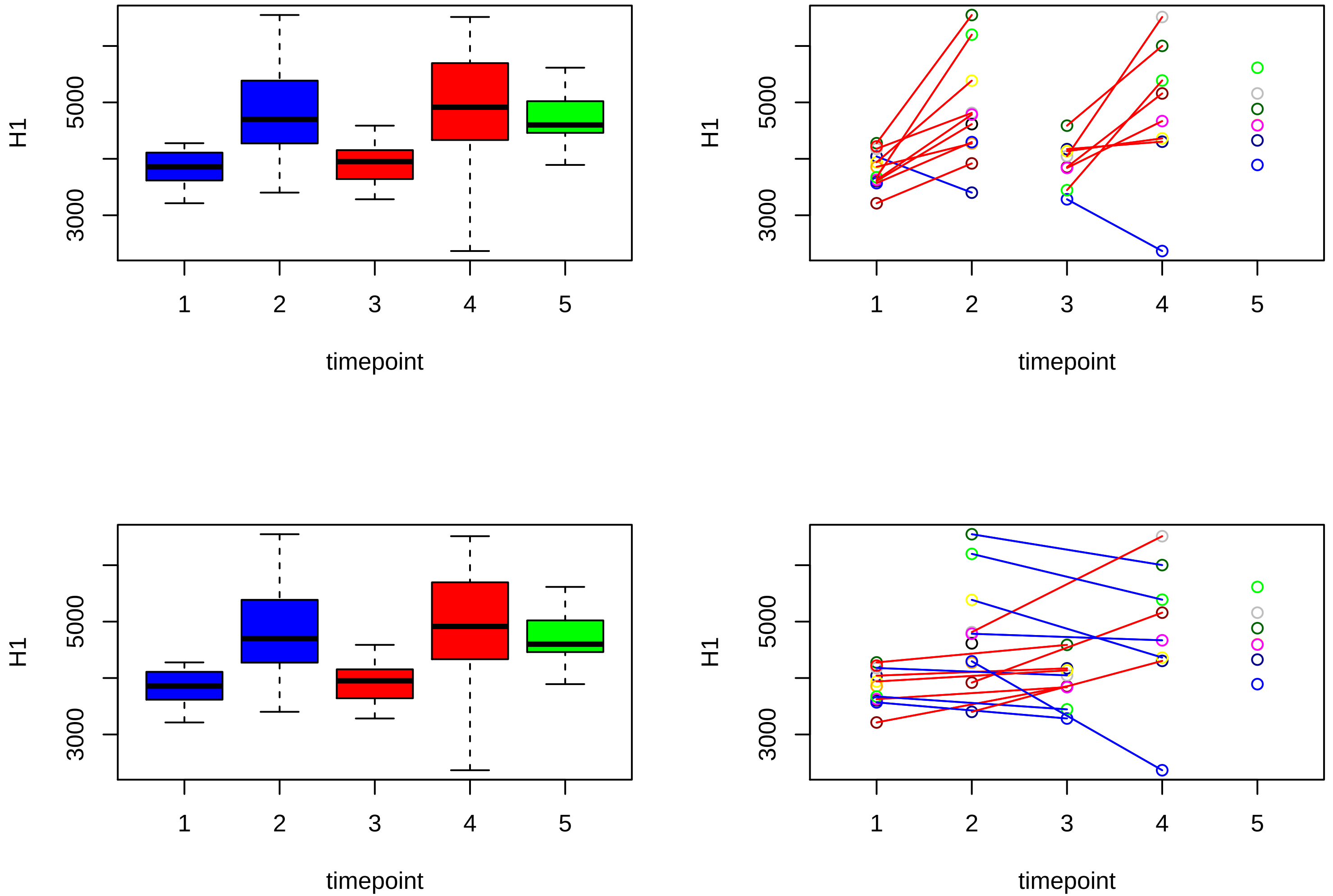
H1
3000
5000
1
2
3
4
5
timepoint
H1
3000
5000
1
2
3
4
5
timepoint
H1
3000
5000
1
2
3
4
5
timepoint
H1
3000
5000
1
2
3
4
5
timepoint

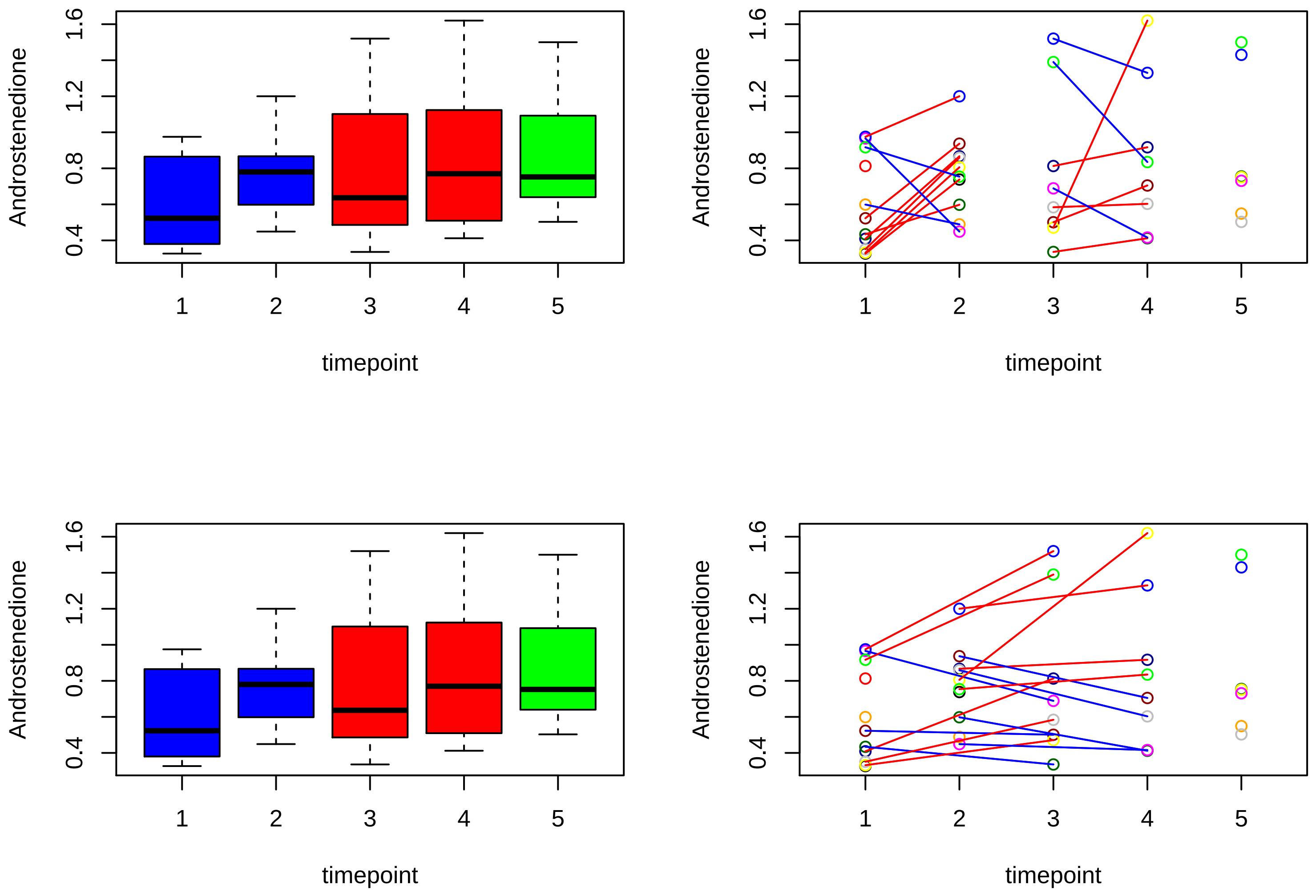
Androstenedione
0.4
0.8
1.2
1.6
1
2
3
4
5
timepoint

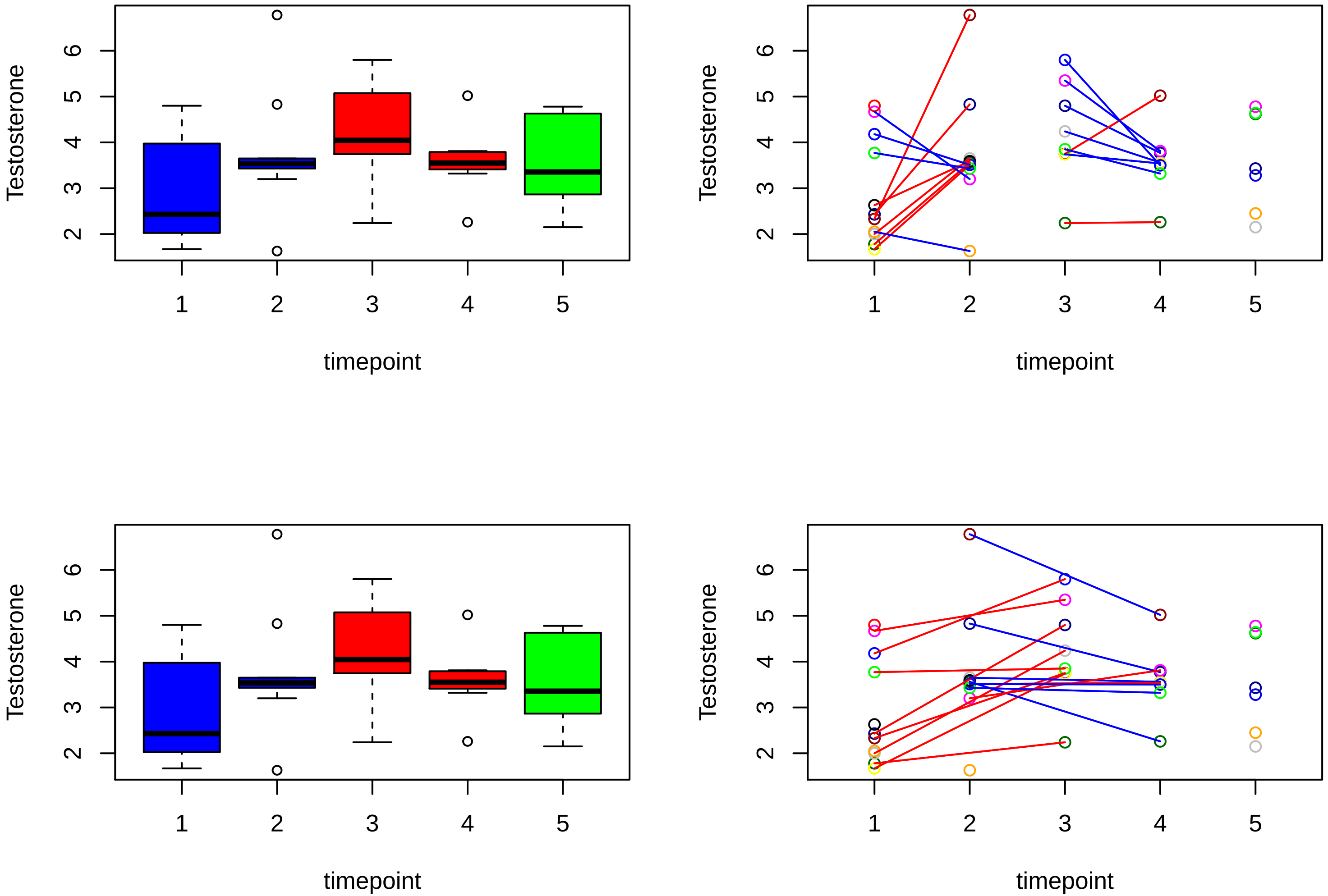

Testosterone
timepoint
Testosterone
timepoint
Testosterone
timepoint
Testosterone
timepoint

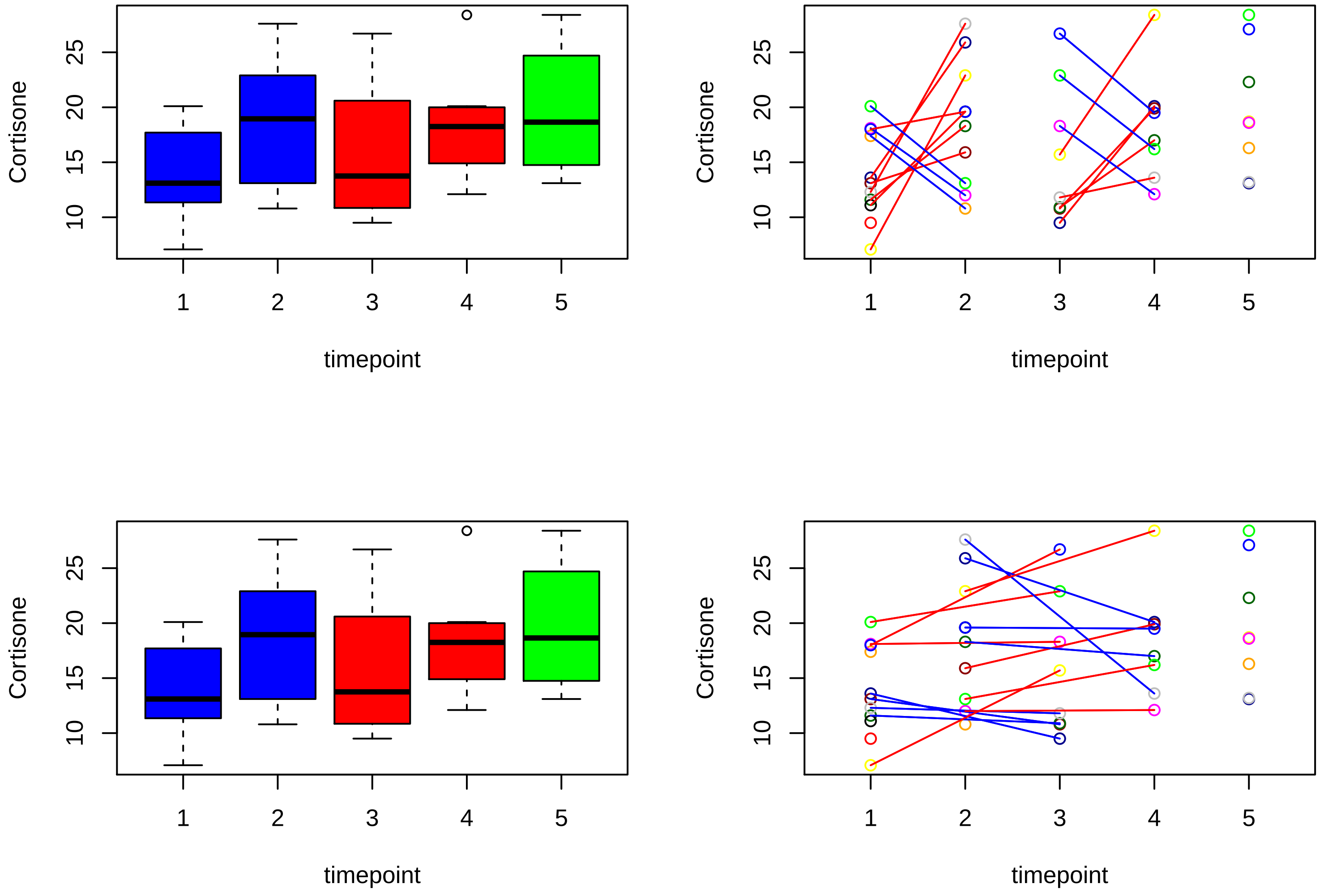

Cortisone
10
15
20
25
1
2
3
4
5
timepoint

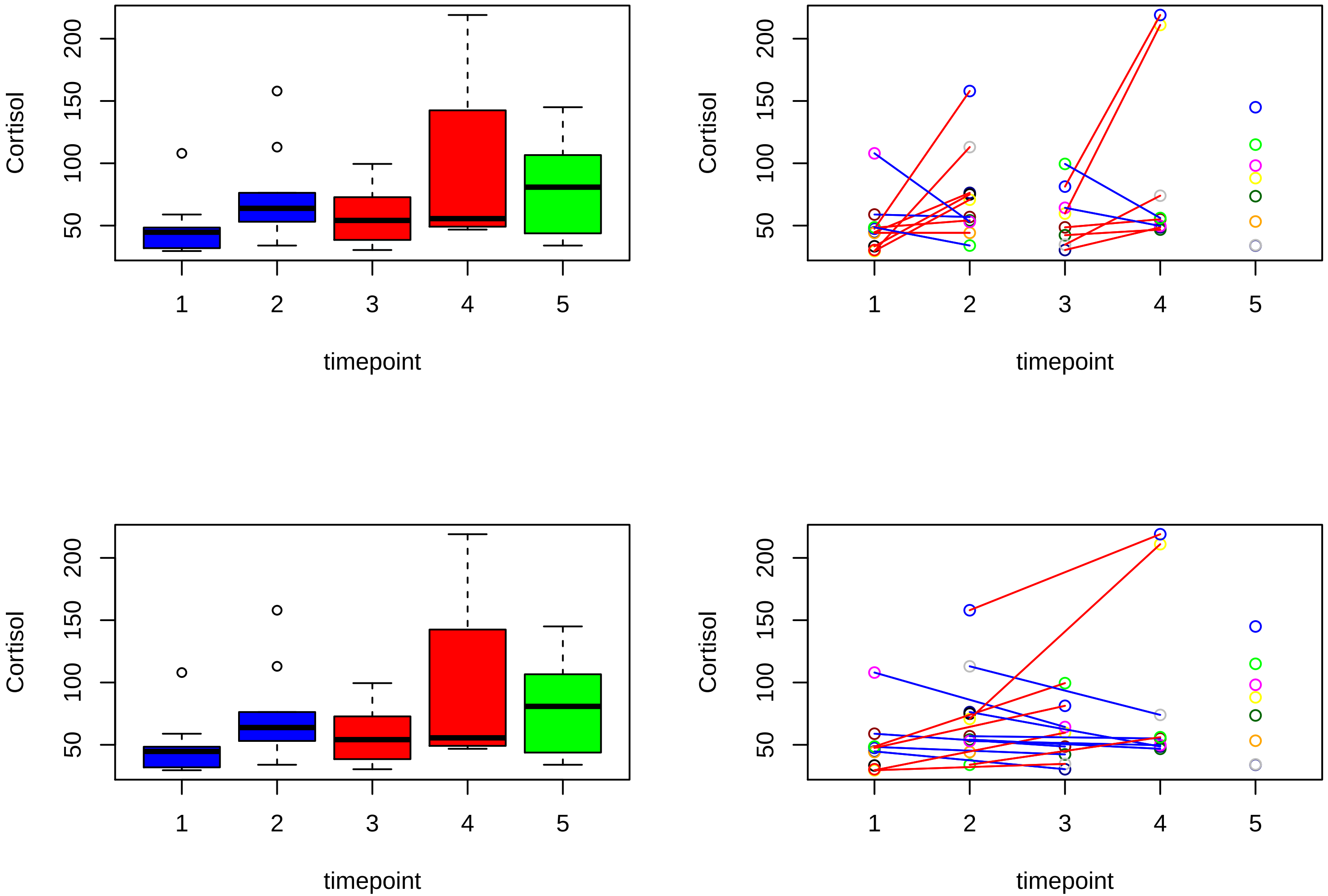

Cortisol
50
100
150
200
1
2
3
4
5
timepoint

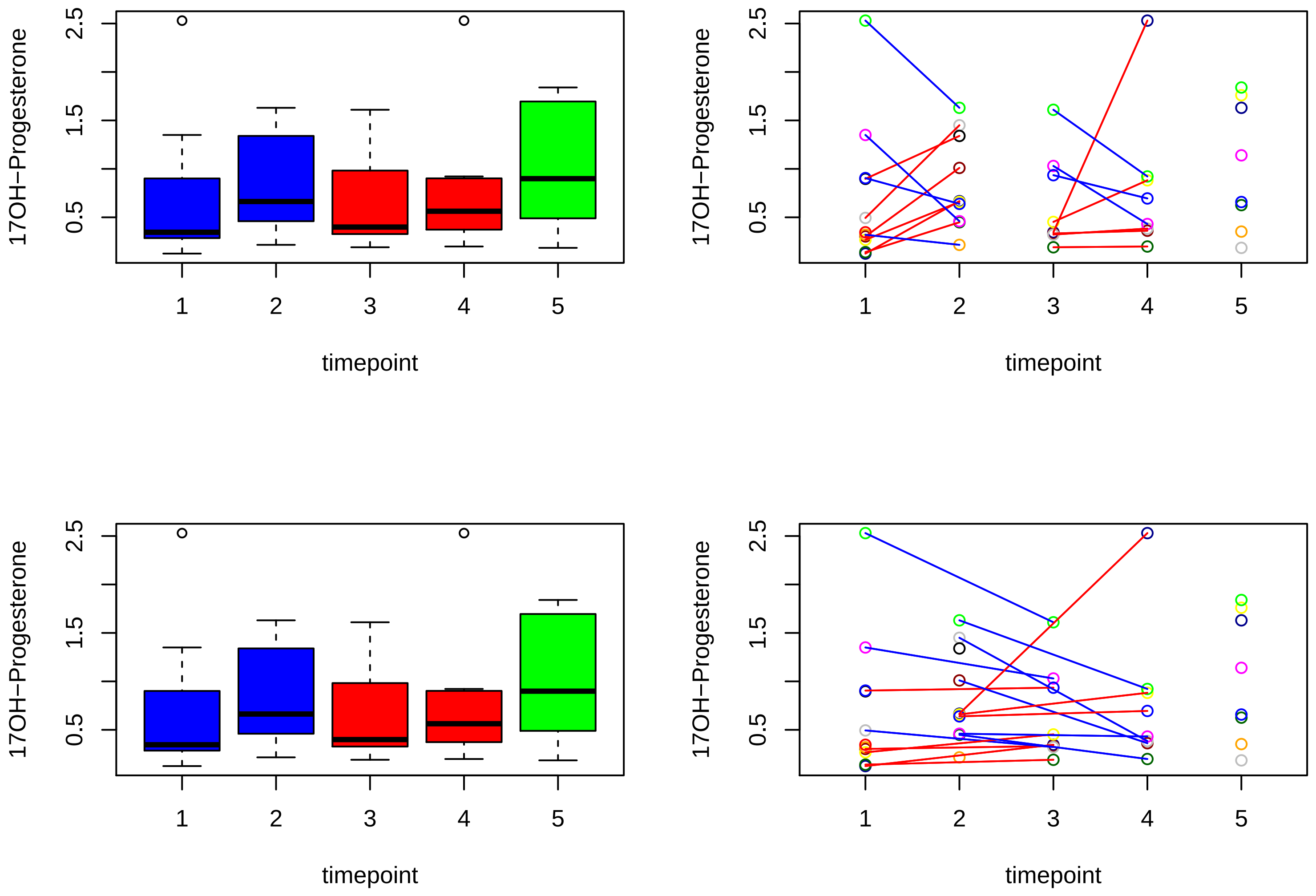
17OH−Progesterone
timepoint
0.5
1.5
2.5
1
2
3
4
5

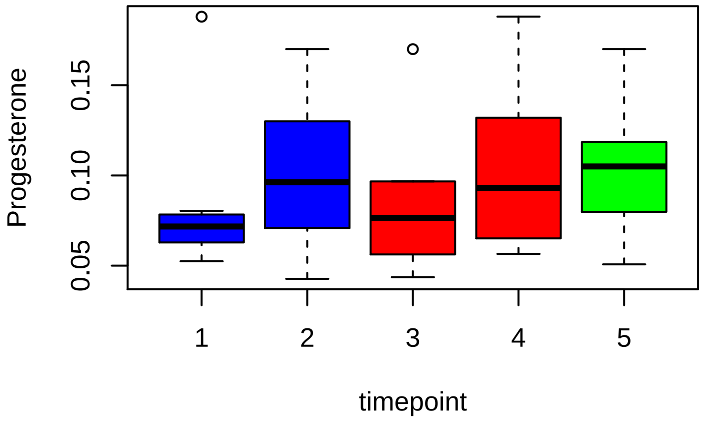

Progesterone
0.05
0.10
0.15
1
2
3
4
5
timepoint

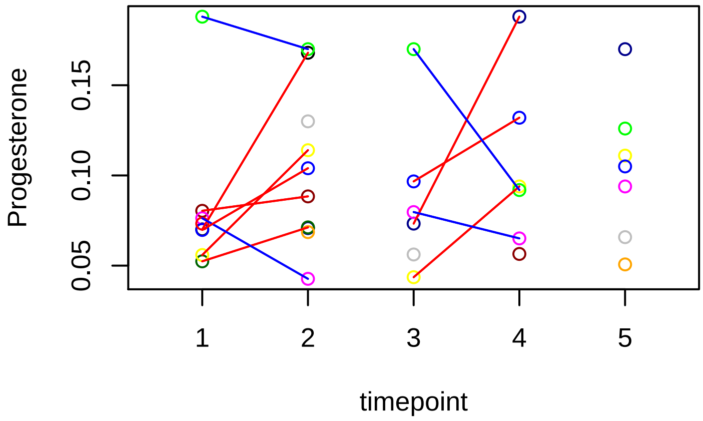

Progesterone
0.05
0.10
0.15
1
2
3
4
5
timepoint

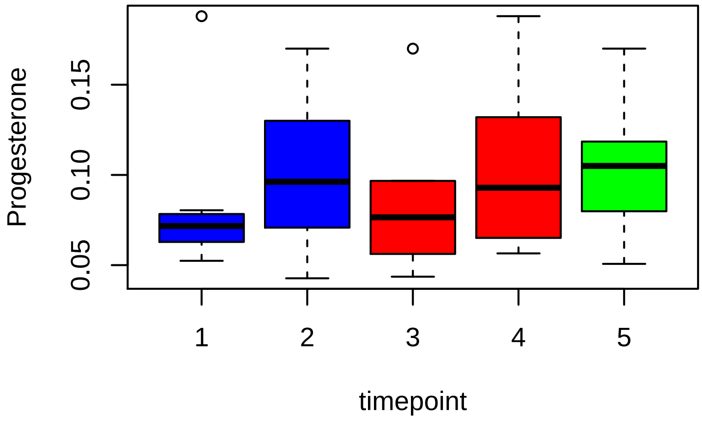

Progesterone
0.05
0.10
0.15
1
2
3
4
5
timepoint

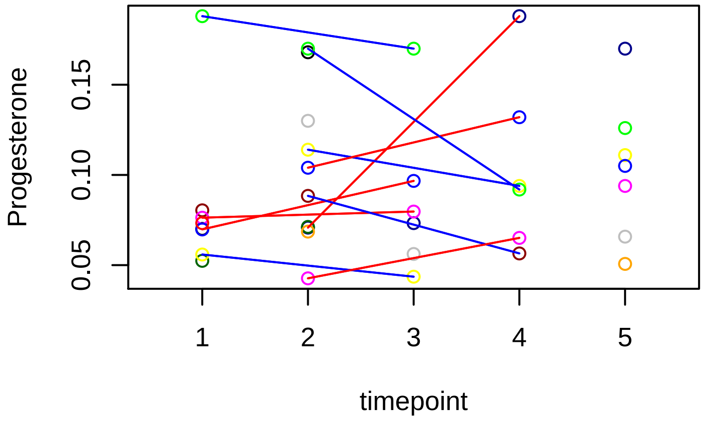

Progesterone
0.05
0.10
0.15
1
2
3
4
5
timepoint

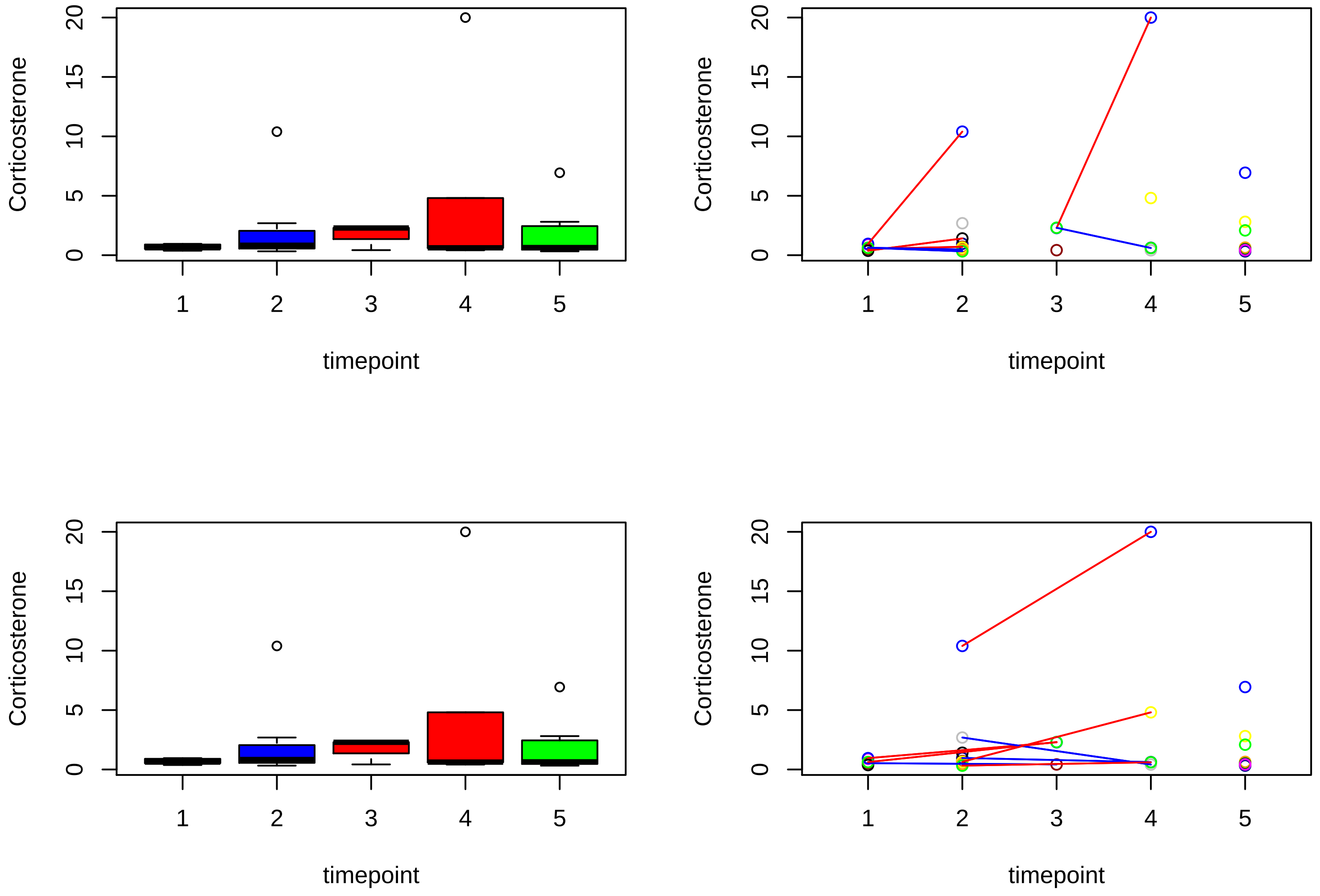

Corticosterone
0
5
10
15
20
1
2
3
4
5
timepoint
Corticosterone
0
5
10
15
20
1
2
3
4
5
timepoint
Corticosterone
0
5
10
15
20
1
2
3
4
5
timepoint
Corticosterone
0
5
10
15
20
1
2
3
4
5
timepoint

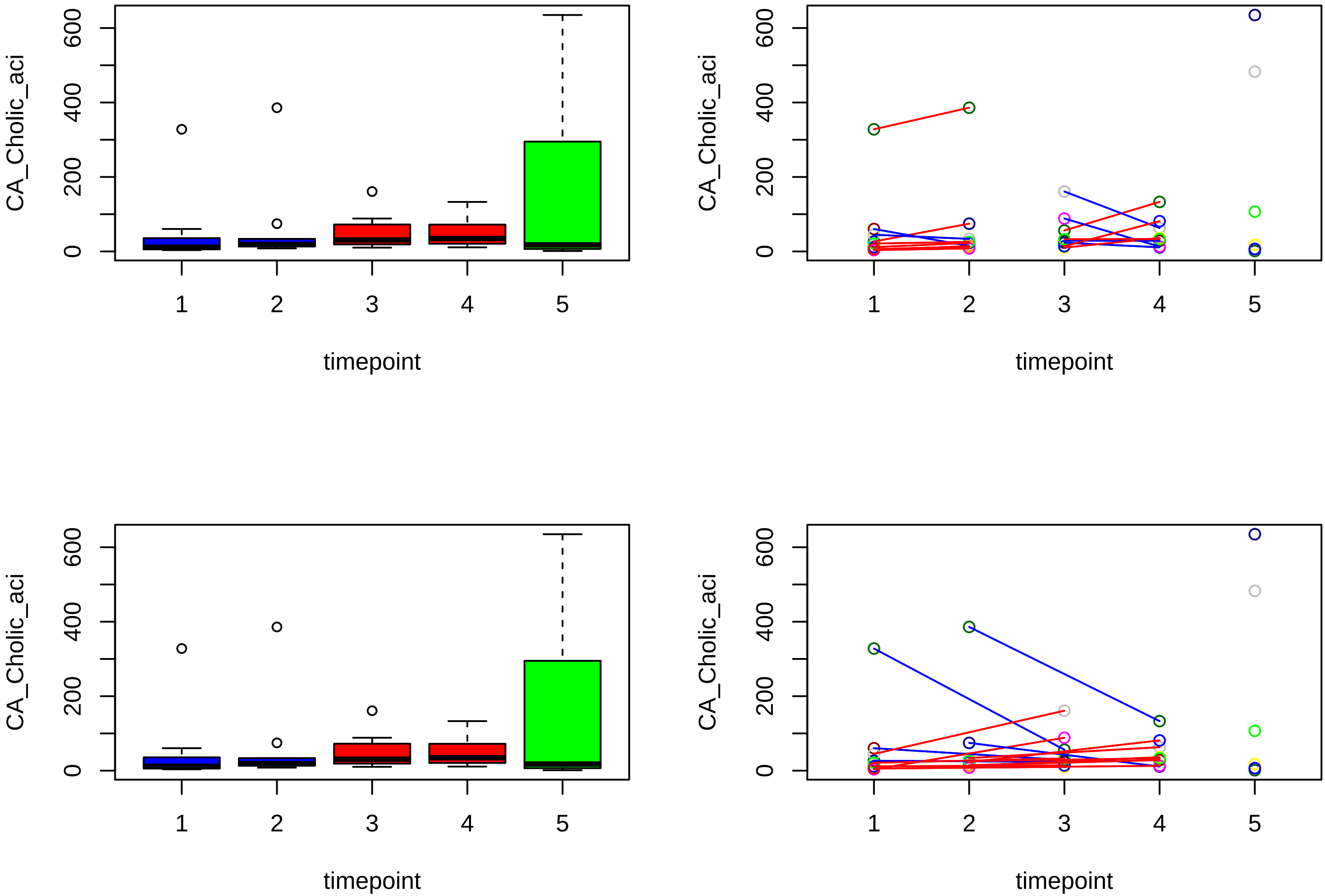
CA_Cholic_aci
timepoint
0
200
400
600
1
2
3
4
5

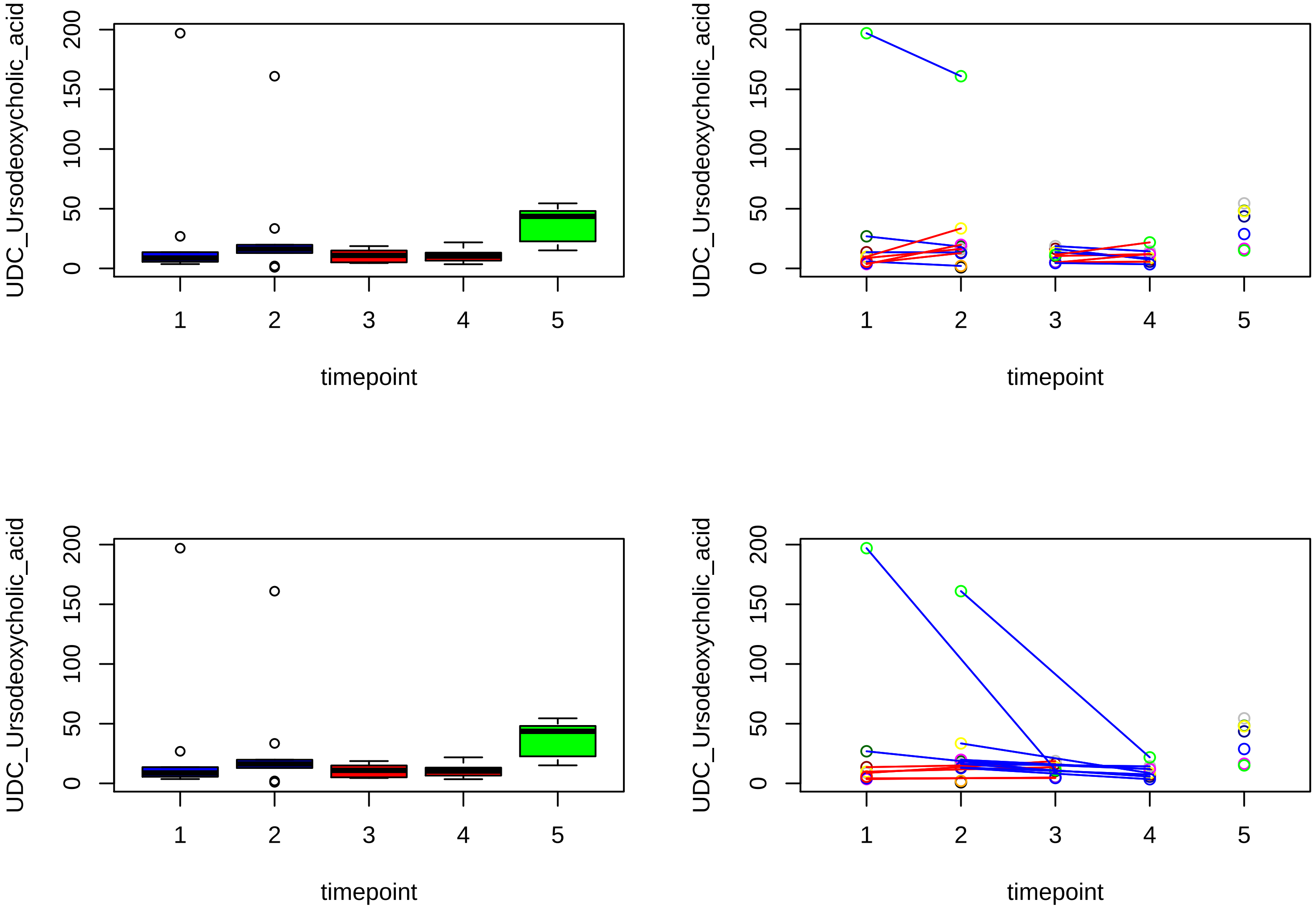
UDC_Ursodeoxycholic_acid
timepoint
UDC_Ursodeoxycholic_acid
timepoint
UDC_Ursodeoxycholic_acid
timepoint
UDC_Ursodeoxycholic_acid
timepoint

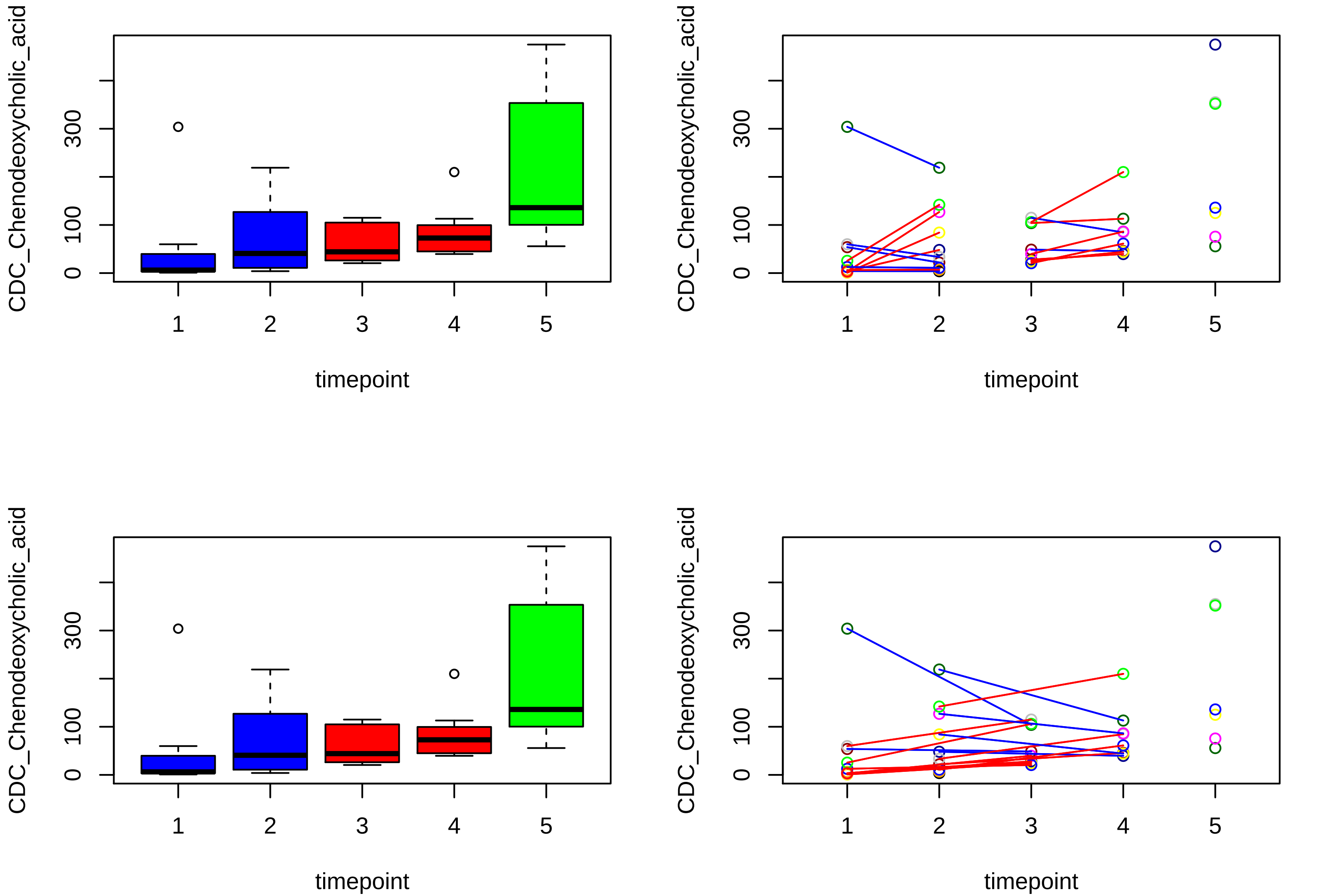
CDC_Chenodeoxycholic_acid
0
100
300
1
2
3
4
5
timepoint

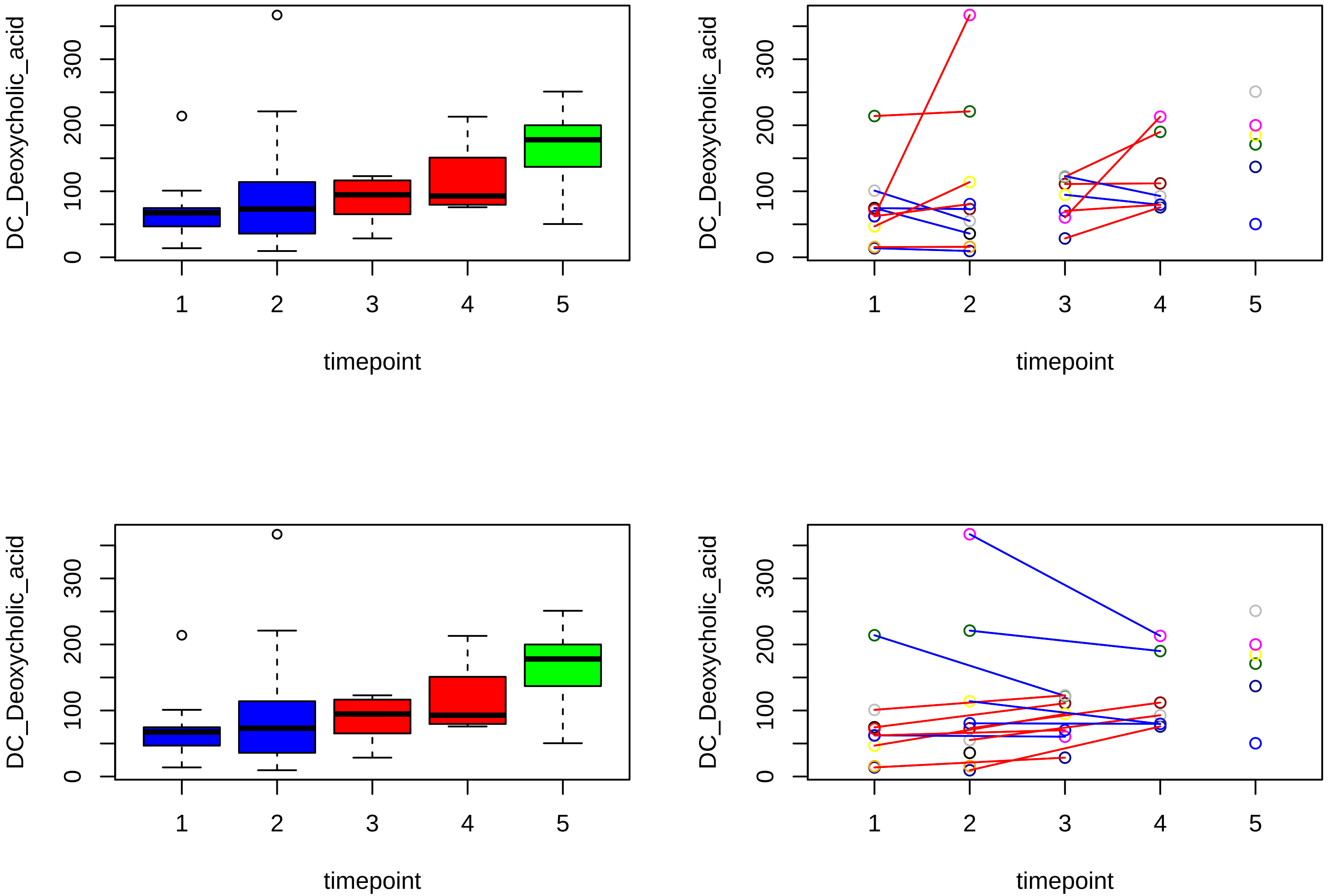
DC_Deoxycholic_acid
0
100
200
300
1
2
3
4
5
timepoint

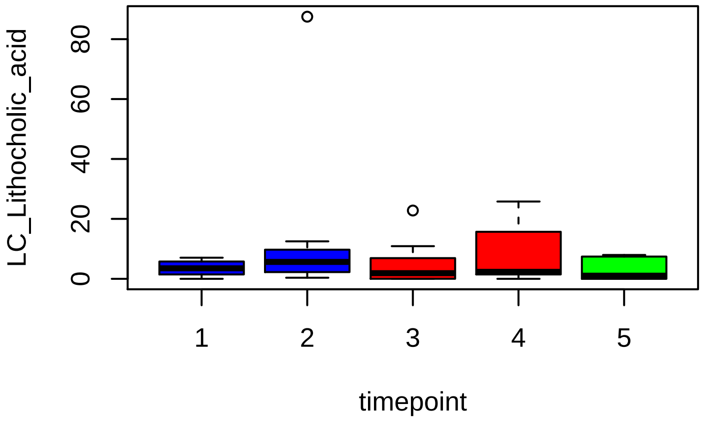
LC_Lithocholic_acid
0
20
40
60
80
1
2
3
4
5
timepoint

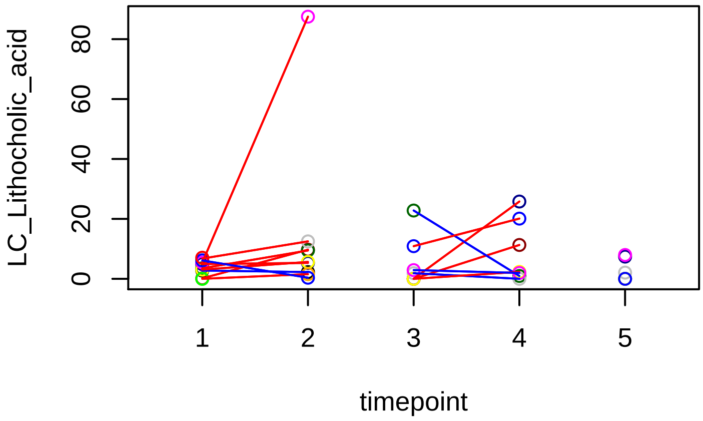
LC_Lithocholic_acid
0
20
40
60
80
1
2
3
4
5
timepoint

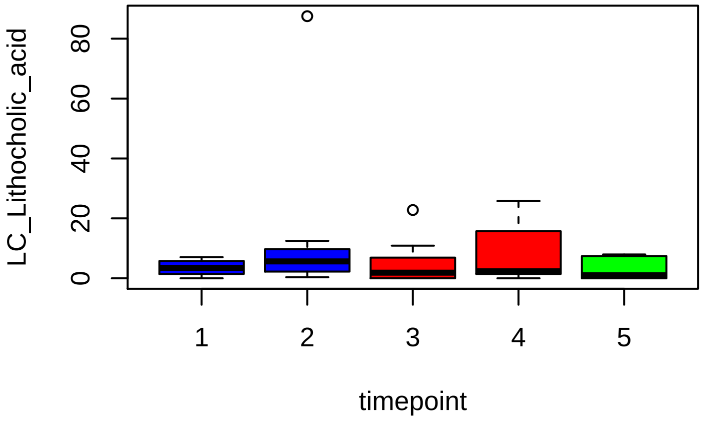
LC_Lithocholic_acid
0
20
40
60
80
1
2
3
4
5
timepoint

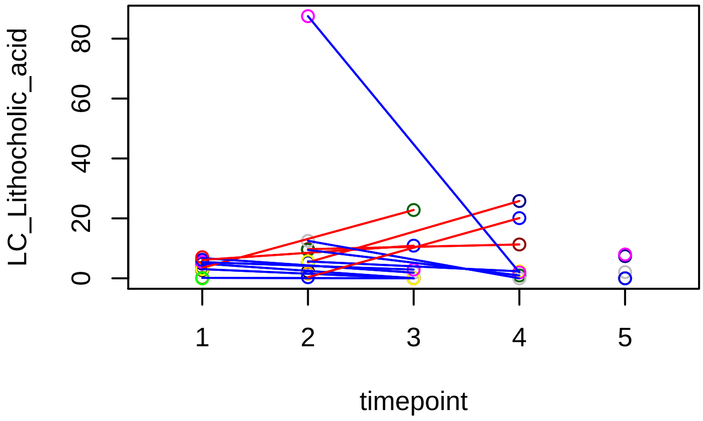
LC_Lithocholic_acid
0
20
40
60
80
1
2
3
4
5
timepoint

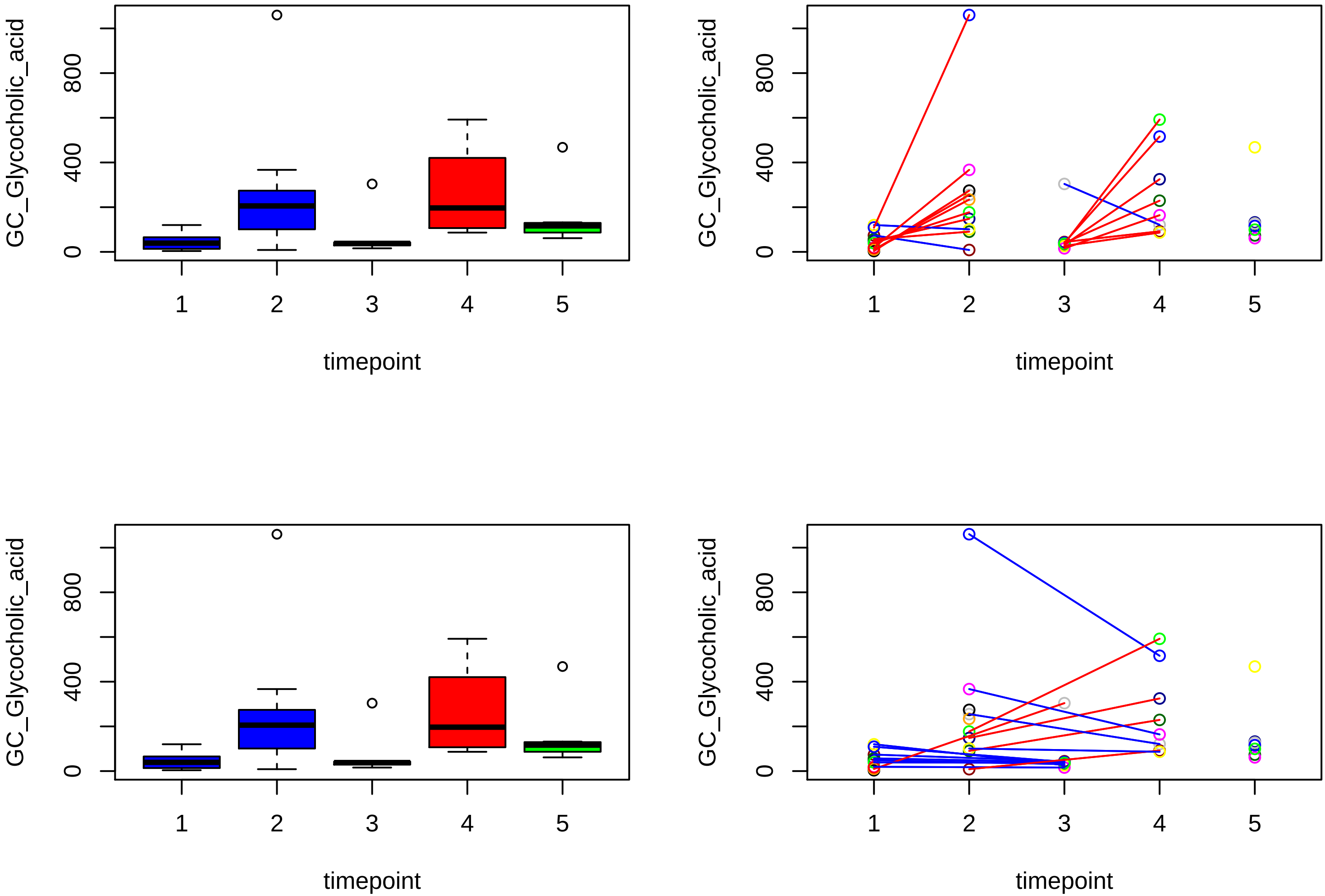

GC_Glycocholic_acid
0
400
800
1
2
3
4
5
timepoint

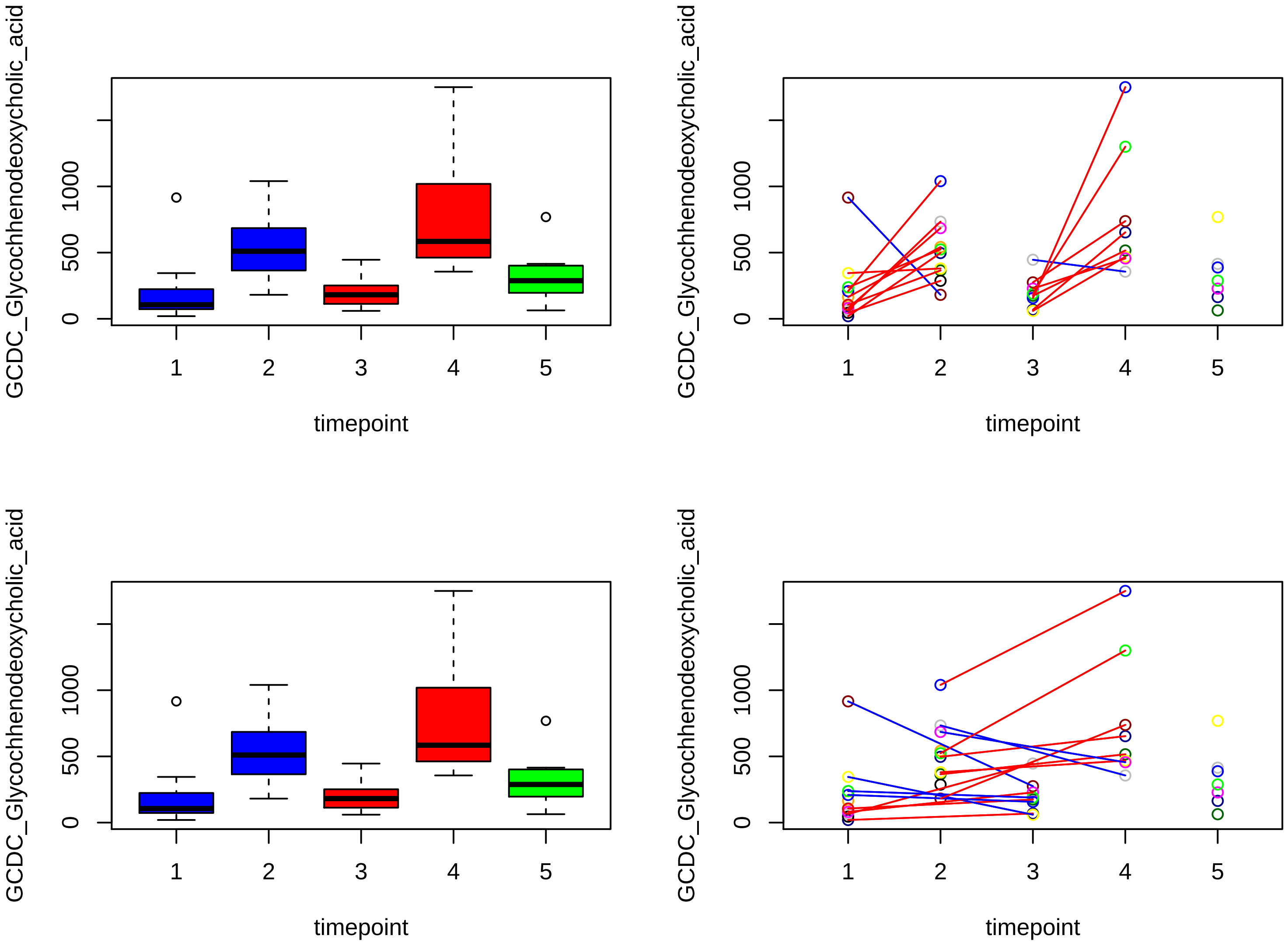
GCDC_Glycochhenodeoxycholic_acid
0
500
1000
1
2
3
4
5
timepoint
GCDC_Glycochhenodeoxycholic_acid
0
500
1000
1
2
3
4
5
timepoint
GCDC_Glycochhenodeoxycholic_acid
0
500
1000
1
2
3
4
5
timepoint
GCDC_Glycochhenodeoxycholic_acid
0
500
1000
1
2
3
4
5
timepoint

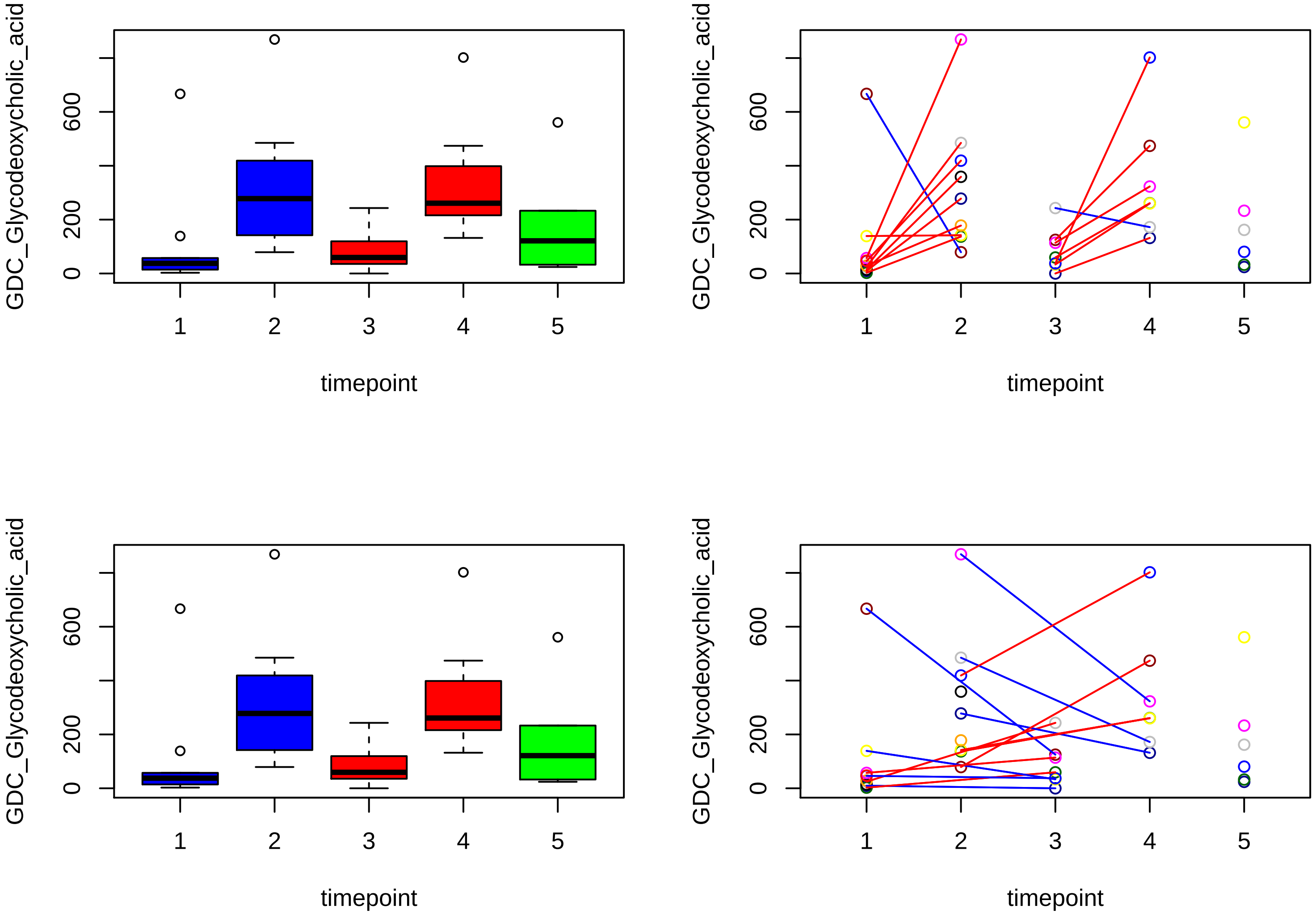
GDC_Glycodeoxycholic_acid
0
200
600
1
2
3
4
5
timepoint

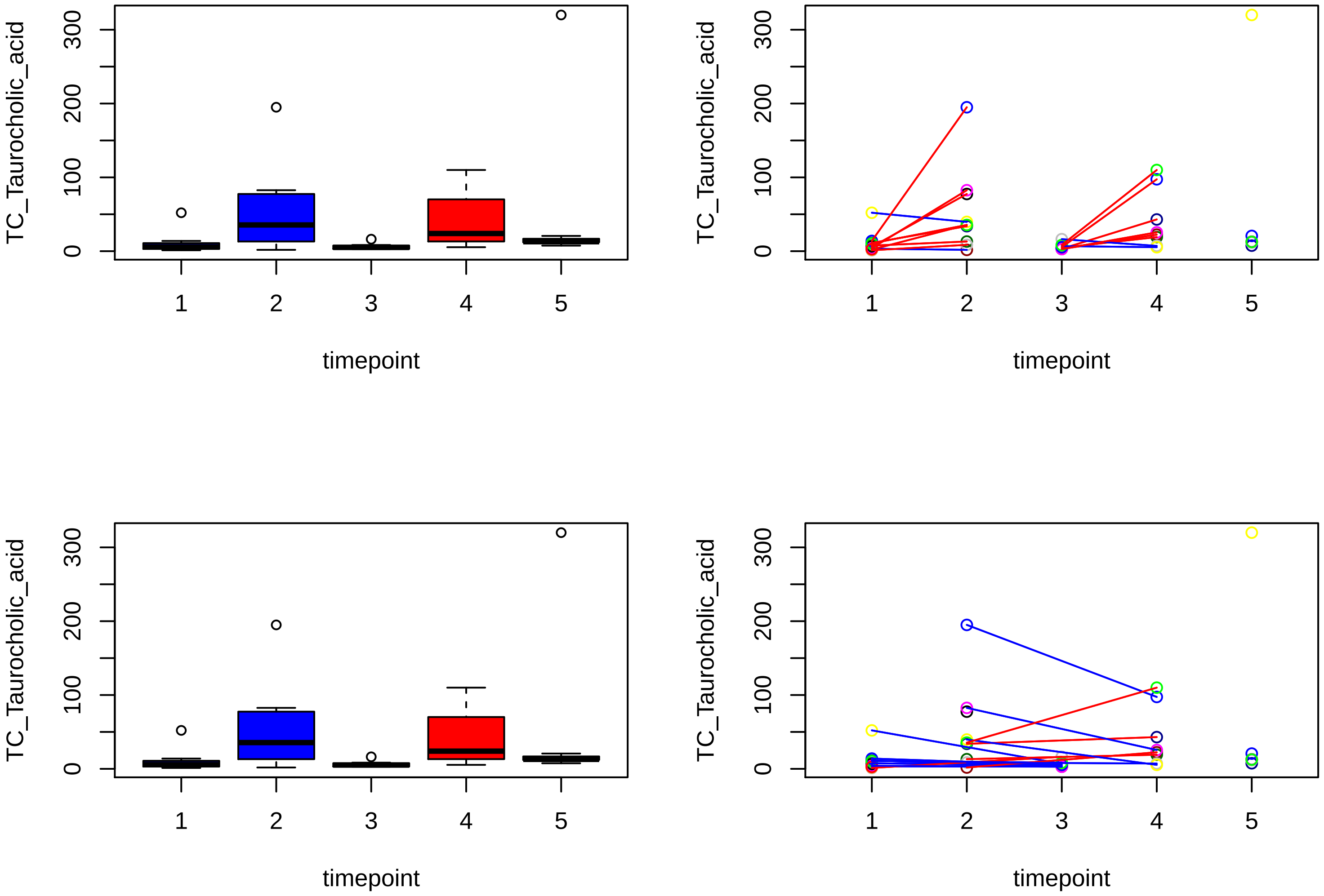

TC_Taurocholic_acid
0
100
200
300
1
2
3
4
5
timepoint
TC_Taurocholic_acid
0
100
200
300
1
2
3
4
5
timepoint
TC_Taurocholic_acid
0
100
200
300
1
2
3
4
5
timepoint
TC_Taurocholic_acid
0
100
200
300
1
2
3
4
5
timepoint

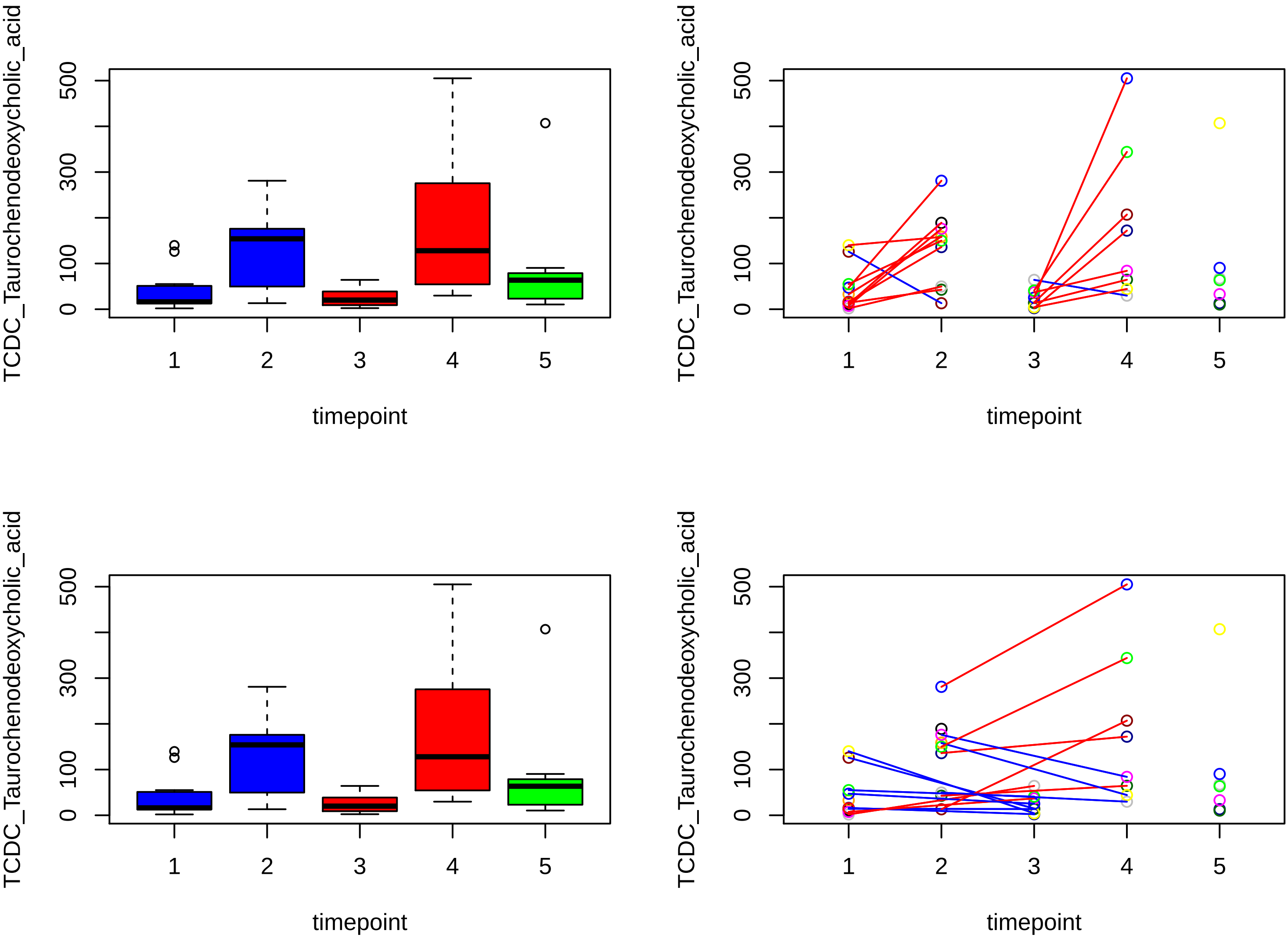

TCDC_Taurochenodeoxycholic_acid
0
100
300
500
1
2
3
4
5
timepoint
TCDC_Taurochenodeoxycholic_acid
0
100
300
500
1
2
3
4
5
timepoint
TCDC_Taurochenodeoxycholic_acid
0
100
300
500
1
2
3
4
5
timepoint
TCDC_Taurochenodeoxycholic_acid
0
100
300
500
1
2
3
4
5
timepoint

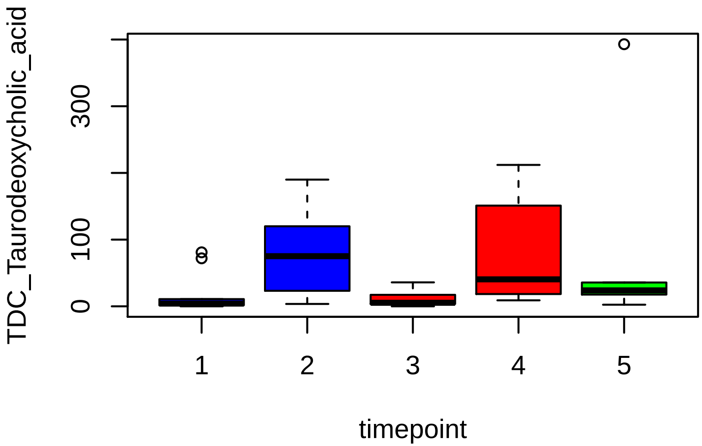
TDC_Taurodeoxycholic_acid
0
100
300
1
2
3
4
5
timepoint

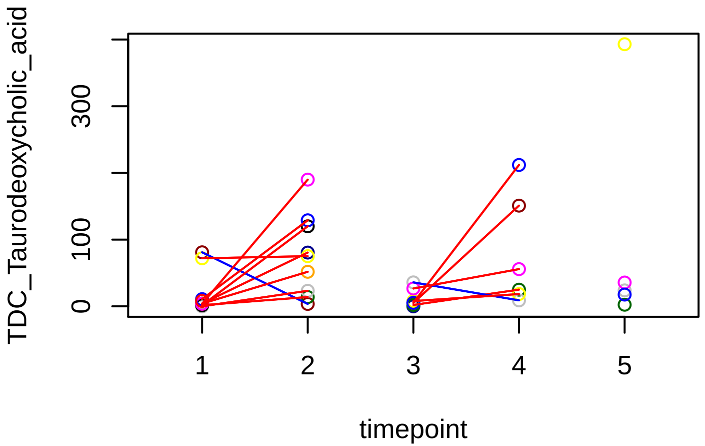
TDC_Taurodeoxycholic_acid
0
100
300
1
2
3
4
5
timepoint

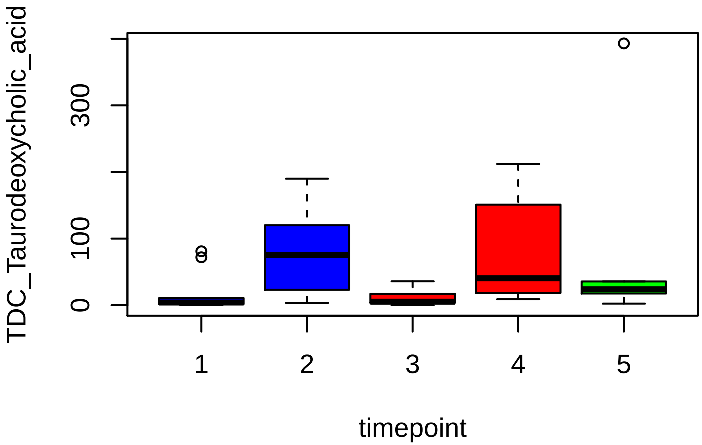
TDC_Taurodeoxycholic_acid
0
100
300
1
2
3
4
5
timepoint

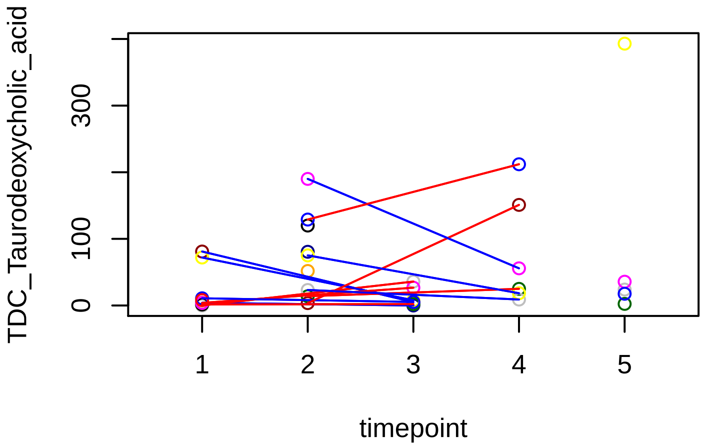
TDC_Taurodeoxycholic_acid
0
100
300
1
2
3
4
5
timepoint

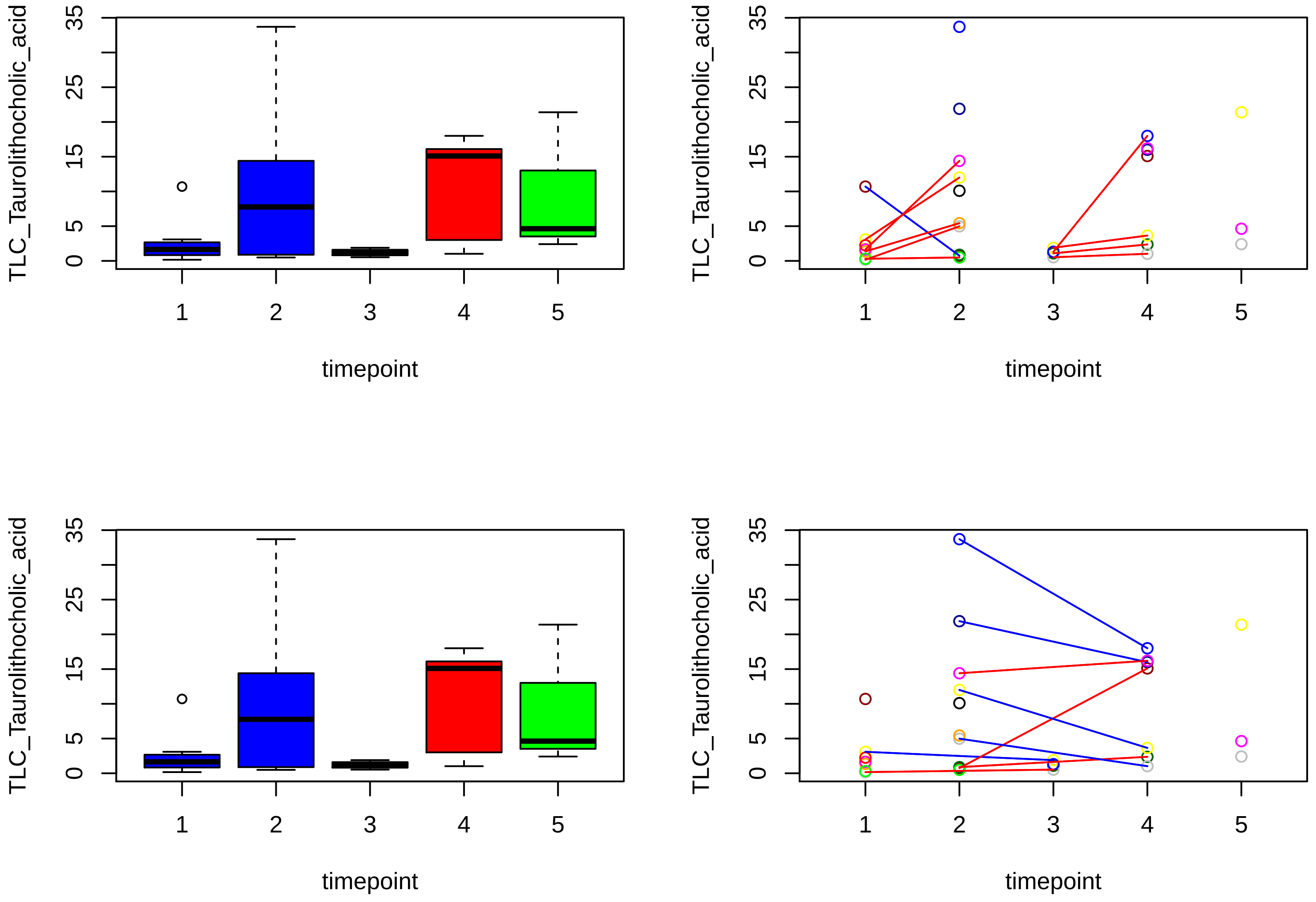
TLC_Taurolithocholic_acid
0
5
15
25
35
1
2
3
4
5
timepoint
